# Supplementary material for: Genome-Wide Analysis of lncRNA and mRNA Expression in the Uterus of Laying Hens during Aging
Source: Genes (Basel). 2023 Mar 3;14(3):639. doi: 10.3390/genes14030639 (PMC10048286; doi:10.3390/genes14030639)
Supplement: Supplementary file 1 [file genes-14-00639-s001.zip › Supplementary figures.pdf]

Supplementary material

Supplementary Fig.S1: Gene Ontology (GO) enrichment analysis of differentially expressed protein-coding genes targeted by cis-acting (<10 kb) lncRNAs (docx).

Supplementary Fig.S2: Gene Ontology (GO) enrichment analysis of differentially expressed protein-coding genes targeted by cis-acting (<100 kb) lncRNAs (docx).

Supplementary Fig.S3: Gene Ontology (GO) enrichment analysis of differentially expressed protein-coding genes targeted by trans-acting lncRNAs (docx).

Supplementary Fig.S4: Gene Ontology (GO) enrichment analysis of differentially expressed protein-coding genes (docx).

Supplementary file: Sequences of the lncRNAs (docx).

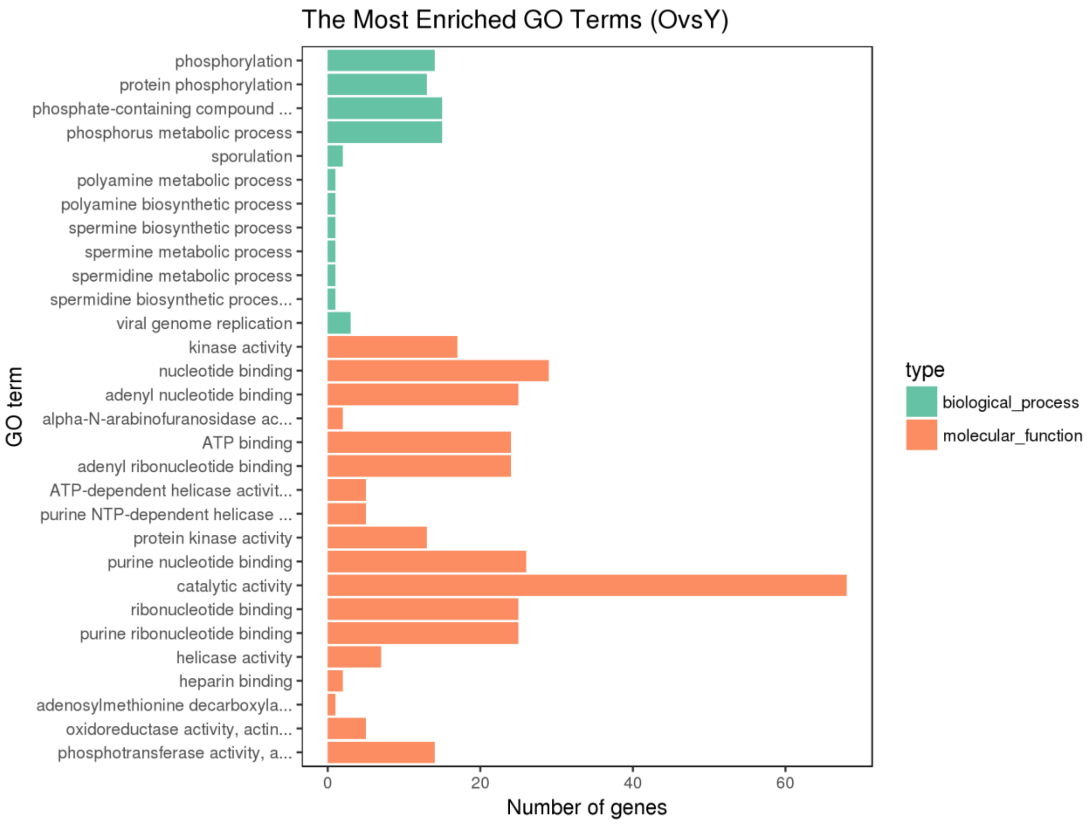

Fig.S1 GO enrichment analysis of differentially expressed protein-coding genes targeted by cis-acting (<10 kb) lncRNAs

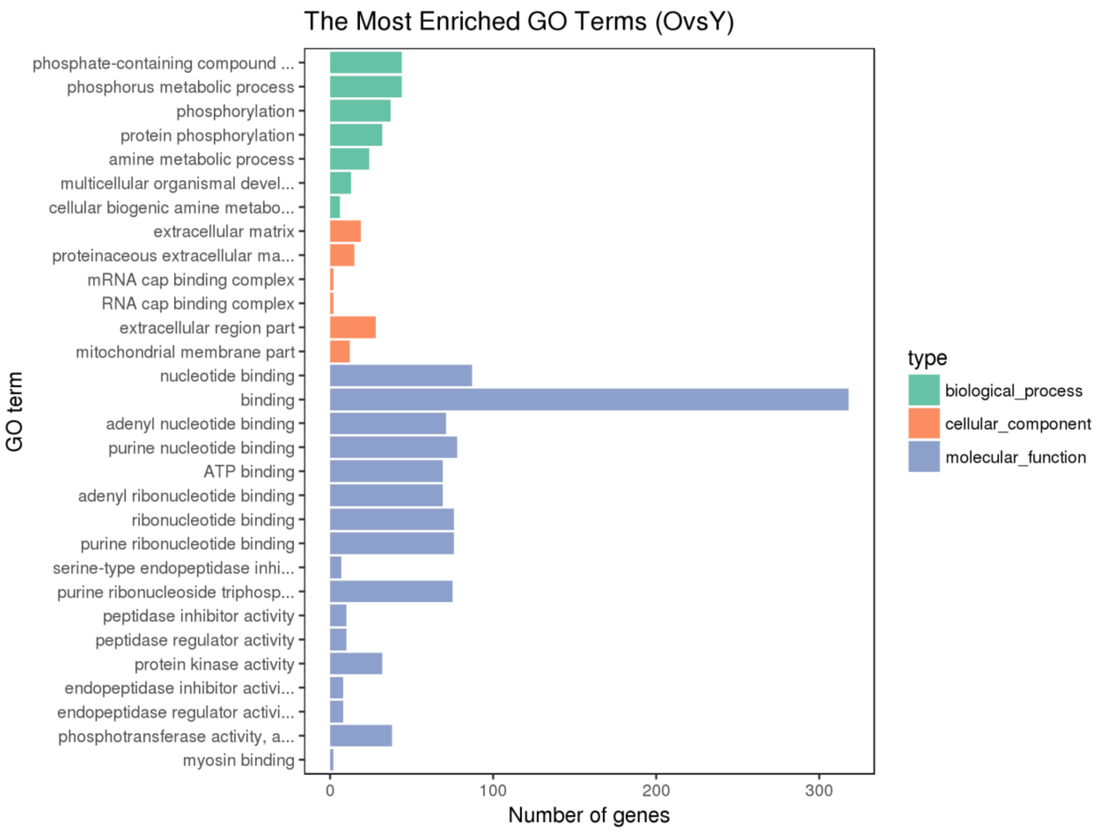

Fig.S2 Gene Ontology (GO) enrichment analysis of differentially expressed protein-coding genes targeted by cis-acting (<100 kb) lncRNAs

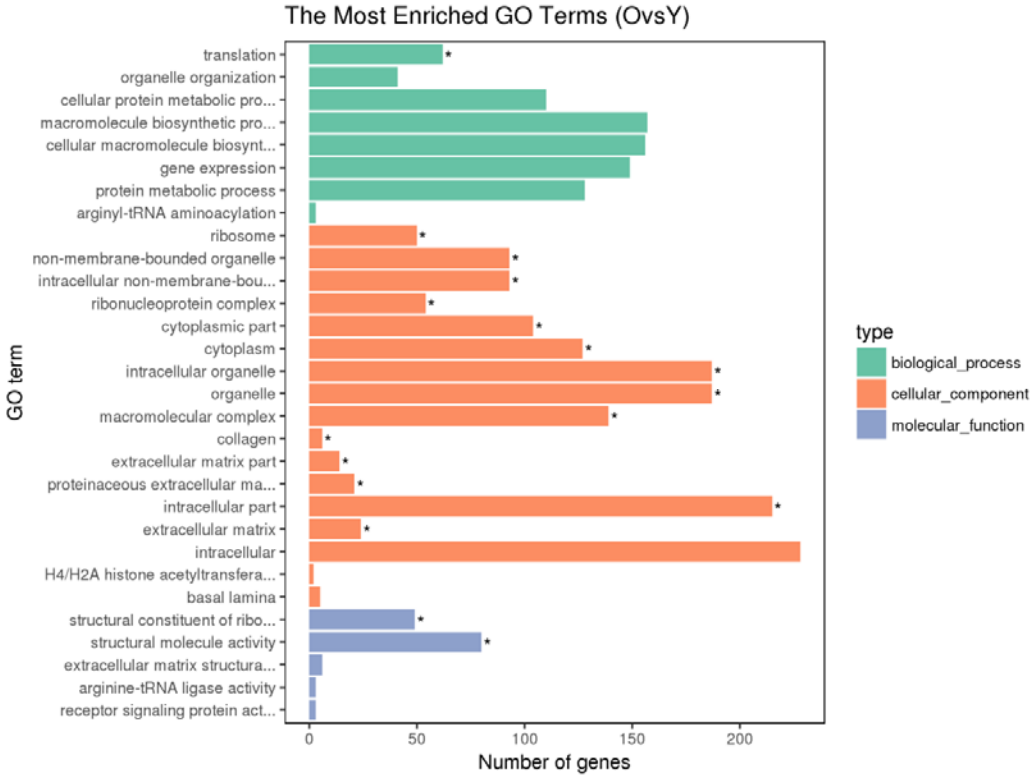

Fig.S3 GO enrichment analysis of differentially expressed protein-coding genes targeted by trans-acting lncRNAs

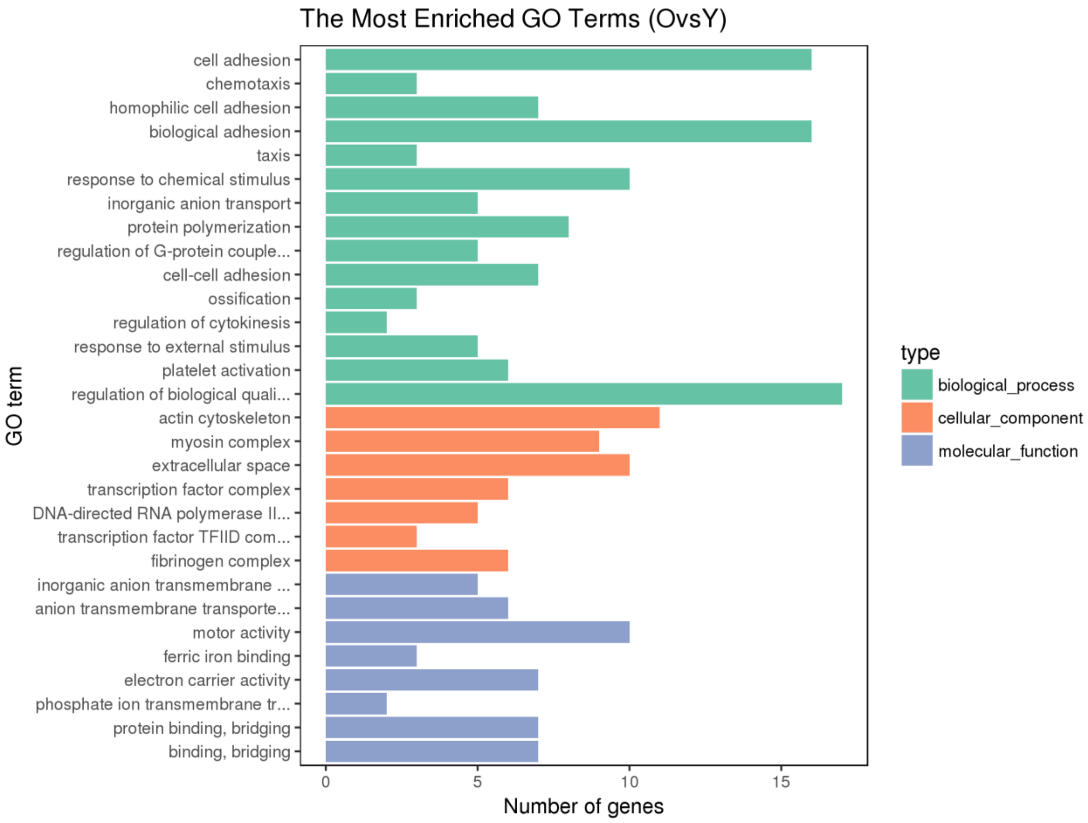

Fig.S4 GO enrichment analysis of differentially expressed protein-coding genes
