# Supplementary material for: Genome-Wide Analysis of lncRNA and mRNA Expression in the Uterus of Laying Hens during Aging
Source: Genes (Basel). 2023 Mar 3;14(3):639. doi: 10.3390/genes14030639 (PMC10048286; doi:10.3390/genes14030639)
Supplement: Supplementary file 1 [file genes-14-00639-s001.zip › Supplementary file S1.pdf]

>TCONS_04570322
CACAGCATTTCTTTCCACAGGAACCAAGGAACTCGTAAGAGAAGATAGAC
TGGGGAATGGTTCCCCAGAGTAGCTATAAGCAAGAAAGCTGTCGTAATACA
TGGGATGAAAATGGCCTtgttagaaaaatattcattggTTCAAGAGAGTAGAGAAATAC
GGTGAAAATAGACTCCTTGTTTTAGCACCAGTTTGCAACCTGAAAATGTA
CATCACCACCTATTTCCACTCCAGGACTCTTGTAGTGCATTATGACCATTAG
TCCTGGACCTTTCATTGTtgcactagaaaaaaaatgccatgcCTTTCTGTTctaattctttgctttttatg
cTAAATCAAGAGTAGCTTAAAATCAGACTTGAGCAGACCAGTAGCTGGAAC
ACTCGGAATACGTTTTTCCTTAGGCACTTCCAGATGGAAGTCACAGcaactttgc
ttttattttttttatataccAAGTTACAAACTACCCTCTAGATTAGCTCAGGCTTCAGAATC
ATTCCCAGAATCTGAATGGTGGATGAGTGAAAGTCTGAGAATTTTGTTCAG
CTCAGGAAGTGTTTGTATCTGTTTTACAACTTGAAAAAACATATACATATCT
AAAATATTGCAGCCTTCCGTGGCTCGGTCATGTGTAATtacttctgcctttcctttttaaacG
TCACTGAGTTAGAGATGCCTTTGGACATCCTGGGACTTGGGTGTCTGAAAG
GGAAAGCTGATAACAAAGACAGAATATAATGTACCCTGTGCCAAAAATTGC
ACTTCTGAGGAAAGATAACATATGGCATGCATGAGTTATTCTGATTGAAGTC
CCTGTGAAAACTGCTGGATTCTTCATCTGTAGATGCCTTGCAACAGCACAA
CAACCTCAGAGAATAAAGCCCGATTGCTTATGTGATTTTGGCAAcaataaaaagac
atttttcagtcTAGATCTTTGCAGAACTGTATAGAAAGACATGTAGGCAGCACCTG
AACTTCATaaactctgaaacaaaaatcttgGGAAGCCTTGTGGCACTCAGAGGCGTACT
GAGTTTATTGAActgaaggaaaggaacagaagcagTTTGGGCAAATGAGTAGCCTGGG
CAACTGCAAACTTAGGTCTTTTTCTCCAATGAGGTCTATTCATAAAGATCTC
CAGAGGTACTGCCTTGTTGATGGTACTTGAATTAGTTCAGTTTAAAAGATGT
GTCCCACACAGAACTAAACTTGAAAGGTTTTCAACTTAAAACACACTGAC
ACTTTCACTGTAGTCAGTGGAATTTCTGTATGTTTACTGTAAAACTGATCTTT
AAGTCCCATGTCCCAGAGCTgatttcttcattgcttttcagtGGATGGCATGGatcagtggtttttttt
aataaatttcaAAAAGTCCCAACTGCATTGTTGAGTTACCAAAAAATGGGAACTT
CAACTGCTGGTgacatggcttttttttttcagttttatatatCCAGTTTCTACCTTGCTGACCTCA
GCTGTTATCGTGATCTATGTAATTAAATCAAACCAATTTGTACATGAGAATGC
TTTACAATTAGACAGTGATTAAACACACCTTTTGCAATGTCTCCAAATTAAC
TTTTCAGATCGTAACTCTTCAGCTGTAGGCCGTTCCCTAGAAAACACCGAG
GGACAAATTAAATCTCTGAATGCACTCTTCAGTACATTtgtggttgttggtttttggtttttttt
tagtacaatAAATACtaattcttttattctttatacCAATTCTAAGAAGCAACAACAATATTTCA
GCATGGCACATACATGAAAATTGTACCAGAATATCGTGATAATTCCACAAGT
TTAACAAGTGGCAAACACAATAGAAACTGGTACGGTGGAGCACTGAATATA
TTTGTTACACAGGATAATGGTATGCACATCTAGTAAATGTCTTTTTCTGGAG
CGAAATTATATCTCAAAACAATGTTTAGTGAACCCACATGGAAAAGAGCAT
TTCTGCCATCAAAACAGGTGCTCGTAGCAGCAATTGTGATGATAAATAGATG
ATAAAAAATCCAGCCAGAATGGAACTTTTTAAGAAGAAACCATAAtgtaaggaa
aaatgaaacCCTCAGCATGTGCAGTAGAAAAGATTCAGAGACAAAAAGATGTCT
AGAGAATGACagatcaaatgaaaacagaatgtcACAGTTGTCAAaagttttgggttgtttttttttttttttt
ttgcttataaaagaaaaaggtgttaaaatgaagcaacagaattgtttcttaaattattcttttatcGTTTTTATATAATA
GAATCAAGTCAGTGTCTGTTATAACACAGAAGACAAGAGCCTCCATACGTA
CAATTCATCCAGATGCTCTTTCAAGCAAAACcacctttcattttctcattaaacACATGCAT

ACAGAATCTTCATTGGGCTACTGGAGAGGTGGTTGGATTTATTGGTGTTGTA
CTGGCTTTGTGTTTGCCCAAGAGAACTCTTGCCCAAGCCCTGGCTAATCTT
CTTGTCCATAATACTCTACAAATGAATTTTTATACAGGCATCTCACATTTAAA
GGCAGACTGTTTCACATGGGAATGTAAGGATTTGCGTGTGTCTTGGGAAAT
GAAATACAAGTTGCAGTTCAGAAAAGTCTTTAATAGCACAAAAAATTAGTT
CGTAAGACTGGAATGAAATCACTAATTGCAGCAAATGTGtgaaaaattcttttaaaaaga
cttGTACTGGTCTAATAATGTGGTTTCTTCTGGTCGCTTCCATtgctccttctctcttctcca
taTTCCTCCTTAGGTTTActaaaaaatgaaggaaacatagtaaatggggagaaaataagaaaggtaGATG
TCCCTACAGACCATTATACACAACCTCCTGTCATCTTTCAGTCACGGTCAGG
AACATTTGTCCTGAAATTAGAAGGGCTGGTGGTCAACTTACAGATTCATCC
TCATGCAAAGCTAGTGGGAAACACCGAGGACACTAAAGCATTTCACACATT
GGCTACTGAAACAGCAAGTGCTGTTCAGCTTGTTTTGGGGGTATCTCAGAT
GTCACACAGACTTCTGGGTTTCTCATTTGAGATACCCTGGGATTGCTGTTTT
CTGACAAAAGAAGGTTGTGGCCATACTACAGCTTATGGCTCCTTAGTCTGAT
ATCCAGAAGCTGtctttcagggaaaaataaagaaaagaaagaaaaagagctgatgTTTTGCTTCTAC
CCTGAATCTGAAGAAGGCACTCTGCTCCAGTTCAGGAGGCAATGCTGAGG
AGGTCAGAGGAGGGCAGGCTCACTCCCAGTGACACCACTGCTGTTGGTGT
ATGTGGCAGGCACACTGTGGTCCTCACAAGTCAACGGCAGCTGTCAGAAT
GACCCATCATCCTAAGTTTTTTGGTCTGTCTCTTTAGTTTCTTTCTAAGTGCA
AAAGTCCTCAGTGGTACTAACGTTTCTAAACATGGAAATTTCCCTTTGCAAA
TTCTGGGGAGCTAAATAACAGCAGGTAACAACATTGCTTCACTGGTGCCAT
TCAGAAATGGAGCCTGTTTGGGGAGAGGGTTGTTGGCTGCAGTTTCCTGTg
aacaaaacaatgttttaaacACAAAGCCTACCTTTTAAAAccttaatttgtattttttttttttttttttgctgcatc
tTCACACTCTCAACCAAAATACTAATTAGCACTGAGAGCTCTACATCAACAC
TTGACTGTGTTCTGGGATATGTTGATTTGTCCCAAAAGTACTAGTTATTTTAA
TTTCGGCAGCATCTCTCCTTCAGCTATTGCTCTGTCAGTCAAAGTCTAGGTA
ATATCCGAAGCAAAACAGATCTTCATCAAGctgatatattaaaaacaaatggctGCAAGC
AGAAACAAGTGAAGTAACAGTCATGACAGATACATTTAGCATATTTCTATGT
TTTATTAACCTCCTGTGTAGCAAGCTATTTGTATGCACAACTAGTTTTTATGC
ACAACTATATTGTAAGTATAattgctttaattaaaagcctcttttccattttgcacCGCTTTCCAGGT
GATGTCAAAACTGTAACTTTATAAAACACAAAGTCACATCTTAAAGTTTCTT
GCCCTTTCTTACTGCCTGTCATTTTGAATTCTATTCCAATTCAACACATGGGA
AAATAGCGGCACATCACACAGCATGAGCCATTAATTGCATTTGCACAGTACT
GTCCTGTATTTGGGATGATAGTCTCCTTGCAATACAGGCATATATAATTCCTG
TTAATAATGTTAATTTGCAAGGAAGCTCTGTCTCTCTAAGAGGGAATATGGT
GCCCTTGGGGTTCTTCTACTATCCTCAATAGTTATAGTTTCACATTAGGTACT
TTCTAATGCATACTTAGAACCTAATGCAAAAGGAACaacatgtatttttccttcttttgacTT
GATGttaaaaactaacaaaaagcAATTAGTTtttgaagaggggaaaaattaagcattttctGTATAGTGAT
GCAGCACTCCACAACAGCTAATGTATTTTCCCACTCATCTCTCATTATTCTTG
CTTTGGGCCCTTGTACTGAACTGTCCCTGCATACCAACCTTGCAATGCTTGT
AATAACAGCAATTAATCAACATTCAGCACTTACATAGGACTTGTTATGCCTA
GATctaaaaatgttacaaaaaaagTGCTGGATCAAGCTTGTGCAGTGGGTTGAATATTAG
ATGAAGAAGGCCACTTACCTTCGGGTAGCTCTCCCTCTGGTTTTATCTATCT
ATACTGGTTTTATCTTCATGgaaagggtgatggagcactggaacaggctgcccagagaggtatggagat

attcaggaccTGAgtggatgcctacctgtgcaacctattgtagggaacctgctttatcaggggggttggactcaatgatct
cttgaggtcccttccaatccccaccattctatgactgtacTGTTTAATAGAAAAAGGACAAATACAG
ACCAGTGGTATAAAAGCCAAGAAACAATTGCTTACACTAGCACCGATGTTG
CAATAGGGCTTGCCAGCACACCATTGGAGTGATTCAAAGTAGTGCCAGGG
AGAAGACTAAGAATGAAATCCCATGGAAAACTGCAGCACCCAGAAACTTG
GTATACTGCATACTGCATAGTCATTGCTGAGGAGTCAAATGGAGGAGAAAC
AACTGGGTACATACAGGTGCTACAAATGTTCTGGAGGAGAGCCTCAGTGTT
ACCAAGAAGACACAGTGTCTTCTGCAGCTCCAGTGAAGATCCCTGCCTGG
ACACCAGCCCAGCTGCCACTCATCACCACTACCAAGTCTAAGCTTGCTGGC
TTGCTGCTCTTCAGAGAAGCTAATGACAACCACATCCTCGGAGCTACTCAT
TTCTACACGGCCCCCAATACTCACTATTCTTGGTTggttttatctatttttataGTACACT
AcacttggggggggggggggtggagggaagCAACACTGTTTTAGAGTGTAGAAGTAATG
ACTTTTCTTCCATCTATTGACAATTCTTGGTGCTTATCTTTACTGTAGCTCAC
AGAGTTGAGTTTGTGTATGTATATGCCTATTAACTactatctttttcttttgtcttttggaAACT
GATCTCATTTCCCATAAGTGAATAAGTAATATGTcatatttctcatttaatatACTTGTGC
CCCTGTGGATACAGAGTGTAGGATAGATGACTCTTCCCTAGAGTCAATTATT
CTGTATGGATAAATGCGTAATTCTCCCAGGCTAAATGCCACAGGCAGAAAA
ATTCACAACTGTTATCCccagcagtaggaaaaaatcATGTTTGTTCATAGTCACGTGGA
CTTATAATATGAACTTGATTAatatgctgaaatgaaaaatgttttgtgtaatcagcaaaataatatttttgattA
TGTTTAACCTTTTCATGCATCAATGAACTGAAGTAGCTGCTAATGTAATTTG
GTGGTCTCAGAGATCAATTACGCTGTTGTAAGAGGCTTTATGTTTACATCAA
TAACAAGATTCAGgcttttctggaag
>TCONS_00006806
ctgtgatgatTGCTGTGCTATCCCAGAAGCGCTGTGGTCACCATctgttctctgctctgcccaa
CGCCCTGCGGGTCCCTGCAACGCATCTCACAAAATGCTGCACAGGAATTCA
TGCTGACCCGACACGGATTGGTGCCGTCTGTGCATGTATTGATACAGACATC
GACATACATGGACATATATCGACATACATGGACATACATGGTCATACACCCG
ACATACATAGGCATAGGTTGACATACATCGACATATGTAGTTGTACGTAGTCA
TACATCGACATACATGGACATACATCCAACATACATGGTCATAGATCAACAT
ACACTGACATACATCAACATACATCAGTATACATCAACATACATAGTTGTACA
TAGTCATGCATCGACATACATGGACATACATCCAGCATACATAGGCATAGGT
TGACATACATTGACATATGTAGTTGTACATAGTCATACATTGACATACATCCA
ACATACATGGTCATAGATCAACATACACTGACATACATCG
>TCONS_00014787
ATTTACGTGGGGTCATCCATCCATTGGGATCCGTCGTTCCCATTTCCATGTCC
ATATGGGGACATCCATCAGTTGGGATCCGTCAGTTCCCATTTCCGGGTGTGT
TTGGGGACATCCATCCATTAGGATCTGTCAGTTCCCATTTCCAGGTACAAAT
GGGGACATCCATCAGTTGGGATCCATCATTTCCCATTTATATGGGGTCATCC
GCCCACTGGGATCCGTCAGTTCCCATTTCTGGGTCTCCTTGGGGATGCAGA
TCCATTGGGATCCATCAGTTCCCATTTCCATGTTCATATGGGGACATCCATCC
ACTGGGATCCATCAGTTCCCATTTCTGGGTCTCTTTAGGGATGCTGATCCAT
TGGTATCCATTGGTTCCTCCCTTCACGTCCCTTTGGGGACATCCCAGCTCCA
GTCCCACTGTGGGACAGCTGACAGTGGGGATCCATCCGTTCCCATTTTCAG
TCCCATCGGGATCCATCCCGACACCTCTGTGTCCATTGGGATCACTTCAATC

CTATTGAGACCTCTCCATTCCCATTGGGATCCATCCCAACGTTTCCATGTCCA
TTTGGGATCACCACGATCCCATCAGGATCCATCtcaacagctctgtgtccttTGGGATCT
CCCTGGTCCTATTGGGATGACTCGTCCATCCCAAAAACTCTGTGCTCATTGG
GATTGCTCCAATCCCATTGGGATCCATCCAACCCCATTGGGATTCATCTCAA
CGCCTCCAGGTGCATTTGGGATCACTCAAATCCCATTGGGATTCATCCCAGT
ACCTCCATGTCATTTGGGATCACCATGATTCCATCGGGGTCCATCCCAACTT
CTCTGTGTCCATTGGGATTGTTCCAATCCCATTGAGATCCATCCCAACACTT
CCATGTCCTTTGGGATCTCTCCAATCCCACTGGGATCCACCCCAATGCCTTT
GTGCCCATTGGGGATCTTTCCAGTCCCATTGGGATCTCCCCAGTCCTATAAG
GATCTCTCCAACCCCATCGTGATCCCAACACTTGGGGATCACCCCAATCCCA
TTGGGATCCATCCCAACACCTCCATATCCATTGGGATCACCCCAATCCCATT
GGGATCCCAACACCTCTGCGTCCCTTGAGATCCCCCCAATCCCCTTGGGAT
GACTGATCCATCCCAACGCCTTTGTGCCCATTGGGATCACCCCAATCCCACT
GGGATCCATCCCAACACCCCTACGTCCTTTGGGATCGCTCCAATCCCATtttggt
gctgcagctgcaattTCAATCAATTCCCCACTTCGTTTCCATTTTTAAATCCTTCTCCC
CACTTAATTACCCCCTTTTTACCCCATTTCCCCACCCTGTTTCCTCATCCCGC
CCTATTTCCCCCAAGATTTACCAAATCTTCTCGGGCCGTCCCCGAGAAACCT
TACAAACTGAAGCAGGAAAATGGGTAATTTTGGGGTTGTTTTCACCtatttctgct
ggaaaaacGTGAATTGTGAAGGGTGGTTTTGGGGCGGGTTCGGTTGAATGCTAT
CGATGGACaatatcattttttattattatttttactgttttgtgtCTCTGTGATTTCTGGGGCCCTCCC
AGGGCCGCACAAAATGGGGAGAAAACACCCAGATTTGGGTCAGCTGCACA
GGACGTTGGCGCTGAACAACGCGGACTCATCACTGtgtgtggagctgcagctgctcacg
CTGGGTTTACCCGATTCTCCGTTTCTGTACCCAAAATGGGAGATAAAATTGG
GCCTTTTTCagttgtttggggtttttttttatgaaaaaaataatatag
>TCONS_00019838
CTTACTCTGAGGTTACTCAGCCCTTATTTGAGGGTTATTTGGTACTCTGTCA
CTACTCAGCCTTTACTCTGAAGTTACTCAGCCTTTCATTGAGGGTCACTCAG
TACTTCGTCTCTACTCAGCCTTTACTCTGAGGTTATTCAGCCCTTATTTGGGA
GTTTACTCCCACTTAGTGCCACTTTACTCAAACGTTACTCCCACTTCACTCC
TGGGTTACACAACAGTTCCAATCCACCTACTCCGAGGTTACTCCGAGGTTA
CTCCAAG
>TCONS_00034214
TgttcagggccccatccaacctgacctctAGGGATGGCATATCCATTATTTATGCAGAAGAAT
TTTACTCTTAATTTTATAAATTCTTCTGCACAATTGATTTAAGTGGTTGTTTG
AAGAGACAGTTTTGCTCAGTCTTTGTTTCCTTATGCTCAATTACATAACAGG
TTAGAAACTTGccatgaaataattattttattaataaaagaggttattaagaaagagaaattacTCCTTTTTG
AGGTTCTGATGACAGTATAGATAATGCTGGGAATCTTAGATTGAAAAGTGGT
TGTCAACTGTAAGCTCGCtcatagaaaacaaacaattagCTTTGTGGACAAGCATATGG
AAAATTCTGAATTGTGCACTTATTGCAGTAATTCTTGAGGTACAAGCAAAAA
TAGATACTACGTATATAAAGGTGATGGAGGGACTAGGAATCTATTATTGTTTT
TCAAGAGTACAATAAGGAAAGTATATAACACATTAGTAATGTTTTAAGGATA
TCTCCAGTTAAGAGAGTACTCCATTGTGCtaaaagtttttgttgttcttttagGTGTTTCATT
AGCAATATGTTGAAGATAACCATgcaatttgaaaatgtaaatcCAAAATGTAACTtaaatgca
tcagaaaaaaattgcattgtCGTATTTCCCCCATAACTCAAGAAGTTGTTGAATTTGTGA

AAAAGAGAAGTCTTGATTTCAATAACAGTCAATGCTACAAGGAAAATTTGA
AGTAGTGTTCTTCAAAAGATAGCAGCAAATCTTCTGACGACATTTCTTTTAT
GGGTTTCTGAAATATTCAAGCTTCTGTACATGTTCTAGTAAATGGCGGTAAG
GGGGAGAAGGTTTATTTCCCCATTCTACACAGCTAAATCCAGTCAGCAGTTA
TTTGTTACTGCTTTGTCTCTGTACATTCCTGTATCACTTGTGCAGAATGTGTG
GGAAATGCTTACAAATCCAAATAATAGTAGTTGATACTAGAATTGTT
>TCONS_00041803
ATTAGATACAAtctttttgcttatttgtttctgtgtcatTAACTGCGGGTCACAATGTACTCTTA
AGCATAAATCTGTCCACAAAACCCTGAAATGCAGCTTGCATCAGGGATGGT
GGGGTTAGGAATTCAAGGGTAAGCAGTAGCTGAGCTGGTCTAGCAATTTCA
TGAGTTGGGATTGCTTGATTCTTCTTTCTCATGCTTTTTGTATGAAATGAATA
GAtctttgtaatttaattttgttgccATTAACTTGGTGTTGAATAGAAAAGGTAAGCTTTCA
CGCATCGTAAGTACTGCCTTTATGTCCTTTATGTCCTGTTAGTTAtctgtctgtgtttttt
ctgttttgtaaatcTCCGGGTATCGTACATTATaaccaaattattttctaaaaaaatgctttgaggAAACTC
AGAGATCATCCAAATGTTTGCAGCTGTATTACCTCATCCCAAGCTGTCAAGG
TCGGGCCACCTGGGGACCCCACCTAAGTTATGGGTGCTCCAGGAAGCACT
Gatgacagcacagcaggtg GCATGCTGCCCTCTGAGTCACAACAGAGAAGTCTGTCA
CCACTGGGTACTGGCACAGTCCTCTGATAG
>TCONS_00049671
CTTAGAATCGTGCCGAGGGATCTGGAGACGTCCTAGAGAGGTTTGGGGGC
ATCCAGGGGGTTCTGGACTCATGCAGAAGAGTCTGGAGATGTccaaggaggatcc
agggagTCCCCAGGCCTGCCTGCAGCCGGACCCGGTGACACCGGAGCCCTGT
GGCGctgtcaccatcctctgtccCGAGGACGGGAGGTGAACTCTGTGCCACCAGCAG
AGGGGACCACGGGTGACATCAGTGTGGAACAGCCGTGACGCGGCGGCGTT
GTCATTGGGTGTCACCGTCACGCAGAGGGGACAAAAGGACCCCCCTGGGG
GCATTGCGTCGAAGTTGGGGACACCGTGAGCAGCTTGAGGACTTCCCAGG
GGAAGTGGGGGCACCGCGTCACCGTGTCACACCCCAAAGGTGGGGACAC
CTCTGTGTCACCCCAATGGTGTCCGACCCCAAAGCTGGGGACACTCCACA
GTGTCACACCCCAAAGTTGGGGACACCCCGACAGTGTCACATCTCAAAGT
TGGGACAGCTCTGTGTTTCCCCGATGGTGTCAGACCCCAAAGTTGGGGAC
GCCCCGACAG
>TCONS_00053177
cgatgTAAGAAGGAGGACTTATTTTACTAACGCCGATGTTGGGATGCACGATG
ACACGATATCATGCGATCTcattgggaCAACCGAAAGGGAAAAGGCCGCATGCA
GCCACATGACGTCTGTGCCGTGCTGAGTCTTCCTTCCCGGAGCGGGAGGTC
CTCTGCGATACGCTGTGCTTCTCTTCTGTCCGTGACGTTACGACGTGGAATg
ccaatagcgaagttacaaacCATGGCATCCTATAATCCTGTGGTTCTATGACCCTATGAC
CCTATGATCCgatggttctgtggttctgtggttccgtgatcctatggttctatgaccctatgatcctatgatccGATG
GTTCTGTGGTTCCGtgatcctatggttctatgaccctatgatcctatgatcctatggttctgtggttccatgatcctat
ggttctatgaccctatgatcctatggttctatTATCCTATGATCCTATGGTTGTATGGTTCTATAAT
CCTATG
>TCONS_00076711
agaaaatattttcagctggGCCAGACTAGCTGGCAAGTCTTGTTGCCCTGAGCCATCCA
TATTACTGCCCCAAATCACTCCTGGCAGAGCGATCAGCGAGAACTGCAAAA

TCTGAGTCACCACCTGAACTTCCATCCCAGATGATGTCCCATACTTCATCCT
GATTCCTTTGGTATTGCTCTTATAGTGGTGTCTGTGTATGCATTTTGGAGGAC
TGGGAGAAAACAGGTAGAGACAGATGTAGATGGCTTCCCTTAGGAGATTTG
AGACTATTTGctgtacagaaaacatttgcagttGGTGAAAGCGAGATGAATTCAACCACA
AGCAAAAGCACAAGATCCAAGGCAGTCTATACCCTCTCACTGCCAGACTAA
AGGCAAGAACTCCAAGGAAAGGAGTAGATGGGAACAAGTCcatgctcagtgctgcc
agcaaAAAGACTCTGTGCCCACTCCACCCCCAAGATACCCCTGTACAACAAG
TATGAGTCTCTGGATGTGAAATGCCCGTCTGGGGGTGATAAGAACTCATTT
GCACTAGAGAAGTCAACAAAACCAAGACCAGAAAATCCTTCCCCCAGTTT
CAAGGTTGTatcctgaaagaagaaatgggcATTATAGGTGACCCCTCCCTGCAGGGGAC
TGCACACCCAATATGCCAAGCAGGCCAACCCTCTTAGAAAagtctgctgccttcctgg
agCTTGGAGGAAGGATGTCACCGGGAAACTACCTAGTCTGGTATAGCCAAC
AGATCACGACCCATGACTGCTTTTCTATATGTTAGGTGATGAAACAGCTACA
CAAAGTTCAAGATCCATCAAAAGGGACTTCATAACCTTGAGATGTCTTCAG
AGTTGAGTGCACAAGTCACCGtcttctctgcttcctccaCTGGATAAGGACACTAAAA
GAAACAGAGTGATTCAGACAAGAAACACTTGGCTTTGTGGCACTTGTGCG
CACAATGAGGTGAAGCAACATTAAAGAGGGAAGCTAGAATTAAGATAATTA
AGATGAAATGAGATCTAAAGCACATGAAGGACAACACAGAATAAATTGAC
CAGTAAAGTATTGCACAGTTATTGCAGATGCCTAAAGTGGATCCTGGGAGT
AAACTGTTTTGGtaagaaagaaagtaaacaaaattttGTGGatcaaaaaaaaagtactgaaaaagtAAAT
CTGCCAGCTTCCTGGGATAATTCAAATGGACAAGTGACttagaaaatttgtttttatttgaa
accGTAACTACTGAGAGAAATAGAAATCTAGCTGTCTAGTTTGCCTTGAAATA
AAATGCGATCTGAAATGAGTTTGAAAATTTTACTGCAATATCTGAGATTATC
TGGTTAATTGCAGAGAAGTACCAATTAATTATTTAGTTGCCatgaaaaacacttctgtat
ATAGAGCTCAAGAAAAGTCTGCAGCCTCAATTAAACTCATTAACATACTGT
GTAGACACATTTGAATGAATCTCTGGAGACAGAGAGCAGAGACAAATGGA
AGCAAGATTTTCACCTCTGCTGATGCCAGTTCAAGTCTGTATCTGTTATGTT
CTTTGGAGTTCAAGGAGAAAAGTCACTGGCAGCTAAATTCTTGGCTTTTTT
ATTCCCCAGCTTGTTTCAGTGCAAGTATAACAGATGCAAAGATAGGATTCTG
CTGTAGGCAAGAATAAGCAGTAGTCTGCATGGTATTTACACATGGGATGTAT
CTCTGAGCTGTAGCTGTAGCCTCTGGATGGAGCCTGGAGAGAGATTCAGTT
CATGTTGAAATAGCTAGTTGGTACTACATTGCTTTCCCTGCAAATAGATACCT
AGAACAGCTTGAGATACTTCAGGGTATCTTTCCTGGTTTACAGTTATCTCTC
CAGAGGTATCCAGAAGGCCCTTCATAGGTACCATGTCATATTTTCCAGTCCC
ATGTGGCCTGGTGCTAATGAATTTCTTGGGGCTACCTTAGATGCCCTGTCAC
ATGTAAATTTGTAGTGGGCAAAAAATGAGCAAACTATGGTTCCAAAAATTT
TTTTATCAGTCCAAgtaaattaagaacaaaacagttGCTAGTCCAGATAAATGACACTCT
TCCACTAATAATGTTTGATTTATATAATCTAATTTATGGGTATGAAGTTAATAA
CACAGTTAAGATATATGGGATTTATCCTCTGGGGAGATAAATAAGGAGAGTA
TGGCAGGTAAGTCCATTGCAGACAGTCCCCTGACTGACAGGTGCTGTACA
ACAGCACATGTGGGGTGAGCAGAACTAGGATGGTGACAAGAAGACCTAGA
CTGTAGCAAATTGACTGCTAGATGGAGCATAATGTGTTCTTGTAGCTTGTGG
ACTACAAGGAATATTTTCTGGCTACACAATGTGTTAATAACAGAGTGAACCC
GTATAGCTCAGCTGGAAATTGTTCATCTGTagatttctgtatttgagaGTCTTTATTAA

AACTGCTCAACAcatgtatttgaaaatacatgtgATGATTTGAAAATACATGACTGATGAT
TCTCATTCAGCAACAGTACTGCCATCACCACTAATACTAGTAAACCTGATAT
CATATAAGATATGTTAGGAAAGTTTGGCAAATGTATTGAGTTTTTCGTAAAC
TTCTTTTGCTACCAGTGATGAAGTGGAAAGATCTTTTACATCAGATTTAAGA
ATTAAGATCAAATACTTGGAAATTTATACCCTATTCAGTTTACTAATAGTCAT
TAATATTCAGTGTAATTTGAAAGCCAGTCCCATTTCCTTGTAAAGACAGTCC
AAGTTCTAGACGAATTAATGGtctgaacagaaaaatgtagaaCGGTGTAATTATACAGA
ATGTGAATTTCAAGAGCTTTCAATTCACAAAGCAATTGTATTTTACTCTAATA
TTTTACTTGTGTTTAATATACACAAAGGATGTACATTTGAAAAGGGTGTCTG
TGAGATATATGTATTTGCACCATGAATCCTCTGTCACATGTTTAGACAATACA
GttaacaaaaaatacaattGCTGTTCCAACATTTTTCTGCCCTCCCAGACTTAGCAGG
GCATGTATTTTTATAGTTTAACAAGCTTTCCTATtagattttgaaaaatgaatctAGTTATT
GACATACTTGAGGCATATTCAATTAGTCTTGATTTGGTATATTTGATTTAAGG
GTTTTTGATTATTTGTTCCATGTTCTAACTTGACAACTTAGAGATGGGATGA
AATTAACCTGTCAGGAAGGATTAATGAACTGATTTTGTTCACTAGTTTAGAT
GATTTACACGAATTCTACTGCAGCGGATCTTGCCACTGGATCaacattcatttctgaaa
atggatTAAAGGTCAATGTTATGATATCCTATACTTTCATATACTAATTAACATTC
ATTTACcagaggaatgaaaagaaaatatgtacaGCAGTGAACTGCTCTGTAGTTTAATTAA
AAccatgttggtttttttgttgttgttggaaaaTTAACATATTAATAAATTATTAGTATATACATCA
AATACTGATCAGTGTAATTAATGGACAAAGCAATTAATTGAAAGTTAGACAT
GCCTATTTTTATTACAATAAATGTCCTATAAACTTTCTTATATACAGTACAGAA
CTACTGATTGAAAAGGTGTGCTTCACTGAGCAGCTTAGCGAGTTGTTACTA
ATAtaagaaagcacaaaaatatgAAGAAGATTATGCTATAGTCTATTAGTAAGCTACAAA
TGATTTATTAACTCAGAATGTTCAAAAATTTTCAAAGTCAGCTCAAGCTGA
GCTTGGTATAGAAATCGGACAGACGGTGTCAGTGCAGGAAGGAGGTTTGG
GTCTGCCTGAAAAATAGAGTGCTTTTGTTCCTCACCATctcagctccagctgagggGA
AAGCGGGGGGAATGCATACATTGCTCAACTCCTTTTGTTGCAAAACACATT
CATTCATATTACAACTTTAAGTTGTAACTTTGttacagatttcttctttcataGCATATGTA
AATgtgaaaatgagcagaaaacatttataataaatcttgctttctgcaaaaagccataaaaataaatggattgaATATTA
CAgaatatctttttcttcatctttgtgGGAAGTTTACAACA
>TCONS_00076851
CTGTGAGTCAGTCAGGAGCACAGTGTCAATCCATATGTACCACCGTGTGTC
AGCCCTGCCAACCAGGTCAAAGGCAGTGTTTCAGACACTGACTTGGCAAT
GATGCTGTAAATAGCAACAGTGCATATATGCCCCTACATAGCATTCTCCACG
AGGAGCAGCACAAACGTGCCCTGATTCTGAGGTGGCCCTGCAAACCAGCC
ATGTCCCAGTGCAAAACAAAGTGGGCAGGCTCACCTGTTTGCCATTCTGAG
CCAGACAGTAAGAAGTGGGCAGGCTCCTTGTTGCCCAAATTAGAAATGAAT
AGCTCTGTTTATGTGCTTTTTGCCCTTAGTTATAGCTTGCACAAGGGATCTC
AGTCTCAGGAACAGCTCACTCAGAGCATGCATTTGGTTTTGCATCAGTTCT
TCTTACCTCACCATTTACCCACTAAGCACAACAGATTTCTTTTAGCTCTAAG
GGAAGCCGGATCAGGCCAGAGAACAAGAGCAGCAATTTTCCCAGTAAGAG
TCCAGCACAGTCCAACAATGAACCAGCTTTTAATGAAGCACCACAGAATG
AACACATGAAACAAAGACATCCATTTTCCATAATGAGAGCCTGAACAATTT
CACCTACATGGTTTAAATCCTTCAAGGAGCAGGAGGATTTAGAAccacagctgtat

ttctgtccGCTTCCCTCACTCCCCCTTATACACGAGAACCCCAGAGAAGCCCAA
AGGAGGGGAGCGGGCAGATGTCTTCGCTGGACTGCAGCTGCCTGGCAAAG
CCCAGCTGATCAGGTTCCTGCTATTATTTCTGCAGGAATCTGTGCCCTAAAG
AGCAATTCCAAGGCAAGCCTCCAGGCTGGATCCTGGCCAGGCTTCATGTTG
CAGTGTTAAAGGGGCAAGTTCAGTTCAGGGTGGGGAcctttttgctttgctctgtgtttaaA
TCTGGAGTATTTTGCACTGCACAACTGTTTGAACCCCCAGGCATGCTGCAA
TGAGAAAAGGATGAAATAGAGGAAGGCAGCGGAGAAGTAACAGAAATTC
AGCAGTAAAGTAGCAGATGGGTTCCAGCAGAGttctgctTTGATTTATGTCCAG
CCAAGCACATCAGGCCCAGGTTTGGGGCCCGGAGGAGTCCAAACCAGAA
GCTGTCTGAGGAGCTCCTCACCaagagaggagcagctgaatCACCAGGCAAGTTTTG
GCAGCACTGGacaaagggagggagggggcacGACATCCTTTAGTAGCAGCCTGGGGG
ATGATAACCTCGCTCACCATCGGCATTGCACCAACAGAGCCACAAGTGAGC
ACTCCCTGCGCACCCACGCTGCTGAGGGAAAGAAACCTTTCACCCAAGCA
CCGCCACTGTTCAACAGATGTGGTTCCCGCTCCAAAGTcaaaaaaaacacccttcaGT
CCTCATAACGTGACAGGAAAAAGACCCCTCACAGGCAAGATGGGTGTTTG
TTTTAGCTAGAGTGCTTCCCCTCAGTCTTGGCTGATACAGATGGAGCCTCAG
TGGTGACTGGCACCTGCAGGGAACTTCTGCcctggaagaagggaaaaggttGTTTTCA
TGGAATGCATCCCACATCCCAGACTGATCTTGTCAGTAACATGCTGAATGGC
TCAAGGGAATCAGATAGATCTTagaacccttttttttttttttttaagtgtaaatGTTTGCCTATTA
TTAAAAAAGCAGAGTTAAATATTTTCAACCACCTGTAATGGTTTGGTAGCCA
TCCATGAACTTCCCCCTCATTCCTAGattaaaaactaaaagcagACCCAAAAGATAGC
TTTTGCTGCAGTAGGATACTCTGAGCACTTACTTTCCTGGCCCACAGTAACC
TCAGTCTGGGAAGGGGCTGCTGATATCCTTTGTATAAGGAGTCAAGTctcaggc
agaaagaaaaataatggtggCTTGGGCCCCCAAAATTATTGTGCTATTTCCTGTTCCAG
TGGTAGAAAGTACCTCTAGAGAGgcaagaagcaaaaaatatgtttcttaCAGAGCAGAGT
ACActctttttgtattaaaatagaGTCCTATCACTCATCAACTTTCAATTTGACCCAAAGC
ACAGTTTGATTTTCAATTTCTTCCTGTGTCTTGCATCGCCATATCTCTGGTTG
TTCATACTGAATTAGCTCTTGAATGCTAATGTACTCAGGGTGACATTTTGAT
GACAATCGGATACACTGCATCATATCGGGTTGAAAGTACCCCACCAGCCCT
GGTGCCTCTAGATGAAACAGAGCCGGAAAGGACTTTGCCAAGCCTCATCTT
GAGTCCTCTACGCTTTGCAACTAAAGACAGCTCATAAATGAAGATTGGGCC
TTGGCAACTCTCCGTTCATTATTGACAAATTAAGAGCTTCATAATTTCAcctctt
cttttgttctttgtgcacagacacacacatacacacttGGTGACTTGCGGTTTTTTCATCTTGACAA
ATCCTTCCTCCGGCTTCCCTCAGTTTTCAACAGCACAACAGGGAGTCAATC
AGGAAAGCTTTACAAGGCCGATAAAACGGATTTGTTTAACTGGTTTCCAGT
TCAGTCCAGAGTATTGTGAGGAAATACACAAAGGCAGTGGGAAATAATTAT
CTTTCTTCTGCAAGACCTCTTGGCCACCATTATCAAGTACAAATGTGCATAA
AATGTGGGCATTTTCAAATGTGTGCACCAACCTGGGTGCTTCAGAGATCAC
AGCTGCTCCCTGAAGGTCACAAGGTAGGGATGGACTCATTCAGATGAAACT
GAAGTTTCGTCATGCTTTCATACCTGTAATTAATTTACCAGAAAGCAGTAAG
CATTGTCTTTATAAATGGATTCTGAAGACCTGTATCAGCTGTGGTTTGCAGC
AACATTTTGGGTCTCGAGGCCACCACCTGTGACCAGTCTGCAGCTGAAAC
CATCCTGATTACACAAACTGTACCAGCGGGGTAGGAGATAAGTGCGAGCAA
AGCAGGAGAACATCAAGAAGGACTTCTAACCCAGTGCCCCACTGCTCAGG

AAGGCTCTCTCAGGCTGACAGGGCTCAGCTGTCTCCCATTTCAAAGCAAG
CTGGGCTGGCACTACTTCAGAGGTCTCCTTCTGCCATTGTGCAGCACAGAC
AGGACTGTCCTGCATGGCCATCTGAGGGAAGgggttgttttatttataagatgggaggaaaaaa
taaagttactgaattgtttttcatttggggtgaagaggaagaaaagagctctcttatttttaatatccaaAGTGCATTCA
GAGTGATTTGCtttcaaagggagaaaaggaagtttGGAAGCTGAGCTTCTACCACGACTG
AGAGTTTCTCACACATGCACGGAGATTTTTGGTCAAAAATCAAGTTCCTCA
TGAATCAAAACCAGAATTCAACTGTGTACACAAAACAGCCCCAGGAGACC
AGGGCTGAGCCAGGTTCTCAAAGCTGATCTCTGCACGCAGGAGCACAAGG
GTGGGGGCAGGAGGGTCTCTGGGGCACGTTCCCTGTGGGACAGGCTACGG
GGCCTTCTATTCACAGAAAAGGGGACCAAGCATGACAGCTTGAGAGGCTG
TTCCTGGGAGACGAGGACAGCCATCATGAGGGCATGTTCCTGTGAGACAG
GAGATGCTGGCTCAAAGCAGCCTGGTTAAATGAGGCGAGTAATGCAGAGG
GGTCATCCACATCCCACTGAGTGCGATCACCTGGTCGCAAGGGCAAGGGA
AGGATCGCCTCCTCCATTTTTTCAATCCAGCCCTTCATTGCTATGGCTTCTCT
CACCACTCAATGAGGCACTGtgtttcaggaagaaaacaaaatatttgtttgacCCAAAATGAAC
TTCAGGGCTTGAAATTCACCAATAAGCCATgacaaaaaatgtgtttttttcagtCAGTCCctcc
aatttattttcacatttatgtGTTGacagccaaacaaaaaaaataagtattcaCATAATGACATGCTGTCTTC
CACTTGTTTTTCACATGAATGGGGTTACATATTTCAATCCCTGGTCCTAAAA
ACttttccagattttattttaaaattaaatagaaatgcttGAGAGAAGAGTTAAGATTACAGTAAACA
TGGATAAGTTTTACTTAGCAACTCTGTGCCTTGGAGAGGACttaatgctatttattttattt
atctgctACACTTAGCTGGATAGATTCCACATTCCATTACAGTTTAGAAGTGATG
TATTTCATCCCGTAACAACAATTCCCAGTTAACTGTTACAATCAAATATTCTT
GTTTAAAGCCAAGCGTATACCAAAAATCATAATGCAAAGCTCATTTCCTTAA
GAATCAACGTAGTTTTATTCTGAGtttccataaaaagaaaatattactttaaaatacaaactttTAGAT
ACAGTGGAAAGCGAATTCCATTCAGATGATGTAATTAACCCCCAGTATAAAT
AATGTGTGGTGTATATTGTAATATGATAATGTGAAAGTCCAAAAGGACTCAT
TCCCAGACCACATCAGAAAATCTACTGAGTCAACAACTGTAGCAGACAATA
ACAGCACGAGTTAAAGAGCAGAGCTTTAAAGCAGGTTAGTCCAAACTGTG
CAGCATCGAAAGCGCCACTGACAAGATTAAATATGACCACTCACACTCTGG
TGTTATGTGAACTCACTCAGAAAAGACTTGATATGTAAGACCTGTCTGTGA
AGCAACAACTCGTTCCACTTGCCATGCTGAACTGCCGGACGTGGATGTACA
TTCCTATCTCTGTTTTGCATTATTGCCACCAATTTTAGAATAATTTAAAAGGA
AGACGACTTTTTTTAAcccattttcttaaattttgcTTAAATTACATTAAGAAAACATATC
TGAAGCTCACCTTCTTGCATAGAGATTCTCTGGGATGTGGCTACAGAGAAG
ATGTAAggagctgaaatgaaaaggtaTTATTGTAGCAATAAAGATGTTGTTTCAACTGT
CCTCTCggatcaaaagaaaaacagcatttcagtgggATATAAGTGACTCAATGTTATATGTAG
AAACTGCACAAAAGCCACATGGTAATGATTACTAAAAGCGCATATTTGTTC
ACTTTTTCTTGACAAGAGCCACATggcttttttaatctattgTATCACATATATGATGTA
ACAGTATATCAATGCAAATCAAACAAAAGCTAAGCAACCTTCCAGCTGAGT
GTCCAGACGCAGAAGTCAGGCAGGAACTTCTGGTTCCCATTCCCAGCTCA
ACCCTCCATGCA
>TCONS_00078817
cctgatgAGGTGGTGCTCCTCTTTCATAATTTAGTGGCCAAAGTATCGACttaggaa
acaaaaaacccatTTCAGTGCCCTCAAACCAACATTTCTTATTTGATCATGTCATCC

TCTACCAGCTTCTCTCCCTTAGTGGCACAGTTAAAGCTATGCCActgtacaaaaata
tttcagccttaatgaaaaatgagatgaaggTACACATTTATGATATCGTGTCTTACTGAGCATA
GCATTAAATGAGAGCATGGAATCCTTTaatcctttttcctgttttcagcaCAATTTCTGTAT
GTCATCTGGTGGCTATTTCAtttaaagcacaaaacaaaacttgggAAAGGGAGTGGAATC
CATATTTAATTCATTGTGCCATCAGTGATAAGCCAAGGAGTAGTTCTTCTGT
CTGTATAAcatgctgggtttttttttttgccccttcCAGATTCCAGAAAGCCCCTTCAACATTC
ACTGCAAGGTGAAAGACAAGTAGGTGGCAGCCTCCACggagaacaacagcaaaaatc
tctgaaaaaaagtaaaataatctgGGAGCCACACAGTTTGGCTTAGAGAGCTGGGAAAG
GGTAGAGCACGTTCTCCCCTCTGACTCTCACACACTCTCAGTAAGTCTTCT
AGCTTGAACGACAAGTGACTAGTGGAAAAGTGTagggaaggagctgggctggaaaaata
attcctgCTGGCTTGCCTagacagacagagaaaaatgacaTGAGAGAGACATCCTGCGCTG
ATATAAAGGCTGTAGAGAAATCCAGAAATATCAGTATGGTAGCAGAATTTTC
CTAGTGTTAGTAGTCACAGAACATGTTTGCCAGAGGAGAAAACAGGTAAA
AGCTACACTCCcatagcagaagagaaaagagccGTTATGACATCGTCTCAGAAAAAAT
GCTCAAGCAAGTATTCTCCTTACCCATATTTAGATTAAAAACCTAAAGGACC
TTGAGATCGTGACAGAGAAGGAGATCTCATGATTCCACCAACTTTCACTTT
GCTTTATGCCTTCACTGCTTTTGTACTGGAGAGGGCGAGGGACACAAACCC
TTAGTCCTTGAGAGGAAGACATCACAGGATTTTGCACTGGGTGCATGGGGA
AGTCTATCGTGAAGTGGACAGATGGAGAAAACCCTATGATTTCTGGGGACT
GTACAAGCAGTATCACATCACTGCCATCAGGCACATGCCTCAGGGCAATAA
GTTCAGGCTCTTTGTCCACATCACTTACTACCAAAATTCTCCTCACGTCACA
TGGGATGAAGAGCCCTGAGTCTGCAGGGAAGACCTGAAGGAGCAGCCTG
CAGACGATATCAAATTGTGTCgcttctgctttctcccacAGCTGTCATGGCCTCTTAGC
TTGTTCCTCTGTTTGCATCCACGCCAACAGCAGTGTCACCTGTTCTCAGTAT
TCAGTCTCTCAAGCTCTTTAAGCTCTCTGCTGAAAGATGCTCCCCAGGTGT
CAGCTCCAATCATCCCTGCGCCACACCAGTGAGGGTGCTACCCCAGTAATA
GTATGCAGAAGTTACATGGAAGTGATAAATACCTGAATGATTTAACCATTAG
TCATATGGCTATCTTTGATAATCTAAGCATAAATAGCACTGTCTTCTTGATAC
AGCCTCTGAAAGGAAGTAAAGAAGCCACTGCAGCACTTAAAGGCAAGCA
GCTACCCTATGGCCTGCCTGCCCCTGATTTCAGGAGGCCTCCAGACTGCTT
GCTGAAACCATGGCAATTTAGCTTGCCTTCTGTATTGGTGATATCTAGGCCT
AGCCACTGCCTGGCCTACTCTGAAGGTAGACCCTAGGATCGGTCATTTCTA
GACTACAAGGTAACCTGTTGTCCATGAAACCTGCAGACCAACTTCTACTTT
CAGCAGTGACATGGAAGCTGTCCTCCCCAAGCAGTCTTCCAAACAATAGTG
ACTATGCTAATGACTGGTTAACATAAGGGAGAACCACGTTATAATGGCATTA
GCTTTTAATAAATGAACAGTAGCAAATACAGTTTGCTTATGCTTTCTTATGTT
TTAGTGTGTAAGTCCTGCTGACTGCTGAGATGAGTAGAGAATTAAACTGTC
TGAGCGGCTGGGCTATTTGGTCATCTCCTTCCTCACTAGTTCTAGTAAACCT
AAATCTCTTAACTTCTCTAAAAACAAGAGAGCATCCTTGAGGACCACGTAG
TGAGTGCTACCCTGCAGTGGTTAAAATGCTGTGATGTATGTAGGGAGGTAA
ATATATATGTCTGTGTGTCTGCAACGCTGTAGAAGAGACAATTATATCCGCA
CTGTAACAGGAAGGTAGATGGCCTTTCTTGTTTGAGTACAAGTAGTCCTTTA
AGTAATAGCATGGTTGCATAATAGCACGGAAATTTAAAGCTGATCTCTGTTT
GCTCTAACAAATGTAAGGCTGGCACCTCGTCAGTTATGACTCATGTAACAA

ATAACACTTCGACCATATTGAACTGTGCGTCACCAGAAGAGCTGTTTCGTA
CTCTATTTTCTTACTTATAGAGGAAGAACAAGTAacagaatggattttttttttttttttttttttttt
tttttttggtgagcTGTGGGTTTCCATTCACTTTGACCTGAATGCAGTGTAACACGAT
ATGAGTGTAAGACTGGCTTTCTTACAAAAACACCTTACCTGAGCAACAGTG
TTAtttctaaagagaaaacatgTTCTACACACTACTCATTGCTGTAAGTACTGGCGAGG
TAACAAGGTCATAAAaatcatcaaaagaaaaagatttaggGGTTTTAATGTCACTGTTCTC
CAGTGTTTGTTAGGAAAATAACTGACTCTTTGTAacagctctctgcctttctgcaggGAG
GTCTGCAGTCCCTGCTAAGGACCACTCACCAAGAACAGACCTGGAGCAAC
CATGATTTCATCCTCACTCAAGTTCTGTGTAGAATTACTGCCCTATCATCTGT
AAAGATCTCTGATAGCCTCAACTGTGTAACATTTCCAGTTTTATTTACTggggt
gttttgttgtgttttgtttgtttgttgttttatcCTGTTtgtcagatgctattttgcTCTTAGGCAGCTTCTTGTG
TCAACTATTTTaccctttttttctgaatagcAAATCTTTTCCATACATTTACTTACTGTCAcc
atttaaaaatcacttttcccATTTAGTATCTCAGGGTACAATACTCATGCGTGTCATAAAC
GACTGCTTAGTCTGGTGTTCACTCAATACAGTTAAACTATTTTTCCAAGAGT
AGCTAAGGTATtgtcctttcttctcctttcatcCTGACAAGAgctctttctctcccctcctctctgtATATTT
GCTATAATGCAGCTCCATATTCTGCTACAAAAAGATGGGAGTGAAAGAAAA
CTCTGTGTTTTCTCCCTTGCTTAAGTTCTTCAAGAAAAAGAGTGGGTAGCC
ACCTGTGATCATTCACAAGGTTCTCCTTCACTTGTGGTAAccttacaaaaagaaaaga
ggagataCACCAAAATACAGAGGCTTTCATGGGCATTAAGAAACAGGTAGTTT
CATCCCAGACTGCTTTTAAATACatagttttcttgttttgttttcacaccCATTAGGCCTGATTG
CCTAGATTCATAAAGAAATCTAATTCTCTGAAGTATCCTAAGGGTTTGAAAT
ATTGAACTTTATTAAATTAGACCTAGAAGGAAAGCAGCCAGTATCACTTCTG
ATAGTGGGATTACTGAGTCATTATGTATTTTGGACATTTTTGCATAACTTTCA
GCTATAAAGGAGTCTGTTTAGAGAACAAGTGGTCAAAAGTATAGAAGAAAT
ACAGGTCCAAGACAATGAACAAAGTTTTCACTACTTTGGAAGGCATTGTAC
AGCTGCAGTATGCCACAATGTCTTTAAATATTGTTCTGATGCAGTCCTTTGC
AGTCCCGAGTAAATGATGCTACAGGTAAATGATGCTAGCTGTAACAGGCAA
TCAAATTACACATGGGAAAGCTTGATGCAGAATGAATGTAGATTCCTCAGT
CCTGATGAACTGCATTTCAGAGGATGTTTTAAAAACTGGCCCCAAATCACT
CATAAGTGGTTGTTGTGATTGTTGGTAATATGCAACAGAATCTCTGAGTCAC
AAAACAGCTTGAAAATTATTAAGATCTGAGTATTACTGATTTCAGCTATAGA
CTGTTTAAATCTGGACTTTGATTTGAGTTCAGAAGCTGGGAAGTGGCCCAT
GTCTCAGCTGCTCACCCTTCCCAAAGTATCAACTACAATATTGAAGAGGTTT
TAGTGTGATTGACATCCAGTCCTTCACTGCTGTTAATGTCCAGCATGTATATG
TATTATCGTATTATTAACCTTAACATCAAATCACTATCAATTTCTGTCTGTGAC
TGTAGGACAAACTGTAAAAAGAAGTGATTCATATATCTGTCCTGCTCAGTC
ACTTCTGATGACAACTGCCAGGAGAGGGATGGGATAGCTGTGGGTTAAAG
CTCAACTATCTCAGCTAAGTTGGCCAGTGACAACATGCCTAAGCCTTTTTGT
TGTAAATTTATTCCACCAGAGCTGTAGGTGTTCACGTGTCTTTCTAGAAGCT
CTAGTCAAAGAGATCGGGAGTTCCCaggtaagaaaataaacaagactCAGTAACACTG
ACAGTGTATTTCAGAGGCCTTATTGTTACAATCCTCGCGTAAAAGCTGCCAA
TgtaaaattataataataataattaaaaaaaaaaaaaaaaaggcagtgaaatgtttgaaaacagcTGATCTTGC
AAGTGACTAAGGCATTTCTCCTagtcctttttctcttttgctgtggAGTCCTGTTAAGGCTG
TAAGATAGCAGCTTTGGGCTATGCCCTTTCAAATCACACCTACCACTCATTC

CTTCCTTACTTATTCACTGCCTCATCATTCATCTGCTCCCATCTATCTGCTTGC
TAATGTCATTCACTACCTACTCCAAAATTACCTCCCATCCACTTACTGAATTC
AGACAACTAAAGGAGTTTGGGTTTTTCGTTTCTTAACTTATTTCTCTAACAC
TCCAAACAGATTCCTCTCTGAAGTGGAGCTGCAGAAACCTGTGGAGCTTG
GAAGGAACTTGCTGCAAGCACAGCCATCCCTTCCTAGCAATGCTTTAGGGT
ACGgatctgttgcccttctctgttTCCACCTTGTAACTATTGGACATGAGAAACTCCACA
GGAGTAGGATGAGCTTCTCTGCTCCCATGAGATTTTTCACTCACCGCTTTAC
AAAATGTGTGCTTAGCTCTGAAGTGTTCCAGACTGAATTAATTACTAAGCA
ACTTTGGTACCACGCTATGCACTCCTCTCAGTTGTCTTCCCACACATGATGA
GTACAACCAAGCTTGTATCCAGCAGCtctgaagaagctgaagaaatagAGAGTTTCTGT
TTCTGCCCAAAGTGACTGGGGCAATCAATCCCAACGTGGGCTCAGCTGGA
GGCATTTTCCTTCACCTCTCTACTTCTGCTATGGCTATGTGAGAAAGCAAGG
GGGTGATTTTGCACAGGACCGCAGCTGTTTTTCTCcacaataaacaaaaatcagagGC
>TCONS_00114521
CAGAAGCTGTGCTCCccttcagctgtgctccctgtgcAGACCCCGCTTTGGCAGGCATG
AAGGCTGGTTCTCCCCTACATCTCCTAGCAAGTGTAGCATCTCCAGTACTAA
ATGTTGTTCACAACATAAACCAGATAAAGCAGGTGCCAGAGATTTTGCAAG
GGTGCTTTAAGCTGCACGTGCTTTATTGGCTGATAAAGCACAAGAGAACCT
CAGGtataaagaaaacaaggtaTTTCACAGGAAGACAACTAaccttttttcttgtttctcttctacCTCT
TATTTCTCCCATAGGAAGAACGTGTTTTTCCCAAAGCAGTTTTTAAGCATCT
TACCACCTCACAGccctttttgttgttttttattctttcagtcTCTAAGGTGTGATCTCCTGCCTCC
ATCCGAGACAGCTCAGGAATCCATATGGTCCTGCCAAGGCAAAGGTCTTCA
GCTCCTCACAGATCTCTCCTCCTCTTGCTGCGGGCAGAAAGCTGGGTAGTG
TTTTTGGACTTTGCTGCTTCAAACTTGTAAAACTTGTATAGCAACTGTGAAC
TAAAACTCAAATGATACTCTAGCAAAACAAGCCTATGCTCCTCCAGCACCT
GCTCATCTTGCAaggatgggaagggaaaatgGATGGGTTTTGAGTTGTTTGAGGCACT
TCAGGAGAACGCTGGGGTTATGCAATTGTAGTGGTGCACTGTCCTCACTGG
AAGAAGATATGAGTTGGAACACTTCCCAGTGACTCCTCTTCCCTCTTTGTGT
ATCTGTGAGGCATATAAAGTCCTTGATAGGGAAGTAACTTTATGGAAATGAA
CTGCACTCAAAGCAAGATTTAGGTATTTGCTTCAAACATAAACATAAAGAA
GAGcatgcagcccagctccctACAGTGCACCAGTTAAAGCTCTGCACGTTGTACCATA
TAGCACAGCACGGCTGGCTTTGCAAATGAGGTCCCCAGAGCATCACATTCA
CCTGCAAAGGCTCAGCTCTTCAGAGGAAGTCAGGAACTAAGGAttaataaggag
aaaaaaaaaaaaaagtcctgaaaaTATGGCCATCTATCACGGTGCTACTCgtgtattttcttcctcactt
tGCTTTGCCAAAGTGTTTTGCTTAGTTTTATTTCTATGAAACAGGTTGCTTTA
TTTGTGAACAAAGCCTTGATggtctttgtttttcctccagtgcTTTTTCAGGCAAAGCTcaag
aaacattaaataaaaacacgaaactgaggagcagctgatggacTGTGGCTCTGGACCCAGAATAATT
AAGACTGAGGCTGTTATCACTTCTTTTTGTATTATGAACATTGTGATGTTAGC
GTTGATAAAATCAATGCATAcgtctttaaaaaaaaatcagaacgAAAAAACATAGAGCCT
GGATTCACCTTTCAGGGGGACTCGGTGAGAAGGAAATGATTGTTTGTCACT
CTGGATGCATCAGAAGAAGGATGTAGGTGGTTCTCTCAGAGTGACACTCTC
CCTTCAGTGCTGGGGAGGAGTGTTGTAGCAGTTCAAAGCCATGGAAATCAA
TGGAAATGTCCCTGGAGAAGCGAGACTGGGTTTGCATGGAAAGAGCATGG
ACACATTTAGGAACTTGCTCGAGCAGGCATCTGCAGGAAAGCCACTAAGT

GCACAAGCCTCTTTGCAAAGTGGATCCTTGTGTCTGATTCTCAGCATGAAA
TGACTGCATGGCTCCAGCCCAAGAACAGCACTGACATCTCCGGCTTGCAG
ACAGTTTCTGTGCATCATCTTGCCATGCTGCTGATGGGAAAGGCAGGTCAA
TTCAGTAGTGATTTCCTGGCTGGGCAATCACTGATAGAAATATTCATTCCTC
CATGTATATGTTAATATGTCTGCACTTCCCTTAGGTATGTGCAGGAATGCTGA
GCATCTTAGTGTGGCAGAGCACCCCCTCTCCCATGCCCCTTGAAATGTGC
>TCONS_00115519
GAGAACCGTCCGAATGGGAGTGGAGGGGAGCCCGTGGTGGGAGCGTGCT
CCGGTTTGGGGACGAGTGTCCCCGACGGGGCGACGCCCCCAGAGATGTTA
TCAGGGGAGAAAGGGTGTGGGAACGGGGCTTGGAGACTGCCCTGCGCGC
TGCCTGCCGTCCAGCGGCCGGGCAGGGGGTGTTGGCTGTATCGTGCAGTG
CTCGGTGGCCACGAAGCACGAGGAAAGGCCGCCGGCTGCCTCTCCCTAGC
TCCAGGCACACAGCACCGGGATGGTAGAGGGATGGTTTGAGGGGAGCGGA
CCAATTTGTTAGTGCAGCAACGGGGTCTGCCCGTGCAATGCAGCCGCAGGC
TGAAGCTGCAGCGTTTGTCCTCCTGCTGCGACTGCTGCTCCGGGAAAGCTT
TGAGGCTGGAAATGGAGGATCCCGCAGGGCTCGGTATGGCGAGATGGGTC
CTGAGTTCTGCACACGAGGTGTGTTTTGCACGCACAGGTGGTGCGTAAAG
CTTTGCCGGGTACCGGCGTCCCTCAAGAACGCTTTGCCCTCACTGTTTGCA
GTCACTGAATGGAAACGTGCGGCGGGCTGCGTCCCACAAAACCCTGCCGG
AGTGCCTGCTCAAAATACTTACTTGGCTCCTATCTTGAAGGATTTGCTCCTG
GGATGCAATTTCTGCTTCAATTGTGCTGACATTACATGGCAGTTTTGGATGT
TGAACAGCATCCTATGCCTAAGGATTCCCAGTGGAGGCACCATCACTACAA
GCAGAATTAGCTCCTCTGTGTGCAATTATGTGAGCTGTACTGGAAGtctgccctg
cagcctccctgcatGTGGATGTGCACCTTCTCTTGTGATGGAAAAACACCTACCTG
CAGCTCTCGGTAACACAAGTTGTCATCCAGGTAGTGTAAGAAAAGGCAGA
GTGCAGAGGAAAGGTGAAACATCCAAATGCCCAAATCAAAGCTCCTGGCT
ACATGTGCAGATACATGTTTTAAAGCCCATCTCTGAGAGCCTGGGAGGGGC
TGCACAGATTTTGCACATGTCCTGCTTTTGCAATGCCTGAATCAAATCACTG
CAAGTCTCACTATGGACACTTCTTGATCTCTGCTTATTGCAACCAACATatgtca
aaaaacaaaaacaaaagcaaaaacaaaacaaacaaacaaacaaaaaagccactaGCCAAACTAATTCAA
CTACTGGAAAACTGTTAGTGAAGGTTCAAGCAGTGATTTTCAGTAGTTAAA
CTATTTTAAACCCAGTTTCAGGTGAGCCTGGCCTGCAAATATCAGTGGGAA
AGGCTTTCAGTTTAATTTCCACAATTTCCTCTGCTTGCTATAGAAAGAGAAC
ATTACCTGTtattcttctaatttttttttcgGTCTCTTCAGCtagtttttctgcttcttccttcagctgcttcacTTT
CTGTAGAGTTACTTTTGGAGGTCCTTTTGCTTTTAGCTTATCTTGAAGAATG
GCGTACTTCCTTTTAATGTCAGCGAATTCCTGTAACAAAACCCCATTGCTTG
GTTTAGGAGCATCTCATAATTAACCTGGCATACCAAAgagtattaaagaaaaaaaaaaag
tgtagaaaAACCATCGGTTTGAACCAAGCTTAACATCTCTCCATTTTTTAAACCT
TAAAAACCTTATCATGAGTGCACATTGAAGAGTGCTCTGTAAAGCCGAGTA
TGAATTGAAACAAACTGCTCATGGAGGAATCTGGAGTGTCGGTGAGGATga
agatgtaaagaaaaaacagttaaagatttttaaaacttacttatgctgtttttatttcttttgatagggcttttctgtgtatttcatttttg
ccaTGAGACTTGATTTGTATGTAATGCATTACCTGTagcatcatttttttaaaacatttttttttttgg
ctggaaTATTTATTGGCTACTTCCATTTCGTGCTGGTTTAGGGTGCCTACGAGGT
TGCAGTTTAGATTTCTACTGGGTTTCTTTCAACCTGGATGCTCAGTCAAGCT

CTGCCtttggagctgtgggtgtctggTTCTCTTCACACAGCATTTAGCGATTTCCAGCT
GCCTCAATGCATTCTCAAGAAACTGGAACTACTGAAAAGCACCAAGCCAA
CCCCAACCTCCACATAACTCCCACTGGGTTTTCTCTGATGGTGAACAGCA
CGAGACAAATGGCCAGACCGCTTCCATCGCTCACATATTTTAATATCTGGTT
TTGTGATTTGCTCTGTTGTGTTTGCTGGTGCCCATGGTGACTTCGAGTCTTA
TACACAGTGCAAGGAAAAATGTCATGGAGTAGCCAGTGAAACgtgtatgttttctttc
actcaAGGAAACTGAAGTGCTAGCTGCTAGaaaccttaaaataaaaaagaaatgctacaTAACA
TAGGGAATTGTACATGGCGTGGCGTAAGGGAAACTTAGCAGAGTTTTGAGG
AGAACAAGATAACACAATTACACTTCTGAATCAATAATACTATCAAACATAT
AAGAAATCTTTTATCCTTTCATGTGAGACAGAAAGGCTTACGTTATCAATTT
TCTGAGCTTGCTTGTATGCTGCTTCTGCCCCCGCTCTCACATTTGTAGCTTG
ATTCCTGTTCATCAGCACCTTCGTCTGCAGTGTGGTAATTTCATCTTCCAGT
CTGGACTGTTTTGCTGAAAAGTCATTGAGTTCATCGCTTGTCTTGTTCATTT
GGTTCCCAGCCTGAAACAGAACAGTTGGAGGCTGTGGTTTCTCGTGCCTT
GTCCATCCCGCCTGCAGGGCTTGGTAATGAAGTAGATGAATGGCAACTGAC
AGACAAGAGGAGCCCCTTACGTGTTATGGAATTGATATTGTAtctgattttgattttatttt
acagttacGTTAAGGAAAAATTGAGATAAACTCAATAATACATTAAAAGCTTTAC
AGCAAGATACCTGTGTGATTTGTGCTCTGATTCCTTGTATCCCTTCATTTAAC
TTCATGAGGGTATTTTTGGAGCGTCCCTGGGAGCTCACAGCATTTTTTAGTT
CGTCATTAATTTTGTCCACAGGGGGAAGAGCTTTAGCTGCTTtgctaaaatggaaaa
agagaaagcacgTGTAGTATTGTGTTGTTGAGAGCAGAGCAAACGCATTTCAAAT
GCACCCGTGCTCACATGCAGAAATGTTCCCTCAAGTAGACAACAACCTTAA
AGCGTGGTTCATAAAAGCGAAACACTGGTAGGGGCCTTGTGTGGGAGAGC
ACCAactgctgtgctgaaggagaGGATGGATGCTGCGAGCTGCctggcagagcactgctggcaaG
GAGAGCTCATTTATGAGGTgaacatattttcttcctcctcagttTTGGCTGGTTCTTGCTCAC
CCATGCTTAAATCTCCCAGGGAACCAGATGCTATGATCCCCCAGGTCCCACA
TGGAGGCAGCTAGCTGCCTTGGTCTTTTGTGTTCAGCCAGCAAGACCAGG
AGCTGGTGGGAGCTACCACCTCTTGCAGCACATCAGGTCCTGCAGCCTAGG
CCACCGtggctgctcactgctggaaaacagcagtcCGAAATCCGAGCTTTCCCTCCAGAGTT
CCTAGGACACTCAGTGTCTGGGGTACGGACTGTCCTACTGCCCACAGTGCC
ATCTCAGCTGGACTTTGCACATTGTTAAGGGAACAGCTTACTCACTCAGCA
GCCCGTGCCTCCACCAGCAGTGTCTGAGCCACTTCCATTTTCCCGTTTCTCT
TATTTGCGTTCAGTTTGTACCTCTCACAGTGGGCCATAATGCTTCTGATTTTG
CCAATCATGCTTCTTAGCTCTCTCGGAGTCAGGGGAAGGTTTATCTGCAGG
ACATAACTTGCCACCTGCTCGATGTCTTCTGGAGGCACACTCTCATCTGCA
ACAGAAATCGAGCTTGAGACGTTCTGAAGCGCATGCTTCTTCCCCACAACA
TGCAAAGGATCATTATGGTTGCCTAGACATTGAGTTATGTGGCAGAATAAAG
CTGACAAGCACAGTGAGGGGGTAAGAGAATTACCTCCAACATTTTGTGATT
TAATATTATGCTTCCAGGTAGTGCTGTTAGTGTTGTCAGAACTGGCTGAATT
GAGGATTTGAACCCACTTTGAACTGGGTTATTAAAATTCTGATGCAGAACT
GAACCCACAGAGGGCTTTAAGTCGGTGCACCGCGCAGCCAGATGTGTCAG
AGCCCATTTCTCCCTTGGCCCCTAGATCAAAGAGGCACTGGGGAGACCAG
GCTGTGAGTGAGGCTTTCCAAGCCAGTCTGTGGGGGTAATCCATTCCCTGG
AGTTGCCACAGTGCTCAGAAGGAGGTTTCTTCACAGGTTAATGAGGAAGG

CTAAGCTGCAAAGAGGGATTTGGGCCAGGCTGAGATAGGGTGGGAAATCA
gagtgagaaaaatatatttagcaaCTCTCCTTCCAAGTACTCCCACTGCAATACTAGCAGT
GCTAAGAGGGGCAGGTACACTGGGTCGGAGGAACCAATGCCTTGCAACGT
CTCATCATCACATCTTTTTTCTACTCACTTCCCTTTGTGGACAAGACCTTAG
GTGATAATGCAGTTGAAACATACTATGCGTTTGTGCTAATGCTCCAAAGTAA
CTGATGGACAATTAAATAGAAGTGCAAACAGCTGTGCATTTCAGAGAGCTG
TTTTCTGTCCAAGTACATTCATCTAAAACTACCATGCGAGTCATTGTACGGT
ACAGCAGATTTGATGTGACTGTATCCATTTCATCTGCCACGGTGCATAAACT
TCAATTTACTAACAAAAAATTCACGAAGAAACGCATTTGCAGTTATTTGTAA
ATGCCATATGTCTTCTAAGAACACATGAAAGAAGGCCAAGGATGATGTTGT
CAATTGCTACCAAGCAAAAATATGAGAACACTGAGGACTTAAATGGagtttttcct
tcaaattctgCAAAATACCTTAAATGCTCCTTGACAAAGAGAGCCCTCTGTCCTC
AGCCCTCACTGCTGTTTGTATGTAGAGGCGTGAATGCTAAAGGATTGCCAG
CATGCAGCCCTTAGAGAATGAGGAGATCAGCTACATGTAAGAgattacagaaaatac
tgatgcATTTCAAGTCCTTTTAAGtgcatttgattaaaaaaaaaacctattagGCAACAAAACAT
CTTTGCAATTGCGAGGGTGGAAGAGGTTTTAGGGCATatattgttttgttatttttgtgcGG
GCTGAAGTTCTACCAGAATCCTTTGATTCCTCGAGTTACAGCTCTACTGAGA
GGACGGAGCAGTTCATCCCTTTGTGTCCTAATTCCCACGCTCCCTACAGAG
AGCCCCTATGGGCTGGGAAGAAGGGGGTGGAATAACAATTCGAGGATGCA
CTGAATATGCCTGGACATCATACACTGTATGAAATCATATTAGTAGCCCTTTA
CTCAGCTAACCCAGGAACTGAGGGAATGACTCGCCTGCTTCAATTTAAGCA
AATTAATAAAAGATGACAATTAGAACGTGGCAAATCtatgggaaaaataatagaaaatgct
GTCCTGTAGGAGAAAGAAACActagagagaaagaaattgaaCAAAAAGATGCAAAGA
TTTGGAGGGGGACAAGAAATCTGATATGTATGCAGCTCAGTCACTTTGTAG
CCATCTATTGGTACAGTACATTTGAATATAtactaaagaaaaatgagtcAGAAAGAGCT
TTCCTTGCATATTTACCTGacaagaagtttttcactttttgaTGAATTCCTTTGTGACTTCT
CGGTCGATTTTAATTTGGTTCTTAGCTCTCTCCAGTTCCCCATTGAAGTGTG
AGCCTTTCAGCTTGGTGTCTTCTGCCATTCTTCTGATGTGTTCAACCTGTAG
ATACTATGAGAAATAGCAGCGGTGTTTTCTGTACTCACTGCTTGGCAAGGT
GAGCTCTCACACTATGAGCCCCAAGTGGAGTCACTTGGCGTGGCTTTGGAA
CTCCTGTAGTAGGAGACTGCTCTGGGGAGCTCCTGAAGTTGTTACATCACG
GCCTTTGTCCAGCCTTAGAAGAAGCCTAAAtcaagttttgttttcctctgtgcaaCCACCAC
CAAACCGTTTCTAAAGGAAAAGCACTGACTTGTTTCCTGTTGCAGTGTTGT
GATATGTCATTGATCAGATTCATTACAGAAAAGGCACATAATTTAATTTGCCT
TTCTATGCTGGAATTACTGTTAAagtacacttaaaaaaaatacaagaaaactaGAAATTAATA
CTTGTCTTTAAAAGTTAAATTTTGCCATTAAATTAATATCTGTGATTATTTAAC
TTAGTGCTGAAATCCCCTGTTTCCTCAGGCAGTGAGAAAAACAATGATGTT
TCTACTTCCGTAACGTCTGTGGGAGGGCCATTCCCGCTGTCCCTGCATGTTT
GGGGGCCTCAGTTTCTCCTGACTGCTCATTTCTACACCCTCTAACTGTGCAG
TAGTGTGGTGTGGTTCTAAAAGGAGGAAATGGATGCCCAGGCACCCTGCA
GGGAGGGGACAAAGCCACTGCAGAGTGATGTTTCTATTGGAAAATGGCTTT
TGCCTATCAGTGTAACAAGCATTTTCTCTGATTGTTAATGAGAACAAACTGT
GGTTTGGGGATTTGtctgttttgcttcctttgcACATGTTGTGTTGGCCACACTTTCCTTG
AGTTATTTTCACTGCTCCTCTGCAGTAAATTGGAGCAGCTGAATACTTGATG

CCCGTATAAACATTATAGGGGGTAGAAAATATTTGGCTTGAAGTTATCTGCA
AAAATTCCTTAATGGTAAATATAAAAGTAACATAAAATTGTATACGTTGAGC
TTAATGGTATGCACAGCAATCTTAACAGCATAAGCGTGGTCCATTCTTGGGC
TGCTGGCAAATACTACCATTTGAATCTTTCTCAGTGAAAATATAGACAATATT
AACCTAATGTCACCATGTCCATTGGCACAGGACACCATGACCCGTGAGATC
TTGGCAGCTTTGAGGAAGGCATGGTTAGAGACACTGTCCCTTGGTGGGACT
GGACCCTGATACTCCTGGCACTGAATCATATGTCTGTGGCGTAGGGATGGA
GTCCATCACCAGGTCACTCACTTCTCTTTGCCCAAAGAGCAAATGAGTTTT
TGTAGATTTGGGGCTTACTACTTCATCTTTGCTCATTTTAGCTATCTACAAGT
CAGCTATCCAAATCTAAGCCAGCTGTTCATgcaatgtggggaaaaaaaataaaaataagcacc
TTCAAGTAATTAATATGGCTTCCTGAGACAAATAGCTGGGCTGGATAAAATG
AAGCTGGAG
>TCONS_00127061
TGGAAAtgcctctctgctcctgagAGGGATATAAGATTTCCTTCACTCTCCTTAGCTGC
TCTACCTAAGAGTGAGGAATGGGAGGCAGAACCATAGGGGAAGATTCTTCT
GGGCCTTGATGGAGttccagagaagaaagaaacGTCTTTCCTGGTGAAGGAAAACAT
TGGCAGCATGAATTATTGCCAATACCAACTGttgagaaaaagacaaacagtGAGGCAG
ATTTCAACAAACTAGAGATTAAAAGCGTGCCTCTGGATTGTGTTAATGGcctc
ctgctgcttcagctTGCTGTGTAGGGAAGGGCTGGAGCTGTGTGGGAAGGGATGGA
CTAGTTGTTACCTTTAATGGCTTTCTTTATCTATCACAAGGATTGAAAGTGTT
GTTTTGAAAATTGGAAGCCGATGGTCTTACTCAGTCCTCTGTCTTCAGGAC
CTGCAGTAAAAGATTTAAAGATATTTAACCACTGGATGAAATGTGCCTGGG
CACCCCGACAGAGACCAGAAATGCACAATAAAGAACAATGCTTCTGTCGT
GAAGGTCATAATCCAATACCGACACAACAATCGTCTGCACCATCTCAGCTA
ATTACATCACAGTGCTTTGAGACTTTTGATGAGGATTAAATGACATGGTgaca
gctttctgctttgataTTTCTGCTGCCTGTTCTTACATCTTCACCTGTAAATTAGAGAG
AATGCACAAAAAGGTCAACACACAGTCCAGAAGGAAAGTGATCAGTGTTC
TGTGCATTCATAGTTTTTGGTCCATATAACTGATTTTAGTCTGAAATTTCCC
TTAAGAAATGGTAGTTGGTTGCATATTAGCTAAATGGAGGCCATAGAGGTTT
ATGCCGTACCGTGGCTGTTCATTAGTCCATGTGAAATAAAGTGAGTTTGGTC
CACctgcagtgttatttttaCTGCAGATTATCTACCTGGGAAAGGCATCAAGAGAACA
AGGCTTCTGCACTACAAACATAATCTGTCTAAACTATAGCAGATTTGGAGAG
TAGAGTTATGAAATGACTCATAATCATGGCCCGTCCATGTATGctgttatttagaaaata
atttcctttttgtggGGAGAAAACTATAATGACATATGACCAAAAGTCCAGTCATTTC
AGTCACCTGTCTCCAAAAGTGGctgaaaagatattttagtCTCCAAAGATTTTAGTCTC
CAAAAATCACTATCACGTTGatctaaagaaagaaaagtgtgatATGCCTGAGTGAGAAAT
TCAGGAAAGTTATAAAAGGTCACAGTTAGCTAGGTTTGGATGTGCTATGCA
GTATGAGTAGCAGAAAGAATGACACATTAGCAGGAAATAGCATTTTGATCT
ACATCTCCATACTTCTGGTTACACATCttaagaagacaaaaataaaatggagttGGTTGAAG
GAAGCCCACTTTGGGATTAGGTGTCTATAAGATCTCCATAGGTACCTCTCCC
CTACAGCCCCCTATCCTACAACTCACTTTAACAGTCAGAAAGTGATGACCA
AGTGCCTGTTGAACGCTGAACACTGAgcatctctgcctgcagagAGGCTAATTTCAA
GCCAGCAAGTACATTTCcaggaaaaacatctttattaatgtatCCACCATCCTGTGGCCTCT
TTTCTGAAGCTTGTTGATGAGTTTTTCAAGAGCTGTTAACATCCAGATGTCT

CCTGTCTCAACTGAGAGAGCTTTCTGGAGAGCTTTCCTAGCTTGCACTTTT
GTGGTTCATAGCTGTGACATGAAACATCTTTCATGATGGAAAGGAACGTCC
CTTCATGTGACCAGCAGGCCAACACTCTGTGGATCATTAGGCAGCCCAGCA
TAGGAGCATTGTGTTGAGGAACACAGCGGAGAGTACAGTAATGGACACTT
CCGATCTGGCATATCCTAAATACTGAATACTTGGATAATTTATGCTTATGCATA
CATTCCTTGGtgagagaacaaaacaagatcTGATAATGTTTCTCTTAAGCAAGTCTCAA
CAAAATCATAGCTCTTGCTTGACAAGTAATGAGactgctgtttttcctcctcctttttgaAGG
CTTCATATGAATTTTTGTAGTCTTGGGTTATTGCCCTGGGGAATGGAATATAA
TTTGAGTGACTGAATGTACTGTCTTTATTTTGACTTTGAGTGGGGTTGGGTC
TAACAAACAAAGAAGCTTCCATGTGAGCAGCCCGGACAAGAGTAGCCTTT
TTCATCCAAGCAAAttgtgcagcaggaaggagatcTACA
>TCONS_00128823
GGCCAACGTGGATGAACATGGAGCTCCTGCCTAAGCTCAAACGCATTTCAG
TGTGGAACGTTCAGAACGTCGCACGTTTCTTTATGAGATCGAAGAACCCTC
TGTTATTGAATCTCTATTCTCCAGGCCAAAACCACCAAGCATGAATGGTAAA
TGCTGTGCTCTTGGTCATCTGACACTGCACTGATGCAATGCAGTCTATCTCA
GACACAACAGAAGGACAGAAGGGAATAAAAGTGCACTGAAATGCAAAGA
GGAATGGAGAAAAGTTTCCAGGATCTATCTCACTTCTCCTCCCTGAAACCT
TTTCATTGAAATATGTTGTGTAGCatcagtaaaataaagtaaaattctGGTGAGTTACATAT
GTATGTTTTGTACCAGTTTAATATTAGCCACAAACACTGGCTTTTACAGAAC
AATTGGTTACTGATTCAGGAGGGAAATCTCCAAATCATAGGATGGTTGATCT
CACTATGAGGCATCCCACAA
>TCONS_00131888
CCAGGAGTGAGCATTTTTCAGCTGGTGCAAACTACTCGTGCTGTAGTCCTT
AGGGCTAGATTGAGTTTCACCAAATGAGAACATGACCAAGAGCATTGGGG
ATTTTGTGGCtgaaaaaccccaaaccaccaGAAGAATATTACATCTCAGGGCAATTAGC
ACTCATAGAGCTGTCCTGAAAAATCAGCTCTTTCTACCATCTCTCATTGTCT
GCTTTATAAAGAGCTGGACTGAAATGAAGATGTTTCTCATGGAGGAAAGCA
TGAGCCTGGAGAAAAACAGgaaaagaaaatcacccTCTCATGGAGTACAGCTGAAT
TTAACAGATGTTTGCTCATTTACGACAAAGTCCTGGTTGCAAGATGGGGCA
ACTCGTCTGGTTCTTGAATGAAAGCAACTATATTACCTCGCCTcaaaggctggagaa
gaggaaacgTTAGAAATAACAACAGGTGAGACCTGACAGAAGAGACGTAATGG
CTCGCTTTGCAGAAAGTGTCAGAAACCATTTACTGCCCATGTctctgagcagagag
gaaacctCTCTGAAGCAGGAATGATTTCTctcttgtttcttatttttacagGAGGAGTTTGTATT
TCCGCCCTTGGATGTTTGCCCCAtttgtggcagtgctgtgcagaagccTTTCTTTCATGGG
GTCTGCTTCATCTGATGTTTATGTAGATTCCTGTCCCCGTGTGCAAATGAAG
TGATAGCAGGATGCCTTGCATGGGTAGACCTTGAGGGAGGGTATCTCTAAG
CACTGACGGCGGAGGTGGTGGCGGTGAAAGGTTGaaagttggacttgatgatctctgtgG
ACTTTTTcaaccgtaatgattctatgattctatgaagtggt
>TCONS_00133966
CATActcctttcttgcttttcctttgtactGCCATACCCTTCTCTGAGCCTCTGAACTGAGTTC
CCTTTCCTGGATGTCCCAGTCCCAGTGAGAGGCCCATTCACCACAGACCCA
TCTGATCTCTCCCCCAGAAGCAGAGGGCAGATCTTCCTCCACCGCTGTGTC
CTTTGGGGATGGATCTGCAGGTTAGTTTACttttgcacagagcaggaagacCAACCTAT

CTGCTTGCATCCCAGgaGGACCTTTGGCTGCTCTGCTTCATGCCCCATACAG
GACACAGCTGGATGTTGCACACTTGCTAGAGCCTTCCTGAAGAATCAAGA
AGTTTGGCAGCAATGTGCATCGGACTAACTCACAACCAGACAGTATACAAT
GCCTTAGCCAAGGCCAGAGGCTCAACAGGATTGGCAAGGGCAGTCCTTGC
TCAATGAACTAAAAGCTTCTCACTTTACCTCTGTGTCTTCACACCTTTGTTC
CTCATCGCCTGAGTAGGTCTTGGGGTTTCAGACCACAGTGCAGCGGCAAG
GTAGTGTCACTTGTCACTGGGAGCTGAGATAGCTCAGGGCACATGTCTGCT
GGGAAGCTGGCTAGAGTCTGCCACGGTATGCTCAGCTCCTCACAAGGAGC
AAGGAGTGGTCTAAGTGATCACAGCAGTGATGATGATTAAGGATTATAGCG
CAAGTTCTCTTCTTCATTGTTACAGtagtttttttaattgtggCTGTAGAGAAGTTAGAA
CGTTCTTTGTTTGGCTTAATACTATGACCACccttagattattttttaaaaatacatttaggaGCT
TCTCTCAAAAATAAGTCTATGGGATAGAGGATCAGGGCACTCAGAGGTACC
TTGGCTTCCTTACTTCAGGAGAATACCAATGGGTTGTAGTTTGAGACTTTCA
TATACATTATGGACTTTAGGTCCTTTCTCTGAAGGTACTGGATTTGGCTATTG
TCACAAATGTACCAAGTGTCTGATTCAGTCTGATTTTGTCTTAAGGTATATG
TGCTGTTGGAGAGGGAACAGGATAACACAATGGAATTGCCTTTATTCGTAT
GTgcgaatcatagaatcatagaatatttttgCTTCATTGGCCATCTTTGTCAAGTATCCACCCA
CCTATAATAAGGAGACATAGAAATGTCACAAAAATAAAGGCTCAAATGCTT
AACACACTGAAGGCTTGATTCAGATCTACTTTAATAAAATTTCCTTCCAAGT
CAGCTAGCAGCATCACAGGTCTGAATCTGATGACAATGCAGAATAaaagaattta
ataaaaaacaacaaaaatgagaCATGATAATCATTAGAcatacaaaaaagaaaccatgaTACGTAGC
GATCTataatttcagaaattattATCATTGTTTTTGTCCAATTTCCCTACATTACCCGAAG
CACTTTAAGCTAAAGCATTTGTGTAAGTGAGATTCTTCCCTTAAGGAACAG
CTCTGACCTTACATTTTCAGCAATTCAATggcagaaaatatattttcttcctacttttggtttgggaaa
aagaaaaccctcaCCTTGCTTTCTACTTTAAGATTCACTTGCTATTATGCCTTGTAGC
TATCAATTCTGATTTACTTCgctgtacagaaaaaaaattgccttcTAGGAGggtactttaaaaaaagtac
ACCATAATTCCTCCATACCATTGACATTTGTTGAACCTTCACTGTTATATTGG
ATTGGAAATTTTGATAACGTCTGTACTTCATCTTCATATCAATGAGAGCCTGA
TgatcaatttattttcttgtcaaaTACGATCCTTAATGGGTTGCATTTTGTGTGGTTGACTAT
TCTgataaaatcaatttaattaaCCACCAAAATCTGACAGAACACATTCAGGAAGTGtcat
tcatttcaaaagaataataatttcaaTCAAAGACAAGTTGCCAACCATTCTAATGAGGTATC
TCAACATTAGTGAGATTTCATGCTAATGATCAAAATGAGATATTTCAGGGTT
TATGACAAATTATGATTATAAACCAGATAGTGCATTAATACTAGGGTGCGCTG
TCAGTATTTATTCACCAAACAACCAGTGTTTGAAGCAAATTCTTATGAGTTC
CTGCATGCATAAGTTGGTAGGGCAAAGAGGGAAACAAATTACCCTCTGCTC
AGCTTGTCATTCAGAAGCTTGAAGTTTTACCTTCGCTTACCACAGGGAGCC
ATGCAATACCCTTACAGCAAGTTTTGTACTTGGTGGGGAGTAGGTTCCCCAT
GACCACATAAGCAGTGGTGTGCGCAGCTTACTGGAGTTTACATTGCTGTAC
TGTGCTATCACTCCATATTATTATAGTGGGAGGTTGAATATCAGCAGAATCTA
TTACAATACTGGATGATTAAAGTACTTCTTGACTTTCCACAAGATTTTAAATT
TGGGATGAAGGAGGAGGGGAACACAGTGCGtgagagaaacacagaaagaaaagaagagg
attATGCTTCAGCTGCCTTTGTGATAAAAGAGTGGTAATTAGTTTCAGTAAAA
TCCCATAATAGTTTTTTTGTACTTTGCAGGAAACCAAATGGCAGACAGATAA
AACAAATTACTTTACTCAGGGCAATTAGCAAGCCATTACAAGGTTCTGCCTA

TGTTTCCCCATGACTGCAAAGATCATGCCCTATTTTTCCAAGAACTTTATGT
TCCTGAAAATCTCATCCATTTTAAGCCTATCATTGTGCGTATAACAGCCACT
GCGTATCCCTGAACACAGCCCGCATTGTAGTAGAGGGCCCAAGAGATAATG
AGTTCTGTGATCATGCTGAGGAGAGAGGGAAGTTTTAACATTTGCTGCTTA
CTCATCTGAAATAAACCTCCAGTGTTACTATTGTGCGCGTGACCCTCTGACA
CTTAATACAATGAAAACATCTGAGTTTTATAGTCCTGCATATAGGTTATGCAC
CAGCATTGAAACTCTGCACTGCAGATTTAAGAGTTCGTACATTCATGGCTAA
TTCTTGATAGTGACCccattggaaagaaaagaggatttcttggcaaaagaataaaaagatttcAAACTAA
TCAGAAACTGCTatgaaaatgacttttcaacattctttttcctttgaaatttttatttcaagaaggCACCTGCCT
TTCCTGAAAAATTATAGTTCAACTTTATATAAGTCAGCTACTAAgcaaaacaataga
aaaatacCTGATCGTGTATGATacagaaaaatcacacatttgAAATAGCTTCTTTGAAGAAA
GCCCCTTAATATGCAGTCAGAGTTGTCTATCAACTAAGTTTtttgaaaattttgaaaaata
tgaagatcATCAAATTTACAAATGGGAAGTTTAATTTAgtaagtttttctcttttttttttgagggatt
GAGAaagcctttttcattttattgagACCTGGGAAATCATACTGTTATAAAATGTCTATGAC
AGTGCAGGAAGTTGTATTAAATTCTCCAGCAAGACTTTGGCCGAGATAACC
AGCTCTACACACATATACTGTCTGGCCATGAGTTCAGTGAAGAATACAGATT
CACATCCAGTCCTGCAAGATCCTGATCTCCTTTTCTAAGTTATTTACCACTA
AGTATTAACAGTGAATAGCATTTTTCATTATAACTAGGTTTGGTAACAACATG
TCTCTTTACTGAAAATTGATATTGAAGatcaaagattatttttgaTTACAGAGATTTGTT
ACAACTCATAATTTCTAGCCAGTTTCAGCCTCCTCTCTGCTGTCACTTCTAG
ACCTCCAAACCACCTCTGCAATTTTGGACCTGGCCAGCCAACTGTTGTAGA
CCAGAAATTATAGCCTGTGCAGCTGGGGTGAAACCTGacatctcagaagaaaaattcatt
tGCACTTTCCCATAACTAATTTCAGTGATAGGATCACTTCTAGGTTATAATGC
AATTTAATCATGTGTGTTAAAAAAGACCTCACACGATGACTTGCATAGTCCT
ACCAGCCAGAGTGAAAGATACTGGTCTGTTAAGACAAAGACCTCAATACTT
GAGTCACAAAATACTCAGATTTAacagcagcattcagaaaaaatagATTGTCTTTGGAAA
TTATGTTAGAAGTGATATGGTTCGTTTGTTTAGCAGTAGGGATTAG
>TCONS_00176931
CTTAATATCTCTACTGCATGAAAGTAAGTTCAGGCTCTTTTGAGAAATGGCA
GGGGGCCgagGTAAGTGGCTGTTCCTGTTTTTATAAGACGGCTGAACAATCA
GCAAACCTCAGCTCTCTAAGGTACTCTTTGTGGTATGGTTTCTTCCTAAGCA
CCTTGAAGTATTTTCCTTGTCTGCGTTTGTACGGTACCTCAGCAGGAGGTTC
GAGATAGACGAACTGGGATTTAACCTTAACCAAACAATAAAATTCCTCTTC
TTTGATTTCTGAGGTTTAGTCAAGCCTTACCAAGGTGCCCAGAAATAAGGA
GCTAACGGTGCAGTGGTGGGAGAGATTGTGTGCGGCTCTTTCAGCTGCCG
GGTACAAACACATGAAAGGCTGTTAGCACAGTGGGAGGCACGCTGTGCAA
AGCTCTCCCAAACATCAGGTCAGCTTACGCAGGTGCGCTGCTTCTTGCATA
TTCCAATTCCTTTTGCACTGTTCTCAATGGGTGCCTATTAGAGGAATGCTAC
CAAGCCGGAGGTACCCACCAGAGCTCAAGTGCTCCGAGTGTTTTTGTAGA
CATTTCTAAATCTGGTAGAGCTGGCTGCAAACTAGCAAGCATCCAACTATatc
ttctgctgctccttctcttgGGAAGGTGTTGCACAATAGTGCCAATGGGGATGACAGGG
AAAGATAGGTGTAAGCCCCCAGAAACATACAGCTGTCTGCAGTCGCTGTGA
AGTTGTTaaatcatgtatttttaatCGATGTTTTTGGCATTGGGGATccagagcat
>TCONS_00181492

CTCACCCATGTCCCTGGGTGCGGAGTACCAGTGCTGTGCGAGCTGGGAATG
GGGCAGAGCAGGTGATTCAAACAAGTCACTGCCTACTCTTCATTTCCACTG
TTCTAGAGACTTCCTCTGTGTAATACAGCATGGCTCTTGTGGTACCTCCTCT
TCATTTTCTACTCAGCACTACATAGAAATGCTGAGAAGGTTCACTGCGTAAC
TGTGTGTGTGATGTGGAGAAAAGAtcaatttaataatttaaaaaggcaaagcaaCAAATACA
GGAACTGCGGCTCATGGAGGAAAGGGAGCACTGAAGACACCCACCTTTTT
GTTTTAGCAATACCTCATATCAAACTATATTTTTGTAGCTATGCATGCTGTGC
TTATTTTCATGAAGAAGTCCACTGCAGTAtgctagaaagaaaacagatttgaaatGGGACC
GTGTGCTTGgcttcaattcttttttttttttaatgtttgcatAGGTTggatttttcactttttcttttcatcctttctgcactct
ctttctctccttgttctaacctttattttttgtattgtATAGACATAGCCACACTTATTTTTACACAGTA
GCTGTGGATCTAATGGTGCTACTGGAAGGTGAGAATTGAAGCAATGAGCAG
TGCGTCACTGGTAGGGTTGACCTCATTTCttattaattcttttcttaCAGTATGAGTAAA
AAAACACTTAGTCTGAAGATGGCACACTTGTGTGCTCTTGGGATGTATTTG
CACGTATGAAGGCAGCTGAGGAAATCTTGAGTTTTAGGTGCTCATCTCCAC
ATTCCCACCTCAGCTCTCCAGCTCTGCAATTTTACAAGTGGTCTGCGGATGC
GTTTTGCATGCCAAGACTGGGAAATAATTTGAGATATAAATACCTTTCCAGA
AAATGATGCATCTGAGAACTGTGGCAGATGCACAGGGACCAGAAGAGAGA
GTGGGAGTCTCTTAAAAATGACTCCTTCtccaagaaaaaatatttgtggaaCTGTCTTGA
CACACagaaagacacacacacacatctcccTGATATGATACAGTAGGATCTGATCCTTCAT
GGAGAACCCAGTTCATCTTCTACAATGCTCCACAAAACAGTTCTGTAGGTG
GGTCCCCATGGGGCAGGTAGGACAGCCccttgctctgtgctcctgggcTGTGGACAGTC
TGGCactggacagcagcagcagcaggagggaagggaaggaataGGTTGGGATGCCTAGTGGGG
GCTGCCTGGGAGGGGTGCTGCCTTTCctacctgctgtgctgtgaggctAGGGGCCTGAT
GGCATCACAGACTCTTTTTAAAGAACTCTTTTCACATACTCTTTTAAAAGAG
AGGGTAAGGTGATTAGCAGTAGAATAATTAATGTGTTTAtcatgggaaaataaaatctta
ggCAAAGCCTATCgagagtcattttttttttcataacaatGTAAAACGTTTGGTTTATAGGGAC
TGTacctgtgacttttttttcccatatggACTCATGCTTTAGAGTATATCCTTGAGACCTGATC
ATTCACTCTTTCCTTTAAATTAGGACTGTATGTTCTAAAACTATTAATATCAA
AAAAGATCAAGTTAATATATATCTCGTCAGACACTGTTGAAGTGACATATGT
GCTGTTTTGGAAGACTTGTATACTgagtgacagaaataaaaaagctgaaatgaccagatgacctttttct
attgttttttttttttctttgatgggtaaattatttttatacttaATAAGTTTACCTACTTTCtaaaatttctaaaataatttc
ttcaagTACAAAATtaagcatttacttttttttagGGTGACCCTGCTGATTGTGTTAATATAAG
AAATTTCTATCTCGGTTGTTGACAAagtggatttttaaaatacttctttctaTGATATCTAGAT
GCTGAATTTTGATTGGAAAAATAACCAGGACTCCTTGGCTCTTGTAAAATG
ACAATGATGGTTTTGAATACTGTGAGATAGCATGATTTCAAGAGAATGATAA
AGAATCATAAATGTTCGTCACTTGAGGAAACAGTTTAGAAAAGAGTTCCTT
TTGTATGCTTGGTATAAAAAGATCATTTGCTTAAACTATTTCAACAAACTATT
GTTTGCTGGAACAAATTTTACCAATCAGTGGGGCTTCAATCCCCAGATTATG
AAATACTTATTTGACTTTTTTAGGTTATAAGGATGAATAAGGAAGGCTTGCC
CAGGGagtttgtggagtctccttctatggagatattcaagacctgtctggatgcccACCTGTACAACCAAT
TATAGTCTACCTGCTTAAGCAgtggagttggactcaatgatctcttgaggttccttccaacccttgtgattct
gtaacaAACTAAAACCAGCCTGACAGTAGTCTGACTTTTGACATTTTAATGGTT
AGCTGCTTTCCTTACTCCCTGGCAATTCTGTATGAATAAAGCTTTGAAACCA
GAGATCTTGGCCAGGTGTTTCAGAACTCCAGAACGTTCCTCTCCTATTAGA

GAGAGGTTGCCCTGCAGAAACAGAGCCTAAAACTCTTCTGGAGATGTAGC
TAGTCAAGAGATGGAGGAAGACCACACACAGGATACATTGGGGAGTCCTC
ATCCTGCTGAAGTAACCAGTTGGAAAACCTGGCAGATGTCACGTGGGATAA
AAAACTTACAGAAAGAATTGGCAGCAGAGTTCAGCTGCAAAGCTGGCCAC
AATACAAATTGTTATTAACCTTGATTAAATATAAGTAATGGCTGACTTCTCAG
AAAGATTGAAATCTAGTAGCTTTGAATGGGTAACAGCAAGCTGCCTTGCAA
TTACCAAGAAGCAAACATTATATACAAGATTTAATGTTGTGGTCATCTGATT
AGCATTGCAAATATAAGTAATTTATTTGTAGCGCCTAATTAAGTTTAAATGTT
CTTCAGGTTTTTAGGGAATATCCTTTCTGTTCTCTATCTAGCAATTTTCAGGC
CTGATGAATATTTGGAAGATTAAATTACGATGTCAGATAGCCACAAAGCAG
GACTCTTAATATGGAGTTTCATTAGTAGTCTCTCCAGAGCTTAAGCTTCTCA
ACAGTTTGTtatttgcaaaattaaaacCACCTCTGAGAGTAGTTTCCAGTATCTGACAG
CAGTGAGTGACTGCACATACTGTGCAAACatacaaatgtatttaatagAAGTAGCTTAG
GAAAAGAGATCTCCTGTTATTCCCAAGCAGACACTGTTATCTATGGCTTTGG
CTTATTTCATTGGGTGTTATTCTCATCTTGTCAGATATGAGTAAGTCTACTAG
GAATAGTTGATTCCAGACAGGTTCTCAAAAGAAGTGTTTTGGTAGATTTAC
TGTTCTGTGTATTGTGTACTTCACAGATTTCAAAAGGAAGCTGCATCTTGTT
AAATGCAATGACTGTACCAGGTGTCTTTGAAAGTGGGATAGCTCTGGCAAT
GGGAGCAACACAGAAATGATAGAAAAGGCAACTATCaataggaaaatgttttccagaaa
aGTCAAATCATTTTTGAAGCTCTTGAAGTAATGCATGCTATGTCAGTACAAA
AGTTCATGAAGCTCTTTCTgctaaaaatgaattttgaataaTTTGATAAAACAATCATGGG
GAAGATGCCCCCCCGTAAAAACTCCACAAAGCTGTCAATCTGAACAGATAT
TCAGAGTCAAGAAACGACTTAATGCACTGTGATGTCACTGCTTGTTTcatataa
atgttttataatttcttcctttccttcctttccttccttcacaaaagaagaaagctaaagaaatattttcattccatTCTCAACC
CTATATTTTCTTGTATTCGTGGGAGTGGAATTTCATACTAAGATCCAATAGAG
ACCAATGCTATTTTATCTGGCTATAAAATTCTTGAGTTTGTCAGAATTCTTAG
CCTATGCTTTTACTTCTGATAATTTAAATAGGAATATTTAGAAATGGGATTCA
GCTATTCGACTTTTTAATCATTCATGAACTAAGCAGTAGATATCATCTTGACC
ATCATagtttctcattatttttattttcagagatgtAAGCAATGAAGTTAAAACTACAGTTATGG
GCATTTTCTAGTATTTGTAATAAGATACAGAAATTGATATAGCATTACACAGA
CTTAACGgtttcagtacttgaaggatGTAATTCATAGTGTTCACTGGAAGCTATCAAAA
GTTTTGTCCTGGAAGCAATACAAGATTTTTCCTTAAAGCTGCAAGAGTAGA
GTGATTccttgacaatttttttttctgacttcctATTTTCTAGAGTCAATAAAGGAGAAAATCAT
GGGCATTTTTATTGGCTTGCTTATATTACAGATGATGTCACAAACACATCAA
GATATATATGGTTCAGAAATATTGTACCATGTGCAGCTAATGCATAATATAAA
TTTAGTCTTCTTAGTTGAAGGAACTCTTGGAATGTCTCTAAATTTAAATGTG
TTGACAGCAAACTAACtcatttgaaaattaataataattttatattcaaatattttcatatagtAGTAATGAT
ACTAAGTAGTCTTACACAAAAATCTATTTGTATAACAGCTGCtattatgtgattttttttta
atgtaaattgtGTGGAAATTCAAAAAGCTCTAGTTCTCTTTTTGAGAGATGCATTA
AACAGTTTCTACTAAACTTTTAGCAATTACACATAATTTCATTATAATCATAT
GAGCTCTTTATACTATTTTCACCTTTTTGAACTAGgatatttctgtaatttcttacAACAGA
ATTTGTAGGTGAAGAAGCAGGGGGAAGAATTGATCAGAATTAAATTTCCTT
GGCTGGATGAATCAGATATCTCTCCTTGAGAGCTTAGCAAGAAGTCTCTTGT
ATTCCACAGTGAGGAAATAGCATGGTATGACTCCTTGGCTTTTATGGGAGCG

AGTCATTCAGCAGAGGTTACTGAAGGATAAGCAGATGGATTTCCAGTGTTT
TCCATTTCCACTTCTTTGCTTAGCTTGGACCAATATTATGGCAATTGATTTGT
ACCACCTCTTTAAAAGGTGAAATGAATGAAGAATGTGAAACACAGAATTGT
AgtggctggaagggacctctatATGTAACACATAAATATATGTTCTAGTTTTCTGGATAG
ATATATGAAGATAAGAAGGAGGCTTTGTTCTTGTCTTGCTGTATATGCCTCA
ACATTTAATCTCTAAAACTGATTTGGTAAtcctaatttcattttttagctGATAAAGGCAAT
TTCAGAAATTACTTCATCTCACCTTTTTAGACAGCTTGTTTGTGCTGGGGTG
GATTGCTCTCTGCATGCACCAGCTTTCTATTTAATGCAGAGTGCTTAGGTTG
CTTAGCACAGACCTGAGTGTCTAGAATTAAATTTCTATGTTCAAGCAGGTG
AATTTCTCCTGGACTGTAGAAGGCAGGACACTGTGACAGAGACTTGGTCA
AGAAGAGTGTTTTATCCTGATATCCAGGTTCTAATACATGTGCAGTTACTGC
ATGAAGAGCAGTCAATATTTACCTATCTCATTTTCAAAGTCAGATTGTAACC
TCACCAAAAAAGACAGCACTGGAAGACAAAGAAAGGTCACTAGCAAAGG
AAGCAGTTTCTATCCAATCCATCAGAAAAAATTCAGAGAAGAATGTTCTGT
AATGATCCACTAGGCCATTTCATGTGATTTGATTGTGTGAAGCAACTATTTC
TCAAGAGATCTTCTTTGAGTAATTAAGGCTGAGTAGCTCTAACTTACTGAAC
TATTTTAGTCAGTGTGCAAGCCCCTGTGAATGGCTCTCACATGGCAACGCTA
TGGAAGATCAGCTGAACTTTAAAAGGCCACATTCTTCCATTGTCCTAAAGA
CTGCTTTATAAGTTCACTGCCTGGCTCCATGGGTTATTTTCTCTGATGAGTAA
TTAAATTTTAACCTGGAGTCTAAGACCTTGAGAAGCAGAGTTTCATGAGCT
ATTAATACAGTTAATATCTCATGagttttcttcatgctgtgGTTAAAAAGTTCACTGTAC
TCACTCTTTACTTTTATATCACTTTTATTAGTTTCttcacatatttatttaaatagccCTCTCT
GAAAAATAGTGTCACAACTTTATTTTATGGTATGATCTCTCCCTAGAGGAAG
ATGAACTTAATTGTATTATGTCACTGTTATTGAGTGGTTCATCTGTACTTAGT
TTGTGCACTGattccaaaatgttttaaaggagGAAGCTGAGTGTATCTTCTAGTCCTACA
CACTGTAAAACAAGCTTTTCTGAGAAGGCATAGACTTAAGGATTAAACATG
TGATATATTTTGCATGTGGAGCTGAAATTTCCATAATCGCTTGACATttggttctgttt
cttttgaatttCTTAAGTTTATGCAacttatttccttctcttggtTAAATGGCTTTGGAAATAGATG
TACTatgttatatatttataatacaaGATTATTGTTCAACCATATACAGTTGTAGGGATATCT
TTATTCCTTATCATATTTTTCAGATATAATGCTCATTCTGCCCACTACATAAAC
TGTCTTTCCTGTGTGCTTTTGGGACAGAATTTACATCTAGTTGCATTTGTAGT
CATTTTCTATGCTTTTGTGACCTTATATCATTACCAGGCAGATGAAATAGTCT
AGTTTTGTTTCTCAGAGTGTACTACTCAGAATTTACTTCAGATGTTTTGCTG
TCATGCTTTCAGTGCATAATATGAATTCTCACCTGGTCATACCCAGATACAG
AGTTGTGTGTAATGTGAGGAAAGAGATGTTGATTTCGGATGGCATGTGCTC
AGTTTCCCACATTTTGTAATGAGTTTTTGTAAAAGGCAGGAAGTCATATCTG
AGCTGCAGTACCTTGCTTACAGAACAAAGCTGTCATTGGAATGATGAATCA
ATTAGCCAATGTACTTGGAGGTGGTGGCTATCTGGGGACCAGGGACTAGCA
ATCACAGCATAATTATGTTAATTACAAGTAAAACCCGAAAATCTCAATGATA
ACTTGTTTGGTGCCTCAAAAGAGTTAAATAGTCAAATCTGAGAACACCTGT
GAGAGCaagtaattatttctgaaagtatAGAAAGGAATAAGTGGCACCTAGGGGCAAAG
TCCGGGCTAGTAGCCTTTCTTGAACTACTGATGTAGGGATGGCAGCAGttagaa
ataataaatcaaaagataaaatgatgggggaaaaaaaaaagggcataTGAGAGGTTATGAACTACTA
GATTTTTGTTAGGAAGGTCAAAGTTGTCAAAGGAAAAATCTATGACTGAGC

TAACTGTGATagaaaactttcttcttcttcttatcttaTAAGTATGAAAGAAATGTTGGAAACAG
TGTCAGTCCAATAAAGAATGGAAATGATTACATCGTTACTATGGTTCATGGA
AAGCAAAACTACtcaagaaatatatattttaatgtagaAGTAGGGATATGTCTTCCTATTAcat
gaaaatcatagaatcgctcaggttggaaaagaccttaaagatcaacaattccaaccacaacctaaccacactaccctaactc
taacaaccctccactacatcatgtccctgagcaccacatccaaatagtttttaaacacatgcagggatggtgactcaaccatc
cccctggggagcctattccagtgcttaacaaccctttctgtaaagaagtgtttcctgatatccaacctaaacttactctggcaca
acttgaggccatttcctctcgtcctgtcacctgtcaccagtgagaagagaccagccccactctgctgtaagcacctttcagat
attggaagagagcaataatgtctcccctcagcctccttttcctcagactaaacagccccagttccttcagtctctccttgtaggg
catattttccaagcccttcgcaagccttgttgcccttctttggacctgctccagcacctcagtgtcctttctgtactgaggaaccc
aaaactgaacacagtactagaggtgaggcctcaccaatgccaagtagaggggcaggatgacttccctagtcctgctcacc
acaccattcctgatccaagccaggatgccattggccttcttgtccacctgggcacactgctggctcatattcagccgactgtc
catcacTACACCAAGgttcctttccatcaggcagctttccagccactcttccccacGCCAATAGGGTTGC
CTGGggctgttgtgaccaaaatCATAGTTTCTTAATAACTTAGTCGTTCTGATCTTGGT
TCATAAACAATTAATGTGATAAACAAAGTTTACCATGGGTAAGCAGTTTTAA
ATCAGAATAACTTGTAGGTAGCCGATTGGGAAGGTATCTACCTCCTTAATGT
TAATCTTTAATAAATATCAGGAAGATATTGCAATTTGAGAGAACTATCACAG
GCTGATATTGTTCCACCATGAGAAAAGGATGTAATTATCATAGTTTTAAAGG
ATCAGAAGTTCATTAATGCTGGTCAACATGACTTCATGGAAAATGAGTCTTA
TCAAACAAACCTTATTTCATTTTGGAAGGATTATAAATtagactgaaagaaaactgcata
gGTAAGATTCAGATTCATAAAGCTGTTACTTTAGTTCTACGTCATTCTAGCTT
TGAGGTTTTTAGGACCTGTCATTTTTTATCATAAaatttcaaaagtaattttaGAAGCCA
CtgttgatttaaaacaaacaaaagggaagATTTTAAACCGGAGACTTACATGTTCTAGTGCA
GGTTGGCTTGCTGAGAAAGTCTATCTGGCGGAACAAATGTACATGGGAtccaa
gggaaggaaaagatggaCTGGAAGACTGGTGGCTGAAACCAAAGTCATGTTAGAAA
CTGggcatcatttttttttaatgatgaaagTGATTAATTGTTGGAATAAGTTATTTGGAGcgttctct
gttttcttttatatttatatcTTATCTTTCTGGAAAACAAGTCTAACTTaaaaccaaattattttttttgctgt
tttatctAAATCTAAATTATTGAATCACCAGGCACCACTTGGGGTGCAGACAaggg
attttttgtctttatccCTGCTTGGCAAGGTATCTGCATGCTGTCCTTATAGATAGTATT
GTGAGTGACCTCTCCctaggtattttttttttcctagggcATTTAACAAAGCACATAAAGAA
ATTCATGTTTTGCTGCAGTTCTCAAGTTATTTAAAGTAAGGAGTTGAAGTTG
GTACCATAGGCATTTCCCTGTTAGATATTCTACTCATAGCTTGCCTCCCTTTC
TGTTGTTTCCCCATTCTCCTTGGATATCACTAACCTTGGTGTTTATCCAGTAC
TCTGGGAATGCAGAATTCTCTTTGGACATTTCACACAAAAACTGTACAAGA
AATACAATTCGTTTTACTAGACTTTTTGTCTCAAATTGATATTTAAGTTAAGA
AGTTATGAGGCAATTTTTAAAGTCATTCTAatgcattttcatattttactCCAAGCATCAA
CAAAATTCCATATTTTGGGGTGATGTATCTCCCAGCACTCATGATTGAAAAG
CTGGTCTAGAAGTTCTGAGTgtggtattttgttttgctttgccttAAAGAAGCATACTTTATT
AAGCACATTTGTTGCCTTTTGTCTTGTTTGATACATTTTACAGTCTACAGAGT
ATTTGAAgtaatgttttcctctttgcaaGTTGATCACGTTTTTTCTGACCTTACCCTTAGA
AAATGTCATTATTTAAATAAGGGATCTTTTAAATTTCATAGCATAGCAAACTG
ATAAAAGACATGTCAGACAACCATTTTGTTGCAGCCATTTCTAGGGAAAAT
CAGCAAAACTAGGCTCTTTTCAAGTTTGTCAGGTGGGTAAGTGAACCTGTC
ACTTTAGACTACAACACCTATTGCCAAGAAGTTCTCAATTAGAATTAATTTT
GACCGTCTTATCACATCTAATTGAAAACACAACATTAAACTCATAGTAAGAA

AGAAGAGTGCACAAAGccactgtgttttatttatttacttatttattttcatgtttatttatttaaggtTTTTGAT
TCATCAAAGCATCAGATGGGAGGCTATGTCTGTAttgtttcttttggttttttttggcgTGGA
TTCTGTTATATAATTTTTTCTCCTACTTCTAACCAACTGCTAGAATGAAACAA
ACACCTTAGTAGgcaacttctattttttctcccctaaatGACTTCATTTTGGCAGGGAGGAA
GCATCTGCACAGTACTGTAATTTCTTCCCACTAATGTTCTTATGAGAAACAC
AGAGGACAAAACCAGTCCTTGCTATGTACAGACTTATGGGGTGGTATTTGC
TTCACTAAAAGCCTCCTTGTTCCAATTTGTTGAAACTCTGTAATGATAATATT
TGGCAGGGATGTAGCCCGTGTCTAGGTGACCTTGCCACTTGCATGGAAAGT
GATTAGTAACAACTCATACTAACTGCATTTTCTTATGTATGTAGACTCATGTT
GAAAAAGCTTGTGTAAATATTGCTTGACTCTCATTAATAAATGTTTAGACAG
CCACAGTCACATTAAAATCCATTCATTTTATTAGAAACCTTCATTCCTTTAAT
TTTGAATTTCCAAATTGgtattttagattttaatatTAACTTCAAATTTTAGAATTAGCCTT
ATTTCGTCTTACAAGTTAAAAACATAGatcaatttcttcttctttctcatttccaaaGAAGGCTT
CCTTCTTTCAagtgatattttctttcagcctgtaagaagttccatacaactaattttttctgtctctgtacTTAGAAC
AAAAACTTTTGCAAATTCAAACAATATTAAGCTGATTTAAGATAAAAGTTTA
TCAGATAGTTAGACACTTCcaatggaaaatacaaaataagctGTTTTCCTATAGCTAAGG
AGTCATTATTCTGAAATTTAACAAAGGTTGAGACTctcaaaatctgaaaacaaaaagtttttt
AAGACAACATAGTTTGATGTCTCCCCTTTTCCCTCGAGCAAGAGGTGGgttca
attaagaaaaaaaacatgtatttcaaCATTTTATTCTCACCGTAACATTAGATAGCATACAGG
TCATGTGCCTTTAACCTCAGGGACTCTCTTCAATAGATTCTATCTGTAGATCT
TTGACAACTGCTTCCTTATAGGAAAAAGCTTGAGgttgctttgtttgcttgtttttgccAAA
AATCTTGCCTTTCagtacataaaaacaaaacaaaacaaaaaaaaaaaaacacaaaaaacccaacatatttaaga
aagaaacGTGTTATATAGTCATGGAGTAATTGAGGTTCAAAGGGACCTCTGGTC
CAACTCTTGCTCAGTGTAGGCTCAATCAAATTGTTCAGGATTGAATGTTGAA
TATTTCCAAGGATGGCCACAATctctctgagcaacttgattCAGCATTTGACATTCTTCT
ATACGTTAGCAGACCAATGCAAATATAtgatggaaaagattttttttcttttacacagTGTGACA
TGAGAGCCTTCATAAGTGACAAGAAATCAAGGGAGTTTCCTGTCTTATTTT
TCCACTATCATAGCAATGTGTTCCTATTAGCTTATGGATAAAACATATACTAG
AGTACCAGAAGTCCAAATAGTGCCTCGCTTTACCTCCCACCTGCTAACTAA
GTTATCTATACACAGGTAGGCCAGACTATTATAGTTTTGGCTGTCATTTTGTA
AGGCATGATTTTATACGTTGTCTGTGATCATCTAGGAAGGATTTTCAGCTGT
CTTATTTTTAGTTTGGAAAATGTCTGATCAAAGGAGACGGGGTTAAGGTTG
ATGCAGGAGAAACACTTTAAGTTAATTTCGTGATTACTCAGGAGAGCATCgc
ttaagaaaaatgttaaaggaaaaatgatgtGTACATGGCAAATCAATGATCTTTATAATAATTT
GTGCAATATCCACAgagctaaaatattttccatcatgCCCAAATGGAACAAGCAAATACA
GGTaaggaaaaacagattaaatatgTATTATAAATACCTCCATACATCAACAACACCAAT
GGATGTTGACAAAGAAGAGTTAGGGTTAACAGCATGGCTGAAAGTTTATAT
AGACTACAAAATATTCAAACACAGATTggggaaaatggagaaataaaaacaaaacatcaacaG
CAAACCAGTAATTAGGCTGGTGCCTCAACCATGGCCACTGAATTGCATACA
TGCTGAGGACCAGCCACTGGGATTCTGGAACATTCATTATCTTCATACTCTG
AGGAATGACCCAAACTTTAATTACTTTTGGATTaggaagtcttttttttttcctctgaatatGTC
CTTTCATAATTGCCtttttcaggatttcttttGTGTTCCTTTGAGGAGATCCAGGGAAGA
TGATGTAGGATTCATTACTGGACCAACCAGGCCACCAGTTTACTTCTGTGCT
ATGCTGAAGCAGAAGGTCTGTGTGTTCAAAATGGAACTTGAGCATCTTTAG

TAGTGTAGTGTGATAATTACAGTGTCACCATGATGCTCCTGAAATAGGATTT
AACATTCTGCAAACTCTCATGGCAGCTTTCATAGGACCAGATGCTTCAGCT
CAATCTGTGTTGACTCAGCTCAAGTGAAAACTGCTTGAAGTTTTGCAGTT
GAGGAAGAAGTTGCTgtatccaggaaaaaaaaaaaaaaaagttcatttttaacCTTCTTTTACTGA
TTTAAGaagctttacttttattttatgaaaaatctttttgagAATACTTTTGGTCTACTGTCGAAAAC
CAAAATCTCAAACTGACTTCATAGTCACTAAAATCTACATAAAATAAAGTTG
TTTATATACttatagatttttaaaaacagatttagaagattttctttttagtggttaaaaaaatatttatcaaacgTGTTT
TGACCAGGTTCCAGCTGCAGAATCATAAAAAACAAGTTAAGGACCTCTCTT
AGTTTCTAGCTCCATCAGCAAGCTATGCACACGACTCCCATTTCCAGGGGG
TTCCTTCAGCTGGAGTTAATTCTGGCAAAATGCTCACCCTGGCAATCTAATC
CAGACATAACTCTTAACAATTCAGGCTTTGCATGATTTCGTGGTGCTTTATC
TGAGTGGCAAACATAAGGAGGTACCTGTATTATTGTAAATTCAACTATGTTA
ATTTGCAATTATTGATACTTTCAAAATTTTATAGGATCTTTTTGAGGAAAGAT
TTGTTTGAAAGAAGGTATCATGACTGTCTTATCAAgtgtaattaaaatgtaaattgttGATT
CCttagaataaaaaatgatttttttttttaatggtgattCACTCTGAGGTAGATATGACCAATATTCTC
AAAAgagttctcttttcttcttctttgctACTGTTTTGAGTTGCTAAAAATTGGAATAATTTT
CCTATGGTAAGTCATTCCAGCTTCCTGACAGTTCGGATAATTTTTGTGGTATA
CTGTCATCCAAATGACTTGATGCATTTGGTATGAATAGAGATGGGAACATTT
TGTAAGTTATAACCACAAGATAATTTATTCATACAGAGAAGGCATTGTATTTG
ATAACTTAtaaatttcattctgttttcatgataaaagcaaaaaaagggTGTGTGGATTGCAGCTGCTG
GCTTAATAATGCTGCTGATGACTAAGAACGGAATGGATCTTTCTGTGAGTTA
TATAATCAGTTTAAAAGCTCTCCACATTCCACCTCTgacaatttattaaaaaaaaaattagaac
aagATGCTGTGAAAG
>TCONS_00185509
gcagTATGTGggaaaaatacttccttttgtTGGCTTTAAGCACAGAGTCTAAGCTGTCCCT
TAGCTCCCGTGCAACACGAAGCAGTGGAAACAGCACGTAGTAATTCCCTGT
CACCCTCTTTCTACCTTATGCAGTTTTCATAGATCTCCATCAAAGCAGCTctca
agcttttctttcctgatgaACTTTTCTTGGGATGCATTTCATATGTTCATCTGTCTCTTATC
CCCTTCCCATGTGTCTCTCCCACAAACACCAGCCTGAGAACTGTTTACAAA
ATAGTCCACAGATCTCCTGGCAGACTCCGAAGCAAAACACAGTCCCCAGC
CAAAGTCTGTGTTTTGGAAACTGTTTCTTTGttttcaaCTTCAGCAGAAACTCTG
GCTGCCAGACATtatggATTTAATTCATTTGGCAACACTACTTAGGCATGGATG
GCTGATGGACCATTACAAGTCATTGGCTACAGAGAAGGTTAATCAGAGAAA
TGTTCTCATTAACCTGAATCTGATTCTCCTCTTCATTACTGAAAACTAGATCA
TTTTCACTATGCAGGTAAGGAATGAGTTACCTTATTATAACATAcgttcatatatatatt
taatgtgtgtgtttgtagcTTCTATGAGagctttgtaaaataaataatatattgtgcagaaacattttatattgccttgaga
acagaaaaacacactaCATATATAGGAACAGAATACTGCTCGTTTCCAGTGCATAAAT
GCACAAGAACAACTCTCAGAAGTTTATTTTGTTAGCTGGTGACCTTGATAG
CTCTTCTTTTctacagtttttctgttttctgtgttcttgtgttggcttcctatttttcttcttcttttttttttaatttagtatcCTG
TCCATTTAATAATTCAATATTTTATgaagatatttcattttccttgaaCTGATTCCTTGCTTAA
AGTACATGACGTTCCATTCCTTTTGGTGGTGATTCTCATTTTGTCTACCCAGT
TTTTCACAAAACCTTAGTAGACTCACGAATGGTACACTACTGTCTCTTAACA
AAGTGcttatgtttttaaaactgatcTTCTCATTGCGAGCTTTATTTCTCAGACTATCAGGT
AAAAacctacttatttatttattttggcaaaCACATGATGCCAATATTTCATGGTGAGTATTGT

GTAGATAGCAGATCAACTTGCTTCCTGAGCAATTCAAACATGACAAAGTTA
TTCACCTATTTTTTCAACTATTCATGAAAATGGACTTCATGCATGCTTCCATT
TATGTGCATAGGAGAGATCAAGACTAAAGGAGCCCAGGGTGTCCTCTATTT
GCAGCCACTGGACTTTACTGCAAGATTCATGCTGTAGAGAGAGCTGGAAG
CAGTACTAACAATTTATCTGGAGAAACCAAGAGCACCACCCAGTTACAGGC
AGTTGGCCACATGGAGATCTAGGTTAAAATTACCTTGGCTGGAGAAGAGAG
TGGAAGCAAGGCTACCACAGCAAAAGATGTGTCCTTGCTGCTCctagaaaggaa
ggaaagcatgAAGGCATCTGCAGCCTCTtggggctgggaagggacttTCATCCCCAGCCCT
TTCGTGTCTGTGAGGTAAGAGGGCTCCTCTGGTGACTTTCCTAGATCCCATC
CAGAGGCATGTGGGTTCATTTGTTCAACAGTAAGCCCAGGTACACAACCCA
GGGCTGGGCATTTCAGCAACTGCCAAGATCGTGGCTTCATCCCATGTGACA
GTCAGCCTGACTCATTCTCCAGAGACTTCCAATGACTTCAGAGGAGCATAG
CAAGGTTATGCTCCTGGCTAAGGTTCCCAAAGTCAGTGGGATGTAGCTGGA
TAAAAGTGCTAAAAGTTCAAAACTGCAACATTCGCTATTGCAACATCTAAA
GCTTTTTATGGACTGATCTGCTGGGGAAATTCTGGTTAGGTAAACGCAGTG
GTGTTTTACAATTGACTCTAATGGCCCTAGAAGTCATTTAAAGAATTCTGTA
CAAAAGCTAATGCTGCTACTCCAGGTATTAACTGGCTTTGGTCTGATAATAT
TGAGAGATCAAAGCCACCCCTAAGTCTTGCTGTAATGAAAGATAGGTTTCA
ACTGTTCCTTAAAAGCAGTGaatcttttcagttctttttcttctaaaagctCTTTCTATTAGACGT
ATTTTGATAGAGAGATGTATTTCTATTAGATGTATTTCAAAAATAGGAAAtgtcc
ctattttttttttccactaaatgCTCCTATCTGTATGTTAAAAAAACCTAGCAGGCTTGCaggt
actgaaaaaaaaaaagccctcaagggaacagcattttgaaatggaACAGGATTTAAAGTGAGAATTC
ATGAATATCTCTTTTGCTGGACGTGACAGCAAATCTTGCAGAGATCTGTGGT
GCAGAGATCTGTGGTGCACATATCAGCACAGTCCAGGTGGGAGACAGCCG
TGACTGCACAGCCCTCTGCACTCTATAATGGTGGAGAAGGAGGGCAGCAC
TCAGAGCAGTAACTgtgagcccagcccagctctgaggTGAGGAAGATGAGGGTACTCC
AGTACAGGTGGTGCAGGTAAAGGTGCAGAGGCCTGTGACTGGAGAGCAGT
CTGTCCACAACAGCTCCTAAACTATGGTCCTGCTCCATGAAACTGGGCCAC
TCTCAGCTTTTTGGCTTGGTTCTTAATGAAGAAATTCAGGATTGTGGGAACC
TCTTTGAGCTCAGCACTAGCACACGTATCACTAGCcatctgttttgccttttgaTGGTTA
GTTCTTTATTAATGTTGAGTCTGCAGGTGTCTTCTGAATACAAATCACTGTA
CTGCACTGTGTATCTCTAAGACAAAAAGCAATTACAGGCACTGCCAACTTG
AAAACATCTATCCTAAAGAAGGTCTTGCAGGTCCAGGGCATGAAGTGTAGC
TGTGAGACAGACCAATGAAGAGTTATTCCAGTGCTCCCCTGTTATGTAGCTT
AGCTCAGTTCACTTCGTCTGACCATGCCTTCATCTCACTCTCTCTACACAGT
ATCCTTAAGCCAATGGATGACAAAAGTCCCAGGAGCTGAGAACTACCAGC
TGCCCTAGGTCCAtactccagcacagcacagactaTGGGGTGGTAGGAAGTATGGCACC
ACACAAGATTCTGGTCTAAATTTGTCTCCCTGATTGAAAGTCACTAAGGCC
CAAGAATGTACAACGAGATAGTTGATTGTTCTACTTAACAGAACTTTGGTT
GTATTGTCATCGGGTTCCTTTCTGTCACTGAGCAGCTGGACAAAGCAGTGC
CCTGCCACACAAACATACATCAGCTGAAAGCCTGGAAATACTTCTATGTAG
AGATGGAGGGAACACAGCTGCTTGAtacagaaagcagatgaaaaggTAAATGTGTGAA
TAGAGTTTATGTGGAGCGGAGGAGGCACAAAATAATGAATGTCATAGAAAA
ACAAGGTCTCCTATTTGTTCCTCTGCATAATGCCAAAACAAGACAATTTTaaat

gggagagaaaagcagcacatccTGAGTGGTGCATTATTAATTTACAGTATTAACCGATG
CATCAAAATGAAGGATTTAATAAAAAGACAAGTAAAGAAATGTCTTTGTATg
gagaacaagaaaaacctAAACCCTGCTGACAGCACCAAGTGACAAAAAAACATGAC
CCTAACCAACTGCTAGTGGGTTTCTTGTCTCTTCACTTTAGCAATGATCTGA
ACAAGAGTTTAAGATGGTCAGATCAACTCTCTGGTCAGGTGTTACAATTCT
GTACAGATGGCTTTAGGCATGAAGTTCCTAAAATGCTCCACGTTTACAGCA
CAATGACACTGTTTGGTAGAGCAAAACACTATtcttcaacatagtcatcaccattagatgtgcat
ttttgccagcgatgaacaagtGCCTGCATGACTCCTCACTTGCAAAAAATCTGCACCAT
CAGAAGTGCcccactgtcactgctgaaacgcaccacccactgcctcactgcgcTCACAGCCACTGTT
CAGTCTCCATCATGTTCATtaaatgttgatgaatgtcaatgggtgcgatttttccatatggaggaattcagtg
acacacctttgcttcatacacgcttccatgtcagacaccgttgtgtcagactgcccctctgctgccatcagtcacatggcaaca
acatgtaatggaatattggtgggaaggttcagcctctctTGCTgtgccaccaacatctgcctctgatgtcatgggccaaca
taataaaataggaggaattacttcTGTAGCAGCCATTGTATGTTTCACAAGACTTCAGCTG
CATGCACTACTGTTGTCAATaacttgtgggtttttttttttgtagcctGAGCAACAGCAAGGA
AACTGAATTTGGCCAGAAACCAAACTGAAGAGGAGTACATTCATTCCTGG
AGTGACTCTACTGACCTCCCTAGAGCTGCTCCAGAGTTCAGGCCAGCCTTG
ACCTGCTTTTCTGCAAATTGTGTGGCTGGGGAAATGCTGTTCCATCCATCTT
TGCTCAAAGTAATAACAAGAGAATTCAGAAGGGTTCAGGGAAGGGTGACA
AACATGACTAGAGCAGAAGAAAGCTCTCATATCAAGGGAGATTGATAAGGT
TGCAGTTGCTTCTCTTAGAAATGAGACAAATGAAAAGGAGACGtaaaaaaagga
gacaaaataataaatagtcTAGGAGAACTCATCCACACACCCTTTGTGATCTGCTAAA
GGGCTTCTAGAGGTCCATGGCAATGTAATCAAATTAAGGCCCTGCTACCTGT
CTCTCCTCTCtggctggctgcactgctgggctctCTGCTTGAGTCTCTTTTTGACTGTTGG
GGTATAAAGACTGAAAACCAGTACAGTACTATACGGCAGACAACAAATGAC
ATTTGAAGCGATTGTGCTTTGCAGCAAGACTTTCACCAGGTTCAACCTAAC
TCCTACCTTCAACTCCTTGAGTTCAAAACCAGCAAGGCACTGCTTGGAAGA
GAAACTTGTGCAAATAATTTCATGTCACACAGTGGACTATGTTTTTAAAAGG
GCTCTTCCTGTGCTCTTCTGTCTTAGTTACATCTCTGCACAAGCCATTCCTAT
GGCTGAAAAGAccacatgcattttttttttaatcatttatgGACTAATGTAACACCGGAGTCAT
CAAAGCTTGAAAGCCACAAAGATAAACAGGCTAAAATACATTACATGTCTT
GAGCTGTCCTAGCAGTTCGTTGCTTCCACTGAATGAAGCACTTTGCATCCT
GCCATTCCTACCGCTGGTATTTATTTGCCTTGGGCTCTGCTGAGCTGATGTG
GTCACACTGCAGTCAGAAACATAACATGACAAAAAAACA
>TCONS_00185981
GCTTGGATCTTGTGCCAGGACCTGGCTTCACATCTGACTCTCCTCTTGCTTA
GGGTGTTGGAGCTGAGCGCGTCTGCAGTGGATGCTGTATGAACATGAACAC
TGCTCCTTTACCTGTGGCATTTGCACGGGCATGGCTGTCCACCAGcagtATCT
CAGTTCCTGCTCCAGACTGTCTGCTGTGGCACACGCCTGAGCCTGAGGCTG
GTGAACACCCTCTCTCTGCTCCCCAGGCAAATGtcaggagctgcagggtggCCTCTG
GATTCCTCAGATACCTGCCCTCATCATGCAGGAAGGCATTAACCTTTGGTTC
TAAGTGAGCACCCTCATATCTCACCTCATTTAGTCTGACTGGCTATTTGATCC
CATCCAATAGAAGCATCTTTCCTTTGGCATCCTGTAAATCCAGGCCTTGACT
TGAGGATTTCCAGTGTGCCGTCTACCCAGTGCTGGTGGAAAATACCTCACA
TGTTGCGCCCAGCTCTTGGTAGGCCCCGGCAAAGGGACTGAAGGGGAGGA

TGGGCAAGAGGGCCTTGCAAGTCACTTACCTCATGCTCTTAGGACATGGTG
AAAGCACCACCAGATCTCAGCTTTTTCCCCAGACTTGAGCTTTTCCTTGCC
TCCAGTTCATACAGAGCTCAGGTTGGGCTCACCTTACTTCCACATAGCACTA
CTCTCCTGCGTTTCCCTCACATAACCTATCACATGTATCTCTTGGGTCTACCT
TAG
>TCONS_00186821
CTCAGAGATGCCTCGTAATGTAAATGCAAAATGTAgatgcttttctatttttctccgAACTT
CTGCTGATTTTTGCACTTTATTTAATGATGCTCCTTGTTCTGTTTCCCCCTCC
TGAAATCTTCCGAATGCCCTTACATCAAAGTGTGTGAAAGCTGGTACACTC
GTGCACGTTCTTTAGAAGAAATGTAAGTTCGGAAATGTCTTCTGATGTTGT
GTTTGCTCCGTGATTATTTTTCAGCCTTATAAAATTTATAGCTTAATTTCCCTT
CTCTTATCCTTTTGTGCTGCCGCCTTCGTTACCTTTCAGCCTTCACACTCTGT
CAGCCTGGTTTTCTGAAAACCCCGCCGTCTCCTTTTGATCAAAAGGTTTTT
GTTCCTGCACAGTGCTCTCAGCCTTCAACAtctgctgttctgcttcagCACAAAATTGA
CTGCCCGAAGACTCTTGTCAAATTCACGATGGCAGTGCCGACGCAGTCAGT
AGCATTGGTGCTTTCAGTGACCCCATAGGAAGGCTGGCTCTTGCCCTCAGC
TTGCCATTCACCCACACCTCTGGAGCGAGGATTTGCTTGGGTTTTCTGCATT
GCAGGATCGTAGGTCTGTTTGCACATCAGACTAGGCTCTGATAGCCCTGTC
ACTTTCAAACTTTTAAATCTCTtgaattttcctattttatatGTAGTTTTCTGTGTATGGTG
ATTTTAAACTACTTATAATAAAGTTAGTTTACTTAATTACCTTCCTGTGTGTT
GCTGGAAAGCACACCATTAGTTTTCTGCTAAGGTATGTCAGCAGGGTGGGT
TTTTGGTATGCGGGTGCCACCAACCTTCCCATTCTACTCTTTGGATTACAAA
TTCCACATGGAACCTGAAAAGTCTTTGCTAGTGCAGGTTTTGTTGCTTGCTT
TCATCAAAGCTGATGTTGTCTCATGACTAACTTTAACTTCACCGAATTACTT
TTCAGTATAGAACAGTTTATTAAAAATTCCAGAGCAGCAGTGGTTGATGGC
TCCATGTTTGACTGCAGTGGTGTCTCCTAGCAACTTGGCTGAAAAATGAAC
ATCCAGCAGGGATGCTCTGACTGCTACGTAAAACCGTGTTTCTCTTGATGA
GGCTAACTCAACCATCTTCACTGCTGTCCTCTGCACTAAATGTTGTGCCGTA
CTGTTGAAAGCGCTTCTCAAGATCAGCATTAAAGGTataatcttttaaaaagacTGCTG
TTGTTAGTTGTAAACAATCACTCCTCTTTGAGATTTCACAGGTACTGTGACC
AAAGCTTATATGGAAAGTACTTTTCAATTGACAAGTGGGACACGGTGACAG
CACTGCAAGATCTACACAGGCAGTAAGCCTGAAGGGgctGTAATTGGAACA
GTAGAGTTTGGCCTGCTGTATTTCACCTCAAGAAATCAAAGGAGCAGTTGA
AGTTGGATTTCTCCATGTTTTTATATTCAGATATCAACTCCACTCTGCTTTGG
GTGTGAGAATACCTCTGAGGTTGGACTAGCAAGTTCCTGTACCTGCCTCTG
GCCTTAAATGATTAAAGGTGCTGAACAGATGACTCTTGCGTGGTTGAAAAG
CACTATAAAGAGCTGAGGGGGCTTgaatcttgcttttttctgatcttttccTTTAGCCATGTTC
CTCTTGAACTAAACTCTCTTCAAAACTTCTCTTTGCAGATGaggctttctgttttattcta
gCCTGTGTCAAAGTAAATTTATAATGGGAGTTTTCTTTCACTTGCGTATAACC
TTGAGGAGCTTTTCTGCTGTTGACTGGAGCAACCTCACAAAGAATAGAGCT
GAACAGCTTTGCCTCATCTAatcatgctggaaaaaaatgtattgagTTCTCTTAAGAAACC
AACggaaagattaaaaatgcaACAATTTTGCACAAATCCATTCTCATTACATCTTTCCA
AGTAACCATTTCAAGCTATTATCAGTTGTTGAGCTTCATTTCTATcactaataaaaac
aatatgTGACAAATAATGGAATATCGATTTAGTGACTTGAAACTTGTGAAATTG

CCATTTTATTCAATGCATCTAtaggcagcacagcagaatgGAAGTTTATTGTAGAGTG
AATGAACCTATTGGTTTGAGCATGGCATAGTGAAATTACAACGCAGCAATA
AAGTTGCAGGGCACACGTAGGCCATCTGTTTCATAgtgggaaaataaacaacattGTG
GTGACGGCCCCAAACAATAAATCTggatttcaaaaagaaaagcagttggaGCTTGTAATGT
TAGGAACAGCATAGGGTGTTGCACatcctccttcctttccatgCTGTTGCTCAGCTGCA
GTATTCTCTCAAATGCTACTTGGTTTTGGCATCTTTAGTTATTACAGTATGTT
GAGTTAAGGTCTAAAAAGCTCTTCAAGTCACAGAACACTGATTCAACAAA
GTGTGGCTCTTCGTGTCTTGCTTATGGTCTTTatcaccttttttattttcagtgtaggCAGGGA
GGGAGATGGTCAACTTGTGGTGCTCCACTACCAGataagaaaaagagcaaagaggTCA
CAATCTATGAGCAAGAGGCTGGCATGAAACCAAGATTTGTTCAATGAtccatca
aaacaaaataagatcTAGTAGCTGGGCCCTGTGTtaccagaaaatgaagcaaCCAGAGTATTG
TAATAcaggaaaagtgtttttcttattaattcAGAGATGTGAAATAAGACGAATCTCTCATTC
TGACTGATCTTTAAACATTCTGAATTGCAGCCCTTCTGTTGTCAAATGTCCT
GTGACCTTAAAGAAATGTCTGAGAGGACTCACCTGGTAACTGTTGACATAT
GTAAATGCAAAATGTGACTAAGTTAAGTACTGCATcctttttcaattatttttgttgcattAGA
AATGTTATAGAATTGTGTCTACctagcaagaaataaaaaaacatctttcttctttGGTGAGTGGG
ATATTCTGCAGGCTATTTCTGCACAATCCTGCACTGTTTTGTGTCCCCCTTCC
CACAGCAGTCCTCTGTATCACACCTGGATGACCTTCTGTAATGCCATCTGGT
CAAATTCCATGTATTGACCTtttgggggaggaggagaagcagacAGAAGACGTCAGGGT
TGTTGAAGCCACTAGTTTCTGGTCCAGTTACAGGGCTCTGCCTTGGGGCAT
GTATATACAGTCTTAGTGGCAAAACCTCTCTGGCATTCCCTTCTCCATTCAC
CTACCTGTCCCCACTTCCTAGCCTCGATTCCTCTATCTTCCAAGACGTAGTG
AGtacacattttccttcagtctccccaaagcctgctgctttgcagagtCAGACCTTCATAGATAATTGCT
GTAAGTATACATTCACTTCTGTGAAAGGGGGTTTCCATCCTTACTGAGCTGT
CTCCATTAGGCATCTAGTCACCTGCCTGGCACAAGTTGGCGCCTGTTGGAT
GGTAAAtgagaaggatggaaggaacatgttttatttgtgtttaagCCGCAGAAGGCACggttttattgtatttt
tgttgtttttcttttcttataacGTTCACCAGCTTTAAGATGAGAGCTTGAAAAGCAccattaaa
atgaagaatggtTGCAAAGTAATAAAGAAACAATCATCTCACTTTAAGGACTTGA
ACTAAAAGCTAAATGACTGTAGTGGTTTAGACTGTAAAAGCAGTGGGAAAT
TTAATGAACACTGACCCTACAGGAAAGTCAATCTGTCTGTTAATGTTCAGTT
ACgttatttcttccaaaaagcaaTAAGCTTCTTTTATCTGATCTGTTGGTTATGGAACCTG
GGGTCAGACACCCGTTGGGCTTAATTCTTCCAGTTTGTCCCACATGAAATG
ACTGACCTTTTTGGTATATCTGCTAAGTGTCCCAAGCAAGAAATAATGTCTC
AGGAAGATAATATGCAGGTTCTTAGGAAATGGTTATAGTTTTGGACAGATTG
TCGTAGACTTGACTGTGGACATTCTGAACGGTCTGTAAATTCCCCTAATATT
AAGCATATTCAGCCAATTCTATTACTTGGTTGAGGCAAAATATAAAGAACAG
AACTAAGATGGATTAACAGTGGCTGGGTGCCCGAGTCCTGTAACAAGTCAT
TGAGCAAGATGCTGTACAATATTTATTGCAAATCTCACAGTAAAAGTGGCAA
TCAAATAAGACATAAACATATGAAGCTCTTTGCTTATGTTAAACTGTTaagaaaa
tttatttacttaacCAGGTGGTAGGAAGAATGTGTGCAAAAGGGCACCTTGACAGCT
AGCTGatttaaagctgaaataattCAGCTGAGAGTAGTATATCCCTTTTCCAGGCTGCA
GTGTAAGAAACCTTTGTAAAGCAAGAAAGCTGCTTGCTTTTGCTGAGCACC
CTCTGCATGAGATGCTGTGCAGGGTGCAGGCCCAGAAGGGGCTGGAGAAG
TCTGCCACTGTTCTTGCCACGTGGCAGAGCCAGCCCCTGGCAAATGGCTGT

caaaaatcacagaagcacctccagctgcaggcagcaatcCTGCTAGCGTTCTGCCTGGTGCATTA
CCTGAGcctcttctgctgcagtgtAAACCCTTTCTTGTCCAATCTCCAGTGGATATTAA
GAACTGTTTGCCATTGCCTCCTAAATTTCAGAGGATTGTTATCATGTAATCAT
TCATCTTTCCTCCACGTTGTAATAATCTGTTTTCACAGAGTCATAGAgtggctcgg
gttggaagggacctcaaggatcatgaatctccaactcccctgccgcatgcagggccaccaacctccccatttactactaga
ccaggctgcctgaGGCCCTGtacaacctggccttgaacacctccagggacagggcatcacagcctctccgggcag
cctgtgccagcacctcaccactctcatagtaaagaactttcccctgatatccaacctaaatctttcctccctcaacttatttttgttt
ccatttctacATGTCTTTCAGCTTTTGACAAAATGAGTAATTACTGCTCTCAAGCT
TTAGCCCAGCAAGCAGATGCAGTGGCACATGAGGGGGAGCACAGGGAACC
AACTGGAAAAGCATTGTGTTTGAGCACTGTGCCAAAGGTGCACTGTGCAT
CTTGACAAATTCACAAATGTGGGTAGTCTCTAGTCTTTAAATGAGTTTTAAT
CTGTGGCCTGGTTGGAACAAGTCTTACCAGGTTGTGGCCAAATTAAGTAAC
AGCCAGAACAGATGTGACAGGACTATTCATATCGAGGGCCTACTCTACGAA
GTTATTTATTTAGGccaatgtttttgtttggcttgaTTTTAATCTTATGTGCTTCTTAAATTA
GAATCTGATGGTATTTGGTGATGCTTTGGGTGGCTTTGCATAGCAGCTTGTC
CTGCGAGAATAGGACTGTGTGGGTGTGGGATAAGACTTCAGAGGTGGCTG
AGCTGGCAACACTCACCAGGCCCCAACACTTACCAGGCCACAACACTCAC
CAGGCCACAACACTCACCAGGCCGCAACACTCTGGGTTTTTCTGGTTAAC
TCATGGGGATTGTCTGGAGACCAGACATGCACGTACCTCACAGTGCACAAA
TGCCACCAGAGGTGTATGAATTGGCTGCATACTGAATGTCACActaaatctgtgtttt
cttccttatgaTGCTTTAACAAAAATGGTGTCGCTCTATTTACATATACATCTCAGG
TGTCAAGTCTATTCAAGTTTATGTTCCTTAAAAGCTTATTGCAATTTAGGGTC
CAATATATCTAACACAACTAGAACTTCACATTTAACAAGTTATAGAGCTTTCT
ATggtttccttcattttccccttttttgtCTTGCTGGCTGACAGTTGGTACTGCAggctgaaaaacagc
ttttccaggCAGCAGCATCCTCTTCCATTAAGCAAATCTTGTTGACGTAAAGGGA
ATTTTCAGGCTGGCAGTTAGCTATAGCTAGCATTCAGCAAAATGTTGGTAAA
ATACAGTCTGGAGAAAGTTGTTGtcttcatgaaaatgaaagggCCCTAGGGGCCCTGT
CCCatgtgctgagcagggctgggatgggatAGTGGTTGGGGCCACACAGACCTGAGGACT
ACAGGGCGCATCCAtagggagcagcacagcttttgctttccatcatctcCACATCTGAGCACAG
ACATCAGAAGCACACTGCAGAGATCAGTGATCACGCAGTGCACGTGCGCA
GCGCGGTGCATGTGGGGCACCACGTGTCTGAGTAGTGACTGTGAGAATGA
CTAATCCAATTAGCTACTTCCTCCATTCCACAGAGCTGCCCTATCCTTTTCAG
CAATTAAGTATCATGACACAGTCACATCTATGCCACGCTAAAAACACTGAC
CTATCTGTGGCTGTGAGAAGCAAGCTGCACCCAGTCCCTGTCActgcctgctgggc
tgccctgggagtCTGTAGCATCCTCCCCGCAATCCTGCCCTTATCGCATGCTGGGT
GCTCCTTATCTCACCTCACTGGCTGATTCTTTTTAAGCTGTTGCATGTGAGA
AtgtgtttccttccttccaacaGCATTAGTCAAAGCAGTTGCGATGCGTTTTGTCTCTCAT
TTACCTGTCAAGATCTGGTTAACGATGCTCTCCTTCCTAATAGCAGATGACA
CTGCAAATCAGCAGCAAGGCCCAGAGCTGGCAAGGAACTTCTGTGTAAGG
CAAAGCTGGGACCAATCACTCACGCAGAACCGAATCACTCAGAAGAAGAA
TTTTTCAGGTGCATCTGATAAGACAGTTGCAAAATATGCATGGCCTTTTGGA
GTGTAAGACAGAATATGAAATACGTGGAGAAAACATTTCTGGGTGGCATTA
TCAAAGATGTTTAGCATATGGTTAGCAGCTTTCAGTCAAGCAGAAGATGAA
CCTTTTGTAGGCTGTAGTAGGGGCCATAATTTCCCTTCAGAGGCACATTTGG

AAAGCACATTCAGGACAACCGGGTTAGGAGGCGGTACAGATGTTTGTGAG
CTGAACAAAGGGGATCACAGGCCTGAGGGCAAGATGTAAACGAAGATACT
TAATTGCAGAAGGGTCAGTGCAAGTAAGGATGCGAGGCTAGAGAATTTTAC
TCGTATGATTAACCTTCTTTAAGCTGCTGATAAGTGGGTTGCTTGCCTAAGG
TGAGTTGTGGCACTGAGCctccatttctttgtctttttgagTCTctgaaatagaggaaaaaagaatacatg
AAGTTTTCTTCCAATGTTTAGTTGTTGTAGGCTGGATAGCAGCAGAGGGTC
ACATTAGGCTCCATgagatgaaataaatgtaatgcaTTCTGATTATGTGTTACTGCTATAA
GCAAAACAGCAACCCTTTAAAGAGTAAATGCTCTTTCTATAAATACTATGCA
TATATTGTAGAACGAAATTCCCGGTTCATGATCCTGAttcttttttagtattttctcaAAGG
AATGGAAGTGCAATGGGTGTTGCCATATTGCCCTCTCGTGGGGTGAGCCTG
TAAATAGCAGCCAAGCACGCCTGGCTGCAGGCTCTCCTCTCGGTGCCGCTG
GGGTTTTCCCTGTTCTGGGGTGCACAGGAGTACCCCACCTGAGGACACGG
TGTTCAGAGACCTCCCTGTGGGAAACAGCCAAAGAAGTCCACAGTGTAAA
CGTTTTCTCCTAATTCTGTTCCTGCACTTCAGTAGAACACAGCTTTGCGGTT
GGTTCCTTATCAGAATTGCAAAGACCAGCCCAGAGCGTCAAAACAATAAG
CGGCCTAAATACAATAAAACTCCTCCACTGAGCTCTCCTTCCGTGGGACTTT
GCACAGCAAACACCAATTAAAAAGCAGGAGGAtgaactgcagtgctgctttgcgTTACA
GAGCGTGCCTTGAAGCAGCTGTCAGACGTGAAGATAGCTCACTGTGAATG
GGAGTCTATAGGCAGATccattttgctgtgctgtgaatgATGCCATCTATCACGCAGATCG
CTACAATCGCCTCCCACGTTGTTATACCTCATCTCTATTCCATGCTGTTCTCC
AGGAATTTTAAAGCAGCCTTCTCCTGTCTGGCCGAGGTGCTTTTGTCACCT
GCACTTAGAGAGTTGTCTGagtgctttgcagcctctgaAATATTTATCGTCTCCCTTCTC
CTGTAAGGCAGGGAAACTCtcaatttgcagatgacagagCGAGGCACACAGATTAGTT
CTTGGATCCAAAAAGAGGCTTACACATATAAAGTGGTGCTTGGGCAGAATT
TGAGAGACTCCGTCCAAAATGTACATTGTCAAAAGCCCCATTCAAAGGCTA
GAGATGTCTGTGTGGGAAGCTGCACAGCCTGAGGGAGAGGAAAACTAACA
GCGGTTCGACTTCTTGTTACAAGCCCTTTATTGATGCTTATGACATAGAAGC
TCCTGAATAAAACTGCCTTCAGAGCAGACTTCTCATGCAGCCTGCTAGCTG
CCTcagtggggaagaggag
>TCONS_00187288
GTGAGAGGCTGCACATCAGTATTCAAGACGCAAAACGAGTTTCTAGACTGT
TCCCAGGCATAACCTTCACCAATGTTATATGGAGATCTTCATGCAAACTTAT
CAGCTTTGAAAAGGGACGATGAGCTGTTATGAATTGTTCTGTactggaaggaggaa
gaggccTGTGGCAGAACTACACAGACAAAAGCTTTCCTTTGGAGCTTACCAG
TTTGACTAGGACTGAATTGTGGCAAAGGCCTGGGGGAAAAACCCTTTCAA
ATAAGTCTAACATGTGCATCTCAGGAAAAGCTTGATGGAACCTAGCAGTGT
GAAGATTGAATAGCCTGGTCAAAAAAAGACGAAGATAAAGGAATGATAGA
GAAGTAGTCTGAGTAATTCCTCTGGAGGATGTTTCGAGTTATATTTGCTGAA
GGCTGTTTCATCTTCCTGGCAATCTTGATTGAAATAAAGTTGTACATACTATA
TTTCAAACATCCTTATCTATGGTATACACAAAGAGCGAATCTTAAAT
>TCONS_00224283
gccttgaatgcctccagggtgggggcatccacaacctcactgggcaacctgttccagtgtgtcaccaccctcatGGTAT
AGAATTTCCTCCTCATGCGTAATCTAAAAGAAGACTGTGGGATGAAAGAGA
AGTCCTAAACAGATCTGATCTTCTGTCTGACACAGGGAAAAAGTTTTCATG

GGGGTATTCCTTGTGCAGACTGTCATCTCATGTTGAAAGCAGCTTTTACAG
AAAGGCATGTGGTCTACAGATATGGAGAATCTGCCATTTTCCTGGGTACTTT
TATTACAGTGGTTAATTGTCTGTGCTCCTAAGAAGCTGTGCCCCAGTGCTAT
GCTGGATTTGCTGGACTTCACCTTTGGGTAATTAGTCCTTGTTATGTCTCTCT
CAGCTAGATTAAATATCCCCTCTGTAGCCAGCATTTTCACCTCATGTGTGTAA
TTATGCCTCGTAACCACACTGCTTTTCAATCTTATTTTTGATTAACCAAACCA
GATTGAGCTCCTTCAGTCTTTCCCTGAAAGGCAGCAGGTTCTCCTGTCCTT
TCTTCACCCCTAGTCTTTGAATTATATTTCACGACTCCTCTCTGCCCTGTCCT
TTCTAACAAATGTGTAGCAGAACCTGACTCAGTGTTCTTCAGTCAGCACTA
ATACTAATTGTGGGACATGAGCTGTGCCAGaacttcttcagcttttttaagaacaaaactaaatga
aacaaaaccaaacccactcTGAACTCCAAGCTCCTACAGATCTTTAGATATCAATATAT
GTTGATACCAAACAGATCATGTTCTGCTGTTATAAGTCTCAGTACTTTTCCAT
GGCTGATACAGACTTGATAAGCTTCATAGACAACACAGATCTAGTTTTTGGT
ATTTTACTgtattgttttgctgttgttgttttaagtaGCCCAGGGACCcacattattattttgttaaCTGTCTG
TAATTTAGTCTTCAGGTTGTGATATGGAATTGTTCAAGCACTGGACAAAGG
AATAAGTTCAATACATGTTATTAACCAGAGTAGTTCAAACACACTATTTCTAT
TCTGGATTCATTTGTTATACATAAGATGTCGTCTTATCACTTCTCTTAATACAT
TACCATCAAATACAGAGGGTGACTGGATGTAGATGAGATGCTGCTAGAGAC
ACATCATCATTTTTTCAGGATCCATTGACTTCAGAGTACTGGATTTCATGCCT
TTAATGTTGTGCTAAACCAACAGGCTTATGCTGAGGTATAAATATACAGGAA
TCACAGTGTGTGTTTTACAGGTAGATCTAATCTACAGCAGCACTCACCTAAT
TCTGATGTAATTAAAAACAGGCAGACAGgatgacagagaaaaatattctgactAACCAGT
ATTTTGTTGATGACACACACTGCAGGAAACATCAGAATAAACAGATTTAAG
CACATTTAGGAGCTTAGATCATATATTATTTCATTCTGTCTAtaacacaaaaggaaaga
atctCTCTGCCAAGCTTGTCTGATTGCTTACGACGAGATTTGGAGGGTTGCCT
TTTGAACAAATGGAggtttttctcttcagtgatCCCAGTCTGGGTGCTTCAACTGGGCA
TCAGTGAAATGTGACACAAAACTCCCTTGATCACAGAGCAGGCTTGTGAA
GGAAGATGAAATAGATGGTTTTGAGACAGGATGAGAGAGGCACTTTTgtacta
aggaaaaaaaggtaagcCATTCTGTGCTGTATGCTAACTATCCCATGTATGCCTTAAAa
ctgggagctgggattgtttgtGTCAACGTCAGTCTCCACAGAATGGCACAGAGTGATGG
GAGCCTGTTGAGAAGTGCTCTGCGTGAACCATTTCTGGGCTAAGCCAGagG
TCAGCACCTGAAAATATGGGTGCAAGCTTGCAATCCTGGATGCAGTAAGGT
CTGCAGTGttctgcagaaaggagaaaagaaccCGACATTCTACCTGGCACCTATAGGCAA
ATCATCACTTTGGATAAGCCACAGTGCACTCTGCATCACTACTAACTCATCA
CTGATAATGGTTGTAACACCAACAGatgcttttcaaaattctttGCTGCAGCATTTCACC
CAAAGCTTTCACTTGCTCCTTTTTGCAAATAATTCAGCAAGTGATTCAGTAG
TTCTATTGTAACACACTGGGCAATGccatctctatttttttttgtttcttgttacATGCCAATGTA
CCTCaactttttaataaagaaaacctGCTGAATGCATTCTGCAGATGATATTTCTCTCAGA
ATACAGGTACAGGCTCTTACTAAAAAATTAGCAGGCTGGTGCATACAACAT
CAGTGACAATCTCACTGAATGACACACACAACAACTTTAAACACAGGCTG
ATGCCTCCCTCACAGAACAACAGCAGCATGCTATTTACGGAGAATTCAGCA
ACTACAGAGCAGCCCGTGGTCTCTGACTTTAGAGAGAGACCATATGACCTC
AGGAGTCTATTTGCTCTTCTGATCAGTGCAGAATGGTTCCTTGACTACTTTT
G

>TCONS_00236753
CTCCCACCCCACTCTTCCTTGATCCCTTCCCAATTCCCTCTGGCTGCTATTGC
TTCTGGGATAGCAGACTTTAATTTGAGTTCCAGAGTTGGCAAAGGCTTCCA
ACTGCATTTAAGCCTGGTTTTGTAGAGGCTTCGttctttacCTTGCCCTGCCGCA
GCATCAGTTTCTACACAAGCTCAGGGAATCAGGAAACAAGGAGGATAAGG
GGAAGTTGACAGCCTGAAAAGCACTATCAAAAGAT
>TCONS_00271065
GAGCAAGCccttgaaaaacagaagtaaacagCAGGGGCAGAGATCCCTCTACACGGAC
CAAGCTCAGCAGTGCCTGTGTCCCTGCTTGTGTCCCTCTGCTGGGGTGCTG
CTTCAGGGATCACCCAGCTGGAGAGCTGATGGTTGGATTTTTGCCCAACCT
TGGCCTGGGGTTATCCAGGGGAGGAGGCCCATAGTCTCAGGACTACACATG
CTTGTGAGCTGCTTGTGTTGTGTATTCTGTACTAAGACACAGGTGGGTTTTA
TCCAACCCTGTTCCTGTTCTCTTTCAGAGTTTTAACTAATCTCCTACAAAGT
TCCCCCTAAAATCCTGTGATTATGATTATCGAATCCTCACTAAGCCAGTCTC
GTGCCAAGATAACTAGTAAGTCTTTTGTCCTCCAGAAGCCTCTTGTCAGCAT
CTTTCATCTTCTTAAGGGCTTTCTCTATCTTCAATGAGCAGCTGAGGCTCTT
TGTTATGCAAATAGATGGTAACTGCTGCCAGAAAGTAGTCAGAGTCCGCAG
TGGCAGCCATTAGTCTTTCAGAACAATCAAACTCCAAACTAATCAGAGAAA
ATACACACAATGTAACCTGTCTTCCACATTATGTTCCACCCCAAATGCGATC
ACactttccttctccatctctcctcctcctgcaagCTAATGACATTGCCATATTTCACTAGGAAGA
CTCTGCCTATGTTATGTCTTTACATAAAACCATTTCTGAAATTGCTGGAGAG
AAATAAGTAAGATGAAACATTGCTTTTTTCCTACTTGCTGTATCCCATCATTG
CTTGTGGCCCCCTACACAAACATCAGCTCAGTTTCAGCTCCAAAAGGGAG
GCCGTTGTGGCTATATCACCATTACCTGGCTGTTACCTGCAGCGTGGGCCTA
CCATTACTCCGACTGGGAGATCAGCATGTTAATACCCTGCAGTCACCCtctgaa
agaatgaaagcaggACACCAGGTCTGATTTCTCACTGACGTGCACCAAATCCAGTA
TCAAGTAACAACAGGGTCCGCAAAAGAGACATGACCTACACTGGCTAGCA
GCATGAGATGTCGTGGCCACAGAAAAACACTACGGTTGTTATCATGGAGAG
AGGACATTATTCATACAAGTTAATGAGCCAGACAGCAGGAACAGTCTGATA
CTGCTAGGCAAAGAAACATTCCAGTGTTTGAGATAAGGTACATGTCATGCT
GTTTAACTATCAGTAGGGCAAATCAGGGCAAGCCAATCTAACAGAGCACAT
TCAGCAGCAGATGTTCAAGAATTAATTGCTTGATTAATCATGACACACATCA
CTGCCTTGCTCTTAACTCACTGAAGCACCCAGGAACCGAGATGATTTAgctatt
tttcaaatacataatCTAACTAATGCAAGGTATTAGTACAGGAAATAACTAAACTTAC
TGTGAATGTTGTGAAAGGGAAAGGTTATTTTTAACAGGTTGTTTCACTCA
CACTCATTCTCTTATATTTCAGGCACCAAAGATGTGCGCATCAGGGCGGATT
GACCAGAATCACCTGAGTGTAACACAGAGGATGCAAAAAAATTACTGTGT
TCTCTGCATCCTTTGGTTGGCTATGCCAGGAGTTTTACTTGGGCTTCTTTTTT
TGGACAGGTCCAATAGGAGTCAGCGTTAATACTAGCATGAAGCTTGCTTAC
TGTAGCATTCTTAAGTCTTTAGACTTGTTACTTCCTATTTGGGATCTCTTTAG
GGTTATAGTCTGTGTCCCTGCtgatttaaaaggaagaaaatgcacGTACATGCTGAAATCT
CATTATCATACTCCTGCTGCCAGACCATGTCACGTGGTAGGAAAAGCAAGG
CTACTCCTCAGTGGAAAAGTAGGAGCCAAGTAAGCAACAGGCCAGCAGTA
CCTGAGCAATGTAAGCAGGTCAGGCTTGGAGATGGTATTCAAGACAACCC

AAGAATCCAAGGCATGAGACAATACTGTAGCAATGCTGCAGATTCAAGACT
AAGCTAGTCCACATGTAGAATGCAAGATCAAGGGTCTCCATAGACATGCAC
AGCGGTAGCTGAGCTAAAGACCAGCACTCTACCAATGCAGCtcagacagaaagcaa
atgccCGGGACTTGGCTTAAATAGGGCTCAACAGCCTCTGCTGTTGTTGGTTG
GAAAGTCCCTGATAAGGTTGGCCGTGTCCTTTAAGGCTTAGTAGCGTCCTC
AGGGCCCTGACACTGCAGATCTGCTGATAAGTCTGCTCTGCTGTAGCCATG
TTGGACTGCAGGTCTTCTGGCCTGATAACCATCAGcagtgggctgctgctgctgttagtcaa
cattttgttgctgtatgacagatggcagcagaggggcaatctgataaaatggcatctgacatggaagtgtgtatgaagcaaa
ggtgtgacattgaattcctctgtgcagaaaaaatggcatacACTGatattcatcaatgcttgctgaatgtttatggagaccc
agcagtggatgtgagcacagtgaggcagtgggtggtgcatttcagcagtggtgacaatgaTGTAAAAGtcaagcc
acattctggatggccatgcacagctgtcgCATCTTGAAATGAAGTGTGTCTCAATCAGTTCAT
CTGCATAAATTGGATAGTAGTGATGACTATTctgaaaaatgatgttttgtagctgagaatttgctcta
tcaattagtgtcattgtgctcttcgtagctgttgtagtttccatggaaataaataggtgaCATTACTTTCTGAGCA
ACCTACATTTATATGCTGAGCTTCCCCAAGTAAATGTAAAATAGAATCTCAA
CCAAAATTCATATGCTCTGCCATTGTCCCTGTGGCAAATATCTGTTACTTTAG
TAGACCAATGCTATGTCcaaatcattttattgcaaTAAGAAAGCAGCCATGTTTTGAAA
GGATTTAACATTTTTAGGTAGccttctttcattctttgtaGTATGGGACATTTTCTGGAGA
TTACAAGGAGCTGTTATCAGcttttatctgttttctgtttttctggttttctttttcctttttttttttttcttttagtcata
ttctttcctatttttttgtcctttctctttttttcccttattctttcttttctactttctttttcctttttcctttttcttcagaatggtCTTATT
GCAATTCTGAAATCACTTCCTAGCTGAGCAACGCCTATCTCAAAACACAGA
TCGTTAGTGTGGATGCAGCATAATCAGGACCAAAATGCTGGACAGAAGAGT
TGGTAGAGCTAAGATATGAGATGTTGATTATAGCAATTGTAGATTTCTAGGA
AGTAATTAGGTATGGAGTGACCCACTGGCATTTTATATTACAAATTGTGATG
AAGTACGCATGGAAAGtattataaattttattttcaaaatgacatTATGCATCAAGACCACTTA
AACTCAAAGGGCATAATTAGACTGCATCTCTCTGACAAACTCAGGCTGGTT
TTGTAAGGGattgccagagacaaatctTAACATACATGGGAAAGTAGAAATCCCTTTG
TACATGCCATGGGTTAAAAGATATTGTAAGAAAGCAATGCTTCATTAATGAG
TGGTCACAAAATCTCttgaataataaaaacaaaacaagttattCTGAAGGTCTCAGGCAGAA
TATAGTAGATGAATAAGATGTAATCTGCAGTATAATAAATGTCTGCCAGTTAT
AAACAAATCATCAGCTGATACTTACTTCAAGATGTCTAAAAGCATCTACATT
CCTTCAAGGCTGTTTAGCCAGATGTGTTTATCTGTATATCTTACTCTGAGACA
GGAAAAATGTCATCCTTTGCAATGAAGAATTCAAGGTATGCTTTATGTGTGA
TATCATAACTATGCCTGAAAACTAGAGTCTCCCACACAGAGATGTCTGTACT
GGTCTGTCTTGGTGTCTATGACTCTAAAGCACTGCAGATGTGCTGGGTAGAT
GCATCTCACCTCTTATCACACCTCTCTTGCTTAACAGACTACTGCTCATAGAT
ACAGGCACAAAGTAAGCATGTGATCTGTGTGGTAGATTTTTCCCTCTCGTA
AAACAGTGCTGTGTGGATGAGGGCAGATATGGTAAATTGCAGGAGGCTGTC
AAAATTTACATAGCGTGCTGAAACCACAACTCTGAAAGACACTGCTCATC
TGACACCTCTAAGCATTTCTAAACTcaaacaggctttttttttttttaatgcctagGGCAcagtctccc
ctcttttctgctgcttcttagGTCTGTGGAAATTTGTGAAGTAGAGAAGAGTGACAATGT
AGTAGCTTTATACAGCACTATGTGTTCCGAGAAAGGTAAAAAACTAAAGGA
ACTTTCAATTCTTTACTAAGGCATACATGACTCTGCCAGAATTGTTCTTGAA
GATGACatttttggaaacattttctgctgtcttcaaCACAGCATGTACTTCAAAATTATTGAAA
ATTAAATCCATACTGAGAGAATTCCATCATCTCCTATGCCAAGAGAACCAAT

CCATGATGATGTGTCAATAAGTGAAAATAGACTCTTGTCAAACAAAACACG
AGAGAAACTATAAAATTGTTTTACTGATGTTTAAAGTCATCTGTCAACTTCC
TTTTGTTTACAGTTCAGTGACTTCTGGGCTCCTTAGGTActaaacacagctctgcagtgc
aacTGCAGTGAGATTTATGATCACCTTGGTCAACGGTGGCCCATCTGGGATTA
AAACCTGAAAGGAATTAAATAAACTTAAATACACTCTTTAGGTCAATGCAA
CAACAACAGCATCTTAATTAGAATTACAGCAGTATCAAGCACAGTGCAAAA
GGctttcatatatatattctctTAATGAACAACTGTTTTATAGCTAAAGCTTTTATTGATCC
TTGGACAGGAATGTAGGCATGACATTTTCACTGACCCTAGAACAAGAATGT
GCACTATGTATAAGAAGCATGTGCAATAGTTGTCAAGATGTCACATCTAGAA
AACACTGTTTACGTTATCAGCAAGGTGTATCTTGAAGgatgttgaagaaaaaaaggaact
gcCCTTTGGAAAATCAGCCAGCCACTGTACAAAAGTCACATAACTTTTGATT
GCAGGTAATTGTATATAAACTTATTGAAACAGAACGTCTTTTACATTGTTTG
CTGGGCTGCAACAAAATTAGCTATATGTGGGTACCATTGTTTTGGAACCAGC
TATGCAGAATGAATCTCTCTGGAATTCCTTAATGCCTCATTAGCAAGTATTAC
CTCACAGTAATTAAGGAAACAAACCGTAGAAGGAATTGTAGAACAAATATT
GTCAACCCTGCACAAATACATGGAGATCTTTGGATTTGACACAGTAACTGG
GTCACTGATTTAGCATTAATGAATTGGTGATTTATCAGAATACATTTAGCTTC
TGGCAAACTACATAGTCAACAATATAGCAGTTCAGTTAGCTGTGTGGTAAG
GAAGATAAAAAGAGCAACCATCTCCAAGTCAGTGTCACAGATTCAGTGGaa
aaagtagattaaaaaaGATGACAGGTCACAGAGATTCAGGGAGGATAAAAAGTCCA
TTCACAAACTATGAGATGGGAGTAGCAGAAGAAGAACAGCACAGAAGACC
CCAGTATGTCAGGGCCAGCTGCTGTAGGAACAAATATTCTTCTCCAGAGCA
CTTCAACATGCCATACAGGACACCTTGGGAGACACTGTACAATAAACACAG
ACCCTAGGGACAGTACTGGAGGTCTTGCATACCTTAACATGTTaggaaaacctgaa
aaattATTTGTCATAACTAcatgcacaaaagaaaaactgaaattgctGGAATGGATCACTAGAG
AGGCTCTTGCTCTTAAACAGAGActggctttttaaaattaactttccaGCATGAATTCAGG
GAACATATTCCTTGCTAGTTTAATTTTCTGTCATATTAGTACTGAGCAATAAC
AAGATTAAACAGAGTTGCTAGTAAAATTTAATAGTTACAACAATCTTAGAAA
GCACAGCTAGAAGTGGCAGTGAATAGCTGATGCAGCAGTTTCTGGACTTTT
GCTTCTAGAAAATCTGGGAATTTCTGGGATCATTGCCAAAGGACCTCTGAG
AACCATTTGGGGAAGTCTTTGAGGCACTTGATGGTCCCTGGTGCCCCAGGA
ACAGGTAGGTACAGGAAGACTTGATTTCTTTTAAGATGTAGCAGGCCTAAA
GAACTCTGTCAGCCCTACTTGATTAAGGGGCCCTTGGagcttttgtttcagctgaTGCT
GATCAGGAAAGGTCTGCCAGCCCCTCAGATGACTAAATAAGATCTGGGTCT
AAAGTAGGGATTCTAGGGTTACTAAGCAGGCAGAGCAATTTTAGGGGTGGT
AGGTATATTACCTGCTAATAATACATCTGTAGTAATGGTGGGAATGTTTTACA
GTGTAAGAAGTTTAGTAGCTGATATAATGTTAGAGGCCTCAACTCAGGTAG
AAATCCAGTAGGAAGATGTTGGTTTTGGACAGTAACACCTATCTCACCTTG
CTGGGCAGTGAGAGGTCCTTCTACCTGAAGGAGGTATGTAACTGTTGAGGA
GGTGTATTTGGTGCAAAGGAGATGCAGGAAAAGGTTGGCACTCTGTGCATA
ATCAGAGATGGAAGAGATTGGCTGGATGTTTTCTAAGACTCTATAGCTTCAA
CAGCTCATGGAAGTACATTCTAAAGGAGGGACAGACTGAGTTCCTATATTA
AGGTGGAAAGTGTAGGCTTTTAAGGTGGGAAGGTTGGAAGCTGCTGACTT
CTTGCACTAGGAGGAAAGTTTGTGCTCTGTGTGAAGTCTTACAGAATAAGT

ATAGTGTCTTGGGTGGAGTAAAGGGGCTGACTCCTATGTCAAacaaaacttctgaaa
tgacTATGCTTAAGATTCATAGGAGCATCAGAAGGAAACACTGCGTTAGTGGA
GGCTGACCCCTCTGTTATAGGGAACAGATTTGTCTACTGGTCTGTTATCTCT
AATGTTTGACATAAGTGTTAACTTACAATGCTATGAAGAACCTGCGGAGTCT
TCTCAGGCTCTCGGGTTATTGTTCCCCTGAGAATTTGTCATGG
>TCONS_00271643
ATCTATTGCTTTGGAGACCAACCCACTTCTCACCCTAAAACACTTTTGATTG
CTGGCAAGTCTCAGAAGCATCTATTAAAACTGCATCTTAAAATGATCTCCAT
CTACTCTATGCACTGGTTGCGTTGGTCAACTGTTGAGATGAGTATTTATCCC
ATCTTGGTCCCTCTAAGGCAAGCAGCACTCAAATCACCTTGAATTTGCTGT
GTTATTTAGTGTTTTCACCTTtcaagttaaatattttttttcttttagcctAGACATAACTATTGT
GAGTGAACCCAAAGGCTGCATAAGAAAACGTCACCACAAATAGTTGTTCT
CTGGGTTTAGGTTTAAGCCACTGCTCAATAAAACATGAAAGTAATGTACATT
CAATGAATTGTTTCACTTAGCTAGGGCCCATGGCAAACATGTGTTGCATCAT
TTTCTGCTTATTGGAAATGAACAATTACAGAGGCATAACGTATTTTAAACTA
CATTCTTAACAAGAAGCTAGCAAAAATCCATATTCCATGAGAAGcttagtttttgtttg
attACCTCGGTAgtaaagcaaacattttcactgaaaagaggtgcaaagagaaacaaagttttTGATTGGAA
AAATGTCCTCTGGAGGACTGAGGCTCAGATTTAAGGCTCTCTGGAGGGTCT
CAAGACCAGGATCTCATTGTGCTGCTGTAGGGCCAAAATGAGCATTGAATT
GAGGTGAACCAAGCCCTTACATCATCACACTGTTGCAAGACAGACTGGAC
AGTATGttgaagacattttctttgAGTGTGAGACTTCTATGCGTGAGGAGCACACCGAA
AGGTGAACATCTGATGATGGGTAAGGCAAGGACTTCCAAAAaacctgtttttaaaatg
tatattgtAGACGAAATGCTCACTGAGCCAATATTGTATTAGATCAGTGTTCAGT
GCTCTTGAAATTCCTATCCCATGAGCATTTCCAGAATAACATCCATGCATAG
GCAGAAgtagtgctttttttgctttgtttctgctctgcaCCTTGCATCGAAAATGGAATAAAGCCT
CGATCAGAACTCTTATTTACCCTGTTTACAGGTGTATGTAAACCGCCCAGAT
AACATATAGGCACACAGTCAAGTAGATATAAAACGAAGCAGATAACAGGTT
GAAGTTTATAACTCAAACACTGATATCTTTAAGAACTACTTTAAAATAACTT
ACTTAGTGAAAATAATCACATATTAAAGGATCGCATAAAGGATCTCATTTTTT
GCTAAAATAGATACACCAATAACTGAAGAAGCAGTTTTCAAGTTATTCAGC
ACTTTTAGCTTTTTTACTCGAGTCCGTGCTTCTGCAAAGCTTCCAATTTTAG
GccagtttcatttctgtcaaaatgaaataaataaaaaaccccaccaaactGAGAGCAGTGGTTTAGATCT
TCTGGAGTCTGTCTGTTACTGTTTCTGTACTGGCATTTAGCAGTGGCATCTA
AAGAACCAGATGAAGATCAGAAAGGAGATAAGCAAAGTAACACAGTTTTT
TTGATAGAGAAAAAAGAGATACTCGCCTATGTTTCAATGCTTTGCTGAACT
GGGACCTGAAGTTATTAACAGATGTAAGTTCTTTGCTCTCACGCTTCTCAGC
TCCTCCATTCTTGTGGTACTTGGGAGAAACCTGCCAAGTCCACAAGTTGTT
TCAATCCTTCTCTGCACTATGTGAGTATGGTGAACTTTCTTCTTGGTACTCA
GTCTGGAAGTATGGAAACACAACACAGGAACATAGAGCACGTCAGGTTTA
TTAATGCTGAATGTGACCAAGCTTGCATGCAGTATTTCATGTCTTTCCAGTA
CTAATTACTGCATTAACTCTGAAGTACAGCGGAGACTTTCTTATTTCTGATG
CCATTTATAGGGTGTTTGATgtctttcttcctgctgtgaTTGCTCTCTCGGGTGAATAGA
GACTATACTCAGCTTGCTCACTGAGAAAGAGGTGCTGTGAGAGTTCGTTAC
CTCACATTTGGAAAGTAAGGGTAGAGATTTAAGCAATTGCGATGCTGCCTA

CTTACAGGGGCCTATTCTGTAAGCTATGGGATTTCTTCCCACAAGTGAAGAT
CATGgatgttttctgctgttccttGGTTGCTGTTGTGAGGCCACTatctttctgcatttcaaacttctgctgc
tgcctacTGAGGCATCATGATCTGAAATCAAATCATGATTTAAGTCATGTCCTAT
ATGAGGAATTGTTGCAGCAAGTTGCAGAGAAGTGATTCATGTACTGCACTG
TCTGCCAAGTATATTTTGTGAGGcggatggaaatgaaaaaaggaTTTTGCTGTAGTGTG
AAACCTCTCCATTGACCGCCTGTATCTGAGAATTGGATGTTCTCAAGTGATT
TATGCTCCATGATGGATCTGAAGACTGCTAATGTTTCCACTCATTGTCAAAT
CATTTTTTTAGACTTTAATAATGCAATGAGATGATCTTCTCCCCCCCGCTAttca
aatgcaatttttcttttctgacaatTCTTGAAGGGAAAAATTTTTCTACTGGCAATTTATATT
GCAGGCTCAGCTCTAGTCTCAGATATCAGTTGTCTTAAGATGTTGAAATGTtg
gttcttctgttttctccctgtgTAGTGCACAACGCCCTCTTTTCATAACACCACATATGATG
ACTGACAACAAAGTCtcaattaattattttccctttcaaacATTAAAACTCTGAAAATTCCTC
TGGCTTTTGTGCAACACCTTCTGACTGCAGGTGTCCAGGTATTAGACTAAAT
TACCTGCAGCCAAACCTTGAATCTTTGCATTCATCTTTGTGCACTCAAATCA
GCCATCAGTAAAATAcaaattcatttccaaaataGATTTCAGCCTCAATGTAAAATGAG
GAATCTTTAACAAATGCTCATAATAATGGTCTGCTACCACTAAGGTGaaataatac
tttttattttcattgcattcAGTATGTTCTACCTAAACAAATGTCACTATCTCAAATGAGGT
ATAAATAGCCGTAGTCAATATGTCTGTTTGTCTTTATGGTTTCTGtgcattcattctttt
ccattgACATtccttattttgtatttgtttgtttgcatctCTGTGACTATTTTGCAGATTCTCATgggaat
catagaaacatagaatgacttgggttggaagggacatcaaggatcatcaagttccacTCCACCTGCCACTAG
GTCAGGTAGTAGATCCGATTGgacagggccccatccaacctggcttttgacaccaccagggttggggc
atccacggcccctctgggcaacttgtGTATATCATGTACATAATATAATCCTATCCTATAATAT
AATCCTAAATAAGTAGAACACCCATCTAAGGCATATCCTTTCAGAGAGAACT
TTGGTATTTTCTCACCTGGGAATTAGCATAGGCCTTCTGTGCATCCAATATCA
AAATTAGTACTAAGTGTAATGTACAGCAATTGCACTTCGCAAGGTTTCTGTT
CCTCTGGAGTCTTACCAGCAgtctgcattttaaatgcagGGGATTTTGCTCTCTTCTTG
AGTGTTCtggtcagaaagaaaagcaccaaTCCTGGTGCTAGTGTTTGGGCTCTCTTAGA
AGCAGAAGCAATTTCAACTGCTGTAGTAAGCTGATAGCAGTGCTGCCCTTC
TTCCTATGCTGTGGAAAGTGGTGCAAAGCATTCTCGTGCCATCTTTTAGCCC
TTTACAGGTTAcctactttttaaattattcttattattaATGTACTCAAGGACAAAAATGGATAT
CTAAGAAATAGATGAAATCTTACAGAGATGACAGAGAAATTCCATTCTTACA
TGTAAATTTCAGGACTTCTAAGCCGCTGTATTTCCTATGCACACAACTCACT
CTGCTTTAACATGTGCTACTGCTGGTTCCATCTGGCTGTCTCTGAAAGCtcaag
agaaaagcaaacacagataCGAAGGAAATTAAAGGTGCCTCACTGTGATATTTTTATA
GAGTGGTATGTTGCTTTGATAATGAACTGTGAAACATAGCATGCAGATGCTG
ATGAATGTAAATGACTGCATGTGTTCCTCTTTTAATGTTGAGTCCATGAGGT
GGATTATTAGTATAAGCTCTGTATTgtaaaaaacactgaaaagagaagTGAAGTGGAAAG
AAGATACTTCTAGTGATTCGTAGTAAACCACATCTTCTACCTGTCTTTTCATG
ACCAACCTCTGATTCTGGTAATGTAGGCTTTGCAGGTTCGATGGTAATAAAC
AAACTTACAAGAGGAATAGCTCTCTTCCTGAAGTCCAGTATGCCTCCTTCC
AGCACTGTAAGGGGGTACCTGTCCGCATTTCCGTCAGATGAGACCTATTCC
CAGGCTTGGGTTGGCAAGGCTGTATTGGTGGGGAGATCAGTGAGACTCAC
GCTTGGTGCGTGTTTCCTGCCCACTTAGTTCAGTTATCTGTTGGTTGCCACT
CTGATGAATAAGTGACTGTGGTTTGGCGTGTAGCTGCAAGGCTCCTGCCCC

ACCTTCTTGTCACTCCATCTGCACACTTCAGATCTTCCACTTTGTATCTGATC
CCACTTGTGGGCCTGGCTCTGGGGGCAGCCTGCTCGCCAGAGCAAGCCCT
CCTCTGGGTGGAGTGCTCAGGCAAAGTAATGCTTGCAccagtggaaaaataaagctga
ctTTGAAATACCGCACATGTATTTCTGGTAGGAGCTCAGTGGTTTAAACAAG
CTGACCTGTGGTTTGTAGTAATGCTGGCATACAGGAGCACAGGCAGGAAA
GACCCATGGTGTCTCCTGGCTTCTAGGGACTGGGCAGATTTTGTTCTCTCGT
ACAAGACTGATGTCAGCTGGTGATGGCATCATTTCACAAAGAGCAATGGGA
TGTGGGACTGTAGTGTTAGCTGGTGAATTTTGCCCTTGTGTTATGCTAGTAA
TTTGATTTGCATCTTTCCTATCTTGACAAGTTTatagaaaaaaagtgatgataATGTGGT
AGTTCAGTGTCTTGAGATGGTTTTAAAGTGGATAAGCAAATTGTTATGCCTC
TGAGGTTGCGTAATTCCGTGGATTTGTCTTACCTCCAGTGACTGACATGTAA
TATTAATCATTTGAGATCTATGTAAGCTTTACAGGCTGGAAGTTGTGTCAAA
ATGACATGAGCACGTGGGTAAACATCCTGAATCACCATCATTCTGGCATGCT
CGGGGGCAGAGGTACTTGTGTAGAGTGTTTTAAACATCACAAAGTGTGTTG
TTGTTTAGAGTATTGTGGTTAGGGCTTCCGTCTAATTGTCCCCAATGTAGCA
AATGTTCGCTGATGGCTAACACTGTTACAAGGACTGAAGGGTTGAGTTTCT
TTGCCTGGcaataatgtattttctattatGTCAAGAGTCTGTTCATTTTCAAGTAGTGCTC
TGTGCTCCTTTGaagaatcttattttttcaagtggtTTCATCGTGAATGTACCCCAAAATGT
CTAGATGTCTTTCAATAGCAAAGATTGCTTTGTTATTGAgtgtattatttattaatagtAC
ATTGTTGTCATTTTCTAGTCATATTTTACAGAATATGCCATTAGCATCTCATGT
GATTTTTTATAAGGAAAGTCTTATTTATAATATTCATGCAAGTGGACAGAAA
CAAATCTCAATTTTATAGTAGTCCATGCCTTTTTTTCATAGCTGAAGCATTTT
AATGAACACGACTGGAAGGGACAGgctcactgttttcattttgatggaACTTTACATAGGT
>TCONS_00279708
CTCATGTTGTTACAGAGTTGAGACAGAGTTTTGGGAAGATTTTCTGATTCCT
GCCTGACATGGGTTCTCTACGagcaaacattcagcaagtacAAAGAGCAATTTTGCAT
GTTGCTGAACATGCAAAAAATGACAGTTGTGACATACGGTTACCTAAGGAG
CCCACTCTTCTCTCCTATGTCTCTACAGTTCTGGATTGATGGATCTGTCCCCT
CCTTGGGCACACAGTCTAATAGGAATCAGACATTGTCACCATTTAGTGTGTT
CTGCAAAGGTATGGAATCAGTAAGAGCATATCTCACCTGCCCTGTGCATCTG
AGCATGCTGAATACAGTTACCTAAGATTTCAGTGTTTGTGGCAGAAAAATAT
CTCTTTTGGAACCATGCATGTAGGCACAAGATTCAGTGAAATTGCACCATG
TGGAAGAAAGTGAACAGAGGGATTAAAAAACATAATGTTACTTCTCCACCA
AAGAGCAGCAGATGGCAGGGACTAGCAGGTCAAATTAGGTTTCCTGTtggtgg
agaaaaaaatcaatgattCAGAGTCTTAGTATATTCTTATTTCAGTTATGTTCTCTGATT
CTCAATGTGAGTTACTTACACGCAACTTGTCATCAGTCTCTGCTTTCAAGAG
TCTCAGTAATCACAATGAAGAATCTCAGGACCAAGAATGAGGTTAGAGAC
ACTAAGCATGACTACCTGAACAGCCTAAATTGTtatcaaaatgattttattttatctctACAT
GTAAAAATCTCaaagctttcttaaaaaattGACTTCTGTTGCAGAGTTTTGGCTTCGAA
GGAATATTGGGTTGAGCTCCAGATCAGAGTGCTGTCATGCATCTTTCTGCTT
TATGAATGCAGTAGTTAGCTAAGTACTCAATACCCTAGACATATGAAAGTCA
GAGGCAGAAATCAAACCCCTCTGgaaaatttttttccttctactctTCATGTCCTTTTGTTC
AGCTATTTAAGTGAAAAGCCTATTACATgtcaaacaaaataattacatccaaattattttccagaCT
GGTATTGtttctaaatgtttttgtttgtcttctcCACTCACTGTGGAATTTTGCAGTTGTTGTCT

TCACTTGTCTCTTCAAAGCTGATCTCATAATGgagattaaaaataagaaagaacaaTTTCC
AATTTCTCCATGGACAAAGTGACCCTTTGATGGGAGGAGTgttgcatgctgctgctgat
gaAAGGAGGGTGCTGCAGAACGCAACCATGTTGTGTGAGAGGGAGCCAAC
ATTTGGTGATGAAATTGATGATTGGCTGGAAGTAATAGGTATAAAATCAAGG
TATAAAACTCAAGTGGTTAGGAGAAAGCTAAGAATATGATTACGAGTTTGA
GCATTTGAAAAATAGATTGGAGTTAATAGTGTAGAATAGTTTCATACACATaat
atgaaataatgaaaagctTTCCTAGCAGTCACAAATAGGGGATAGACTTTGCTCTACA
AATGTGAAGCATAGGGCTGAGGTAGTTTGTGAAAGCACCTACATTCAGGTG
TCTCCTTGTGTGTGAGATGTCAAGGTTTCCATTAGAGTTAATAGAGATCTAC
TCAATACAGTCCAGGGAACCTCAAGGGCAATTCTCAGCTGACCAGTCAGTT
TTTGTGTTAGAGTGAGCTGACAGCATGGACTAAATCTCACTTAACTAAAAT
GAATCTTATTCTGGATATCCAGTCCAAGCTGTCACCTGAGCTGTCTTTGCTG
TCATCAGGGAGCGATCAGTCTTGCAGAAGGACTCTTACATTTCTAAAATAG
GCTGTTGATCCTTATTTTAGGCTATCAATTCCTATTTTAGGCTATCTGTTTCAG
ATCAAAGGCCTGGTCTTGTTGCTCACTCACAGTATTCACATTCTTGGGATAT
CTACAGGTTCTTTTCCGATCTCCTGCCACTATACAAGAAGGAAATGGGCTG
GGGCATGGGCCTTCTGTTGCTACTTTGTCATGTCTGCCAGATCCAAGTATTT
CTAATAGGACTAAATATCTATGTGTAGACAACTGAACTAAGCATGAAGGTCT
ATTTATAAACTATTAATTATTGTGACTCTCATCCTGATTCCTCACTTAATGAGC
ATTGGAGTTACATAGCTCTGAAGGCTGTAATAAACAGACTGACTTTATTGTA
GCTCTCTGTGTATCAACAAAGTAAAAAAGCCATATAACAACTAGAGAGATC
ATTCATCCAGAAGATGGTTTATAATGTGGTATGGTAATAGACGCAAACTGGC
ATAAATGAACATGGTTTCTGATCCCAGCTTTATAGTAGAGTTGTGTAAATGT
AGTCATTAGAGTAAAAGCAACATTGCTATTTGCGAAAGTGTTTGAAGAAAA
GACTTGATATAGTTTTCAAAGATTGCAGTATATTCACTATGAAAAAgaacttgcttt
gtttttcctttttttaaaattttttattttttactagcTGATGCTGTTATAAAAAATAGATAGCTGCAGAA
GTGTTAGAGGAAAATGCTGTAGTGTGCTTAGACTGCATTTTGTGCAGATGG
CATGCCTGAGTATATAAAAATGACCCCTTGGGAATCTGTGTGCATCTTTGTT
TTAGAATACATCCTGGTTTTATTAATGAAACAAGtgaaaaggttttgtttctgtgtgtgagGA
AAagatattacaaaaaaaaaaaggaagcagggGAAGAACTTTCCTCTATTTTAATTAGAAAGT
AATTGCATTGCACCATTAAAACAATGAGCTCTGTCATGAAATTCAAAGAAC
TTGGGATCAGTATTGGGCATAGATGTTGTACTGACAATTGTAGGTTATTTCTT
TTAACATCAGTTGAGTGGAGAGAAAAAGTGctattttaattgaaagaaCAAATATCAC
AACCTTAATATTGTTGAAGTAGCAAGTCCTGACACTGGCAATGGGTTATGTT
TGTAATGACATTCTTCAGACATAGGGGCATTCAGAGCAGAAGCTTTtgtcaaata
aacaaaaaaccacaacacttacatcacacagaaaatacaaatttccTTTATAAGCTAGTTAGCTTTAGG
CTGAGGAAGGTTTAAGAACGTTAGTCCGTCTGCTATTCCACATTGGTATTTT
ATCATATTATTGTGTCATCAGCATAACAAATGTATTTGGCAGAGATATGTTAC
TACTTTTCCTCACCAGatatctcagaaaataaaaataaaggtataTGGGGAACTTCTACATGA
CAGTTTCAAATCAGTCTTTAGCTACTACAGGTCTAAAAAGAAGCCAGATGA
CAGAGAGAGATAAGCAAATTAAATTACATACTATTGGATACTCAGTGTATTA
CACATTAGTGGATAATGACTGTGACTAACAGCTATTTTGAAACCATGAACTT
ACCGCAAACTCactccatttttgttttgttctatgAATAGAtcataaaatatctgaaaacacAGTGCACA
AAATCTAGATTTAGAAATGACTATGCACTTTCTTATAATTCACAGGAGGtaacat

aattaaaaatcatCACTTCTGTTAATGACAAATAAAACAGGCTATATGTAAGTATTTA
CTATTAGCAGAAACTGATTTtagacatctttttttcttaagaatcGGATGTTACAGAACAAG
ACCCAATctaaagaggaaaggagaaatttggacttttttttaagttaaacaGTGAGTTATATGTCATTCA
AAGATAACACTTGTATGGTAATTGAGGTAGGGATTATTAGACAACAATCCAA
GGAGATGCACAAAAGTTGACACACCTAGCGTTAATTGTATggcatgtttgttttctgtatt
aaaagaaGTCAATGTGCACTCCTCCAGAatgtattcagaaaaaaatattttaaaagacaaaggGGAA
TAGAAATCTAACATGCCTCATTATTTCATATTCtatctttctgtctctttgtcCTAGTCTCTT
CAATCAGACCATCACAACATTTTCATAACTTTGTCATTATTAGACACAAGAG
GAATAtcaaagatgaaagaaaggatAGAAACAGGATAACTGCAGAGGAAGCATGTCAT
CTGGCCGTGTCAGTAGTTGCTAATTAACTTATCCTGTATCTTATCACCATACA
GTATGTGTCATACATGCAGCCCAGTGATGAGCTGTTCATCAGATATTCTCTT
GAAATCAACTATGGCATCTTAAGCCATAGAATCAACTAGCttgagaagaaaacactgc
GAATAATTAGTTCCTTCCTTTATTGCAATTACTCCAATGCATGTagataaatgaaaattt
tatgTCATCTCCTACTTATCTTGCTTGTTCTCTCTGTTTCACTTAATTTTGCCCA
TCTTTACAATGTATCAGTAGGTTGAATATGGAGCTTTTGAACTGACTAGGCA
TCGCTTTATGGAGTTTCTTTTAATGTTGAGAATGTatgtacttttgttttgtctgaagAGAT
TGAGGAAAtagatttaaaatgtattagttTATAAATTGTGAATTTCTTATGAAGAAAGTGA
GAGAGTGTACTGTCAATGGAATTGAACAATCAGTTTCTTACTGACACAACT
GAAACAGCATgaacttcttaaaaatacagatcCACCAAATCCTTTTTATGAAGATCCTACT
AGAAAAATAACGGGCTATTAAAATTACTCTGAACTGGATATCTGCCTTGAAT
GGATTACGTGAATAATAATCTCTTAAAACATGACTGTGTTATTCTCCATGTGA
TTGTGCCTGAGAAGGGAGAAGGTACTGCTGGCTCCATGTAAGTTTGTGTCT
TTCCACCTAGCATCTCATTGCATGTGATTAGAACTGACCAAAACATTCCTGG
CCATGTTTCTGTTGGGCAATGCTGGTTCTATAGAATGAATTCTGGTAAACCT
TACCCCAAGCTAGGGACATATTCTGAGCCAACATCATATGACTGAGAGGTTA
GGAATGAACTGCCAGCAGACAAAAAGTCTTAGTATAagcaacatttctgctttttttcatgc
ACAGACTTGTGACGTTCTGGCATCCAAGCTTCTCAAGTCAGACTCATTTCT
CATGGTTGATCGAAGGTGGAATTTGGGGAACTTGAGGATTATCTGAGCCTG
GGGAGGAATTCTGCTTGGGAAACTGCAACCTGAGACAAGAGGTGCTATGA
AATGGTGTGAGCCACACGTTGGAAATGAGGTGCAAGTCAGACTGACAGAC
TTTGTAAAAGAGACCGCGTCAATATAATTAACACTTTCTTGCACTATGCCCT
TTGGCTGAATGAGGtgataaaaataacagcacagtTTTGTACAGCAGTTAGTACAGAAG
CGCATCTGCAGCAAATGCACATTTGATTCAAAGTtacacagacttttttttttctttttatttgtgg
TTGTTTCTTTCAGGAATTTTATTCTTATCCCAAAGATCTGgaaaagtatctttttttcttttttt
tctttctttctttctttctttttttttttttttcttaagaactGAAAACTGCAGTTCTCATATAATTACATAGT
GGAGAATCCaagacataaagaaaaatacaaggaaTAGTGAAACACAGAGAACTTGGGAA
TTTGATGACAGTGTAGACATTGTAAAAGGGCAAGAAGAGAACAGACACCA
GCTCCTCTGTGTAAATTCTCTCTAAATTTTCTTTATGCTTTATGAAGTCTCGT
TTcctttatatatgtatatatttttctcccctACTATTCCAAAAATTGTTTCATTGGCCTGAAATA
GTCAGGTTATGTTCTAAACCCtaatagaaaaagaatttgaaactAATGatactgaatttatttgtttgtcC
TGCAGTTCAGTAGCATAAGTGCTTTCTATCCCTTGGACAAACTTGCCTATAG
CATCTGATTTCTGCTGGAAAATACCATTACTGTGATATGCCTTTGCTTTTGAG
TGCCATGAAGATGTATTTCTCACATGCCTGCTGTATGAGTGATTGGATTGGC
AGCTATAATCTTTATGAACTTTGTAGTGCTTACATAATGATCAACATTTGTGG

TGAAAAGCACTTGGAATATATTTTGGTAGTTACAAGAATACTTCAAATGATG
AATTGCAAggctttgattaaaaaaacaaaatgtaagagcccctgattttcattttccatatgGTCCCTCTACAT
CACATTAGCAGTGAGCAGTTACTTTCCAGCCTTTGCTCTAGTTCCAAGGCA
CTGGTAAGGGGCATCAACATAAATCTGCTTCAGATCATCTTGGAACTCTTAT
TTGTGCAGAA
>TCONS_00318608
CACAGAAGGATCTGCAGTATAAGCAAAACTTTCTTGGAGACAGATGTCAAC
TGCCTCGAGTCTCTCCCCAAGCAGCTGAGCACACAATGGTTTCATCCAGGT
TACTGGGTTTGTCAGGTTGATTTTGAGCTCTTGCTTCAGGTAGGTCTTCAGT
GAACTTCTCACCTTTGTTTTAGGCCTCTTCAGCTGATTTGGCTTTTATAGCA
GTACAAAAGATTGGGTGGTGCTAGCCATCCACTTaggtacaaaaaaaccccaagttATA
CccctttattttcattattaactAAACAAGTATTTTTGGTTTTCCTAGTCTGGTTACGTATGT
ATTTCTCTCTATTTCCCTTTGTCAGAATAAGCGTCATAACTGCAGTCTGTTTC
TTCTAGATCTGAACAAGACATTGGAACAAGTAAAGCATGACTATCACTTCA
GCTTCTGTTTATCTCCTCCTCATTGGACCTTCCTGTGGACTTACCAGAACTC
TATCAAACAGTATCACAATATCTACCTTTGCATCCGGTAAAAGTGGGGGTCA
CATTAAGACTGGCTGAAATTCCAGCTTATGAAATATACTGAGGTTTCAGAGC
CTGTTCCAACATGAACAAAACCAATATCATCTGTATTACATGCATGAAGAAA
CCAAACCTATTCCAGGAGACCAATAGGAATTTTTTCTGTAACCTACAGATTA
GTAGCTCACCTTGGGAAAAAAGAGAGCCATACTCAGCTCCCCTCAGAGAA
TCAGGCATTTGAATTTGAGCTTAGCTCCTTCCAGAATATTCAAACAGATGTG
TCATTTAACCTCCCTCACAAGACTGCACTTCCCAGGATGGAGGATGGTCCT
GCTGTATCTTGAACATCCCCCTGGCTGCAGTGCATAGCAAGAACATAGCTC
AGCTGGTAAAGCCAGCTGTGAGGAGGAGAAATAGGGGATATTCCTTCCTAA
TCCACTGTCTTAACTTATTTTAACTAAagtgttctggttttgttcaaCAGTCCACAAGTTG
CTCAATTTGGACATATTTTatcagggaaaaacaaaaaagtttgctttttgcttATA
>TCONS_00335497
TTTAGATGTTCATCTAATCCAGCTCCAATGCTTTGAATGAACAATTTGGTTG
GAACCGAAGTGATACTGGTGCTTGGATAAATGCCAGCTTGCTGATCCTCTG
TCTGAtgaatactgaagaaaacaactggAAGACTTGGCAGAAATTACATCAGCATCCCT
GATTGAGAGTGTGCACCTGCCACTTGCTGTTCAGCTGGCCATAAGATCCAG
ACATTCCTATCCCTCTGAAGAGGATCTGAATCTAGGAACTATGGCAGAAGT
GAGAAAGGATCCTGGGGAATAACTAGCATACATGAAGATGCTCAAGTATTA
AGGACGCTAGTGATGTGAAAGCCCTGACTTGTTCCTGGTCTGGTAGTGATC
TGGTGCACTTGTGCTAGCAGAAGTATCAGAACAGTGTAGAAGAATTGTCCT
TCCTCTTGACAACTTGCCATCCCATACCCAAGTACTTAGAGTGCTAGCTTAA
TGGGAGATCCACATTCTCTGCTTGCCTTGGAGAACGACTCCAACTCTTGCC
TCAGAGAAGATAAGGTTACAATAGCACTATATGAATCTTTTAAGAAGCCCTT
GCCCTTGCGGTGTGAAGAGAGTATGGAAGAGTactgctttgggaaaaaaataatagcaatgt
TTCAGTCAACCTAGGCAGGTGAAGAAGATAGAGTAGCAAGGGTCAAGGTT
TTGTTTAATCTTTGTTAGAGAAGGATTTGAACCCAAGTCTCTCACAACTGTT
GGCTGTTGAATGATGGAGAAGCCAATTGCGTCCACTTCTGCATCTCCTGAG
AGAGGATAGATGTGGTGGCAGCTCTGAGCCAATGAGCCACCACCCAGTTC
GTAGGTAGTGAGCACCTCTCTTTTAGATGCTTCCCATCACTTGAGGAGCCTC

CACACTTATTTTCCTTGAACCTCATTGAGAAGTACCTCATTCTTCTATATGAA
GTACCAGGATACTGTATATTACAAAGGACTGTGAGTTCTAATATATGCCAGC
CCACATGATTTCAGTACCTGAGTCTCTGAATGGATTAGCTCCTGACTGCTGT
ATGCTTCAGTTCACCTTTGATACGGGCATTAACATTGAAGTCTGAACTCTCA
GTGGTGATGTAGAAGACAAACGCATGAAAGATTGTCCAGTACTGAGACAC
TGTTGTGAGGTGGCAATAGAAG
>TCONS_00336182
GAGCCGAGCTGCTGGTGACACCCCCTGTCAGCATTGGGGAATTTTCCTAGC
ACAGGCAAGATTCATATGAAAGCTTTAACTCTACAAGTCCTGAAAATCCAC
ATACAAATGTattccagaatattttttaaccaatGCGTCCAAGATGCTCCGTTTTAACAAGT
CAGGTTCGATGCTACAGTACCCTCCCAGGCCAAGAGAAGATACTTCATTTAT
CAGAAGTCCTGCTATTTTTCAGCTGATAAGCAGCTCTCGTGCAGTATTCAGA
TCATACTCCAAGAAACACTCAGATAACCACCATCAGGTGCCATGTACAACA
TTCTACATCTCCATCTCCCAACCCTTAAAAGAAGTACTAAAATCAAGTGGA
AATGATTCAAAATtgactgctgtgtttttcattgTGCTGAAGTCTAAGGAAAGGGGGACA
GGGGGAGTTCAGGCTCTCcagagaaataattatttcctaatGTTTGCAGTAAGAGGTCAC
AGCTTCTACTAAATCATCCCATGTGCTGCTAAAGTACACACTCTTTTCTCAC
CAATCTTGCTTTTCCTACCGCCTAGAGCACCAAAGCTACAACATAACCAGT
AGAAACCAACATGCAATTTATAAACAAATCTCATTTTACTGGAACCTCTAAG
CTTTTGAGAATTTATTATTCCAGATTTAGCCTAGTATTAAACAAGAGTGAAAT
CCAGGCTTTATAGAAAGAATTAAACTTGCTAACGCGCCTTAATGAAGGAGA
ATTAGCATGCAATCAACCCCAGCCTCATTTACCCCAAAACAGGAGCTGTTT
TCCCAGGAGGGATATGGAGTGCTTGTGACTGCTGTCGTGTAGGAGCTCACG
TGGTGCCTTCAGCCTACACTTCAGGTTTTGGAAAATACTTCAATGTCATCAA
CAGTCCACTCTCAGTCACATAAACTTCTGACTATTTCCCCCACTTTCTTTTAA
TGGGTAAACAACTCCTAACTTCTTAGTATTGCTCGCTTGGCTAATTGTGCAT
AAAGTTACATAGAAACCTCTCTCACTTACTAACAACTAACATCTCTTTCTCC
TTcgtgtttgcctttttttgcttATTCCTCACAAACACACAACAGCTGACCTGTTGGCCAT
TACTCCAAGAAAACTGGCAGTCAGCAACTTGTTCCTTCTGCCATCTAGTTC
CTTACCCTGACTTCAGTTTATATACACAACCGGAGCTACGATCTTGCAAACA
TTTTGCACACGTCGCTCTATGCTGTGGCGAGCTTAAAATCACCCCTATCTCA
TCAGATAATCTACAGGGTTCTCACCTGTGGTGCTGCCGTATGACTGGTGATT
ATGGTTCTGAGAGAGAGGAAGTGAAGAAAATACCACGTTACTCTGAGGTT
TGAAATGttaagagaaactgaaatggagATAGCACTACATCTCCGGGCAGCTCACAAGT
ACGAAGAAAAGGCTTGGGATTCGGAATAAAtcggaaaaaaataaaattataaacaaCTGA
GAAACAAATGTGGAAAATGGGTTTTCTGACAGATTTTGTTATTCCCACGC
>TCONS_00372534
GGAGCGGCGGTAGGGGACGCGCGGGGACCCTCCGGCAGCCGGCGAGGGG
CGAGGCGGCCTTGTGCCGCGTGGCCGTTGGGCGGCAAGATCTGGGCGCCT
GCTCCGAAGGGGCGTTGGGGACGGCCGCCAAGCGCTGAGGGGACAGCGG
GGACGCGCTTCTAAGAGTTCTGAGGTGTGTTTGGTAGGAGGTCCTCACGG
GACGGCATCGAGCTCCGAGGCCTACTGGACACGAAACTGGTGTACCAAGA
GTTGTAAGTTCTTAAAACTTGTATCAAACTGATGAAAACAAAGACTCCGAG
CAAACAATCGGAGGACTCATGTGAGAGCATCGCCCTTTCCAGAGCGTAAA

GCGCATCTGGTTCCGTTGTATCGCTCTTACAACAACGCGTGAAGAAGGCAT
CTGTGGAACTCCCTGTGGGATCTGACTGCCCACGCCTGGCAGGAGCTGCC
GTGCCCATGGCCTGGTTGCCCTCCCTCAGcccccacctgcagccctgccctcGGAGCAC
CGGGAGGAGCAGCGGGAGCGTTTGTCTGCTTCTGTGTCTTGAGGACATTTG
TTCCAGAAAattcCTGTGGAAGTGACGTTATGTGGAAGGCAAATGCTtgacatttcc
attttgttttctcaacCGGCTGAGGGAATGCCTTTCAGTCCCAGCTTCCTGGCTGAGA
CAATGAGCTGCACGGGGCTCCCGTGCTCACATCCCCTCCCAGCGATAACAT
CCTCTCAGCTGTAGCTCTTGCGTGTGGACAGAGCAAAGGCTGCCACACAA
GGCTGGGCAAATCCATCCTGGCAAAAGAGGTCCCCGAGTGAAAATTTgtattta
agggaaaaaaaaaaaaacctacatcACATCCTTAAGTTTCATTTTGGATGTTCAGGTGTTG
TTTTCCTGTAGACGTCCTGGGGCTTGTATGAGGAgggtgtgcatgtgtgtgtatgcaagT
ATCTTTTTTCCGTTGCTTACTTGGGGACAGGAGGTTGGACGGTGATTTGTG
GGCACTCACTGTAACTTTTTAGGAGCTGCTCTTGGGTCCGTTCCAATCTGA
CAGCAGCTGAGTGGCACCTCGGGAGTACGTAGGGCTGTGGAGATGCTGAC
ATGCAATCCTCAAGCACAAAGTGAGAACATGAGCTTCAGATGGAGGATAAT
CCGGGCTCAGAACAAAGCCGTGTTTATATAACGTGCTGGAAACAAAGGTG
GCTCAAAGAAGTTGCATTAGCTTACGAGGAGCGTTTGTATTGTGCTGCAGT
TTTTCATGCTTCGGGTATTGCACTGTAAAACATGTCAGTGTGTTAGGGCCAA
CCTTAATTTATTGTAGCTCTTCTTCTTCCTTGGCCTTCCCACCCAAGAGCAG
TGCCGTTAGCATTGACACCATGGCTGGGAACAAGTGCAGCCAGGAGCATG
CtttttgtgctgctctgtgctcagcaggtTGCAGTCTAGGAACTGTACGGTGCAGCTGTTAAG
CACTCACTTCACCTTGGCAGAGAACCTCATCGTGCCAGCCCAGGATGCAGT
GGTGTgacacagggagctgtgggAAACACACCCGTAGGTTCACTTTTACTGGTTCGT
TGGGAGAAGTGTTGTCGTCCAGCCTAAGCTCACGCTTAGTGCTGAAAAAAT
CCACTAATTGTAAACCatcctgtgttttctgaagcagttcCCACACGTGACAGATGCATTTT
CTGTGtcaagatatattttttctccacagaagCTGTTTCCTGATGCGATTTCTCTTTGTTAGT
GGttcattcagtttttttctcaccCCAACCTGTCCTCCTAAGACCCGATCTAATTCTTgtcctt
gtgtgtgtgtgtgcataccGTGAGAAAGGCTCTGTTCTGCGAAGtgtataaaaatacagcaagtgca
atttctgttttgtattcagGGCGTGAGAGTTTATTAGAAGGCCCTGTAAAATGATCAGCG
GGgaacaaacaaaactgagaaatgaaGAAGTAGCAGTGGGAGTGCAGGATGTGCAAGA
GCAGCAGGGCTTAGACAGCGATTGCTCAATGGCAGATGTTGTGACTGCAGC
ACGTCAGCCCGGGCAGGACCTGCTGAAGTGCCCCCATCCCAGGAGAACAG
CTGAGGCTCACCACCTACTTTGCTCCTAAAGCACTGTGGCGGATGTTTACA
GCACATCCTCGCTGGCCTGGTAACCTGCAccttgaaggaaaaacattGCAATGTCAAA
CAGCAGCATCGCTCTGTGCCTGAAAGGACAGAAGGAATGTGTGGGATTGG
GGTGGGCGCTGGAGCTTTGTGGGAGGAGCTCTCAGAGGGCACTCagcctgctgg
gtgctgggtgcAGGACAGCCAGGGTTAAGCTGCACTTTGGCAGTGGCTGCTCTTC
TGTGGCTTGGTTaacaagctctgctgctgtgctgggcatcTTCTAGAACACAGGGGACCGGT
AagcatgctgcagctgagctgagcttgGCAGGCTGGGAATCAGTCGGTGCTGGGCCACAG
AGCTTTAGTGGTTTAGTCCGAGATTTGGGAACAGCCTATGCTTGTGTCACAT
TACATGTAATGAATTAACAGGTTTAATCTATTAAGTCGTAGTTGGCACTGGG
AGCACAACTGACAAATGCTTACGCCGTTATTTCTGTCATCTGGTTGTACTTA
ACTTTTGATCATGGTGCGTTTGCCTTAAGGGAGGTAGAAACTCAAGTCCTC
CAATCGTATGtaaagtgctgtgctgtgctctaaGAGCGGTGTTTCCGTGCTGGAATTGCTA

ATTGGTCTGTTCCCACCTTACATCTGTACTTAAACTGCTGGGGGACAACTGC
AGTGACACGCTTGTGAGAAGCGGTGAAAGCGCAGAGTGAAGAAACACAC
AGCCCCTTGTGTTACTTGCACGTGCTGACACATCACTTGCAGATCCCCACG
CTGTGTACTCACATACTTCAGACGCAATGGAGATGTTTTCGTTGCACAGAG
GTGTTTAtttctttggtttctgtttGCCAGCACAAAGCTCTCCCCGATGCAATATCCACT
GAGCAATGCTGGAATGGTCGTGGAGAATGATAGCAAAGTCACTTACAGAA
CAGCTGTGGTGTTGCATTGgtcagctcagtgctgtcccCGGTCACCGAGTATGGAGAA
AAGCTCTGAGTTTCTACATGAAGTTCTCTTCAGGCATCACTGCCGCAGCATT
CAGTAAGCCACATTATGAAAACCAGTGCAGAACTCCTGGGTTTATTACAGG
CATGCAATTAAACAACATTTTGGTGAAGAACAGAGCTCTGTAGACCCAAAG
GCACTGAAGCGGTGGTTTTCTGCGAGCGTGGGACCTGATGTACTGCCTGCA
GTCATGCTGTCTTGGTATCTGGCACCAGGCAAGTCTGGCTTCCGTCACCAA
GCTGTTGTGCCCCGCTGATGACCTGCGCACCGTTTCTTGGTGTTAATGAAC
AGGACTCGGGGCTTTCCCCGGTGCCCCCAagtttttctccctgtttctaACTGTCTAACT
TTGACAAACCTTAACTGTATTTAGGCTGAATCTCTTCTTGTTGGGCTCAAGG
TTTAATTCTCTCTGGGGAACTTAATTCAGAGGAGCGCATCTTCTTTGAAACC
AGGGTTGGGTTGTGGTGGTGTTGCACCCACATCCAACTGCTCTCTGAGATG
GCTCTGTGAGGTCATTTTTGTCACTGTGGTGCTTTGGGACAAGAACTCCAG
TTTGGGCAGGGGGGAATGACGTCCATGTAAGGGATGCACGTGTTTGGTttttac
tgctgctgctctcagttggccaaatgtttgcaaaaaaaCCTGTTAGCATGTGCTAGTAAAAGTGGC
TTTTGTATGTTTATGTGTCTTTATTTTACACCAGAAAGCATCTGCGTTGGTCT
TACCTTCAGCGAATGCTCTCAAACCTCTGTGTTCCCGTGAGAAAGGCACAC
GGATGATGGCAGTACCACGCTCTGTGTCTCAATTACCCACGCTCAGGGTGA
ATTAATTTAGACTTCTAGTAGATACAGAGAAATATCAAGTGCGGTAAAAGCC
CTCTGCCTTGGAGAAGATAGAGCTGTTCACTCCACTTAGCCTGAAAGGAAA
GGTGGACGGAGgacagcagggcacagcagaaTGAATAGCGGTGATGGGGAGAGGTA
ATCATGCCATGGAGAGCTTTGGCACGAGAATGGCAATTTGTACGTGAGCAG
TGAATCACTTTAGCCTGGAAGCTGGAAGAATGGTGCAGTTCTGGAGCAGG
CTTCCACATGGAGCAGTTTGTTCTGGAGTTcacatcatttaaaaatgctggGCATTTTGT
CTTTACCGGGATTTGAGTATGGTGTACTTTTTATTTCTCCCCGTgggtgcagagcac
agcctgccaCCACTTCCTTCTGGAAACTTTGTCACCTTGCCTTAGCAGAAGCAC
ATAATTATATGATTGTTTATtacacaacagcaacaacaaccaCAAAAAGAACTCCATGTT
AATCAGCTTTCAAGCTTATTAGCTTCCCGTTCAGGCTTCAGTCGTACCATTC
TAGCTCTGACAGAGCACTCTGGGGTTCATTCATCCTTTTGCATTGAGCAACT
TCCATGCTCCGTGTGACTCAAGCCACAGAACTCCTCCAAGTGCTGCCTTAC
TGCTACCATATCAGATAACCAGATAAATGTTTGTTTGTACAACATCAAATTCT
GGCATGCAGAGAGGTGTTCTCACCTACCTGTCGTGCCTTGAGTTGCTTGTT
TCTAATAGAGTGACTGCAGTTATACTCCTGAGGAGTTTAGtatgaatttcattttccacC
ATGAATTGTAAATTGGAGCCACAGCTTGCAGTGATTCTCAGTAAGTCCTTAC
CAGAATATTTGTGTAGGTTGGCTCTTCTGAGAACTTACAATCAAAGCTGCTA
CATTCACACTGGACGCATCAAGCTGATATTACTCAAACTTTGTTCCTGGATT
AAAAAGGAGTTAACCTTTATCTTCTGA
>TCONS_00552241
aaacaaacaaaaagtttagCAAGCTTTTCCTACATCTTCCACTAAGTTCAGTAAataattca

gagaaaataagttCAAAGAATATCCTAATTCTTCACTGAATTCTTCTGACAGTCTGT
TAACAAACAGGTTTCCACCCCATTGTTGAAGTAACTACACAGGAAAATGCC
CTTATGTAGACATCCCAGCACGAAACACAACTGTAATTCTCCGTAACTCTGA
TCCAGTGAGATAGTGCACTTCCTCATTTCCAGATGAAGTCAGGACTGTCAC
AGATCTAGAATCAATCTGACAGACAGAAAGCTCAGACATGGAGCTTTGTGc
atgaaaacagcaggaaagcagagtaTGCAGAGCTAGAAAACACATATGACATCCTCATCT
AGgagaaaatgtcttcttttcccGAAATTCCTTTCTGCAAAATGGTGATTTGTGTATAAAA
TCTTCATGAAAGTTGTGATCCAACTGACTTGGGTTTTGACATATAGAACTTC
CAGGTAAATTTTTGTATTACAAaagttttattaattttgaGGATGTGCAAGCCGTGCAAA
ATTTAGTCTCAgttctttgcatttgtttacTAACAGATAAAAGAACTATACTTTTTCACCA
GtgtacatttatataaaaaatatataaacaaatataaataaatttaggtttgttttctgttagttTATCTATAGTTTATG
TCATTACTGTACGGAGAGTTTATCAGCATCAGTAtaaatcttttccaacctatCAACTTG
TTTCAGTTAAAATATAGTGGAAGGTTTTTCTAAAATAGTAAACACTTAAGGT
TTCCTGCAAATTGGTAGCAGGCAAGAAGTAGGgagatttctttatttcctctgatGAGGGA
GATGTAGGGATGACTCAGACATTATTGTGAAGGACTGAACATACATAGCTG
TCTAGCTCATCTTACAGCTAGTGCTTCTGCGTGGCTTCGGTATGTTCTCTCTT
GGCCAGTGTACAGATCAAAGGAGTAGATAGAGATATGAGGAGAAAATGGG
GATTAGTGCTTGAGGGCATGGCCTAAAGATCTAGATGGAAAAGAGAATGCA
ACTGGAATATTAGtggaaaatacatcagaaatttacagaaaacaagtaaTAGTTTAATTGCTCATG
AAAAGAGTGCTCGAGTTTGCcctttcttatttcatttttttaggCTAACCAGTCTGTAATAGT
TTTTCTTGCTCTTGGCTACGTGTCACATGAACAGCTGGTATATATTGCTTACA
AATAACAGCTCTgaatgagagagagaatgatTGTAAATTTGATGCATACTCCTGTCTCA
TCTCTTCCATTGATTCAGATATGAGCaatgtaaaatgatttttttttttaattgcgTTTGTACAAT
GGACATTATTAAGAAAATCTCTACAGAACAACGTTATGAACCTGTGTAGAA
ATATTATTACTTCTGCATTGTTGGGAATCCAAAACATCTGGATTAAATGTGAC
TGAATGCTGATGGAGTTGTTGCTGAAATTTCAGGATCCTTGTTAATCACAAT
AACGTTGGTACATTCAGAATTTTACTTCTCTTGGTAATAAACTGAGTATATAT
GGTCTCCTGGTCCCTTTTTAACTCCTTTCCGTCTTCTCTTATATTAAACTCAT
CTCTGTATCTTGATGTCATCACAGCACATAAGCTTAAACAAACTGATTATATT
GCTTTCTTGCAATAACTTTTCCAACATCAGAACTGTGTTTATATTATACCTGG
AACTACTCAAAAAGCTCTAGGTACCTTAATCTCATTAGTAATTAGTTTCACA
GGCTACATCTTTGTAAGTCCATCTTGCTTTCTTCCACTACCTTAAGAGTAGC
ATCCTAAGAAGGATGCATACCTccagaaagctgcatttttttactctctctCCCACATGCATGC
TAATGGATTTCTactacatttcatttaaaagtctACATTTCAACATATATGAATCCACTCTCA
TAAAtgattcatatttttaaataattctgtattttgattTCAAAATTCTTTGCCTGTGTGTCTATAGC
AAAGGATGAATGTGAAATTGTTAATTCTAACATACGTTGAGGaaggtgaaataaaaa
aaatgccctTTTTTGATATACAGCCTAGTGGAAAATCAGTCACTGTTATTAAACTA
TGACTGACTTTACATCATGAATATGTGAAAAAGTATttaaacttgaagaaaaaaaataacat
ggaGGGTagaaagtaaagaagaaaatcctgcTGTATTGGAAGAGGGATTAGTGTGGTCCTA
AAATGTCTCAGTAACCTAGACATTTAGATCTCAGAAGGCCTATGAGAGGATA
TTTTTTTTGAGGCTTAAAATGTAGAGTTGGCAAATGTAAACCTATTCCTAAA
CaccaaataaaaacatcaaCAAGGATTAGAACATGATAAATTTCTCAAGATAAAAGCA
GTAGAGAGACAGCTGGTCAacagctgtgaaaacagaattaaaacttGCATAAATTGTAAAC
GATATTCCCCAAACCAAACTTGAATCCAAATAATACAATATGTATAATCTCAG

CACAGGATTCAAATTGGGTCAACAGGACACTGTGACGGATTCTGTGGATGA
CTGCCAGCATACCCAGGCAATTGCAGCCCATTGACCAATGTCAAACAGTTC
AAGTTTGCTTTCCCCACAGACTctgtttttcaaataaatatatctTGCAGTATATGAGCCA
GTAATTACTCAACAACATAAAGCTGACTGTAAGATTTGGAGCTTAAAGCAA
ATCGCATTTAATCGTAATTCAATATGGCATGTGAGTGATAAGAGTAAGGATTT
CAAACATACATAGGATTTAGAAGAATGAAGGAAGGACAAATTCTGGACAGT
ATCAGAattagtaaaagaaaatgttaaaggGTAAAAGACTAACATATACGAATGTGTTGGtt
tccttaagaaaagaaatattttttttttctgtatgttctaCATAATGTcagtttatttgaaaaaaaaaatctatatagTTGTT
AAGTCc
>TCONS_00555953
CCCCGGGGCTGCGTGCTCAGCGCCGGCGCTCTCGCGGCCCCGCGGGGACG
TCGCCGTTCCCTTTCCAGACCAAAGGTTACCGCGCGGGCGTGCGTGGCTGT
CCCCGAAGCCCTTTAAAGGTGATTTCTCCCCGCTTTTCCCTGCGGAACGCG
CTCTTGGAGGCTGCTCTGCatgaaGCATGGAAttgctgaaagagaaagttAGTGCATCCT
TGGACTCCTGAAGGAAGAGCCTTCCTTATGGGACCACTTTCAAGcaaatattttgt
gaGTGAGCTGTTCTTCCTCCCGGAGTTCTTTTGGAAAGCCGTGTAAGTGCCT
GTTGTTTACAAACGATGATAGAAATAGCTGCAAAACTTTCACAGGAGATCA
TAAGATAAAAGGGAATCGGTCATCTTAAACAAAACTTTGTAAGTTAATAATG
ATGCTACTGCAGAAATAGTTAAATTGCATCTGAAGTTCTGTATGGGCTCGGT
GTAGACTTTAACCCGTTAGTGGAAAGTTCTGAAGGAGGTGCTAAATACAGA
GCAGTACCGCATAACGATGCAATTAACTTCTATTCATAAATAATTCGACCTAT
GACTTTAAGTTAAGGATTGGAGGAAAGCAAAGCGATGTGATGAAATCCAA
GTCCTTGAAAACGCTTTACCAGTCTGTGACTGCTTTGAGACTTCCTGTAAC
TCCTTATATACGAGTAGAGACAGCTGTGGGAGTTGTGGTTGTatcccccccccgccc
ccttTGCCGTAATTTTCCCCCCCCGATCATTGGTGTATCAACAGTGATACGAGG
TGGATTCTTCAGTTCACAgttaaaagaggaaagatagCGTGTTTTTGATATCTCAGTTG
TCCTAGTGTTGTGGAGTATGTCAGCATGGGTAGCATGTGATTTGTctgagtttgtttt
cttaaagggAAAGTAGAAAGCACTGTGCAGAATGAAAACTTACACTGCCAGCTT
TACTGATTCAACATGCTTGAGAACAGTAAAATCTTCATGATGATTTTGGAAT
ACAATGTGACCTGGAATGGTCTGCTCTATTAGAAAACCACTCTTATGATGGA
GGCAGGTAGTTGATTCCACCATTGGTTGACTGTGTAGCGCAGTCtttcctctggaga
tatttaacCAAATTTTAAGCAGGAGTTGATACTTGAGCGTATTGTCAGGTGGGGC
AGATTTTAGTAGAAAATTTGGGTTTGGGTGCCTTTTGAACAGTAGCCGTGC
CCTCCCTGAGCCTCTGAAGTGCCTGTTGCCCACATCCGCCCCCTGTCCTTCT
GTTAGCATGCTTCAGCATTGGTAAAGAGGAGGTACTTCAGGTGTTGCATAA
CTTTTCTAAGTAAATAGATCATGTGTGTGCAGATATCAAAGGCAGTCTTAGT
GCTGTAGCTACCAGATTCCTCTTAATGCTGAAGGCAGTTGTGTAATTAACAG
CCTTGTGTACTGAACTGAGACCGCTGCTGAACCTCTTGAGGGAATTACTTT
GCCTATTATGTTGGTTAACTTTCCCTAAAAATTAAGTGTTGCTGACTTCCCAT
TAAAGTGATGGGGGAGGATTTTTATAAGACTTAATAAAATGAACCTGAGCAT
TATTTTTCCTGACTCCTGGACAAAGCATCTACAGTAGCAGATGTGGTTTGAG
TGATTGGTTGAAATGCAACCATTAATAAAAGGGTTGCTTCCAGGGGCAACT
GTGGTAAGATAGGAAAGTGAAAAGCTGTGcttaaaaacacttctgtacGTTTTTAAGAT
GTTCTCGTAATTGTTGAAAGAATTGTGACTTATTTCTGTTACTAGCTTGAAT

GCCCTGTTGGgataacagaaaatgatttaaattaGATGGGATGATTAATATTCCTTGTTCCat
cttttctcttggaaaactTTGAGCTTGTTAAAAGTTTCAAACacttagtttttattttacatttccccccccc
ctccctctttGTTTGAATCTTTGCTTTTACAGCATCAACCTTAGGTGGTGTAATTTA
CTGCCTATGAGTAATTAGAGGATTTGGAATACTGCTACTCTTCCCAACCTTT
GCGTGTTTTGTAAAGCCAATGGCAGTCATTCaagttattttctctctctctcatgcAAAGGG
GAACTGTGATGGGTATCTGAAAATTCTACAAGTGAATTTCCGTGGTTTTTCT
AGTGCCATTAGCCAAGTTAGAAACTTCTTATATGAGTAAAACTTTAACATCA
ATTCAAAACATATGTTCTTGGAAGCCAGTCTTGTTAGCTCCAAGGATGGAAT
TTGAGGTTATAAGAACTAGtgggaaaaatattaatattggTTATTAAGGGGTGAATGTTC
TTCCTAtccttttggcttttttaaatggaaggcAAACTCATTTTGCCAGGCTGAAGTTCTCGT
CACTCATGCAGTGTCCACAGGAACCTCAGTCAAGCTTGGCATGATTTGGTG
TTCCAGAACCTGGGCAGCACTGAGAAGTTCTGCAGCTCTTAAAGAAGGCA
CTTGCGTGAGTTTACTTGCTTTGTCAGCTTCACTCACCTCTCCTTTGTAGCT
CTACTTACAGCATGGACTGCCAGAGTTGTGTTTGTTTACTTGTACATGCAAA
TGAAGGCACGACCTGTTTCTAATCCATCTGAGAAATCCAGTGCTGAATGTAT
TGCAGCTGAGTTAGACCAACAAAAAGCAGACTGTGGCGTCTTTCTAAAGC
AGGGAAACAAAAATGGATCTGTTGGG
>TCONS_00854110
GGCTGAAATGATGGCAGTGGGCTCAGGCAGTGTTCTCGGGCTGGGCATGT
GGGAAAATCCAACTAGAAGGACCCAGAGAGGGGGAAACGGTGTGGGTGca
caggaagcagagctgctgaaatgcttcccctgggatggagctgtgaaCTTTCTGAGCATCCTTTTTTCA
GTAGATGGAGCCTGGCGGGGAGAGTGAGGCTGGAGGAGATGGGCTGCCCA
CGAGGCAGTGAGATGCATCTGGGGGCTGGGGAGAGGCCTCCCTAGAGGTG
TTCCCTGGGGATGTCCCCAGAGCCATTCTCTGCCTGGCTCAGGACCACTGC
ACACTCCTGGTTGAGAAACACGAAGCTCAGCCCTGAGATGAGTGCTGCTT
AACAGCCACTTCCCTATTTGCAGCTTTGAAGTTCTGCAGATTGTGTTGGCA
GCAGACTGGAGGAGAGATCAACCACAGAACACCATCTCCTTCCTTGCAAAT
AAGCCTCAGTCCAGccagcaacaaaacagcagctgccCTATTTCCAGGGCTGGTGGGA
GTGCAAAGCCTTCTCCTTGTATTCATCAGCTGCTGGTTCTGCTGTAGCCTCC
AACCGCATGTTGCTGATTGCTTTCCCCATGGTGGGCTGGACTGTGAAGACC
ATTGGGCTGCCAGTGTACCTGTCGACCCTGGGACACCAATGCAGTGGTAGA
GAGCTAATGAATCACAACATCACTTACGCATTAACTTCTTTGAAATGCAAAG
CACTCCCCTGGTATTTCAGTCTTTTCCATCCAGTCCAAAGCAAAACCCCATA
GATAGAGAACAGTGGGGAGCTGCACAAGGTAACACTTCCACTTTGTGTCT
GGTACAATGATTGCATCAGCATTCATTACAATGGAACTTGCTGCCCTTTAAA
GCAGTTCGGCACATGCTGTatcctcagctttcctgcttCCGACTGGCTCTGCGCTGAAC
TGCTTCCTTGCATGCATTCAAAGCAGTTTGTAATTAACAATcacattgttttcctttgaac
atCAACAGAGATATAGAAAACAGAGATTGAGAGCTGAGATGTTGGTACGAC
CGggtattattttgctttcatgaaATGACTTTTCCTATTTTGCTGCAAATTTGGTGCTGATG
AAAAGCTGGGGCAGCGTGCAGGCAGCCACAAGGCTTCACTCACCAGCTCA
TGGACAAAGTTCTCTCCTCTCACTATGGGGAAGCAGGAAATTTGGGCTTGG
ATGCCCTTTTACCTTTGCCCTTGCAGAACACATTCTGGACACCATTGCTGCC
TTCACAAAATAGCGCTCAGCAGAAaagcctgcagagaaggacGAGGAAAACCAAGA
GTATGTGGGCTCCTGAGCTACCCAGCATGttggagcagaggggctgtgttTGAAGCCTT

GGCTCTGTGAACAAGAAGCAGATTACAAACTTCTTCAGTTCTGGTGCAGA
GCTTGGATGGGAGGATTGCTCCCTCCATCTGTTCTTCGCATAGGCAATCTGC
CTTTGAAAGCCATCGCTGCTTAAGTATGGAGGAAGCGTAAATGCAGAAGCA
CCTGAATATTGTTTAACAGATGGGGGCACACATTTTTCAGGCAGAGATTTCC
TTTCCATTGCCCCACTTATTTTAGAGGTGTTTTTCAGGACCATCCTTCCAATG
CCTGGGAATAGGATCCCAGCCTGCGGTTTGTGGCGATGGCAGTTAATTTCC
AGCAGCTAACTTTGCTGTGAAAACTCTTGTGACATTTGTGAAATTTGCTTtca
tctccttcccttccaACTGAGCTCACATTTTTCATCAAAGCCTTCCAGGAGGATCACA
CTCCATGCCACACGCTCCACCCTGGCAACGAAGTGGCTGGAGTTCATCCGT
GCCATTCTGAAACACTCAAGCAAGCCTGATGAAGcacacagccaggctgcagcctgTG
AATGCCTCTGCCCACATGCACATCTTCcctccctgcaggagctgcccttCTGTTGATGAT
GCTCGCTTGTTCTTGCCCCTTCTGCATTAATACATACAGCACAGTGGCTTTC
AGCTTCATCAACCCAAGGACCACTACAAATCTTCCAATGGGAACGCTGTCG
TAGACTGCTGAGTGGCAGAaaggaaattgcttttctttgcaactTTTTTGATGATTCCTTGC
AAACAGATGGTGGACCCCATGCCTCAGGGAGCGCACATTGAAGCCACCAC
CTCACTGAGTTGGTGAAACCAACAGAAATTGTGTCATATGGACCCCCATAA
GGCCCATCTACTGGGAGAAGTcacagctggagaagaggaggaagaaagaagtaaaaatatagGCA
GAGAGGATGGACCTCAAATTTCTCAGTTTACATCTCACTTCCCCTTCCCATT
CTTTAGTCATCATATTCTTTCTGGGAGAAAGTGGCTGttggaaaacagagagagaagac
GACAAACACTGCTGTGGAATTATTTAACGAGCCAGAATGAATTGCATTTGA
AATCATTTAAATGGGATCTGGGAGTATTTAACTGCAGCACAGGAACTTAAC
AAGAGTGGGAAAGGATTTTGTGCTAATTGCTGGCAATGATATACCTACGGA
GAGAGAACATGCAGCAATTAGTAAGCAAGGCTGTAATTTCAGCCTAaggtccct
gcctgccctggAATATGCCATGGATTATCTGTTGCAAGGAAGACAGGGACTTGGT
CTGCAGGTGACCGAAGGGCAGCAGATGAGGTTCTCATTGGGAAGTCATTA
GCCAAGATTGTCACACAGGAGATAGGTCTGCTCAGACCCCACTGCACCTTT
CCTAACCTGACCCCAGCCCTGAAAGAACCATCCAACACAGCGCCAGGTGA
GACCAGGTGTTCTCACTATGGGCTAAGGCAGAAAGCCACCCTGCCTGGCT
GAGCCTCTGCCACCTGACTGTGGCACACAGCCTCTGACAGCCTCTGCTCAT
TGTTTTCTATCTCTGTTCTCTCCCCACGATGCCTCTGTCTTTGGTTTTTGTCT
TTCAATCAGTTTTCATCTCAGTAATTCCCATTCAATGGTTAGTCTCTGATCTT
TCTAATTGAGATCAGAGCTTCATGTAAtagggagggggagggagactatttttaatttgctaCG
TTCCCATTAATAACAAGTTCTAATTAAAAAACCTCCATCTTCCATCCTCCCA
AGTTCAGAGGAAAACTCTGCAACTTCTGTTGGAATCTTCTCCTGATAAATT
GGTACCACAGCCTCCTAGAACAGCTGGTCCTCTTTAGCAGGCATTGTAATTA
ATGGGAATAATAAATGCATTCAAAGCAGGTTCGTAACCTTCCTTTCTGTGCA
CTCAGGCTTCTCTTTGCATTGCAAGAGACACACATCAGCATCTCCGCAAAC
aggcccagcagagctgcctaATTCTAAGAAGCCCAGGTGCCAGTGGTAGTCACTGGG
CTGCCACAGCCCTTGCCATACCCTTTGTGCAGGGCTCCATTTAcaccagctgctggc
tggggctgcCTTGGGGTGCAGATCAGCTGTGCTAGTGATGGACAGACTCTTGCT
CTCCCTCTTAGCTTTTGTTCATCGCTACTGAGTTAATGGATTTGTCTCAGGA
GCGGCCAAACCGGGAGCCATCAGCACAGATATCCCCAAGATGAGCAATCT
GTCCTGAGCAGTGATTTCAATTAAAGGGTCATTAGCGTGAAATCTTATCCCA
CCCAGCTCCCATTTGCCAGCAGGAGGTCCTTCTCCCCATCCCAAGCCTGCT

CTGCGGTTAGAGCGcgctgctcctgctggagccCTCCCGTTTTGctaaaacacagcagctgctgcta
atAAACAAACATATTGATCATAGAGATATGGGCTGTCAGGGACTTAATTCATG
AATGAAACGTGCTGGGGAGCGCCCAGTAGGAGAGGGCTGTGCTTGGAGAG
CTCGAGACCAAGATGGTGCctggaggtgggagcagaggctgcttAGCACTGGGGCTGGT
GCTAAGAGTTCAAAGGCTGAATTTGTTCACGGCACAGAAAGGAGCAGAGC
AAATTTCTCCTGTGTCATTATAGGCCATCACTGACAGGGAGGGAGGTGTTT
CGGGGGAGGGCTCCAGTTCCTTCCTACTGAAGTATTCGGGTCAACCCAACC
ATTTAGGGCTAATTTAAGAAGACTATCAAATATTCTCCAGAAGCCAACCGTG
AAGTTCTACAAGGATCTGAGGCTTGGAGAGAAATAGGAGCTGCCTGCCAG
CTGTGATGCTGGGACGGCAGAGCATCGGTGCAGAAGAggggagagaagcagagctg
caggtttCCCATTTTCCTACCCTCCTGACAGCAGTTTTCCTGATTTGGGTCAAAA
TTTCCCCCCACGTATAGACAGTAAAACTCTCCTTTTGTGTGACATGGGGACT
GTGTGTATCGCTGAGATACTAATGGCTGCTATTGTTTCTATATTGTTGTCATT
GTCTAGCATCTTATCAAAACAAGGTACAATCAACTCACAGGTGGATCCCAC
ACAGCCAGGGGCTGTAATTACAACAGGCGTGTATTTGAATATAGCCTTTACA
GGTGCAATAAATTACGCTGAAGCAGCTGAGGATGGAGGTTGAACTGTAAC
AGGCTGCCATACTTTTGGTATTAGCCACGCCAAAAGCTGTCATTAAAATAGG
CCTCAGTATTTATGGTCCTGGGTAGTGGAGGCCTTGCTAAGATTCTGATGAA
TGAACTTCCAAGGACAGGAATAGCTGTCagtctgaaatatttatcagtCTGAACTCTCT
CTGCGATTCATGCCATGGTTTGGCTGCCTCCGTTTTTCTTCCCGTCTGGTTG
>TCONS_00862230
CCTGTAAGGTCAGGGGAAAGGGTGTTTAATGCGAATGGAAGGACTACTTT
TTCATGTGATTGGGTCTCCTTGGGTCTGATGCTCCTTCCGATTTCCTTCCTTG
TGGCCCCAGAAGACGTGCTGTAGGAAGCAGTCTACGCAGAGAGGTGGTCT
GGATGTTAATGCCTTACTTGTTGCACCTCTGAAAGATTGCTGAATCTGTGGC
TTTGTGTTGCTCTACTCTGGACAAGGATTTGACTGGTGTTTATGTCAAAGTC
CAACACTAAGTGAGCTCATAACAACCGACGTCGTAGTTCTTGATGGCTTCA
AAGGGCATTTGATGCATTCATAAAACATCCTCAGGTTGGCTCTGCTTATGTT
TTATTACTGGCTGTACCAGGGTTTTGGTGatccttcatagaatcatcacagaatggcctgggttga
aaaggacctcaaagatcatcaagtttcaaccccccctgccatgggcagggtcgttaaccactagaccaggctgcccagcta
CTTCCAAAGtgtcatctttcttcttcctttccccattTGAATTTGTTAAAAATGGGTGCTAGTA
ATTAGGTGTTTTGTTACAGATAGAAGATAAAGACAGAAGAAGTGTTCTCAA
CTGTTCTGATTTCTTGCCTATTGGTAATAATTGGTTGTTTAGGTTGCTGTGAA
TATGTAAAATAGAGGAAAGAAGGCGATGAATAGATCCAAGGCTGTACAGGG
GCTGAAGAGCAGTTATGACTCACCACACGCACCAGCATTCACAGGAACTA
GAAAGCAGATATGGCTTCACACTCAGCATCGGCTTTACCGTGAGACAGACG
TCTTCAGTAAACAAGCAAAACTAACACTTCTGTGGGACAAGTTGTCACGTC
CCTCATTCATGACAAAGTTCTCATTTCGGTGGATACTGGATGCGAGAATGTA
ATTTTTGTATCTCCCTATCTCCATTTTCCTCCTGGTGCATATAAGCACACCAT
CACAGGTGTGGCAGAGATGCTGGCAAacaactatatattttttgccaCTCCAAAAATGG
AGTCTTTTCTatctgttttctaaatgcttGAGCATGCTCAGCATTTTGCTCTTTTGGCTGC
AGTCTTTGACAAAACATGCACAGAACTGTTTTTATGATACGCATTTTGTGGG
ATCAAAGAATTACAGGTTTTTAAGGGCTGCATTGTTCTTGTTCCAGAACTCA
CCTAATTATCTAAACCCGATACCTTTCTTGTTTtagatgaaaagatgaaaaaatatcagTGG

TGCAagtcttctctcttctttcagcttAGATAGTTGATcgtttttctgttctcagcaaAGTTCCCcttaaaaca
tgtatttttaattgcacaAGCTAAGTAAAGCTGGCTGCTCAGCATTCAGCAGAATTTTG
GAAAGCCATATGCACTCTAGGAGTAGCTGCATATTTGAT
>TCONS_00877465
CTGTAACAAAGcctgtgtttttctgctcaTCACCCTCAGACACGGCCGTAATTAAATGC
AGATATTAATACCGAGGGCTACTTTGTGTTCCATAATCCGTAGGAGAATGAC
CCATTGCACGGCGCTCGTTTGAAGTCTTAGTTAACCTTTAATCGTTTGATGC
TGAGTCTGGCTTTGATTTTCACAGCAGAAGAAGTGCGTTTTGCAAAACCTT
CAGGAAGGAAACGTGCCAATCCCACAGTATTTCTCATCGTGCTGTGGACGT
TACTGTCAAAACCTCTCGGGCAACGCTGTGAGCAGTGAGGGCTTTTTCCAT
TCCTCGCTTGCGCTCTGCCTCTTGTAAGGCAGCATCGGAGTAATGGCTTAAA
TGCTGCTAACGTTTGTTTTGCTGAAGGAGACCTGGGCCGGTAGAGCAGGG
CGGCGTGGCGCAGGTCTCAGATGTGGAATTTCCTCAGCACGAAGcactgctgga
ctCGGGTCCTGAACCCTTCTGAGAGGTAACGGCTGATGGAAGCcaaggaagcaga
aagctgtGCAAAGAAGGACTTGGAAGGGAAATGTGCGTCCACCATCTGTCTTT
TGTGCAAGATATCGTATGCTG
>TCONS_00948138
CTTTGCTTTATGAAATTTGGACAggttctgcttctcttttgaaCTGTCTGTTCGGCACTG
TGGAGTTCTTTAGCTGGAGAAGTTCTGGGTGTTTTCAGTAAAAGGTTCTGA
AACACTATTATTAGCAAATGCAGTTTTATCATAAATCCAGGATATTTTTGGAT
GGTCCAATGGTTCTTTTTTTTGAGTGTGTAGGTGATAGGGTGTTACAACCTA
TTATTAGCATTGAGATAAACCATAAAATGACTGTGGGGGTTTAAGCAGGCAT
GTTGATAATATGATGATTTACTTTCTAAAAATACAGTGCTTTGCAATTTATGC
CATGCATGTGTGTTTAAATGTAGTTCCTGCCCCCcgaaaggaaatgcttttgttgAGACC
TGTCTTTATCTGCCTCCTTCCCATTCCTTTTatcccagctgctcctgcttctGTGACgggtcct
gctgcagctggcagcatggCAGGCCTAGCAGCGGAAGATTGACGAAGCCAGAAGCT
AGAGCTATGGTGGCACATATGCTGGTAGCAGGGAGAGGAGCTATAGAAGGT
GCACTGGTTGCCACAGCAAAATCCAGGAGCACTTTGGAACCAGTTGTTTT
GTCAGCAGGGACTGAGGTGTCCCCTTGTTTCTCGGTTTCCTTTACCTGCTTG
TTCATCACTGTGTCTCTCATTGCTCACTGGTTTATAGCACAGCTGGTAGCAC
TCCACAGTGTCATCTGAGAATAGGCCCCAGCAAACTCTCAGGGAGGTGCC
AGTTGCTGCATTGGGAGCCTGGGGATTGATCACaggggcacaaggagctgcaGGGGG
GAAAAACATGTCATTTTTTGGAAAGTATAAGAAGCATGTTCTGTTATTCACT
GGCAAATGCTTCTCCCACAAAAGAGTAAACACTAAGTTTGGGCTCTGAATA
AAGTCATATTCCTCTGACACTGAGAAAAGTTAGGAGCTGATCTGTTAAATAT
TTGGAGATGACGATCAGGCCTCTGGTGTATGAACTTAAGTCTTgcttaaaatcaaaa
caacaaaataacataaatCTCAACCCACCCCTCTGTAAACTTCCTCTCTCCTGCCTCTG
AGAGCTGCTTTTCTCCAGCCAACTAGTATAGCCCTTCAGGACTATGCGCCA
CTGTGGACtagccctgctgctggggatcCAGTAGaggaaagatgaaggaaaagtgTTCCAACACA
GGGTCAGCAGCACAAACAAACTAGTGGCAAATgctactttattttaaaaagaaaacaacaac
aaaacaccacagtgtatgtgcagaaaaaaatgtattgtctGGCTACTACTATTTGCAATGTGCCAGA
AATTGTTTactctcacattttctttttcgTTTTTTAAATCATTAGGAAATTGTTTGGGTTTTG
CTCTGGCTTCTGTGGGtgacaggaaaaatatttgccaaGTGGCAGAGGAGCAAGCCACG
GTCATATGGAATCTGATCAGGTTGTTTAATACAGCCTGTGCCCCTCTCTACTT

TCTTCAAGCGATTACAAGGAAGGACAATTTATCACCAAACTCAGCATGTCT
GATCCTTGAACAGAAGGGTGTTTCCATAGCTGCACAAAACTCAGCCTCCCC
ATGCTCTTTGCTTGGATATGTGTGGCCACACATCAGATCATTGCTCCAAAAT
GCCAAATCCAAATAGCTGGATGCAGATGGGACTGAAGGACCACACTTTGTC
TGTTCCACTCATGCAGTGAGGTGGCTGCAGCGCACTCCTACTGAGGAGCTC
AGAGTTTATTTAGTTTGGAGCCTCCTGTTCAGCTGAACTGTTCaacaaaatgagca
gaaaaagaaacatcttccAAAGTGGATGCATCAGCCTGTCTGCTGTGTTCCTGGTGTA
CAGCAAGGAAGCTGAGCCTGAACAAGGCAATGAACACCAGTAAGCATTTC
CAGAGTTTCCTGAGATAAGCTTTTTAGAGAGGTATCTTTTCTCTAAACCCTA
CGAAGACTCATAAACCGTTGAGCAATCAAGTGACAAACATCTGCCCCTGCA
GCTGTATTTTGGATCCATTGTCTTGCTAAGTCAGTGCCACTGTGCCAAACAC
CTGGAGCAGCAACAGCACATGGACCAGGCACCCCAAATTCTCTCTAGCAAT
TAGGTTGCTTGGGGACTTCCCAGCTGGAAACTAACtgcaagcagcaaaaagcatttAG
AAGAGGTCTTGCCAGTCAAAGCTCCTTACCTTCATAAAAGTGACTTTGTCA
TTTTCCTGGTAAAGCTTGTGGGTTGTGTCCATCAGCTCCTTGCAAGTGTCA
AGATGTTCCCCACAACTGACCAGCTGCTCATACAGTGCTTCCAGCTTCTGC
CTCTTCTCTTCCCCCAGTGCTTCCAACTGTTCTTCATATTTCTGAGCAAGCA
CTTGCACTGCATCGTTGTAATGAACCTCGAAGTTCTGCTCCTGCCTCCCAA
AATTCTCCTGCAAGGCATTTGGAGTTTTGTTGTTTGGCGTCAGACCTGAAC
CATAACTCAGCCAAAGAAAAGTCTTTCTTGGGCCTGCCTTAAATTCTCCTT
GCCAGGGATGCAATGCTAGTAGTAAACAGAAGATGTTACTTGACTAATCTG
CATTTTTACTAATCATTCACCCTCACTGAGGCTAAAAAGAGTGTCAGAGTTG
GTTCCAGACATCATGAGCATCAGAACTGTCTCCAACCTAAACCCAGGAAGC
ACTGCTTGGGAATTATCCTAGCCATAATCTCCCAAGTATTTCTGCCTACACA
GCCAAGCATGTCCTTTCACTAGAGAAACCATGGCAACCCAGTTGGATCTCT
GGATTTGCTCTGGGGGGAGCTGAGCAGTTAATGCTGGTATCCTCACTCACT
GCTGGAGAAAGCATTAGGAAGTAAGATTTAAGGAGACAACAAGACTCTAT
TGACAGCAGCTGCCCACTGCTCAGGCACCGATGTTCTTTAGTGCCACATGC
AGTAGAGAGAACAC
>TCONS_01021835
CAGAAGATTCAATTCTGCACttaggaaagaggaagaggactCTGCTGaactgGGAGTAGC
TGCCAGCAACAGCCGCTCACAGGGAAAGGCAATGGCTGTTTACCTGCATtgc
tcagtgggaccaggaaGGACAGACTCATTGTGTCCTCCGCCAGAAGACAGTGACAT
GACACAAATGGTAGCACAAGGCTCTTAGTGCTCCACTCAAGTGATTCTCCA
TGGATAGACTGTGACTCCCCAAAGGCTTCAGAGGGAGGTAAGAATGATCGT
ACAGCATCAAAAGTGCTGTTTCATTCCACAGCCAGCAGGTTTTCACTCCCT
TGAATCCTTCACCTTCAAGGTCTGGCACTCCTTGTGACTCtcttaaaatgaatgag
>TCONS_01084405
AAACTTCAGGAGGCATTAGCTCAGACAGAATTCATCCAAAACCAAGACTG
AGGATAGCTGGAAAGCTGGATTCAGCCTGGAATTTGTGACAACACCAGGA
GGGAGGGCAAAGCGGCTGTCATGAAGCAGTGTGGGCACCcatctctccagcagcag
agagcaatCAGCTACCCACACTAACACCCTAACCCACGTTTCAGGCAGAAAAC
CAGCACTCCCATCAGCACCCTTCAGAGCCCATCTGTACAATGGGGCTGAGA
TATTCCACTTCCTGCCTTCCCTCCAGGATGGAAGCTGGCAGATTCAACGTG

CCTACAAAGCCTTCCCCAGAAACTGCCGCTAAGAGGGCAAAactgttattgttattat
gATTAGAAACCAAGGTCCTTCTGTGCAGTTATTTAATTAAACCATAAATACA
AACGGATAAGTGTCTAATGAGCCAGCTCTACACTGTGTGTTTATATTGCTCT
GAAAAGAACAACGCTTGAACTAGTTGCAAATAATAGTGCTCTCCCCCAGCC
TGCTATTCCCTGCAATCAGGAATCAGGCCGCACAGTAGTTTGCTGACAAAT
TGGTTCGTGTTATGAAGATAATGAAGG
>TCONS_01093310
CTCTGGGAGGTGTTTCACTTTCGTTCAAGTAGATTTAGGTCTTTTATTGCTTT
CATGACACAACTGCTAACTGGGACCAGAACCTGGCATGTGCGGCAGGCAG
CAAAGTCCTACTCCCAGAAAAGTTCCTGTTCGGTTCCTTGCACAAAGCCCT
GTGCTTCCTCTCCGGCATCTCTGGAGCCTCGTAAGACCCTTCCTCTTCCCAG
CACAACTCAAGCTGATCCCATCCGTGCTCCTGGAGCTTGTCGTGTTTTTCCT
GAGCGAAAGGAAGAGCTTCCCAATCTCTGCAGATGCCATCCAACCACCCC
AGCCAGCTCATTGCTGCCCTGCTGAGAGATGCCCACGGGGGTAGCACAGG
CATCGTGCCTGCCTTCCACGGCCAATGGGGATGGGCAGGGACAAAGGTAA
GGCTGGCAGCATGAAAAGGCTGCTTGTTGCAGCGTTTCATGGCCAGTGAG
CGTGACCAGCCTTCAGGGCTAATTACCAGAGGAAAGAGAAGTATGTTCTTG
TCAGCAGGCCTATGGGGTTAGTAGTTAATGAAGCAGATATGCAGGTAGCTT
GTTCGGTGGCAAAAGCGAGTGACTGCCTTGTGTCCTCGGGGATGTGGGCA
AACCCTGGTGTTGTGCTGGCAGGCTTACAGCGTGAGCATGTCTCCGTGCTG
CAGGAATCCCTTTGTATTGCTGGGTCGTCGTGTGCCCAAAAGCTAGTTGTC
TGctgtcccagtgctgtgctggtcTCCAtgcaaaagaagggaaagggcGCAAGGGAGTAAACAAA
GTGTGAAGTCTTTGCCCAGTATGGTACTGCTAATGAGGAGCAGCATCTGTG
GTATGAGACTCCTACAAGTCCATTGTGATAACTGCTACTTGCTAACATCACT
GTTACCTGCTATTTGACCCATCGGTGAGGTAATATTTGACAAGGGATCTCTG
TAACACAAAAAAGCTGACTTATGTATCAGACAACGTTATGCATCTCCTCTGA
TGATGTTCCTTGTGTATGTACCAGAAATGATCCTTTTGTTGTCAAAATGCCA
CCAATTTGAGCTGATGTATGGCCCATAGAACTGGATGAACATCCCAGTGCA
AGACTTGTGACCCGGTGTCGCAGCAGAATGGCCTGGCTGCACATGGGGAG
CACAGAATGCAGCCCTAAGGATGCTGCAGGTCAGAAGTGAGAAGTGTTGG
TG
>TCONS_01105747
CACCACCTCAGTGTTGCATCCAGGATGTGCTACGGGTACCTGTCCCCttgctcct
gcactgctctgacAGGCAGaggagccctgtgctgctcagggctgtgtgaGCAAGAAGCCTTGCTGC
TGCAAATAGGGTGGGGGTTTCTCTTTGTCCTTGGCTTGggtttgcttgtttgtcttAAGA
AAATGATTAAGCTTTTAATGTataaggaaagggaaatgtttCCTACAGAAAATGCTAATG
AGCTGAGCgtttcatttgtttctggaaaTTTGTAGTTCTTTAATTTTATGTATGTAAGGGA
ATTTTTCTTATCTCTGGGACAGTTGGAGGACATTTCAACCTTAAAATATGAG
TTTCCTTTTTAGCGATGTTTAACacatattgaaaagaaaaaggccaGGCTGTATGTATGAA
CAGCAATGTCATGCCCTGAAGCACACATCCCCTTTTGACCACGTGAGTCAG
TCCATGTGCAGCTGAGAAGGTAACACAGGATTTTCTGTACTTGACGCCTTT
TGCAGATGTGGTGCTGgggctcccagctcagcctgccaTCTCTTTGGTcgagaaaaagaaatcat
gggAACAAAAAGATGTTCCTATGTAGGAGGTTTGTTCCATTCCAGATAGCAG
CGTCATGAGGCTGAAGCTGGGGAtgtgctgtgggctgctctTCTGCGATACAGGAAA

ACAGGCAAGTGTACAGAACATGTCTGAGGAACAAAGGAAGCTGCTGCCCT
CATAGGGGTGTACTTTGGTTGCAATGAATGTGACTTCTTCATCTCTGTCCAA
CGTATTTTGATCAGATGAGAGACAGTGTGTCCCTGGGCtttgtgctgctcagggctgccA
TGCAGTGCTCTCAAAATACCCTGGGGTTATCTCCCAAGATAAGGAGCTTTGT
ATTGTGCTCCTCAGCTGGGCTACCACCCTCTGCTTGCTCTGATCTGCTCCCT
TAAGAATTCTGGTGTCACAACCCTAGCACACTGGATGGAGAAAATAAGAGT
GCTTTGTATTCTAAATTTACAACACAGCAGGGTGCAGCCGGCTTTAAGCAG
GACAGAGACCAGAACAGCAAGGCCGCAAGGCAACTGAGTTTGACCTGCT
CTTGATGGCCAAATGCTACTTGCTCTATTTAACCATTGTGACTGCTGCAGAC
gtggagcaaagcagcagctctg
>TCONS_01119732
CGTTTGCTCTGTCAGCCTCCATAGCGTTAGCATGCTTGGCCGGCTGCGGGG
ATGTGTCGGCACCTGCCTCGGCAGGGCAGGTTACAAAGCCTGTGCCCCGG
CAGCCTCCGTGTTGCTGGCTATATCTCAGCCTCCACCTCCATCAAAACCCTC
TGGGCCTTTTCTGCAGCaatttgtgaaaataaacagcagataACATCTGTTGGAAGGCTtgtt
tgctgttctttccttctctttcgTCTCAGAGTATATAATTAAAACCAATTATGACAAGCGCA
GCACGGTGGGGCCCGGCGTGCCCTGGCACAGGAAGAGCCTGGTGAGGAA
GCACGCCCTGAACTCTGCGCTCCGGCTGCCACACCTGGGACGTGCACGGA
AGGGCCGGGATGCGGCTCCCTGGCCCGGCAGCAGCACCAAAATGGCCACG
CAGGGGTTCAGCAGCGTGGGGAGAAACAGGAGAGGGCGAAGGTGTTTCC
TCTGCTTTCGGGTGCTTTCTGGACACCACTGCCATGTTTTCCTCTCAACGGC
TTCATTCTTCTCCGAACCAACGCTTGGATCCGACTTTCCCATCAAGGGATTT
TATGACAAAGGAGCTCGTCAGCAGGTTTCCACCTGTGGTGCTGACAAGGG
GGCTTCTCGTGTCACCGAGCTGAGGAACACACTGCGCATGTGCCACGTGA
ACCCCTGCATTTGCTTTGctaaagcacagcaggaacagaCCCAGCAGCTATAGAAACC
ACCAAAGGCTGAGAAGCATTTTGTTATGTTAAAAAGGGAACAAACTTACGC
AGGTTATAACACTTACTTTGCATTTATAGAGCATCCTTCATCCAAGGACATC
AAAGCACACTGCAAATGCATTCCTGTGACATGCTACATGAGGGAGCAAATA
TATCCACTGGAAAGCACTATTTATGATTTAACGATGTTCTGTGAGCTTCCCG
ACTTCATGCACCAGATCCACAACAGAGCTTGCAGCAGCAAACCCATCGCT
GTGTCCCTCCTGCCatctctctccagctgctgggctctggctTCTCCCTCCGGGGCTGTCCC
AGGGCTCCGAGCACCACTGGCTCCTCTTGGCCAACCCTCCCGGTGCATTGG
CTGGGCTGCCCGGCTCCACCCGCACTCAGGGTGCTCcgtttgttttccttgtgatGAAG
TTCTTTTGCCCGTTGAGTCCTTGAAGTTGGTGGCaagcagaaagctttctttcttttttgaaga
gCTGCCAAATGACATCAGGCTGCTCAGCTCCCTATAGCAATGAGCTGTGAG
ACGCGCACCCTAGGCGAGAAGCAGGGATATGTGCCTTTCCCCCTCAGCATG
GGGTGCCAGGAGCTGCCTTCCTGGAGCCTGGCGTGGCACTACATGTGCTCA
TGGCTCCTTCTCCTGCTTCCGCTGGGAAGTTTTCCATTTAACCCCAACAGGT
CTGCGGGAACCGGCTCTCAGCACACCAGCAGCATTGCTTGGCAGGGATGG
GCAGTTTGgtttgaaaagcaaagcaaaacaaacacaacaggCTCCAACCTGTGTTCTGCTGCC
TGCAATAACCCAAAAGGGTTTGCTCTGGGGTcaaggcagccagcaggacCTTCTGCA
TGGGCGcttgcagaagggctgcagaggcagctgctggtCCTTCCCCATGGCTGTGGGTAACA
AAGAAGCCCCACGGCATGCAGGTGATGAGCAGAAGGATGTCCCTTGCCCA
GGTGACCGTAATTCAGGTAACACAACCTCTCATTTGTTGCCAAttcccagctgcag

gctgccagagtCGGGCTGGCAAAGCCTGGCCATGAGGCTGTGATGCTTGGGAAC
GAGGAAAAATAACGGAAAAAGGGATTTTCAGATACATTTGGCTTTCTAAGG
AGAAAGGCCTGCAGAAAATGTTAACTCACTGGAAAAGTAAAACTGCTCTG
TCTGCACTTTGCTTTTGTACAAAAGGTCagcattttccaggaaaaaaaaaaacaccaaaaagct
CCTCCTCCATTCAGTTCTGGTTCAAAGCATGCACAGGCCTTAGAGCCATGA
GCCCTCTGCAGAGCCACTGCAGCCAAGGGGCTGATCCCGACCCCTGGGCA
TAGGTGGACAGCTATCCCCTGACCTTCCAGGCTCTGCAGCACGAGCTGCAC
CacataattgcttttttattaaagactTGTGCTGTATGGCGCAGTATCTCAGTGTCTTGCTAT
TTCCCCTTGCAACAGACCTTCTTTGTCCACAGTTATCCTAACCAAGAAGCT
CAGGAGCCACTTGAGGGACACAGCTAGATTTCCTGCTAGGCTTCCCCACTC
acccttctgctgcagcctctgctcccatctcGATGGGAGAACCCCCTGAAGAATCTGAAAAAC
ATCATGCCAAGTCACAATAAGACTTCAGTGTGGGCTGGGGGCCCCAGATGC
CACCAGGGTGCAGtgacaaaaatgttttccaggCTGTGTCTGGACCTTCCCAGCCATCC
TCCTCCCCATTCCGTGCATTATCCTCATCTTCAAAAAGAAGCTGTTCCAAGG
GCCCTGTGTTTTTAGTGAGGGCATGGATAACAACTGCTGATATGCATTTTGG
AACTTAACATCTCCTCCGAgctaaaagagaaaaggggaagatAGAAaccaggagaaaggaaagaa
cacaaTGTAAAATGAGGGTAGAGAAAAGCCTTTAAAGTGTAGCCAACTTTATT
CAAAGGCACTGAGAAAGTAATTACATTAGTTTACAAATGCCTTTGTGTTCTG
CAGCCTTCCTAGGTTTGGGCTCTGTTTCCAGTTTAACTTTGTCAGCTTACAA
ACCCACCCCATCCCTGCCAAACACAGGCAGAAGCGTGGCTTCTGCTGGTG
GCCCCACAGGCTCCTTGCCAAAGGCAGAGCGCCCCTGGCCCCTTTGTGGA
CAAAGTCCTACACCTCCTCCAGGAAGGAAAGGTTTGCAAACAACTTGTGA
GCTTATGGCATTTATGAAAGATTTCTTtgatcaaagaaataaaagtggGGGTGGAAAAG
CAAGCGCTTTCAGGCAGCGCGTGCTCAGGTTGCAAACACAACACCCTTCC
GCCAGGTGCACCCCAATCACAACACCCCAAGCACCCATCAGACAGGGCTG
CTCGGTGTGACCGTATTGCGGGAAGGGCCATAAAGGAGCATGAGGTGTCCT
GgcatgctgcagctgcaagTGATGACTTGCAGAGCCACAACAGCTGGGCAGTAGTCC
TGCTTTGCCTTCCCCAAACCAGCCCTGCCAGGGCAGGATGCACCTCATGCA
ACACAGTGCCCGGACATGACCCCAGCAAGGGACCATGATTGGGTAAGCAG
GGGCGTGGGAGATTTGGGAGCGTGATGAGTTTTGGCCCAAGTGGCTGCAA
CAAGCCCATGCTGGCCTTTTAGCAGCACGTGAGACATGGGGTGATCAGAG
AATcgtttgagttagaagggacccttgaaggtcatctagtccaactcccctgcaatgaatacagacacctacagctcaaac
aggtgctcagagcccatccagcctgacctcaggTGTCTCCAGGATCTTCTACACTGAGCACTT
GGAATCTACAGAGGGACGATGCTGATGCCTTTGTTAGCTCTACCATTCCTGC
TGGCTTCCAGTGgaaaaagaaggtggaaaacaaacaccacataTAAATAACTTTCTCGAAGC
ATCTGCAACACTTTGCAAGTGCTTGCACACATACACTCACAAAAGAGCTCT
ACAGATCACAGCCAGCGTGAAGCTACAAAATGGGTTCAGTTGAGCCCACA
CCTGGCACGTCCTCCTCCTCCATAGACATCACGACACAAAGCGCTTCCTTAT
TCCACAGAGCAAAACAACCCTGGCAATGAGGCCGGGTGTCCTGGAACAAA
GCAGGAGTGAGCAATGTGGGGCTGCAACCGGGCACGTCTCAGGGCAGCA
GGACACCCTGCAGGGCCAGACCCATCTCCCTGCTGTGCGTACGAGCAGGC
TGCCCTGCAACCAGGGAGCTGCTGCCGGATGGGAGGCATTAAAGGCAACT
TGGTGTGCTTGCTTGTTGTTATTCAGAACTATCTATCCCGTCCTATAATTGAG
GAACCCCCAGGGAGTGTGGACAAAACCAGTGTCTCCAGGGCTGGAAATTG

TGCTGGATAAGTGCCACAAATGAGGAGAAGTGTGGAGAGAGCCACACAAA
AGCCTCAGGTGGGAGATCGCTCCCCAGACCCACATGCAGTCAGCAGAGAG
CATCCCCAGCATTGGGCTGTCCCGCACCCCCGATCCCACAAACCTACCCCA
GAATCACAAGGACAAAGGCAGGGAACAAAGAGAAGCAATTCTCATAACAT
CTATTTGTTCTGGCACACTGTGCAGCTCTCCGTGCGCTCCTCTCTCACGGCT
AATGGCAGCGCATCCGCTTGAGTGTTGCTGTTCCCTGGTTTCTTGTTGAGA
ATGACATCAATTTCTGTcaagcttttggagcagctcttgcTCTATAAAAGATCCTGCAGAG
ACATTGCTTCCTGCTGGCATTAAAGGAGAACATTTGAATCAAGGACACACA
TCCACAAGTTTTAGACTTGTGAAAGCACATGCCATAAGGTTTCTTTTTCGG
AGCAAAAAATTGAAATTGGAttaaacagagagagaaacagcGCTTTCAGAAGCATATG
AAGGTGGTCTGGCTGTGGTGTGATGTCCAGCAGACCCCCACACCCTCAGA
GTCCCCTGGAGCACCAGCAGTTATAGCAATCATCTGCTGCATTGATTGCAGC
TGATAATTCCTCAGGGTATGCCACTTAAAATGCTCTTGCATTTATAAGCTGTG
GGATAGCGTGCTAGAATAATGCTGCAGTGAGTACAGGCTGTAACTGTACAC
TGGAATAAAGCTCCAGGACAATACACTGGGAtggctctgttttattttccattcccCTGCCT
CTTACCTCTGTCATCCATCACACGGGTGCTTCAAGTCTGAGAACTGTGCAA
CCCATGTCAGATCAgtgcttctttgtttctgtggctgaCGAGAGCAGCCAATTCCTTCCCTG
ACAAGATAGCTCAAGAATTAAATTGTTTTAACAGCTCCTATCGGCTCACAAC
CATCAAACCAATGCCCTAGGacacagaaagagaacagaggTCCAGATGCATGGCTGTA
CACTGTTTTCCTCTAGCAGACTGGGGACGTGCTCATGCCTCCTCCTCCAATG
GTCCATTGCAGGGTGGGGGAGTAGCTCAGCTCCAGGCCATGAGCTCACGA
GGTacaactgctctcagcatcAGGGCAAGATTGGGAGTTATTCATGGAGGTCTTGTTA
TTCCTGGACATAAATTATTGATTTCCCCTTTTTGGGGGGCTTAAGGCATAGG
GTTGCTTTGATCTGTCTTGACTATCTCTTGCTGCACATAAGGAGTGAAAGTT
TGCTGATTTTAATAGGGCTCAAGAAAGGGTGAAAAGGTCAAATGTACTCAG
TCATGATCCATAGGCCCAAGTGAGGCACACATCCTCCAGAGACAGCTGGCT
CAGATAAATGACTTTCTGCCACACTGAGGAGGTGGAAGCAGGTATTTGTAG
GCAAGGTGGAAATGCATGCTTGCTGTAGGTTTGTCTCCATTGTCCTTTGCTC
ACTTAATGaacagcagctgatggagccTGGAAGTTCTGTAACTCA
>TCONS_01162696
gCCAAATGGGAATACAGAGACATTTCAGGGAAAACTGACAGGATCTGCTGC
TCCTTTCATTGACTCCAGTAATTATAAACTGATCCCATGGTAGCAGCAGGAC
TGCATCTAGACTTTAAATACCGTGATTACATTCTCAGTACTGCTCCAACAAT
GAGGTATATCTTGAAAAGCAGTAAATATTCTTGTGTTCAATCCTGCAGCTCA
TTCCTGTTGTACCACGAACATACTGAGTTTGTACGTCTTTCCTCATGAATCA
GATAAATGCATCTCCTCCACAAGCTGCCTATTTTCTTGTATACTGTGCATCAC
TTCCCCTGAAAATTGGAAAGAGAGGGCTCTTCTTCCCCTTTGGGTCAAGGA
CAACTGTAATTCTCTCCAGGACTGTGATTGCCTTGTGTGTCTCACCAAGGG
ATGCCCGTGTGCTGCCAGGGAGCAAACCAGATCCATGCAGTGTGTTGAGT
GAAAGCCAGGTATGCTATATATGGCAAAAAGTTATAAAGCAATTTTTAATAA
TCTTGTTATTTACAAGCAGAATTTTTATTGTACTTGCTATTGAAATCATGGGA
TGTGTTTCTCAACTGTTGTGCTATTATCAGCCATTTCATAGAATAGAATGgggt
gggttggaagggacctcgggatcatcaagttccaaccatCAGTAAGCATGCAACCTCACCAAGAG
CACATCAGAGACTCAGGTTTGGGTATACGCTACTGGATTCTGCTTAAACTTT

GCTGTCTGTCAAATAAATGTTGCTGACAGCTGAGCCAAGCCTCCTGCCCCA
CCTGGTGCCTGCCCTTGATGGTGCTGTGCTGCGTCTTcagcttccagcactgctgctgact
cCTCTCTTGTTTTGTCCTGAGATCAATAAAAGCTTCCGCTCATGAAGGACTT
GAGCCTCAATGAGTGAATAGATGCAAAGTGGCCCAACCCACGAGTCAAGC
TTTTTGTCTCTCTCCAGCAGTAGGACCGTATGTTGGATGCCCTTGGCTTCAA
GCCCAAGGGCTGTAGGTGCTCAGGGAGCTCAGCAGGGGAGCAGGGTGGG
CAAACCAGCAGGACCTGAGCCAGGTGCTTCggaagagctgctggagaaagtACTAAC
ATTTCACTCCTGACAGCTTCGTACCTGGAGCCAGATCTTAGCGTGGTGTAA
CCTCTGCTGCCAAGGATTTTGGCATTGATTTGGCGTATGGGAGTAATTCAGA
GTAGGTGGAGATGGTGAGAGGAATGAACCCAGATgaattttgaaatacttttcagtgaaatgt
tGCAATAGAGCCAGATGATCTACAGGGATCAAGCTCTGATGATTAAATTCCA
cgtttaaataaataaacatattaGAGTCCTTCAGCACTAAATCACAGTGATGTTATATTAG
GCCCTGAAAAGACGATTGAAAGATTACTCATTTAGCAATAttctagaaattattttatgtg
cCTGCCTTTAATATCCTCTGACATGTACTTTTTAAGTGATTTATCCGTGCATTC
ACAGACATCATCTTTGCTTGGAATACAGCAGAACCAGGTCTTTGATATTTAG
ATCCACATAATTGTTCTTGGAATTTTTTCTCTGGATGAAGCTTTTGGCACTGA
TGATGCTTTATGTGAAACAACCCTGTATCTGGATCTCATTCATTACAGatcttgaa
aagaaatgtcttGGCTTTCAAACAGGGACATGGAACTCTTTAAGTGATGGATTTGA
ACTATAAATGTGCATGTACATTTCAATATACATTCCTTAAATACGTAGAGCTT
ATGTAAGGATCAGATCTGTATATTATAGGTAAGTGTAAATTCTAATATTTTGC
AGTCAATGGAATAGGCACCTTTATATTAAGGGTTTGTATAGGGTAATATTTGa
ggaataaatatatttccttgACTTCTGTTTCGGTTATGAATACAAATCAAAAGTGTTTCT
GTACATGCAGTTCTGTCagcttgtttctgctgtttatcCTGCTTTGTACTGGGGTGTTCAG
TGGTTGTCCTACAGGGAGTGCTGGGCTCCTACAGGGAGCTGGGATCCTTGG
GATCCGGGCACCTGGGCTGAGAGCCGTGCAAATGTAAAGCACAACAGCCT
CTGTGCCTGTTTATAAATTACTACAATGCAGCAGCAGTGTAATAGATTTTCT
GTCTCTGATCTGGATGCTTCTAATTCTCGAAAGGTGTTTATGAGCTGTGACT
CatcccactgcacagcagcGTAACTACTTGTAGTGTGTAGCATTTAAGTCATCCATCTA
TCGCTTCAGGTTCAGGTTTATCACTGCAGTGGGAGGGTGGTTCCTGGAGCC
CCCAGTGTGGATATGCTGTTGGGTGGATGGAGTGCAGTACAGGGTTGTACT
AAAGCAATGCAGCTTATATGAGCAGAGCCTGGCAATTCCAACTCAGAACTT
TTTATGCAGGCTTTTGTCTCAGGCTGACCTACTGTGGCATCAGCACTTAAGG
ACAAGAAGTGATTTTAgtctcatctctgtgctgccTAACGCCTGGCCTCTGCTGAGTGT
ACCATGACTGAAGCCAGTGGTGGTCATGTGTTTGCTTCAGACTGTCATAAA
TTGCCTTTATTAATGGCAGATTATATAAATTTCAGCCAAATCTTTATCAGCAC
TTAGGCCAGACACCAGATAAACAGTTGGCCTCAGCCCTGGCCTCTAATAAA
ACTGTACATGAAACTGGATCTCAGCTTTGCTCTTTGAGCACATGCTTAAGC
AGTGAGCTCGTTGCTCAGTGCTGTAAGCTGGCAGTGCTCCTCAGCCTCACT
GCCCTGGCTCTCCTGAGCACTACAGTTGGGGTTGCACCACACTTCTTTACA
CCCCCAGGGATTGGCTGTGCCCCACATTGCACATTTTGAGGCAAGCTTCTC
TCAACATCTGTTCTTTGAGTTCCC
>TCONS_01202028
gtttttctttttgttttgttgttgttgttgtttgtttttgtttttgttttttttttttcagaagaaatcatCCAGGTGATCTCAGA
GCCGGGCACTCCCGTTGTCAGTGTGTAGCAAATACGAACAGGAAGGTGTG

CGCGATAGCTACGATTCCCGGCAGCAAGTTTGATGGAAACTGTTCGAGAAC
GGAAAAAATAGTTCTCCATTAAAAATAGTAcgagaaaataaaagaaaagaaagaaaagggag
aaaaaaaaaaaagaaaag
>TCONS_01203094
GGAGAGCTGACACGAATGAAGAGATTATTGGCTCAGGCCTTCACATTATAA
GTgatgttttccattcagagaAGACATTGTTTGTTACCCTAAACAAAGCTACCTTTGTA
GAACATGCATAGTTTTCAGTGGTTTCAGCTCATCTCGGTACTTGTTAACTGC
ACTGAATTCTGGTCATTGTGCTCATCACACGGAAGGCTTGACAACCTGATT
AAGAAAAGTAGCTGTTAAGTTCAGAGTCAAAATCTGAAGTGAATGTTGCCA
TTGCAAAACATATTGTCCATATTCAGACTCAAAACTGTATTCCAATGCAGTAT
ATGAAcaattatgtaaatatttaaagtaaGATATAAATCTCAGAAGGAAGATCTGTGTCTG
CTGAACTTCCATGACTTTTAAACAGCATTAGCTGTTGCTACAGCTGCATGAC
ATGGTCCAATCCCagccttttctgtgtttgtgctgctcctgcctttGCCAGCATAAGCATTGTTGT
GATCGCATAAGGAAATGAGACCAAGGATTCAGCTGTGTTAGCTCATCTAAC
AACATGTGATGAAATTGATAGTTTCAGACTGCAGCTCTTGCCTGACACGGC
TTTGCTGTGTGGTTATGCAAGTTGTGTATCACAGTGTGGGAGGTGTAGCGC
AGCGAGAAAAATGTTTGGTGCTCCATCTTTGGAGTGCCTGGGCTGATGCTT
TTGGCCACAATGCCACTTCAAACTGTACGTTAAATTACATCATAAGCAAAG
CATATcgtccaaaaaaaaaaaaaggggggaaatcCTCTTCATTTTAACAGTGCAGTTAATCTC
ATTTCACCATGTTCACCTGGCTGGTGGGACATGCACTATTTCTTGTATTAGA
CTTAAATTGACAAAATTCAAGTCTTGTTTTCCAGACAAAGTCGAGCATTTG
TTTAGATGTTTTTTACTATTTCACTGTTACCCCCTGTATGGTTTAACACATTG
CAGAATTTAGCCATCAGTTGCAGGTAATTTTATCATAAATGACTTATAAATAA
CCTAACCTGTGACActaaaagaaagctgaaggcaGACACTGAAGTGTTTGAACTGTCT
GCAACTTAATTCAGTACAGTAACTGTTGAAAGTTTCATACAACAGCTTATTT
ATTCCACAGACTCCTACATGTTTCTCATTCCATATCACTTATACTCTTAAGTTA
TTCAGGTTCAGTAGAGAAGAATCTACATTTAGCTCCTGGATGGCTGTTCAA
GCTATAGTTATAtagcttatatatatatatatgttatatgaTACCAACTCTGTGAAATATTAGAAT
TGATCTTTATTGTAATTTAATTAATATTGATTTTatgaacagattttctttcttggtgCCCACA
GATGATCCTACTGGACACTTCTGCTCACCAAAACGGACTGTGATTATGTTCT
ACTGATATCAGAAGTCTCGTATTTATTTTTGGCCCATTTGCACTTGGCTTGCA
GGAGTTCTTACACAATAAGCTACTATGCCTTCAGTTGTAGCAGCTCTTTGTC
CAAGGCCCTTCTGTCTGGTTATATCACCTAGGTTTGGCTGTCTTTCTGCTGA
ACTCAGTATCCattaacacagaaattaaagagaAGGCAAACAAGTCCCATGTTTGAAAA
GCAGCTTGCATCTGCCATACAAGGAGCTGCTGACGTCATACAGTATACATAA
GCAAATAGATTTTGGGAGCCATTACTCACAAATTCACCTTGTTCATATGTAG
TTTGTCTGGATGAGGAAGCagagtatatatataaaggaGGCTGTAACACCTGCTTTGT
ACGCAGAAGTAGCTTTGTAGCCAGTACAAAAGGcttagctttgtttttccaacaGATTCT
CACTTGAAGATACTGTGTCCTTGTATCACTCAAGAATGTTTGTGTCAGGGTC
CTGAGAGAACCAATAGCCCCTCGTGGGGCTGACCAGCTTCACCTGGCACC
TCTACTCATTCCCTGCCTGTGCCTTGGGGCTGTGTTTAAACTCATCTTAGGC
AAAGTAGAACTCTCAGTTTCCATGctgaaatttcagctttttgttcatCATTGCACCAAGTC
AGGGGCTTGATGCAGTCCTTAAATGGCATTGATGAATGTGCAGATTCCTCttat
attaatttaaatgcataaggtttttttttgtgtgttcttaTTTAGGTTGCTATATTACTTTAGAATGATCT

TGAAAACTAATGATTCTTCCCAGTAATGATAGCTTCCAGATGTTGAGTTTGA
AGGCTAGTTACATGATGCCATTATTAAATTCATGTAGGATTAttggatgttttccttttccc
agaaTTTGTTCTGTTTAATGATGTTGGAGAGCTGAATGAGTCATTTGGGGAAT
AAGAAGGGGGCATAGGTAAGAAGCTGTATGAATTTGGTTCCttggaggcatttaagg
ccgGGTTGgagggggccctgggcagttTGATGTAGTACTCGATCTAgaggctggcagccctgcctg
tagcaggggcgttggaacttggtgattcttgaggtttcttccaaccaaggcaatctatgattctgtgattcattgcACTGC
AGTCCTTGCTGAAAGAGGTACAAAGTAACTTTGTATTTGAAATGTGTGCAG
GCTTTAGGTGAGTGGATTGTTTACGATGAGATGATacctgcaatttttttctggaaagaaagc
ccagcaggcagctgttaATCCTGTAATTTAGCCTAGGGGAGCAACTTTACCATGGTTA
CCGTGTGTTTTAAggtatatttttgtttatgaaaaAACAATGAACAAGCTAAAGCTGTGA
GTAGGGCTGGGAAAGAATTCAAATTGTGCTTTAAGCAGAGTGCTTTCTGAG
TCAGTTAGTTATATTTGTGTCCTTAGCCATAGTACTTTCATTTCCCTGGGTTT
ACTGTACCAGAATTATTCATAAATTTTCAATCAGGTCTGAGCacaggtttgggtttttgt
ttttttgacaGAATGATTCTGTTAGCCCTCTCCCAGTCAGGCATATCACCAATTTCC
AAACCAAATAGTTGGGAATGAGGCAGGCGTAGTATAGCATTGAGTAATTTA
ATTattactaaatattttattctctgaaaagcagcacatcACATTTTCTGATGCTTTGTAACAGGGA
AAGCTAATTTACAGATTAAGTTACTAGTCAGAGATGAGGTGATGGGGAATC
TTGCATGGGGAACGTGTCTGTCTAGCTGATGGCCTTTATACTTATTCCCCCC
ATCTAAGCATTGCATGAGAAGAGTACATACATCTAAAGTGCAGGAGAGAAA
ATTGCACCAATTTCCACCTAGACCACCATGATAAAATAAGTCTCCTTTACAA
CTTTTTTGTAAAGTACTGCTTTGATGCTATTGACATATGGGTATTATTGTAGC
AACTACTTAGTAGAGGTGTGGTTCTTtgtaaattaaaggaaaattgtTCAAGGGTTATAG
AAACTTCTCAGGCTCCTAAGACTAACAAAGGCCACTACCTAATGCTTAGAT
ATTGGATATGTTACATTTTTACTGGAGTACTTTGCATATATGTGAAGCACTGC
TGTTCTTGCACTGGCCTTGGTGTCATAAATTTGCATTCCTCACAGAGCTGTC
TAGTGTAAATTATCATGACACCAAGAGCACAGTGCTAAATCCTTTTACTGAC
CTTGTACCATGGATGGCTAATTAAGTTGGAAAGAAAGGGGAGGCTGTACAG
TTTTGTTGTACATTTTTACAAATTTACAACTGAGGGGATCAGAAATTTCTCC
AAAATTAACTTCTGAGGAGTGAATGACTATCATTGCATATTTTGTATGGGTG
CAAGATCTATTACTTTTGTCCTTTCCCTTTTGCTGATGCAGATGTGCAGGTG
TGATTGAGCTGGAAGGTACTTGGATACTGTAGAGGTGAGTTGTGTGGCTTA
AGAACCTGTATAGCTTAGAGTATCACCAAGGTCACATAGCAGATAGCAGAA
CAGAAGCAGTAATCCATGCAGATCCAGTCTTGTGCATTAACTGCAAAGTGT
AATTCTCCTAGGGATTAAGCACTGTAGTAAAATTCAGGCTGGGTAGAAACA
GGTACCTTTTCATGGCTTTCTGCCCAAACTAATGTAGACTGCTCTATTGCAA
AGCTACATAACTTTAGTACAGCAACACAGTGCTTCTACTGTACTGCAGCAAT
GCTCAGCATAAACTGTTTTTACATGGGAATAGCAAGAAATTTCTTCTGGTAA
CGTGTAATTTCTTACTGGGGTTTTTGACTTGTTTTGAAGCTTGTACTGTTCTT
CCTAGCTAAGGAAGATTCCCCTGTTCCTCCTCCTGAAGTACCAATCATATTC
CTACTTTACCTACAGTGGATTCTGTGAAGCAGTATAGCATCTATAGCCACTT
GGGAGCCTTGTTTGAATTATCATTGGGAGCTCTGAAGTGTTTGGGTACTTCT
TGAAACTGGGAAGGTGCTCTCTGTATTCAGAGGAGAGAGGCACTGCATCT
GACTTtgtaagaaaaatcttaaataggttgaaaataattctgaaaacatGTGTGAATATGCAGTATGTAT
AGCAGCTGTGTGTAAAATGTTGTTAAAGGCTGTTATTATAACCACTCTTCTC

AGAAGGAATGGTGAGGTATtcaaacaggctgcccagggaggtggtggagttccTGGAAGTTTC
TAGAAGATGATAGACGTGCTGAGTGACATGGTCTGGAGcggtcacaggcatgggttga
tggttggactatatgatcttattggtctttccaaccttaatgattccatgattcttttcCTCCAGTCAAGTCctattcttct
gttttactcTTTTTTGGGATCAGTGAATGAGGGTTTGCTTTGAGTGGGTGTGAATT
CAATGTCAGTTGATATGATTCTGAGCCAACAGCCTTTGTTAGGCCGAATGTA
GTGAAATAAGCCCCCTTCAGAAGTACTTTGAACCTGTTTATAAGTAGCATCT
GATGAGCTGTGTCTGAAAGGAGCTGTAAATGACACATCATTCATCTAAATTC
CTCTTCACCCTTCACAAAATAACTatcctgaaaaggaaaagatagtAAGAAATTGAATTA
GGTTTCCTGCTGTTGAATGCTTATGAACAGCAGCCTAATGCAGTAAGCTTG
GTAATGAGGATGGAGTGCAGTGTTAGCAAGGCCTTGACattaaaggggaaaaacaga
TGATTCTGAGACGGCTGGTGTTGCAAACAAATGATCTGTATTCAAAAGAAG
GCATAGAGCAACCATTAAAGTTTAATGGAATAATCTCTGGGAGGAGGTTATT
TTCATGcagctgaacatttttcttattagCATAACACATGGTCAGTTTTGTAATGAGCATAA
TAATGTTGTTCATCATCCAGTTGTTCTGCGTAAAGGTTAAGTCTCCTTGAAA
GTGTAGTCAAATAAGCTAGCTTACTATCCATTTTGGGATGCAATTCCCTTTGT
AAAGAGAGCATATTTGAGTTCTAGATACTAGCTTCATGGCACTAGAAACTAT
GCTTTTTAgaatattcaaaagaaaaaggcagtgtTAATGCTTTGCATTAGCCACCTTTGAAG
ACTTCCTTATGAATTAGCTGTATGTACTCATCTTAATGTGTCCCTCGTGAAAT
GAGTAGGATTCTTGTTATGTGTGGAGCCTGCAAAGAGATTTGATCCTAAAAT
GATCCTAAGGTGAGATGCCAGATTCGTTGATTTCTAGAAATTCTTATGTTGA
GGTGCATACCTCTTGGGAACAGGTTGTGGATTGCATGAGAGGTTGGACAGC
TGGGGGCTGAGCTAATGGGAGAATCAAGTAGTAACCAGAAGGGCAGTAAC
CTTTGGGAGCCTGCTCcccttctgtgctgcttcagctgtgctgccctgttCATTCCCGAGAAAGA
AGAATGCCTTTCCAGGCTTTGTATGGATTGCTGTTAGTTTCCATCATAGAATT
GACACGCCGCTTTTAGGTAGTAACTTCAATAAGGAATCAGTGAGTACAGAA
GCTGTAAATACCGAGGGCTGAAAATTGCAGAAGTTCTATAATATATGCCCTA
GATGAAACCACCCTCATATAAGCTGAGTATTTGTTGCAGTGAAGTTTGTTCT
AAAATTAGTCATTGTGCGCTTTATGATCCTTGTCTATTCAGGATTTTTAGAGG
ACCTGCTTATGATAGCCAGAGGCAATGTGTTGTTGCTATCTATGCTTTGTACT
CGGtccagcatttgcttttttctgttctcacaCTTTTTGTTGTGATCAGCACATTCCTGTTTGTA
TACAGAACTTCTGCCTACCTCTACTCTTTCCCCGCTTCCTGCTATGCTAGTTA
ACGCTACTTTATAACTAGTAAAATTAAACTCAGTTCTACAGACATGCACTTA
TTCATAGTACAGACACACAAACTGTATGTGAGCGTAAGCTTgatttcctgtttgctttcc
taATGCAGATGTTCaaggttggtttgtttttattcttaaagAAACAAGTACTGTAATGATGTTC
TCAGGTTTGCAGTGAGATCCCTGATTTCTCGTGGCAGTGTTTGTTATTTAAG
CCTGCCATAAACTCCTTCCAAAAAGCTTTAGAGACTACTGCAGCATGAAAG
TAGGCACTAGTGGGAAGAGCAGCCTAAATACTGTTTCTGAGGTCTAATGAA
GATCATGGATGAAATCAGGTGCGTGTGTTGAGCCTTAATTAGTCATCTGTGA
AATGGTTCCTAGCCATGGTCCTCATCATGGGAAAACTGGATTTCCAGGACT
GATGCTGCTAGATCTCGAGTTGTCCAGCTTTTGCTAAGTTTCCTCACCCTGA
AACATTTGGTCACTAAAAGGGAAGCCTGGAACCTCTGGTCCTAGTTGACAA
TCAGCAGAAAAATCATGTACGGAATACAACATTCCTGTAAGTACTGTAGAA
ATACTAACAGAGATGTGAATCTGTTGCAAATGTCGGCTGTAACTAGTTCATT
TATGGATTCACTGAGTTATGTCTGGTCATATTATTAGGAGTTCACTTTTTACA

TGTAGGAAAAGACTCCCTGGTTTCAAACAAACATCAATAGAATCTGTGCTC
CTAACCTGTGAGTCTTCATAAAGTCTATGTAGGAGGTCTATTTCCCATCCTC
CCCTCAATACTATATATCAAGGCCAAACTCTCCTGTTGTAGGGGTTTGTATCC
TGAAGTGATAAAACTGCAACATTTTGACCGTATAGTTCTTTATATGAACCTT
GCAATACTTAGATCTCTCGTATTTGGAGGgtttaaaatattataaaaaaataatttctaaccTTTG
CAGTAGAGATGCAGAGAACCCACATAACAGAACTTGAGTGTACACTTAGA
ATTTACTTCTAGTAAGTTACATgagtttttgcatttttgatGGACTAAAATCTTATGAACA
ATGTTGAGCCACTGTGTTGTTGACTTAAGACTTAAAGCCAGAAGTGTGTCT
TGGTGTGTATGATCTATATTTGGCACGCAAGCGCTTCGTGTGTAGAACAGAC
AAGTCTTTTGCTTCTGATGTGTAAAACCTCAGTCTtagaacactgaaaatactACATAA
CTGTTCAGTGTCTTTCTCAGTTAAGGTATCTTTCTTACTGATAGATATCAACT
GAAGCGATTAGAAGCTGTGGAACATATTACAACAATGACTTTAATGGAGAC
ATTCAGAACAGTGGAAACAAATTATGAAGAACACCTGCAATTTCTGCAGTG
TGAACCCTGCATTTTGTCACTCAAAGTTATGCAAATTTATATTCACTCCATCT
TTAGCTACTCACAAACCTATTGTGGTGAGCtaaatttcttcctcatttgGAATCAGGTAT
CTCTGGTTGTGAAGCTCAGAAGCTGTGTACTGTTAATACTTGGCATGTCTTT
CAAAGCGTTTCACTAGGACTGTTCCTAGCTCAGTTTAATTCTGCTGTTATTC
CACTGGTGGAACTGAACAACTGATGTTTATCGTGTCATCCAAAATGCAATG
CAATTGAAAGCGAGCTAAAAAATGATGAAAGGCTATAGGGAGATGACTAAT
AATTCTTGGGCTTTTGATTTATAACAGAAGATAATGCCAAATTTCACTTGAA
CTGTGAAGCAAAGTCCATATTTTGTTACAAATGCCAGATAGaatgaaaagaatttaaa
acatttatcagCAGTAACAATACCATAAAAAATCAACTACTCTTTCAAGTGCGTAG
TCTGAAAAGGTGAACGTGCCAGCCAGCCTGGACTTCTAAGGTGTATAATTT
CTGCCAGTTTTTAAATTATCTCTCCCAATGGAAAGAGCTGAAGCTTTGCAG
CTTTTGACTTCCCAATTAAGATGGGCAAAGCATTTATCTAACCCTGTTCTGC
TTATTGTGTTCCAGGACTCAGCAGGACTGTGTGATTTTACAGAacttttctgtggttttt
gaTTTATGTGCTCATCCTTCAGACCGCTAATTCACTTCATGTCGATAGTGGAG
ggaaaggagagggatgGGAAGAATTTACTTTCTTCAGATGCTTTAAAGGAAGTAGA
AGAATGTAAAGCTATCAGTGCCTCAGTTGTTTGGCAGTCTCATGGTCGTAA
GTCACCTAGTGTAGTCTATTTCCCCATATTGAGACTCTTTGCATCTATCtgagtct
gtttttttcccccattttacAACTGTCCTTGCATCTATGCCAGTTTAAAGGCCTTACAAT
TATTGCATCATGTTTCAGTACATTTTCCTGGATTGGAATGTTGAATTTCCTTG
AAAAGAGGATGAAAATAAGCTATGTATCAGTGCGCTATACAATATCTTACTT
GGCCCTTTCATCCATCTGCTGTATAACTTCAGGACACCTCTTATTAAGAGGT
TCTAGAAACTATTAATAGGAGATAATTTATCTTGTAACGAATCTAGTAAAAG
GGCTCCCAGTATTCTGAATGGAGATCAGAGCAGTCATAccatgttcattttctttttcttcac
catTATTCTCTTGCAATAGCCAGATTGTGAGTGCTTCATGATTATTGTTAGTGA
CGTTGCTAATGTATGTGTTCCAAGCATTTCTTATCACTACGTTCAATTTAGTT
TCAGctattttgaaactgaaaatgctgattACAATATCAATCTCTGTCATACAGCGTGACTTT
GTATATGGGCAGTGCTATCCTCATCTTGGCCCTTACAAAAATCAGTAGAAAA
TCCCGAGAACATTTAATTCAAAATTGGACTGAGCCTGTATACATTACTCCTC
TATTAATACCTGTTTTTTATCCTTTATATTATTTAGAACAGAAGGTAAAGTATG
TAAGCCTTTTGCTCATTCAAATATTGCTGACAAAATCAAGTGTATTTTGGCA
CTTGGCAATGGTGGGCAAGGTTGTCCAAATTGTACTGGTGTTGAAATAGGA

AAAGGTCAAGAAACCAAGCCATTACTTGTGTTTAATAAAAGTAAGGCTGAA
TTATAACACAGCTCTTCTTCAGGAACTACCTAGTTTTACTAAATGTATGCTAT
GCAATATAATATGTTTTCTATTGCCTGCAGTGATGTCGGTTATCACAGGTGAG
TATTTTAATTGCTTCTAATTAGAGGTATGATTCAATTTTCAAGTCAACGAAAA
ATCTGTAATTACGTAGGTTGAtccgaaagtaatgcttcctgtttatttctgtggaaacaacAGTTGCA
AAGAGCAGAGTAACACACTTCGATAAATCAAATTCTCAGATACACAACACT
ATTTTCCAAGATAGTCACCATGGTTAGCTGTGCACTTTCACCTGCATACGTT
CCCTGTAGAAATCTGCaacagtggaggtgacccactgtttcacagctgctacaaTGGCATCACTG
CCCACGTAGTCCAACTTTCACTGTGCTAAGTCATTGTTCGGCCTCCATATAC
ATTCAGCAAGGGTTGATGCatgtcaatgggtgcaactttttccacatggaagaatgcaatgacacatctttg
ctctCTACACATTTCATGTCatacaccattttgtcagagtgccaTCTGTGATATGACggcaaaatgta
atgaaatattggtgggGAGGTTGGACCTGTACTGCCAGTACCACCAACATCTCcttctga
catcatgggccaacataatagGAAGCGTTAGTTTTGGAGCTGCCCTTGTCATTTTAAGA
GTTACTGTAACAAATACATAAAGTCAACCTATCTGCCTGACTCAGGGCTGTC
TCTGGCCTGTAACATCATGGCAATAAAGATAGCTATGTATAATTTGTTGTTAG
GCCAAGTTCATTTTCCCTTGACATTATGAATGCGAATGCACAATGCTTTGGG
GTATGTAGTGATGATGTTTGCCTGTTCATTCTACAGCAGTATATGTTTTCTCC
TTATTCAAGTCATAGATAAAATGTTCATAGGTGAGAACAGCtcagtaaagaaagaaac
aaaagaggaagTAGATTAACACATATTTCAGAACTGAACTTGTTTGTTAAATAGTT
CATAAAGAATAGTAAGGAATAGAATCCCAAAATGGAGTGGAGAGAAGTGT
TCTCAAGTAAAAAAGGCACTAGTTCGAACAGGTAAAATTTGTGATGAAAA
GTGGGAAGGCCAAGGAAAGGACTTGCTGAGGCAGAAAGGGATGAAATGA
TGATAAAGATTCTGAGAAAAATCTGTCAGCGTACTTGTAGATTTGTAGAAA
GGAGCCTGTGTATTGAGAGTTCAGAGATAGTAATACTTAACAGATGCATATC
CTCTCTTTGAGAAAGAAACAGGCAAAAACAGGCAATTGAAACCTGCAGCA
GCTTCCCTTATACCTCTTACAATAATACATGTGAATGTGCAGTTTCTCAGTGG
TGTTTGTGAGGAACAGTTTCCTCAGTAGATGTGTTTCTGACTAATGGATTAA
TGTATAGCTGTTTTGGAACTGTGTGGTGAGTCCTTGAATGCTGTTTATAGCA
CAGGTTGCCTAATTAGTAATCAGGAAGAAAATCCTGCTTGGTGGTAAGACA
AACATGGTTGTGTTTTCCAGACCTAATCTGTAGGTAACTTGTGGATATAGAA
TGAGCATCGACTGTTACACCACTATATTGGCTctcattgctttctgttttcttgtctgCCTGTA
CTTCTTAGCTGTAGTCTAAACAATCTTGAAGTCTGAATTAAATATGGAACGA
ATTTACTCCAGCTGCTAACCGGGCCagcaaaatacagcagagaaaCACTTCAACACCA
CTGTTCAGTTCCCTGTTACTGCTGAAAAAGGGCTTTCTGTATCTGTTAGTTT
TAGGCTTCTTACAGAACTGCACGGTCAGCCCTGCAGTGATTGCAGAGGATA
ATGCTGACTGAGAGTGTCAAAGACTTTAGCTAGAACTGtaatcattttcttccattctcttG
AAGTATTctcttattttctgctgtaacTTTCTCTACCATACATGAAAATTTCTACAAGAATC
TTCGCAAGCTTGAATTGTTCTGTACATCTGAGCAATAGGATGCTCGGTTTGT
CTGAATTACAACGGATTTGTTAATGCCAGCAAAACATTTGGAGATTATTTAG
ATGAATTACATTACTACTATTATCATTCTCCCTTTTCTGTAAAGATAGGGAAT
GAAATTTCCTAAAGCTCCCACTTACAATGCTCATTCTTGCTGATTGGCAAAA
ACTGTTCTACTTAAGAAGAAGAGTTGGCTGTACAGCCAATTTGCTGATGCA
GTCTTAATTATCTTTATGGGACACTTAATTCCTGGTACACTATAGAAGATGAT
GTCCCTGTCCTCATTCCTGTACTTACTCTACGGATGACACTTTGCTCTGAGA

TGCTCCATTCTGGCTCCTTCTAAGAGGTATTGATGCAATGTAATGATATCTTT
CTGAGGTTCTTTTACAACTTCAAACTCCTAAACTCAACAAGGATCTTGTGT
GCCTTTGTGTAAAGTAGGcttgttttctcattcttaGGTTTCTGCTATGCCATTTTGTGGA
GATGTGTCATTCTTTCCCAGTTAAAGGCCAGAATACAAGATAGCAATTACAT
TCCGGCCTACAATCACCTTCCCTGCTTATAATCGCTCCACTAATAGTGTTACA
GATAAAATTAGATTCTTTACACGATCTACATCTCACTGTTGtgtgttggttggttggtttgt
tttctgagattACAAAATATGCTGAAAACTTAATGTTAAATTCTGGGGTGAAAAAT
CACCCTTCTTGCAGGTTTGTCTAGACACAATTTTGTTGAAATTGACACAACT
TTGGAAAAAATTGTCTGAAATACTTGTAAGATTATGTTTTACTGTGCAAAAT
AGTTCAGTCTCAGTTATCCTGGCTATAATAAGTTCTATAGTCTTTGTACAATC
TTCTGAAACCTTCAAAGAATCTTAGTCTTTTCCCTTTACTATTGACTGCCATT
TTTATATATCTAGTGGTCTAACATTCTAACGATCACAATTGATTTCTTCTTGTT
TGATATTGCACTTGGTTCTGGGAAATTGTTCTTTGATAGTACCTTATCTTCCC
AACTGCATGAATATTAGGAAATCCGGTAATGGGAGCTAAATGTGGGATTTCT
TCTATATTGATATATGTGATTACTGTAGTTTGGGAGCATGCAGCAGAGAATTT
AAAGACCAGCTGGTGTGACAACTACTAGACTAGTTTTTTTACTTATTGGTGA
CACCTACAAGGTGAGACATGTAAAGAAGTGACAGCATTCCAAAATGGTTTC
TTTGACTCTGCAGACCAGTTCCTTCTAGACAATCCTCTGGGATTTATCATAC
CACTGCTTGAAAGCTTTTGAGAACAATCTCATGGTATTCCCTGAACATGCA
AGGGATCTTTGAAGATAAATGTACTTTCCATCCTGGAATGTCCTCCTTCTCAT
ATCTTTCATGATTGTGCCATGTTGTCCAAGAGACAGAGGGGAGatggaacaaaag
aaacaattgTCATTTTATAAACCTTCACAGAACTCCTAAAAAACTTCCATAGCTtct
attttgttgttctgtCATCTTATGCAtgatgctttgaaaataatgatAGGTTATACGTTCATATGTGG
GTTAAACCAAACTAGAAGAGTTTGAACAGGCTTGACAAATCGCTTCTATAC
CTCCTACATAAACTGATaggagtgagagagagagagtctgAAATTTACAACACTTCATAT
AGaaatttttatctttatatAGAAAATTGCCTTATGTGCTGTGAGTTTAAGTTGTTGACT
GTGCTCTGGTATCACCAAGGCAAGGTGCAACTTGTCACTGCTTcaaaaggacag
aaagctCTTCATCTGAAGCAGTAGATTGACGGTGactgaacctctgattgaccacctgaggtgag
tgctgagtcagctgcaggggcacgggtgaatgcaatttcacctgagtgaccggaaggggtggagccaggctgcacccca
cagagaccccatttaagggctgactcccaaggaggaaggatctctatctggagatcacttcCCTCGGAGCTCTT
CTGTGAGCTCAGGACACAGGTAAGCTCtttacttcatttcttctttgtaacacgataatctctctggtcc
aatacctgtatacctgtttgccatataTAGACTTACAAAACTACTAGTTATGGAGATATACTCT
AAAAATGAATCAGACCAGAATATTTGACAGTTTTTGCATCCTTTTTGAAGG
ACACATGATCTGCAGTAGTAACCAAAATAAGGTTCTTCAACTCTTctattgccact
gtttttcttttctttggccTTTAGATTTGATGCCAGCATCTGccactgtttattttctgtgaagacAGAG
GTACTGGAACAGTTCAGACATCCCCTTATCGTGGTCAGAAAGCACAGGGA
GAACACAGCCTCTAAAATGAGTCCTCAAACACAAAACTGCCTATTGCCACA
AGTGGAGCATCTAGCAATGACAAaagcttctaaaaataaatgttgataAGATCACTTGCAA
GAAAACGTACTGTTCTTGGCTTCCCTGTGAGATAAATGTTGGAACCTTTTAA
TCTGAACTTTAGAAATCAATaggtatttttgaagaaaaagcacagcctTCACTAGTGCGACA
ACCATGTTTCTGTATTGGtattttctcccttcccatTTCTTGCCTAAAGCAGAAGGCTGT
GATGGTGTTTTTCTGTTAAACCCTGAGCAGAAttatgctgattttttgtgtatttataaTTCTT
TTGCTCTGTACTGTGCTCTTAAACTTCTGTTCCAGATTAAGCTGAGACTGTG
GAAGCCACCCAGTTGGTAATGACATTCTTATTTAACAAACAGTGATAATCGG

AATAATTTCTTTACCTCTACCAAGCTTTCTTGGGATACAATGCTTTAACTTAA
GCAGACCAGTCCACTGTAATTATATTGTTGTAACTGAAGTAGAAAAACTGA
AGTTAAGTACCTAAGCACTTAGAGCTAATTGGAAATTTGCTTCTGATTCTAT
TGGAAACAATTTTACTTGACTTGAGTTTTCCAATTGGAAGTTATGGCAGCTG
CAGTTAATTGCAGCCCTTGGGTTGATACACAGAGGGGCTCCAGGATGTGGA
GGCAGAAACTGGTAAGAATTGCTGGGTTTCCATATGCATCTGTACAGAAAA
GTGGACTGTGCGTTTGGGTGCTATTGATATACAAATAGTATTCAACgttaagattta
aaaaaagaaaaagcattccCTGACTCCTAGTGCTATTAACACTAGGTTTTGGGCAAAA
GATACACTTactgatgaggaaaaaaatatatcttgtTGGGTGACTTGCCAGAGGAAGGTTTA
TAATCCTTTGTTAAAAGTATTTGTACCATGTCTTGTATCAGGCTTGTTTAGAA
TCGTTCATTTCTGGGACAAGGTTGTCCCGTGTCAGATTGAAATGGCATGCT
GTGACTAGCTCTCTTGTTCTAAAATGTGCTAGGATCGTACGAAAGCAGTTC
CTCAGTTTTCACTCTTTCTtagccttcctcttgctcttGCAGGATATCTTTATGGCTTGAT
GTCTACCAAAAGTGCTTCTCAAATGTACAGTAATCAGTTTTTATGCTTAACA
GACAACTGCTTTGCTAGAACCAACCAACACCAggcttctcccttctctttcttgGACAAC
TTGTAACCTTACGTGGAAATTCAGCCTGAAATTCAACTGATTTGAAAGCTTT
GTTATTGAATTAAAATGACCATGAATGCCAATTTCTGCGGCTACAAGCTAAT
TTGGTTTTGTAGTGGAAATATTGACATCTTAATGTATTTCCCATATCTCCTAA
CAAAAGGACAGCAGACATCTGTCTGACTCAACATAGATCAGAGTAAAGTT
GATATATAATTTATTGTAAGTGTATGCATTGTTCGGGTATGTTACTGACAGTG
TGATGCCTCAGGGTTAGTATAGTTACTAAGACCAAGGGCAAATCTTTCACCT
GTGTAAGCAATACCATCGGGTTCTTGACTACTTTGTTAGAAAGACCAAAGC
AAATCTGAGTCatgggttttctgtttttttttgtaattatttttatttttgttttgaattatgAAACTCACTCCATG
ATCCAGCTTCACCCATTCCTGAATTTTGAAACGCTTTattgtttgttcttgttgttttagCAT
CTGATTTCTCCAAAACCAATCCAACCTTCCTTAATCCTGTTAGGTGTAAGAG
CAAGGAGTAATGGTAAGTGTACTGTGGTTATTTCCACTGGTCTTCTCTGGAA
ATGCATCTGTGCTATTCCATAACAGTGACAGTACTGAAACAATTAGTGCCCT
AAGAGCCAGTTGCATGCACAATCTTATAGGACATAAAGTGCTGGGAGACAA
AATATGCTTAGATGCTCTTTATTGACACTATCAGAATACcaaactgggaaaagaaatgatt
ttgtgaAGCAGCTGTCTAACCAACTCCAAGATTTAAATGCAGACTCATAAGGC
ATGGAAAGGCATGAAAGGGGATTTGTGAGGATAAGGAAAATCACAGGAGA
GCAATTaaacagaacacacacacaaaaaaagcaacccaTAAGAATAACAAGTTTGCAGTACA
GATATATCTGAGAATACTGGAAGAAGTGGGCCAGCTGAGGCTCAGTTTGGA
ACaataacagaagaagaaaatcatgcTTTCTATCTTCAACCTTCCTTTTCATCAGTGTCTA
AAATTTCCTGAGCTCCTTTCATACTGCATGAGTGAAGTTCTGCCTAGCTCAT
CTGTTCTGTGGCTGTTCTTTTAGCCTATGTTCAAGCTGATTGCACAGCTGCA
CTAGCAATAGGTTTTCTATCAGTCTTCTCCTTCTGCTAGACAGTGTCATTCTA
TTGCTTTTCTGAGTCTCAACAGctttaattgatttttaaaaatagaagtgacAAAAATAGTACA
AAGACAAACATATTACCCATGGCTGTTCCTGAATGGCATATCATAATTGACT
TCGAGAACAGCCAGCACAAATGATGATAGAAGTAGTTCCAACAGCAAGTT
CTGTGTTGGAGCCCACTTGTGCAACATGAGCAGTTTACTGAGGGGTGAGA
GATTTCGTTACTCAACCACAAAGTGTGACTTGGTGATGGGAATTATATTCAC
AGTTGGTAAAGTCTGGAAGAcaaactttcatttctttttcctctaataTCTGGAGTGACTCCA
GTCTGTACAAATCAGTGCATGTAACCTTTTAAATTCCTGGGAGGTGGTGTAG

AAGGTGTAGAACCATGGTTGAATATGGTCAGCCTTTTTGCTATTAGATGGGC
TGTTGCTTGGCCTGATCTATCTTGTATGTTCACCTTAATTCCCATTACACAAT
ATCAAGATGGAAGTTTTAtaaggggggggggaaaaaaaacccaaaaacttGCACTAGGGGAA
GACAAGAGGGAGATGTTTCAAAGTCTATCTGAAATTAGCAAGAGGCaatattctt
tgcttttattttctaggaaatatatttaaagttgTAAATTACAATTTGAGTAGAACTTGCTTCTTGA
ATCAGGGGTGTTTCTGACCGTACAgagcttcagctgctttttctAGCATGGACCACCTG
GATAGTACGTGTAGTGAGAAGTACAGTGGGACATTGGATTTGATGGCATAA
AACAATGGGAGCAGCATAGAATAGTACATCTGAAAGAGTGAGTTCCTCTAC
ATGGGAGGGGAGGGCCAAAGAAAAAAGCTGGTCATGTAACTTTTAATTGTA
TAGGTAAGTTGCAGTACTTATTAATAAAACACTGAGACACAAGAGGATATG
CTTTATTGCTCCAGTAATAGCTGCTTTGGGTGTGCTGAGGAGTTTTCCTCAG
CACACAACCTGGGACCATCAGTTCCTCTCAGCTGATGATTAACTGGGAGAA
TCATTAGAGCTTAGATTAAATTTACCAGCCTCATAAAGCCTCACAAGGTAAA
TGTGGAGTTGGAAAAGATGTTACAGTGCATGAGATGGGATGACCTCCTGCA
GAAGATACTGGACTGGTAGCTGAGAGAACCAACTGCTGTAGCTGCTCCTGT
GTACTAACTTCTGGTTCAGCTGGATGTGTCCCTTGGTGTTTCTTGCTAGTCA
GTTGTTTCTTACTTACACCTAGTTTTTCCCCATAGTGAGTCTAACCTGTTACA
CAGTCTATTTATACTGTCAGCAAATAAATACCTTTATCATGACCTTTGAGGAA
TGGAGGATGGTCAAAGCGAATCTGCTATGAAAAAGTAGAATATTTCTTAGG
AAGGTTTTGCTTGCTTTCCGTGTGTGCTTCTGGGATGCAGTAAAGGCTTGG
AAATGTAAAACTGCAGAAAGGTTTTCCTATTTCTCCAAATTCTTATAAGCTC
CTGTAGAGctcaaataaaataatctgaGATACAGTGGGAGATACGGGTATTATTTCTGG
ACCCCAAATTCTTATGCTGTTTTGGTTGAATTTCATTTCGGCTTCTGTAGTTC
AAAACAGTGCAGCATGGCTTACAACTGCCTTTCTGATGTCTTCATTTGATGG
GCTTTGAGATAGAATAAAAGAGTAGCAGAAATTCCACACAAGAAACAGCA
ATGTTCAGTACTCTTAACAGCTTTTCCCCAGCGTACTGCTCTGATTCACAAG
GTCCTAAAGCATGAAAGCTGCCTTGATCTGATTCCTCACCCAACACAATTTA
ATCTATTTGGTTTTGGATGAGTGTGCAAAGTTCACATTGAGATAATGTTTTC
ATGTGATGAGTACAAGATAATGTGAAGGGCAGCATTCTCTCAGTGACCACA
TTAGGGCTTTACAGACCAGAATATCCAGAGCTTGAAGGGACCCACAAAGTC
CCAGCTCCAGACTCCACACAGAACAACCTGAGTTAAGCCATGTATCTGAGA
GCTTTGTCCAAATACTACTTGAACACCTGTCCTGAAGTGTGTGGAGTGTGA
ATGCTTGCTGGGGAAATGGCTGCTTTTATGCAGAAGTAATGATGTTTGTGGA
GTTGGTAAGGAACACAGCTCTTAACTCTGGCCACTATTACTGGGCAAGCAT
CCCATTCCCTTCTAGCTGCAACAGGTGGAGAACTATTTCACAGCTCATGGA
AGTGAGCAGTAGCCTTCTGATCATACAGGTGACTTTTGGACTCCCATCAAG
TAACTCTGTGTTGTGGAGCACCTAAAAGTGCACATAAAAGGCAAAGTAGA
AAAGAGAAGTGGCAAAAAACTATCCTCAGTCCATGATCCAGCTGGTGTAA
GACATGAATGGGCTGTGTGGAAATGGATGCTGGATTCTCAGCCTGCTGTAC
CTTGTGCCTGACCAGCTGTATTCCACTTTCCAGATCTATCAGATAGAGCAGC
ACACCCATGTATATTCTGTCACTTGCTACTGCAAGAAATGGTATTTCAGGCA
GCCTGATGGCACCAATGCTATGCATCTGTCAGCTAGCAAAGAACACTATTTT
CTTTTGACACTTGCCTTACTGTTTCACAAAGAGAAAGCCCTAGGTTCGTTC
AAGGCAGCAAACAGTCATTTAGTTAGCTGAGGCAGGTTTTCTTCCCTTGAT

GTTGTATTTTCCTAGAGGAGCGTAGTTAAGAAGAGCATAATACTTCAGAAG
GGTCACAGATAAAGtcaattaaataatatttattatgaATCTACCATTTAGTTACTGATTCTT
TTTTCAGAATGATTTTCAGCTTCTAAATGCTGCATATTTGTAAATATCAGTGC
TTTGCCTGAATGGGCAGCTTTCACCTGGGGGTACTTTACATACTTTGGTTGT
ACTGTTGCACACATGGTCTATCTGCTGATTCTAGAATGCTGTGTTCTGCTGA
TTGATCTTAAATATGAACATAGGTCATAGTTTTAAGTGGAAATTTCATAAAA
CCATGACGAATTAAAAGAACAATAATGAAAGCAAACCTCACAGGTGTGTG
AAGATTCAATGTTATAATAAGATTATTGTTCTCTGTATCATAGGTCTCAGAAG
TCTTTGCAGAACATTTATGGCACTTCTATCTAATGGCAGTCTGTTAACATGA
AGGTTTTGGGGAGCCATTTGTGGCTTTCCATCTATTACATTTGTCATTTAGTT
TCTATCAGAATCAAGAAGTTAACAGACACACCTGCATGTGCATACAGCTAT
CTGCTGAAAATGCAATTGAAAGGGGGTGTAAACCTATTCACAAAGGCAGA
CAGATACTGCagttttcacattaaaaaaatataggattttttgaagctgaaatgaaacattttgatcTTTGACTT
TGAAACTATTTTCTAAAATTGACAAAATAGATTCGACTTGTTCTGCAGTAAC
AAAAGCTTAAGTTACAACAGCTGTGTTTGGCTCCATTCTGGATTTACATGTG
AACTTTTGCAAGCGTTTGTTCAAATGTTCCTGCACATATTTTGTGCCTAAAG
GAAAGAGAactactgaaagcaaaagatCTGTCAGGAAATATGACCTGTGCTTCCATAT
CCCAACTGACTTCATTTCTGTACATCTTATTGCCTTCaccagattttcttctgaactaGAA
CAAGTTAttacagcttttcaaaatgaataagTACATaatctctcctgctgctttttgagAAGCTCAGCTGC
ATAAATTAATTTACCTACACCTCAAGAATGTAGAGGATGCAAATAGTAGTAC
AAGGCTAATGCCCTTAGAGAGCAGATGATTGACTGGAGGTGCCTTTGCTTC
ATCATAATGTCTGTACCAAGCTAAAGACAGCTTGTCATAAAATAACCATTTG
CCAAGTCATTAAAGCTTATTTTGAGATAAATGGATTCTTGGCATGCCAAAGA
TAAGTATGAGGTAGATATATGTTATTAGATCCGTTTAATAACGagataaaagaaatgga
aagaatatTTCCAATCAATTGTCTGTGATATTTACTGTGATGGGCTGGACCAGTG
AATGCATCAGTGAAGTAACTCAAGGCTGAGTGAATATGTTGCGTGTGAGGA
ACGAAGTGGGTGGGATATGGATgtgtttctcattttccctgAAATTGAAATGCTGAGGatg
tttcattttggaaatgctgTGCTAGTCCCTCTGGAAGGCACCACGCTCTCCTTACAGCC
ACAGTTGTACTACCAATTGAggttcttctttcatttttctgaccTATTCTGCTGAGTCACTGA
AGCACAGCTAACCTATTtgccttcttcctcctttcactCTGGGACGGCTCAAGCTGTCAT
GCATGTTCTCTTGATATAAATTAAATATGTGTGCATGCTTCCATGTCATTGGA
GCTGGGAGGCATCATAGCAATTATAGGTTTAGCACAAATTCTCTCGGTCTGC
AAGATGGGAGGCCCCTTTGACAAACTACTGCCACTGAGGTGAAGGGAAGC
ATTGCTTTGTtgttagggatttttttttttttgccttctttggTCCTTTGCCAAAACAGACTCATCT
AAGGACCACAGTCGTTACCCTTGGTGTGTTCCATTTGCCCtctgaaaagcttttatttgc
cAGGTATCATCTCTGTCCTTAACTACGTTTAAGCAAAGTAtgatttaatttgaaatacaga
acaaaggaagaacGTGTCTAAACCCTCTCCATTTCACAGCTACTCTGAGGAATTGA
GGCGAGGGTGAAATTTAAACAGGACCTCAAGAAAAGTGGCACAGTGTcctga
tagaatcatagaatgacttgggttggaagggccctcaaagatcatctggttccaacacccctgctgtgggcagggttgccaa
tcactaAATCAGTCATATGCAGTGGAGCCCAGCAAGGAGAGAAGTCCCACGTT
CTCTCTTGATTGCAGCAATATGATGGAGCTCAGCAAGGTTGGACTCATTTTT
TCCCACTCACTGCCGACAGTACAGTACTTTTTAAATCAGCAGAGTTCTTGG
ATGTGctgcactgtattttaaagcCAACCCCTTAGGAACTGTCAACAGTGTCAATGTCA
GTATGTTGCAGCATAAAGCATGCTTAACATTTCACATGTTAAACAACTGATT

TCCTATCTGCTGAAGCAGAATTCACTTCTCTGTGGAATATTTCTTCAGTCTG
TGTGGTTCTGCTTCTCCTGATGCTTTCAGTGTCCTTCTGACAAACATGTCAT
GATTAGGGCAGACATAATGAAAACTGAGGCACATCACTGGAACATATCCTG
GGCTCTCGTAACTAcaagctgtgatgctgtgaatAGTGTTCTTTGCTAGATTTCTCTGAT
GGAAGCTCTAATTCTCAGAAATCTCCCAGTCTGAGTTATGTTACTGGTACAT
TTTAGCACAGTTGGCAAGCTTACCTCAAGAGATATCCAGGGCTGGAATATC
AAGAGTGTTGGATGCTTGAACTTCAGCCAATTCCTCTGCAGGGACCTTGCA
TGACAAGTACTGTATTTGGCTTGAAAAGAGAGACCTAGCACTGTTCTCACC
CTCTTCTGTAGGGAAAAAGCTGTCTTCTCAAAATTTCAGGGTGATTCTCCC
CAGGCAGAGGTGTCTACCTGTCTGCAGTGCCTTCCAAGGCCAAGATGTCA
GACTGCTCCTGAAAAAATCAGAATTGTTACTTCACTTGATGTGCACTCAGTA
TGTTTATGCCTGTACTTCAGACAAAAGGTTTTGCACCCTTGAGCTTCTTAAT
TTCTCATAAGCACTTCAAGAAGCCTGTTGTAATGTGCATGCTTCTTTCTACA
GCAACCTTTCCCtag
>TCONS_01203097
TTCTTGTTTTAAACAGTGGCATTCTTGGAGGATTTCCCATCATGCCATCCCTT
TTCCATGTACAATCACAATATGCCAGTGAATTGCTTCCCTGTTGctCTGTATTT
TAGGTACCTCTAATGAGGAATTTATATCTGAAAAAGAATCCACACAACCATA
ACATCGAGAAATGTGATGTACTGCGTCAATCACGTTTACCCTATTAATGTAC
TGTCTCTTCAGTAAGTTCCTTTGAAACATCATCACATCTTCATCTTAAATCTA
GAACTGAGGAAGAAATAGGACTCAATTAATGTGTGACAACAGATCTCCAA
AGAAGTAGTTCTGATGGTATATCCTCAGCAGTGGTAAAGGAGAATTACAAC
ATCATTTATTCTGACgctgaggaagagaaaatcaatcACCTTTAAGGTCAAACTCCTAGT
TTAGAAAGTCATGTTCATCTCAATGTTATTATTTGCCTGACTTACTTGTATCT
CACTTGTATCTCAGTAAGTACATTATCAGTGCATGcatatttcatgcattttgcAAAACC
ATGCTGCACACATAATTgagctttctctttgtctttaGCTTCTTTTGTAATGTGATAAAAA
GACAGGATTTAGTGGCCATGTCAGGCTTGCTGAGGAAACATAACtttgaattttctgt
attttctgtattttggatTTTCAGATATATTGCCAGATTGCATTTTGAGCCTATGTATGTG
CTTGAGCATTTCAATGGAAAACATTGCTCAACTTGTAAAATAACGTGGGTG
AGTCATGGTTCCCAATCCCAACCAGACTACCTGAGagttgttattttaaaagatgccTTTA
AATGTCTTTCTCTGTGGCCCAAAAATGCAATGTGGCAAATCTCATATTTTGC
AAAGTGAAGTCATgattatggaaaaaataagcacttcattttctcatttctttctgtatttgcaCTTTTAGTAT
TTATTCACTTGTTCATTattgctctttttctcttcagtcaCTCTTGTTACCTCCAGAATTGGG
ACCTTTTTCCGGTGCCCCTATTGCCTCCAGTGTTCTCTGCACAGAGAGAGG
CAGTTGCAACTCTGATGGCCACCAAGGAAATAGAGTGAAAAACCTGAATG
TTCACACTTGTAAAACCATACATGCCAGAATCTCAAGTCACATTTGGGTGG
GAGCGTAAGCAAGGGTGTCAGGAAAGCAACCACATCTCGTTTTACAGAG
GAAGACAGAACGACTGCAGAGATAGCATAAGGGCGGTTGAATGAACTACA
ACCAACAGCTAAAGCAGCAAAAGAGGTCCCAGGGAAATATGTCATGTATTT
CAGGGTATGCATTGAAATTTGGTCgacagctgcatttcagaggtgGCAGCCTTCACTGC
CTGCTGGCAGTTGTTCAAACTTTTATTGCAAGAGAGGACAGAAATGAAGA
CACAATAAGTGGGCAGCACAAGACACATATGTTCCATCGTGTAATTTGTGAT
ATGCTTCAGATGGTATTCTTACATGAAATGGATTTTAAGCTCTTAGATTTGCA
AATGCTAAGCATATCTGATGTTATAACCGCTACCATTCAACTTTgacctccttcccttc

cttttgaAGAATTATATCTTTAATACTTGATGCCTGCAGTGACAAAACCTTCACTA
AAAAATGTGTCTCTTCACATCAGAGCTGCCAACGGAACATGTTTTCAGATG
TACCAATGGTTTGGTAACTTATATCTGAAAATGTGAGTCTGATCACTGCAGA
CAGTGCGGGGGGGAAATTCACACTGCCCACATTCTGCTGCCTTCAAAGAA
CCTCAACGTTTTCCACTTTCTCTCACCTCTTTCTGGTGGTGTTCTGTCCCAC
AGCAGCCTTTAAGTGGAGTCAGGGTCTTATTTTGAGCAGCTTTCAGAATGG
TTTCCTTTTCTAGAACAGTTCACTTGTCAAATTTTCTAGTgccaagcaaaagaaaagtt
cCAGTTTCTGATGAATTTAGTCTTCATATTTACCTCAGATCAAAGTGGACCTT
TTTGATGTTTATCTTTTGTCCTGCCcaacttgatttttaaaagacacAGGtactttcttcctttaaagC
ATACACTTGCTATTTTTACACTGCATAACAATATTCACAAGCTGTACCCCTGT
CTATCAACACATAGCagaatactttcatttttataccCCTCAAAAATCACCAGTGTAATGTT
AAATACTGCCTGCTTCCTATAACAACCACTGAGCCGCAGCTTCTCCTCAAA
CCTTCTCATATATTATTCCCTTAAATCTTAGAGTCAAGCGTTAACCTGGAACT
TGCTGTGTCTGTGCACTGAGATCCACCACGAGTCTTTCCAAACAAGCTCTT
TTGTAGGGATGGAACTTCCCCCCCACCTAGCGGCGAAATCACAGACCAAC
ACAAAATGCGAGTGAGTAGCGACGGATTGTGCAGCACGGTGATGGCACGC
CGAATGGCTGACACTGTACGGATTTGATACTTGGGTTATTTATGTGACACTT
CAATAGAGACAGCCTTAAGGTGAGTGTTAATCCACCTGCTTGCATCTCGATT
GTATGGTTTGAGTAAAGCCGTGTCTGAGATGCTGGTTTCAGATGCAATTTCA
CAAGAATGATatgcatactttttttctgaactgaacCCTCAGCAGTGATGTATGTACTTAAAG
AAGCGTTACTCGTGAGTTGTGTTACTCTGAAGGGGTGGAAATACAGAGTGA
AGGCAATTCAGAAGTGCCTGAAATCCTAGAATGCAGCCACTAGAGTGCAG
CTGAACTCTACTCAGGGAACAGAAATAATCAAATAGGTACTGAAGTgctctaaa
aacaaacaaacaaaaaagtaactTGAGTTGTTGCAGTAATAATACTGCTGTATGAGTGTT
TTTTTCGGGTAAATATTTAGTTCTACTGTGTTAGAATCATAAATGTTACCAGT
GGCTGGTGTATCTATTCTAGCCAAAGAAAATGTGTACAAATGCAGAGGAGG
GCAACTAACAGACCATAATAAATGTGTAttgaagaaagagagaaaaaaaaatggttttcttaGT
CTGGGAAAGGAAAGTTGGAGAAaagagggggaggaaggaagcaagcaTAGAGACTTT
GGGTGATAGC
>TCONS_01203174
gctcttttttgttcagtGGGGGGAGGACTACAAAAGTCAACGCGTTCTTCCTGGGCTG
AAGGCATCGCTGGCTTGATGCATTTGAAGGGTTCAGGAGGAGGAATCCTTG
CGGGCAGTTCCTTGTTTGACACTGGGAGCCCAGGGAGCGATGCTGGAGCA
GGGCGTGCTTAGCGCCCAGGTGTCTGTCTGATGGAACTGGTAGAGCAGAG
TGCAGTTCAAGTGCaaagctttttctctgtgaTTGCACTGGAGGTTTGCTGTTGGATTT
TGCAGTGCTGAATACGATTTTTACAAAACTAGGGAAGAGTTGCAGCTTCTC
TGCGTGtaagcacagaaatgtttgtctGCAAACATGGATATTTGCTTTACCAAAACGGAT
TAACAAAAAATGACAGTGTGCTAAGATACAGGCAGTGTTTGCCTTGTGTCC
AAGCACTGCGTCAGGACCTCCTTGCTGATCCTTACATTTATAcgcagagcagctctgt
gttggTGTATGtgatgggagcagagctgctcagcataGCAAGGgcccagctgctgtgctgcccgtCCTGC
TGCCTCAGTgcctccttcttcctctctgccctcttGCTTCATCCAGCCAGATGGCAGCGTAG
CTGTGGGATATGTGTTTTCCCACCAGCTATGGGGGGAAAATGTATTTAACTC
AATAAGGGTGAGGAAGATGCATTATGCTCTTAATGGAATGTGAccatattcatattttt
atttcaagttccCCACCAGTGTTAATTAATTATCACGCACGATGAGCTCATTTAAAA

CGTATCTGGATTCACACGGAGCTGCAGTACATCATTGACTGGTTAAGAAGC
ATTAAAATCTCACTCTTTGGTGTATGTGCAGACAAATGTAGATGTTGTATTA
GCAAGAAAGGTACTAACGGCAACATGGAGAATAAAAATAGGGGATGGTAA
GGAAAACGCCGAAGTTTTACACAATCCATTAACTTGATAGCTTTAGGATCAT
TatgttcatttccttttgaaacatATTTAATTCAAAAGGAAACTTATGGAAGAATAGTCTTC
AGGCACTTAACATTACTTAATCACTCTAAAAAGATTCGCCTTTTGGTGTAGC
AGTAAAACTTTATAGGATGATTGCCTATTAAGTGCCTGTCCTATAGGGATAGT
TATTTCTAGTGCAATAAGACCTCCAAGCTGTGAGGTACTAAGTGACCCTGAt
ggaataggaaaaaataatgttgctTCAATAAGTGGACTTATGGTCTGTTGAGAGGCTCAC
CTCCTTACTGCTCTTGCAGTGGTAGAACAATGTAATTCTATGggctttcttcttgtttgca
AGTGCATGAATTTAATTCTTGAGCAAATGAGTTCTCAGAGGCTGGGGGACA
CTCCACGAATGACACCGATATGACAGAGGATGCCATGTGTGAAGTTGATAT
TGGGTCAAAGCAGAATGTGTCTGACACTGGCATTGTGTGTTTTTAAGGTGA
AAGAGAACGAATGGGCTGTGACGACACTAAATGCAGCAGGCAAACTGAG
AGAGAGTTTGACCTTTTCTCCTACGTAGTTAAAAAAACCTCTAAAGTCAAC
TTACCTTGAAAATGACTGGGATTATGAAGCTATGCACCCATCCATACTGGCA
GTTTAGGAATTTAAGTTAGAcagtggctgtgctggtggagaACCCAGATGGGATGTCCT
TTGGGCAGAACAGTGGGACAGGTCAGCTTGCTTGCAGCTACCTCAGATCCA
TTTACTGAGCACTGAGGAGGGGGTTGTGATGTTCTTCCTTGTCTTTAGAGT
GTCTGATTGGTTAGAGGAATGCAAACGAGCGTCCAAATCAGATGtccccctgctct
gggtgccTCCAGGGCACACCTATCGACTCTGTGCTCTGAATGCGGGGCCTGGA
GTCTGTTGCAGAGGCACTTCCATGCTTTATTCTTTTTGGCTCTTTCTCTATTT
TACCATATCGTGTTTTCATTGATCActgttcaattttttcatattttagaCTTGGAGAGTCActg
ccacagcagggctgggctgcagacAGATATCTCACTTTACATCAGCAAAGATCTCCTTGC
ACCTGGAGGAAAGCCAAGCACCGGGGAGAGAGAGCAGAGGATGAGATAG
TGCTGCACATCAGAACGGCCTTGAAACGAATGTCGTGTATCCTCAGCTTTG
AGAACTGAATTCCATGGGTTGTAATTGAATCCTTTGTCAGACCCATGGGGCT
CAGTTCTTCAGCTTGGATATTTCTGTCTTCACATACACGGGAGATCAAAGAA
GCACTTGATCATGCGCTGACTCTTCCGGATTCTGATAGCAGCaaataggaaagagaa
agaataactGATGTGAATCACTCTGACAGGGAGGTTTCACTGCAAAGACCAGGC
TCTCTCATGTCGAGTAGCTGCGCAGTGATTGAGCCTCTCCGTGGCTGATAGT
CTGTCCCTGCATTGCTGCTCGCTGGTTAATTCAACTCTCCTCTCAGTTTTATC
GAAGTTACATATCAAGTGCTGAGATGATGCTGACTGATTTATGCTTCCCAAA
AATTGTGTGGTGctgctgaatgtggctctgCTCCCACATGGGACCAGCAACACCCCAG
CCCTGCCAAGTCTACCCACTGTTGgtctgctgcagcctcctgtCTGGGATCACTGCTGC
ACAGCTactgtttgctctttttgGCCCCCTTGAGGTGGACCCATTCAAGTTGTGGGCT
GAAAAAGTTTGTGTTTCTTCCTTGGAAGATCTGTgttaaagtttattttctttcaggcgtcagg
aaaataaaatttatggTCTTCAAACACGTAAAGAGGTATAATTACCAACGAGATTTCT
ACTTCATTCCTGCAATAATAACCCTTcctaaattacatttttcaaacTCAGTAAAATGGAT
CAGTCACTGATCTCATCCCTGTGGTGCAATGCTGTTAAAAGCACAGGAATA
TTAAAATCCCACAGGTGCAGGAGGAGCGTGTTCAGCTAAGTAGTTtaaagctgct
gtgtgccttttattctgaaaatgtatttacacTAAAAATAATCAGATGTAATCACTGTTGCCTGTG
GATACAGATGTACCTTTGTACCGATGTGCTTTTCATATAAATTTTATTCCAGG
GTTTGAAGGGTTTCTTTGTGGCTGTCTGAAGCATATCATGCTGCCCAGTTGT

GAGACGACGGATCTTGGAAGGACTCACCAGTCccaccagctcctgctgggagaTATCC
ATTAAACAAGAAGAAGGAGAAACATACACAAACGAAAGCGAAATTCTGaag
tttcatttctgctgtactCCCTGTCAAGGAGAGGGAATTAAATTGGAGagTCATTCAGGT
GAATTCAGCTGTATTTGTTGCTGCTGTAAACTTGAAACTCAATTTTGG
>TCONS_01203305
ctggcagggatAACTGGAGATGTCTGCAGTGATGGACTGgctgccagcccagctgcctgggac
tcctttttccctcttgctCGAATCTTCTAGTTTAGAACTCCACTGGAATCAACCTTTGCA
GTGCACACGCAGTGCCAAACCACCTCATCTTCCACTCTCTGCGAAAGCAC
AAAACCTACAGAAGGAGAATTTAAGGACAACCTACCTGCTCTCCACAACA
GCTGCACATCAGCCACACTGTACAGCCAGATACAGGACGAGATGTTATGTC
TGGTGGGTCTCCTATCGTGCCAAGCTCTGCATAGCTCAGAAGTCCGGGTCA
GAGGGCGGCTCTGAGCACAGAGGTTGTCGGCCAGGCCCTGAGCTGCACGT
CCATCAGAACCCTCAGCGTGAGGCAGTGTTCGCCAAGGCCCGGCTCCGAG
CGATGCAAGAACGGCATTTTCTCCCCGCTGAAGGTGACAGATTCCCCtctgcaa
atgcagcacagacCCGAGCTGAGCACGCAAAGCGCCAACTATCGACAGCGGAGAT
AAACTGCGAATCGAAGCACGCTTCAAGGGATGCTGACGGCCCCGAGGTGA
ACTGACAGAGAGCAGCGCTTAGCGAAGCggctttctgagctgcttcAGCCGAGGCAC
GAGTGTGGAGATGGTAAGCAGTCAGCTGAGGCCACGCGAGGAAATGAACC
CAGTGTTGCACAGACGACAactctgaagcagcagcagaaatgcgCTGTAACGGCTGCAG
CGCTGTAAGCGCTCAGACGGCGACTCCCGAGCGcggagcagcagcacttcccGACCT
AACGAGCGCGCAGCGCCACGTCACGCACCGCGAGCCCACGGCCGGGCGG
ACGCACGACAAGGACAAC
>TCONS_01223472
TGATTCCTGCGTGCCTGAGGTCTTCCCAAGACATCACAAGCAGacagaagagagg
cagaagTCAGTCCACTGAGATGAAGATAACTCCCGACTGAGAGATCTACTTCA
GCTGGCTTCGTTATGACCTCTTTGTAAGGGGACTTGGTGGTGGAAACGAAG
GCAAGAATACAGGAATGGTGCAGTGCCAGAGCAAGCAAAGAGCTTTAACA
ACACACATTTCATTGCTTTAGCCTTCAGAAGACACCTCATGATCCTACAGGC
TCAATTTCTCTGCCTCAGGTATTTATCAAAAATTGGGTTAGCTCCAGATTGC
ATGTGCATTAAACAGGTGGCTGTCCTCCAATAGTAAAGTGAAACAGTAGGA
CACAAGAAATATACACAGCAATTGGCCTCATGGCAATTCTGCCTCTCCATGtt
attaagaaaaaggaaacaacttcCTACCCCCAGCCTGCCTGAGCAGCCCAACACTCCGA
AGGCTTTAAATGTAGGGAAAGAAGCAGCATGAAGGAAGGGTAGactaaaggaaa
taaagagccTCTGCAAGAAATCAGTATGGGACAATCCATGCCATGCCGAGCATGt
aatagaaattaattttccccTGGAAGAAGATCCTGTCATTAGACCTTTAtgtcacatttttctgtttgc
caTATAATCAGGCTTGAAGTTGCTGCTCCAGGAGCAAACTCTTAACAGGgcctc
cctcccccctctcAAATTAAAGGAGAGATAATCTATAATCATCAAAAAGAAGATAAT
AGATGCTGACTGCTCCCTAAAAGTCGTTTTtgagttttcctttctgagcAGAAGCTAAA
CAGTGTCTACTGTTATTCATTAAGCAGTCAGCATCCTCCTCTGAAGAGCTGA
CGTCAGTCGGCAGCCTAGGACCTGCAATTATCATCCCTCACCATTGAAGTG
GATGGCTGCTTAGTTACAGCACGATGCACGTCTGAAAGGATCTGGCTGACA
CCAGCTGCCCTCCTTCAAAAAGTTTCA
>TCONS_01257308
GACCCGCTGCCCCCGCAATGCAAGGCCGCGCTGGCCTTCCATTGCAAGCA

AGAGGAACAACACCAGTccgctccctgcagccaggggaGGGCCGTGCCAGTGCCAGT
GGTTTTATTACAAGCTGCTGACCGAGATttcCCCTTGCTCCGCATTGACAACG
TGCTTTGGCAAACTGTGTCCCAGGCCCTCCTGCTCCCCTGAAAGCAGCACG
GGGCTGGGGAGTCAGGCTGCCTCCAGCCTCAGCTCTTACGGCATGCTCAGT
AAGGTCAAAGGGGGCCTGCTGTTAATTTGCTGATGCTTGAGCTTTGTTTCTT
CCCAAAGGAAGCAAGTGTGCCTGGAGGACAAGAAGGAGGCACCGGGTGA
GGGCATGCAGCAGACGCACAGTGAGCCAGAGGCACAAAGAACCCTGAGA
TGAGACCCCACGGAGCTCAGAGCAGCAGCCACGAATCCCCAAAGAATCCA
CGGTcgctttggaaaacatcagcCAGGCCTCTGGTGTCCCCGGGGTAGAACATGGATC
TCAATAAAGCAGAGCATCCGATGGCTGCATCTGTCCCACGCGCTGTCCGTG
TCACACAGGTCCATGCCTTGTGCCAAGCTCCTGGCCATGCAGGAGGACCCC
CAGAGATAGGTTCCCCAGTAGTATATGACCCTGATCGCCAAAGGACTCCCG
CTCCCCACCTGCCTCCCCAGTCATaggatggcttaggttggaggggacctcaaggatcgtcttgtttca
acccccctgccatgggcagggttgccaaccactagatcaggctgcccaggatcccatctcACCaggccctgaatgcct
ccagagatggggcatccacagcttctctgggcagcctgttccagcacctcacccccCTCTGTGTAAATCCCT
TCCCGCTCCCTTCCCCACAGTTCACAGTGTTGCCCACGTGGCAGGCGCTGT
AGCAGAGGGGGAAAGGAGGGCACTTCTCCCCACGCCTGCGAGAAAGGGG
CCTTGTAGCTCCTCAGTGGGCCACAGTGTCCAGTGGGGCCTGGCAGGTGTT
CCCACTGCCGGGTTTAAGTCTGCTTCTTTTCCCATTC
>TCONS_01279468
CGGAAgggcagagaaagggaaggggaagagaataaaaaggaaggagaagagaagagctgCGCTGTCAC
TGCTACCGCCACCTCAAGAGGCCTTCCCAGGCGCTGGCCCAAGGAGGTCC
CACAAAAACCACGACGTCAGGAAGAGAATGCCTGGTGATGAGCTACAGCC
AGCTCTGTTGGCTGCTGGGTGTGAAGCGGGAGGACCCGAAGCCCGGTCCC
TTGCACGTCGATGT
>TCONS_01279624
GATCCGATGCTTTCAGGATTCAAGTAGGGCAGCACTGACAGGGAAACATTA
TTATGGTTAATGAgcagcatgcaaaaaaaaaaaaacaacaacaacaacctgAGCTCCAGAAAATT
CTCTTATGAAAGTTACTATGGGTATCTCATCAAAGCCATATTAGAGAGACTG
CCATCCTGTTTTGTAGGACAGCAGCAACACCAAGTCCCAATCAGGAAATCT
GCATTAACAGGTTTTTACATACACCTAAACCCATGTTCATGTCTTTCATGATC
CGTGTAGACAAGATGAATGCGATCAAGGCATAGAGGTACCAAGTGACCATC
TCTTGTTTGCTCAGGAAGACTTTCACTGAATGCAGATCTTCAAACCCACTC
TTAAACTCTCTTAATCATAACAGTGGCcttcttcaaatattttgttcccAAAAGCTTGCAG
GAATAACCCTGCCCAGGAGCTGTAAGAGACCAAATATTAGtaatgctgctgcaggag
cagagctgagtcATGGCGCTGCAGAGACCAGGGCCACTCACTCTCCAAGGCAGC
AGCAGTATTTGCTGTTAAAAGGGTTTAGACGGCAGTAGCAACGACTCCTAC
ATAGAGCACTTCTGAAGTGTTTGAGACTTCAAGGCAAGACTGGAGCAAAG
AAATTTCAGTAGGAAGTAAAACAATGCAACATTTTACTGCAAACATCACCC
TTGTCATCTCTGTGGCTCTGCCAACCAGTGCAAGATGGATGTTTGTCGGAA
GGATTTTGCTGCTAGAAAAGAGCAGAGACTTCCAACCTAGGCAACAGCAA
CACAGACTGGCAATTCGACACTGAATTACAATAGGAGCTGTGATGAGCAGT
CTCCCTCTTACAAGATTGCGTCTCATCATGTTCACGGAGACATCTGACCCAC
GATGGGAACTTCTAGACATGCCCGAACCACAGGCACAGCCAGAAGTCACA

GTCAAGGACGGCACAACATAGTGAAGTCAACGAATCAACTACTCCATTGG
CACATCTTACAGTCACTGGGCTGTACTGCATTAGTCACACTCCTGAACCAGT
ACAGAAGTGGCGTTTGCGGGATTTTGTTCCATGTTCCACATCCAGGTCAC
TGCAGAGACCTCTGCAAAATTCCAAAGGGCTCGGAGACAAACTGTTGTAC
ATTCTAGAACATTACAATGCAGTTTCTAAAAATAATGCCAATTACGATCAAC
GCAGCAACATTTGGAGCAGAAATATGGCAGAGGAGCATTTTCCAGAAGTG
AGTTTCAGAAGAGAGACCGCGAGAGGGCTCACCATGCATCCCCGCGGGCA
CCGCAGGAGCCGCAGGCACGGAGCTCAGCGCGGCCGCACTGCACTGCTG
GTGGCTCCTGAGCAAACCGAGCCGGGAAGCCCAGATGCAGGAAATCAAAC
ACGGAGCTCTCACACACACTGGCAGAACAGAGATGCTGTGCATGccaaaagcat
ttccatttcGAACCGCTGGGATTTCTCGCTTTGCAGGCACGGGTTATGAATATGGG
GAAAGCATTATTCTTCTgttcaaagttatttttcattaattaatgAACACAGCTATACTCTCTCT
GTGCAATGAAATGCGAGTGTCATTTAAGCAGACAGCTAATTGTTTTATAGTG
CAACTAGCCAGCTCTCCTTTGTGCCCATCACAGACTGAGCTCAGGTGCATT
TCTTAGTCggtccatttatttattttctgataccACAGATCTGCAAAGATTTACATAATAGCA
TGAATGTCTGCTGGTAACATAAGCCCTCTGTTTCTCTGCAACACCTCAAGC
AATTTGCTGACaacatttttctgcaaaattagATATGGAATTTGGAGCAGGAAGCTGCTG
TCTTGACAGACAAGAAAATTCCCTTGTTACTGAACAAAGGGTTCCTCTTTA
TTTCGTGGCCAATAATGAACCAAGAAGGTTTGAGGGAGGGAAAAACATTG
GGAGATTAATTATGATGAACTGATTTGGAAAAGCAGGACTCGCGCTTCCCT
CCCCCCGGTGCCCCCCGGCCCTTTCCAGTATTGTTAATGCAAaacaaatcaggaaaa
aaaatgtacaggaAAAAGGCTCAGAACTCCCCCAGGAAGACCTGAAACAATGAG
ACCAAACCAGAGGATGTGGGGATGCACAGCCGCAAGGATGGAAATGCCCA
GGTGGATTTCAAGGCTAAGAGCAGCCTTCAGCCAGCCGAGAGATGGCAGA
GGCGTTGGCTGGAATTGCTCAGCACAGTGTTTGCTCCCTGTGGAGGCTGTG
CAGGATCCAAGCAGGGGTGGAGGAGCAGGATGGGGGCATGGGAGCCCAG
CAcggctctatggggcactggAGCTGATACTTTCCGAAAAATCCTTTCAAAAACAGGA
CTTGGGAAGAACTGAATAATATTCTTATTTCAAAGTTGAATTGTTTCAaagctga
aattatttcaaagctgaaattatttcaaagctgaaatGTTACAGTACAGTTTTATACTTAAATTCACT
GTAACAGTGAAAAGAACAAAGGTTCTCTCTTGTTTATAACAAGGCAAAAG
CCTccacaaaatacaaaaacaaagctatAGGAAGCCTCCTGAAAAAGGCACTTTCCCTAT
TCCACATTCACAGTTCCCTGTTCCACTCCCCCGGCTGATGCAGAAAGTTCA
GAGCACAGCACTCCAGcacgcagctcctcctgcccggCCCTCCATGTGCCCACAGCAC
CCTCAGCCCGCGCTCCCTCCAGCACCGGGTCTGGGTGAGGGGATGTCACA
GGGCACGTTCCATACCAGGCGCACTTTTGCTGGTACCCACTCTCATTCATAC
CTTCCTGTGTCCTTCACCAGTACAAACTGGCTCCCTTCATTCAGGCTTATCT
ATTTTTACGGGCTTTCCAGCATACACACactgctcagaaagcaaaacgTGAATCAGAG
GTTGCCTTGGGATGCCTTCAAAGGAAGTGGGAGACAGAGaacagaggggaaaaca
aacaaacagaagtaactGTTTCAAGATGTCCCTACAGTCATCACAGTGCCATTTTTCC
AGTACCAGAATCGTACATTTGCTTTCCAGCTGCAGTTTCCATTTGTGGTGAT
ATAGGAATGAATTATGCCCAAATAACGTAGGGGTCTCCTGCACCACCTGCAt
acactgctgctggttttgCCTTCCCTGTGATCATCATCTGTGCTGTGACTTCAGCAAAT
GTACTTAGCTCGTGTGGGGgaattaaaaatatctgcagCATAAGATAATGGAACATATT
CTATTATTGGAGACTTTTGCCTGAACACGTGTTACAAACTGTGAGTCTTTAT

TGCATTTGCTGTAAAATGCATTAGCTAAAAGGAACAAGTAGACCAATTACT
ACACGAAATCTTAATCACAGCCCCGTGCTATGCTactgatggcagcagtgccaacCAAG
AAGGTCTGTCCATTCACGGAGACAATCCCCTGACGTACCTAACTGGTAGTG
TTACCCACAGCCGTGGGTATTTAAGAAGATGGTGGAACTCCATGGGAACGT
CCCGTGCCATTCATGGTGAGCAGCAGGCGTTGCACACGCCAGGATACGCA
GCTACCTGGGCCACGCTGCTCAGCACGTGCTGCCCCTGTGTGGGACTGCTG
CGGAATAGGAAGCTGAATGCTGCAGAGCATTTGGATGAGCACACAACGGA
AAGCAAAAGCCATATAAATATGGAAAGcacaggagaaagcagcaaattAACACTACAG
AGGATTGTGTGGATCTGTCGTGTGCAGAATTCAGAAGTACGCCCAAGCCCA
TTGCTTGCTGGGAGACAGTGGGAGACAAGAACCATGTCTGTGTCTACGTGT
GTATAGATATGtgtacatatttatttaaaagagagCTAAATGAGGACCGGTCCCAAGCACA
CCGGGATCCTGAAAGAGTGTCAGACAGCAGAAaggcatttcctgtgctcaTCCCCTTG
TCTGCCCACCCTCATGCCAGAGCCCCGCTTCCTTCGCTGCTGCTCAGGcatca
gcagagcagtgtgtggTGAGGTGCAGGTCTCACAGGACacaccagctgctctgctttgcttatCGG
GGAAGTCTCCAAGCAGCAGCTAAGAGAGAAAAGGCTTTGTGTAAACCACA
GCTTTATGAAGCACGTGGCAGGGAGAGCAGCCTCACAGCCTGTGAGAGCA
CAGCACCGGAGCACAGTGAGCCAGgagccacagccctgcagccctccatTGGCACCAAA
GCAAAGAGGGAGCACACGGTCCCACTTCggtttccttctctcctcccaccATCACCAGT
GAAGTCTCTCTCAGAGCACTGTTCAGTGCCAGGCCGCCAGCTCCGGGAGC
GTGCACCAAGTGGTGCTACCCCagagcaacagcagctgggagatcTCCcgtgagcagggctggc
aggtcTGAGAGGCATCAGGGGAGCAGCAGATTTGGAACTGCAGCGGTTTGGG
GCGAAGCGGTACAAATGAGCAGCACTCCGGGGAGGTACTGCTCTCAGCAG
GTGGACTTCTCATTCTCCACAACGTTTTCTGAGACACTGCAGGAAGCTGAA
GGCTTTCCTCTCGGCCCAGTTCCTGTCTGATTCTCATGGTGAAGAGGGCAC
CTCCTAATTCACTACTGAATagtcatggaaagaaaaaagtagttCGGGAGCAGCTGACTG
CATTCAGAGCATAAAGAGAGAGCCAACCTCAGACAGTGATCTGCATCTCAG
CGTGCTTCACACACAGCCTTGCACTGTGCAGACCACATAGCGATACAATAA
AAACATCCTGACAGTTTTACCTCAAAAAGAGCACCTAAAGTACCCTTGGCT
AAAGGCAGCGCCGTGGCAATCTCAAACTCTCCATTACGTGCTGCCTTAAAT
TCCCATCTTCCAGCACATCCTTCCTATTTGCATCCACAACAAAACGCACGGC
ATGTTTGGGAGAGTctttcagctcagctgtgccTGCTCCTACTGCCTCCAAAATAGAAC
CCGAAGTAACAGAACCTAAAAAGACACAAGCCTCCATTATTGTGTCTGCAT
GCCTGTATTAGCAGATGGCCGTGCCTGAGATTACAGAACGCATAGCAAACA
TTGCCTCCCAAGAGAGGGTGCCTTTAACACCCCCGGCTGCCTCACCGTACC
CCCATGGTGAGCCCCAGCGATGTGCTCACACAGACACCATCACCCACcctctg
cccagccctgccccggTACCCACCGAAAATGAGGGACAGATGTGCAGAGCCTTCAA
CCACAGATGCATCAGAAAACAAGATAAAGGTAGATGGCGTTGGCTCCTCG
GattgttattttaaacatttccattatttttctgtattctttaaTTGATAATACATGGTCAGCACATTGTAAT
TCattctctgtgaaaaaaaacaaacctcaatACAGCCAAGAGGAGCAGGCAGATAATAGTCT
TAATTTCATTCTTACCTTTTTCACCAGGCTTTATTAAGCCACATTGCTCTCTT
TGTGTTAGCAATTTTTATGGCACGAGaggcaaaacagaacagagaaaaatcagttCTCACAA
CTCTTGCCATGCTGCAGATGTGAACTTTCTGCAGAAACACTTTCTGTTCTAT
CTGAAGTGCCTGCATGCAGAATGGATGTATGCGTTCAAACCCAGTGGGCAG
CTGTCAGTCAGTAGGAAGGAAATGGGATCTTTTGCAAAGATATTTGTAATTG

TAATTGTCAAGTTTAGCAGATAAGGAAAGTTGAGCATCATCAGATCCCCAC
ATTGCGCATGAAGACAAGATAAGTGCCTGCTCTCCGACTTCTGTTCAGCAC
GCATGCCCAAGCTCTCCCCTTTGGCTGGGTCTAAATGCATGCACCAGAGAG
ATGCTGTCCCCCTACGTGTTTAACCCCAATACCCAGATAGTCTCAGCTGGTG
CACCAGTGCTCTAGACAAACTCCTTGTTGCTGCATTAACGAACACGTTTTG
CTCCTTTCCTAAAGCACAGATTTTGGCGAATGAGTAATTCCACCACTTTATG
GCATCCACAGATTTTGTTCCAAACACTGACAGTTTTCACAGCCGTTCCCAC
TGAATTGCAATGCACTGCTCTTTAGGCAGTGTTTCCTCACCACTGAGAAGC
ACTCCCGAAATTACGCTATCTACATAAATATTGAAGGCAGCACTTTACATCT
CCATCCCTGCGTTAACCCACAGAAGGAGGCTCCTCGTGGCGGCTgtaagcagtgc
tgcagggccctGCTCCTGGTCAGCCTTCCATCTCTGACACTCAACCTCTACTCAG
AAAGAGGGCACGATGTTGGGATCAGCCTTCATTATGAATCTCTAAACAGCG
GATGTGGACAAATAGCTAATAAATGTTTGATGTCAGAATAACAAACCAGAG
ATGGCAGAAAAAAACCTGATGAGGCTTTTAACCCCCATCGGGTACGTCAGC
TACTTGAAACTCTCCAGTGGGACCAATGGCAGCTTTTATAAATCTCcattaggaa
gcatttctttcatCGTGGATGACTCGGTAATCCCAGCAGGCTCACAGAGGGTTATGG
GGACTGCAAAACTCTCTCCTACTCTGCTCTGTGTTAAACTCCTAAAATAAA
GCACTGCATCCAACAGGCTTTGCCATAGGTGGTAATAATAATGCTATTTCAG
ACCACATTCTTCTGCCAGCTATTCTGGAGCAAATCCAGATTAATTCCACCAA
ACTCAGCTTGAGCTGTTGCAGCTCTGCGGCAGCGCAGGGACAGCTTTGCG
GGCCACATGGCACAGCACCGAGTGCTGAAGGCTCCGAGCGGCACTGCCCA
GCCCCGCTCCTCCCCAGGGCAGTTGGGATACACGGTGTGCAAACAATATGct
gtaaaacaaattaaaaaacagaatttcctgCCAGCTGCCAAAGCTGCCTCTTTCGCCGCGCA
ACCACTTTGCCCACACATCGAGGCTGGGACCAGACCCTGCAGGCCGCCCC
GATGCCTCCCTGCCCACACCACGctccccaggagcagccccacagcacagcctgcttgCAGCC
TATTCATGACTGACCTGCCCTGGGACACCAAGAGAAGCGCTTTGTCCTGGA
GACTGCGGTAGGTCTCCTTGTGCTCAGATCGCCTCCTGAGCGCACACACTG
ctaaaggaagaggaaaaataccTTAATTCTCCTACCAGCACTGAAACTACCGGTGCAAA
AGGCACTAGGAAACGTCCAAGCACTTGAAATcttagaatcgtagaatgatttgggttggaagg
gacgtttAAGATCTTAATAAACAACTTTTTCAGGATCTTCAGACGGCAACTTTTC
TGTGTGAGTGCTTTCTGTttgcctgctgcagcacagcacccacccAGAAGGACCACGAGG
AGCCCAAGGAGGGGTGCAGGCAGTGGCGGACCTGAAGCgggctgggaggagaga
gaTGAGCACAGAGCCAGTGACCACGGGCACAACTGCACTCCAGCCGGCTC
TGCCAGACTCTGGCTTATTGCTTGAGACCAGAAACCCTCGCGCCATACCCC
TCCTCAGCCACAGACACCCCCTGAAGATGCCTGGGGCTCTCCCTACACCAG
TGGATGTGCCACACACTCCTTTGTGAAGCACTCATTGCTGTGCAGACTGAA
GTACGGCGTGTCATGGAAACTGCTCGGATATTTTTAGATGTGAAAATGCACC
AGTACAGTAAACTGggacacagaaaagaaaacgaAGCCGTAGCCCAGCATCATAGCA
TTCCTAACAGTGACTGTGGAACTGTATTAACTGAAGTGCCATTAACTCTGCT
CTAGCTGACATCGGAGATGGATTCCTGATGGGGCTCATCCTCTTTACAGCAG
CTTGGTGCTTCCgctgggcagcaggcagcagcctgagGAAAGGAAGCCTCACTTCATTC
AAATAAAAGGCAAAACTCTAAAACAATATTGCCTATAAAAGTTGATTTCACT
CCAGAGTCCAAGCTAGGCTGCTTGAGTGTCAAACCAGAGCTGGGTCACCC
TCAGAACGACTCTGCAAAGGACAGCCATCCATCAGCAAAGCACATTTAAA

GTGTACTTCTAAAGTGTAGGGGATAAAAGCAATTAAAGGTGTAGTCCATATT
TACTGCGTTGATTTTAACCGTACTTATTACTGTACAGGACAGCACTCATTTT
GGCAGGGTACAATTCAGATTTGGCAATGGGCTGTTAAGCACATTACAATCC
AGTGCACACAGCCAGCCATGTGCCATAGTTATGTGCTCCCATGTTCATGTGG
CTCCAGACATGAGGCCTCCTTCAGCTCCTACAGAGGTCGGGGCAATGGGA
GTCGGACACACCGGGATCAGGGATGCCAGCAAACCTGCTGCATCCCTGCA
CACCTCCTAGGGGCAGGCAGAGACAAACTCCTTTCATGTTCTTACAGCCCT
TTTTTCTCTAGCGTGGTGCATGTGGGGTACTGCTGTAGAtgttgctgcttctctttttccca
gctGTAAATATTTGGCTCCATTTTCCCTGCATCTTTTCCCCTATCTTTAATACCA
TATAAATAGAAACCCGCACAGTTCCTCCATTTACAAATACATCAAGATTAAT
TTGCATGCTGAAAGCTtaaattgaatattttaaatCGGACACGGATAAAACAGTAATCC
ACTGCCGGGCTGAATTCCAACCACGTAAACTAAACACATTTGACATCGAAG
CAATAAACAAGAGCAAAGCCAATTGTCCATTAGGATGCCCACTCCGCAAAT
AGGACCACCAAataaaagccaagaaaataacatcacaGATCCTGAGCCCCAGAGTGTCAT
TCCCTCCCAAGCTCCCTTCCTGGCTGAGATGAGCTTCCCAAATACTCAGAG
CTCCAAAGCACTCTGGGGTTGGATGTTGAAACCAGCCTAGGTGATGGAAA
GGGAACCAAAACATCTCCAATTCATTGTGATTCACCTGTAAAACAGCAGCA
CGCTGTGATTTGTTCCTTTTCCAAGCCCTTGGCAGCATCGCTTTTAGAGTCA
CAGCTGCATCTCCACAAACCCCCACCCACCCTGCAGGGCCGGGCACTGCC
GGCCGTGATGCCCACAGCGCACTGCCGCCAGCCCAGCGCCGCAGCGCGGA
CCTCCGCTGCTCCTCCGCCGCTCCCACACACGAGGCGGCGGCTCCCCGCCC
TCACTGCGCCTCCGGGGAGTCTCGCCTCGCCGGGCTGTAGTATTCAATCTG
ATGAGTCAGCCCCGAAGTTGTGTGGATGGTTTTAAAAGTCACATcgttttaaaaatg
ttaagtCTGTGGgggatgggtttttttaattcactttctGATGgttattttaagcttttttgCGCAGGC
>TCONS_01318073
TGGGAGGAGGCTTGGTTGCTGTGGTTTAGAAGACTCTCATAAAGTCTTTGT
TATAtgtaggtttgtttgtttttagccttaatttaattttatttaattgaaaGACTTATTACTTTTTTTGGATAA
AACAGTAGCCTTGCTagggagagagggggagaagagtaagaaagaaaagatggagcTAAAGTTCA
CAGAGTGCTACCTCTTTGAATGTGGCAATGAGTGTCGGGCAGGGcctgtgattttta
aaatattttgaaagacgATTAGAAAATATGCTTAATCTACTTTTTTAGCCTTTGAAACA
TTACTTCATGAGCCCATTTCCAAAAGTTCATAATCCTATAAAAGGGATATATA
ATGAAGTTTTTAAAAACCCTTGAACATCGATTGAAGTACTTTAGGATTCAAA
GAATCATATTCATATGTGTGCATAATAAAACAAGAGCCTTTATATTAAAACTA
GTTACCTGCCATGGCTTCTGGGCATTTGGCTTTTGAATTCTCCCAGCCTTCA
GTCTGATATTTGATACCTCTTGCAGCCAAAGCTCCTGGTGAGGATATGAAGG
AATTCAGGATGACAGTTTCAGTGTCTTATCCAAACAGACGTGTATTGGAAA
AGGACTAGAGAAATGGTGACAGCAGATTCAGtCTAcctgaggagaaaaagaatgcaGT
GAAACTTAAATACAACTTTTCCATAGTGCTGACCAACGCTACAGTGCTCAG
GACTATCCTGATAGTGATTCCAAATCttccaaggaagaaaatggaggGGGTG
>TCONS_01388278
gtcagggctgtgctgggggggaTGTTCAGGCTTATAGGCCTAGCTGTGCGTGAGGAAA
GATGGCCTGTGCGGTAGAGGTGTGTTTGTGAGCTTCAGACCCATAGGGGGA
CCAAGTGTGTGTGGAGGTGGCTTCGTGCCCACCACAGGTTCAGGTGTGCA
AGAGGACCTTTCGGGGCCCATCGTGAAGTGGCTCTGGGGCttgcagcagcttctgcct

tGTGGCAAAGCCTTGGTAAAGGGGGGCTGCATCCGGAggggagagctgtggggctgaa
agactgcacagctggaggaggGAATGGCCTTGGAGGAGAAGAAGCTTTAGCTGTGGC
TGAGAAGCCTGGATTaggaagagaacagagcagagagaCAGGGTAAGGGGATACAGTG
CTGTTCCTGCCACGCTTTGCAGTTTCCTATATAGAGGCAAACACTCCTTCCC
TTATAAATATAGAGACCTACACAGGCTTGTCCATTGATCTCTGAGCATGTTG
CTGACAGACAAAGCTGGAGGTCCAGAGCTTTGCATTCTGCCCGTGCCTCGT
GCAGCCACGTGCTGCTGTgtctctgctggctgtgctgctgggctgtcaTCCAGGGCCCTCATG
GTGCAGCCGTTTCAAACTAAAGGTGTGGAAACTTGAAAGCAACGTATCCC
AGGCTATGGCAGCTGTGTTCTGCTCTTCAAAGGCCAGTATTACAGGATTTCT
ggtatctatctatctatctcaTATAAGAAACATGTTGCTGTGCTCTCTATTTACAAAGATT
GAgctttgtagcttttttttctccgTTTCttacaaatggaaagaaaagcacaaagacCAGGACTTCTGTGTC
AGCTGCTTTGCAAATAGCTGATGCTGACAAAGCAGGATGCAGAAACCTTTC
TTCAGACTGAGTGAACGTATGTCAAACAGTAAGTGAGTTGTgatgagaaaaacagtt
tttatgaAAGCACGAGCCAAGAGCAACAGCTTGACAAAGAAGTTTAAACTTTC
CCTGATAGCTCTTTGGGGTGTAGATAGTTAAGTTTCTGCATTTGTAAGGGAA
ACATGGCATTCCTTAAAGCTGAACCTCCTCCCTTGGGTTTCCACTGGATGCC
TGAGAGATGACAGTATAGGGTGTCTCCAGAAGGCTAATATTAAACATGAGG
AATTGGTGTGGAGGGAGCAAGCAGCAAGTGAGATTCCAGTTTTGACCACA
GAATGAgctaaaaacattttgcatcaGCAACTCTATCCGGTTCCTTACTCATTCTCTGTA
CACAGCTCCTTCTGTTCAAAGGATTCCTCTGTGCCCTCACATTAGTGAATGC
ACCTGCCCTTTGTGTGCAAGCTTCTCCTAAAGGGTAAATTTGCCTCAGACA
AACAGAAAGTGCTGAGGCAGTGTAACGTGAGTTGAATTAACCTTGgaagggca
gccccagctcagcctTGCTGTGTTTACCCAATCACTATCCACAGCAGCACGAGCAA
GGCTGACTGGGGAGCTGATGGGTGAACTGTGTGGACACGTGGAAGAGATac
aggaaggaaatggaaaataaattggGGGTTTAGCCAGTGTGCTGCTTCTAATTCTGCAGC
TGTGTGAAGATGGGGAAGCGACTTGAGCAGTCCAAGCACGGCCCCAGCGG
TGCAGCTCCTGGGATGTAGCAGCAGCCACCACACCCTTTCCTGTTCACCCT
GTGGCCACACGCAGCCCCGTCAGCTCTTCTGCATCAGGCAGAGCCCTGCG
GAAGGATTGCTTGTCCAGATGCAGATGGCTCTGGCACCGATGTGATCGGCA
GCAAGGGGTGGTGAGTGGCTCCAACAATTACACCCCTGGTGTGCCAAGTC
CAGTGCCCCCCAATGGGACATTGAGATCTGGGAGCTGCTTGCAGTAGCACT
GATGTTTAGCCACATCTCAGCCTCATCTCCTGTAAAAGGCAATATTTACTTA
CGTAAAAAGGTTTAATGCATGCTTCGTGTGGGTGTTCCAAGTTAAAAATTG
TCAGTCAGTGCAAACAGGTCTTGGGTGAactactgaaaagcaaagtgCACGTGCTGT
GCTACCGCAGTTCTTTTCAGAACCGGGTATGACCTGGTGCCCCCTGCCTTA
ACTCCTTTTAAACAGCACTGCAGTCGCCTTGAGTTGAGTCCTTCCTTAGATA
AAAccaattctgttttatttccttcttggTCTGCTGTTGATGGTGTCTCAGTGAGTCAACAAG
CCGTGCGTGTGGGGAATGTCCAGGACCCACACCCTGGTCCTTCAGATCGTA
GCAGTGTCCTGCTGGAGGATAACCTGGCTGTGACCATTTGCAGTGGGTTTG
GATGCCTGTCTTGGCAAAGCTGCTATTGATTGGCATGGAGTAGTCTGATGA
AAGCATGGAGGAACAAGGAAGGCTTTTCTGCAcggaggaaagcagcaggaaagctgtG
CAAGCACTGGAC
>TCONS_01390676
CTTGTCTTTGTGAGtgaaaacGTTTCTGTCTTCGGTGTGCAAATGGAGATGCTG

CTGTTGGAGTTTAGCCCACGGGAGTGTCATACAGCTGCAAAGGAGAGGAT
GGACTCAAAGCATCACTGCGCAGTGCGACTGCTGAGCCCGTGGGGTGAAA
GCATGCTGGATTGGAGATGCTGGAAGAAAGCACAAGCAGTCCTTCAAGAT
GAGGTAGGAGGGATGGCAGTAACAGAAGAGCACAGCTGAATCTCAAGATC
TCAAGCAAAGACAGCAGGAGTCTGAGCATTTAAATATGCCTTCGCCATGCT
GGAGCATTCCAAGCAGTGTGTTCTTCAAGTTTGGAAAGGCCATGTAAGATT
TCAGTGCATCGGCTCAGCCTGGAGGACAAGAGGACACAGACCTCAGAGCA
AGGAGGGCCCTTACCCATCAGTGACTGCTCCATGGATGGTGGCACGGAGC
GCAGTGGAGaCAACATCTACAACTGGAAGAGCTCACAGCGCCACTGTGTTA
AAGAACAAAGAGAGCCATGAGTGTTCCCAGGCAGCAGCCGGAGTGCAGG
TAAGTGtggagcagccagcagggatGCAAGCATGCTCAGCAAAAGAGGACAAGCCT
GAAAGTAAGTCTGAAATCCTTTGGGAATTTTCCTCACCTCTTCAGAAATATG
ATCAACATTTGCCACTCTTCTGAGGCTCTAGTTCAACATTATTACTGCACAT
CACTCACCTGTACAAGGACAAATGGGTCTCTCCATGGAGAACAGATTCCCA
GGTGCTGTGCTGTAGCCTGGATGCTGAGTTCAGTGGGGCACTGCTGATTTC
CACCTTTTTCCAGCACTTTTTAAACAGCGTGTCTCCAGTAGAGCACCTCCC
AGGGTATTTTCACATGGAAACACCCCTTTCCCAGCGAACTGTCAGGGGGTaa
catgaattattttaatggcCGCATCACTTCAGCCAAAGCCTCCACCATCTGCTATACATT
CTAATTACAAATGCAGAAACCATAATTAGCAATCAAAtcacattcaaaaaaaaaaaaaa
aaagaaagaaaatgagcaatTAAAATGCACATTCTGCCAGGCAAATCCACCTCCTCAG
CCAGTGCATCCACCACATCACTCGCCTGTTACAGACAGGGGCTGAATTAGT
CCCAGGCCATTGGAGACTTCAGTCCTGCTGGTTTCCCTGACAGCAGCTTCC
AGGCTTGATCTTCTCCTCTTCCAGGGatgctctgcagctccagcctgctGTTGTATGTGTT
GGTGTCTTGCTAACGCTcggggtgatttttttttttgagggatcAGTTTACTTTTCCAATGTC
TAGACGTACCTTGTTGTTCCCTGCGCCCTGCCTCTTCTGGGGTTCTCACAGC
TTATGTGTCAAGAAAAGCTTGAGTGTTTCAGAGCAATATGCAGGGATTGAT
GGTGACACATTTGCAAGAAAACACCCCAGGCTGAATTCATCTTCAGTGCAG
CGCTGCTGCACTTAGCAACCTCATCATCCTCTCTGTaacattcattttgaaatgtgcaTTA
ACATTCAAGCGTCGTTTATACTCTGTAAGTAATCCAACCCTACAGAGGGCTT
TTCTCACATGTTCAAAACTCCAAAGTCTCCTGGAAACACAGCCATTaggaagg
gctggagcctggAGGGATATATGAAGAGGGAGGctttgctgggagctggggaagcctgtggctgctgt
gctttgtccCATGAGATGGAGGACAGACAGCTCCGCCCGACAGACGCCAATGCT
TTTAGGCAGCCCAAGAGCACATCGTGTCCAGCCCTGGGAATCAGggtcagcagc
ctgcagtggctcagagcagagggaggaaacTTTGCTTGGCTAATTCAAAGGAAAACTTTGC
TCGAGGGATTCAAAGCTCTGCCTCTGAAGCGACTGCAGCCCTTTTTCCTAT
GGCACACTCCCACTCCTGTGTGGGTGGAGAAAAACTCCTctgaggcagcactgcagt
ctCCTTCTGATcttcctgcctgcctggccCTGCTCAGAGCCATTAAAGTCTCTCTTTTTG
GCAATAGCAGGTTCCATGTGGAGCTCAGGGGACACACAGCGGGCAGTTCC
CACTGTcccctgcccacagctgtgctgtacGGCGCAGCTAGTGTGCCGGGCAGCACTGA
GGGCCTTACGTATGTACAGCTGACTCCTATTGCATCTTTTTGTCTTGAAAGTA
TATATCATCTTACCACAGGTTAAGAGCTCACAGCTAAACCTGACCCTACTGC
TCAGTGTCGGCACTCAGAGGCAAACACACAAAAGCTAATTTTCACATCTGC
TGCTTCAGCTGTaaatcatttctcattttccctctAAAAGGCTGCTCAGGTAATTCTCCCCC
AGCTGCCTCTCAGCGCTGCTCCCTGAGATCCTCATTGGCAAGGAGAGCACA

AGGCACCCAGACCTCGGAAATGTTTCAAGGCTTCACACCGGTTTTACGCTA
TCTTGCAGCGAAGCGGCAGATGGGATTCTGGACTGATCAATATCCTAAGTA
GAGCCAAGTGCCGGATCACTGATCTGAGCTGAAAGAATAAATGGTGAATCA
AAGGAATACACAAGTACCATCAGTTCTGGGAGGCAGTTCTCTCCCTGCAcca
gcccagctgctgtgccTAAGGAGCAACCCCCGTCCTGCTCACAGTTCGGGGATTATA
AAAGCCAAACAAAGGACATGCAACAAGTTATAAATTCAGAGGAGTTCTTC
CTGAGtgcccaacagcagctgaaCTGTAGTTACGGTTATTATGCAGAGACAGACATAA
AACTATAATGCGGATCAGCATGTTTTATCGCAGTCATTTGACAACATGCCAA
GTCCATGCAGCTTAGCTGTTACCTTTCTACAGCTCGCAGAGATTTACCATAG
TTTCAGCTTAATCTGGGATTTTATTATGTAGTTATTTCAATCTCACTTTGATAA
TGGCACTCCAAACGGTGATAAAACAAAGGAAGATCGTATTTAGATACAGCA
GTGATGAATAATGTTAGAAACACCTAAAGCAGAAACGGAATGCATctatctgttct
gttttgctgtacTCAGTGGAAAACATCGAGAGAGCCTTTATGAAGCATTCTATGAG
TGATAAAGACATAGCTCCTACTGGTGGCTGAGATGAGTGCTTAATTGGATGT
TCCCTGTCCTCATTACTTATCCATGTGAGCATTTCTCACCCATCACTGGTCTG
AAAGCCAGCAGAGGCCATGAGACCACTTTTTCTGAGCCCATGACTCAGAC
CAGGCACTAATCTGGCTATGCAAAGTCCTCCACACATTTTGCCCAGGTACA
GTGCTTGTTCCAGCAAGACAAACCTCCTGCATCAGGAGCTCCTACTCCCTT
ATTTTATCCTGAACAGAATCTGGGATTCTTACCTGAGACCAAACCTCAGTGC
TCCCTGCATCACCGTGGTGGGACGTGAGGCTGATGCTCGGAACACACCAC
TTTTCTCTCTGATCCTGTCGTTTAGATTCAAACAGGAGTTATTTAATCTGTTC
TTCTCCATGACCATGCAATGCTGCTGGTTcgaaaagcagatttttctaCAGCCTTGTAA
CAAGGAGATAAGAGACTGGCAGCGGATAAAACTACTTAATAGCAATTGTCT
GCTGCCACGGCTGCACAGAGCACGAAGGGCAGCAGCTCCACTGAAACAA
GAGATGAGCACGGGGTGCAGGCaggagccagccctgcagctgctctctgggGTGGTGGTA
CTGCAGGGAGAGCCGTGCAGGAGGCTCCAAGCAGAGCAGCCACGCTGAC
TGCTGAGAATCAGTTCATCCTCTTCCCCAGCACGTCTTGCTCTCTGCAGGTT
ACACCAGTAACAGACTGCCAGGGCCACAGCCTCAGGCATAAGTTACCAAT
GACAGCAGCACCCCCAAGAAGGGGgacctggagctgcagcctcccaCTCTTTGGCACA
GAAATTGGAGAGGATTTTTGCTGGGCAAACGATCCCTGCTGTATGAAAAtgc
aacatttatttcaaaagcatgaaataaagaGTTTTACTTAAAATCAAGCCATAAATCTGTGGGC
ATGCTGCAAGAGAATGAAAGCTCTTATCAATATTTATACTTACCTCCTCAAA
GAACACAGGAGCTCATTTACTAAATATCTTTGAGAAATCAAGCCTTCAGCC
AGACACAGGCCAGAGGAAGCATTTTTGCCAGGCAGGATGCTTCAGGAATC
ATCTTAACAAGGAAAAGCTCCACAGGAGAGCACCGTTCTCAAAGCTGCCC
ACTACTGCTTCACACGTGATGCACCAAGTGCAGGACACCAGGCCTGGGAG
AGGATGCACCAGCAGGGGATCTGTCTGCAGATCCACTCCAGCTCCTGTTGG
CTTCAGTGAGGCTTGGAGCCACCCCATGGAAATGTCAGATATCATTAATACA
TCAAACACAACATCAATATACACTGGCCCTTGGATACAGCCAAACACAGCT
AATGGAAGCCACTCCATAAGGCTCTATGGTGCATAATCAGCAGCCATCCCCA
TGGCCTGCCCACAACCCCACAGGGCTGGCAGTTGGGTCACGTACTGCATC
AACAGGTAGAGACCTGGCTCTCCATCTCTTCTAGATCATGAGACCGAAGGC
CTTCCAGGTGGGAGCTGCAGTTT
>TCONS_01394809

CTTCAGGTATAGGAAATGGAGAAGTCCCTCCATGGTGCAGTTTGACCGTAG
TTTTGGGAACCAGTGAAAGATACTGTCCCATGAGAGAAGAACTGCTCAGA
GCAGTGGTTTTCATTATTTGTATCCTGTCCATGGTCTGAGAGGTTGACAGGA
gtcagaatgaggaaaaaacCCTTGGCTGCAATGAATTGTTATCATCTCATTCACTCTAC
CCGTTCCTCTTGCTACACGCCATGAAGGGATTGCTCTACAAATACTGATAGT
GAGTCTTATCTTGTAATTATTATACCTAAACTCCATCTAAAGCCTGTTCCAGC
TGTCTGTAACAGCCTGAGTCCAACCTTtgtgcagaaaaagcaaaatgtttccaGCATTTCA
ATTAATTTCTCCTGTCACAAAGTGTTTTATAGGCTGTCACTGCTTGCTGGCT
GATGAATCTCCGAAGCTGAGCTCAAGTAACCAGTCACTTACTTGTACAAGT
GGCcacattttctgcagcatttctgggACTCAAAACAAACAGTCCTTATTTACGTTATGTGT
ATTTAGTAGTCTAATTGTAATGGATGTAGTTTAATTTATGCAAATTGTCATGG
TCTAATGCATCTTTGTCTCAACTGGAAAATTGGATGTGGAGAAAATTACAG
ACATTTTGGCTGTATAGAGCCTTCCCAGGATCAGTAGGTCTCTGTGAGGCA
GTTCTTCTGAAAAAGGCAATTGCAAAGAAGGAGCTTGTTTGACATGTGAA
ATTAGGGAGAACAGAGTGAAGAACAGGTGGATGTACAGTATCAGGATATG
AAGGCAGTGAAAAACAAGATGTTgaggagagatggagaaaattAGAGGCCCTCAGTG
AGAAGGGCAGTGGGCCTGCTAACACTGACAGTGTGGTGGGTGGTCACAGT
GCTGGTGACAGGACCCCGCTGGAGCAGTGTACAGTCACAGCAGTTGTTGG
CTGTGGTCAGGCCACCAACCAGCATGGTTAGTCCATCCATCTGAGTACtgggt
attttgtttttactcATAACAGGAAAGAAAGTGTTTGCTTCATCCACAAGTTTCATAG
CAGCcattcttctgtttcctccaTCAACTGGTTGCTGCTATTGTCCTGATTCCCAAGGCA
GCTCTCATAGCTACGGCAGTCTCTGTTTAAGGGTGTTAAGTTGTGGCAGTAA
TAATGCTGGCTCTAGTGAGAATGCAAACGTGTAGTTTGTTGATCCTGGACT
GCAGAGCAGTACTACTGCCTGGCAACTACACCACAAGAGCTGGCGGGCAG
AGCTGTTTGCAGCAAACTCCTCTGAAAGAGGAAAGGGTGTTttcaaatttcttctttcc
ctccctgtCCTCCAAGTTAGAGCCTGCCAGGAAGCTCtggagagaagctgtggtgcttGAGG
CTAACTACAGCTGTCTGAGCTCTTGCTCAGACATGGACCCCCAGCAAACTT
TGCTGTCTCTGCAGTAGGTGTGCATCACTGAAGGCTGAGGATGGTACTGAA
TCACTGGACATGTCCCCTTTACAAGCAAATCTGAGTGCATACATCAAATGCA
TGTTTTAGGTGCCAACTTTTCATTGAAACCTGGTCCCATTTCAATCTCTCCT
GAATATTCGGTGTTCAGAGGCTGGGCTGAATCTGAGTGAAAATGTACCAGG
GAACTTAACTGGGGTTGTTCACCAGCAGAAAATTGATGCCAGCAAAttagagg
aaaaacaagcacTGCTTGATCTGTTCTTCCCCAACAGCTATGTTAATTCATGCATCT
TGTTTGCTCTGTTTTACAAGGATACACATGGTACTCCAAACAGCTGTAAAA
AATGACGTGGACAGAAGCTGTGGAGGACTAGCAGCCCAAACACAAAGAGg
cATTCCAGGACCAGATATCAATAAAGGACCCGGCATTCAGTGTGCTGTGCA
CCAACTCTCTCAAAGCATTTGAGGCAAAGaagaggaggaggtgacAGGCATGAGA
AAGTCTGGATGATCAAACCAGCAAGAAAGCCACTTCCATTCTTTTATGAAG
AGACATCCTGGAGAGACTTGCTGTACTCAAACATTCCTGCAATGGCAAGTA
CTGTATATCAGAGGAGACTTGTGGTATACTGTCACTGCAGTGGAGTATTTTG
GTGAgtgggttgttgtttttagtttttggGGCAGaatgtttttcagtttttcagggATGTTATCCCACCCTT
GTACTTGGTAGCACATGAACTGGGATTTGATAGTAATATAGGCAGTAGTAAA
GCCTGAGAGATGCTGAAGTGCTAGACTTGACACTGGAGGACAAGAGTGAA
ATCACTATGGCTGCCTCTCGGACTTTAAGCAAAAGGCACCTTTCAGTGAAG

TCCATTGGAATTTGAAGCCAGACATTGCAGTATCCACGTAACTGCTCATCCC
CctgcttccttctctgtctCCATCAGGTTCCATTGATGTGCCTGTTGAcccacctcttcctcctcact
ACTGTTTGTAGTTCAAATGCAGACTTTGTTCTGCATGTCTTGTGGAAGCCTG
GGTACCAATGTAGTTAGGCATGGGTCTATGGGTCTAGATGCAGTTATGTGTG
GGTCTGGCCATCCCTGGGAGATGGTCCTTCTTTAGTGTGACTCCTGTGGGAT
TTTATTTAGAGCCTTACTAATCTTGTACACTTTGGAAAGTTTCCAGACTCTTT
CTGCCCCTTGCAAACTTGTAGGCTAATGTGCATATTGCTTTAATTTGTTCTTC
AAATGAAACCCAATTATGCCTGTAGGTGCCTGGCTGTTAAGTCAGTAATTTA
CTTCACGGTAGCTTagcatgttttttaaatgcctgtTTTGCTATGAGGTTTCTGTTCATTGA
AGG
>TCONS_01403014
CTTCCTGAGTGACCAGAGCAGCAACAGTGCGGCTGCGAGGTGATGGCCCC
CAGATCCCTGACATGGTCACACCTGTACATAAGCTGACATCTATGCCATACT
GTCCAAATGAGTTGGCAACGGATAACAGTCACCTCGAGGTTCTTAGGGGGT
CTCTGGTTATCTGCCCTCGGTGTCATGAGCTGATTCCCAGCACACAACACC
CGCTTTGAGGGCATTTCCTTCACCTGGCATGGCTTCTCCTGGATCCCCTTGT
GCCGTTCCTGGTCCAGCAGGTCTGGATGCTGAGCGTGTGGGACAGTTCCTA
TTTCCCTGTGTGAGTTTTCGTGGTGGGTTTCCCCTCAAAACCCATTCTGCAA
AGTGTTTGCTGTTTCCTTCCGTCCGCTCCTTGCAGACGCCTATTTTAGAGGC
TGCCTTTGGAAGCTGCGATCGTGGCTGTCCTTCACACGAGGTCAGGTTAAT
TTGGGACTCATCCCTGGCATGTCGCTGATTTACAGATCTGTCTCGACGAGCC
TATTTTGGGTAAATTCACCTCACCTCCCCCTCAGCTCTTGCGAAATTACCAA
TATTTCACCTTTCTcttgtttaaataaatgaaac
>TCONS_01416781
ctgctttaaaaaaaaaaaaagccatataTATTTGGGATGGAATTGTAAACACTTTTTAGAAA
GTTCATAAAATGATTGTATAATTCTTTCCGCTGCACGAGGCGAAGTGTTACA
AGGTTGTGTTAATGAtctcaggaaaatatttatagtgCTTTTTCCAGCATAAGAgttctctcttcc
cctctcaTTACATTTTATGATCAACCTTCACTTCACTCAATTACTTCAACAAGCA
GGAAAGAACCTTCCGTATCCACAGATTTTAAAGTGATGCATTTCACATAGA
AATGTTTGAAGTGCAGCATGGTAAATGCAAAACACGGAAGGAATAGCAAG
CAGTAATGACCCATTGTGAAGTACAGCATGCTCCAGCTCCTTGCCATGAGA
ATTCAGattgtgtttgctttcaggTCTAGTTTTCAGCTGTGGAAGTTATTTTCCTTAAGG
AGATCTGAGATGGTGATGTCACTACCCATGAAATAACAAATGCCCAATTTTC
CTTACTGGCCTCTGAACATGAAGCACATATCCCAGCGTTCCCTGACTCACTC
CTGCAGATACCTGAGCGGTCTCATCAGCCTACACGGAATGAGAATGAAGCA
GAGAAATCCTTCAGGACAAAGGATGCAGTATAAATGTAAAGTAtcagtggaacag
gttgtctGTGGGTGACTTTCAGTGTGCTGatccagttctgttttcttttaaatatgctCTAAAAACA
CGGCAAGCTTCAAAGCATGGAATAAGGGAGGTGGGTACAGCATTTTATAAA
TACTCTAAAAAATTCCAAGTTTCTGAATGTTGCAGTCCACGGGATGCTGCCT
AGGAGAAACACAGCGTTAAGATGGTGGTTGAGATGCAAATGCAACATTTTC
ACATGGTGGATAATTTTTAGATCTTCAACTTTTAGggttccaaattaaaaaaaatcactttgtg
CTTTTGGTAATTTGTCCTTAATGGCTCTGAGATTGATGTGCTGACTCCAGAG
AAATGCCCAAACTTTAGCTTATTTGCCAGAAGGTAACAGCGTCAGCCACAT
GAAGTGTCTCCTTCGCGTTGTACAAACCTTAAAGTTTTGACTGATATTTCAA

GAAACAGTAATCCATTTATAAAATTGCattcaaatatttaattgcaGAAAACCTTAAATC
AAGATTTTTCTCAGTATAAAACAGTTGTAAAATTTATCATGAGGATATTATGA
GATTTAAGTCCTCTATTAGTTCCCTTAGGTGGTTATTCTTTGTATAAGATGGA
TGCCTTTATACTTTTCTTGTTGCCTCCTAAGTGCCAGGCATATTTTCTATTCCA
TTCCAGGGGAAAGAAGCAGGAATAAGGAGAACAAGGAAGaagcagaataaagaaa
atcGGTAAGATCCCATTTTCCATGCTCCTATCAACTTCACTTTTTCTGGTATTA
GCAAAACGTGCTGCATTTGGCTTTATCCTGTTTCAGAAGTTGACCTAATTAA
TCACTTACTGCAACAAAATttagaagctgaaaaatgaagtatCATTCTGTAATGACAGAG
AGGACTGAAGCAGTGCAATAGGCGTTGGCTTTTGTAGAACCACCTGCAGG
AAAAGAACTCCAGCTCCTGGCATTTAGCAAATGCTGGTTGTGTGTATCCTG
ATTTGCAGAAATGTAATTGTCTCAAAATGATGCGGAATGGAGATACGATAAA
AAGGTTAAAATACACTTCTTATCCTTATAACAAATATAAACAATGTTGTGAA
ATACCAGGTAATGTGTGATGTGGTATATTAAATGTTATAAATATTCCAGCATG
AGACTGGGCAAGACAGCCTCTCACAGTtgtctaataaaaaaaaatcaaactgtagTGGATTT
TGGTAGTGCTGAGAAACACTCACACAAACACTTAATTCTtgtaaattaaatgcttttaaa
ggaaCATTGATTTGCATTGGCCAAAAGTTGTTAATTTTAAGATAATTAtagttgttcttt
ttctctctgtcataCAAATCTGATCCTGGCCCTTTAATTTCTACTTGTTCCATCAGGAA
GAAGAGCTAAATCCAGCAGACCTTACTTTGGCAAAactcccactgaagtcagtgaggGT
TTTGTGAGAGTATGGACTGCATGATTTGTCTCACAGTTCCTGCTGATTATTT
GGAGTGTGATTGCTCCCCGCCTCAAGTACCAGAGCTCCTCACTGAGTCTGA
CATTCCCACAGCACATGGCTGACAGCACTTTCTCTCTGTAAATTGGGAGCT
TGAAAAGCAGTGTAATGCAATAGTGAAAAATACCCTTTTCACCCAGTGACC
TTCAAAGCAACCGACTGCCCTGGGAGTTCTGAGACTGCCACTGCAGAACC
AACACTACAGAAACACTTGAGAATTTAAAGagtgtattttaaattcatgcTGAGAATTTA
AAGAACTCTTTAAACAATCTGAAAATGGGTCAAATTCTGATTGTTAGAAAA
CAACGCATCGATTGGGCGTTGCCCACAAAGGTGATACCTTATGATGAAGTG
GTGATTCACGTAGTATTTCCTCACTGCAACACTGTTGTGTCCCTGcaaattttaattc
tgtttcccTTGCAGATTACGATAGAGAAAGGCAGCTGAAGAGTTAACAGCATCC
CGTCTGCAGGATACCTACTGCTACAAAAGATAACACAGGGCAAACATAGAG
AACAAGCTCTTCATCTTTTTCACTTAAATCTGAGCAAATGCAGCACCAATG
AGAGAAAGCAGATCTGCATGCATCCATGCGGTCCTCATCCCCAAACAAGAT
GATGGAGTAAGTTTCCCACACTCTGCTGTGTCAATGTGTCATGACCTTTCTC
CCAAGGTGACACCTTTTGGGTAACAGCACTCAGTCTCTGCTGTCTTTCAGT
CATGTTTGTCTGCCTTCTTGTGGAAGCTGACTCCAGGCTCACCAGACGCTG
CCAGAGTGGTGATTTAGGAATTCAGAGTGGTCCTGCCAAATTGCTCTGCTT
ACATCACTTGCTGAGGACAGCAGCCAGGCCCATGTGGAGCTTTCCTGTGGC
TCTAGGGATAGTTGGTGTTAATATGACTCAGTAATGCAAAGCTGCACAATGC
CACCTGGAAAACTTCATCCAGTTCTGGACGAAGACAAGGAGAACCCTTTT
GAAGCAAAGTAATCCGTTGCTCGTAAGTAACTGCTCCAATCAGTGTTCTAA
CTAGAAGATATTTGGTAAGTGCAGAGACCCCACATTTAGCTATGcgcatttttattta
tttgggcCTTTGCCAAAGGAAGGCAGCTATTTTTCCATATAGTATGTTTAATCTTA
TTAATTAACTAGCTAGATTATTAttaataaagtgaaaaaaattagGAATAAGCTTTTCCTTA
CAATTTAGGAAGTAATAGTACAACTGCAAAACTCAGTCAAAGATTTCTGAA
CGCTCCCTTAAAATCACAAACCACATGGAAAAGCCACTGAGCAGAAACCA

TTAAGGCTGCATTTATTAAATGTCCACTTATATAAAAGCTAAAAATAGCTTTC
GAgtttttccaattttcttttcataacagTCATTATGATTTTCTGCTTCATCTCAGAAAgtaattttgct
gctgctgtagtgTATAATCTATGTGCCCTTTCTCGGTGCTGTACAAATGACAGAGA
GCTAATCAGATCAGAGCAACAATCACGTCATCACTGAGTAACTAGCAATAA
ACTGTTTTTacaaggaagaaagatgaactATTTTTAGGGtaactgaacagaaaaacagaaaatggttAT
ACTCAGTCTTACTCTACATCATTGCTATGAATTTCaaaagcattactttttttcctgggcAGC
AGACTAATAGCTGCAATAAATCTTTGTATAAATGGTCTAGTCAAATAGGGGT
TTTAGGGATTTGTTATAAAATGTTGAGGTATAAAAAATGCCCTTTCTCCCTGT
CCTGTCAGCTTTGATCCGCAGGCAGTCCTCCAGCTCTGATCACTAAGTAAC
CTAAATGCCCAGGGAAGAGGCTGCTGGTGGCAATGCAGTACCTTCTCCCCA
CTTAACTAACTGCCAGTGTCCTACACAACAGCCTTCCCTCCATGCTCACTGA
TAATGTGCGTAGCAGAGAATGTGCTGCTTCTCCTTGTCCTGGGAAAGATCA
ATGTGCAGCTGTTGAGAAAGACTATCTTCAGCTATTCAGCAGCGTGGGAGG
GCTTGCATGAGGATGTCCTGAGTATCTACGGACATGCTCCTTAGGCGTACAG
TGGGATTCAAAACCTTTGGGGgcatctgaaagaaatgtaattaaatagCCCTGAGCTTTTCA
AACCACTTTCTCCTGTGGCCCATCCAGAAACTTGTCAGTAAGAATTTACTTT
CTATTACTAATTTCACCTATGACATATGGAAAATAACACCATTCAGTCAAAAT
GTAGTCATTGGATTTTATTTGAGCTCTATTAGCAAATATACTTAACGTAAAGA
CAAATTAGGAACTATACAGTTACCATACCCTCCTTTGATAGAGAAAATAGCA
GATCAATTAATTAGGTGTCAGAAAAGGCTAAATCATTTATTGTGTCCAAATT
ACATCTGAAAAAAActatcagtgaaaaaaatccattcttaTTTaggcaggaaaaagaacaaaattcaaTA
AGCCAAGAGACTACAGAAGAACAGCAGGAGCTCTCGTCACCCAGACTTAT
CTCTCTGCTTCTGGTCTTTTGATTGTGACTAAGAGGTGCTCATCAGAAATGA
TGAAGCGTGAGGGACAGTTTCAAGCCTCATCTGCATTCCTCTTACCCTGGG
TTAGCTGTAGGTCTCATGATGCAATTTTAAGGGCCGTGACAGTTGCCTGTGG
CCCACAGGTCAAAGCCTGACTACAAGGGAGCCTTGCATATCTGTTCTGCAT
ACCCCACCATACGCAGGAACCACTGTGCCATATTTACATCCACAAATAGCCC
TCCATCCCTACCTCTTTGGGTAGCACAACCACTTTGAAACCATACTGCATAA
GCAGAACAGCAATGTATCTTCAATGGAGAAAGGTTTTAGTGGACCACAGCT
AGCACTTTCACATAGACAGCTAAGAAAACTTAGAAAAAGGTATATGTTTCT
ATATGCAAAAAATATAGATTagttaaaaacaacaagagATAGTGGAACGAGCTCAGAA
TCCAAGTAATTGTTCTCAGAACCTAGTCCTGGCGCTATTGCAATGAACTGTC
TCCTGGAAGCACAATCAGTTCAATTGAACCATGCAAAGAAAGTATGAGGA
GGAGTGTGCAAGCAACGTCTGTGCAGCTCAGAGTGAAAATGGCTCCATTC
TTGCCAGTCACACTAGATGAATAAAGACAATGGGAAAAGAGAGGGCCAAG
CCAGGAGGTCCACAATTATACAATACCAAGTACCAGACCTTGCAAAAATAT
AATAACCTTAGTGATACGGAATAAGGAGCAGAGCTATTACGTAAGTTTTCTG
TGACCCTGGAaaaggatttgttttcctttgatctCTCTGCAGATGCATGCCATTCCCAGCCT
TCTAAAACAAAGATTTATGATGAGGTTCACCCTAGCACAGATCTCATAACA
GATCTTCCCATCTCTGTTTTGTTGTCACTTAAACACTGGAGGAACAACTTCA
TATTTTAGGTGCACTTTATCCATCAGAAACAGATAAGGTACAACAAATTCatat
aaaaatgatgaaagcaaCAATGTTTAGAAAGAAGTTACCTTTGAGAAGCCAACCCAC
AGTCTTCTTACTTGATGTCAGTATAAAGAAGGAAGAGTTCACAGTACAAGC
CTATCTTTAC

>TCONS_01435976
AAGACGAGCAACTTGCAGGTTTGTCACAGTAGTGCCTGTGAGGACCGCGC
CAGGACGAGGTGCTGTAACAGCAGTGAGTTGTTGagtttgctgttgctgctgcacTTGT
ACttgtgctgcttgctgttgtGCCCCTTGCTGTGTTTGCtgcgctgcctgctgctgaggaagctgtagcttct
gcttttgcaacttgatcagctgctcctgcaaaaacacaacactttcttcatttcctaTCTTTTACAGGGctacaggaa
aaagaatggaagtgGAAGCAGCTTTGAAGAAACATTCTATGCCAAACTTCAGTTAA
GATCAAAGAGCTAAGGAACAACTCCTTTACCCAACAGGCTGCCAGTGGGT
AAGCAACAGCTAAGCAATTCTCTACTGCAACACTTTGTCCAGAAATACCAA
CAAAAAAAGGTAACCTGAGCACCAACAATCAGCAAGTTCGGAATCATATCA
TTGTTTAATTGTGCAGCCAACAAAGCCAGACTTTCAATACTGTGGGGCTAC
AGCGGGTCAAGCAGACTTCCAACCCCAGGAAACAACCCCTGAGCATACAA
TTATTACCTGGTGTAACTTGAGTCACCAGTGATCTTGTCTGGGTCTGCACGG
GTGTTAAGTTTGTCGTTACCACAGCTGATGCTGTCACCGATGTTACTGCACG
AACCCCCTGCGTAGATGCTATGGTCACCAGCTCTGTGGAGGCCGCAGGTGC
TGCCGCCGCTCGCTGCTGGGTATGCACCActtgtgcagctgcagtgcctccCGAAGTCA
GTCTGTATACTTGTCCCCGTGACTGCTGTTTTGATTGTGCCTGCCTATGGGA
GAAGACAATTGAgataacttcagaaaacaataaaagaaaactgactGTAGTTCTTATACTGCTA
CGCTGTTTAACAGAGTGCTGTATTCCAGAAATGACAACTCAAGAAAGAGA
CAATAAAATGCAGAGATAGTTCTaaaatttgctttcattttgcataTCAGTAGTAACTCCTG
GATAGGTTAGGATTCTGGTATCCAATTGCTCATCAACATGGCAGTCAGCTGT
TGGTAAGTATCAGACATTTTACATCCTTGTCAGCAGAAGGGAGCCAGGTATT
ACTCTGGCACACGCATGTAACCTCTCACACATTAACATCATTGAGTGCTAGG
AGATGCACAGCAGTGTATCAGGACAGAGCCCTTTCTCTGTCTCTAGAAAAG
CTGTACTATTCTGGCCCTTCAATGAGCATTAAAATCACTGCTGCCTGAGCAC
ATTAGAAAATAAACTGGACTGGCTCTAAAACACAAGAGCTTTCAACCTATT
ATCAGATTTACTGATACAGACTCTACATTTCATGGCATGTAAAGCCATCAAA
GCTTTACAATAATACAACTAATTGTAAGTCCTGTAGGATATAAGATTCTACTT
TCCCAGCAAAGATGAATTTACTCAAAGCTTGGgtttttaatctattttaacAGTAGCTGC
TACAGCTACAGTCTCTTTTCTGAGTTCTTTGGATAAACAGTTAAATACTAAA
CCGAAGATGCAAGATAATCTGTACAAAGAATACAGCATCCTGAGGTGGAAA
AATGCTCACACCTATTATTGCAATAGTGATATTAACAGAGTTTTAATcagtgaataa
aaaaagaaggtgCCCAGCACActcaggagagagagagagaaagaagaagaaagaaatagccTTTATTT
CACTACACTGACCTAAAGTTCTTACTTTCCAAAGCAAGTAAAAGCACGCAG
ATGTAGtacacagaaaagaagaactgaAGTTACTACATCAGACACCGAGTCCTCCCTTT
CTTACCAATACTGCTGTGTTGGCCACAGTAGTTACTGCAGTCTGCACTACTG
CCTGTGGCTGCTGAACTACCTGTGCCTGAGCCTGTGGCTGTGGTTGGGCCT
GTGCTTGCTGGACAGCTGGCTGCTGAGCTTGCTGCTGGCcggcttgctgctgctgaga
ctGCGGGCCTGCCTGTTGCTGTGCCCTCTGCTGTTCAGCCAAAGCCTTTCTG
CGAGAACAGATGCATGCTTTGGATTCTTCTGGAAAGGATTCATGCCAAGTct
gaaaatgagcagaattttattactgaagacagaagaaaaaattctaaGAGCATTTAATGTCATGTTTTAG
TGGATAACACTGTGGTTTTGGTGAAGTTTTCATGATATTCCCATCAGCACTG
GAGCCTGCATAAAGACAGACATTTTAAGGGATGTGGAGTAAATGATAAAAG
AACACATGTAAGTTACTGTTGTAACTtgtgcacaaaaaaaaaccatacaacTAAACAGCA
CAGACATCAGAATGTTTGCAACACAACTAAATGGGAAGCCTAGTTTATAAA

GGAAAtcttaggaaaataaaactctttATCACGTATGTGAGCAAACTCATGCAGCGAGTA
AAAGCTAACCCAACCACATGAGATGTGAAGACATTCCAGTAAGAAGCCAG
AACACAAGGAAAGAGgtcaagaacagagaaatagaaGAGCAGattgaaacagatg
>TCONS_01443812
agaaaccacaaaaaggagcaaaacaaaactaactAGGATGTGTATATTCAGTCCTCCATTTCTG
GACTAGCGATGGTAACAGCTACTGCATCTGAAAGAACAGCAGATTTGCAAG
GAGTTGCCAGAATCTGATGGCAGCGTATGGTTGCTCTCTTTGAGGGGTGGA
ATATCTTCTACAAGCTGGAGTCTCAGGGCAAACTTGGCAGAGGCATTATGC
CTGGTAGAGCCATGCTTCCCGTGGGACCTGGCTGAAGCATGGCTTCTCTGA
GCTTGTGCCAACAGGGGCTGCGAGTTAAAGCCACTATCTGGGGCCTATAAT
CTCCAGCAGGAGGGGAAAATCTTCTGGTATGTCTGAGAGAACTCATAGGTT
TACAGCAGCATATACTGGAATTCAAGATGCTTATTTTTAGATTATTATAGCGT
TTCTAAATTTATCAGGTGAACTCCTCCAGTTCAAATGCCAGTGAGAAAATTC
CTAACTTCAGTCTCAGATTCTGAATATCCTTCAGCTtaacacaaaagaaatacaCTAAT
ATGGTTACCGAATTGCCTTTCGTCTCTCCTGTGGATCTGGGGAGACGTAcgtct
tcatttcttctttaacaCGCTGTAGCTGTACTTCTAGATTCTGTTCCAGTGTGGAGCGAT
TGCTCTCCAACCCTGCTGCACAACTGTcatactggattttttttcatcacattcTTGAGTTAA
TTCCTTATGTGAAAAGGGCAGAAGGGTACATAAATGAAATCTTCAATTGAC
TGACATGTGACATTCAAGCAGGCTCTTGTTGGTCTCACACATCAACCTCAG
CTACTGGCACTCTCCCTGACACTTATGTAAACCCAGCAGGCAGGTGGGTACt
gttacacacacacagtaaGGCTGCATAGTGGTGAACAAGCTGTATACACCACAACAC
AGCATGCAAGTTTGCTCTTTACAGCCACAGCTTTAACATCATAGATCATTAG
GGCAGCAATTgcaagaaaagtgaaaaataattacaaagttGGAGATGAAGGGATTCATGTAC
TTACTACCTAtggaaaaacagctttgctttttagtTGTACGCAACAAATGGCTTAGCCGGAT
TTCACTTGCTGACACTTTAAATAGaaccattaaaaataagttttctggACATTTAGTTGATA
GATAAAGGGATAGCTCAATTTACTTCTGTGTCAATGAGCCAACTTACAGCA
GCTGTGGACCTGAATGACTTCTTCAGCTGTCTGTaaaattttgaaaacagatgGATAAA
TCTGTCATTCCAACGACTAATATAGGACTGAGATCAATGTACATAAGCATTG
GcatgaaattcatttctgatctatttcaaaaaaaaaagagttatttgAGATCGCTAGTACTGAGAAGCACA
AAAAGCTTTGAAACTAATTTGAAAGTTAACTTTGCTGTGATTTTGAACCAcg
atttttattatttatcattATAGGTCTTccctgttttgatttttaattactaCAGAGGACAAAATCTCAGTG
TGATTTAGCTTTTAGAAATTCCAGTCTTTTATTTGTTACTTGTTGAAAATCAT
TCCCGACTTATTCTGCTATTTCCAGCTTTCCTCCAGAATTGATTACATCATCA
CACAGCACAACAGAGCTTCGTGAGTTATGCTCATTTGTGACTTTGACCTTA
ATTTTGAAGAATGAGAGAAAGGtttaatacattaaaataaacctACTTCGggactgaaaagaaata
caacatgAAACAAAGCATGCAATTAAATAAAGTTGATGAGACTCTGGAGATTT
GCAGTATTTGAAACTTTCAGGTGTCTGGTAAATGAAATCAAACTTAAACTA
ACCATATTCTGACTCATTTTTGCACTGTCTCAACTCCTGCACTGTACATCCTT
GACCACAAAGGTGCAAATAACTCAACGGCAATGCGTAAAGATGCATGCAC
CTAAAGCCTATAGGTACAATAAACTCCTGCTCTAACTATAGCACTTGACTGC
ATGGAGAAATGTGCAGAGCAAAACATAGGACTGCCCCCCAACgtctgcctgctgcc
tgccctaTTTCATCCATATGCACTGTTCAGAGTGTGTTGAAGTGGTTCTACCAAC
CTGGCACTTTTGACGCAGCTGTCTCAGTTCTTTAATGACAGGAGCTAGGCT
TGACTTCTTCTCTGCCACCATAGCATTTAGTTTCTTTAC

>TCONS_01455276
aagaagacaaaaactGAACGCGGAGTGCAAGGCTGTTCAGCAGCCATCAGAAGTC
ACTGCAGATCTCCCATCTGACCGCCGTTCAGCGAGAGCGCCTCAGCAGAG
ATGGAGGAGGCACAGCGTGAAGGAAGGCAAACATAGCGGAGTGCACTTCT
TCCTTATAAACAGTCTGTGGGAGTCCAGCGATTTACATGTGAAGAGAGCTG
CCAAAATATCCTGTCCTTCCCCCAGCTCTTCACAATGGTAATTCCTATTACG
GTTATTACTAATCCAAAGATCATGAATAATGACAACGAATAGTAGAGGTGAA
TGCTGAGATGTGATTAACGAGTAACGATACAAAAACGGGGAGGTGGGCAG
GAGGGAAG
>TCONS_01464392
CGTTTGTAGAGCGTTTATACATCGTTTTTACTTTGGAGTGTTCTAGGGTGCC
CAGAAGTTGAGCGGGGAGCCCTTAGAGATCCCTTGGGAAGACTAATGGGG
GCCCTAGAGCATAGGAGTGGACTGTCTTGTTGGCATCTGATTGGCTGGGAG
GCGGTTCCCCTGatgtaattaatttaattacCCAATAGTGCTCTTCAACATTCTGAGAAtg
gtgagaaagaaaatagataaCTCTCCAGGTCTTGCAGCATGATGAGGAGCTCTTCATC
AGTTCCCTGGCATCTCTGACCACTTCACAGAGTTCCCAACTGTGATGTTACT
TCAGCTGCCAGAGCACACCTCAGTATCTACAAAAGGCTGGAACAGTTAAG
AAGCAGCCAGGCAGCTGCACAGACTTCCTTCCCACAACTATTCCCTAGCCT
GTGATCAGTAATTGTTCCCACAGTTAAGACTGATTCTATGCTACTTTGGTCA
CCTCACTTCCTCTGGCTTCATTTCATTATTAAGATGTCATGTAGCCCTAAAGG
ACAGTCAGTTTTCACTTAGATCTCCAGGTAACTCAAGCCCTCTGTAGATGA
CAATCCTTAGAAACTGCGACACAGACTATAATTCTAAAAATGCCTCCTTTAC
TTGGCATGACAAGGCACACACCCAGCACAGAAGGAGGAACGAGGAGTCC
ATTCACATAACTGCTCTCTCTGCCAAGCAACTATCTGTCAGTGTGCCTgagtgc
tgtgtgctcagcatgCTGTCACCAGAAACATGCCTCAGCCATCTGAAAGCCATGACT
GCTGTACAGTAACTGTTGAGTTgcagcctgaaccactgattgagcacctgggaaaaagacccagtca
gccctgggagcacaggtgaaggcaattcagctgtgtggaggctggctgcacctctcttagatgCCTTTtagggctgac
tgccactagggAAGAATCTCTGGTTGGAGTTTCCTCCTTTGTGGATTCTTTCCTGT
GAGTCTAGATCCTTGGAGATGGGTGAGCacctttacttttcctttgaaataccaTCCTATCTG
TGATTGTCTCTTTGCTGTTACACAGCTGTATTGCCCTTCTGCCGTGTTGATCT
TCCCCGTGGTGACAACTGTCCATCCTCTGCCTTGTTCTCAGCCCTGTCAGTA
CTACTCCACTAATGTTCCTTGACTAAATACAGCCCTAGTCTTTTCTCGACAG
AGAACAATGCCACAGCATGCAAAAGTTGTGGTTGCCGTCAGACTGCTCAG
AAAACAGCGTCTAGCCACAATGAAAAATACACGGGATGAAAAATACAGCC
TCCAAAAGAATTAGATGCTGAGCGACTAAGCTACTGACACGCTGACTTTGC
CCAGCACAATGACGAAAGCAGGACTCTTTCCCTTGCTCAAGCTGCCTAACA
CACAACGTTAGCGCTCCCTTTGCGGCCGGCGCCACAGACCCGCGGGCTCT
AGGACCCGGGCTCAGCAGCCTGCGCTATGGTGCGCGGCGGAAAGGCCCTG
GGCTGCCAAGCGCCACACCGCCGCGCGGCCCCCGCCGGTGGTCTCCGTGG
CCGCAACGCTCGCCTGGCCCGCCCGAGCAGGGGAGCGCTGGGGCGCCGC
GGTGGAAGTTTCTCCTGATGCCGTTCTCAGATGCGCCGGGCAGCTTCACCG
CCAGTTGCTGCTGGTGCCCGGCGCCTCTGAGAGTCCTCTGGTAACTACCTG
TCTTGGGACCGTCCGAGAGCAGCGGCTGCCGTTCCGGTAGCGCGGGCTGT
GGGCGGGCCGCGGGCGCGGCGCGGCTGGGCGGCCCTGTGGTGGAGCTGT

CTGAGGGCGGCCAGGCAGGCGGGTGACGGCAGTTCTTGCCCGAAAGGTG
CAGATGAGCTGTGCGTGCTCACCACTAATTGGCAGCGGCCTGCCCGACTTG
GTGGTGACCCTGAAGCGTGAGATAGCAGAACAAATGCCTTCGGGTATCAGT
ATAAAATAAACCAGCAAAGAGAAGATCTAACCCGAACGAGGTCTGTGGAT
GAACCAGCCAGGCTTTGATAAGGCTGTGCTTTCTACTGatagcatggaaaaaaaaatca
tatatataCGCATTCAGTTGTGCACATATTCTCAAAGGCATAAGAAAGGCTTGCC
TGCAGGAAAAGAGAGTGTTTTTATCTTGCTTTACGTATTGCAAAAGTATCAT
TTGAAATGCATAGATGTCTTTTTCCATTTGAGAAATTGAGgatgtttttaaatgagtatat
tatcttgttttcttaaaattatcgttttcttaaaaatggagtTTATCGTGAATTTTCCAGTGCATATGAGA
AGATAATTACTTAACTTATGTACGAtaatgtggggtttttttcccccagaagaaaattcagtttagtggtc
tttttttttccccttccataAGCTACACACTTTGTTTATTAGCCTttgatgtttataaatatttaaagtgtGG
AAGTCAAATgaatggggccaggctcttttcagtggtgtgcaggaacaggacaaggggcaacgggcagaaactg
gaacacagaagtTCCATACGAACATGAGTAAGAATTGATTTACTATGAGGGTGATag
agcagtggaacaggctgtccaggcaGCTTGTGGAGTCtctctctctggagatattcaaaaccggcctgaatgctt
tcctgtgcaacctgttctAGGGAAACTGCTGTAGCAGTGGGTTGGACTTAGGTGATCT
TTCAAGGTCTCTTCCCCCCTCTGTTTCTGTGATGTTACACAATTATGCTTCTG
CATGCTTCAGAAGATGATGTCATATATATCACCAGTAGAGAATTACTAAACA
ATGTGGCTCTAAAGGGAACACCCATGCTTAAAAGAGTCGCTAAAAGTGTG
GTTATGCTCTTTTGGGAAAGACAGGGAGATAAAGTGTTTGGAGAGAGTTAT
TTTTGAAATCTTGCTAGTATCGTTACTTGTCAGATGGACCAGCTGTATTCATT
CCTAAGAGTCTGCAATAATTTCCAAAGTGTATTCCCTGTCTGCAAAATGCTG
AGAGTCTCTTCTTGTCAGAATTTTGTTAGGAAGTAAGACTTGTTTCACAAG
AccttaacaaaaataatgagGAGTCTTTATTTTAGGAGACGTATTATAATTAATTATAAA
AGCACAATTTGAAGTTGATGACACTACAGATATCACAAATCTGGTGAATTA
AGCCCTTCATTGGTCTTTATATAGATGATACAAAATGGAATGCTATTATAAAG
GCAGAATTTGACTAAATTATGGAAATTAAACAGAGGAAGTAATGAATTTTTC
TAGATGTTTAAGATTTATTACACattgagtcatagaatcattgaagtAATGGCATAATAAAG
AAATCTGAtgtgctgacttttttttcatcCAGAGTGTCTGTGTTTGGATTCTATCCTGCCTG
CAGTTTTTATGTGCTGTTGAATTTCATGGTATCAGACAGACTCAAGCTTTCA
GGAGACTACTTAAAAAGGAATTGATTAAGAAAAAATCCTAGTTTGAAGAAC
TAAAGTGAGAATCTTCCATAGTGTTGTAATTTCTACAGTCTATGCAGAGAcag
ggtttctttcttttctgtttaaaacagaGGTGATTGTGCTTTAAATgttgatgttttcattttaggaTGACAG
GGAGGTACCACAGAGCAAATATTTAAGTTTGAGTGAATGCATGTGTTCAAC
TATTTTATATCTGTTGcacagaagggaagaggaataGGGTAAATGACAACTtcataaaagtgaa
aatgaaacaatttaatAGGTTGCTTACATGTCACTATTTCCATAGAACTAGATTCTTCT
GAAGGAGCAGAAGTGAGGTCTTGTTTATAGCAAACTTGTAAAGCAATGGA
GATACTCAGGAAGAAGGAATGTTGTGATTTAGTGCAAAATGTTGAGTAGGG
TtgattgttgttattttttttttttttccaataaagagttttgtttttctagtatAGAAGCTACCTTGAAGCTGTTT
TACTGGCAATGTATGTTCTTGTTCATTAGGGTACTTTGATATACCAGCAGTCA
GCCTTGAGTTTTAAGTGTTATTAggtgaaaattaatttcagatgGTATATAGAGTATATGA
ATATTAGTTTGCAATAAACTTGTTTGTGGTTTCTGGTTGGATTTGTGTTATGA
AATTTAAGTTATGATGTAATTAACTCTGTACATGTAGTCTACAAAAAAACTA
AGTTATTCCTTACAAGGCATTATTTTATAAGGCTAAAAGCAATTCTTTCCATT
GAGTTCTAAAACTGGAAGATCTCTCAGATACACAGGAGATTTCAATAACAA

AAGCAACATTTAGTGATACTTTACTTAACCACTCAATGTTAACTAAATTTAA
CATTTAGTTAACTTCAGTTAACAAGTGCAAAGCATTCTTTACCGTACTTAAC
CATTTATCTTCCTGTAACGTCTTGCTTGTAGACataagttttaatttttaaaaagaaagttataGC
CACTGAAGTACAGATTCTTGCCAACTTTGAGGATTAGTCAGGGTGGTGACT
CTCCTGTCCAAACATCTAATAGTTAGTAAAATCACAATAGTGCATCTAATGA
GGTGAGATTGTTTCCTAAGTTGGTAAATGTTACTATTTTAAATTCAATGTCAT
TATTTCT
>TCONS_01515649
CCTTCGTCTGACTCTGCACTGACAAGTTCAGTTTCAAAAAAGAAGGataacttg
ttttattttaccaGGGCTTTGTGAGAGGCTGAAGCTTCCGGATAGGATCTGCAACGC
TCAGGTTCAGAAGAGAAGTATGAAGCTGGCTCAAAGCAAGCTGGGCTTGC
TGGAAACCAAAGGTAAAGCATCCGCAGTCCTCCCGCAGAGTGCATAAACT
GTGTTTGTGGTTACACCGTAGCAGCAGGAGCAACTCTTCTGTGCCTCAGAG
AGCTTAGTTGCTGTTCTGCCATCTTTGCTCTTTGAAATGTGAgagcaaaattaatttca
ggagaaaagtaaaTGGATTTAGAACAAGGATGAATTTGGCTTCTAGTGCCACAGG
AATGCAAGAAATCACCCAGCAAGAGAAAGAAGGCTAAACAGTGCAAACC
TCTCATAAATCTGGAGAGAAATTTTAATCCAAAGTGCTTACTGACCTTCACT
GATGCTCTCCTTCATCTCACCAGtgttttattcttcttaaaTCCAGCCCCACCACTGGCA
GACCTTCAGAGAAGTCGGTTCTTGCACAAACAGGAAGGCAGACTCCCTTC
CCATTAATTGAGGTGGTGCTTCCAATCCTCAGCCAGAGGACACAGGGGCAG
GGAGGTGTTTGCCTCCAGGACCAGGATTGGAGCTGGTCTGGAGAAATACAT
TTCCCAGGTGAGCAGATCTGGCCCTTCCTGGGCATCAGGTTCAGAGCCAGC
TCAGGGACAAACTCAGCACTGTCTTCTGACTGTTATGGGGGGAACCAATTT
CTATCCTGAGTGCTTCAGCCCATGCCATGCAACCAGGAGTGTTGGACCCCT
CCAGATGCCCTATGAATGAATTTCTCAAAGGCTGCCTGGGAGGAAACTCAA
TCacatttcagtttaattaaGTGATACCCAAAGTGATGACGTCTGGAACCGACCTTGCT
GGATCTTCTCCTTCCCGCTGTGACTGCCAtaactcctgcagcagcagctgagattGCTCAG
ACATCTGCAGCTCCATGTGGAGCCTCACTGCTGGCTTTGGTCTCATGTTACC
CAGCTGAGATGTGATCCTGGCAGGTCTGGGTGGGGGCTGCCTCGTGCCATG
CTGGGCACATAGCGGTGTGCCCATTGGCAGCTCAATGGGAACCAACAGTG
GAATTACAGCAAAGAATGTTTGATGGACCACAAAAGGCTTTGAAATTTTCT
TCCATGGAAGGCCAGTCTGATCCTATTATAGTCCACAGTACGTCTGCAATCT
GTAGGACTTGCCGGTAACGTGGATATGCATTATTcctttaataaaagcaaatgatggCAAG
GAATGTGGCTCTTTTAACCTTCAGCTTGTGTAACCTGCAAAAAGGGAAAGC
AATCAGCTGGCCTTCCAGCTCAAGAGGTTTCTCCATGTGAGTCTGGCACAC
GCACTCAGCCCGCTCCTGACACGCTTCCAAGAATCCCGTCTGTGTCCCagtaa
aaaaaccccaaccacTCTGTGTTGAGAGAGGCCCATTCAAGCTGCAGCACACGCG
GCCTGTGCTGCTCATATTTCCAG
>TCONS_01631638
GTCCCACTTGGTTGGGTCTGGGAGGGAGAGAACGCAGGCTGAGTGGAGTG
TGATCGAAAGTGTCCGGCGTCACAAACCGCGAGGAGGAGCAGCGTGTTCT
CTCCTGAGCGCGAAGCGGGCGGGCGGTGCTGCTTGTGCAGGAGCCGCCCG
TCATTGATGACCGTTCCCGTCCCACTTGGTTGGGTCTGGGAGGGAGAGAAC
ACAGGCTGAGTGGTTTGTGCCTCATTAAAGGGTTAAAAAACAGAG

>TCONS_01661432
ACATTTATGGTCCTttttgaaggaaggaaagcacaagGCACAAGGCAAAGAATTTTGCA
AGtgcaaaataaatgttgctGAATGaaggagaatggagaaaaatatactGCTTTGAGGCATTTAGGT
GAGAAAGCTGTCTGAAGGATTACCTTAGGATATGGAATATGTTAGCACAcggg
ggttgtttttttttaattgcttatcACTAATAGAAATGATTTctaattgttaaaaaaattagaatatCTTTTTAG
AGAAAGCTACCAAGTGCAACATAAAATGATTTCATCTGGGAGTAGTAAATG
TCttctttatctttcctttttttttctttttctcttcatgtctctttatgttttcattaatttccCTTCAATTCAAATTGTTA
GAGTAGTAAAATCTTCCtcacaaatatttatatgaattaggcctttaatcttttcatttgtaactgaaaatattaact
TAGAAGAGTTAATCATCCTTTTGGTTGAATCTCAGTAACTGACTTTACTATAA
TCTTTTTGCaaataagtaataaaactgtatttcgaatagaaaaaaaaaataatggaaaaattttgtaaatattttttaatgct
gtctAGCATTATTTGTGTCAGTCAGAATAATTAGAAGTTTTTCACAACACTCAg
tatttaaacaaacataaaagtaAAGGCTATAAAGACTTTAAAAAACACTGCAAGTCAATA
ATTCTTCATAGTTGATCACTCAGCATCATTTGTACTCAATAGCACAATTTTgta
ctatttttttaaaagtactcAGTCCTTCAGAAGTCGAGAGTCAAAATTTATCTATGAAGCT
ATGTCCTTTAGCAAAACCTAATTTAAATGTTACACATATGGGAAAATATGTTC
ATACTGTCAATATGAATACTAATTTGCAGTGTTGATCGTGCAGGGTGTGGGA
TCAGTGTCTGAAATTAGTGATTTCTTAACGTGATAtaacaaaatctatttttcttttatgattaaat
tattttggatCATACATAATCattgaaaatatgaaacaatATTCTTTGAAGAAGTATAGCAATAA
TATATAGTAAGAAACAGAGATACAAAGAACATTAAACTAAATTGAACAATT
GTTTTTACCTGGAAAACATAGTATAATAATCCAAGAGAAAAAGCCCAAACT
CTCTGCTTATACATCAGctctatataaatatatatgtgtgtgtgtaaagtGTATTGAATATATTGCT
ATATTGTCCTTATGAATGGTAATGATATCAAAAGAACATTTAAGGAACAGGA
AGGATAAAGGAATATGAAGTGTCCTATAATGAACAAAATGACAGAAGTTGA
AAGAAGTAGTGTCTGGGCCAAGGTAGTACACTTCAAATGCCAGATATGTGA
ATTTGTACCACCATCTTAAGATAAATCTTCCTTCACAAATATACTTTGAATAG
ATCTTGTCTATAGAAATTCCACAATAAATGAGTATTCCATCACATGATAACTA
TCAATACTTATTTGTGCTGAAATGTAGGTTTATGAATGGGATTATTAAGCCAT
TTGTTAACTTGAATGTTAGAAGGAATCCAGGATGGAAGAGCTGGAAGCCTA
CAGGCTGTACTGCCTAATGAGACAGGATTGCCTGAAgaatcattttctttgttatgaAAG
AAGCTACCAACAACCCCTGCACTTCTGCTGCTTAATACTTTcaattaggtttttttttaatg
cttttgccCTTCTGTTTGAAGCATACCTTTCCTATTTGGAAACGAAGTGTCTTAAa
gcaattcttttctttttaatatcaagTGCAATGTGATGAAATAGAATTTTATGACAGTTACCAT
TACTATTTCACCACTTGCCTGTCTTTTGAAAAGTGTTGCAGACAATGCAGG
CAATGTTAAGGCCCAGCTTGTGTCACTGTCACAGTATTAGTACCAAAAATTA
TACATGCAGTGGTAGtacactggaaggaagaatAACAATTAAATAATTGACTTGAATT
ACATGATTGTATACAGTTTTTATCCCACAGGTTTTAGATTCTTTCAGtgcacagag
agaaaagctgTCCCATGAACTATCACAACAGAAGGACAGAAGAGTTGAGTTTTA
TTATGAATTCATTTATTGCACAGAGTTAACATTCTTCTTTATAACTTGAATtaca
gaatatatttaaaagatgaaaaatactgttaattCTTGCTGGTCAGGGAGAAATTCCTTAGTGCC
TACCATAATGCAAAATGCCATTTGTTTATGATTTGTATTTACCATTACTGCATG
GAGCACATTgtgctttgtttaaaagaacCTTATTATCTTTCAGATCTAGACTATCATCTTT
TTTTACCAACATTAATGATTGTAATTGTGATGATGCTTCAAAAGGGAAGAAG
TCTGGGACTGTTGCTAAGGCACCTTCCCTCTTGTTTCTGCATTTACTTTCTA
CTTGCAAAGGACAGCAGAGACCCTTTGAAcatctttattttgattttaaaaaagcacacaaataaA

TGAGTGTTAATGCTTCTTCAAAATTGATGTAAAGAAAATGGCTAagtaaaagagga
gaaataaacaataaagttttttgtttttttttttttcaagtgaatcGTGtttaaatatcacagaatcacagaattgcagtgGTTG
GAAGAAACCTCCAGAGATCAACAAGTCCAAAAAAAACTGCAGAGATACAa
agctgcacagGTGCAAGACCctgcacttgcttttgttaaacctcatctcattttctgctgcccagctctccagtctgtc
cagatcttgctgaatggcaacacagccttcaggtgtgtcagccactcctcccagctttgtatcatcgGCATACTAAC
TAGGGGTGGACACTCTCCCATCATCAAGGTCATCGATGAAGATACTGAACG
AGACCggacccagcactgacccctggggaacactgctagtCACAGGTCTCCAACCAGACTGTA
CCACTGATCACAATCCTCTGAACTTAGCCAGTCAGCCCGTTCTCaacccacctcac
tgtccactcatctataCCACACTTTCTCAGCTTCATTATCAGAATGTCATGGGACACA
GTATCAAAAGCCCttctgaagtcaaggtagatggcatccactgctctccccacatCTACCCAGCTGG
TGATGCCATTGTAGAGGGCTACAATGTTGTTCAAGCATGATTTTCCCTTagtga
atccatgctgactattcctgataaccttcttctttaccaattgcttggagatggcacttttccagggacagagatgAGGCTG
ACTGGCCCATAGTTTCCCGGAtcctccttctttcccattTTGAAGACCTTGACATTGGCT
ATTCGAGTCTCCACacacctctcctgttctccaagaCCTGTCAAAGATGACAGAAAGCA
GTTTGGCAATATCCTCTGCCAGCTACCTCAGCACACATGGATGCATGCCATC
AGGGCCCATGTATTTGTGCACAGTGATCCCACTTAGATGCTCCTGGACCAC
CCCCTCCTTGGTGTATAtctcttttcagaaaagcactgataAAAAGATTTGCATGAGCATG
AAGAAATTATAACATTTTTCCATGGGCTAGGAATCCACCTTTCAAAACCATT
ATTATTCTTGGAAGATTCATTATATCATGCGCTGAAAATGTTTCACAGTTTAG
TATTGGCAACTCTGAGAGACTTATACTAACCAATGTTGATGAAATGTTCTTA
AAATTATTCAGAGAAGAATTAGAtctcaaatttattttccttttgttctaagtgtaaatatatttttttattgttttc
cagTTGTTTCCTATCTTTACATAAATAttgtaaaagacatttttactTGTTGCTATAAGGGG
CTTCGTTTTCTTCTCTCAAATTTCCTAACgttctgaagaaaagaaaacagaaaagagagtgCT
CCTGGCACTTCTAATTATAGGATAATTCGAATGTCATTGAAGGGAAAGGGAA
TGTCCAAATAGTGATGACAGAACAAGAAAGGTAGACAACTGACCTTGATTA
CAACCTAGAATTGTCACTCAACATTACAGAAATCTTTAGGAACTGTTGtttcata
aattatttctgacaTTTACAATAAATATTGAATTAGATCTCCTGAGGTGTTtgtaaagcaaaa
agaaaatgtctttgaagTAACAAGACAAAATCGTACTTATTGTGGTTCTAATAAACCC
TCAAGTAGCATTAATATGATTGTTTACATCTGCACCAAATTCACATAGGTTCA
TGTATTTATATAGATTTCTATTCTCTTCCTTGTGATTTTCCTTGCTATTTGGAA
GAGTGTTAATACACTGATTTGTAGATTTTGAGCCTTTATGTTCTTCTCTTTAG
TCTTACCTATACTCAGATGTTTACAGCATCTCTCATGTTATtgatttaattatatttttaatatc
tgcCAAAAAAATTAGTCAAAAATAGTAATATTGTACTATGtatcttcagaaataattttataat
tagCATATTGCTACCATCTTGGTCTGAATTACTATAAAACATGTTGTTTTGATG
TTGCTCcatctagaaaaataaatttataatgAAAACTTGATatccttcatttatttctgtttctttctaagaagaaac
aaacattaaGATGTAGGCTCATACTCTGAG
>TCONS_01692422
AGACAGCAAAGGATGCTTACAGACCTAACTTTCTTCCATATTGCAGCCTCCT
TTTTCTGGCAGAGGCCACTATATTGATTCACATCTAGCAAGAGTCAGCATTG
TGAGGGTAGTCTTAAATAATGCAGTGGGGTTTGTGTCATCTGCTCTCTGTGA
GCTCCAGTGAACTCCATAAAGCTGCTGTGGGGCTTTCCTAGTGTGTGCTGT
TTTGTCCCCCTCTCCTCCACCCACACCTCAAGgacaggaaacagcagaaattcGGTGCT
CAGGAACAATCCTTATTTTGGAGAGAGGTTGTCTCACATTTGAGGGTGAGC
ATTATTCTCTTGATGCCTTTGGGCATCTGACACTTGGTGGTATATTGCCACTA

TCCTACCCCAGCCAGTGAGCTTTGGAGTCTGCTCATCCTGAATGCTCTTTTG
GGAGGAGCTTGGATTCTAATGCTGATGGGGACTGCTCCCAACTCCCCAGTG
GCCTGATTTTGCCCATTACTGACACTGAGAGGCACATCCTGCTATAGACTGT
CTGGGTAACCGGGGCTGGCTTATGAGAGGGgtgctgctcagccaggctgGAGTGAGG
ATGTGGAAGTGAGCACTGGATCACCTGCAGATCTAGCTCCAGGCGTCAGCA
TCTCcagagtgctgctggaggagcccaGGGTTTGCTGCATCTCTCCATGAACATGTCTGG
ATTCATATTCATGATGCAAGAGCTGTTTTGAGCTAATAGCGTGCTGGGGAGA
TTTGCAGAGAGTGAGAAAATGAACTCTCATGAAGACAATGAGTCTCCAGA
CTACCTAATGACTACTTAATGAGTGTCTAAGCATTGCACCGTTATTGAATTTT
AATTGCCTTCTCTCTGTCtttgtgctttaaaatgaaaaaggagaaaacactaCATGTAATGAGGG
TGGATGATTAAAAAGTAATACCTAGGAGACAGCCAGGCATGTGTGGATAAG
ACCACAAGAATAATGCAATGAGAGAAAGCAAGACTACTGTGTGCTTGCCA
CCTCTTTGGATAAAAAGTGTGTTTTTACAATTGTTAAAAATTTAAGTTATTTT
CAGCATAGTTTGGTAGAATAATAGATTATTGTGTATCTGTGAGGCAATTAAA
GTTACACTGAATGAATTATGGGTTAATTGTTAATGTACAAAAAGTCCAACCC
ATGTTACCAGGTCAGTGCAGATGGGCTTTTTAAAGATCGCTCTTGGTGTTGT
GTTGTTACTGATGTCTGCTTGGATTATTTTCCATCATGTTAGATGTGCAAGCA
TAACTTAGAGCAGTAGATAACAAGAAGGTCCTAATGAAAGGAGACCAAGG
GAGCCCTATATCCGTGAAGCATTTAAGCATGTGTTTAGCTTTGTGcatcagcccca
ctgctgctaGGTGAGCAGTTGTGCTGCCAGGAACACAGGTGGGTATTACTGAGA
GAAACACAGGACAGGGACTTTTTGATCCTCAGCAAGGTCTTCAGAGCTTC
AGGTCCCAGCTTTGAGGCAATGAGCACCATCACTGGTGTTCTTGACATTGC
CAGCCCTTCACTGGTGGTCCAAAGTGTCCACCCTGGATAGTGTTGATGACA
CCATCAGCTCCTGTGCTGAATTCGGGCTAATGCAGTTCAGTTATCTGACAAA
CTAAGCTGAAAAAAGCATGTCGAAGACACTATTGTTCTAGAGAGGCATTAT
TTAGCACCAGGAATGCATGAAGGAGTCAACTGAGACAGTGCAGGGGGAAT
CCACTGAAGAAAGACCTGCTGTGTGTCACCTCTGCGTCATCCTCAACATCA
CCAGTAACTGAATTCCCTCCCAGAAAAGGGGAATTTTCCCTATAAAACAGC
TGAATTATTCCCTTACAGTCTAGTTAGTCATGGAGCCAGAGGGCTATCTTTT
AttgcaaaggagagaaaatgttCAGTCATGTTAAGCAGACTGTAGAGACCCAGATCAG
AAGCCAAGACACTAGATCTGGATGGTACAGGGGAAGAGACAAGacacaaataaa
gcaaattaagtttgttttgcatttcccCCTTCAGCAGCCTTCCTAGTCTCCACAGGCACTCTC
TCAAGTGCTCCTGGGACACATCAGTACAAAAGTGAAGATCCAAACTGGCG
TGTGGTAATTGCAGAAGCTTCACCTACGAACTATATGGAGAGCACTCTGAA
AACAACTGAAGATTCAATAGAAAGCATCCCCTGAAGGCTTCAACCACCAG
CAGGGAGTTCCTGGAGGACATGGATGTTTGCCTTTGCTGGTAGCCCTGTCA
GAACCAGCATGATCCCTGCTCACAGTGACTGACCACATGCCCCCACTAACC
TGCAGATTAGGGGTGCATGTGGTCCCTAGGATGagaagaaatggcttcaagttgtgccactg
aggttcaggttagatattaggaagaatttcttctcagagagagtagtgaggcagtggcacaggctgcccagggaggtggtg
cagtcaccgtccctggaggtgttgaagaaccgtgtggatgtggcactgagggacatggtcagtgggcgtggtgggggtg
ggctggcagttggactaggtgatatGAGAGGGGGTTTCCAACCGcattgattctgtgattctctcatGAG
TGAGTTTGGGGTACTGTGCGTCACAGCTGTTTCCAAGTCCTTGCAGCATTT
CAGGAAAGTCTCCCTCCACATTCCTGATGATAGATGTAAAACTTATGGGTAC
AACTTCTCAGAAACTTTGGATCGATTGTTGCAATCTCTATTTGTTTATTAACA

ACTACCTTTTAAGTGACACCAGAGCTATGTTGGTGTCACATCCACTcctctgatttt
cctttcttaagaGTTGATCTTTTCCTTTACACTTTCCAAGCATTGCCTCCACTTTAAA
TTAGTTCTGAAAGTTTGAAATAATTACAAACAAGGATTCTTCCTGGGgatgaaa
gcaaagcatcaaggaaaacatcacagcaagacaaaaataatgttctggCTGCAGAGACTCCACAAAGC
CAAACATCAGAGAGACAACTCAGTAAAGAACTAGATTTTCAGCTACCAAA
AGAAGCAGGTTTCCAGAATTCTTCCATGTAAAGAAATACCTAAGAGGATGT
AATAGAAAGAGGCGTTTTTAAGCTTTATTCTTAGGAAATAAATTGACAGTA
TTTTAGTGTCATCAACACCTGAATCTAACTCTTCGCCATCAAAATACTTACC
TTATTGCTGCATTGGTAATCAGAAGAGATGGTACAAGAGTCATGCCATTATT
GTCAtctaagaaaaatacagtttttcaaGTAATTAATCAAAAGTTTGGCCACCAAAAGTGT
AACGTGGCCTGGATGAGTGAATGTGAAGAAAGATACTTATCTTCAGGATCG
ATGTCTTTTTTGTAGAAAAGtaaaaggaatttgaaaatgtCTGAAGTATGAATTATATCA
ACTGGTTGTGTGAAAATGAGCTTCAATCAAACAAGCTTTGATTTCCCAAAG
AGAATAAGACACCTTTTGCACTTTCAATATATTCAGTTGCAATCAAAAAGAA
TGGACAgatatgatttttctttctttctttctttcttttttttctggcaacAAATTTGAGGATTTGTGAAACC
TGAGTTGATATaataatggcctcaagttgtgccaggggagattcaggttgggtgttagggCAAATTTATT
CCCCAAAAGAatggttaagcactggaatgcgctgcccagggcagtggttgagtcaccatccctggaggtgttcaa
gaaatgttgtACTGGGGGACAGTGTTTAAtggagaaatattggtggtaggtggacttttggactggataatc
ttggaggtcttttccagtatcggtggttctatgattctaatgatTCCCATGAATTATAATGGGTGGTGTTC
TCACATccacaaaaaaaattatatttctccATGGTAAAGCACCAGTGCAATTAAAGCTGTG
caagagcaagaagaaattattccctGAGAGCCACAGACCTCCTTGTGTAGGTCATGTATCT
GGTGTATGGTCACAGGGCCTGGTAAGGATGACTAACAGAATTTCAGGCCAA
AATGGGAATCTTGGTCCATTTCTGGCTGTGGGAGCTCCAGCAACAGTGCAC
ATTGGAGCCATCCATGGGTGCTCCATCTGCCTTCAGCTCTGGAAACAACAT
CTGAAATAGAGATGTGTAAAGGTAACTTAGAACTAAAGGACCTTTCCAGGG
AGAGAAACATTAATCCTTAGCCATGATCTGCAGGAAGTAAGCAGCTGAACC
CTTGGAATGGCACTTTCCCCATAAATCTGCTTCACCTCAGCATCAGATCTTT
TCTAGATGTAGTTCTGCATGCTATATTCAGAGCTCAGAAAGCAATCCATACT
GAGCTGGGTTTACTTCAGGCATTTAATCCTGTACAGGTGACCTCCAGAGAG
CCTTCTCACACTGCTCATTGTGCTGTAACTCAGCAGTGACAGAACAACGTG
GGAGAGGTGAGGCCTTGCCCGGGGCCCACAACTGTGTCTGACCTGGCTGC
TCCCGTGCTTTGGCACAGACCATGGGTCAGGGCTTCTCCTGTTCTGCTCTG
GGCTTCTTCCTGGATCTGTGGGGACAGGAATGTGGGACATCACATGGGCAA
TGCCATACACAGCAGGTAAAACCTCAGTTCTGCTGCTACGTGTATAGAAGG
AGTCTGCTGGACTGTTGTGCGGGATGTTGTAaaatcaccatccctggaggtggtcaagaactg
tgtggattaggcactgagggacatggtctgTGGGCAGTACTGGTGctaggtggatggttggactagatgatct
tagaggtcttttccaaccttaatgattctatgatctgtccATAATTCCAACAAGGCCATATGTTGATAC
GATGAGGAGTCTCATCCTAATGATAGAGTTGGAGCTGAGGTGCAAAGGGCT
ATTGCTGTTTAGTGGTAGTGACTTTCTGCATTATTGTAACCATCTTTCCCTAT
TCCTTATGCCTTTGTTACATCCTTCTCACAGCTATGAGTTACTGTCACTGAA
GACTGGAAGCAATTTACTGCCACCTACGCACAAGTAGCTAATTATAGCCCA
GGAGCAGGCAGAAGGAGCCAGGTACATTATGaatctgtttttattaataAGCAAGCAG
CTAGGCTGAGGATTTCCAATTATGCCCTGTGTGTTGGCAGCAGATTTGCTAA
TATCCTGGCTTCTGTTTCGGGAACTAGTACAGCAGTCCTGCAGGGTAGATGT

ATGCTCCCAGTGGGGATGGGGCTATAACTGATGAGTGACGGAGCCCTGAAA
GAAGTACACCATGCCCTAAACACCATGTGCCATTGCCCCAGGACTTGGCCT
TTAAACGCCCATTAAAACCCACTAACTTTGATACCTGTTGTGGAAGCAGGA
CAGAAACTCATTTGGTGGTGATGTGAGCTTTAGgcatttattctccaaaagagctcTCTTG
CTCGGAGTTGtgactttctgcttttcctttctccacttAGTTCCTTGCATAAGCACTGCCATTG
TTTCTGGCTGTCCAGAGCTTGGTGACTCCTCAGTCACCACTGTTGTCTGTG
AGCGCTAACAGCACTGTAAACATGGCAGCCTTTGCAGATTCAGATAGGAAT
CTGAGGTTTCCCACAGCTGGCATTACTGGGGCACATCCTGTCAGTCCTCTG
ACCCGATATATCCCGAACAGGGCACTGGGGAGCACTGGGGAGGAGGCAGT
GCTCCTCCTCCTACCTAGCAAATCAAACGTGAACAAGAGAGCTCACAATGT
GAGGCCTTCTGCTATACTGGAGTACTGCTAAATGTGTCTGAGTTTACATCTG
AGTCTGATGAGTTTAATATCAGCCATCAAACATAATAGAGCAGACTGTCTGA
ATTCCCTTCCTGGGATAATAAGATATACTGAAGTAGGAATGATTCTTTTCATG
TTAATAAACTGACTGACTGCTTTTCATTGCATTGGCACCATGAGGAATTTGA
ACTCAATTCAGAACAAAGCCCTTGATCTGCCAGCAGGCTGAGTGGAGGTT
GTTTGAATTATGCCATTGTTTTAAGATTTATTAATGTGATGGTGAAGCTTCTG
TCCTTCAGCAAATGGTCGCTATGAAATATGAACTCCTTTATTAAGGGAAATT
AAGGAAGAAGTGTAATGGACGACAGTGTGCTCTGAAATGTTATTCTTGTTG
TATAAGCTTTGCGTTATGGTTTTACCCAGAGCGGTCAGCCAGGCTGGATCAC
CAGCAGTGTACCTGCTCAGCAAGCAGGGCAGCTCCAGAGGATGtagaagtgaaa
aaagaagCCGTGACAGGCAGCTGGGGCAAATGAACGAGAAGGGAAATGGCA
AAAGAGCAAACCAGTGGGACACATCAGCACAGGTAGGCCCATGTGCACTA
CACAGCTCCAGAGAAGCACCAACTTGTTATCACATGAGAGCATTTTGCGCA
CAGGGATGAAGATGGGATGGGAGTCAGAGATGTTTGTAATATGGAAACTCA
AAGGTACTGGCTGGAGAATCAGACAAATTGACACACTTCCATCAGTAGAG
AGAGCAGGCAAGAAAATTAGGTGATAATGAAACAATTGAGCTAATCCAAG
AGGTAGATGGGGTTTGGGTCTGATATGCTTTGTGGGTTTGATATGTCAGGAG
GTGTGCTGACTAAGTTCTTCAATCAATGTGTATATGGATGTGCATGATAGCTA
ATCATAAGCATGGGTTAATCAGACAGACTGCACTAAGTGTGCTCTTACCTTT
AGACTTGTGGGTAGCTCTGTGCATCGCCCACTCTGCTCATTTCCACTGAGA
GAAATTTTGTCCACCACACAAAATTACACGTTATTACCCTGTACATTGATTG
TGTCCATCTGCTGGGCACCAGGCACTGCTACCTGGTCACTGTGGTATGGCT
TGGTGGTGGCTCCTTGGACAGAGCTCTCTCGAATGCCATTTCACAATCTGT
GGGTTTGATGTGGTGACTGCCAACTGCAGCTGGTCTCCAaatggggagcagaggga
ggtTTCTTTTGGATCTGGGCTgtcaggaggaagaggaaagttGCCAGGCCGCTGGGGAA
ATGGGTTTTTGGGGTGGCCAGAGAAATGGTGGGAAGAGACCGGACTGGGA
AAGCCATACAAGGGCAGGAAGCAGTGGCTGGGGAAAAGCAGATGTCTCCA
TATTATACTAGAAATTGGCTTACAGTCCTTCCCCCACTCCTTTCACCACCTaatt
tctgctcctgctgagaTGACCCCACTTTATTGATTTCCTGAGATCCCATGGGAATCAC
GGGGGATAGCAGTGAGCTGCACAAAGAAATGAGCAGTTTGCAGTATCACT
GAGACACCCGCTAAGTGGCTTACAGACTTCCTCACTGACAGCTCACAAACT
ACCTGTCAGTGGGGAATTGTCAtccaactgcttttcttttattattattatttatgtatttatttccccaGGG
AGGGGGCTGAAAGTACTGGAATGGGGCCAAAAGCACTCCGTGCTCCTATC
AGCGGTCTGAAATGGAGCATAATTTCTTTGCTGACAAAATATGATGGCACA

ACAGTTTGTAATGGGGAGATGACAGGGAAGGCATGGCAGTCATGCAAGGT
AATCTGGTCGTGGTGAGAAGCAAAATGCACCCAAGAAAGGCCAGTTCCTG
GGCTATGGTCAGCAGGTGCAGAGAATAGAATTACCTCACTCCTGGAGAGCT
CCTTGGGCCTATGCAGAACCAGCACCTGAATGAGCATTTGTAAGACTGAAT
CTTTGCTGATAGACATTCCTAGGGTGTAAAGAAGGACTGGGGAGTATAAAT
AGGAGGAGGGGAGATAATGACAGACTGCCTTTTGTGCATCACCTGGAGTTC
TGTTGGTCTGAGCCTGAAATCATATATGAAGACAATGGAGAGGGACAGAAA
ACATCCACAGCAGTCTGAGCACTTCCACTGAGACACGTAGTCAGACGGTTT
ATTTTAACAAAGATAGAAGATTAAAAGATGACTCATTGATCCCTCATGTTGG
GAAAAATACCAAGTACATAAAGCTTTCTTGTACTTTGAGACAAAACGCATC
CCAAAGCCTGCAGCTGAGCAAGGAATCCAGCTGGGCTCAGGTTGGAATGT
TTACTGGTGAGGGCTGGTGGAAAGGCAGATTCTCAGCCTCAGGTGACCTG
CAAATCAATACCTGAAGACCCTGTGCAGGCCTGAACAGCACAGCTACAAG
GTTCAGGCCTCAGCTCAGTGTGGAGTTCTGTAGGTGAAACAAAAGGACTG
GGATATATGGGGAGCGATGGCCTTTCTGCCTtcctgggcagctctgcaggtaCACTTTTT
ATGCATTGATACAAGcaagaagcatttatttcatcagcaggatctttttgtgtttgaaagTGCTAGGGA
GAATGTTAGCTGATTGGAGAGGTGTTCTCTTCTTAAGGAGCATCACACCAG
GAGTGTCAGCCAaagctggttttcttttccaagaaaaatgaTGCAGAGAATTTCACACTTCTA
CTACAGCATCTTTTatttaatcaggaaaaaaatcttcagaggTGTTGCTACATCTGATTGCAA
AAGCCCTAATCAGTCTTCTCTAGTTTCAGCTGCATTGCTTCTGAAAAGATCG
TCACCTCCCATTGGAAGTGCAGCAGAACATGAAGGGCTGCAATTATTTAGT
TAGGAACGATTTGCTGTTGCTTCTGCCTGGTTAGTCTTCCCTCACTCAGCTG
ATGCCACTCAGGAAAGGGCCCTTGTTGGAAGGAGGGGATGCCTAACAGGC
TTGACAGATTGCAGCATAATTGGCTTAATTCCCCAGAGACTAAGACTCAGA
ACAAGTTTTCAAAGTTGAAACAAAAGTGGTGCAAGaaatccttttctctttcactttaGCA
CACAGAGCACCTCACCACAGATGGATTTGCCTGGGAAGACTCAGCTATGA
AAAGTGTTTTAGAGCCCCTGAGTGGACCCAGccccacagctctaCTCTGAAGAAA
GGTGACGGTTGTCCTTCCCTGACTACCCAAACAAACAGGCTCTATGTGGTA
TTGAATACATGATATTTGGATTTGAGTAGGTATGTTTAAAATGTCCTCTGAAT
GAAGCAAAACAATGCAAGGAGGTGAGTGCCTCATCCATCAGATTTTCTGCT
CTCCCCCCATTTCAGACCCCAGAGTTGGGAAGTGCTGATCCTTCTCGTGTT
CATTTTCAAGGAAAGTATAGGGCACAACTGGAAAGAAATGTGGTTACAGCT
CCAGATCCACCCACGGGaagggagggctgggagcactgcagcatgGGCACTGAAGGATTT
GTCGAGGTTTAAAGTGGACAGGCACAGGTGCAACAGGTGTTTGCCACAGG
TGTGTTGGAAGAACACTATGCATTGCCAGATTTTACTGCTGATGTGCTCTGT
GGTCTAGAGGTCTCACTGTGCGATGTCTTCCAAAATCTATCTAGACTACCCA
GAATGTGAAAATCTATAACTGTTTTCAGATCCCCAGTGTTCTTTCAGTAGTC
TGCCTTGAGATGACAGTGCATACACTGGTTCTCATGCTCTTGTTATAGGCTAT
AGTCTAATGTGTTGCTGTACAAAGTCTGCATTCAGTAGCTGGGCTCTCCAC
AGCTCCTTCTTCTAGCTGCATAATGGGTAACTGGAAAGGGCAGATGATAAC
AGCTGCACAACAGATTGATAAGCCATCTATCAAAGCATTAGCAGGCCTGGA
TGACCTGCAGCATGGACTGGGCTGGCTGTGAAGGGAACTTGCTCTGTGTCT
GGCCCCCAGtatgaagttttctttctcattctctgttccaagaaagaaagaatcagcCTTTAGGGTTGACT
TCTTCCAAAAGATTTAGTGTTTGATTCCCAGTCTCAGTACTCATGCTTTCTC

CTCAGCTTCTGCTTAGTCCTGAAAGTCTCAGTTTCCAAGATAAATCCATCTG
CCGGAGCTCGCCTGGCAACAGCAAGGGCTGCATTCAGGCTTAGTAGTGTG
AGCCcgagagaaagaaaagttgtCCAAaatgttgttgttcttgttgagTTAGTTTTCCC
>TCONS_01702025
TCTCCCTTCTTGCACTCTTACTCCAGGCCCTGCAAGTAGGTTCATCACTTTG
TTCTAACCAGGGGTGCTGGAGACATTCTTCAGCAGTTGCCCGTTCCCTGTA
AAGGAACAAAGTGTTTAAAGAAATATCCCAGTGAACTTGAAATCTAGTTAA
GATATTCATAGTTTTCTTCTAAGGAAATGAATGTACTAGACTGATAGAACTA
CTAAAAATGTTCTGATAGCTACTTTCTACCCCAAAAGACCAACAGTCCAAG
AAGATGAGCTGTTAATGTTTGAAGTAAAGAAATTCAGAATGTTAAATACCA
CAGTTTACTGTATTTCTGAGCAATTgtctttttccttaaatctaTGAAAGTCATGCCATAT
TTACATTCAAGTATTtctacacacaaaaaaaaaacccacaaaaaaaaccccaaccaatCAACCACCA
AGATACAGTAGACAATTTTGGTGGTTGACACGCCATGTGAATAATTCTGGA
ACTTGCACAGAAAGTAACTACTTCTGTTTTGAGAACTGGAATTTCCAATTT
GCCTCCCCTTAGAAAAGCATACTAGTGAACTATGGAAGAGTAAGGTACTCT
ACTAATACATCAGAAACAGAAGGCTCAAAGCACAGCTCATATAAGAGACAT
CAGCAGGGTACACATATCGAAAGCCTGTAAATGCAGTGTAGTGTGGTACAA
TCTCCTGAAATTTTCAGTTCTAATTGCTAGAGTTGGACACATTCAGAACATG
CAAGTCAGGAGGGAAATAGAGAACTCTTATAATGTGTATGAAGAGTTATAT
GCCTAAAGTTGTCTtaaacttgaaaaacaaaaaatcaagtATTCACTTTAAAGTATCTATGC
TAATACGTAGGACTGGATTGGGTTGTGTACTTTACTTACTCTGGTTTCTTCA
CCAGCAAAGTTTTCATGAAATCCACAGCAGACTCTGATATGAGGTCAAAGT
CTTCTCCAGTGTAACATACATTCATTTGAGATATATTTAAGAATGTTTCTTGT
TTATCATCCCCTAAGAAGGGTGATATCCCTGTTAGCATAATATATGCCAGTAC
TCCAATGCTCCtaaattgaaagcaaaaaacacaaaaacaggttttgttttgtttggtttgcttttatcttgcttgttttctttt
taagcttttcAACAGTGTAATGATGATCGTTAATTTGTTCAACTTACCACATATCGG
TTGCTGTACTGATTGGGTCATAACTCAGAATTTCAGGAGCTATTCAGGGGAT
AAAGAAGTTACATTTTTTCTCATACAAAGACATGATACTGTATTTACAGAGT
GCAAAAGCAGGCACATGGaaaaatttttaataaaagactGTCATGCTTCAAGTGGTTTAT
AAATAGATATTTATATTCaagttatttaaataattactaTCATTAGTTAGTTTcaggtaaaaagaaaa
ctgttggCTTTTTATTGTCATCTTCAGAAGCAACTATCATCTCTTTACCAATTAAG
AATGCACTATACAAATCTcctattgaaaaataatttcaatatcCAACATTCCATATTGCAGCA
CGAAAACCCATACTCATAAAAAATATGCTATATAACTTAAGGTTATTTTCAAG
CACATTTTCAAAGCCTagcatgattttatttttaatgaaatggatACAAAGCCCACCAGGCTA
GAATACCATGTTCAGTAAGAGTATGTATTTTTCAACCTCCCATTCAAGTAATA
CTTGTCATCACTGCTCAGATTTGTCTATAGTGTGTACTTATCTTCAACACAA
GCACAAAATTGTTAGTCACTAGTCTAGTGATTCCAACTTTTACAGCAGTATC
ACGTTTAATAACTTGATCTTCTGACACAGTAACTACACAGCCAGTCTGGCAT
TCATCAAGTTTTGAGACAATTCTCCACTGTTGATAGTACCCTCCTCCTCAAG
GCTACACCAATGCTGTAGTAACAACTTACCTACATATTCTGGAGTTCCCATA
ATTTCTCTTAATTCCTCACTGCTCTTCACTATCCTGGAAAGGCCAAAATCTA
CTATCTTTATGTCTCCCAGTGGAGACTTACTGGTTAGTAGAATGTTTTGTGG
CTGAAAAGTAAAGCAAGCAGATGTTCAAGATCACCAAAATCTTATGTCTAT
GACACCATAAAATTGCATAATTATTTTAGAATAACAATGATATGTTCTGTAAC

ATATACAGTACCCAGgacttttactttttttttttttttaaaccaaaggTGAACTTGAAGAGGCAAA
CTGGCTCAAAGCAGTGGCAAGATTTTCAgttcagaactgaaaattaaGCATCTACCTTT
CTTAGCTTAGAACTCATACAAAAGACATACCAGTAGCTACCACTTAGCAAA
AGGATATGAAGATCTCCACACTTATACTTTTAGTCCTTTGTATtacctttcttcccttctta
tTCCTTCTAATGTTCCCTCCTTGTTACTTCCTGGTGTAATATAACTCCTGTTTA
AGAGTGTCCAAAAACATTCAAgttcatgtttttaaatagtaattaAACTTTCTTAACTTTAC
CTGTTTAAAGAAACTCTGATGCAACTGACCTCTATCTTGTTCAAGGTACAA
GCAATTGCATTACAAAATACTAActgaagaacagtaaaaaaaaaacaaaaaacaaacaaacaaaaaa
aaaagccaacagaagTAGCAGCCTGAAAAGCAGGTTTTTAAGAACATCACCTTTGG
ATAAGTTTGTTTGCTCTGATGTCACTAGATCTTGGCTGTCATTCCTCAACAC
AAACTCTGCAGTCATTCAGAAGTCCCTTATCTTCTATTTTGAATGGCAGCTgc
tgagtttaaaaaaatacttaaaaaaaaaatcttaacacTAACTTTAGTCAGTGGGTCTTAGTCTTTTG
CAACTGGAGCATATAATTGCTAGACCAAGTCCCTGTCTCTCCGCACTGGGC
CAATGATTACACCGTGCTCTGCCATTAACTTCTGCAAGCAGTAAGACTGAC
AGTTCACACTACGTTCACACTTCTGGTAAGCCCAACTACCATGAAAACGAA
GAAGGCTTTATTTACTGAAAACTTGGATTATCACATACTAGTGGTGGTTGGG
GACAAATGAGTATGAACACAGAAGCAACCACTGAGGGATACAGTCCAACT
GGATTAACACCAACATGTATAGTTTTCTCCtattcacatttttcattCCCACATTGGTTG
TGATGACGCTATTTTACATTAACATTCTTCCTCTtgataacagaaataaaactgtacAAGA
ACATGTTAAAATCTTGGCCCAGCTCTTCTTGCTACAAGACCTGAATAAATTC
AAGTTCTTTCATTCCTGTAAAGTTAAGCCACATTATTTAAAAGGAACTCCAA
GATgtctattttctgtttgtaaatgTATGCGTTAACAACAGCTTATTTTTGCTTATAAACTAG
GATCTAGTGGTGATACAAAGGATTTTGGAGCGGTATATTACcatattcacatttttattct
gtatttgtattagaaaagcaaacatctgaaaaaaaaaaggtggccATGCCTCCTTAATTAGCATCTCAG
ATGTTCTTCTCAGTGGGAAATTTAGTTGTGTTCTGAAGAGGCAAAGAGAAC
GCATGAGAAAAGCATTTGCCCTCTATATTTAAACGAGGAAAAGTTTTTAATG
ACAAGAAATTGTCTGCATTACAAACCGACTCATAACCCTAAATCTATTtgtactg
aaataaaaccacttttcttcttttaaggaGCTACTATAATGTTCAAATGAAGCAACAGTTCAAA
CCCAGCTTTAAGTCCTGAAGATGATATAGCTTTAGAACTACTATCATTTTCA
GCTCAACACTCAATTACTCTAAAACttaaagagaaggaagattGTTTTCTTACACCTAT
AAAGTGgtctctttattattattatttttacactGCTGTACTATCTAGGCTTGACAGTACATTGA
TCTCTTGCAAAGACCAGACAGGCTAGAGTCTTGCCTACTGGCCATTTTGCA
AAGGGTCTTGCACTCGTTATCAAGCAGCCATGATATGGCTACTGTAAACTG
GCAGATGGCAATACTTACAAGCCCTCCTTCCTACTGCAGAGAGGCTTAAATT
AAGATTTATGTGTGATGTATACTGAACAAAAAGACTAAATGGACCATTTTAC
AAGATCAGTATGTAGACAGAGTTCACATTATGTGGTCACATTAGTCTACCCT
AATGCCACATTAGCACTGCTAGCAACACGTAGCttaggaaagatgaaaaaatgaataGAG
TTGTATTTTTCTAGGCATACAGCATCAGAATCTTGGCCTTTAGGAATCTGAG
ACATTGGTTGAACCTCTAGAGTCATGTACAAGTAACACCAGTGAAAAGCAT
AATTTGTTACTGCAAATGTTGGCCAATCTTACATCCCTCATTAAATTAGATTA
AACTACTGTCACATGAAAGAGAGCACAGCTCTCTTCATGTTCTGAACTTCA
GCATAAAGGACAAAATAAGACAGTGCATACAAAGACCTGTTCTCTTCTAGC
AATTTAGGTAAGTTGCACAGATCATTTATAGTAAACAGACTTAGTAAAATGG
ACACCCTTCAAACCTACCTTGCAAGTAGGTTTTGAAGTTTTGTAAACTGAT

GATATAATGATGGCAAAATCCATATTGCAAATTTTCCATTATTATAGAAGTCT
CTATGAGAGTCTGGCCATaatatttcttgaaaatttaGTTGACTTTCAAAAGAGTACGAC
TAAGGACGCATAATTATCTTTTTAAACTAAACATCTCATAGtaacatgaaaaattaatga
agttAACCATCTGATTCTACATAGGACAAATGATACAATTTTTTTGCAGTACAT
TCCTAaatgaggaatttttttttcatggaaccCTTGAGTACTGTATTACATTTTAGCAGATAAG
AGTTAACAGAATTTTTATTGTCTGTTTCTACACATCTGTGCAGAGAAAACTG
TACCCAAGCTTCCTGAAAATGGGAAGTACCCAAGGAATTATGCAAACAAAC
TGGAAAAGTAGGCTGGTTGGAATATCCTGACTACATTGTGTGAAAAAGTAG
TTAAGCAGGCTGATCAGTGTGCCCTACGAAAAAGTTTAAGGTCTCAAACAC
CTTTAAGGAAAGTAGAATTTGCTTGGGGGTTTAGTTTTTTTGGTGAATGTAT
TCAAgtacaaataaatgaatgcaaCATTCGCTAAAGAATAGACATAATAGGGTGGCAGT
GCACTTTATTCTGCAAGATAATCAAGACAAGTCACATAAACTGAGCAGCAA
AGTTATAATCAAGATAGATTCTGTAGAACCTAGATTCTCTACCTGCCTAAAA
ATAAGTAGCTGTGCTCTGTTAGAGAAATTTGAACTCCAGATGAACTTTTTAA
GACAACAGAACACTTTATCTTAATGTTTAACTCCTTTTATTTACCTAGCTGTT
CCTTCAGACAAGAAATACCACACTTCTATTAATACTGCACTTCTGTATGATG
AGCATTCAGTCTTAATCTGCTTGgaatactgattaaaaaaacacGATACAGATGAAATTT
CCTCAACAGGACACTACCTCCATAGTTCAATTATCTTACTTTCAGAACACTG
GTATAAAGTGACACCCAAAGCAACTTCAAATTATAACTCTATTAATTCCTCA
CAGTACACAACATCTTGGGAaccatacagaaagaaaaaggtgtcTGTTGAATAAGCAGA
GTAACATATACTATAGGAGAACACTGCTTGAAGCAGTTAATTAGCAATGTAC
AGCTTTATCTGTCTCAGACAAGCAGTAGCTAGTCCTTCCTCAACTTCcctacttttt
taaaattataatttgaattcttttccattcctaACTAGTTAAGCTTTACCTAACACAATCAAGTCA
AACAGCTGATGAAAAAAGAAGCTTCCCTTCCTCTGCAGCTTTTATGTGCCA
CTAAGACAAAAGACTCGTAACTACACTCTATGCACACCctgcaaattaaataaaatcaaa
acaatgcTACTGAATGATATTTAATTTACACTCAGTAGCATTATGTTTGCTGTTCT
CTGAGACAAAACTGAATTATGTTATTgtattttgctattaaaaacaCATGAAGTTTGAGC
CATGTTTCACAATAGACAcacttttggaaagaaataattGGACATCAACTCTGCTAAGCC
ACATCAAAACACTCAGTTCTTCTCATATGAAAGTTTTGCAGCTAGTAAAGA
GGCAGACAGAAGCAACAGCAAGCTGTCAGCCTCACAGATCCAGTTGATCA
GATTTATAATACAAGACTAAGACAACAAGCTTACAGTCGCATCCTAACAAC
CATTTTAACAAATTCTTCTTCTAGGGGTAGAGCCAATGGGTTAATAAGCACA
TCACAAAGAGCAGTACTCAACtaattatgttttattaaagCTAGCAAAGCAATTCTGGG
TCTTTTAACCTCTGAGAACTGGAGATGTTCGATTTTATGACATCAGTTTTTT
GATCAGTTGGGAGTTTTTTAAGCAAGCTTTCTTCTAAGCTTTGAGAAAGCT
ACTTGTTTGCAAGCCAGAATCAACTCTCCAGTGTACTTTCACAATTAAGTAC
TGTAATAACATAAAAACACCATAATTTATTAGACACCCAGATGCTAGGGCTA
AGGAACACCACAGCTTTAACTTCAAAAATGTACAAATATATACTGTCCACA
AAAGACATGTTTTAATTTCACATAAATTGCTTCAAGGAAATTCAATGGAAAT
ACTTTTATCCAGCACAGTATATTTACAGACAAAGCAGAGACCCTTAGTATCT
TCTATAAACATACCACAACTTTAGTCCTTTTGGACACTTATCACTTGCTAAG
AAATTTTCAATAAATATTACTACAATTATTTGATGTGCGTGACTAAATCAATG
GGTCACAAAGTTTTAGGAGAGAGACTCTAGGGTGATTTAAATAAATGGTAA
CACAAGCAAATAGATGAGATATTTGTATACAAATATTACACATTTTCTGCTTT

AGCTGATGAATGGTTCAtgattttctgttaaatataaGTACGTATAGCTTGATACAGGAAT
CTACTGGTAGTTTTGTAACAAAACGCAATGAAGTATAAATAAGGATTCTGGC
AACAGAAGACACATGTAGTGCCTTACTCCTTAGAAAATAGAGGAATTAGAC
ACGTAGGAGAATTAGAAAATTTTAACATGGGATGCTTTTAATACTGGTTTGC
TTAGAAAAGTAGTTCAACCTCAGaataaaattgctttatttcataAGAACATATGGATGAA
AGCTGTATAAAATTAAGTTTACcttattaaaaatggaaaataattttctggaTCCCTGGGTTAC
AACTTACATCTTAGCTATATGTAATACTCTGGATTTCTTATGTCAAAGAAATC
AACTTCTTATGATAAAACTGCGTTCGTAGTATGATGTGACCAGTAAGGAAGT
CAGCAGTTAGGAAGTAAGAACAGTACTGCAACACTTTTACCTTTAAGTCAA
GATGAACAACATTGTTTCTGTGCAAGAATGAAACTCCTTCTAAGATCTGCTT
CATTAGTCGTTTCACATCTTTCTCTTTGAAGGCCTCCTCTCTTTCAGCAACA
CACTGGTCAAAGATttcacctccagcagcactaCAGAACAGTATGAATGTCATTATTTC
AAAAGCTAAGCTAGAGCACCCATTTTATTATACTAGCATAGAAAAGATCAGC
AGATGGTATCACCCAATGGTGatattttcagttattaaaCAAGTTGCAAGCTCTAACGG
CCTAAGAAGACTTCCATTCAGTATGACAGCTTTCGAAAGAAGATTAATATTC
CCTAATTTAAGTAAAATAGATTCTACAGAAACCGTTGAGACGATTTTGGAAA
TATTAGTGACTGTGCTCAAGAGATGAATGCCACACTTGTTTAAGGGCAGTC
CTTAACAAAGATGTGTCGTGCTATTTCTATTAAACTATATGGAAATTTGACCT
AAGTAAGCATTCATAAACACAATGTAAAATAGTTCTGCTCCTAACTCTCCTC
AAAACACTTTGTACAGACTCAGAAAAGCCTAAATATAAAGTTTATATCCGA
GTTGTTTGTGAAAAATCTGGTGTACAGGGATGCATTAAGTTTCTACACAGCtt
tcatcttaaaatatttttgaaagttttAGCTCTCAGAGCTTGATTCCTCATCTTTTGCTTATATA
TgcttaaataaataacagaagaaaaatagaaaaataactaCAGATGAGTGGATGTTCTTCGAGTAC
TCCAAAGCAACATGCAAGATCATCTCTAAGCATAACAGAGGCTCCATCTTA
AAACAAGAATGGGCAGAACAACATGAGAATGGGGTGACTGTACATATGGA
TTGCTGAAGTACTACTGATCAATGCACAGATGAAATCAACTTTTGTGTGAA
ATTATAATCAGCTGAGTGAAAGATGGTGAAACCCCCTAAGGACAGTCGTCT
TCAGTTACCTCACAATGACTTTGCACAAGTGTTGATCTTTACTGATACATAA
CAACTTCTTCTACCctggcttgctttttttttttttttttttttaaatatatcatAAATTATAGTACCTAAA
CCACCcggaagaaaaagatattttccaacAAGGACAAGTCAAGCAAACAAGGCAGTTCT
ATAAAATGGTGAAACTGGATTACGgactctgagaaaaaaaaatcacttaaatagtattcataaaattaa
ataattgaaTATAACTTGGTTGATATGGTCCTTGTGCCTTAAAATGCCTcatgttttatattt
ctttttattttaccttcccagattttcatgttatttaatCTCCCTGGAGAACGTcttgcaagaaaaaagaaatttagaa
GATGGTTACATCTCAACGTTTTATATAgagctttgtgtttctgttcatCGTATCGGTTAACT
GACACAGCAGTATGGTTTGCTTTATCaaactgaggttttttttttttttttaaagaagcagcCTATT
GAGCATTTTTCTTAATCCTGTACCAAAGATGATGAAActaaagaatgaaagaagttATC
ATGAAGTGAGTGTTAATAATTCAATTGTTCTTGAAAAATGCATACTCCTGAC
ACCGTTCAGTACATTTCTTCACcaagtttttatttaattcaagaaaaaaaggtagTTATATTCCAG
CTTTGGACAAGATTCAGATACTAATTACCTTTTAAAAATGGACATGAGAATT
TAttctaatattttaaatcatattcccttaaaaaaaaccaatatCACTTGAAACTTCaagataaatatataaaaatgc
cTTCTATAatcaataaatgaaacaaattccATTTTAAGAATGTCATAATAGGTTACTTGGA
AGACTGATTTAATTCCTTGTAGCATTAAATTTTGATGTATGTATAGTGATAAA
GCCTGCTATCAAAATTACTATTATGTTTTACAGCAGCAAGCCTGGAATTTTG
CTGGAATCTCTCTGCTGcttgaaacaaaaaaagcattaaatctGCCATTTCTGTCTATGTGA

AATATGACTCATTCCCCAAAACAGCTGCCAAATAGGGatcacacttctttttctttaataag
cTTTAATAAACTCAGCTGAGAATCAGCATGGGACAGACCTTACACGGTCCT
GTCTGCTGTATGCCTACTTAATACACATATGCTACAACTAAGTTATGGTAACA
AATTAGAACACTAAAGTAGACACCAAGCTAAACCACAAGCTACCCAGAATT
TTAATATTATGCTTAAATAACTTTGAATTGATTTTCTTAGAAATCAAGGCAAG
AAAAAAGTTGAAACAAAATATCCCATTATTGAAAAGACAACATAATATGAG
AGCAGAAAACACTAGATACAaaaaatacctttatttttaaacagtttttagTAATCTGCAGTACT
TCTTTAAAGATGATGAACCTAGGCATCTTCAGAAGTGAGCCAAACAAATAA
AGCCTTTAGACACACACATCCACTTCATGACTTAAGAAACATGACCATTCC
CAGCCACCAGACGAAAGTATTTCACTTTACAAAGAAGTTTCACAtggcaaaaaa
atgttttaacatgtaacatttctttttgtttgtttgtttttaaataaatcaactTCTCTATTTAGGATGTAGAACTT
CTTCCAGACAACTCACTTCATCTCAAAATTGCCACAATTGAGCATATAATTA
GTGATTCTTTAGAAGAACATGGGAAATTTTCAAACTTCCTTACTGCAAGAC
TGTTTTGTGTATTGTTGCTGTGTAGCCGGCTTAACTGACTTCTTTAAGCAAA
ACAGTCTGCTGACTATGAGCCAAGTCACAGTGGTTTGAAAGCACAATGTCA
AAATGAACTCCAAGAAGATTCTCCTACACATCTGTGCCAAATTTTGCAGTG
CTGCTTAACAATATTTTCAACTTAGTTATTCAGTATTCCCTATCTAgaggtcaggaa
aaaaaaaaaaacaacaacaacgaagcagagaagagatgaaaatacCTATAAGTATTTCAAGCACAAC
ATTGCTATGACTTCAGCATCCATGCAGACCAAGAGTTCTCAGGTGCTTACCA
TGTACTCATCTTTCCCTCCAAGAAAACATCTTCCATTAAGTTTTTGCGTTTA
AAATGCTAATTATAAAGTAACTGCTCTGCAAGTTACTGGGTACCCTGACCCT
CCTCCACAGAAAATTTCCATAggtaataaaaagcaaagaattgtCTTTAGGACAATCAAA
TATTTAGGTGAACAAGTCAACTTAATCTGGAAAAGATGGATGAGGAAATAT
TTAGGGGCACAACTTCTTCAAACTCAAGAATTGCTTAGAACACATTATTTCT
CTAGTACACATTCCCTTAGCAAGGTGTCGAAATAAGATATGATTTATTTCCA
AGCTTAAAAGCTGCAGTGACCAGAATTCTTTAGAAATTCATGTATTTATGAC
AGTACTGTTGTTGTACCACTGAATTCTCTATAGTCTCCAGCTAAATCCAAGT
GCTTTTAATCACTGAAACCATGCTAACACTTTGATACTCCTTATCAGAATAA
CTGAAGAACTAAAAGTCAGTTGAAGCCTAGAACATTAACCATGGTTACAAC
TATATTGACTTAAAATTCAGTGGAGACCGAGCCTCTTCCTTTGAACGTTTTC
ATGCACTGTATGAAATTCTACATCCTACCTCTTTCAAGTGCATTCacaatatattaat
atttcCTTGGTGAGGTGTACCATCAGATGGCACATATGGGAACTGAGAATTTTT
GAAGTGTAAGGCTACCCTAACTTAGCAATGTGCCCAAAGACAGAGATCACT
GAAAATCCATATCTGATGAGGACATATGAGTAGAAAAGCATTCATACACATT
AAATTGCatagactgtttttttcttcaacttctCAGTTCCTAGACAAATGCATCCCCTGCCCC
CATGGTCAATGAAGGAGCCTAAGAGCTGAATGAAATGCGGAGTGCAAGGT
AACCAACAAAGTTTGAGAAGGTTCCCCTAAATCTCCACTGTTGATCTGAGA
TGGCCATTAAGTATTAAACCTTTGTTAACTGCTCTAGTAGGTAGAGAAGTTG
CAAACATTGGAATAGCACAGTTATTATCACGACAGTACCGGCAGCAACATT
ACCCAGGTCCAATAGGTGGAAAAAGTACTTAATGCAGCATATCAAATAAAC
CCCAACCACCTTCTCAAGATGATGAAGGTAACTTTCTTCTTGCTTGAAGTA
AGCAAATGGAAGTCTACTATATATTCACAAGATCCCGGCAGGTAACTCTCTA
ATTCACATCCTATTATGTGTATCCAACGATACTTTCCTAGATTTTGaagttacacaca
cacaaatttgtttaaaatgtgaCCCACTAACAAGCTAACATCTATTCCCTCCATAGATGTAT

AATCATATAGTGATGAATATTTATAGAAATGCCTGCTCTGAAGCaccaaaagcactgt
tttctttcaaaagaatcAAAAGTCATTAGGTTGTACACAATTACCGAGCCCAAAagttgca
aggaaaaaaaaaaatttgaaatcTGAATGTGTCAGGTTTTCCGGACTTATGTTTTTCACTA
AACACCCAGGTTTTTATTAATTCAATTTTcactaaaatagaaaaatgtcaCTGTTCCATAG
GGTAATTCCAGGATTTACATACTACCCCTTCAGACTAGCTCTTCAATTCTCTG
CAGTTCATTGGCACGTAACCTCAAGAAGTCAGTGCTTGTGTGGATACAGAG
TTCTGCTATCCTGCAGTTAGAACTAATGTTAGAGCCTCACTGTTTTCCATTAC
TTACATTAACACTTTGTGGGCAATTTTAAAGCATGCTTTCTGTAAAGCTGatag
tattttaaaagaatcttCCATGTGATTAAGATTTCTCCCTGCAAAGGCAAAACAATAAC
AGCTCACCTCCCTTACAAGAGATCCAACAGAGGAAACTTCTAAATCTTAGC
AGATAAAGTTATTGGGTAAcaattaagtatttttctcctttccatgaTACGGATCTGCGACACA
GACCAGGAAGCTTAATAGAAACTGCAATACCCTAGAGAAGTTTACAGTCTA
GATACGTCCTTTAATCCTTTGCATACAGAATGTCAGAAATCTACTAGCATTCA
TACCTGTGTTGTTGCAACATGACTCTGGAACATGCTGACATTAAAGAAGCA
TCCAGACGTAtttgctgaagtcagtgaaagagtgtttttttaatttacaactCCAACACAGCTATTTATC
TAACAAGGTATTACTGATGCATGTAAAAATAATCATGATGCTAATTACTGAA
CAATCTAGTTAATactttgtattaaaaatgttttgtaggAGAACTGAGTTCTAAATGAAAATT
AGCAGTTTTACATAACAATTATCACAGTACCCATCGCTCATTTCAAACTTTG
AATTCTACTGCTTGTATTACATCCCTATGGGAACCAGAAATTTAAACTGTATT
CTCATTAAATCCCTATTACCGGtgtttaaaacatatttttcatctCCAGTAACGGTAGTCAG
ACTCTAAAAGCCCTGTTATGCTTTTGTATTCTAACAAAAACTGGAATATTCTA
TAAATCACTAATAGCATAACTgcacaacacagaaaaagaatatattcaaGAAAGAAGCTTgc
cttcataaaaaaaaaccaacacagtcTAATGCCATTTCACTGAACTTCAAAGACGCAGTTT
CTTTTGAGTTCTGAAAAACATCAGGTTAGGCTGCAAAATTTCTCACCATTCA
TTTCTGTTATCAGAAAAGGGAACAGCTGTACAAACTAGACTGACTTAGCAG
GTAGGTCAAGTCACTTCTCATCTATAAAGCCCTACTGTTACTCTCACAGTTA
TTTTAAATCCATTGAGTCTGAATATGATCTTAACTGAGCTGTTcaacagcaaaaagc
atttGCCTAACTCTTAGAATCTATATGAGGAAAAACTAAGCCTCTGGAGTatcaga
aactgcagaaattccCTACCTGGACAAACTCAATgtagggaagaaaaacatttaagtgTAGAGCCA
CCTGTTACATGTCATTCAAGGTGAAAGTGGAATACAAAAGATTCAGACTTT
TGTTTCACAAGTCTTATTTTCTCAAAAAAGACACCGGAGTACTATATTTGGG
ACGTCTTGAGTGCATGCCCAAAAGGTCTGCAAATAAATGCTGATATAGCTCT
CTTGCCTTTTTCCATCAAGTGGAAGACAAGTTCTTGTCTGTATcacattaagaaaga
aacagttaGCCAGTACGCAAGCACTGAGTATCGGAGTGTTTGCTGTTCTAAGCT
TATGTACTTCCTGAAAGATCTTGCAGTTGGAATACTTAATAGTTTAACCAGTT
AGAGCAGCcaggagaaggaaaatttcTATGCTAAATTCCTTAGAAGTATCCTATCCGA
GTTAACACTAAGTAAACTACTGTTTACTTAGATGTTTACTTTGATAAGTGTTA
CACTGAGAACAGTAAAAAATTCTGATGCTGGTTAGCTTTGTCTACAAACAG
TTCCAACCCTCCctcctcaaaaacaaacaaacaaacaaaaaccccacaacaacccaacaacaaaaacaaacc
ccaacaaaacattttcaaagctaACATTCTGTTTAGGTTCATTTTGAAGATATTCACAGTT
CTTCATTCAGGGTAAAAAAAGAGTTAACTACTAAGTTCTTCACTCTTGATTG
CAGCAGGTTTcaatgctttcatttaaatacatgATGTTAAGTTTTAATACCTTTTTCAATAC
AGgtaaaatgaacagattttaCTCCATTTTGTCACTGCCAATAACAGAAACTTCAGTGT
TCATACAGGTActctttgcttctctgtgtATCTTCTGTTAGTGTAGAGTATACACAGTTC

AGATTTCACTAAAAATTTCTTCACCAAGTATTTACTTTATATTTCAGCTTAGA
AAGATACTATGTAACTGGAAAATTTCTTATATGCTGCTGCAGTTACAAGACT
GCACATAACTTCCTGCAATACTGATTTTCTTGGCAATGTTTTGAGCTCAAGC
AGGTGACAAGCAAgcaataaaattgtttttcacaCTATTCATATGGATGCAAAAGATTTTA
TACTacaattttaaacaaatactgCAGAAGGTTATTCGGGAATTATTACTAGTACTTACTA
TTCCAGGACTAAAATCATCTCAGTTGCAGTTTCATAAACTTCATGAAGGTTA
ATGACCCAAAGATTGCATTGTGCCAATTCAAGGACTGCAATTTCATGGATTA
TTTCCATCCGACAGTCTTggccctttcttctttttctcatgaATTTTGCTGCAAATTCTCTTT
CGGTGTCTTTCTGGATACATTTCTTCACCACTGCaaattttcctctgaaattaaagaagaatTT
TAAGTTAGAACTAAGTCCCTTATTGACAACCTTAGTTTAATCCGtgtaaattaatattt
cagaataCATGAGCTAAATCACTCTTAAGGGACATGTTTACCATGTGCTCTAAA
ATTAGAAAGATGCCTTGCTCAGTGATAGCCTCTAAAGAGTTGAAGTTTCTG
GATTCTGAACATTCTGGATTGTGATGTTCTTATCTCCattaaaaataaggattttaaaacaga
aaactacaACAGCAAAGTATCAGTTCAAGCAACTTCAAACcttcagaaatacagcagaaatg
aaCTGTGGTAGTTAGGCAGATTAAGATATCCTCGAGCCTTCCATAAAAAGCT
AGTAAGATGGTATATAAGCAAGGAAATTATGCTTTCTGTGACTATACTTAAC
CAAACGAGTTGCCTTGAATATAGTGGTAAATATAGTAACCAGACaacagcagaca
gcagcttCATGGAAAAAGTGCTCAAGATCCCACTTGATTTATGCTACGGAATAT
CCATactcttctctgtgtttttcaaaaatGGCACCTCATGCTTTCACCATGACATATCTTCAAT
GACAACAGCTACAGTTTTGACTGCTTAACAGTTCTCAAAAATCCCCTATTGT
CTCACTGTTTAAGGAAcgcaaaatatttcaaaaagtaaGGAAACTTGGCCAATGTAAAA
CATTCTAACAGTCTTTGCAGTAATGACATCGTGAATACAAACATACACTCTG
CAGAACTGAGTACAGTGCTTAACCCATGTTAATGAGTATCATTTCTGACAAA
ACACAGTGGATGAGTCACACTAAGCCAGCAGTCCAGCTAAAAACATGCTG
CAGCACTTTTGGAAGTCTTCCCCCACAGCGTTTTGGGCTGAAGTACCTGCT
CTAGACTTCCCAAGCCACCTGGTCCACCCAATTCCATTCATATAGTACTGAT
TTTTAGAATGAAAAGATCTATGAATTAGCGTATCATCTAATGCGATGATGAAT
CCTCATCCATTTAAGTATAGTAATCCTACAAAATTCTTACCATACAGGTATAA
TGTTAGACAAAATGATCATTTTTACTTCCATGACAACATTTTCTTATAAAACT
GCAGGAATAACTTGGAGAGTCTTCACTCTGTTTCCTTATATAACTTATTGTA
GGGTAGCTGAAACTAATAAAATTAGAGTCCTCATCTCGTTTGGAATACAATT
ACTTGAGTCTTGCCTTTCCTATGTCACAAGCTGAAACGTACAGAATTAACTA
ATTCAGCTCCTGTCGAAGTTTTCACTCTCCCAGCTCCTAGCCTAGTGACTGA
CTGCTTTCCATGAAGAGCAACTGCTGAAAGCATTTGCGGTCACCCTGTTTAT
AATAGAAATTTGTTTCAAAACTGCCCAGCTGGAATTAAGTAATAAGCATCC
ACAAAGTGGATATCTGCTAGTCAGGCTCATTAAAACAATCTTATTATTAACT
ACTGATACAGTCACAGTTATTCAGGAATGACATTTTGAATAAGCACAGAAC
AATAGGTATGAGAAAATAGATCAGAGCACACCCATACAATAAGGCTGACAA
ACAGCAagaatatgctttaaaaaagctttgcaaatgcatgaaaatacaattgtattttgTAGCACTAGAAAG
GTGTAGTCAAAAGGTCATTATAAAATGGTTTAAATAATACAGATGTTACTGC
AGAATGCCATGGGAAGAATAGTCACCCAAAGCCTAACAAACAGCTATGTGC
CTGCTACACATCTAGCAAAAAATACCAAATGTTTGTAGTGTGCACAGCTTAT
CAAGAACACTGAAACATTCCAGAGACTATGCACTGTTGATCCCCAAAATAT
TGCAAGGATGgtgtttcttttaaacagaGTTCTAGGAAGCCTGTGATTAGTGTTGTGGC

TCTAACAGTAAACATCCCTCCAGTACTGCAATCCTAACCACAATTGAGAGC
AATCTTCCTGATGTAGCCAGGGAAAAGGCTTTTAGTTATTCCAAATGAAGA
TGTGAGACTTTATGACTTGTGTACGATGTAAAGTTATGCATTTATCAAAAGT
GTTCAAATTGATGTTGAAGCTGTGTTTCAGCACCAAATCCAAATAGCGCAC
ATggagaaaagtaattaaaaaaaaagagagataagGGAACAGAACTTGCTGTTAAGGATCTTA
TTTTAATACCTAGTAGATATGCAGATACTGTGCTTTTATACCAAAAATCCAAG
CCACAGAGTTCATCCATATCTGTTTTTAAGTAGAATTACTcatacagaatcacacagaat
ggcttgagttgtcagggaccttaaagccctTCTAGTTGCAACCCCTTGTTGAGGGCAGGGAA
ACCTCCCACCAAATcaagctgctcagggccccatccagcctggccttgaacatctctcTACTCACTAC
CCATTGaatgaagaacttcctcctaatctAAAACTCCCacctttttaatttaaaaccattctgtCTTTATCTA
TCAGTCAGTCCCCCTTCTGAatgtaagctcccttcaagaactggaaggctgcagaggtctccctgaagcc
ttctccaAAAGAATCAAGCCTAGCTCCCTATATGTTCCTTCATacaagaggtgctccagccc
tctgatcatctttgtggctctcctccaacagctccacatctttctcaTGCTGGGgtcccagacctggatgcagtactcca
gatggcACCttacaaaggcagagcagagaggggaccatcacccccccgccccccccctccctccttgtcctgctggcca
cccctttctcttttgatgcagctcagtgATGCCTTTTTAGCCTCCAAATGTacagtttctttctgaaagaatg
TCTCTCTAGCGCAGagcaaaaagcttttaattCTAGCACCACTGAAGCATCCAGTAAA
AGTAGTTCAGAAAAGTTTAAAATCTTCTAATTGCCAGAGCGTTGTTTTGAAT
TACACACAAAACCTATCTGGAAGTAAGCTAATTAGCCTAACGAGCCCAATG
ATGCCAATTTATCACACAGTAGTCACTAcggaaataaaacagaattcaggtaagaggaagaaac
agcaaGAGACCACAGTGTGTGGCCACACTCATGCTGTTGACTGCACTGGACA
GAAGAAGTTGGCTAGGTGGTGGAACTGAAAGGACACTGCACAGGAGTTTT
GCTGGCTGACAAGTATTTATTACAAGTTGTTGAAGGCTAAAAATCAAGAAC
ACCTTGACATTATACCTATTGTTATTATAATACTATAAAGCTATTATAATAACTA
ACCACTGTCTGTGAAACTCACCTTTTCTTAATACAAACCCAGAAAAGATCT
GACTTGACAAACCCGATACTTCTAACTAATAGAACTAATTACTGTGCACCTA
CCTAGATTCACATCTGAGACTATTTCAGAACCAAAGATACTGTCCattgggggag
ggaggaaatcAAAGTGAGAAGTTAAGCCAAGAGATAAATTTGTAAGTATTTAATT
TACAATACAGACCATGTagcagacagaaataaaaacagatatgAAAAAGCTTTCCCAGAA
AGTACACCCAGATGGTGCACCTTTGTGCTTCTTAAGATGTTGCTTACCAAAA
TTCCTTTCAAGAAACAGATCTGCTAATAAATTGTGAAAACAGTATTAAATGT
CAACATTACCAGTTTAGATATGTTAAGAGGAGCTTAAGGGAGAAACAGGGA
GAAAGTTACATTGAAGGAAGAACAATTTTGAGAGGTAATACTTCGTGTTCC
TTTCCAAAGTTAAAAAGTGACTATCAGTTATTctaacaaaaatgcttttaaggGTGACTT
AAGTAGGAAACAAGTTGTTCTCAGTCTTCACAAGAGCCTCAGTCAAAAATT
ATGATTTAAGATATCATATCTGATCACAAAGAAGCTTAATGTCATCTTTCAAG
ACTGCCAAAAGAATTAGCATTATTTTATCTGGCACATAGTATGGCTTAAAGG
AAAAGCCTTCCATCACAAATCAAATGTCATATCAGATAAACAAGAGCATTTA
TGTCACACCAATTAATTCTGGTTGTAATAGGCTCAGGGAAACAGAGAACTA
ACCTTACTGTAATAGTTTTAAGATGAGCTAAAAATCACTGTCACTACTACAG
ATATTAGCaactatttttaatatgtttattttacagaaagcttagaaattttttttctgagatCCATAACACTATATG
CAATTTATTACATAATTCACgtcaaacattttttcttgggggtgattttttaatacaatttttgATTTCCAA
AGAGCCAGGTAAGGGGAGGTTCCACTTCTGATGTTAGTGAACATTTCCTCA
CATAAGTTTGTTCTCACATGGTGGAATCAGCTACTTGCAAGTCTTGCCTTCA
CCTTCTCATTTCCCCAACTTTTTCACTCAAGCCAACAGCTGCTTGGAAGTG

TGACAGCATAACTATGCATTGTTACTGATAGAACTGCACAAGAATCACATAC
AACTAAGATCTGAACTCCTGAAAGTTACCTGAGAAGTAAACAAGTTTATGC
CAGAAATTTCGGAAAACCAATTCAAGAGCTAGGCTACCTTTCCATGAAACC
AtagtaaaaaatgaagaatatatGATTATTCTGTTCAGTTTCCTCAGATGACTAAAACAG
TCATTTGTATGTCTGGGTGAAGAACATGCTtgttcattgaaaaaaaaaaagtggaatgaGGA
AAATCAACTGAATTGAACATGATATACCTTTATGTATGCACTGCACATATTTC
ACTGGCAGAAGATTGCTTGGTTTAGAGAAGTAACTTAAGAATACTCTtatgtttc
agagaaaaagcagtgccAACTGTTCAGGCATTTTTCCAACTCACCTCTAGAACCAGG
AGTGTTAGTTTTAGTACATAAATGACGGCTACCAACATGtgcatagcttttttttttttttttaa
acattaaaagaGTGCAATGCCACATCAACAACCTGAAGAATTAATAGCCTAATGGT
ATTTTGTGTGCAATTATCTAGGGGGTATTTTGTTgaggaaaggaacaaaatatGTGAAT
ACAATTATACTGGACACTTCAAAGAAGTTTTAGGTAACGTGAATCAGTGAC
AAGAAATTTTGATTGAGGCCATGTTTCTGAAATATGGAGCTCAGACATCCA
CAGGGTGTATTGTGCGCATACTCAGAAATATGCGTCAAGGCTGTCAccacaaaat
aattaaagtatttttccgaattttacagtaaaattatGCAGTGTACAGATGATAAGGATAAAGGTCA
AGCAACTTTTGCCTTGTCTtgcacaaagaacacaatactTTGGATTAGCTCATCACAAA
TGAGTACTGCTGAAATGGTTTAAGAGCATGCTGACCCTTACAACATTAAGC
AAAGAAAGTTCAATATCAAACACCATGCAATGTCAAATGAAGATGTATCTG
TGAACATTCTGATTTGGGGACAATAAAATCCATATGGCTAAAGACTAGGCC
AAATGTCAGCTTAGGCAACTTAAGTTTTCCATCCAGAGTAGCCATGCTTTTG
GTATTTCCCCTGAATTAACTGCTAGAAAAAGCAGGTTCTATTTGGGTATACC
TGCAAAGGTCTGTTCAGCATCCAAGCCAGGCCTATTTGAGTACATAAGCTTT
ACATCCCTAAAGCCATTTTTTCCCGCAAGCAGAAATAACGAAACCTGGTTT
CCTGTAGTCTTGTACCAAAGTTGTTCCAGTGACATCACAGCCAGGTTCCTC
CCCTGCTACCCTGTGGTTCTTTCAGCAGCGTTTGTCCAGGTCTGCCACCCCT
GCCAGCCACCAGGCCCTGCATCTCTCCTCCCCATTTTCTGATCACCATATGG
CCCCAGCTGTGTGCCTGTGCTCCTGGCCCCTGCCCACCTCCGGCACATGCA
GGAATGGGAAACAGAGCACAGGCTTTTCCAGCAGACCCACACTGACAGCC
AGGCTGGAGGCCAGTGAGCAAAGTGTCCCAACAAGCTTATACACTCAGTG
GTTGGGCCAcatctgctctctgctctctcctTCAAACCCAACATATACAGTTCACATTCTC
TTCAGAATTCCCATCTCAAGTTCAACTTTTAAGCACTTTCCTGGTTCTGGGA
AAAGAGATGGACACAGAGCGTATGAACTTTCTTTCCATAGCTGTTTCCTGA
GCAAATTCAGCTGCTAAATTAAATGAGCTCCAAGAGCAAGGACTGCCTTTT
TTCTTGGGTAAAACCTTGTCCTATTTGGTTGCTAATTTGCCCAAGCCCTTTG
CAAAGACAGACCTACTCAAAAGTAAGTGTTTCCACACTCTGCAAgcagataaaa
gcaaaacaatttctGCTAAGTGTATGTTATCAACAAAATGCCATACAAAGAGACAGC
TACAAGTGTCAGAGTGACTTTCTATGTGCCTTTTTAGTTAGCCACGACAAAT
TAGCAAAAGTTTCTCTTCTTCACATGTGTGAAGTACTCACTTGTTCTCAGAC
AAACCCAGTGAAAGAGGTATTTGAAATTGAaggaaaaccaaacacacacGCTCCTAA
GTATGGACTTCTGCACTGGAAGGGAAGATGAAAACCTTTGTGGAGGTAAA
GAGAGGAAAGACAGTTGGAACTGAAACACATTTTGATACCGCACAAAGGC
AATGCTACCCTTAGAACAGAGATGTAACTCATGACACAACTACAGGTGCAG
AATCACAGCCATTACATAAAGATCACCTTTAAGTATAGCTCCAGCCACTGGT
GACCCCACACAAATAGCCATCCTGAGAACTTTATGCTCAGGGTTTGCTGT

TGCATCCTTGCTCACAACGCAGAAGCAGTCACTCCAGTTGTGCCTGTGTGT
GCTAGAAGGAACCCTCACCAGTTTTTCGATTAGGCCAacagctgccagctcccagtGC
ATGCTCTTACTTCAGAGCAGAGTAGCAGAGTTTCTGAAAGCCACTGAGCTT
TGAATTTAAGCCACCGAACCGAGTTCAGTATCCTCCTCTGCCACCTGCTTAC
TGTGCCTCCATTGAGCTGCGGGACTGAAGGGCGCTGCTCGTGGGtactgcagctg
ctgggctgaaCGCTAAACCAGCAGGGCGGTGTGCCACCGTCAGGCCCACCGCAC
AGCTGGGCGCCTCGCAAAGAACGCAGATTGCTCATGTTGCAGCACATCCTG
TGGTATTTCACTTTATCTTCGTGATTGGAAAACTCGTTAGATAAAGCACGTC
TAACTCCGAGCAAGggcactgtcatttttttccgGCAGCGACACAGTTTCAGATAGAAC
CACCGGTCTGGCGGTGCTAACGCCGCGCGCGTGCCCACCCCCGCGTGCAG
ATATGGAGCCCACCCGCCCTGAGCGCGCGTGCCGCCAGAGCGCTATTCTAC
TCACCAACCCAGCCACGGGTCGGCTGCTCTCACATCATGATGTTTTAACGA
ACCGGCGGCAGACCCTGCCCCGGACCGCTTCTTCCCCCTCCCGGTGCCAG
ACACAGCCCCGGTGCCCAGCCCGGCGGCGGCTTCAAGCGGGGCTCTGCGC
GCTGCAGCTGCCCTCACAGCTCCCCAAGGTCGCCCCGCACAAGGACGCGG
CCGCACACCCACCTGCCGAGCTCCCTGCCGGGGCTGAGGCTGTAGCGCTG
CTGGAAGGGCTCGGTGCGGATAGGCGTGCGGATCTCTGTCAGCAAACCGC
CTCGCCTTCGGCCCCGGCCGGCAGCAGGCTGAGGTGAGCACTCATCGTGG
GAGCGGCCGGGCGGCGGCTTCTCTTCGGGGCTCATAGCTTCTCCCGTGACG
GCAGGACGTCTCTTCCCGCTACACTACGCCCCAACTCAGAGCAGCTCCCAC
CGACCCAACACCCCTCAAGGGAAAGAGGAGCGCAGTACGCACGGCACAC
CGCGGCCGACAGAGGGGCGTGACTAGGGGGCCCCGCCCTTCCACCCTCAC
CTTGGCGGGCGGGGGAGGGGAGCGCGGGGCTGCACGGCGGCGGGGCAGC
GCCACCTGTCGAGGGCGGAGGTGGTGAGCGCGGTTGGAACGAGAGCTGG
CCGCCGGCGGTTTTGATGCCGGAGCGTGTTGTTGGCGCGGCACCGCTGCA
CAGGGATCTCAGTGGCGTGGTGAACCCTCACGAGTGCCTTGAGGGAGCCG
TGGGATAGAAGGGCGAAGGCCTGAGGGTAGAAAATACCAGTGACATCAGA
AGCCCAGTGTCTTAGTATAACAGAAAGTTGGATCTGTTGCACTTTTAGACA
GAATTATCCGGAGGATGTgacaatgaca
>TCONS_01702097
CTATAATTTCCTGTAGCAAATTCTCCAGAGAGGTGGCTGTGTACCTGTGCCT
GACACAAGGCACATGCATTGCAGGTGAGCCATAGCACATAGTGTAAAACTG
GAAATCTGGGAGATATATGTATCTGAAGCTGGGTTTGCCTGGGTGAAGCCTT
CACAGCCCTTCAGGAGTTTTGGAGTACCTGGGCCTAGCATGAGGCTGAAGg
tgccctacagaaaaacagaagtctgGCATTGCTTCCTGTGAGAGCCGAAAGCCTGGAACT
GGAGCCAAAGGTCTAAAATAGAAATAGACAACATAGCAGAGTTTGTTGGT
GATTAACATAAACCTTCTGACAAAGTTTTTAATTTCGGTCAATTCTAAATAA
AATAGCATATGACCTAAGAAGCAGTGGAGAAAAACTTGTGGGAGAAAGAG
CATAAGTTGCTTTTTCCTTAGTAAGCTTTATCACAGAACTGACTCACTGCCT
AAAACTGGTTAAACAAGGATGCTCAGGTGTTCAGGAGGCAGATGCCTTTA
CAGGTATCTGTTTAAGGAAAATCCACGcctttcattaaagaaaaaaaaagaaaagaaagaaagaa
agaaaaaaggctgttcTACATTAATGGGCCAACTCATCTTATCCAGAAAACTTTGATC
AAAAACTCTGGTGGTTCTTCAAAGCTGATCCTAACCACTGTGGGTGAAAA
GCCAAAGAGAAAATTGTGTGAAGGTTTTGTTACCTCCTGATTTTCCTTGAG

CTGGAAATGCTAGAAGAAGGGCTTTACTGTTTCTCATGTTGTTGTATCCCCT
GTTTCTCATCACACTTAAGAGACTTTTGTGAAAAGAGGATTACAGTTCATG
ATTGTGAAACTTTTGTTGATGAGAGAACATTACATGAGCAAAAGGACAGTC
TTATCTGTAGGTGGTATTACTATGCCTTCTGTGTTTTTCCACCATTAGCATAA
ACTTGGACTCAGTGGTGAGAAGTCCCTAACCTCTACTCTTTATCATAACTTC
CCTTATCTTATCTGCACTGCATGGAGGTCAGTGATGTCCATCATTTCACAGG
ACTTGCCTTTGTACTCTGGTTAAAAAGAGGATGTTAGGTAGATTGCAGGAG
TgctttctgttgcctttttctTAAACGTTGTGTTCTCTACCCCCTGCCTCAGGTACACACT
AATCTGAGAGGAGTGTTGGTTGCTAATAGATAGAGGCAAAGCTGTACCTGA
TGAGCCCCCATCCATAAAGTCAAAGAGCACTGCTGTCATACAAAGTCAGTC
AATTTACAGGGGAGTTTAAGTGCTCTCCATTCATCCAAAACTATCTCAGAAA
GTTTTATGAAACTCCACAAAGCTGTTGTAGGATTATAGTGGATGGGAAGATC
CAAGAAACTGGGTGGAGTGGTGAGAAAATGTTAAGAATAGCTGGCAATTA
ATCTTAGCTCCTGGGACTTAGGGACTTTCCAGTGCTGGCAGGTCTCAGTAT
GCATGTTGTTATTCAGGCTACATTCCAGGAATGCTTAATGCTGACTGGTGAG
TGTGTTGGAGAAGTCATTGTACAGGCACTGTCACCCAAAGGAAGCACACC
ACACCATAATCATTGGTTTAACAGTCATTTTGCAGACAATGAATGCTGACAT
TTGGACAGTAAATGGAGCAAACAGTAGAAAAGGGCGGGTGGGTAGGGAA
ACCTTTGTCTTTTTGATAGTTAAGACCATTAAATCTGTTAGCATACATTGTCT
GTACATGGGTTCTGTGATGCTGTTGCAGTTATGATAATGACAGTGTTGTGAG
AGAGTGCTAGGGCATGTGGGGAAAactgaactgaactgaaaagaGTTTCAAAACTAT
TGAAATAACAACAGCCTTTTGTAGAGAAAAACCACTTTGTCTGTCCTGTAA
GATTGtacttaaaaatactgtaaagcAGGAAAACCAGGAATGAGAAAAAGGTGGCAGG
AAAGGGGAAGATGTGGTTTAGGGGGAATATAAATATGCGAGTTACCATGCA
AATTTCAACAAAGTCTGGActtcagtctttattttttataatacaTTACTCATtagctgtgacagctgtg
ctgtgccctccctgctgcagtaAATCAGTGTTATGGAATAAGCACAGAGAACTGGGATG
AGTGATGAGACCAACCAAGAGAGTGAAGAGAAGGGAGTCTCTTCCTGACC
CAATCCAAGAAAAGAGTCATAGCTATGGCAGGCATGTGTACATGTCCATGC
TGCTCCATCAGGTCACTCCTGGCTATCCCCTGCTCATTGTCCCTCGTGTGTC
CATCCAAGGAGTTGCCAAGACCCGTAGTCCCTGGGCCAAGCAACCTGTAC
ACTGGATGGTCTGCTCTGGtgctcagcagcaaagctgacCCTCCCTCATACCTTGCCTC
AACCACATCCCACCTGTCACCCTTCAGCCATCCCAGTATGCTGAGGCAGCT
CCAGGTGAAAGGAAGAGGCAGgaggcagaaaagcaaacacactgCAGCAAGAAGCCG
CCTCTCAGTGGTTACTGTGGAGCCAGTCAGCCACTTTGGAACTTGATTTCC
AGATTACTAGCTAGTTACTGCCTCAGATGTGCAGGACAGGATCACTGCCAC
ACTTTGTCATGGGCCTTGAGGGAACACACATAGTGCTCTAAGACTCTTTGA
GGAAAGGctcaattaattaattaatcattatgatttttttctgtcagactGTAAAGTCTTGCTGATCATT
ATggattatttttacattctaGTGCTGGTTGCACCAGCTCTATATCATCAGACCTCCCAC
AACTATCTTTCTGGCCAGCATCCTTTTTCCTACTCTTACAAAGTAAATGTAGT
TTTCTGTATGATATTTCTGCTCCTCCCACTATGGTTGCAGCCTTATTCTAGTG
ACTTCAGGAAGTTAAACCATGTCCTTACTTTATTCAAGGAATGTTAGTGGTA
AAACATAGGTAAAGAATATAAAGGATGTGCCAGGCTCGGTTTGGGTAGCTC
AAGTTTGTGACTCATGGTGGTAGAAGTCATTTCAGGAGTTATATTAATGGTG
CTTTTCACTCCATCCTCTTTTGTCCAAGACATCCTTTCCCCTTGTTCCACTG

GTAATGAGGTTTCCTCCCCTGCTTTCCAATGATAACATGGTGCCCATCATCA
GTGTGATGGCTTACTTCCCAAAGACAACATAAGACATCTTGTACTGAAGGG
AGGGTTGCCTCTAGCTTGGAGAACTGCCAGCATTGGTCTTCTgaacagtatcttttttt
tttatccaccATTGGAATTCAATCTTTTGACTGctttcttctcccaaagagAACCTACTTCTGC
CTCGAAAAATATCACCAAACTAGAACTAGTGGGAAATTTTCAGACAGGTA
CATAGATGTGTCCACTCTGAGTGGCCTATCTGAAACTGCAGTCCTGTGCATA
TCACATGGTTGTATTGATGATGGCATTGTTTCCATATTCACCATACAAAGCTT
ATTCACAGCTTGCTCTGTGGGAGGTAGTCTCAAATATGAGTTATCTGGAATG
CCAGTTATGTGGAGCCTTCATGGAGGTCCCAGAGGGGCCAGAGTGCTGCT
GAGGTTTAATCTGCTGATAGAGGAGTGGATGCCTCTCTATTGTCACTGTGGG
TGGATGTGTTTTGTCTTCTGGGTCCCACCAGAGCGTGAGGAAACCAGACCT
AACAAGAAGAATGGTATGGGAGAAGCTTAGGgtaaaggaaaaaggatgaTTAACAG
ACTAAGAGGAGATTGGAGCAAATGGTCAATGCGCAAAGCCTCCAAACTGA
GATCTAGATACTGAACTGAGTGAAGGAATGAACAAAGTGATCACGGGTAGT
TCAAGTGGTAAACATCTGGCTAAATAAGCTTAAACCCCTCCTGTGCTAGCA
CTGCCCACCTCTAAGTATCTAACTTTCCAGTACAGGACTGAATTTGATTGAA
AGTCCTTTGGCCAGAAATTGTCACTGAAAGTAAAGGCATAAAACAAAAGA
GGGACATGTGTGTACTTTATGGATAATGTTAAACCATTATAAAGGCTTAGTG
CTTTCAAAAGAAGCAGTTACCTTGCTACCACTTCCTTTGCTTCTTCAATGAA
GGAGTGCAGGGAGTTATACTAGCAGAAAAATACCCTTTGAAGGTCTTTGTC
TCTTCAGAAGTTTGCACACTGCTAGGAGCAAGAGGTTGGAGAATTAGGTC
AAGAAAGAGAAACTTTCACTTTACATATTGGTCTGTTGTGGAAAGAGCCAA
ATGTGTCATGCAGACATGAAAGGTCCATTTGATATAATCAAAACTCTCAAAA
CTGTGTCCTATAGAAGCACTAGTGGAACACTAACCTCAACACACTCATTAC
TCTGAGAGACGGAGCTTTACTGTCGAAGGGTTGCAGGCTTGGCTTCAAAG
CCAAGGCCTGAAACAGGGTGTTTAACACAGGGAAGAGAAGAGCTGTACA
AGAGGGCTGAATGCCAAGAGTTAAATGTTATAATAGGagtatttttaatgtcaaaGTCC
TTTTTTAGGTTTCATTGTGTTTACACATGACTAATTTGTAGCACTGATTTTAA
CTTGTGATACATAGATAGCTCCAAAGCAGTCATAAAAGCATATAAAGTAATT
TATAGAGAACGTATATATAcattacatagaaaaaaataacaagtgTGGTATATAAAATGTTAT
GCGAATTCTTACGTAAGAATTCACCCTGTGACATCTGGTGTCATCACTGAG
ACAAGATGTTAGAGAGTGATAAGAAAGACAGTGATGTTACTAATGCTATTG
ACTAGCAAGAATAAGTAGCAGGAATTCATCCCTTTTGAGCCATCAAAGTCT
AAATACATGGTTAAAACCAGAAAAGAGTATTTGTGTCAGGGAAGAATGCA
AGGGAATGttgattttctttaatatttcctATGTTcagtttttgccttttattttcttacaaagaGGTCGGTTTGT
TTAGCATTGAGGCTGAAATTGCCTGTAATTTCCATGGGCAGCAAATTTTGCA
TTTGCAGTACAATTAGTAATATTATCCCACTGACATGCCTGTGAACTCAAAA
ACTCGGAGAAACTAACTTATGGGATGCACTGTGATAGGTAAATAGAATTGG
TGTTCAGAATTCAGTTACACACAGCATTGCCCTGAGGCTCTCATGAGAAAA
CCCTAGCATTCTGCGTTACATGTACAGACAGTGTAACACTATCAGAAGAGAT
TCCACAAGTGCATGCGGTAGGTGGGACTCACCCCAAGCTGAGGGTATCTTT
TAAATTAACTGCTGTTTCTGAGCCATTATATATGACCTGCTCAAGGATGTCTT
CAGCTTTAAGTGTCCTGCTGAGTAATCATAAAGGAGGAGGGAGTAGCGTTG
TTTAGGGCAGGAGGTTAAGAAGTAACCTGACCAGGGAAGAAGCCGAACTA

AAAAAAAGTTCAGATCTACAGCTGAAAATAGTATAACTTACCATTTTCCTCT
GCCAGTGGCTGactcatcacttttttttctttacaaccACAACGAATCATTCTCAAAAATGGA
AGTGTAATCCTCTGTTTTCTACACTTCTGTCCTTCCCCTCATCTGGGGAAAA
CCTGCTTTTTTCATGCTGCATTTCTCATGACACATTTGGCTGGAGGGTTTCA
TCACAACTTTTCTGCTGGGGGCTATTTGTTTTATCTTGCATTTTGTGGTAGAT
AACAGACCTACATGCTGCACAGATTAGACTAGGCAAATTTTGCCACAGATT
TCTTTAGGCACAGATAAAATGCCTTAGCCTACCCCTCATGTCTGCACTTGAA
GCAAAATTGTAAAATATCATGATGGTAATGGGAATGTTGAatgatttctgtgtttcctttatt
cctcaGCTGGCCACTTGTGTACACTAATGAGACACGCCAATATTAAAGGTGGA
AtttggagcagagcagaggccaACCTAATAAATCTGCTGTTAGCTTGGGCTCTGCAGTG
ATGTTGCTGTACcctgggggaggaagaggagcagctgagcgCTGGGTCTgagggacagaaaaggaa
accaTCCAGGGGAAGGCATAGAGACAGGATCTCTGTTGCTGAGggctgaaggcaaa
aaaaaaaaaaaatactttgtagAGGGAAGTGTTCTGAGGGAGCACTGAGGGCAAAAGG
ATGTGCAAACTTCTCTGTCAGCAAgtggctgggctgcagtgagagAGTTTGGAAGTGA
AAGGATCTTTAAACTTAAAAGTAGTAACAGAGACtgtattaataaataaattaggaggagaa
aggaagggttTGCTACTTAAAGTTTTTCCTCAGAATTTCCTTTAGAAGGTTGCCT
GCATTTCAAAGCCTTttccagaaagagaaagcaatccATTCTTCTTCATCATCTAGTATAG
CAGAGATTTTCTCCTGATCAAGCTCTAGCCCTTCTTCAGGCAAGGGGCAGG
ACCAGCTGACCCTAGGAGGTCTCTGCAAACATGACtggtgattccatgatcttGAGCA
CTTAAAGTCTCCACTCTGTTCACACCAATGAGAAAGTTACCTGTTCtagcagga
gagttggactcgatgatctttcaaggtccctgccaacccctgcaattctctGATTCCCATCGGTTATCAGTG
AAGCTGGAATCTTAACCTGAAAGGGAActgatttctgaaaatcatGGCCAATAGATGA
AAAGCAACTTATATGTGAAGGTCTCTGTAACATATAAGTTTGAATTTGGTGG
ATGTAGTCTACTGTAGCTTGTTTGCCAAGTTAGAGATGTTTGACCCCTGTGA
AACTGATGGATGATGTACCTACCTGCTTCCCCCTCAGCAGAAGTTCTTACCC
ATACAGCACTGCCCTGGTTTCAGATGagataattttcttcctgttgtgatgctgtgttttggccttagaag
aaaaataatgttgatagCACACCAGTTGTTCTAGTgttgctaagcagtgctgtacaggGCCACggatgt
ttcagtttttcagcttcttgtactgTCCTGCCAGCGGTGGGTACTGGGGGTCACAAGAAGCT
ATGAGGGGACAGTACAAGGATAGCTtacttaaactggccaaagggatattccatgccatatgacatc
atgcaaaatctttaaaaaactGTGGGGAATTGGCCAGGGGGATAGCTGCTACTTGCAGA
CTGGCTGGGGATCGATCAGTAGTTGGTGAGCAATTGCGTTGTGCATCATGT
GTGTTGCAAATATATAGgtatataattattataattactattatttttctgttcctcttctgaCTTCTTAAGTA
ATTTTTATCTCAGTCCATgaattttccagttctttttccttcccgATTCTCTTCCCCACCCCAC
TGGATGGGGGGTGTGAACGAATGGCTgcatgctgctgagctgctgctgagttaAACCACAA
TGAACACTTATTTATAGACTGTTTGATAACTCACCAGCGGAGCCAAAGATTT
GAGTTGTCACTGTGACATGTTGGCCTCTCCACTTGTTCATATTCCTTCCTACT
ATGTGAAAGAGCAAAGAATGCTTTAAGGAGCTAGATAATCACCGTTACATA
CTTCACTAGCCTCCAGGGCAGGCAGGTGAGCAGGTTGGAGCTAGCTGTGT
CTGTTCCACCAGTAAAGAGAGAGGCTTGGTCTTAAGAGCTGCAAGTCTGG
TGTTGTCATGAGCATGAAATCCACACaagtgtgaaagaaaaagactgcTGGAAGAGATC
AGTCATTCTCCAGAAGGGCCACTGGCTTGGACTGAAAACCCTATCAAAATC
AGAACATATAAATCTGTAGAAGTAAAAAGTTATAGAGCAGCTCTGAAACTA
TGATGAATTGGAGACTATGTGGATGCCAAAATCTTCTCTGAAAAGTCACTT
GCAAGCACTAAATTTTAATATCCCTTTCTCACTCagtggttgggtttttgttttttttttgtgccttc

AGTAAGAAGGCTGTGATTTGTAGCTATTCAGAGCAGTATGTTTTCCTGAAGT
TATTTAATACCAATTGTTTCTCTTCAGTCACATTCAGAAGAGACTTTGTAGA
GGGAAGACTAGCAAATCTCTGGAAAGCAGATCTGAACAGCTGCACCTCAT
CACTATGAAAGATtaggaagggatggagcctcTGGCTTTTGGCTAAAGTTGGCCAAA
GTAAATACAAAGTGGAATCTTTAGGGGTTGTGATGAAACTAAAAGTAGCAA
AGAAGTAAAGCAGTCAAATATATTCAAATCACCAGTTGAACTAGATATTTGC
ATTTCCTAAACCTGATTGGGTTAAGTCAGACCATCATCTGTTTTGGACAAAT
AGGATCTTTCATAAGTGGGATTATTTATATTAGAAATTATTCTTTAATGTGAGT
GGAGCTATTACAATCAAGTCCTATGTTTGTTATATCcctgttgttggtttttgttgttgtttgtttg
ttttcttgtttttttttgtttgtttgtttctcatttagACTTGGAGTTTCCTGGAAAAGAATgtcctttaaaatat
ataaagtaCAATTGATAGTGATTCCTCATGATCATATGTCACTTATACTAAAGAAT
TTAAATGTCAAAAGGAAACATTATGATCTTTATAGGACTGTTCCTCTTTATAT
GGTTTGCACTGAATTTAGATAAAGAGCATACATTCTCATGAAATACTTTAATT
GAGCCCAAATTAGCATTTCATTTGGCCAGCTCTACTCTAAAGTGGCTTAGCA
AACTCAAGTCACTGGAATAAGCAGAACTGTGGGTCTGATGTttcctactgatttttttttt
ttttaatcaaaaataaaagttagCTGCATGTTACATCGGTTGGTGTTTATACATTCTATTCCA
TTTGGAAGGAAATATTGAAATACCTCTTCAGCTGAGATGGCAATGGGTTTG
CCTAGAGTTTGGGGAGAATTTCTACACTCCAAGCTGCTTTCAGCACCAAAG
CTTAAGTACTGGGAGTAGCTCTCCTTCATCATCTTGGCTGGGTAGACATACC
TGGAACTCCAGGGTGTCAAATCCCAGGTAGCACTGTGCCCAGCTGCTAGA
CCAGCTAGAGGCAGCACAGACCACCTTTCATCAATTAGCCTTTAAGAAGGT
GGCAAAGGCAGCCCTGATACAACGGCTCAAGTGGCTGAGCAACACAAGA
GGTGGAGCTCCTTTAGCTTTCCCCAGTGGATGTGGACTAGAACAGCCTGAG
gacctttgctttctgcagcagcagcagcagaagcagcagcagcacactgtgaGAATTAGCAGTGCTTTGG
CAAAAGCTTATATCTCTACTGGGGCTAGCAAGACAACCAGTCCCATCCCCAt
gtccccctgtccccatcccacatCCCCAAGTCCTCATCCCCACAAACCCATGTCCCCCGT
CTCATTTTGGGAAAATTGTTGATTTTCTCAGACTACTAGAAGCATGTAAAGT
CAGAGCAGTTGTTTAATCAAAAACTTGACTGCTGACAATAAGgaagacagtatttttt
ctttttcctcaataaTGCTGTAATAAATACCTGGGTTTAGAATACACTTTTGTATTGTT
ACATACGTGTTGCTATAGACACTTTATGGTACAGTTCTTTGTTGCTCAACATT
TCTACAAGTATTTATTTACCATCATTTTTACATTGCTGCTGCATTCCTCCTAGA
GGGGGAATGAACCCATTTGCTGAGGACTATGTATTTCATGTCCCATTGAGCT
GTAGCACCTATAGTTATTTCTGGCCTCCTTGTACTTACATGTCCTCTCTGTCA
GATGTGTGCTTGAGTTATGATCGCCTTGCTGCATTCCAGCTATTATGAAGCT
GCTCTTGACTGATTCTCCAAGAACTGCCATTAAATCTCTCCTGAactcacatttcattt
ttcaaaccaCATGGGAAGACAAAGTGTGTTCTTTTTACTATTTATACTTGATCACA
CTGCTTAGGAACAATGCATGTCAGCTCTAGGCTTAAAGTGCTAAATAACATT
ACTTAATAAACACTTAGCTATTTTGTCCAAACCAACTCCTTCAtcaaaatgttgaaaa
attTATTGTACCACTGGGACAGTTCTGCCGTAGTCAGTTCtactcttctctcttctttcatttgt
gctttaggaaagaaaaacaatataatCATTTTTATCACTCAAATATTTGTTCTGGAGCCcggag
aagaaaaatagttacACATAATGTAATtgatatctttaaaaaaaaaacaacaatcttGCAGACATTGTTG
TATTAACCCTTCAGCAGCATCTATCAGTCCTTTGGCTGTTGACAAACAATGC
CAATTAACATCAGCGAGATAGTTAACTACAGCTCCAGGCAGTATTTTCATCC
AGTCTATTGCCAGTTGAAGGCtttcttctttacttctttACACCCTGGTGGTGCCAAATT

TAGCATGTAGAGACTGAGAAGTTCGAAATTCAGTGAGAAGAGAACGAAAC
TACTAGTTTTcttattattaaaacatttgATGAATAACTATAATAGTGAAGTGGCTTCAGT
TttctgatagattttttttttcccaattaacTTGCATATTTCaggatgctttttaaaaattccttgtcttccatgtggcaaatt
attctttcttaatACCTTGTACTTAGAGCAAGGACACTCTGGGTATGGACGTAACTG
GTGGTACAAAACCTGGGTAATTAATTATGCTGTTTGCCAAAGAACAGgcaaca
aaggaaagaagagcatTACTTCTGGAGATGCAGCAGGCACTTTATTAAGTGATTGTA
CCAGCTGGAAACAAatttttggctttatttttaaactacaaaTTAGAAGTTTAATGCTTTTACT
TCAAtgagaaacaaatatttaggAAGATTACAGCTGATGCATTCTTATGATTTTGACAG
GATTATGTTAGCTTCATACAATTACCCATGCTGATGTTTGCATTGGTTTTGAT
GAGACTCTTAGTACCTGCAAGTCTGAAGCTGACTCTTACCTCTCTTCTGTCC
GATACCACAACTAATTTTGGAGTGAGGAAGAGAACATGCACCAGGTGGGTT
ATGTTTCTAGTAAGTGATTTCCTTGAGCACATTAAAAGGAACCTCGCCAAG
AAAATATCACTGTAGCATTTTGAGTCCAGCTGGCATTGTATTGGAGGCTGTC
GAAGGAAGGAGAGAACAGCAAAGGCTGAGCCATGTTCATCTGTGGTGTCA
CTCAGCCAAATTTACCTGTAGGTAAATTTGACTTTCAGTATGCTCTAGAAAT
TGTTTTAGTCTGGGATTTTGTGGAATAAAAAGGCAGTTTGTGTGCACAGTT
TCAGTTGTGATTTGCACATTCactttttttccatctgaatgTAGTCCTGAAAGCACTATCA
AACACATTTCAGTTCTCTGGTTCAACTGCTTGGGTGTATAGGCACTCAGAAT
GTGAACATGCTGATGAGCAACAAAGTCTGGAGAAATCTCTAAGCCTTCATT
TCCCAGCTTTCCAAGACAGAAATTGATAAGAGGTGGTTTAAAGCACTAGTA
AGAAGACTGCAATATGGGGCTGTGCTTTGGCAGTAGGCCTGATTTCATGGT
CTCTACATCAAGAATGGCTTATCCACATATTGCAGAATGCTGACACAGTATG
AGCTCACACTAGCACTTCAAATCTGTTCCAAAATGTTGAGAGGTTGCTATTT
CAACTGCTTGCCTTACTGTCAAATCAAGTCATTATCTGTCCTGCTGCAAGCA
CAAACTCCATGTGCTCTGGCCAATCAAGTGAAAATGTGATCTTGGTCCCAT
TCCTGAGCTAAATTACATTTGTGTTGCTGAATCAAATTTGTCATGCTGTGTA
GTACCATTGTGAGTgttcctctcccttctcccagaTAAACAAGAAAGCGCAAGGCTGGA
GTTATTTTTCCTTAGTACTTTTCCAAAGATTGCTGAACTGTTTTGGCTGGAA
CACTCACATAAATTCAGTTACATGAAACATAGAAAAGTTCACCAGTGTTAC
ATGTACAGCTTGAAATGCGCTTGGTGATTCTACCATCTCCATGTCTCCCATA
ACAACTCAGTCCCATGATGTAGCTTTTACGGCTACCCATGATTAGCAGGTAG
GTGACAAGGCACTTGTCTTGCTTTAAAATTTGGTGTTGAGTAAACTCTGTC
ATATACGTAACCTTAATTTCTACAAATTAAGTGTGACTATTTGATGAAAGTAA
CTGATCTGTTTCTCTAGGCTTTTAGACACTTTTTTGATCTTCACTgaagaaatcaaa
ttaaaatcTCATCTAACTCTGTGTGACATTGCCTTTGTGGGAACTCGATATGAAA
AATGGTAATGTCTGCAGTAAAAGCATCTGTTTTATACCTGATGTGCATATACC
TAAGATGCATACAATATCTGGAGCACAGTGCTATAGCCTCCTGGCGGTCTCT
GAGAAACAAGCTGTCCCTCTGCCTACTCAGTAGAGTAATGCTGAAAATGTT
GCCAGGATGCAGTCGCCTACCTCAAACCATTGCTAGAGGCACTTCAGCAGC
AAGACCTATTTGTGCAGTAAAAACTGTGAGGAAAATGTCGGTGAACTTTAA
AGACAAGAAGCACTGACTCCCctaggaaaagcagaaataggatcggagaagaaaatcagaatctG
AGGGTGTCAGGAGGAAAAACAGGTGTTGTCTCAGCAATGTTGCCTCCCAC
TTTGCATCCTAGTGTGAAGGATATGTTTTCTTTGGCATTCAGGACTTCaggaatt
>TCONS_01702463

CTCCATttgtctattttattttttaaattcagttgtTTCTAGTTAGGTCACTTTAGTGGAGAGCTA
GAACAATGCGGCCTTGCATCCTCCCAGCTAGAAGAAACAATTAAAAGTAGG
CCTACGACATTCACACTGAGTATAAAACCACCCAACCATTGAATAAGGGAG
GGTTGAACCATCACAAGCTGCCTTTCTTATtgtgctggttttgttttttttctagcagtgAAGG
AGCTTAGGCAATGATCTCAGCAGAAAGGATTACAGAGATCAATTGTACCTG
AAGACAGACTCTTTCCTGCTCTCCAAGgACCGTTTGTGAGAAGCAGTGCCT
CACCAGGGAACCGTCTCACACATCCTGTATCTGGCCTAACTCCCACC
>TCONS_01741999
GTGTTGCTGAGGAGAGGTCAAGCAAGAGGTATCTTTGGCTACTTACAAGGT
TgcctttagaaaataaaagaatacatTAACCCAGTAAATGCATCCAGGAGAGGATTAAATT
GAATTTTAAGGGTAATATAGAATCTGGGGTAGTATGACTGTGTTCTTCTCATT
GGCACCAGGGCTCTTAGGATATCTGAGTCTGAGACAATATTCTTTCTTGCCC
TCCCTAGACTCCACGGAGGACCACAGAGGCGGACTTTAAAGACGGAAGCA
GGCAAATATGTCCTAGGAAGTGAGCTCACCTGAGCTGAACTGCGGTAACTG
GCCAAATGGCCGCTCCTTACCAAAGcacTCACCTGTGGCAAGATCTTTCATC
ACAATGCCTTGGCAGAGGCCTCCATAAGAAATGTCTCCCAAAAGTAGATGG
TAGTTAAAGCATTGTGAAGCAGTGAAGAGGGTGTTTATGGAAAATCAAATC
AGGGCTTTACTGGATCAAAATGTGTCTGAAGAGCCACCAAGGCATCTGAG
AATTTCTCTCCTGGTCTTTACCTACCTCCGCAGTTGATCCATCTATTAAGAAA
CAGATTTGTTCATGGCTGGactccaggcattttctgATTACAGATCATCTGAGATCCAA
GGAGAGCTGTGGAGATGAAGCAGAGAAATCTGGCAATAGTGTGTCATCAC
AGATGAAAGAACTGCTATTTGCTTTGGTGCCATACGAAATCTCAACACAGA
CTACAAAAGCGTTTCTTCTCCGGAATTCATCAAAGCTTTTGGAAGAAACAA
ATAAGTCAGAGGACACTACTGAACAAGGTGAAACGTAGTAGTCAGACCAA
TCACAGCGCAGACTGAGATGGAAGAAGTGAGATGCTGACATGCTTTTACTT
GGTATggaatggatttaaaataaGCGTTTACCTACTGTCGAGCTTGAAAgtgaagctgaagaa
gaaattagCTGAAGaagtTCCAGTGCTAGGCTGCtatgaaaacagaagactttaaaaaaaattgagaac
CTTTTGTATCAATACTGGAGTAGCACAATCCTGcTGGTGTTGGGACTGCAGG
AGGCACAGTGTGCTCGGCTCAGAAGCAGCACCAATCACATCTCCTGTCAA
CAAAGCAACTTGCTTCACGGAAACAGGGCAATGAAAACATCCAACAGACT
TCATATTTTATCCATATGTATAAATGGGGAAACAAGACAGAACCTGAGAGAC
ATATGGATGAACTGCACGCATGCATACTCACGTTATGTTTCACATGCATAAA
ACACATACTTCATATATTGACATGAAATGGCATCTGCAATCTGTATTTGATGC
TTATTGGTTGTTTtcatcttaatgattctatgattctaccatCCTGTCCTCTCTCCTCTGACTCC
ATGCTGAAGAGACTCTGATCTCCTCTCCTATGGAAATCACCTCATATTCTTG
ATGATCTTACTGCCTTTCTCCACATCTTCTGTAGTTCCAGACGACAGATTTAT
GGAGATGAGAAGAActtgaaatgaaagcaatgctCCAAATGTGGGTTTCTCACAGATT
CTATGCAGTGGTTTGTAGCACTGTTCGACCTATGTTGCTGGGTGGTCTATTT
CTCTTGTAATAATTTCTGGTGTTTATCACTGAAAGTCTTCTTCCATAACATTT
GTCCAAGCTACTCAAACTGCTCCTTCAGTAACCAGATCAGTTCTTGGCTCTT
ATTAAGACATGATTCCTATTTCTGGtgtgcttgggaaaaaaaaaagtccgTTACTGTTTTGC
TTGTTACCTTGCCTTTATATCTAATCCTACCACAATTTATGCCCCACTTCTACC
TTCCTCCTACAGATTTCACTCCTACAATTTAATGACTATGTTTTTAATTCCCG
AGAGTCTCATTTACTCCTGTTCAGCAATGCTAGCTTCTTTTTCCAAGTGTAG

AAGTAGAATGACACTCTTTTTAATTGATTTAGATTTACGTTGTTACTAAGTAT
CACACAGAAACATACCACAAGACTCCATGACACCATTAAGCATTTTAGCCT
ACAACTAATCCTTTTACTTAACCTTTCAAATTGAGAAACACTGTGGTGGATA
TTCTGCATGACATCTCTTTACTAGAAAAGAACTCTTGCATTTCCTTGCTGGT
CTTCCCATCACTGTATGCTGTGTGTGTACTTGCAGTGCCTCAGTAGCACTGT
AATACTACGTAATTAGTTTGTCCCTTCAGAGAGGATCACAAGGACAAACAC
TGAATATATGAATAAAACTATTTAAGTATCAGAGCTGCATACTCACAAAAAT
GTGCATAGAAGAGAGATACTACAACTGAAAATGATTCCTTCCTCAGCAATG
CATACTAAAAGATCTTCTGCCTGTCAGGAATCTGCTTCTCCTCAAAAGGAC
TGGTCAACATGTCCACCCACTTCTAATGCTGTCTGtttcctccttcctgctttGGACTCA
TCCAGACTTACTTGGAATAAAATCTGCCCCTTGATGAGAATTCCATGGCAGC
ACTGAACTGAGTGAAAACTGCTACAGGGATGGCAGCCCCTGAAAGCTATG
GCAATGCTGAT
>TCONS_01770385
CAGGTTGGGGATCTCGGAGCGTTTGATCGTTTATTTATCCCGCCGGCACTCC
CTGGAATTTTGGAAGACctcccagcagaaggaaggagccAGCACTGCACCACCTGTTG
TTCCATTACCTCGTGGAAGGCAGTGGCAGAACAGATCTCACAGGGAATACG
ttcaggaggaagaaagaagtgcTGTTGTCCCCAGGTCAACCTTAGCCCACCGTTCGGAT
ACGTAGAAGATACACAGGGAAGGAGGAGTTTATTCAAAAGATAAAAGCTT
AGCTAGGCTGTGGCTATCCCAAACAATATACTTTTGTAACAGGTTGTGGAAC
AAAACCAAGTTACATCAtcctgccagctgtgctgttcCCGGGGCTGGCAGCTCCTCACA
CTTCAGGCATGTccaggcacagccccagccccgctgGCCTACACCAGTGTCCCTGGGCC
AGAGCAGTGGCACTGTGAGCCCAGAGCACACAGCATCATCCTTTCCTTTGT
GATGCCACCCATCTCACCATCGTCTGTGCTGATTTCTATTAACCGCTCCT
>TCONS_01811032
TACGATCGTGCCACCAGAAGCCAAGAACAAAACATCAGCTTCCATGAATGT
GGAAGGAATAAGAACATCACAGACGgtatTTGGAGCAATTGTTCTTCCAATG
ACTGTCTCACGTGTAAAAGTAAGGGAACGCATACCTGCTTCCAGGAGGAG
ATAAAGactggttgaggttggaagagagcTCTGGATCCATctgttccaaccccttgctcaagcagggacag
cTAGAGCAGGGTGCACGGGCCCATTTCCATGTTTgtcttttgaagatctccaaagGAGAC
CCATGTTCTCTCTGGctagcctgtgccagtgctctgcctCCTGAGCAAAAGAGTGCCTCC
TGAGATGAAAAGGGAATTGGATGACAGCACTGACGTGTcttcaaaaatatgaaaattgc
AAGGAAGAAATATACTGTATCTAAAAGAGACTTTTGACAGTTGCAATATTTT
AGAATTATACAATTTTGTCaaacaagcttttttttcccctatatcCAGTTACACGGTAGATCAT
CTTCTCTTCTTGGAGCAGAATAAGGGCCAAGGGAGATTCCATAGTGATCAA
GTGCATGGCAGGTATTCTGCCAAGAATATAGAGCACACCATGTGGCTTGTG
AGCAACATACTTCTTCCCAAGTATATTCTTCAGCTGTTTAAAAGAGAGAGG
AGGTAAAAAGTAAAGTGCCTGAGAAGATCAAGCAGTAAACATGCATTCAC
TGCAAGCAAGTGTGCTGCTTGAGAGCAGGTGCTGCTTAGCCCATGCTGAA
GAACTGGCTTACATTCCAGGCAAGACAGAGATAGAACTTTAATATTTTGGA
AGTTATAAGGTCATGAAGAATAAGTAAGGTCACAAtgagttttccatttttcattgaTATCA
TGAAATCGAAGGTTCTGAATGCATTTCTCTCTTCACTTGAAGATCTGGAAAT
AGAAATGTGACAAGCTAATATCCTTCTTCAGGCACTGTGAGACAATAACAG
CCGTCAGTGAAAGGGGAAAAGCCTCCTGTTGAAAATCTTTACTATTCCATG

AATGCCGTTATCGCTATAGCAATTGCTTTACAGAAAAGTCAAATGCAGTCAA
AAGAAACGGTTTACTGAGTTAgtttaaaacatgtattttctttatatatacatGCCTCAACTTTTA
CAAACTTTTGCACAATCCTCtctgtaagaaaacaaagcttatAACTCACTACCTAAAAGA
TTTCTTCACTTCTCATACAGGCATTTCAGTCAAGGCAACTTAATTTTAAAGG
ATGTCAAAGACTATTACAGAATGTTTTCCCATAGGtgacaagaagaaaagacaagagCT
TATTTCTGtgcataaaatgtatttttgaggTGCTTTGTCTTGCTGACAAATACAGCTTGTC
AAAGAAGCTGTACTTTTCTAATTGTAAAACTGATGATCTGTAAATTAttccatattt
tctttcagcttcacTTCTTAATTATTAGAAGGCAAAGCTCAGAAAACAGTgattctttaggaa
aaaaaaaatttcacaCAAACTTGTAATGAATTATAAAAAATCCTGTTGAGAATAGTTA
TCATCTCCAGATCAGTCAGGGTCACGAGCGTTGCCAATGTACACAAGTAAC
ACAGGAGAATACAGAAGTGTTCTCATTAGCAAATCAATACAAGCAAATGGA
TGAAGCCTTCTGACACTAAAATTAATGACGGAAATCCACATCATTAACTTGG
AATGGGATTCTCTGTGGTCTATAGTCTCAAGCTAGTTTTCAAGGGTGAATCA
ATAGttaggaagaaaagctgaaggccAGTATACAAATTTCACCTGATTATAGGTACATTC
AGCGATATGACCAATAACAAATACCCTTTTCTTCACTTTGAAGTAGAGAATG
TGATGTTAATTCAGTTTTAAGGTTGAGCATTCCTCAGCCTTGCCAGCCAGTT
CTCCTAAATCCCGAAGCTAAAAACAAGGTCAACTCTTGAGATGCAGACATT
ATCTACAGGAAACGGATGTTGCAAACTTGCAAATCATCTCACTTAACATATA
AAGTATTCTTGCTCTTGACatcatttaaaagataaaatgcatCTCTCATCATCAATTTCCAG
AGCTATACAATGCTCCAGTTAAAATAGTAATTACTATAATGTTATACAATGTA
AGAGTGAGTGCTATAAAATTACTTCAGCATGAATTGACAGGGCAGTGATGT
TGCAGCTTAGTGAAAAACACCATGGTCAacagaaatctttttatttgtattgcaTAATGGTTT
TGCTGGTTCTGCTCAGcctaaaaataatcttttgctCTTAAATACTGAATAAACAGATAA
ATTCCTTTATAAAAAAACTCAGATAAAGAAATACACCTAGCTATAGCAATGG
AAAGAGCTTGTTATTGAAGAAATAAGTAATCAAATAGTTTTGCCAAATATTA
TATCTTAACTTACATTAAGTGCTTGTAGCGCAGCAtgaatattaataattaaaaatgctggAT
TTAAATAAATTGTGAGTACTTTCACAGGAACAGAGGAACTTCCTGAAGTAT
TACTGAAAGGTGCAGGAATTTGTCTTAAAAGCATTAGGAATTCTCCTAGGC
TGGTTTAGGAAGCAACTACACAGATCTTGGGAACAGTCATTTACTTCTCTCT
TCTAGAACTGGGTTTTCACATACCTGTCATGGATCCTATGCTCTCTGATGCC
CACACACGATCTCTTAGCATTGCAGTAACGCTGAGCATCTCCCTGGGATCTT
AATCCAGTTGCTGTTGCATCTAAAGCAACTCGCACCCTAAGCAAAGACAAC
ACATGGTACACACTATAGCAGCAATCAAACCTAATAAATCAGGCTGTAACT
GCAAAGTTAAACAGAAGAGGTAAGGGGCATGGTGAACAAAAGCCACTCCT
ATTTCAGATAAGAGGAATACCCTAATAAGGCCTCATAAAAACATTAGCCTCT
TTCTTCATGAGATGAGTTTAAcgctatttttaattaaaattgttaTCATTTGGAACAGAATTT
GTATTCATCCTTTATCTTGTATGTTactttgagaaacaaaaatattttgatcaaATGTTgtaacagcat
ttattttcacacaTCACCACCCATAAAAATCAGTCCACACAATCTGTTCAACTATGaa
actttcaaaaaataaaaataaaaaagcagaggggcaggaggggattaaaaaagaagagagagagagagagacagaca
gaacTGTCCTTGCATGTATAAGTAATTATCAAAGTCAGAGCAATTGTTATTGTT
GAAATCACACTCAAACTCCATTCCTATATCCTGAAGAAACTGCTTTGAAGTT
AAGATGTATTAGACCATGAGGACTCAAAGTAGGCTCTGGGAAACGAAATTG
CTAGCTCCAAGTTATTAATATGTCACTTTAATGCAATTCATTTAATGTGTGAG
ggtttttaatacaaaagtTGAAATAATCCCATCCTTCTGTCGAAAGAATAAAAACTACA

GCACTTACTgtaactgtttcttttctttttctttttacccaACTACAAAGGAAAGCT
>TCONS_01849588
CCTGGAGGAGCAGGAGCTCAGGTGCCAGCCAGTCCGGCTCTCAGCCCCGG
GTTCCCTTTTTGGAGAGGCACACAGTTCTTCCCAGGTACCTGATGGAAATA
ACCTACTGATGAAGTGTTgccagacagaagaaaagatctGAGGATTAACACTGCAGCT
GGTGGATTGGTGAAAGAGTGATATGAAAATACAGggcaaaggaagggagagaggaggc
TGTGAAGGAAACTTGAAATTTACGGACACCTAGTAAATGAGATGACATGGA
GAAGAGATGCTTGTATGAGTTGGATGACCCAAAACACATATGTCAAGAGGT
CCAAATGGAGAAAGAGATCACCAGT
>TCONS_01881907
AACTATCTAAACAACAACTGCATCATTGGATTGGAACATTCTGGCTGAAGC
TGGAGCAGGCATATGTGGTTGTCAGGAGAGACAGAGGGGGAATTGTCTCT
TTTATCCTTGTTTTgtgattgtttttccttttgtaatcaGATTTCTTTTAACCTGATTTTGAAA
CTTTAAATATCTAACATAAATTGATTTGAATTTATATTCACTAAGAATCTGAC
CTAGAGGTTTTGGACATTTATGCTAAGAAGTAAAATAGTTTATTCCCTGATTT
ACAGTGCAAAGCGTGGCTATGGGTTGTACCAATTCCCCAGGACCCTGAGCT
TCTTACTTTACCACTTACAAACACCTGGAACATTACATCAGTGGCTTACTGG
TTTCCAGACACTGGATTTTCACCCTTGGTGATCATCTTGGCAACTGTGAGA
GCTGGGTGTGACTCACCTATTCAGAAAACACGGCattaagtgaaaagaaaataatgactgT
AAAGAATTTATCTGCAGTATTATTTCCTATTATACATTTTGCtattgctttgaatttttctctt
ccaggtcTTAACTAACCTTGCGAGTCCATAATCAATGAAAAGCAGGACTGGAC
ATGCAGATTCATACAACTGTCCTGACCAAAAATCACATAATCACTCAGATGT
TTGCTGAATGAAAGAGCATACAAATGCTCTATAATCTCATAGTACCCTTTCG
CAAATTGCTCAACAAGTTGACTACCTGATGTAGGAAGAATAATTTCCTCTGC
TATTTTGTTCTGTATCCAGCTCTCACAAGCTTCATTTGAAGACCCACAGTTC
TTGTAGCAGGAAACGCAGCTCATAGTTAATTCCTCTTCACCATCTCAATGCC
AATCATGATTTTTAAGGATCATTATaacacaaaaaatattatttttcaaactgAGAAGTCACA
GCCTGCTCATTTGCTCTTTGTACGGATGTTTCCCATTTCAATTTATCctccttgctttt
cttggaATATTTGCTATTTCTAATTTAACTTCTCTCAGATAGAGGATTAAAACTG
CATAGCTTTTGAGATGTGGGCAAACTGTGGATTCAGATAGTGGTATAATTAT
CTTGTCtattctgctttccattttcctaCTAATTcctaacattttatttatgctttttgttgttctttttgacTGCTACTG
AACAATAAGCTGATGGGTTCATTGAATAATTTCACTTCTGAGCATTAACACT
GAGCTTAGAGCCCATCATTAAAGACGTGACATTAGGACTACTTTAGTGCTTA
tcaatttacatttttctgtactgaaaGCCACTGTGATCTATCTAGGCATTCAGTTTTGTAAACT
GTGCAGTTCTTCACAGACAGCTCATTTACAAATAAGCTACCAAGAACCATT
TCCCACCCAGTTGTTCTCCTGGTGAACATTGCGCGACTATCCGTGACTATAT
TTCTTATCAAATATGCGTTGTTTGTAGAGTATAGAGAGATGCATTATCTGTTC
CTACAGAGGAGGAAGTCTTCAACCTAGATCCATAACTGAAGAATTTAAGTA
TTTTGTGTCATATGTTGCCAAGACCTAAGTCATGCTGGAGTGCATCCATGGG
ACAGCCTAGACAGGCAGTGTGATACAAGTGCTGTTCTATAGATTATGGAAA
CATGGACTGTTTTCTTCTGGGATATTTGCAGGATGATTTCTGTAGTATTCAAA
TGTTCCTTCAGCAATTCAGAATAACTTAATGTTCCTTTAAAAGTACATGGAA
CATTAGAAAAGTTCTGGAAGAAGCTTGGCCTGTTCCTGTTTCACTAAGATT
CTTTCAGTTTAAGTGGATTATCATAAAGCAGTATTGACAATTGCCGAGGCAA

TTGCACAGGGTACAGCTCTCTCCATCAGCTGAGCAGAGATAATTAGATTGA
AAACCTGGCTGCATTAGTTTAATCCAATACAGTACCAAAATGTACAATCAGT
GATACATCGTATCATCTCAGCACTATGTCAAATCAATACCTGttctcagcaaaaaaaat
gtgcaagttATGCTGGAGATATGACTAATGGGAATGTTTTTTGGATCGTGTGTTTG
CACTGCAAGGAGTGATGACAGTTGGTTTCATCTGGAGTAATTACAGCTCAG
TGGCTTCTAGTTGGGTAATTACTCAGAGGAAATTCTCTGAGGGTAGGGAAG
CAGATCTCAGTTTTGTGCTGAAAACATATCTAATATAAATTCCTTAcatattgaaga
aaaaatatcctcTCCCAACAGGAAGGCAATGAAAACAGAGTAACAGAAAGACGT
ATTTATTCAGGACCCAAAGAAAGTCGTGTTTAGTaggtgattctttttttttttttttttcctgg
atctTACAGTGTCAGTTTTGGTCATCACAATGCCATGAGTTTCTGGAACAGATT
AAAGAGAGCAGGAAAGTGATTTTGACAAAGCACTCTGTCATCTGCATTACT
AATGCACCATGTCTCAGAAGGAGCAAAGTATTATGTACTTCATAAAGTATGC
ATACAGTGAATGCAATTTATGATACATACTTCATGAAACATTGTGAAAATGT
TGTATCTTGAAATTATCTTAGAATGGTTCTGACCTCCAGGAAGGCCTTTGTA
AGCAGAATGAATGTGTTAATTCAAGAATGTGCCTAGACTATGAATTCAGGA
CATTCTCCAGGAGAGCAAGGAGGTATTTTACATTTGATACCTAAGAGTGCA
GTTCCAGAAAGCAATTGAGTAGGGACAGGATGTTATGTACTGGAGCTGTAT
TTGATAAAATGTTCCCTATTCTCAAAGCATTCCTGCTATTGGAGTAAGGAGC
CAAGGAAAGGGGAATCTTGACAGGAGAAAGTCTGAGTCAAGAGGTGTTG
CCAAGGCTCTTCTATTAGATTAGAAAGACTACCTTCTGTTTCATTCTTCTGTT
ACAAGGATAACCTGAAGCCTTGAAGTCAGTCTATCAAACCTTTTGCAGAAC
TTCTTGGCAATAAAGCCTTGTTGGCACTGTAGAATTCTACTTGTAAAAATCT
GGTCTTTAAATGAATACTATCTACAAATCTTATAAAACATATATTCCTTCTTCT
TCTGTATTCCCAAGCCTGCctttaaataaattcataacTGTGGCACAGCGTGAGGacttttgc
tgttttgtctCCTTATAACCTAGATGAGTGTcagtttgcaaggaaaaaagaaagccatcTTTCAGTG
AGATCCATCAATAgctttgtaaaaatgaaatgcttgaCACAAGAGACATTAGTGTGTTTTAT
TGTGAAATTTTAGTGCCTTCTGTGTGCATAGTAGGATTCTGAAACACCAGTT
TTCTGACAGAAGTTGGAAGTTTCCAGGTGGGAATGATAGCATGGGCTTATT
GCAAATTCTAATTCAGGCTTGGTGGAGGACTCAAGGCCTTCTGAATTACTG
AATTAAACTTGTTTTTGATGAAACTTTATTTCATAACATTTTACATCTCTGTAT
TGCCATTTTCAGAACAGTTAAGTCTACCTTGAGGAATTACTGTTAGGATTAC
TCTATGTATTCAAACTTCTAGTAAATAGAGAACATTTTATCACAGATCTTTAT
TATAACTAAATAATATCTGTATGAATACAGTATCTAATGTCTGGATCAGCACA
GAGTGAGCAGATTAGGTCTGAGCCTAAATACCATAGCATATTTCATTGGCTG
AGCTACCTCTTTACAGTGATTGTAATTTTAGCAATGATGGCAATAGTAATCA
CAACATACTCCCTAAAGAGAAAGACCTTTGCAATTTCCCaagtgaatggaaaaataatt
ttgtaggATCAGTAAAGATAGCAATAATTGTTACAGGATTTATTGCAATTTATGTG
AAGAACCTCAGTTGCTCATGGTAGACTGTCAGAAGTACAATACCATGAAAG
TGGAGGCCATAATAAGCCTAAGCGTAGCATAGTTTAAAAGACCAACTTTTT
GTTGCTAATGAAAACAAGAGCTTATAAAGGGGTGAAGTGACTTCTTTAGAA
GCAACAAAGATCCCAGTGCTGCAATGCCATGTCACTTCTTCAAGACAAGAA
TGTGAACATTTTCCAAGAGTTACTCAGAGCTAAGTTGTTACTGGTAAATTG
AAAAAGCTTCCTTCTCCCCCCAGATTGAGAAGGTCACTGATATCCCAACAC
AGAGAGACagtaaaatacttcagttaGGCAGTTCTTTAGTCTATTACCTGCTTGCTTAC

TTTGAAGACAACATGAAATTGTATCGGGAGTGTTGGAAGAAACCAGCCTTC
CTAAATTCCCTTATCCGTTAGGACTGAATCCCAAGGGACTCTTCGTACCAGT
GTCCTCAAcagttcaaagtctgccctccagaagtctgGGCAGTTTTGCTGCCTCCCcctcctgactt
caccaagaattgagaactcCACCATTTTGTAGTCACTCTGTTCAAGAGAGCTCctgacctcc
acatctcccaccagtcaTATCTACATATTAATTTAATATGAGAAATCCAATTAATCTTCT
TGTCCACACACTGCATAATACAGAAAGGGCAGAAGCTTTTGACTCATGTTG
AGTGTTGAATGTGtgcaaagaaaatttaaatttaaattctttttctacttttcttgaATGCTTTTTTGAATT
ACaagacagttttcattttgtcaaCCTTAAACACATGAAAATGTTGAGGAACATCTTGGC
AAGGTGGTTATCTCTAAACCCTGCAAATGACTGAGTCTGAAAATGTTTATCT
TATATTTGaagcttttcagtattttatacTGCAGTTACTTATTTGCTTAAGATAGGGATaatgaa
aagacattttcaaTAAATGAATACTTAATTCTCTTACTCTCCTTTCACAATAACAACT
TCTACAATGTATTAAAATTAGAATAATGAAAGCTTGCAATGCTTATGTTGATG
AAATTCAGTgactttttcctgaaaatagtgttcagaaaatacaaagaatttcTGAAGGACCAGAGCTT
AACAAAAATTTGGATGCCTACTGCATACAGAACAATGTGTGGGCAACTCCA
TTCTTTGTATGCAAACCTGTATTTTGGAGTAGACTAAGATTTTTCCTTCCAGT
CATAGCTCTCTCATAttagttaattttatttatctatctatttatttatttttgcaattaaaCATCAAATACAG
GtcttgaaaaaaacatttccttttgtgaaCAAACTTGGGCGCTTTAGAGGGAAGTACTGAAT
GTTGATTCTCTTAAGGTGGTGCTTCAAATAAAAGCTACTCTCAGTTGCGTAC
ATACAGATTTCAATGAAttgatttaaaaagaagaggatCAAAATTGAAAGCAGAATTTC
AAattacaattttaattttatgacAATGGAGTTGTTGAGGTTAGAATTGACAGACGCACGC
AGTTGAAGAAAACTACGATATGCAATTAAAAAACCACTTTTTTTGTAAAAG
TTCTTTTTTGccctttatttttcagactaTTGTCTCTAAGACTAGAGCTAACTTTATATGT
CTCTAAACTAGCATTTAAATCTAAGAGCTCGCAATTCAGGAAGTCAGTGTAA
TATCACTGACAGAGCCTAGGTAGCAGGTAAGAAGGGATCCAATGCTAAATG
CTACTCAGGATGACTTTGTAATGCCTGTGTTTATATACTTTTGCTTTCAGCAT
TACAATCAGGAATGAAACCTCTTGTCTGGTTAGAAAAACACTTGTAGTGTT
TTTGCATTCAAGCCTTTCTGATGTTAAAAAGGAATCATGGTAGACCTGATAA
ATTCAAGCACTTCATGTAGACaattgtgtgtgttttaaaataacagttttatgattttctttaaagGCACT
CCCTCTGATTCCCACATTCTTGCATTTAGTTCTCTGCCCTCTCCATCTTCCAG
AGACAACTGTGGGTAGTGATTTGCTCCACAAAAAGGATCGATGAAATGAAT
GTGGCTAAAATAAATCTCCAAAATAATTCCCTGGAGGAAGGACAGTCTCTG
GAGCCAgacctgcctgcagctcaggctGAGGGTTGATTTTAGAGAGAAGTTTTAGCAG
ACATAGACCACTTTCCTTGGGTTTCTAGTGATGGAGTAAGCAACCAGCATT
GCCAGCACTGGCCTAGCCTTGCGTACTGCTACACCATTGGGCTTTGACAGG
TAGGAGAGAATTGCTCAGAAGCTGTATCTTAAAGGGATCTTTGAATTACAA
GTCTTAAAGGGAATTCTAAAAGGATCGTGGATGTGGTTCCAGGGAAAGCCT
GATTATTCTCATTAACCTCTGCTGAAATACTTACATGACTAATGTGAAAAATT
ATGATTTATAGAAAATGATTTTCGAGTGAACCATCACAAAATGAACGTGTCA
ATCAATGTAAGTACTGTGGGATGTAACCATCTATTTAAGATTTTATAAAAAA
GGGTTGTGCACCAGTGTGGTTAGCAGTGGTTTGGGCATGTTTGATAGTGTC
TGGAATTAAGAATACATTTACATTATCAAGTATAGACATGTTTCAATTTAAAC
TCATAGAGAAATGCAGTACCAACATATTGTTCCAGGTAGCAGCACACAATG
AGAGGTCAGTGAAGCTCCAACAAACAGTCAGAGGGATAGTTAACTAAGGA
ACAACCTACTGATTTTGTTTGCTACAAACATGCTCAATTGTACACGTAAAAA

TGCTTAAGGATGGTCTAACTTTCTCTCCAAGGAGCAGCATCCCTTGTAGCA
CTGTATAAAGGTTGAGCATCTGCTTGTTGCTAGATGAGGAATCCATGATAAA
GAAGCTCCTTAGTTATAGTGTACTCACACCCAGGGCAGAAAATCCTGAGAG
TGAGTCATCTATCTAATCAAACAAGCAAGGGCAGGGAAAGAACCATGAAG
CTTAAGAGCAGGAAATTCAAACTAAAATCAAGACACACATTGTTCTGGTAG
GAGTGATTGAAACAAGCTATCAGGAGAAGTGATGGATTCTCCATCTTTCAA
TGTCTTCCAGAGTGGGCATGTTCTCCGAAGATTTTGTTTTGCCAGTTGCAA
GATACTCAAAGGACAGGTTGAAGGACAGTAACTGATGAGCTGAATATACTG
CTGCACTTCTTAAGGACTCCACGGAGGACCACAGAGGCGGACTTTAAAGA
CGGAAGCAGGCAAATATGTCCTAGGAAGTGAGCTCACCTGAGCTGAACTG
CGGTAACTGGCCAAATGGCCGCTCCTTACCAAAGcacTCACCTGTGGCAAGA
TCTTTCATCACAATGCCTTGGCAGAGGCCTCCATAAGAAATGTCTCCCAAA
AGTAGATGGTAGTTAAAGCATTGTGAAGCAGTGAAGAGGGTGTTTATGGAA
AATCAAATCAGGGCTTTACTGGATCAAAATGTGTCTGAAGAGCCACCAAGG
CATCTGAGAATTTCTCTCCTGGTCTTTACCTACCTCCGCAGTTGATCCATCTA
TTAAGAAACAGATTTGTTCATGGCTGGactccaggcattttctggtACAGTGTTTATTAA
TGTCTTGCCAAgatgaaacatttctgaaacaagatTAGCATCAGTATTTACAAGCAAAGG
GTTAGCTCTAGAAATAGCTCAATCAAAAGATGTCAGAGAAGGCAACTATTC
ATGGGAAATTATTTGCAAAGGATACATTCTTCCTTCCCTAGTTATGCTTAAAG
CTTTCTTCCCAGCATCAGGTTGACTCTCTAGTTATAAGCTTAAATGAGGAAA
AGACGTATTTACCCTATAGAACACagctctttattttctgcttcctttatGCCTGCATAGCTGC
CTATTGATTTCTTAACCTGTGTATTTCTCATCTATTGACTAATGACAAAGCCT
TAGCTGTGTCAACGCTGTCAAAGCAGTCTGTGTTTATGCCACACTGAAGTG
GAGATAAGaggaattttactttttttttttctttttaattgaaataatcTCCACCTATGTCTTGCTAGGAA
GAGGCTGCTCTTGAAATCAGCTATCTGGGGACTAGAGGGATGGGAAGAATT
GTCCTGGAAGAGCAGGATGTTTGGAGATGCACAGGAGCTACACTAAGACC
ATACATACTACATTAGCCAAGGACTAGCGATGTCCTAAATATGAGCAGAATT
TTTATTCTAGCCCTCCACCCTTGCAAAGCATGTTTTTGGGGAGATTGAACtgtc
ctttattttctgttctgaggATACAATTAACATATTGGGCTTTTGGATGAGTATACAGTCA
AGTTTTGTAAATGCAATACAGTTGATTTGCAGTGCCCTGCTGTGACACCAG
TGACATCATAACCTGAGTCCATTATGTGACCAAAACAGGATTCAGGCGGAG
TGTGTGTCctctgaacaagaaaaaaagcaaacaagtaaataaccaaaacaaagcaaaaacaacaaatctCAAG
CACAAgctgaaagcaagaaaacaactacatagagaaaaagggaaaagaaaatggtggTTATTCGTTGTG
TGGTTTTAAACAAGAGGTTTGCCTAGGCTCTAATCAATATTTTAActcatttcattaa
aacttaTCACACAAATGTTGAACCctatgacaaaaataaataattaaatgaataaattgcAATTTTTT
AGAGGGCTATAATCTAATCAAGAAAATGTTCTAAATTGGACTTGCTTTTACC
AGCTGGTAATTTTTGAACATGTGAATGCAATGTAAATAAGTTTCAGCTGAAT
AGTTAATTCTTGTAGAAAATGTACATAGCCTAACTAGAGGTCatgtgattttctgttttcc
tgttgtataaagtatttggaaaaaataatgtgataTGTTGCTCCTACTAATGCTGTACCCTGTCCCT
TCATTTAATTACTGCAATTAATCTTAATCTGTCTTTGTTTGGCAACAGCTAGG
TTAGGAATGCTTATCTGAATGCTTGCAATAGAGATGGCTGTTTTGATCACTG
AACTCACCATCTTTTCTTTGTCAGATAAAAATATCCAGCATTTTATTGCAATC
TAAATGGGTGCTTTATGGTTCTGCAAAATACAATTGTGTATTACTAAGGGAG
AGGCAAAAGTATTTGCTacaaaggaatattttgtaaacagtgtaaaaatgaaattcagtgtcTAGAACA

TTTAGCGCTGTTCTCTTTGTAAAAATGTCACAGCTGTGTTTCACAATGTATG
AAGCACTCACATAAGCATTTAATTCACTTATATATCGAGCTACATAAAAGATG
ATGCAGTAATTAGGTCTGAAACAGATAGAAGGCCTACTTTAGATAATGCACT
GTCACTAAAATAGCATATGCTGGCTTCCTTCAATTGTGTATGTTTTAGCCTGT
AAAGCTGTACTTTTGATGAGATGacagttactgaaaataatagAATAGACAAGATTTAA
ACAAACTTTTCTGAAATAGATGCTCTTCAGTTTTGCCTTACTGCATCTTTATA
GGCATTGCCACATGTCCATAGAACTTCGCATAGAATTTATTGACTGTAATTCT
AGGAAAACACAATGAAGGAGTAACGTTTTCACTGAAACAAGTTTCAATTTT
TTCATGGACAGTCAGTGAGTAGAACTGTACCTCCCATAGGCTGAAGGATCC
TCCACTTCTCTGGTCTCATCAAGGCAAAGATCAGACAAGTGGGTAATCCAG
CCAGTGTGCCCCTACTCTTGATCATGTGAGAGCAAAACTGTGCATGCATGC
AGAGGACTCGGTGTTCTGTCTGGATAGTCACACCTCCTAAGATAAGACTTT
TTGAGTAGGCATTCAGCATCTTTGTAAGCAAAGAGGGTTCTTTGGCTTCCA
CtccaatgctttttctttctagagcCCAATTAAGCCAAGttctttgatgtttttgttAGGTAAGTTCACA
GTGCAAAGAACAGCTGGTC
>TCONS_01881983
GTGacaattatattttcattaacatcatagtatttttaaaataattcatcttCCACTGCATCTACATTTAAGCT
GACCTCTGGGATTGAGCCTTCAGGCTCTATTCATGGTTACTGGGACATCATG
TGTGTATGCAGAgacttatttttgctttcctcctcaggCTATTTAACATTTCCACTGCATACTT
TGTTGAGACTACAACTATGCCATTAGTCTTTAagatgctttcttctgcttatatatttcttaaaacac
aaTGAAATAACTTTACAAACTGTACAAGATAAAGCATATAGCTTGTTtgtgttccttt
ctgtttgctgtCAGTTTTTCATCAATTGCTTAGCATATAAATGAAAGCTCAATCTGA
GGATGAGGAGTCCAAATGTGCACcgttcttttctttcctctctcataCACACTTATTCATTCA
GACTCAGGAAATCCTGAATGATCTTGTCACAGCAACATTTACTACGGCTCA
ATGCACGTATAACCACATAATGGagattttgttctctgtttttgggagaatttttggaaaaaaaaatctcaga
ctTTCACAGAATGCTAGAAGATACACAGATGCTaggagaacagaaagaataGCTTCAG
GACCATTTCAATCATTTCCTGAAAGAAGACCTTATTATCAAAAGtgttacattttatta
ttgGCAACCTCCAAAATTCATGCTATTATTCTTCCTATAGTACTTGCAGTTGTC
TTCGGCTCTTCTTTGAACAGGTAAGGGACTGTAACACCTCTCTGATTCAAC
AGGGCTGTACTTTCATCACTGTGATTTCAGGATATTTGGTCTCTCACTCGAA
CAGTCAAGACCTATCATCAAAGAGCTTCATATGAGTTTTTGTGGTTTTACTG
ATAACAAATATACTCAGATCTACATCCCAAAGGAGAAAGATATGTATGTCAT
AACAAAATGACACATTCAAGAAAAGTCGGATTGCACATAAAGTGGCAGTT
ACTTTTTTAGGTATAGAAGGATGTTTAGAACTCTTAGTCTGCTTTTGATCATG
TAAGCTTTGGTTATGGAAATCTAGCATTTTCTTCAGGAACATTTCTCTGAAG
CATCAAGTAAATGGTTATTAAGTACTTGTACTCAGTGAATGCTTGCCTGCAA
TGCTGCAGAAAATGGGATTCTTCTTAATGCTCAAGTGAGACAGTCATTaatgaa
agcagcatttcacaaTGGAGATTGGTCTTTGCAGAAAATTGGTATTCAAATCATAGT
GAAACATTGACCAGACAAACAGACcaacaaaagcagctgcaacTCAGCTAATGGATT
TTGACAATATTGATATTCCTAAGTGTGTGGTCAGAAACAGGGAGATAATAGT
TAGTAGAGGCTCCGTTCAAGTGTGGTCTTTAGAATGAAGAAttgagaggagaaaag
aacaaTCTGATTCCCTATTTATGACCAGATAGTTTTGATGGTTAAAGCATGCAA
GAGTAAGCAAAGGCAGCTCATATTGTTCACTGCAGTTTAATATACCTCTCCT
TCATCCTACCAGTAGTTGAGAGAGGTGAGAGCACATAGCCATAcgaaaaaaaaga

caactcaATAACTGTGCTGCAGATTGGCTGCCCACCACTCTTACAGCTTATTATA
TCCTGTCTTTGCCAACTCTTTCCAAACAGTAAAGTAATAATTAGACTTTTATT
TGTTAGAAAGGAATTTTCATGTATGTACTTAAATGGTCTTAATGTACAGACT
GAAGTATTTTTGATCTGGATCTTCCTTTACCAAGAAAAGAGGAGTCCTTATC
TTTGTTTCTTATGCTTAAGTTGATTGACACAGCTCTCTTCTGCCAGCCTATTA
CAGGGGGTACACAAGATCCTCATGAAGGGCTGCTTCAGTCAGCAGTCAAT
GTTCTCATCTTTTTATGAAAATGTAACATATTATTTTTGAATAGTTAATTTAAT
TTGTCTGCTCTGGAGGAAGTGTCCTGTTTTGTAAAAGTACTTTGGTTTTCAT
TGCACAGGTaaaaaagctgtgaaacagcaaaCTTCGGTTTACTGCTCAgatccattttcattttagga
GTATGTGGTTTAAAACTTCTGGAAACTAAGTAGTCTTCAGATGCAATTCAGC
TCTTGCAGCTTCCTGGGAGTCAGCCAGAAGAATCTCTTGGAACTGAAATCA
TTGAGAAGCCCAGCTTCCAAGTACTGGAAATGTCCTCAGCTCAATAGTTGT
GTGAGCTACTCCAATGGGATTACAGAATTTTACTAGCCTGCCTAAAATGTGC
TTAATGAGAAGAAATAGTCACCTTAGGATCAAGTGTCCCTACATTACAGGTA
CGAATGAACACGAATGCCTCGGAGTGAGCTGGTATTACTTAAGCTAAATAG
CTATGCTGTACTGTCCCAATGTGTACAATATTTAGGACTCTGAGTAAACAAG
TTCCAGAACAAAATCAATTCAGTAAGAGAAGGTTGttgctgacagagctggaaaatgctg
tgtaagcactgctgtCTGCCATACGCTCCAGGAAACCCCACCACAGAGCTGCCAGTT
GCTACTGTAATTATAGCAGAGGCAAATGATGTCACTGCTAGAGGGCAAATG
AAAGAGCTTGATGTCATCAACGTTAGCATTATTTATGCCTAAGCATCAGAGC
TTTAAAAGTCTGTTCTTACAGAAGAAGCACCTCTAAGCATTcactttcatttgaaaattg
tAGGCGCGCTGAAAGCTCCATTGCTTTTCACTCAAATACAATTCCAAATGCA
TCATTTCTTTAACATACTTTATTTCAGTGCTACAAATTGTTTGGAgcaattgctttattt
tctaatcACAGTCAACAGATTCTGCTGAGCAAATTGTAAATGTAGCtcaaaaaatgaca
aaaagccATGAGCTTGTCTAATGCAACCTTTTACTCCACCTGGCTTCGACCAAG
GCAAAGGTTCTTTACTAAAAATGAGATTTGGGACCTAGACTACTAATGTATT
GCATCATTATTGTAACTTCATTAATCCTGAAATTACACCTGAATCTATTTGTT
ACATTCTGTGTAACTTGTGTTCACTGGATTCTTTCTAAAAGTAGACAGGCAT
TTCCAGGTATGGCTTTTTGTTAATAAAGCAGGTGTTCCTACAGTAAAATTCT
GACAGCATCTGCTGCGACTGGAATTACCTCATCTCTTAAAATCCTTTGAAAT
ATCTTATGTGCTTATGCTTGAGTAGCATCAATGACAAATATGTCTCAGACAC
TTCATATTTTACTATCTACCTGGATGTGCTTCTTTTTCTAAGAGTCAGTGCCA
GAAAAACATTCTCCAGTCTGCTTCGTGTAGAGTGCAACTGACTGACAGTGC
GATCACTTGAAAACAGTTCTTGCTGCAGTTTATCAGTCAATGTTATATCTTCA
TTTGTCAAGGATCTGACTGTTTCCCGTAAAAATAGCCAGTCTGTCCTTAAGT
GGGTGTCAGTAGCTCTGTGGTCATGGTAATAGAGCTCAGCATCTGGTAGTC
ACTGAGAATACAAATTCAGCAGCCAGGATCAGTTTTGCATTTTGAGCTGAG
CTCCGCCATACTGTGAGTCCGCTGCTAACAAACCAGCTCAGGCTTGTTTTG
GAGAGCTGCTCTAACACTTCTGATTACAATATAAGCACTTTTTGCACCGAGG
ATCAGAACAGCCAGTTAAAATCTGAAGCTTGTACATACAAAACATCTGATTT
ACAGAtagcctttttcttctttttcttttttcttttgataacTGTACCCTGAGTTGTTATACAACAAA
AGATCTTTACCATGTAGGAATCAAACCCATCGCTTGATTATTACTTTTTCCTA
AAATAGATGAAATGGAAATGTCAGGGGTGGTAGTAAAAGCTTTTTACTAAgt
gggaaatatttcttcaaaaggTGAATacctcagcaggaaaaaaaacagtgctgctTGGAAAGCCCTATAT

TCCCACAAGTCTTTGTAATTTTGCTTGATTTATGCTATTTAATATCCATACATC
TaattaaatcacattttaaatatgtCTGTTGATGTAATACTGAGGGTAAGTTTCTGTAGATCT
GTGGTAGTTGACCCAATTTTCTGTGCTCCAAATTGCAGCACACAGATGTGT
GCTAAAGTCAGTCCAGAAACAGTGCATCAACATAGACGTGATTCTATATGT
GACCTAAGCAGCTCCatctctcagctttttctaGACTTCTACACCTCAGTGAAACTCAT
CAACTTGAGCACACAATTGTTTACTGTTTCTGTCAGGATGAACTTGCTGTTG
GCACTTGTCTCTATGTCTTGAATTTTTAAAAGGCTTCTTCATGGTAGTGTAAT
GCACAGTATAGTCTGCAGGACTGCAAGATGAAAATCTTGATTATCAGTGCTT
ATAACAGAAGTTTATGATACTAGAGAGCAATTATGGTAACAGATTTCCTACC
ATTAGGAGCTGACAatctaaacaaataaaatatatttcacataAGAGATCAATATACAATAAG
CAGAATTGTCTTCTTTCTTCCAGTGCATATATTGCTAACTTAAGGGATTTTGA
GCAGGACAAGAagttgtaaataaaataatgagaaaagcaATAGCTGGAGATGCATGGCTGA
AAGAACTAAGATTATATCAGTGTCCTAAAGCAGGACAGGATATAGATCCATC
AGAGGTGTAGGGCAAAGTGTCATCCATAAATGGGAGCTCAGAAAAATAGG
AAGGGAACGTTGTCACAGAAAATAGATTTCAATGTATTTACTTATCTGTATT
AGTGAATCTGTGGATGCTGACAAAGACCCTGTTGCACTAAGCTTCTTGCTtg
atgcttgtttttttgtgacAGTCCCCCCAGATTTCTACAGATTTCTGTGAGAGCTGTAGT
AGTATAACTCCTTTAGAAACAGGAAGCAGGCTTTTATCAGCTGGAGACCAA
GCTGAAAACCCATGCATGGATTTGCatgaataaatgcaaaaaacaGTATGTCCAAGTGC
TGGTGTGAATATTTTTCACAAAGTAATTGCAGCTTCtgctcactgaaaacattttcctatgC
ATCGTCAAAATAGTTGCTTATAAGAAGTAAGAAAGATATTATAGTTCGTGAT
ATTTGAAAAAA
>TCONS_01909696
caaaattttctcttcttcagtagCTCTCTTATTCCAAAAGCGTTTGCTGTATGAAACAATGA
TGAGAAATGGAACTAGCAGCCAAAGACAAGCTGTCTACAGAGTCTGATTtta
acagattatttttattagaaaacatTGGGGAGTGATTGTGAATGACAACTGGGAGAGCTCT
TTCTCCACACTTACTCCACATCAATCTTAAATGAGTATCTCCTTCAAGGCGT
ATCTCATCTGCTCCAGTTTTAGCAGCAACTTAATAAAGTGCATTTATCACCT
CTTCTCCACCTAAAAGCTTGGCTAGTTCTGCCTTCACGTGGCTCCTAACGTA
TGTGTCAACCCCTTTTCCTAAAAATGCAGTGATTAATTTTTGTTGAAGagcattt
cctcctttctcactCCTTGAGGCTAGCCTCACTGGGCACTTGTGTTGCTACACtaaaaact
gttcatttttcctTAGTTCATCTTTTATTGAGAACTGGTAATGGTTGTAGGAGGGTATT
TGTGATCCCTCTGCCTTCATCTTCCAATGCTGTAGGGCACAGGCATGGTGAG
CAGTGTTTCTTTTGCAGCAGGGAAACGTTCCTTTCTTCCAAGCTTCCAACA
CCACTTGATGGATGTGCACAGAGACTTTGGGGCACGCTCCTCAGCTGGAG
CTACAGTTTGCCTAAAATCCCAGGTGCTACATAGATATTAATTAACTTTGATG
TTGTTTAATGGTACTCAGTGTGCATTTTAATGAGACGTACCTCATACCATGTG
TCAGGCCCAGGCCAGGCTATGCTGTCATCTGAAActccacaggaaagaaaaacacacca
TTAGATGATGGAAGAAAATTGTGTCTTGGTACTATGTAAAGATCcattatatttttcctt
ctgcttttcattcaaaatattgATGTTATTGTTACATTGAAAGGTGGTGGGAATAACTCAG
ACCACAGGGTATTTCTCAGTTATTCTGCGTTATGGGCTGGCCTTGGGGCATC
TTCTCTCCATCCCATTCCTATGAGGGCCAGCCAACCAACTTCTGAGCTCACT
CCTTTTCCTTTGCCCTGTCTCTTGGAAGGGATAGGAGAGAGTGGGGACCCA
AATTTGCTGGAGGGCGCTACTTTCAGGTGAAGGATGTCTGGCCTCCCCTCC

AGCATTACCTCCAGTGCTGCCagcccttctctctccttccctacGATGTGCACGTTTCCAC
AATCACAGCTCTCCTTActttttctgctgtcagcaACTGCCCTAGGTGCTACAGATGTG
TATTGAGGAGCCCACTCAAGCTTAAGGCGGATAAATGGTTTCAGAAATTAG
CCTTGACTGAGTCTCCAGGGTGAAAAATGGAGGCATTTCTCTCATACGTGT
TAAGACTTGCTCAATGTGTGCTCTGCATCCACTGTCCAAAGCCACTGCTGC
TCACTTGCTttgtttggactggatggcaTCACTGACACGTTGGTTTATGTATTGCAATA
ATTTCTGGAGTAACTTTATTTTAGCCTCACTTTATCAACTGCTTATTCCGCAA
TAAAAATTTCTCTTGGCCCACCAAGACAGAGCACAGCATGGGCTGCCCAG
CTGCATTAGGAAGAAGATTGAATAACCAGAAGTCCTTCTGTCTCAGCAATG
GGAAAGAGGAATGAGCAGGAGAAGTAAACTACCTTTTCATGTGGGAACAG
GGAGTTTGTAATAAGAAAGAAgtcattcagaaataaatgactgAGATGGCATTCAAAAC
ATGCACATACAGAGGTATATTCTCACACAAAGACTTGTAAGAATTATTTGAG
AGAGGCAACTGGTGCTGTCCCTTGCCCACCACTGGGAGCCatcttttgtctttgctttgtt
CTCCCCGCAATGATATATATGGGGGCAAGAAATGACTTGACAAGAATCTAAT
CTTGTAACAAATACAAAAGTAATATCATGCTGTGTTATAACTGTAGTTCAGC
TTTACTGCAGGTCCTGCGTGACAATGCTCAGCACTATACTCTCAGGCTGCCT
TTCAGGGAAGATGCCTCATCCAGGAAAGAGTTTGCAGAGAATATCCATGGT
GGTTTCCTCAATCCAATGTATGTTAAACTCTTAGGTCAAAGCTGCTGGCAAC
GTTAAGGTCTGTCTGTGGTCTTCTCATAGAGACAGTAGTGCTCATCTGCAG
AAATTGCCTCACACCTGGAGGagcagaaatatgttttataatTTAGCAAAGGTTACTGA
AGTGTTGGCTAGACGGAGGAGCAGACTGGTTAACCTCATGTCAATTTTATT
AGCGTGAAAAGAGCTTAAATTTATATCTGGGAATATAATTTATGAGttctgactttta
tttttttatttatttatttatttatttttgaagtgcaTGACCTGTATATATCTTCACTGCATTTCTGAGCT
TACTGGGCTGTCAGCATATGTGTATCCTGAGAGTTGAGAGAGAAATAATTGT
GTGTGATCTTTCATGTTTACTGTTTGAGTAAGATCCAAACTGTTCCTGTGCA
CAAGGAGCCAAAGGCAAAACATTTGTGCAGAAAAAGCTGTGTCTACATGA
TCTGGTAATGCAGTCCAGTAGGAGTAGATCTGTAGAGCACTGGAGCTCTAA
GGTGCAAATCTAGCAATATCCGTGCACCCACATTCCATGATATTCATGCATC
AAGATGCTTCCCTTTAGAAGCTTCCAGTCCAGACCTGTTGATGGCCAGTAG
AGACTGGAACAATGCAGTATGGGTGTCCCACATCTCCCACTTTAGTTTAATT
GGAAGAAAATCTGGTTTGTGTTCTTGGTAGTCATGCTGTGTTGCAAACCAA
CGTGTGTGAGAagtgaaaaggaagaggagaattGCTTTAAAAGCTCTCCCCTCATCTGA
ACAAAGTTTTGaaccaaagaaggaaaaaaactccaCCGATTAAAGAAGCACATCAAGCA
TTTCCACACCCAGTTCTCACAtccagaaaggaagaaaaaaaaaaaaaagagggaagagaaaattgA
CAAGTTTGTTCAGCACCTGTTGCAGCATCAACTGTGCATAAATGAAGTGGG
GAGCCTTCACTTCAAGAGGTATTGGGGAGTCATGCAGTTGTCAGTCAGCTT
TGAAGTATGACCAATCAGCAAATACTCTGGAGGTCTTactttattaaatgaaaaatgcaatt
tagATTTTAGgttctgtgtttaaaataaaatttccccCTTGGTTTTAAATTCCCATTCCTGTTATT
CTCCAGCTgatctccagactgaacaggtGAAGTTACATCCCTGCATCGTGAGACTGAA
GCAGCACATACTGCACTGgtggATTTACTTCTTGTCCACATCAAAATGCAGAG
ACTGCTCCATTCATCAGGTCCATGGCATACAAAGGAGATGTTATCAACATGT
ATCTTGTGTCCTAGCAAGGAAAACTGAGAATCAGCAGAAGCATTCAGGCA
AACAGGAACAGATGCCTGAAAGATCTCATATCAGCAGCAGGTGGAATGGC
TTGTAGGTAAAATACCTGTAAATGAAGCCTCTTCCCAACAGTCCCAGGTatgc

actgcagaagctgctgcgAAGGACAGAAGCATCAGCACCATTCACCTTGTGTTTCTT
GTCCTTgtattcaagaaaaaatgtgcCTATAATTAGAAAGCAGAATTAGTACTGAAACA
ATTTTCACATCACTGGTCCTTCAGCCTCCTCAGATATTATTTGTGGTATCTGC
TTTCATGCAAGAGGATGGGGCCAAATAAAAGCAATCACAAGGGTGGAAAT
GTTCTcagcaggaggaaaagggattatcaaaaagcagaggaaaaagatttcTGAACGTTTTGTAGTCC
TGGGCATCCATATGCCATTTGAGCTATGTAGAGAGTTAACAGATAgctctaaaaata
aagaatgcattatttattaACTTGTTAGAGCTTTCAGAAAATAGGAGCTGAATTCACCT
GGCTAATAAGCTACCTCAGATCACGTCCATTGAAAGATACATGCATATGAAT
TGCATGAACTGTGCGTTTGGTCTTTACGAGGCCATTCAGGAACctaaagttatttttc
ttctaggaAAAATACACGGAATAGTTcagcttttgaatattttataaagTCTGTATGCCATTCTAT
ATCTGCCCCTGCCCATGATTCTCCCTACGTAAGTGCGTGTAAttccaattttatttcaga
acaaGATTTGTTTGTAGTCCAAAGGATGCGATATCACAGAGGAGattaaagcaagaaa
acatgCTGTTGAGAACATAGCATGAAGGCTATTTTTTGGGGTCTCATGACCTTC
CAGCCCTATACGATGAGCAGAACAAACCAGTGAATGTACCAGGATGTTAAG
AATTTACAGTTTTGACAGCCTGGACCTGCTCTCTCAGAGCACATTCTCATGC
AACTTGCATTCATATTCTTGTCTCTGTCCAGCTATGCTGAATGGGGGGAGAT
TAGCAAGATGGGCTAGAGTGCTGCAATGGTGTCTTGTGCTGTGGTCAGATG
TCTTTTGCAGTCCAAGGAAGGAGCTTCATGGGTTGATGAAGAGGTCTTGTC
TGTCCCCACATTGCTCAGAAGGACATAGGCCAGGTTCAGAAGCGAGacttctta
aatattttcataatctTTGCATTAACTAGCAAAGTGAGAACTACTCTCTGTTAAAAGTC
ATTTCATCAACATAAATGGAGGTTATCTCAAAGGCAACAGGTGTTGAGGAC
TTCAGAGCCTGTGCTGCCCTTTCTGGGCTGTTAGGCTGGATAATCCTGGAA
AAGGCTCTCCTTTGGTCCACGGCAAATTCAGAGTAGGAGCTCAGGCGTTAT
GGTCTTCCATGGGTATTGAGGAAAGCTCCGCTCTCACCCTTCTCCCATGCTC
CAGCCATTCATTTCCCTGACCCTGGTGAcagccatccctgctctgcacaCCAGGGCCAA
CTCAGGACAAACCAGTTGTATGCCAGCCCTGCCAGATATCATGGAGGTggag
ggcagaggaggagaaaggatgCATCAGAATCATGGAGATCAtcttctgcaaaggaaaaatccTCAA
TGAAGAATGAAGCCCACAAACCATATAAAAGTTTATTTGATGACTAagtaggga
aggaaaaaaatatatatgttctCCACAGCTCTTGTTTTCAATACATGCATTAcgtattatttatttaca
taattaCCCATTGCCTACTGCTATAGCTTTTTATTTGCAACCTCACTTGGAAAAC
TACACCGTGTGTTGGCCTAAGCACCCAGTCTGAATGCAGAAGTGGTGTCTG
GGTGCATAGCAATAACTTCTGGTGCCACTGGTcaattttaattgcatttggCAGGCTGA
AGTTTGAGTAAGTTCCTTCCAGTTATTTTGAGCAAACGGTGGGTAGGTACA
AAAAATATCAGTAGATGTTCCAGGCTGAAAGCTAAACTCAGCCTTAGAGAA
AAGAGGGCTTTACCTCTTAGTATAGGCTTCATGTTTTTTGCGATGCATAGAA
TTACTCACTTTTTGTATGATTTGCTTTCATTCAGTTTTCTCCATATGTCCAGA
GACTGTAGGATGTGATGCAATTGAGAGAACAACCATTAcccttgttttaaaaagaaatcct
tcaaaaaaaaatgGGTTTTGTTTCCAACTCCcgggttttatttttcacattcaaagaaaaaactTATTTCT
TGTTCTACCTATAACATTATATTCCTTCTCAGTTTTGAAAAGGTCACATCACC
TGATGTAATAATTGCATGCAGCTGTTATGTAATTGTTCCTCATATTTCTATAAA
AGGCAGTAATTGGAAGAGTTTCTCGAATGTTATTATGCAGCAGGTGCCTCCT
GGGGACAACAACAATAGCTATTCATCTGGAGGCAACAGTCCCCATagggagctc
agggctgcccGTGGGAGACACCATGGACCTGGGAAGTTATGTTCTCCATCTCCTT
GCCGTGCAGACAACAGCAGAGCGTGGGTAGGTGAACTTTGCCAGCTCCAT

CTGTCCCACAGGCTGGGTGAGTCAGCTTGGGAAGCAGCATGCATGTGCAC
ACAATGTCCCTCTTGCCAGAACCAGGAGCAAGGGTTCTGCTAGCAAGAGC
CTGCAGCCAGCATCACTCAGCTCTCCTTACATGTCACCCTGGGACCCTGGG
CAGGTGAAGCAGGAAAGATGTGGGGCTCTCCATCCCTGCTTTCTGGAAGAT
TCACATTGGGAATAACTAGCAATACGTGATCTTCCTTTGAGTGTCCTGCCAC
CAACTGCAAAACCCACTCTTCTCCAAGCCCACAGTACTGTAGTTCAGTCTT
CTCCCTCCAGATTTGCTTAGAAGGAATGATGAAGTAACATCCACTATTTACA
TATTACATTTAGATTAGATTTAGAtttcaaggccagactggacagggctttgagtgacatggtctagag
ggaggtgtccctgcctatagcagaggggttggaactagatgatcttaaaggtcccttccatcccaaaccattccatgattcta
caattAGAAAACCGACTTCAGAGACTCATACTTTAACAGTCAGTGTTCATGAG
TGTCTGTTCAGATGGGTTGTAGAGCAGCGTGGCATTTTTCTCCCAGATGACT
GAATTGGGTTGCACTGACTTCCATCACCCAAAGGCTGGCATGAACAAGATG
ACTGATTTATACAGGAGCTCCTGAGAGAGTAAATACCAAAATAAGTCATCT
GAGGAGCTCCCATGCACCAGAGAGCAACCTAATGCTAACTGTGACACCAG
AGTAAATTGCAGCAGTCAGCTGTTACATTCCATCTAGCCGTTCCGCTCGGTT
TAATTAACCTTTCGGGAATTAGCGTTTTGTGGACTTACGGTACCTGCTCTGA
ATTTCTGGCAGT
>TCONS_02059755
CATCTGCCGGATCGTGGCCGCCAGGTGCTTTGTGCTGTAGCACAGAGACTC
GACGTTCTGCTTTTCGCTATCGAAAGTCTCTTGAATGAATTCCAGCATGAAA
CTGGACGCCCTTGGTTCAGCCGCTGCACACGTCCACGTTATACGCCGTCAC
ACGCGCACCCCTCTGTGAACCACACCTGGGCACATCCCATCGCTCCGGTGT
TTCCAGCACACAGTGGTGGAGGAAGAGCTGTGGTACGAATCACAAGAGTaa
agcacacagccagcagcgTGTTTGGCCAACACCAAATCAGCGAGTCCAGCTGCAAG
GTGCCAAAGGGCAGGACCAGCCCAGTGAGATGCCATCTGCTTGGAATCCT
TTGCTTGCCATTGACATGGGACACTTCATTGCCAGGGGACAAAGTCACTTC
ACCTGTTCTGCCAGTTCTTCCTCTGAACTAGCCCTGTAAACACTAATAATCT
TGACACAGCCTGGAGCAACAAGAGGAGTGTAATCACAAAAGGTCACATAC
TACAATCTGAGCATGAAGTTTTATAGTTCTGAAATGAAGCCTCAGCTTTTTT
TTGCGTGACAGGCTTTGTAATAAATTTCTCTGGGGATTAAACAGAAAGGAT
ACATTTTTTCcagtatattatttttaaaaagtaagttTTTGAAGCACTTATTTGTCCACTAAGG
ACTGAATCATCCTGGATAGCTCTTTTCTTTGTAGAGATTCCTAATCCTATGGT
ATAATGTCTTTTGATAATCCTGGTTTGTTGTTAATTCCCTACTGATGGGTATA
CTTTTTGCATCCTCAGAACTCCCCTGTGCTTCAGTTCTTTTTATGCAACCTG
CGTCTCTTTCCCCCCAAATGATTTTTTGCTTGGAATATTTCTCTGTGCCCCTG
CGGTAGGCATTCTTTCAGACAGCAATTCACTGTGAACTATTTCAGGTAACAT
TAACCTTCAACTGGACAGAAAAGGCCATGGGAGAACATTTGATCAGCATCA
GAGaatatacatgttttttttccctgggaatAAATTGAAAAGTCCTTTGGAGCCGCCTATGTG
GGTAATTATTCTATCTTTGCTcttacatattttctgtttctcttttgcttcttgCTTTACTTCTTGACT
GTCATTCTcaagtgaggaagaaaaaggtcaCACAAAAAGCATACTTATGCTACACTGCA
GTAAATAAAGGCTACTGTGCAGCCgtgcagaagaaatgcatttttgcatcacccttattttcttccctta
atgATCTGGGATGGTTTAGAAAATCAGGAGTGATTtacatgaaacagaaaatatgatgTTTT
ATCTGTAAATGTTCTGAAGAACGGGGATGGTTCCTGATGTTTCTAAATTGCA
GGCTGCCCAGCTGCCAGCATTCTTAGCTTGCTGAGTGTATCAGGTGAAAGG

TAACAAACAAATCCGGACAGGCAAACATCTGTCCTGACATCCCTTTCTCTG
AAGCTTGGTAGCAACACTGTGTTACACCTCAGGAAGAGGTGCCCTGGGGC
CAGAGACCGCCGGCATCAAGGCCCAGTCATCACCAAGCGCATCCACAAAG
TAGGTAATTAACAAGCCACAATGACCGCCTTTCTGAACCACAGCTTTGTCA
TCTTGAATACAAGTGATACTTGTACTGCACTGAATACTCAGTGACATCACTC
TCCTCTGCCCGATGCGGAAGATGAATTTCTTCAGCTGTGCTATCTGTATCTAT
AGCATAGTCACTACATATACTGTACAGTATACTACCACCTATACTGTATTTCA
GCTTTACTTACAGTGATAGGAGCTTTCCCTGGGAAGTGAGGGGGGAGCCA
GAGCTGGTTGGCCATCCTGGGGCACTCCCGCTGCTCTGGGGTTCCATCTCC
ATCAGGATGATCCACAGCAAAGCTGCCAAAAACTCAGTACTCAGACTCAG
AGCAAGGAGAAATCTATTTGCTCCTGTCCATGCTGTTACTGTAATCGCACTC
CGCTTTGTAGCAGAGAAACTGTACAGGGAGGCATCTCTAATGGAAATTTTA
GTCCTGGCTCTAAAAGAAGCCATGTAATTGCCTCAGGCACTTCACATGACC
TGGAATTGTTCTTAAATATTCACCAGAGGGCCACATTCTGAATGCTAACTTG
TCacatgcatttctgtttctccagcttctttcacagtgttttcttaGACGTGGAAGATCTGCAGATAGGT
GCTTGACCCATGTTTCTGGGGAGAGCTGTTACACAGGTGATATTACTATGCC
CACACAGCAGTGAAGCATATGAGGATGTCCACTGAACCTGGTTCTGCTACA
GTAagggtttggggctttttaGGGTATATTGTCTAAAACCTTCAGCTTCAATGTACATT
TCTCAGCATACGCTGAACTGGCAGATGTAGATACCCTTTCTTAGGCAGAATT
TTATGCAACATGGCAGGAATACGTTACATGTTTCCTGGAAACACATGAAATC
TTTCCAAGAGTCTGTGCAAGAAAGCACCAGGCAAAAAACTCTGACATAAG
ACAACATGCTCAGGCATAGGAAACAGTTGGTTTTACGTAAATAAACTAACC
TTATCCTATTACAAATAACCATTCACGTTCTCACggtttctcatttctctctgctaATTTCTA
ACTTTCCAGTCTTTTGCATTGTATTAATTCCAGTTGGGTTACAGTGCTGAGA
AATCTCTTTTTTCTCTATCAGTTCATACCGTGTAGGTACCGTTTCTCTTAAGT
AAATAGCAGCATCCACACCATTTCAATTTTAAGCTTGGTGGTCTTTGATTTG
CAACAGAAGGCTATGATTATTGTTGTTGGCAGTACTTTtcttatgtctttttttttttggtaaa
gatTTTGTAAAGGATAGAGCAGATGAAAGAGTTTGGGTCTGATTTAAATTGCT
AGAACGTATTATCACAATTTCGACTCAGCGTGCTGGAGAATATTTGCAGTAT
GCTTTGAATGGAGTTTCTGGGATTTTATTCACAATTGTGTTTGAACTTACCG
AGTAAAACTGGCTCGATACGGGGTATCAAATTAGGATGTTTTAGAATCTATT
CCTATGCATAGATTTCTCAAAGACTATAGGAGATTATTTCATACCAGCAACTC
CTGTACCAAGGGATTTTTGCCAGTGTTCAAGAAGATTGGAAAGAGGCCAA
CAAAATCCATCCTGTTGATTTTCCCCACATTGCGAAACTGCTATAGCTACGG
GTACTCTAATAGCAGTGCTGGTAACCGTTTTGCTGGGAATGCTGATTGAAA
AGGATAAAGAAGCTGGTAGAAACAGGAATCTCTTAACCACGAGGTTTGGA
AGGGAACTGAAATCTTGCCTATTCGTAGCCAAATCCTCATCCATGATTTCAC
CTTTCCTCCTCACTGTTTTCCCAGCCAGCAATCCCAGAGCTATTCCCAACAG
CAAGAGACAAACTTAGAGTGATGGTGGAGTTGGTTGGTTACCCTGGGCCA
GGCTGAGAGCTGTAGGAAGGGTGTGTGGGCTTGTGCCAGCTTCTGGCAGC
GCTGCTATGCGATGGAGCAGAAAATGCAGCCTTTGGCACACAGAGAAAGA
CATTCATTTACTGACAACCCCACAGCCACCTGCTTCCAGAGAGTCTTTGCA
CTGATAGCATCCAGAcagctttcttctcagtttcttaTCCACCTTGCaaactgctgtttgcagagggGA
TGCCAAAGGAAGTTAGCTGTGAATATGCTGCTGGCTGTTGCTCAGATAGGC

TCTTTTAGCACATTTTTAGGATGATCATATATGCAGGTCATTTGGGATCACAG
GTGTGCCATCATTTTCTGACCCTTCTGTTTAGAGTTCAAAATACAGTGAaactg
acttcttttttttctagagaacTCCTGGTTATTATGGGGCAAAGTATGCTTATGAGTTCTGT
TGGGATTGCTACATGGAAGCACAGCCCTCGGACCAGggattcttcattttcttcctgagtG
AGTGTGAACAGAGCTGGCTAAAAGGCAGAGAGCACTGTGGAGCATTAATG
AGTATGTGGAGAGGAAGGAATTAATGAGCTAGGTGCTGCACTGGCAGCAA
AGGTGCTGGGTGGTAACAACGGTGGAAGAGAAGGATCCTTGTGTATACAG
TGAACATGCTGCTGATGTCTTAAAGTAGTCATATGAGGTATATccagcagaagagaa
ggaagcatACTGCCTTTATTCCAAGACGTTTGTgaaattccatttaaatatTGCTCATACTTA
AGCAAAGATGCACTGAGACTGCAGGTGCCCAGTGATGGCTGGAGTGGTCA
GAGGGATGGGGAGCTttgtgctggagaggagctggggAGTGGATGCTGCATTTAGGCT
GGCAACATAAAATAGCATAGGGGACAGAGGTGAATGCATGGGAATAAAATA
GTTGTGAATAGAAACGTGATTTTCAAGTGAGGAAGTGCTTCCTaatcatgggaaaa
aaatcgTAACCTGAAAAAAGTTTTGTAGTgaggtggtcaggcactggaacaggtgcCCAGGT
GGGCTATCAAATCGCCAGCCCTGGATGGCTGGgtccagctctgggctggctGGGCATG
CAGGGGTTGGCCTGGGTGACCTCCAGAGGACTAGGCTGTTTTGTCCAGGTT
TTGTGCCATACAGGGAAGACAGATGGGAACAAAGCAGAACATGTCTTGCC
ATCTTTTTTAGTATTTGGTTCACAGCTTAGCAGCAAAAACGATGCCTTCCCT
GTGTTTTTTGAAACAGAGAAACCTAGCAAATCACTTACTGATTGTCCTTCTC
TTTATCACTCATTTCCTCGATTTAGTTTTCACCAGATTCAGGGTTTTTTTGCA
GGAGAGCTGAGAACGACCACATTAATGAACAAAAAGAAGTGCTGATGGTT
GggaaaaactttatttttcagtgaagagTACAGATGAGGTAGAATGGAGATAAAGAAAAtagc
tggggagggaggaaaaaattgAAAGAGGATTcgagagaaggcagagaaaaaaagaggtcaGGGAAAG
TACAGTTTGCTAGAAACTTTCACAAATTCCAATTTCTTTGGCAGTTCCGGTG
GCACACTCTCttaatcatttaaaaagcaaaatgaaatgtggaaaCAAAGCTGACTCTGCTTTCA
CTTTTGAATCATGGAATTACAGAACTGACAAAAGAGAGGGCACTTGTGTGG
GCAGAGGCAGCAAGAGCAATATGAGTCAGCCCTCTTGCCCCAACTCCTGTA
ACCCACTGAGCTGCACTTCAGCTGGAACAGGCAGTGGATCAGGCCCATCC
ATCCCCGTAACTGGCAGGAAAGATCCCTTGTTTGTAACTCAGGATATcttcttcatt
gcttttgtttgtatCTGAAACTAATTTTCATATACTGGCA
>TCONS_02128172
GTCTGTTAACTCCCACCTGGGAAGCGGTTTTGGgtgccctacagaaaaacagaagtctgG
CATTGCTTCCTGTGAGAGCCGAAAGCCTGGAACTGGAGCCAAAGATTTTC
AGACAGATTAAAGGAGATTCCCTTGAGTAACGTGCCACACATCACGGCACA
TTGAACAGATCAGTGGATGCCCTTGATACcacaaatgaagaaacattCTCTTTGCTGT
ACATGCTCAGGTACTTTCAGAATTGGGTTCAGCCCAATGCATCTTcgaatgtattttt
ttccattgtgaaCTGCACTTCTTGTGTCCATATTGTGTACAAGGTGGGTCATGCAAG
AGTAGCAGCAATGAACCTCTGGCACTGCTGAGGCTGACTCAACCAGCACT
TGTGAGttatattgctttttaaaataaatttataatcAGATAACAAATCCAGTCACTAGCACTTC
TTCCAACTTCAGATGCTGGTTCTTTGATCGATATTTTTGTAGTGCTGACTTGT
TTTGCTTAGTTTGGTTTAAAATAAGcctttttctgaaatgaatgagTGAATATGAAATAG
AATGTAATATGTATTTGGCCTCTTTGGCCAAAGTCATCtatcatttttgttgcttctggCCA
GCACAAAAAAAGAGCTAAGCAGGGACATAATTATaagcaaaaagcaaactgaGCAGC
CGCAAGGTTGGCTAGTAGCTAGCTAGCTGTATGGCAACAAGAATGAACACT

TGCAAAGGTAATGCTTTTATGAAGTCCAACcagtgttgtgggtttttgttttgttttgttttagttttgct
tCCTAATAAAATCTAACCAGCTAAAGAAATCTTAGCTATGTGACTATAAAAA
ACTACTCCAAATGAGAGAATGTTTCCCCCACTTCTAAAATCAAGTATGGGT
GGGGATTGCTATTTTtagatttaattttaaaaataattatggcttctgttttaaaaatgtcttaCATTGGTGTC
ATGCTCTAATCTTCAAGGATTGTCCATCCTGCACTACTATAGTGCAAAGGCC
AAGTATATGAAGTAAGAATATGCTATAATCTCTCCAGGTTTGAATCAACACTT
AGAAAGACACAGTATTGCCAAAATCTAACAAAAGTGTGTTTAGGCCAGTTG
CCTTACACTGGCTATTGCTAGGATACCAGTCTTTTGGATTCCTGATACCCAA
AAGGATTATTCACTCTTGGCTTCTCTACATAATGTACAAATAAAAGGGAACT
ATAAATCATACCTTGGAAAACTCAGTCAGGCTTTAATGCCTTAAACTTGCCA
AGTTTGTCTTACTAAGAAGCAGCTGTTTCATACACTGAAGTAAGTAATAAA
AAGTAAGTTTTTGAACATAAAGGTACAGcatacagaagaaagaggagggatTGACAAG
AGAGGTGATTCCCATACATGTAGATGTAGAGGGGCAGTTAATTCCCTGGTGT
ATGAcaagaatatttattattttcttcaacaGGCTGCATTTAAGATGTGTTAGACCATCCTGAT
AAGGAGAAGCAGATTAGGTTATCTAATCCATACCCGAGTTATGGAGTACTGC
AGTGCATGTAACAACCACTGCATGCATTATCATCAGCTTGTACTGTGTCAGG
GCACCAGAGTGTactgctttgcatttgttttgaagcAAAATATACTTTTAGACGCTCACAGA
AGAATCAGTCGTGAGTGCAGAtataacaaatatattttgtgcctgacttttctttccccagacaATGGC
TTCTGCTCTGGTTCCAGACTGAGTGTGGCCACCTCTTTCCACAAGGTCCTT
CTCAGTGCAGTCAACAGCAGTGACACAAGTGTGTTGTTAATGTGCTGGTGG
GTCAGTTAGCAGCTGGGGGGCTGTAACCTGGCTAGGCCACAGAGGTGGGC
ACACAGTTCTCCCCCAATCTGAGAACTGACCAAACCTCACAGCTTGGCATG
TGGTTCCATGTGCAAGAGTGCTTCAGAAGCACTCCTCTGCTTCAGAAGGGC
ACTTCTGGGAGAGGAGGATTCCCCCAAGGACAAACCTTGGCGGCCACTTG
AGAATGTAATACTCTCTACTGAAAGTACAAGTGGAtcacatttttcctgaaaggatGAA
GGGATCTTCTCTCTTCTGGGGAAGGTAGATGGCAGAAGCAGTGTTCTCTGA
TGTTATCACAGCCTGTTATGTCAGCTGTGGAGGTGCCCAGACTGCTGGATC
AATAAGTACCTGGAAAATATATAGCTGTTTCAGAGACAGAGACTTGCAAAT
AAACTGTTTCTACTGTTTTAAAGTCAAACTGTCTTTgaatctgctgttttctttctgcagttgtt
TGCTTCCAAGGATACTGCTTACAAGACATGTCAACTGGAGGGAATTACATT
AGCAAAATGGGAAATCCATACAGTTAGCTGTGCTGTGGTtgactgatttttattcttGG
CTTACAGTGTAGTGATATTCAAACTGTCAGTCTCATCTGAGTTGGATTTAAA
TGAATGTGAGTCCAAACTGGCATGTGAAATTCAATTAAATCACCACATCAA
CTTCTGTATTTGCGTTCTGAGGATTGAAACTTAAAATAAGTGTTAATGCTAT
GAATTATACACAGTGACGGAATTTCTGGGGTCAGTTTCCAGGTGAAAGGTA
ATCAACGAAGATACAAGAGTAATGAAgtcttgttttgaaatgcagtgCAGGTGCAGTAtcc
agcagaaaaataagttaatCAATGAACTAATTTGATAGTTCAAATTCTGAGTACAGGCT
ACTCACTGGGCATATATAATGCAGTTTGAAAGACAGTGTAAATTATTAATAA
CATAACTAGATTGAATGTCTCTATCCATTCATTCATCCTGTAAGCGGGGGCCT
TACTCCTAAATATTTAACAATAGTTATAACAACTAacctattttcttcctttccagcatGTAG
GAGGAATTACTTCATTCACTTGTGACAAATTTATTTGCATATATGAATGTGTT
CAAGTGAATCTAAGCTGGAGAAATACACATCAGTCAGTGTTGCAGATGAAT
TGGTTGCTGCTTGgtggcatcccacagctcttAGAGATAACACAAGGCAAGACCAGATT
CTGCGTAGGCTCTGTCTgcttcatgttttctgtttgttgatGAGCTGTTTATTGTATAGATGT

TGTGGTAGATCATGGCCATGATGTTGTTCATACAGGTCTGGCACAAAATATC
ATTATGGAACCTCTGATTCCATTACAGCCGTGGAGAGCAGAACGAAGTCAA
GGTAAGTGGAAGAAGTTGCCCCAGTTATATGGCTTCATATAATAAGTGCAGG
TAAAactgctttcctcttcttttggCATGACAAGTCCTGCTTGTGACTGTCAGTTCTTGGG
TCAGGGTTGTCTCTTTAAGTTCATTAAAGAACTTAATCATCATGCATCCACA
GAAGCctataaaacattttgttagCCTAAGACTGTCAGAGTTATTAAGATATCTTCTTTT
GCCAAGATAGGAGTCAGGGGCAGCAAGCCAAGACTCTTGAGCCCTCTGAA
GTTCATGGCAAAGAAGGGTGGCACTGCAGCAGTACACCTTAAGAGTTTAC
AGATGAATAAACTGAGTTGTTGTCTGACTGGTAGGCCAAAAATCCATACCT
TGCAGTGATTAAGAAGTTCGTGAAATGAAGAGAGAATGTGgtccccttttcttctgtct
tatattttttctttgctctgttcttCTGAACAACCAGTGCTACCTACTTGAGTAGTTTTACTT
GGAAAACAATTCTGTGTCCTTactgttaggaagaaaaatagtgtCTCTAATTGATTGTCTT
TGcttatctttcttctttttccttgaaaCATTGGGATTGTCATTCACCTGCAGTCCTCCAAACC
TGAATTTGATGCTCTCCAtctcttctttcctcagttttcttgtatttcattCTTGGTAAACTATTCCC
AGAATAAATTTTTCTGACTGCTTTGATATTTTGGAGTCTAACATGGCAGAAA
TATATTTGTTGAAGGTTAGAAAAAATCAAGTGAACAAGTCAAAGTTCCTGC
TAAGACAAGGAGAATGACAGTTGAAAAGTGATCCTGGTGGTTCAATCTACA
TGGGATAAAACACTGAATCTTTGGGCTATGTCTAAATACAACTGACCCTTCT
TTCATAATCACTAACTTTTACATAGTAATCTATTTTTCCAAACTCACACTACA
TGATCTGGACCTTTCAAATTTACGGTCTTCAGGAAAATGTAAGTTCAGTTTT
AACAAGCTTTTCAGCTTCTTACATCATCATATGAGAAATTGTATTTGTCATAA
TATACCGGGAACCTGTAAAGATAAATTGGTTGTATTTCATCACAATATGATTA
GGATAGATTAATTGTGCTGGCTTTTGTCGGATAAACACTGTACAAAGGTCTA
CTTTGGCTCTTGCAATGTTTTTAGCCAGACAGTTTTGATATTCCTTTCTTTCC
GtgaataaaatcatttgtgcctcTTTAGGTGCACAGGTCATTTTGCACATTGGGTGTAGCT
CCTAAAATGAGCCAGAGAGGAGGGGACATAGGCATGGGGGAACAGCTTCA
GGAACTGTTAGAGAACCACTCCCAGCTtgttaaaaaattatttccatgtgAGCTAATGCTA
TTCCTAAAGCTCTTAGTGGAAACAGAGTTCAATATTATCTAGCTACATCTGG
AAAGGCTCTTTTTGTCAGAATGTAATTTTTATAACATGGCTATTCTAGAAAGT
AAGTGAAAGCTTTGGTTAGTTTTCCCTCTAGTTCCCTGAATGCTCCTATAGC
TTTCAtagagatatatatttttttt
>TCONS_02217494
CTCTCTGTGAGCTGCTCTACAAACCAGGTACTGCTCATACAGTGCTAACAG
GCAGCTGGCTGCTCCTGGAATGAGGTGAAAGCCGTGTTCTCCTATGAGTAG
AAACTCAGGCAGCTCAGGAACAGCTCCTGGTGAGGCTGGTGAAGGGGGA
AGGCAAGGGCATGTACTCTTTGCTAATGGGAAGAAGGCCTAAAGCATCTCC
CCAGGCTTCAGTGTGGGAAACACAAAATAATTGATGAATAGGTGGGTTTAC
TTCCTTGCCTGAAGTTGTTGGTCAGTCTTTTCATTAGGCAGTTTGTTTGACT
GCATCCATCCTTTCTGTTAATGTCTCACACAGCAGATCCAACACCTGCCTGC
TCCAGAGATGGGACTTGGAGGAGTTGCTGCCTGTGGAGGCTGAGCCCCAG
CAGTGGTACTGGTGCTCCTCGTTGCCGTGTGGGATCTGCTGGCTGGGATGA
GGAATGGCCAGGGCCTGGATGTCCTCGTGTTGGGAAGAGCGGAACGCGGA
GCTCAGTAAGTCCCACTTGGCGGCAGAGACTTGTGAGTGATCAGCAGCCC
TCGGGGGAGAAGTGTGATCCTGTTTACCTACAGCCATAACTTCATCACTGA

AGACAATTTTAAGCTTCCTAGGAAAGTTAATTAAAAGGTCTGAAGATGTCC
CTATACATGATTTCTGAGCTTATGCAGCGCGTGAAGGTGAAGTCACATTGCA
ATAAGAGCATCAGGAGGCACTACCGAAGCATTCTGACAGGATGGCAGCATC
CTTCCCTACATGTTCCAGGACATGCACAAATGCTAATGACTGAAggtatttgaaata
tatttagatcAGACTCATGTGGTTCACTTCACTTCTGTGCGAAGATCAATGTTCCA
ACCTTCAGGAACTGAGTTTCAACTGGGAACACTAGGTCTGGCCCTTACCTT
TCTTGGTCCCATGGATCCAATATGTTTTCATTGAGCTTGTGTTTTGGAAGCG
TAATATGCTGCACCTGTAACATGCTTGTTTTCACATGCAGAGATTCCTTTCAT
TGGCTTATAGCTGCTGTTCTCAGGTTTTTTATTCCAGCTTACCTGAAGCCTT
GTAGGAATAATCTCCTGTAGGCTGTAATACCAGAATTGCAAGGACATCGTTT
TCTGAGGCTGAGAATTTAACCTTCACAGCTTCAATAGATGTTGAGTGCTAG
CAAATTTTTTTGCTAGTGGCAATCTGAAGTTTAGACCTATTTTTAATcacaattttg
aaaaatatagtTTCAGAAATAACTCCTAGGAGTCAACTGCACTCAAACATAGATTT
GTATGCTTTGTGCAGGTATAATCACATGGTCTGAGTGTGACAAATCTGTATC
AGCAGTTTGTAGCAAGAAGTATGCCATATTCATACCTAGGTTCATATATTTGC
ATTAGAAATTAATACAAAGGAATTCTAAGCAGTTGTAGTTGAGTTGAGGCA
ATTATGTTGTGATTTGCTAAATGCTCAACAAAGTAGGAAAAGAATAGGACT
TTCTTAATGTTGTACTTATTCTTTGAGAGAAGTGTTACAttcctcttctgtctttctaGTC
CACGTTCATGGTAATGGATACTTCTGAAGAAGTACtgataaattaaatataattttaagttTC
TCCAGAGCCTCCTGGGAATCCTGAAGTAGTTTGCTTTTAGTAAAGAATTAA
AGAGCAATGCTGCTGTTATGAAAATGGCCGTTGTCGTAACAatgataatttttttaaaag
gtagGAGaactaaatcacagaatggctaaGCTCGGGGTTTCAGAGCACTTTAAGGACAT
TTTTGTGGTTGCTTGCTTTGGGATTGCTTAATGTAGTCATCTAAAATTCAAG
GCTCCTAAAATCACCCCATGGAATCACTGCATCACTATGTGGATTTTGCCCA
AGATCACAGTGGG
>TCONS_02245846
ATGGCCAGGGCCTGGATGTCCTCGTGTTGGGAAGAGCGGAACGCGGAGCT
CAGTAAGTCCCACTTGGCGGCAGAGACTTGTGAGTGATCAGCAGCCCTCG
GGGGAGAAGTGTGATCCTGTTTACCTACAGCCATAACTTCATCACTGAAGA
CAATTTTAAGCTTCCTAGGAAAGTTAATTAAAAGaAATCTGGAAACCCCAGT
TTTCACTTTGTCTGGTCACCCTGGCCCTCCTTCAATGACTGCAGACAGGAC
AGCAGCTGTGACCAAGGATAGGCAGGAAGGAAGCAACCTCTGGTGTCGGA
TGGGGACACAACCAGAGCCTGGACCTTCTGCTGCCATGCTGTGGATTGAG
CGTTTCATTCACACAAATGCACAtttcctggcagtgctggggctggcCTGGAATCCAGAC
CTGGTCTGCTCAAGAGCAAGGTCGATTTGTGGACTGCTCTCCTGGAAGATG
TTATTAGGACAAGGCATGGAGTTGTATGTGTAAAACAGATCAAGATGATTTT
CTCAACATTGCACCCAAGCAAGTGCTGAGAAACAGGTTGGTGCTTGCCTG
CTCAAAAGGTTGGGAAATCTTGCTCAGCTGGGACTAGACTGCATGCTTTCA
CAAGAAGGATGATGAGAAGCTGCAAAACTCTGGGCTGAGGACTCCCCCTT
CCAAAGCTGCCCCTCACACATCAGACACAAGGAGTGTGTGACACACTGCC
AGCAGAACCACTACCTCAGCAGCTTCCGCTGACCAGCTCCAAGCAACGTT
TCCTGTGTTGGTGCccaggagggctgtggggctgcagtggtCTCAGGTCCCTGAGACAAT
CCAGAGAGAAATCTTCTGCATCTTCCCCATGGCATTGAAGGCAAAAATTCA
TTTATTGCTCAAGGTGTTGCCTGTCAGAGCATGTCTGAGTCACCACTGAGG

ATGCAGAACCATCTGatgggaaatgagaaagaagatcCTAATTTTAAATATCCATTGCCC
CTGCTTCCTGTGCAGCAGTAAGTGAGGAATGTGTGTTCTCCTCTGTTTGTTG
CTGGCTTGTGTTTCACTGTATCTGGTATTTAGAGTTTCTTCATTGTGATGGCT
CATGGAGACTTGTCATAAGCTGTTCCTTGTCTCTCTCCAAGCATGAAAATCA
AGTTTTGGGGGTTGTGCTTAGAATCAGATCGTATCAGTAGCTGTTGGCAGG
AATTCACAGAGATGCCAAAGAGAGCTTTACTTTTTCCCCAGTTCCCTACCA
CTCTTTCCCATTGGTACCAGCTGGTACAGTGCATTTGTCTTGGATTTTTTCA
GTTTACTCAATACCTCCAACATCCCTTGCTGTTTTTAGGAGATGAGGAGGCT
GACCAGCACTTAATAAGTGCTTACCATCTTAAACATGGTCTTAATGTTATACC
TGGCTTATATGTGTAAGAGATAGTTGTGATTCTTATGCTGCTGGGGTaccttctctg
tgctttttctgaTTTGGAGCCCAACATGAGGCTGTCTCAACCTGCATATTCAGGTAT
GGGTACTGAGGACAGAAATTACATCCACTTTTTCAAACTCTCTCCTCacacttct
atttatttcagtttgctcctttctcttttgcaaaTGGCAGATAAGTCTTGTCAGTGTTTTTTTGGCT
TTGCAGcgatttcttttcttttctatctccAGTAATGTGCAAATCTAATGGTTTGCTATTTCTg
ctaaatgtatttatttccaagctCTCTaattcttgtgcttttttttgttgggtGAGAGAAGAGACAAATTTCC
AGCAGTCAAAAAATAAACCATCCGCAGTTCAAGAAAAAATTGAACCCTTT
CTAATCATTGCTATAAAAAGTAATTGTTGCATGTGGGTTCCTTGTCAAGATT
GGAAGGCTGACCAGTAGGTCCCCGAGAAGCCTCTAGTGATGTGTCTGCTG
GATTGGAGGTGTATCACAGGTGAGCAGAACAGTAACTACATGAGCCCTTAA
AGTGGAGTGAATGCAGATGACACTTTTATTCATGCTCAGCCAGATTGCTTG
GTATTGTAtcctttctgaaggaaagtgaaaaatgacACTCTGGCAACAGGCCACTGACTGCA
CAGCAACTTGGCGTGGGTCTCCTGTTTGTCTCAGCCTGAGATGAACATCAC
CAGAGATGCCAGACTGCTAGCCACAAGCTGAGAGTGTTAGAGAATGTCTT
CCTTTAATCACATCTCTGccatttttagagaaaaatacatgaagGAGGAGGGACATTTCCTG
TCTGTTTGGATCCTTGGTGGCACCAGTGGTAGTGGTTTTATCTCCCTTTGCA
TAAGCTCTCCTAGCTAAGACTAATTGGTAAGCTCAGTGCAGTTTATCTAGCC
CCAgatatgaattatttttcaaagcttctcatttttttgcttcccatttccttttattttattgtattttaagGGGAGTTTTT
GAAAGCCCTCAAGGGATTAGGAAGACAAAACTGTTCGACTTCCAGTTGCA
TCTGAGCCACCGTAGCTATTGCAGTGGGCACCAACGTGGCCAGAACCACC
TTGGGACGTTTCATATGGAAACTCCATGGTTTGTAACTGTGAGCTTTCACGA
GATGACTGGGTACACCATGAATGTAACTCACAGCTCAGTCTGGCACTGTGT
TGTAACTGAGGTCTTGGTATGAAAACCTGTGTGGCCACAGGGGATTTTCAT
CATTATACAATAATCCACATCATAAATAATCTTTCCAGGCTCCTTCTTGAATC
CTTCtatgaaaaacagtttaaagAGAGAAACTGGAGGAAGATTTTCCTcttcaaagaacatttaaa
CTATTAATTTTGTAGGAAGCCTGGATATTATACAATTtcaatgtttttatttcatccaAGCA
ATGGCACAATAAGATATACAACAGCCGCCACTGTGGACTAGGAAACATTCG
ACTGAAAAACTGGAGTTTATGTTCTTCCCATTGGAACTGGCTGCTATCTGTG
CCTGCCTCTATGGGGTGGGTATTTCCCTGACTAAATAgttgtgtttttctgaagttaGGGC
AGTATCTTTGCTTTCAGCATTGATGCAGCATGACATGTACAGTAGGACCTGT
CATAATCCATGAAGTCAGTTCTGTTTCAATGTTAATAAGTCCTTAATCAAGC
CATTTGTTTCCTCCAGGATGTCACATTTAGCAGCTGATTGTGAAATGCTTGA
GAACAGGAATTATAGGAACATTATTTAATGCTAAGTGTTATCCACATCATATC
TGAAGCTTGAACATGCCAGGAAAACATCCTTCATATACTGCAAATAATCTAT
CCGAGCTTTCAGGATCAATCATGAATTGGCTGGGTATGTACACCATTTCTCT

TTGTGTGGCATAATATTATACACCATATTCAGTAACACATTGCTTATCTGTTAC
CTATTTTCTATGGCTGGTGCTTTTGCTTTATATAATGATTCTGCACAGCGTTT
GACCAATCACTTTAAATCAAGATTGGCACTTTaaatccaacagaaaaaaaactgaaatttaaa
acCAGTATCCCTCATGCTAGTCTGTTTGTGCTATataaggagaaagaagatgttcaagccaagc
agctgctggtgggtTTAAGTGGGAAGCTATCCAGAGAGACCAAGTTTATGATTTTCA
TCTCCTGCTCTTGATGCTGTCTTCAAGAGGAACAGAATTAAGGGCTAAAAT
CGTACCTTTAATATCTAATTCCCAGAAGTCTTCTTCTTTTCCGAAGAAGTAA
ATAACACCATAGTGAACTCATGGCAAATATTTTCCcaatgcattttccttctgtgtttaaattaca
tttaatgATACGCCACTTTCTCTTATGAACTTGTTTGTCTGCAAACAGATGTGAT
ACATACTGTCAGCAATGTTatattaacaaaaagaaaaaagaaaaaagaaatgagaaacataGCAAC
ACAACCTTACCCGAGCACTATATATGCTATGTATGTTTCTACAGAGCAGTGT
CATTTAGTCCAAAGTTTCTGCAGAGTGAAACATGGAAAAGCTGTGGGTTTT
CTTAACTTAGGGAACAGTTTACATTCGTATCGTAAAGCTGTGTTGTTGTAAC
ATCGTTCTTGTAAACCAGACTCCCAGTGGTAGGTGTTGTGTAGGCACAGGA
GGAAGAGTCCTGGTTGCATCTGTGAGGACAGTGTTTACCTTCGTGTTTTCT
ACAGTTTCTGTTGTGAAATAGTAAAgaaaggtggggaaaaaaataggaattatgaaatatttctcga
gattttattttgtttccacaaCTTCTATCCAAGTTAAAATGGCTTGGTTTCAACCAATGGG
TCAGAATTACTCGGCGTTTCGAGCATTCGTGTTTGCCACAAAAACGTTGTG
TTCTCCTAACAGCTATTTTTCTctatgaaatgctttctttgaatTTAGGCCAAGTCATAATG
AATGCTGGAGCAATTAAAGTATCAGCCACTATAAGGAGTCTTGGGAGCAGA
GGAGACAGACGGGATCAGGGCTTAAGCTGTGGAAATACATTTGTGCGAAAT
AAAGGACTGATAAACCTCTGTTTCAAACAGAATAGTTTGTGTATGCTAAGC
TCCCAGTACTAAACATCTCCTTCTGCCCCCTCCtactaagcaaaacaaaatttagaTACTC
AAACTTTATatgtgaagaaagaaaggaaacagagtatttctaattttttgGCAGGTGCTCATAAATTT
ATGATAGCAGAATAATAACACAGCATGGACTGGTTAGTGGGAAAGGTGCTG
TGAGAATTTAGAGTATGACTTTATAAACTGTCTTAATTATTAAATCTTCAGAT
TCCAGGCAGGTTTAAGTTCATTTCCATGTGTCATAATGTAGATGTTGTGGGT
TTTTAAGCCAAAGTGTTAGTGTCTAGGACTGAATCATGCCTTCATATTCTGC
AATCAATTCAGTTGTTTTCATG
>TCONS_02285168
atgtatttacatatgtatatatagacaTAGATTAAAGCAGCCCCACTTCTGCTGCATCTGgccat
ttctttttgttccacCCCAGCTGGACCTGCCCGCCAATAACAGCCTGGGTACACAgaca
gctcctgctgtgctcgCTCAAGCATTGCACCCACTCCTCCCTCTTGCACGTGGAGAC
TGTGCCACCCCACCAGGTGCCAGCAGGGACGTCTGCCCGCAGGGTATGGC
TGTCtgagcacagcccaggctgAGCCCTCAATTATTAATGTTATTTGGGTAGTGCTGC
CGGGCAGCTCGGTGCTCGGTAGAAATGCACCAGGAAGATGTGACAAGTGG
CCTTATCTTTATGGGCTTTATCCACCTCCACATCTGGTCAAATCTTGAAATTA
AAGGCAACTTTCTGAAACGGCCAACAACTCACCCAGCCCTGCGCGCTCCT
GCTTGGTGCTCATTTGCATGCTATAAATATTCATATTGTTTCAGCTCCCATCC
TTCGATGACCAAAGCGATCGCATTTCAAAGGCTCTATATTTAAAGCACACCA
CACTCAGGTGGCAGCACTGCTTTAAATTTTAAAGAGCTGTGATGCTCTCCA
TGCACTGCAGATGCGCCCAGAGCTCCACAACGCAGGAGTGGTCCCCTCCTA
TCACCCTCCAACCTCAATGGGCACAGTGAGAAGCCCCCAAatgcagtcacagaatca
caaagtcAATatggttggaagagatctctaagatcatctagcccaaccatcaactcatcaccaTCGCGTGCACT

CAActgtgtcatagaatcatagaggttggttggagaagacctctgagatcaccagGTTCAATCCCAACCCA
CTGCCCAcaaccctcagtgccacatctccacattgAGCACCaccaaggatggtgactccgccacctccctggg
cagctgtgccaatgcatcaccactctttcagagaagaaatccttcctaatatccaacttggGTAGAACCATTAGG
GCTGGAAAGACCACTACGAACACAGGGAGGCAGCACTCACTGCTGACTTT
CCCAAACCTTCTccattcataaatatttcaccGATAAGAAATCAGCCTGCCAAATCTTAA
CAAGTGCTttgatgaaaaatacagaaagcctTTTCCATTAGTGCTTTTGATGTTCTTTGCA
TAGAGAATAACGAATGCGGCAGATCGGTCTTCTTACCCTCCTCTGTCCTCCC
TCACTTTCAGCTTTTATCTGGCAGAGCCCAGAGAAAATGTGAGATTTGGGC
AGGAGAAGCACATTCTCTGTGGCATCCTCATTCTTCTACTTCACACACCTTG
GGCACAGAGAACgccaccaggctgcccaaggaggtggtggtgtcaccatccctggaggtgctcaagaaccat
ggagatgtggcactgagggatgtggtcagtgggcacggtgggatgggcTTAGGATGGGTTAGACTTGG
GGAccttagaggtctcttccaaccttagcgattctatgattcaactCACACCTCAGTCTCCATCGTGAC
TCAGTGGGAGGGGACCAGGAGTGTCAAAGCCCTTCAAAGTCATCTTAGGG
CAGACAAAATCCCAGCTTGgttttgcaaatatttgcacCCATGGGCAGGGATTGGGTCC
CTGACACCTCAAGACAACACAGAACCATTGAGCTCCCTACCCTGACCCAAT
GGCATGTCAACTGGGAACCATTTGCACAGCAGATTCACACTCACAGCTAGA
ACCAATGCCATGCTGTTGGATAAAGCTGCTCTGCAATGGAGATTTAACGTAT
TGCTTTTTGGCTAGAGACACACTGCCAGGTATCACTGCATATAAAGGGATTT
TTTGACCCAGTGGGGAAGTGTTAAAAAAGCAATTCGGTCCTCAAATGGAG
CCATTTGGGTGAGTGGGCACCACTGGGAGGTGCTCAACGACCACCAGGTG
CCCATGGGAGGAGCAGCACCGTCCTCACAGCTCCACACTCAGCTCAGCCC
CGCGGATGAAGCACCAAACCCCAGCACTGGGTTTGCAACActttgctgctcagctctg
aatTGCAGCTTTTATCATCTCCTCACACTCCGGCTGTAACATCCAAAGCAATT
AGAgccaactgattttttcctatatttacTCCTTAAAGCAGAGTTTTCTTGTGTGGGGCAGA
GATTACGGGTTTGGGGCTGCTTCGCATTCATTTCCCATTAATGACTCAACCA
CTGCTGTCTCCTCTTGCTCAGATGAAGCTCGCTGCGAGTCCAACTAACTCA
ACAAACATCAATTTAAAGcagtttcaaagaaaacagagcaactgGCATCTAGCAgaatttttgaaa
acaagaatCCCAGCAACACCGAGAGTGTTTAAAGGCACTTCAAAGGGAGCACA
CTGTTATCAGCCTGAGCGGGATGCTAATGTTTGCATGGAGCTATTGCTGCTC
ATTATTCATCCTCCTTTTTGGCACTGGAAGCAGTTGGGAACTCATCACCCAG
GGCTCTTGGCAGGGCTAGCACAGCAGAAAGCTCCCTGCTATCAGCATGGTC
CATTTGGGGGTCTTTGACCCCTCCAGTTATGGAGGGAACAAAGCAACCCTA
AGTCTGCAGAATGTAGCATCAAACCATAGGGTGAGATTGGTCCATAAACCC
ATTGTGGCCATGCAGAGGGCTGAGAGCTGGAACTGCACCCGGTGCTGTGG
GATTGGGTTGGGTTGGTTCTGAACTGGGATTCTGTGTttggattgatggttggacgTGG
tcatcttggaggtcttctccaaccttaatgagcCAATGGTTCTAcaacatggttagtgggcacggaGGGGAT
TGATGGTTGGACGTGGTCatcttggaggtcctttccaaccttaatgagcctatggttctatgaaacaTATC
CCCGTGGGCTATAAGAccagagcatctccagcagcaaTGGCATGGAAGCTGCAGGAGA
TCCTAACCCAcctgctccagcccagcagcagaagtgcagaGCACACAAACCCCAAACACCC
TAAATCTGAAGTCTGCTCCTTGCTTTCAGTGAGAACCTAAGAGATGAGATG
TGTGCAAGATGTGACACTGTGCTGCAGTACACACTGCCCTGAGCAGATGA
AGCACGAATGCCATGGGCTGAATGGACtccacccagcacagctcatctGCAGGGTCAG
CATATGCAAGATCAGGACCTAAATTTGTTCCCTGCCTCCTGGGACACTGATA
ACTCAATTTTCTCGTCGTGCCATGCAGACAGATAGCAGCAGTGTACAGCAG

TGATACAACCCCTGGCTGTCacattgcatttctttgtgcCAAGACATCAAGgttgggaagcagc
agctcaggagaCAGGAGCTGTAGCCCTGATTCTATTGCAGGGATGGTCACAGTGG
GAAGCAAGGCTCGTGCCAGCCCAAATGTTTGCTATGAGAAGCACAGATCA
CAGCTCTGATTATCAAAAGCTGCATGCAGCAAGGAAGGGCTCTAATTTCAC
CCATCGAAAACACAAATGTCACGAGAGAAAATGAAGGCTGTTATTACAGCT
CTGATCCTGTGACCCTTATTTGCAATATGCAGCTTTtaaagaatcagagaatcattcaGAT
TGGGAAAGATCTCTAAGACAGTCACGTCCAATcgtccccccaccccatcacgcccactaac
cacgtccctaCATGGGATCACAGAGtgactgaggttggaaaagccctccaagatcatccagtccaaccatca
acccaccaccaccatgcccacgtagaatcattaaggctggaaaagacctctaagaccatccagtccaaccccaccaCC
CCCGCTATGCCcgctgcccacgtccctcagtgccacatctccatggttcttgaacacctccagggatggtacctcc
accactgccctgggcagcctttctcagcatcacagtcTGAATTTCAGGGTCTGGACCCCTCATAG
CAATGACTGCGAAGGGGTACACTGGTGCCCAGGTCAGGCAGCGTTGGCCA
AGCAGCTGGGGATCCTCGTGCACAAAGGAAGGAAGCACCAACGTGATTCA
TCCCACTCCTGTGCACTGATGCCatttctttctaagaaaaacGAAAccccactgaaatcagcagcG
GCTGCTACATTTTATTCCTCCCTTAATCTAAACATATAGTCATAAGAGCAAGG
TATTTGCTATAAGGAAATGAGCTGTCAAGTGATTTACGGGCTTGCTCTGTTT
CAGCAGCTCCAAGCCTTCTCCGTTGCCTATTTTTAGCAAGCTGTAAATTAAA
GGCAATAAGGGACGGGGTGGGAGCCAGGTTCAGAAATTTATAGAGCCAAC
TTATCTACCTCTCCATTACCCCAACGTCCCATAACTGCAGCATCCAGTGCCA
CGTGTGCCATTATCACAGACTCATAgttggagctgggagggacccTGAAGGCCATCAG
GTCCTGCGCTgtgcagggacacccacagctccatcagtgctcacagccccattccCTGACCTCGGGT
GCCTGCACCACCTCTCCCAGCCATCCAGCCCTCTGTCGAACCTCAATCCAA
TAGCAGCAGTTGCCTTGTTCACATTTTATTGGTCATTCCAGCCACAATTATTC
TAATAATTCCCATCAACAAGAACCGGGGTCCGACGCTGCAGAAACTTTTAT
TTGCGGCGCCTCTGCTGTAAACGATTACAATAATTATTCTCCCTCCCATAAA
GCACTTTCTATACAAGAGCTTTAACAATGGGAAGTTATTACCAGAGCTTTAA
CAATAGAAAGCTATTACCAGTGTCCCTGTTTGCGAGAGCGAGGCGCAGGG
AGATCAAATGAATTGCTGCAGATCATTCAGCGACTCGTGGACAGGAGCAG
AACTGCCATCTCCCAACCCTCCATCCAGCATTGACTCGGGCAGGGAAAAA
GCCACGCATCCTTGCAGAGCACATCAAAAGGGGAAGtttatttcagagaaagagCAG
ACCTTAATCAGCAAAAGGGCCATGAAGAAACAGAATACTTATACAAAATTG
CGTCCTGCTACCAAACAAGATGAAATCCTCCGAGGCAGCTATTAAGGAGAT
CGCCCAGAGGAAGGGAAATTCAGCAAACCAGATTATCTCTAACAGGCTTAT
CCTCTGGAAAAAGAAGTCATCAGATAGCTCCGAAAGACAATTAATGAAAG
GTAGGCGAGAGGGGAAAGTGTAACAGGGTGACAATGAGGGGGGACATCC
ATGGGATTGTCATGGCTGATCTGGAATGAGGGCATTACGcactgctggaggaggaaag
ggatTTGGAGGTGCTCAGTGCCTCGGGCGCAGGGCTTTGCCAGAGCATAGTT
CCCCTATTAAAAAAGCTGTAGGGAGAAGGAATCCTATGGAGCACTGTTGGT
ATCCAAGGGCTGGGTAGACGGGTCAGGGCACGAACCACAGCGCACCTATA
CTGCCACAAATACTGCCATCATCTTGGGAACCCAAGAGCGAGGTTACCACT
GCCACTGTGCACagaatccttaaggttggaaaagaccactaagatcactcAGTCCATCCAACAAC
CCACCAAATGCATGCACTCCTCCAAACCACAcaagggtggtgacgcactgaacaggttgccc
aaggaggctgtggatgccccatccctgcaggcattcaaggccaggctggatgtggctctgggcagcctgggctgctggtt
ggcgaccctgcacatagcaggggggttggaaatagatgatcattgtggtccttttcaacccacgccattctgtgattctatgaa

ggaaTGGCACAAACGGGACCCAAGAAGCCTGGCAAGATCACAGATTGACCT
TAAGctcatttacttttcttcttaaaacgTAAATCTCGAGCCCATAAAAACATTGGCAGCCT
GCACCGGAAGGCGCTCAGTGTGCTCTTCTATCGCTCCGCAGGGCAATAGGC
TTCATCCTGTTTCCTCTGGCCCCAGATCAAGCATGCTCCTCGCAGGAATTTT
CCATTATCCCGTGGCAAACATACCAGGGCAACGCGGCACTCGACTGCCTTA
CACTCTGACCCCTCTTTAGTGGCGAGATCGTGCATAAAGCACAGTCCATTT
CTTGTAAAACACACCATTTCTTGTTATCCTGACACTGCAATTCGGGTGAAA
AGTGGCTGGAAATCCCAGGCTGCAATGGATGGAGGACGGATGGATGGAGG
GCATTCTTGGCTCCCTCCTGTAtcctcctcccagctgctgagcCCTGATGCATTCCCATA
ACCCAATCCAACAGACTCAAAAATCTTGGAGGCACTGGGAACTCATCCCA
ACATCACTGGCTTTACTTCAGTAGACCCTTTTCTTGCACTGCTTTTACTGCA
GTCTGAAAACTCACCCCTGAAACCCATGCAAgcatcccactgcctgcagatgGATCCC
CATATGGCAAGCGATGGGTTACGCCCTGGGCATCACCGCAGAGAGCATCCC
AAAGCTTCACCTCTGGGTAGAACCAAACCCATGGTTGGCAACCAACCCAT
GGCACTGGGCTGGAATTGGATGGTTTTTTTAAGgttcttccaacccaactatCCTATGG
GTCTATGATcattaaggttccttccaacccaaccatcctatggttctatgatctttaaggttccttccaacacaaccatcct
atggttctatgatcgttaaggttccttccaacccagccatcctatggttctatgatctttaaggttccttccaacccagccatccta
gctccatgatctttaaggttccttccaacccagccatcCTATGgctccatgatctttaaggttccttccaacccagccatcC
TATGgctccatgatctttaaggttctttccaacccaaccaccctATGgctccatgatctttaaggttccttccaccCCA
AtcatcctatggttctatgatctttaaggacccttccaactcaactaTCCTATGGTTCTCTAAACCCACAA
TCAGACAGTAAGAGCTGTACAGCCCCACGCTCATTGGGAAGGGGTGACCA
AGGGACACAAAAGATcatgctttccctttctttccagaGTTCTGCTCTTGAATAACGTGGT
GGCTCGGCTCCAGGTAAActcacccagcagcacagtTCGGATCATCTCGGAGAAGGC
AGAGGGCTCTGACTCCAAAAACACATCCTTAATTACATGTGTGTGAGCTGT
TTTCCTTGGCCTGGTTGCAGGGGACAGAGAACGCCGGGCGTCTCGCCCCG
CAGACCTCTGCATTCAACCCACGCCCTTCGTTAAGCAGTCTGCAAACGAGC
TAAAAAAGCCTTAATCCTGCaaacagcttctgcag
>TCONS_02316836
CTGAAGAAATTGGACAGAAGAAGCTATCCAGTGATCACTTCTATGTCTTTC
TTTACAGTTAAGCTGGAATTTTTCAAGATTACAGAACTGTTTCCAACCACTG
TGAGATCTGTCACCCTTTCTATGCATGCAGCTGATGGCTGAAGTTGGCTATC
AAGAAATTCCCTTGTAGCATCTGCTTCTTGAAAGTCTGTGCAGCACGCTTC
AGGACATCATTACGTCACTGATGCGCTCCCAGGTACTAGGAGAACCTAAAA
CCAGCAGTGACGGATCACATCTATCCTCGAAGCTGACATATTGCCTGGGAA
ATCATCTTCCTGGCTTTGAGTGTAATCCGCTGAGCTTCCCAGCCTTACAGCG
TGGTGGAAATGTGGCTCTGGCTCACAGCCACACAAACCCAGTCACTTTAAT
GAGTTCCTGTGTTTATAACTCCTGACACTGCCACAGCCACCAGGTGCTCTC
AGGATGCACGCTCAGGACAACCTCAGCCTGACTGACAGAGCCTTCTGGCT
TCACAGCCCACTGGGCCCAGCATGGAGGTAATGTAAAAGTGAAAAGGGGA
AATTGGCTGCAAGTTAAACACATGTCTACACATTTTGATAATTCAGGACCTC
AGGCCATGATAGTATTGCTGAAACTAGTGTAACAATTTATCAGAAAGCACCT
TACAGTTTGTCTCTTACATTTAAGGGAGATGTGGATGGTGTTCAAACCCATG
ACTGGTTGCACACACTGATTTCTTC
>TCONS_02392564

GTGGATATGTACACACCACTGAATCCTAGGAGTGAAGGGTTCTTCCCTTGG
CTTGGCAAGATGTGACATGCATCCAGAGGGATACACGCTTGTGTCTGTGCC
AGAGAGCTGTAATCATCATGTGAGTTCCCAGCGTGTCTCAACgcccagagcagag
ccctgGTTTGAAGATCATCCTGAAGTGTATTATTCAGCTGCTGAAATGATGCTA
CAAAATGAGTATCATTTTATGTTAAAATGCACGCAAAgccaaaggaaaggaagaatgat
GTGCTACATAGTAGGGAAAGAAGACCGAaatacagcagcaaaactgaTACAACTGAT
CCATCAGAGGAAGCTGGCACCACATCACGAAGCTGTGTCATCCCCAGGTA
AGATCCTCACTTATGCCCTTTCCCC
>TCONS_02402859
GGGACTTTTTActacatttttgctttcaaaagtaGGCATTCCCACTGGAAGTTCTGACATC
CAGAGCCCTGCCAGCTTGTTTCTGAAAAGCTACAATACCTCCCCAATGGCT
TTTGGAAGTTAACTGAGACTGTACTGAGTACCTGTAGCACAACTAGCACTT
GGCTACCAAAGAACCACTTATCTCAGCATTTCCACCTAGGTCATGCAGCCA
AAAGGGTGACCACACCTGACTTAGGGAATAATGCACCTATTGTGTAAGAGC
AGAAGTAATCCTCACCCACAGTTCTGTCTTGTTCTGTGCATGACTTGGACT
GCCAAATTTGAGATACCAActcatttgtgtttctgtgagaCAGACCACTGGTTGGCACTG
AGCTCTGTCCTACACAAGTCAGAAGAGTTCAGACTGCCCCTGCAGGGAGC
CTGGTGCAGGCAGGGAACGCAgtggaaagctgcagctgtttgctaTCAGCCTGAACCAC
ACAGCACGAAGGAGAGAAGGAACTTTACTGTGGTGAACTGCTGACTGACA
GGACTGGGGGTGAGGATGCTCCTAGCATGGAGGTACAGCAGCTTCTGATGT
GGAACTTCTACCTTGACATCACATGCTGACTGCAGAACAAGTAACAGGTGC
TGCACGTGACAGAATCCAGTCCAATCCCTAGCCATGACTGTGGTTAGGTAA
CCTTGGATGGCTGGATGGACGATGACAGTATACTCAGGAAAGCTGTTGGAA
AGCGATCAGTTTTCAGACCAGTACTCATAGCTGATCTTGGGACTGTTTTGG
AGATGGAGCCGTTGTCACAACGACAGTGGAAACTGCTTTAGCTGAGCCAG
ACACCGTTCCCTGCTGGAGATGGGAGGCTGGTACTGTTTGAATTCGCAC
>TCONS_02404676
TGGCTTAATGATTTACTGTTCCCTCTGCTCTTATCGCCTGCCAGAGGAGCTG
GCTTCTTTTTGTCTTAAAGCTAATGCACCAAATCACCCAGGAAGGCATGCA
GCATTGTCAGGAGGACGgtTTTATTAGTGCATCAATAACGACTCGGTCAGGG
CACCGCTTCCATGTCTCCAGTTTGGAAATTACGGTCTAAAGTTCGCGATTGG
AAACAGCTCACAGCCATCAGCCGTGCAATTAACTCTGCCCTTCAGCCTCCA
CAAAtcaggcagctcagagctgtgctctctcTGCGGGACCTGCAGCGTtggtgtgacagcagcagac
CCCGCGGGAGCGCTGACTCAGGGATGAAGAGAAATGGCtttgcttgcaaaaaaaagc
tgagctCCGCCTGGATCCCAGCTGGAAATGCCTCTGGGTGCCACGTCTGGGAA
TAGCACGGCTAATTCAGACCCTCCTGGGAAGGTCctggcctcctgctgctggttcTGAG
GGTGCAGGAGGCTTCGGTGGGTGGCTGGAGGAGCAGCCATGGAAGTGAG
ATAACGACAGCCCTGCAGGTGGAAAAACTGCTAAAGTGGCACAAATGGGA
GCTCTCCTGTCTGACATCCCCcatctgctcagagctgcttctgGTGTGAGCAGCACTTCC
ATCACTCCCCCAGGAGCCAGACCCCTCATGCATTCACGCCAGCCTAAAGCC
CCGCTCTGAGCCATTTCATCCCATTATCATTGTTTTTCTCGCATTTCTGCCCA
CACCAGTGACATCGAGCCATCATGTTATTATCTGTCAGTAATACCTACAAGC
CCAGCTGCATCCGCAGCTTTCTGCCTACAGAAGACACGTAGGGGTTTTCCT
CTTAGGTTGAAAATCATCATCATAAGCAAAACCTGCTTTTACCCCATCAGAG

CCCATGGCCACAAAGCAGCGACCATCCCCCAGCCCGGCAGAGAGAGCTgag
caataaaacaaattacCCAAAGCCATCTTAATGACACAGGCTCGGGGCAGGCGCTTT
GTCAtcctttgcttctctttacgatgtgcatttgtttatttgtggtTTTAAGGAAACGGGGTCTGGCCTT
TAAAAACGATCTCTTTGGGGAAACACGTGTTGCCATCTTAAAAGCCATTTT
TCTACTGCATTTTGCCTGAGAGCCTGGAGTGCTTTCTCGAGGTGCTGGTTG
CCATGGGACTcagccagcccagggctgcactCCCACCGCCCACCACGACTCAAAACccc
acactgctgccaccagaAATGCCACAGAGTCACCATTGGCAGAAGGAGAGGCAATA
ATATGCCCTGAAAGCAtcccaaagctgctgcttctgcactcTCATACAACTACCAAGGATG
GAAAGGAACTCTCAGATCCCCAATCCAAccccaccccactgtgcccactgcccacacccctcag
tgccacatccccacggctctggaacacccccaggggTCTCCACCACCCCCTGTTCCAATGACCT
GTTTACCAATCAGCCCCTGactgctccctgctccttcctcccctgCCATTTACCCAGGGAGG
TCCCCAGCCCCACAAGCAGCATTTCCCCTTTGCTGCACCTGGAGCTGTTTT
TGGGCAGACCCACACCACGACCcaacagcacagtgctgcctttgTCACGCCTCGAGGA
AAAGCCTTTGTATACCAAACTATTTAAGAATCCATTTCAGTTCTGATATACTC
AAGGCACAAGCAATTATTCACCTAAAACTCCCACTCTTTGGCTTATTGGGC
ACCTGGGGCTGCACcacaaaactgaaatgctgctggtTTCCAGCTGgtaggaaggaggaagaaacc
TTCCCAGAACAGAGCCCTGCTGGGGGAGGCACCGGCCTTGGAGAAGGGCA
TTGAAGGACTCATTCCCTCCTCATTTTCAGGCACGAAAAGAGGAATTTCTG
GGCAAttctgcagggagaggagggtgGGGGAAGGCTTTCAGCTTCAGTGCACGTAGG
GTGATTTCAGCCCGGCTGTCAGGCAGAACCTTTGTGTGTAACTTGGCTTGG
AGCAGAGCCGGCAGTGGGGAGGGCTCCCTGCACGCAGCCCCACGAGGTG
CAGGGGGGGCTCATGAGGACCGCCATCCCGAGCAGCCCTCCTGCCCTCCA
CTGCATTTGGACCCCCCCTACTACACTGTACTCCCAACTTCCTCCTCACGCA
GTGTTGCAGCCCAGCCACCCATCACGTCCCACTGCTGCGTGCAGCCTGCTG
AATTCTTGCCTACCCCATCCCACGGCCCCCACCACTGCTGGGACATGCCGG
TGGGACACCCCAGCAATGGGGCAGGCAGAACCCCCACACCCCCACTTTGC
TCTCCTTGGAGAGCACCTCCTTCCTCACCCACCCACACCCTTCGCTCACTAT
GGACTCAATCCAAACACCAGATTTCAGTGTCCCCCACCCTAAACCCACCCA
TGGACCCTTTGGACAACCAGGTGggctccttttcctccctgctaTGGAATCTGCAGTGC
AATACCAAAGGCAGAGATGTGGTCACCTGTGCTATGCCATGCCGTGCCGTG
TCATCCATCACCATCCCACCCCTGCCCAACACACAGCCCCTGCCCGCAGAG
CCCCACATTaccttccagctgctgctgcacctcgCCCAGGTGAGCTGTGCTCCGCCTCAAG
CACCTCCCCCCCACCTGCAGCCGGGGGCCGGGCACACCAAAACCCCACAC
CTCAGGGCACACCGAGCAGCTCGTTTGGAGTTTCTTCCTTCAGAACCTTCC
CA
>TCONS_02429461
GTCCCTGCTCTGCACGTGGATGCTACATTGCTTTCTCAGGGCAccatcagcacagc
caggaACTGACAACCTACCAAAATCATGAGCTGAGAAGCAAATCAAGCAGCC
CCTCCAGCTTCCCCATGAGCAGTGGGACCAAGCCCCAGGTGCTGTCCTACC
ACcccaggagctgagctgcagcacacacagccaggCAGAGCCCCAGCTGGTGCCCACACCA
GGTGAGCGTGGCCTCATGTGGCCCCGGCTTTCCAGGCTGATGGCCAAAGA
GAGGAGAATGTGGGCTGGATCTGTGTCACGCAGCATTGCGGGCTCTCCGG
CTCCCGCAGCAGAGTGaaggcagctccagcccaggaggctcagccagagctgctctgctctggttcTCC
CAGCACAGACACAAAGTCCCTCTCCTGCTCACACTGCTGAGATGAGCCGG

CCCTCCCCAGCCTCTTTGGCGTCTTGCAGGCAGCGAAGCGAGGAGATCTG
CAGGCAGGAGTGGCTCAGACTCCccactgcagcctccctgcttggcattgctctctgctgcttccctgc
gAGCTGTGTTTGTGGGTAACACCCCCAGGGGGAGACCCTGACATGCACAA
AGCCAGCTCCCAACCAGCGAGCACTGGAAGCAGGCACACGCATGCAGAG
CCTCTCCTCAACTCTGCTTTAATATGCATGCAATATggccagagctgctctgcatctGAC
AGTATTcacttttcagaagagaagcagcagcagccagcaaacAGCACGCATCTCCCTGCCAGAG
CAAGGTCGGGGCAGGCggggtgctgggagctgcggcTGCACATGGGATGGACTCTTT
TGAACTCAGACATCATCTTCAGAGCACGATATGGAGACAGCAAAGCTGTCA
CAGTGGCTGCTGTCATTTCAGCATCCTCTCAGAGCTGCCAGTCTGCaccagctg
cagcctgccctgggTACCAGCGTTACCAGCACTCATCCCGTGCCCACGAGCCCTCC
CAGATGCACCACGTGTGGCACCGTGTGTGCCTGGAAGCTCCTTCTTCCAGA
GGATCAGCCTCAACCCAAGCTGGTGGTGAGTGGGGCAGAGGCAGACTGCA
GATCACCGGAGCACGACCTGCAGCACCTCATGCTTTGGGGAGGAGGGGCT
CAGGCAAACCCCCACGTGTGCCATAACTGCTGCTTGATGGAGACagatttctctttc
ctcctccccagaaTCACAACTTGGATGATAATCCCTGGATTGCTGCCATCGCTTTGG
GGCTGccatattaacaaaaaaaaaaagggttctCATTTGATCATCTGCTTGCAGTTAATTTAG
TTACAAGAGTTTTGCAGTGATCATTTTTGAGATGGCAATCGGAGCATCTCTG
CGAGCAGAAATGGCTATCGTTCCCCATCTCCTTGGTGTTTACATGCAGATAA
AGACATTTTGTCTCCTAAAATGAAGCCAAaatcagccccagcactgccgATCGTTGCA
CGGCTCTTCACGAGTGCCGGTGTTGCTTTCAAATCACACGTCGTCAATAAA
TCTAATTCTAACTGCCCTGCAGCAAATGGCACCAGTCTGCACTTTGAGATCT
CTCCCACACATACTCCACTGCCTAAAAATACCAGCGCTTTAATTCTCAAATG
ACTCGGCTATTGCACAGccaccttctttttctttaatgtacACTGCTGCATGGCTCGGTGCA
GGACATCTGTTACACTACAAAGGGTTTGGGGAAGCAGGGACCAGGAGGGG
AAACTCATCCCTGATGTCTCACCTCTGCATCAGCCAAAATATAACCTAAAAC
TCAACCCACTGCCAAAGAGAAGGCCAGTTGTCTCTGCTGCCCGGCCCAGG
CACGCTGGCAGCACTCAGCCAGTGTAGTTTCTGCCTCAGGACAGAGCACA
TGTGGATCTCAGACAGCATGGACAAAGCAAAGCCTTTCTGGACCTGggctgga
agaaaagagaacttTTATCAGGAAAGGTGAGCCTGCTGGTCAGGAGAGCTCACCAT
TAAGCTCTGACAGGGCTAAGATAAAGCCATGTTGTTGTGCTCAATGACTGG
GAACTGTAGAGCCCTTGCTCTGCCCTCAGCATCCCAAAGGTGCAGGCATCC
AGCACCGCACTGCCAATGAGAAGCAAGGACAGCAACTGTTCCCAGGCAG
GTGGGAATCTCCAGGACTTCCACTCATCAGCTCTTCGACACAATCAGCTCC
TCCAAGTGGGAAAGCAGTCCCCAGCCTTTTGTTTGCTGTGGGTCCTTGAGG
GGCTTTAGGTTTGCCCAGTGTGCTACCCAAACCCAGGTACGGGGCAAACCC
ACAGCCTGGCCAGGTTCTCCCATGGATGGTGCTAGGATTTCGAGGCATGAT
GCTCCGTCTCACCTCAGGGATGTCCGGGTTGGACCCATCCCCAAAGGACA
GGAGAGCACTGACCTGTATGCCTTGGCTGTGGGGCTCTGGTTGCTTTGAAC
TCCACGGCAGGCTCCCAGCCCACCCAGCCATCCAGGTCCTTTAGGAGCTCA
CTCCCAAAATGAGAATGTCATCTCACAACCTCACCTGGAGAAATCACGCTC
TCACAAGGTACTCATGTGTATGTGGGACAGCTTGGGACCAGAATAACTCCC
CCCCCCGGCTGATCAATGTGTGAATAAATCTGCCTCAGCCAAAAATAACCT
CTGGacctttctccttcctgcagtaCGCTTGAGGGGGGGAAGCCAGAAAGAGGCTGAG
ATCTGGTTAGTGAGTACAGCTGCCAGGCACTGGATTACAGGGAAGATTAAC

TGGATGTTTGAGGGTGTAAACTTTGTATGAGCCCATCTTTCTCCTCACTGTC
TGCAACCCCTGGCACCACAAGTTCTTGGACAGGGAACAGTACCAGGCACC
ACCAGGAAGATTAAGGGCTTCGGATCAGGTTTATGACACATATGTTAGTGC
CCTGGCAAGGCTATAAACACCCTTCCTCGTTTTGGAAGCCTGAAGGGCCAG
GCTTGGCAGGAGAGGCTCCATCCCAGGATGCAGCACACTCACGAGGCTCC
TGAAGAGGCAGCCACCCAGGCGGTGCATCCTTACGCCTGCTAAGAGAAGC
AtggaagaaattgaaaaatgggagctgggaaaaaaaaaatctgcatttcagaCAAATTCCAGCCTTGTCA
GTCCTGGAGGATTACTGCGCTGGGGACAGCGACCTGTGAGCTGATTTACAA
CACCGGGAGATGGAGatacaggaaaggaaaggcaactTTGAAGAGGTACAGTCAGTCC
CTTCGTGCCTTTGGAGAAGTTTTGCCACCCAGTTGCATGATGCCTCCACGT
GTGGGATGGGAACCACTGCACTGTGgtgcagcacaggggcaggaaGGGCtgtttccagcaca
gctctgcataaAAATGACCGAGAGATGAAAACCCACTTCATTCCTTTGGGTGTCC
TCACCATGCAACCTAAACTGATGGCTTCACAGTGTCCCCACAAGCATCTCA
ACCTGCTTGACCACAGCACAAACCACATTAAAATCCAATTATATCAGCACA
ACTCTTCTAATAGCAAAGGGCTCTGAATCATTTAACTACTTCACAGCCTGAA
CTGATGCTGGAAAGTCATCCTTGCACCTGAGCATCACCAGGCACCAGCACC
AGCCAGCTGCTCTTGCAGATCCCTGACAAACCTCCAGGAAGACCCTGGAC
ATCAGTCCCACCTCCCAGCCATCACCCTCCCGAGATGCCCACAGGGATGTA
TGTGTTGGAGCATCATCAGGAGCAGAGATACCCATGACTCCCAGAGCACTG
GAGCCACAAAGACACCAGCTTTCCATCTCTACAGGCAGCACCACAAGCAA
GAAGCTCCTGGACAACGCAGGCACAACCATCCAGGTACCAAACTCCAGCT
GACACCTTGTCTCTGTAGCAGCTCGCAATGCAGTGCCATGGTGTCAAACCC
ACCAGGAGAACCTCATTCTAGCATATAAGCAGATGGTGGAAAATGAgcttaagc
aaatattttgccAGCACCTACTTGGCCAAAACTGGGGCATCCCCAGAAGGTTTTGT
ACAGCTTTAACAAAACCTATCTTCCTAAGCCAATTGTGGTTATAATGATGCA
ATTTGGGGGATAAATGAGGCCCTGAAGGGCTGGTGTTATTCAGGAGGAAAT
CACAGTGTCTGGAGGCAGCTTGCCATGGCTGTATTGAGACGGCATCGGCTG
ACAGGAGAATAGGAACcagtgtgagcacagctggaggcagcCAGGCTGGCTGGATGCTC
ACTGGGCTGATCCAACGCCAGGCTCAGTGAACACCCACCCGGTGCTGAGC
TCAGGACCATCCCTGCGAGCTTCCCCGTGAGCTGGTCCAGCCCCAAGCTCA
GTGCTGGGTGGATGCTCAAGGGCTGATTCAGACCTAAGTGGATGTTTGCTG
GCTGGAGACAACACAGAGGGAGGCAGGAGGACATGGCACATGGAAATTG
GAGCAGAAGGCCACAGGTAAGGACCGGTCTCATCAGAACATCCACCCATA
ACAGAAAATGGGAGCCAGGGGTGCAAAGGAATAGCTTGGGACCACAGTG
GGGGGCTGCTTGGGGCAGAAGgcagctggaaaagcagagaacCCCCCCTCTCCACTC
CAGGCTTCCAGGAATCACTGCCCCACCGAAAGCCTGCAGCTTGGAGGAGG
TCTGGCCACCTCTTCACCCCTCCTGCCCACACATCCAGTGGGGATCAGGCA
GCTCCCAGCTACGGGGGGCACAGCAATGCCTGGGCTCAGATTCCTGGCCTA
TGGGTGATGCCTATGGACAGAAAATCCCGAGGGTGAGGATGACAACCCCA
CACTCAGCCTCTGTCCAATGGAAGCAAGGATGCTCAGCACATCCCTGACTC
ACAGACCCCCTCCAAGCGGCATTGGGGTGGGACAAAGCTCCCCCAGCCGC
AGCACCGTCTCCCGCACGGCCCTTGGGGGCAGCGCATCCCCACCTGTCAC
GGCGGGGCAGAGCATccccagtccaaccccaccGGGGGTAACAACGGGGAGCGAAG
CCCGGCCGGCCCATCCCCAGGGAGGATGCAGAGCATCCCTCGGGGCGGCA

AAACGATGAGAGCTCCCCCGGGCAGCCCCACCGGGTGGAGGATGCAGAAC
GGAGGCTGAGCATCCCCTGCCCGGCCCCGCGGATAGCTG
>TCONS_02432575
ACATGTGGTCCCTACTCTTCACCTGCATTCTCACAGCACAGGGAATGTTCTG
GATATCTGGGCTTCACTTGAGCCTCCAAACTGGAGCTGAACCCTCCAGAAA
TTGTTATTACGTAAATGCAAAGAACAAAGGTGTTGTAAATAAAGGAGCTCT
ACAATCTGgtttttgcagctgctgtgctgcgaATGTGTGAAGTGTAAGGAATCTTTGAAAG
ACATACCCAAGTCTTAGTTTTTACACTGAttaagttttgttttggtttttttaagaGCCTGATTG
TTCTTCAAAGCCAGCTGTGAGGAGTTGTGCACCGACTGCGTACCAACAAG
TGACTTCTGAagtgcaaaagcagaaagaatcaGATCCCATCACCAGAGACTTTGCAACC
AAATACACACAATTCACGAAGTTGGAGCGCTGTGAAGAAtgtctccagctcctgcaga
aggAGGTTCCTGTCGTCTGCAGGGAACCGTCGCTGTGCGCCTTTCTGAAAGT
CAAACAGCAAATCCAGGCTCTCACCTCAACCTCCCAACCCACCAGGGGAT
GCACAGACCCCACGCTGAGCATAAATGGACGTTCAGCTGCATGCTCCGAGC
TGAAGCGTTCACAGGCTGAAGGATAACTTGCAGTCGTTCACAcccaggctgagct
gctggcactgtgTTCTTCCCCAGGATCTGCACGTTACGACATCAGCTGGAAGTTCA
GAGCCTTTGTGTATTTActgcagcagctggatgaCTCAGTCCTCAGCAGCAAGCTGA
GACACAGAGACGCTGTGGTCACGGAGCTCAGGAGCAAAAGGGCACCTGG
AACCCACACCCCAACTACAGTCTGGCAGAATTGAACGTTATGGAAAGCTGT
GTGTGGGTATGTGATGGACTGCTGCTCGCACAGCGGGTATTTGGACTCCCA
CCTTCCCTGGGCAATGAATTGCCCACAGGATCTGTCACAGCACTGCGTGGG
CGTCAGGTTTCCTTTGTGCCATTTGTCCAGACCTCTCCGTTGTGTCTGATCT
GATGAGAAAGATCCATTCCAGGAGGATTTGTTCGTAGAACTGCAGAGTGGC
TGTGgttggagaggagctctgctggcTACCCAGTCTCGCTTTGTGTTGTTAGTGGACATC
CTTGCTTGTATCTGAATTTGTAAAGAGCAATCAAACCTGGAAAACAGCATC
TGCCATTTGTTGCTTTTCCAATCCATCAATTTAAATGGTTGTTTTTCCCACC
AAGAAGTAAGAAACAAAGCACTGCAACTTCGTCTGCACCTCCCAGGAAGA
ACCTGACCAGCAGCTTTGCTCTGGAGCTACAGAtatgttttctgtctgtgaaaGGAGGT
TGCTGTTTACCTACATTTTATCTGTTCAGAAGGCTTTCCCTAGACTGGAAAT
CAAGCTTGTTCAATAGAGCTCATTAGGTAATGAGCTTACTCACTAAGCTCAT
TAGTAGGGAAATTCATCCCGCTCAGCCCTCTGCAGCCCAAATCTTGTCTCCC
TGAGCCTAGCTTACTGCTCATTTGACCCTTAGCAAGAGAAGTGACTTTTAC
CTAATTTGATctcttttgcatattttcattcAACAACTGCCTTGCTGAATGAATGTTTCTATT
ATTTAATCTGGCCTGCTATtcctctgcagcaggaaaatatCTACAAGGGCTGTAGTCTTT
GTGCACAACGTGCCAAGGAGAGAATATCATGCCAGCTCATTTCAAGAACTG
AAGCCGAGCGTCTGACTCAGTAGGACTCATCCTGTTCCATGATGGATGCTC
CCTGGATGGAGGAACAGACGGTGGGGCAGATGCAAGTAGGCAGGAGAAC
GAAAGAGGCACGAGTAGAAGTTGTGATTGCCTCCTGCGAGCTGCCCGTGA
TTGCCCATCTGCACGCAATGGCGTGGTCGTGCAGGGAGCTTtggagcacacacaca
gcagaagaaagggatTTCATAGTGTGGTTCACTGTGCAGAGATCAGAGAGCAGGTC
TGACAACGAGGATCTCTCACGGGGTGGGAGATGGGCTGCTATGCCTTCCTG
AACCGATGGCACAGCTGAGACGTGTGACAGCGAAGGACGTGTGTTGGCAG
TGGTGCTCCATTGATTCATGCTCAAAGAGGCACATTTAGTCCTGTTTCTAC
TCATTTCAGGCACTGATTACTGGAAAAAGGCTCTCGTGATGTCATGGAGTT

TGTTGGATGGACAACAGCCTccttctgccttccctctgaagaagcagagagaagcagttGGAGCT
CTTCTTTAATACTGATGACAAAAGGTCAGGCAAATATTGAAGCCTCAAAAG
ACAATAAAGCAGGGTtacaacaaaaatgaattctgaGCACAGGGAAGGGATACGTTCC
TTTGTACCCCAGCAGTTGTGTGGCTCAGAGGGAGATGAGCTCCGCAGCAA
AGGACACAGTACCCACCCTGCACATATCTGGGGCATCCAGTTTGGGCCAGT
CCTGATCCAATGCATGGGCACCTGGGCATGTAAGAACACGGGCACCTGAAC
GCCCCTGGGCAGTAAGAACACATCAGGCACGTTCTATCAGCCTCCATCCAG
CCGTGTTTCTCCAGAaggagtgctgctgggcagctctgGAGCTGCGCTATGAGCCAGGC
AGCAGGATGGGATGATGGAGAGTTTGCTTTCAGAGCGCACACCTGATCCA
AGCGGAGGCACTGATGGAGATGGGCTCCAGCTCGGAGCAGCTCTCTCCTT
GCAGCTTTCCCTTGGAAGAGAGCTCTCTTCTGCCAAGCATTAGGTTGCAAG
AATAGCTGGCGTGCGTTATCCTTTCCAATTGAAaaagaatgctttatttttgtccttACCGT
GGTGACTGTAATTAATATTTAAGGAGGATGAGTCCCTTGTGCTACTGCTGCT
ACCAAAATAGAGAAGAAGTGCGTCCTGTTGATGACATGTCGTGTACAAATT
ACACACCAGGAGAGCGTACTGAGATGGAAGTGAGAACTGTGGCGACCGG
GTGAAAACacaagaaggagaaagcaatAGTAAAAGTGTTTTCCTTCGTACAGAGAAG
AAATACGGGGGCTGTCAGGTTGTGGGGGCAAACCTTGTTATCTCAGAGCTG
CGCATTTAAACCTTAAAGGCTGAAAGCAAATGGGAATTTATCATCTGCTGTT
TTCACATCTGATGCGAAGCCCCATGTCTACAGGCaaaggagaaacagcagcaatttgTG
TCTCTTAGATGGAGTCGCATGCACAGAAGGAATAAGGCAATAAGGACACG
AGAAATAATTATCTGCACACAAAAGCTGAGGGTACTTCAGCACGCTGAGGA
CGAGTTGTTGGGCTAACAGCAGCTGCCAAAAGCCAACCTGacagctcccactgctg
ctgcaaggcagcAACTCGGGCAGTGCGGGACTGGGCGGCTGCGGGGGTCTCATC
TTTGTTCCTGGGAGGTCTCATGGCATCGCTGCACTCTGAGCCCGGCTCTGT
TTGAGCTCAGCTTTCAGGGGCACGTGGCCCCATTCTGcggggctgctccctgcagaag
CAAACAGTCCTTCTCAGGGGCTTCGCAGCGGCCAAATTTCGCGAAGTCCT
GAAGGCAAAGCGCTCGGGCTGATCTGTGTTTTAATTCTGCTGCAGATGGCA
GGCAGAGATGAAAGCAGCAGGCTGCCCCGGGCGCTGCGCTCCGTGCCTCT
CCCGAAGTCccgtggcacagcacagctctgcacggCTGCCCACCTCAGCACGgtgctttcagctg
tgtttgtagGGGAACGGACCCCACGTGCAGCACTGAATAGCAGCGTGCAGCCCA
GACCCaaaccccacagccctgcacagacGTCCCCGGCGCAgagccaggagaagagaaagaagtcGG
TAAGGAGTGAGAGGAGGAAAGGGCCAGATGTTGCAGCATCGTTCCTCAGG
CTGCAAACCAAAACGAAAAGCCAGGAAAGATCTGCACCTTTTAAAGAGGT
AATGAATTGTTTCCCCCTCTGGCAGGCAGCGATTTGACTTGCCACATACCTC
CGAGTAATTTCCCAGCAGGTTTTAATGTTGGAACAGTGGCATTCTGCTAgatat
tgattttaaaataactctTCCACCTTTACTTGTAACCTGAAGAGGTGAATTACAGCCCT
TGAATGTGCTCTAACGTAGTGCTGGTAAATGGTGGAACTGACAGCGCCTGT
CTGGGAAAGCCatggggctgagctctgcccgGGGCGGCCAGCAAAGCTGGGATGGGttt
ggcacagagcagccctcagtGTAAGGCAGCTGCCAGGCTGAGCCCTCTGCTCCTGGA
GCTTGTGCTGCATTAAGCACCCCCAAACCTGCTCTGTCCTCCGGTAgcaccaca
gcagctctgtcccttcGCTGCAGATGGGCACAGTGATGCAGAGCCCAGCATcgcagcagt
gcagcaccaaAGCTCCGCACCTCTCTGCTggtcctgcagctctctgtgcccGTGCAGCAATGC
ACGCTGAGAGCAGATTTGGACTGCGTGGATGTAATCAGCTGAGAGATGTAA
TTCGTTTTGGCTAATTTGTTGCAGGCCTTTaacttcccttcccctttcacCGCCTTCATAC

TGACAGTGGCTACTCTGTCCCCTATTTTCAGCTGATTGCTGTAAACTAATTA
CTTTTACTGGGTCAGCACTGTTATGAAATTATCATTTTATGAGACTAAAAATA
GCTCCGATCCAAATCAGCAAGCCAATCACTTCCCCTCATCTCCAGTGAAAA
TACAATGCTCTCTGCAGTCTGCGTCTCTCAGGCACTCGCTGTGCTGCCGGC
TGGGGAGGGAAGCAGGAATGAGGAGCTGCGAAAGCTTCCTCAGCTCAGTT
GCTATAGATGCATCTCTGCCAAGGCTCCCATTGGTAAGCACAGGAGTCGTG
TTGTGGTTTTAGGCAGCACTGTTACTCAGCATCGTTCAAAACCATGTCCTTC
TCACACGAGCTTGCCTTGATGGAAAGTTATTTGGCCTTGCCCATTAGAAGT
GGGGTGTTTGCATGTGCGGGCAGTCATGACAAGCAACCAAAGCCACAAAC
AGCCCAAGGATGGATGCGTTCCTATTTTTCAGGGTTGTATTTGAGTGAATTG
AGAGAGTGAAAAATCCACCGAACTCCACTTGCTACAGGAAGGAATAAGGG
CATTTTAATACTTCAGGTGAATGCAGATAGCTCGAAAATGAGATGCTTGCTC
GAGATTTGCATCAGGGCAGTGAAGGAAAAGGTTCCGGCTCTGATCCTGATA
CAAAGATGCTCCGTGCAGTGTCTGTAAATCCAGCAGGGGAAGAAAATTGAT
ACCACACTTCATATAAGGTGAAGCTGGAGTCAGGAGAATGAAAGCTCGAG
CAAATGGCTTCTTTGTGTAGCTCCATCAATTCATTGTCTGATTGGGCTCTCT
GTGTATTAAATTCAAGCTGCTCCCTGGCCTGTGGACAGAAGGATTGTTGCC
CGTAGGAAATGGGGAGAGTGATGATGTGCTGCACCTCGCTTTACAAAGGA
CATGTCCCACAGCTCATCCATCTTCCCACTGGCGATGTTAGATatgcaaagcagcatt
tctatCAGAGCAGTATTCCTGATTCCACTCAGTGGCAGTTGCTGAGGTGGTGA
CACACAGCATACGATTACGGGGCTGAGCCTCACGCTTTGCAGAGCGCCCG
GCATGGCAGCGCTCCAGGGCCGATTTATGCCTTCCCTGACTTTGCAAACTC
CTCCTGGGTTGCATGGAGGAGGGCGAATGGGGGTTGCTATCAGCCCATGGG
CCCCGGGGCCGtgcatggagcagtgctgcagagatcCCCATCACgcagtgctggcagccctgcagtgct
cagcgctcagccaggctgtgctgctcgGCCGGCAGCGCTTTGGCACTGCGAGTTTCACAC
CCTGTTCCACTGCTTGCATTTCATCCCATTGCTTCCTTCCCCAAGACTGAAT
AccagtgaaaaagaaagccCAAGAAATCAACAAAACAAGTAACAAGACAGTTGATT
ACATCGGCTCTCTCTCGCTTCCCCTCCTCGTGCTTTGCAGAACCTCGGGCTC
CAGCTGGGTAACATCAGGACAGTGGAGCAGCCCCCAGGCCTGAGCCCAGC
CCCCCCTGCACTCAGGATCTGtgcactgctcccagcccccccagcactcagagcaggctgcagaCA
TGCAAGGCCATTGCATCTGCTGTATGACTTCCCCACGCTGACGTGGCTCCTC
AAACAAACTCTTCGGATACGGTTCTGCTGTCCCAGCACAGTGACCAGCTCA
GCCAAACCCAAAGGGACAGCACGGACCCCACGGGGACAGGTGTGGGCAG
GGGGGTTTGCAGCCACGCTGTGCTCACAGAAGCTGCCTGGGAGGCTGAAc
atactgctgctgtcaggtgaaaggaaaacagccaTCAACAGCAAAGGGAAACATGACATGGG
CAGAAGCAGCGCGGGCTGCAGCGGGGGGGTCCGGCACTGCATCCCTGTTT
GTCACATTGGGGCACAGGGATGTGGGGTCGGCCCCTCTGGAGGCAAAGCC
CCATTAAAAGTAAATGGAAGCAATGCGCTGGTAGCAAAGTGACACCTTTTA
TGATAATGAAACTCAGTGTGGGTGTAAAGCTGATGGCCGTTGCATATGAAG
TGAGGTAAAAGCAAGAGGGGGCACAAATGCTGGCAGATATAgggtgggtgctgca
gggtgaggggCAGCCGCTGCCCCCAGAGGAAAGTGCTGCTGCGTGGATTGGGT
GGCTGGCCTTGAATTAATTTGGGGTGGTTGATTCAGTGATTCAGAGCGATGT
GAGATGCACCTAAGAGTAATGCTTTCATTATAAAACCTCCCGAGCAAAATTT
AACTCGTGCTAAATTAAAGCAGCCTCTGTTAAAGCGGGGCTGATGAGCACA

GCAGAGGGCTCACAGATGCAGGGTCAGAATCATTaattcctttgctttgtttcccatCCC
AGAAAAGCTGCTGCACCTCCCTGCCTGCGCTGCTGAGTTCTGCTGTTGCTT
TCGTGCCTTTTGCTCAAAGGGAAAACCCCTTTTGGGTTTATCATGGTTGAG
CACCAGGCTGTGCACGGAGGCTGGCTCTCACAAATGGCGGCGACTTCCTT
GCTTCCAACTGAACAAATTTAATTTACTCCAGCAAATATCTGGCTtagatttgttattt
taattattaacgTTACTCAGCTGGAACATCTGCTAATGCTTTCCTAGCGCTAGCCTT
GGTTTTCAGTGTCAGTAAACTTTCCCTTTATAGAAAATGGGCTTGGAAACA
CAGGAAAAGTTATCTCCCACGTTGCAAAATGCACCGCCACAAGCAAAGAC
ATTCCAGTGCCAGGGAAATGTTCATTATCTGATTTAAGTCTCAGGGCTCAAA
ACAAGAAATCTATACACACGTGCATACGTTTGGCTTAAAAACTATGGCACT
GTATATTTCCTGTTTCAATTACTGGCTAAAGCAGATGATATTGGGatgctctgagca
gtgctgggatggcagcagctAACAGGCAACTGCTTAGAGAGGCGAAGCGAGGTGGAA
TAAGAAGCATGTTAGGAACTCTGGCTGCGTTTCTGGCAACTGCTGTGAGAT
GGGAATTGCCAGCGCTCTCATTAGTAACAAGATCCTTCCAAGCACTGCTAA
GTGATTtctgaagagagagagagatgaactaTGGTGCGAAAGTGCCAGGAAGACGGGAAT
GACGTGGGGCCAAAGTCTTGTTTCTGCAGCTCACTGATTTCCATGTGCCGC
CCTGGGTGAGGGGCTGCGGCAGCTGCCGGCCCCGAGGAGGTCTGCATAGC
ACATAGCATCAACCTGTCTGTCTAGCACAGGGTTTGGAAAGCCAGCAGAG
AGGGTTTGGTTTCGCACTGCACAGGAAGGTGTCAGACCCATGGCAGGTGT
GCCTGGGGCAGGCAGGTGTTGGCAGAGGGCATctctctgcaggagctgcctggaTTTCG
GAGTGCCTTACAAGTCTGTGCTAATGGCAGCGATCTGAGCTGGCAGACTGT
TTGCCAGAGACAcctattttaattaaactcCTGCAACAGAAAGCCCCACATCAGTAATA
CTCCAAGTGTCAGCTTTAAGGCTTTGATAGCACGTTATCAGTCCCTTTGCAG
TGAACGCTTACAAAGCTATGCTTAAACACCTGGGTGTTTTTAATTCTTGTCG
ACTCATTTAAAGCACTCATTCGTCTACAATTTCTATGATTGCAACAGATTGA
GGACACTTTCAAGTCCTCAGGAGCACTCTGTTCTGTCACCTATGTGttcagagag
ggaaagggaaagggaacaAGACCAAGCACCGATGGCAGCAGAGCATTAACTGCACT
CTGCAGCACTTTCTGCATGGGTTTACCAGGAACTTTGGTTCCCAAGCAAC
ACTGAGCATTCCCTTGGCCCCAACTTTCCTATGACAAGGATAGAAAGGAGG
ACATCCATGTGAACACGtgtctgcagacagaaagatAGGCGCAGTTATATGGAACAGA
AGCGAGACAGCCTTTTGGTGACTATGACAAACATGGACATTTTCACTTAAT
GAAGAATTCAGACTGCCAGTTGTCATCAGATTCTGCGCCTCCCTGACAGCT
GAATTACGATTTCAATAATTTACAAGTGCCATTGTGAGCCTGTCCATGCCTA
ATAGGCACAGAGACGTGTTTAAAGTCAAGCTTAGAGGATAAAGTGGTGAC
CACGTTACAAGAGTGGGGATTGTCTACATCTGAGCATTAATCCGTGTGAGC
TCTCCAGAGGGAAAACTCTTTGCAAAGTAACGACAAACGTTGTGTGTCCT
GATGGGTGGCATTAAGAGATGATTGCCTACTTTCTTTTTACATAACCCCATC
AGGGTGAGGGCTCGGCTATTCTTACAAAGGCTTTTGTTCAGAGAGACAGA
AGCTCAAATCTATAATCTCAGTAGAAATATCTGTaagggagaaatgaaatagaagggaaaa
aacagcGCCCTCCAGGCTACGTTTCCTTTGCTTTGATTATTCCATTACCTCTGCA
CCTGTCCATGGCAGTGTTTGAAATTTCACCACAGTGTATGCCAATTACTTTT
TAAACCCACCACTAAACACAGACAGGAAGCCCATTTCTTGCTAAATTAAGT
GAGAAAAAATCCCACGGAACGTGTCCTGGAGAACACTGGGTGActtcaggagca
gagcagcccacaATACCCATTTGAATTCAGCACATCTATAAGAGAGAAACTGTGA

CCAACATTGCtaaaagagacaaaatgcaCATCAGAGATTCTGTGTAAAATCTATGGCT
CAAATGAATGTCTGAAAAAAGCCCGAGCACTGCTTGCCAACTTCCTTCCAC
TTCTCTCTCCTGCAGAACGAGAGCAAATCAGCACCTACTACTCCATAGATT
GCTTCTCCTGCATTCATTTACCATTGTGCCAAGCATTTGTCAAGTGGGTTTaa
gtgctttttctgtttcatacgTCTCATAAACACAAATTCCAGAAGTTATCAGGCAAAGAA
CAATCAAATCTTTAACACTTCCATTGCGTTTTGCAACATGCATCCAGAAAAC
TGCTTACGAGCAACGAAGTCTTGCTCCTACTACATACTTGCAGGCTgctgagtaa
aagaaaaagtaatactCACCCAAGTGTTGTCATGGTTACAATAGTGTACCAAAAGGA
AGCAGGAATACTGGTGAATTTGCTTGCTGAG
>TCONS_02449062
GGCTGAGCAAACACGAAGCTGTAAAGGAACTGCTTCCCATCCGGGCAGCT
TGTGTGATACATCCCATGGGGCAGCAGTCCCACTTACCCACGAGGAAGTAG
tgataataacaataataataataatgtattttaatattttaatacttaatacttattaaattattattatgtCCCATTTGTTAT
TGTTATGCTCCATTATCATTATGTTCCCTTCGGGAAGATGTCCCCCCCACGGA
TCACTACttccataataataataataataataccacCACCACAAATTCTAGTAAGTATTATC
TCTTATTTCCCACCCCCAGGTGCCTccggcagctgcagcagcccggCCAGGCTCTGTC
CGTGCCCCCACCTGCACCATGGCCCAGGGAAAGGGGTGGTTATGGGGCGG
GGTGGTGCACGGGGGACGCTCCCATCCCGGCCCCTCTCCGCTCTGTCCCCG
CGGCTCCCGGCGCGTCCCCCCCCCGGACGCTCCCGAGCGGAGGAAGAGGC
GGCCGCTGCCCCCCCCCCGCCGCGCCACAAGGTCACCCCGCTCTGCCTACA
CGTCCCGGGAGGCCGTGCCCCCCCCACAGCCCACGGGGGGGTCCGGGAG
GGGGCGGCGGACGGGCGAAGGAAGAGCTCCGGATCCATCGGTGCGTCCCG
CgctgcctcttcctctccttcctcgCAGGACGTGCGGCGTCCGGACACGGGAGATCGTTTT
AAATCCCGAACGGAAGAGATGAACCTATCTGGGGATcttctgcaattaaaaaaaaacaaa
ccaaaaccgAAGAAAAGCATCGGGAGATGAGGAGGAGGGCTCCGGTTCCGAA
GGTCGGTGctctttgcagctctgcagctcttcgTGTCGCCGTAGCACCGTCCTGTGCCCGG
AGCCAtcggtgctgctgctcccgcTCCCCCTAAGGAAGCAGAGGGACACGGAACGTT
CtccctgtgcagggctgcattGCTGAATGCAAAGAGTGCAGACGGCATTAGCAGCACC
CATGCATTGTGCTGGGATGGTTTAATCCATTCTGCATCCCCTGCAGCCTCAG
CGTGGTGGGCTACGCACAGAGCAGCGCCACGGACTGCAGCCGAGCACGTt
gccaaagcagcagagagccTCTGGGGCAGGCAGTAGATAAGGAGAGGAAGATGCAA
ACATGAAgattaagaaaagaagaaaaaaatcacaactaaaagaagcagaaataacaaaTGAGGATCTCG
GGCCTTCAATGCCACGTGGGAAATGTTACACAGAGCTTCAGTACGTTCTAA
GAGACGAAGGAATGCCAAAGACGGCACAAAGCACGAGGAGGGGATTAAA
CACCCCCTGCTCTTAAAGGAGGAAGACGTCCTGTGCCTAAAGACgaataaaaaa
aacatcattcCCTGAAACCCGAGAGcagaggATGGCAGTAAAGAAGTTCCCTGTGC
GGTCAGCgggagaaggagggagagcagagaaaCGGCCCCAAGCAGCGCTGGAGCGCA
GCGTGCTTTGGGGTGACGGTGAAACTCTTCACAAAAACgagaaaaagaatataaaaa
atcATGAGAACGTCCTGTGTGAGGCACTCGTGTGCTCCGTGGGGTCACATGG
AGGTGTCGGCGAGGCTCTGCGCGGTGCCGCGGCCGTTGCGGGATGCGCCG
TTCTCCTGCGCCCCGTTGGCGCTCGGCTTctcctgctgtgcagggctgccctggCTGCCGG
TCTCCAGGTCGTAGTGGTCTTCATC
>TCONS_02460419
gattgatctagattgatctagataactccatgacagaCTGTTAAGTCAAAATAAGCTCCATCCACC

ACAGTCTTCAAATCATTATGGAGCCTAGCATTACTGTATTGAAAGTGAAAGA
TAGGCTTCTTCTCTGACCTGATTCTGGAAATGTGGGCCTTCATCGTTATCAG
TATTGAGACTGAGTGGTAAGAGTTGATGGATTGTCCAGGTTCTAgtaaatccagaa
ggatcaccgcttcctttcctctcccaaaAAACAGTGCGCATCACTATCCCCACTGAGGACTG
TGTCTTCAACTTTTTCTCCAATCGAGAATTCTCACGTTGCTACTCCATGGAC
TCTGTCTCTTTTGACTCCGGCTCATAGTTCTGACGCCACAGCGCACCAAtggt
aatgatgtgatccaggaaactgtcaccgTGGACCCTAAATGGGTTCAGTTGTTCTTGCTAA
TTTGCATACCGTgttgtttctgttcctgtgtgtgtgttcgtGGGACCCACCGTGTGCAAACTTT
GCTATATTCCGCCGTTGATGCCATTGTTTTCAGTGCACTGAAGCCAACGTTC
ATCTCTGTATGCAGTCCCCTGGTTGTAATCCACTGATTTGAGCAGACGCGCT
GATCCAGACgctcttcattttgctgtgtgacagctgtgcattgCCATCCAGAACGTAAGTGGCTT
ATCATTTACGTCCCTGTTGccgctgctgaaatgcaatACCCACTGCATCTGCTGTTTGG
CCTTCAGCATCTGGGTGAGAAGAAATGGATGAATGTCAGGggggtgcaatttttcca
catggaaataCCAAAatccacacctttgcttcgtGTGGCCTTGATTTGGAGAAGTAGGccacaat
atatttttattgtgtttttagAAAAGGCTGATTTGAAAAAGTAGAGATACCATATACTTCA
GTagtgtatttttagaaatactgGTGTTGACTTGTTTATTATGTGATTGGTTCTTGCTTGC
AAAACTGTAATAGCTTTTAGATGTTGTGAATAGTTATGGATGTAGCATTATAT
ACTAGGAAGCATGTTGTAAGGCAGTTCTGTTTTGACAAGAGAcctcagcagaaga
aacatAATAACGAAAGGCAGGGCCTCCACACCAGGCTGAATTGTCTCACTCT
GGGATTAGGTTGAAATGCATCAGGCAGATGTCAGGATACTCTCCTCTGTCTT
CCTTATCTTCTACCACCCCAGTGTATATCATTAAAAGTCAGGCTGCCAAGCT
TATCTTTATCCAGGGACGAGCAGATGTCCAGAACTAATGGTTGAGTGCTGA
GTGAGCAAAAATTGCAGCTTTGGTACCTGTAAATTACTTCTAATAGGCATTC
TGCTACTGATCATGTATCTGTGAAGAAAGTTCATCTAGAGGACCAGGAAGG
AAGGCCACTGGCTGAAGTAAAAGGCT
>TCONS_02462923
CAACACGACAACAAAGCACttatccttttctctctgctttgtcaCTTCTGCATCTGCATTGG
GGAagaggctgctgcagaggagacTGACTCTTCTAAGCCTTGCTGTGTGATTTGGCTC
CTGAATGCCCAGGGCACCCTGCAGTTGCACACTTTCTTCCCTAGCTGTTTTA
TTAATGGAAGTTGACATCCTCATGCTGGCTCCCAGGGAGGGATCCGTCTGA
GTCATTGGCTGTAACACAGCCCTTTTCCCACAAAGGGCTCCCTTCAGCTATT
TACATCCCTCTCTTTGATCTAACTTTTGACATGAAAAGACATGTCAGCCAGG
tagagggaaacaaacaaaatgcaggAACAGTCCCACATCTCTGCTTGCACATGATCCGCT
CCCTAGCACACGCCTGCCTTACAGCCACAGATCCTTATCAGCTCTCTGATTT
CCTTGCTATCATCTCTGTCTTCCCTTCGTCCTGTTGTTTAACTTCTAGAGGCA
AATATGTGCATCTTGTTCACTGCTCAGTAAGATGCTGTGGCACAATTGCCTA
CTCTCTCTGGTATTTCCCTTCTATCTATAGTATAACTAGTATGGGAAATGCCC
TCACACATCCTCCTCACTGCATGCAGTGCCAGGAACAGGTGATACAGATCT
TCATTTCCTGCCTTAAACTGCTGCAGTATTTTAgcaagcagctgtgtgcagctcaCTCCA
TAAGTAGCAGCAATGCAACTGTAGGTGAATTCCTTTGTGGAAACCAGATCT
TTCATTGCTAACTAGCTTAGGATCTttcagagtttaaaaataaaaataaaaaaaaataagaagactAA
ATATTGTGCAAATTCAAATTAGATGCACCAGAACACAGTATCACCcacctgtgttttt
tttctataggaATGGGTGTTCTAGAATTGCTGAGAAAGCAAATCTAATGGATAAGG
ATGTTGTTGAGATGCAGCTAACTCTTTGGAGAGATGACTGTTAGTGCCAGG

TACAGAATAAtgtgggaaggggaaaaatcaAACAGAGGTTAGTTCCTACCTTTATCAG
CAGTTAATTTTCTTGTGGCGTGGGCCAGCTTGTTGGGTTTACACAGCTTGGT
GCTTTTGGGCAATGTCTGAGCTGATTGGTCTGGTTATTTTATGTCTTCCAGGT
AGTAAACCAGTGCTGGAGTGCTCTGGTAAGGGGGATGTCTGCCAGGTCCA
GAGCCTGCAGTCCCATTTAAGCTGTGTTGCTGTGGAATGTGGCTCCGTTGG
TGAGGGTTCTCTACTTTACCCAAGCAGCGATAGCTCTCTGCTTCACCTGGA
CTTGTGCTGTGATGATTTCTTGGGAAACTCTCAGCAGCCAGTGAAACAGCC
CTCCTAGTGTTCTTTCTTCCTACAGCCAGCAGGGGAGGTGGGCTGCCTTCC
TTCAGTCTTACTGTTACCACAGGATTCAGGATCCTCTCAAGCAAtgggaaaggaa
gcaaaaggGAAACAAAGGGCTTTTCCTGCCAGCTCTTGTCACAGGAAGCCTTA
TGCTTGGAAGGAACCACCCCCTTGAGTGTTGGGCTTTGAGACTGTATCAGC
ACAGAAAAGATCAGACAGAGGGAATTCCTGTTCGCACTCCCCCTTCTCTTT
TGCTATCTTCGTGTAAGGCCACAATTAAAGATTATCTGCTTCCAAATAGCGT
GGAGTTAACACCCACCAGCATCTCTCTTCATACCTTTGGTTAGACCTTgagagc
agcaggatgcaggCAGAGTGGCCATACAGAGATTGAGTAaactcctgactccacacaggactacc
TGAAAATTAAACCATATGTCTAAGCTTGTTGTCCAGACGCTTCTTGAACTCA
GACAGGCTTGGTGCTGTGACTGCTACATAAGCTTTAACACTTGTGATGAAG
TAATTGTTAAGTGTGAGCATTACCTTACCAGGCTATGGAGACACTTATGTTC
TTTTGTTAAGAACACCTCTACTCTGATCAGCATCAACTTCCTACCAGAGCTA
TGTGAGTACAGGTCCATTATATGTTCCCTCGTGAGGAACACAATGGCTAAA
CTGTGGTCCTTTCCACTCACATCCAATATTCTAGCCCAGCCTCTTATGTAAA
AACACTTCTATGTGTTTGAAAGTCACCCATTAAGAGCCATGTTTGAGAGGA
AGCAATTGCTTCAATAGAGCAATATTTACtagctttgcagaaggagcacaGTGAGACTC
ACCCCAATGAAAGTCATGTTGTGTTAATGTCTCTGGAGTGGGCTTACCTTGT
TCAGGCTTAAcctttgcttccttcctgtCCTTGCAtctgctgtgtatttttaacCTACTGCTTCAGAG
GCATCCTGCACACATTCTTCCCTAGCCGCCTCCTGCCCTTCCTGCCTCACTT
CATCTACCAGCTTGGGGGACAGGAGCATGCCTTGCAGCTGGCATCTCACAC
TGTTCACTTTCTGCACTGCCCTCC
>TCONS_02480689
ATTGCCTGTAACATTCAGGTAACATCTTAGGCTGCCCTAAGTGGGGCTCAG
ACCAGAGCTGATGCAAAGCCCATGGTCCCTCAGTGGCCAGCAACTCCTTC
ACCTTCATCTGCTTCCTCCAACTTaaaaacTGTCAAGAATTACGTTCTGAAAGT
CTCACAGCTGCATGCACCCATCTGCAGGAGTGCTTCAGGGAACTATGGCAT
CAGGAGGCAGTATGGAC
>TCONS_02507738
CCTCAGGGGCCGCCTGGCTGCCGGCGCCTCGGCCGCGCAGGGCGGGGCAC
GCCGCTGCCCTCAGGGTAGCGGTGTCGCGGTCGAGCCGTGAGGAGAGAGC
CGCCCCTTCGCCACTCGGCGAGGCTGGAGTCCGGGAGCCCTCCCTGGGCC
TCGTCTGAGCTCAGCCCCGCTGTCGGGAGGGCTCTGTAATGCCACGGGGCC
GGAGCATGGAAGAAGACGCAGGGAAGGAGCTGGCGTTTCGAACTGGGCT
TTCACCAGTTACTTTCATCAGGCCTTGTTCAGAAGATGCTCATAGGAAAATG
GCGTGTTGTTCCCTTTGGGGAGCAGGATGCGACTGCTAACAGCCCTTTCCT
CAGAAACGATCACTGTACAGCTTCCAGAGTCTTTTGAGACCATCCGCACCG
TGTCCATTACATATacaaacttagaaaaaaataaccctcTGAACAACTGTGGAACAAAAAG

GAAGCTGCTGGTTTTATAAAATAAGCTTAAGAAGGAGGAAAGCCAGCCTTG
GTGTTAAGTGATGTTCAGAAATGAACCATGGAAGGCCAGTGCTATCGCTTT
CCTTCCACTCCGAGCTGCTCAGTCCCAGCCCTTACTACAAGTCATAAAGCT
ACAGTCTGATAGCTGGCtgcaaataaaatgcttttcatgttACTTCATAAACTACACCTTAG
TCTGGCTGGTTTGAGGTTCTCCCTTGCACAGATTATATGGTCACTGACTGCA
ATGATCGCTGTACTTTACCATGTTGACACACTAAGCCTTGTAAACTTGCACA
GAAAGtcaaaattaattcagttttactTCAGCCTAGGTATCAACTTGTTCATGTATTGCAA
AATGcatcctttttttatttaatgaggGACAACACAGAGTCAGAAGTTAATGTCCAACTATT
TTCTCTAAGCTATTAATTCACTGTTTCCTACATTCTGTACCACTGATGTGTAT
TCCAGTCCTTTTGGGACCATCCAATGCTG
>TCONS_02515112
gttcaagctgggcttatgcctttctaattttttccCTCATATCCTAGCAACATCTTTGTACTTGCCCC
AAGTTACCTGTCCCTTCTTCTACAGCAGGTAGACCTTCTTTTTCACCTGGAG
CCTCAGAAAATGTTCCCTGCTCATCCATGACAGTCTTCTTCCCTGCCAGCTC
ATCATACAGCACAGGAGTATGACTGGACTGCTCCTGCATCtctaagacttccttcttgag
gagtCGCCAGCTTTCCTGGACCCCTTCACCCTTCAGTTCTGAGTCCCAAGAC
ACCATCACTACCAGTGTCCCGAACAATTAAGAGTCCATTCTacggaagtccaagGT
TCAACAGATTTTGAAATTATGATCTCTTTAAGGGATGCAGTTCATTTGTCTC
ACACAGTTGAGGTAAGAGGTATTGAGTGTTTTCCAGCCTTAGCTTATTCTGC
GGTACAATGTGTCTCAGGGAACAAGCAGTATAACCAGGCTTCACTAGAAG
AAAGCTGTTGCCACTCATGAATGTAGACAAGTGTATTATTCATTTGATACATT
AGTGCTGTGGGAATCTTTAGTCTCATTGTTGAACGTAAGTTTATTAAAAGTC
TTTGCTATACAtctaattttctgcttttaagcaGAAAACTGATACTATGAAAACTGCTATTA
TGTCATTATTACTTTATGTCATTATTATGTTGTTACATGGGGAAAGATAGATGA
TGCTAAAAAAGAAGGTGTACAGGTGTACATCCCTTAAAAGAACTTTGATTT
TTTGCAAACTTTTTATATGTTCTGGATTCTGGCTTTCAAATTTTCCTTCCAcgta
catttttaaattttttttactcagaattTCATCAAATAAAGCTC
>TCONS_02516799
CAAGAAACCGTCTCGTCAGCTCAATTTCAGaattcttcactcagaaggAATAACCTCT
GATTAATGTAGTGGTTTTTAAGCCTTATTCTCTGTTTAAAGCATTTTTGGTGT
ACTTACAACTCACACCTAAATGCCAGTGTAAGAACTTTCACTTAAGTTCTC
AGTCATGAGGGAAAATAAGAAGACTAAAATCCTGATGTGTTAAGTGGCAGC
TTCTAAGGCAAAGAAGGAAACATCACAGCACATTACGGATGTGAAGGTGG
CCCTATAAACTGTGGAGACGATGAAGGCACTTAATAGATCACCACTGATGA
GAGTTTCCCGGAGGTGGGGGTCCAACCTGCCCTTACAGCAAAGTTTATCAG
CCTCTCTACTGTCCTGCTACAGCACATAACAGTACAGATGATCTGAAGACT
GGTGGGTTTACTGCTAAGTTAAATAGCGTTGTGAAGGTGAAAACTGAGACT
TGTCTATGTGTAATAGGTAAGGTGTATCTCTAGCAACATACCTTTGATTATTT
TTTGGTGATAGCCAAAAAATTTCAAGCAACTTGTTTAGTGACCAAGCCCAG
CTAGAAGCACAGTTTCTCTATGAACTTCACTAGGCAGCAGAGAGACAACA
GAACTTGAAGAGGAAAATTTGTCCTACCTGTAATCTAAAAAGCCATGCAGT
AATCTCTACTTTTTTATGGGCTATGTCTGTAATGAGGACAAACAGGCTTATA
ACCAGAGAGCTTCTGCTAAGTGCCTGAAATCAAGGAGATGTCACACTGCA
AGCACATGTACAAATTACTGTCTGCTGTCATTtgtcagctctgtgcagctaagtgatttttcttgct

tataTTGTGCAGTCTGCTTTTTCTAATATGaatttcatattattttctccttgaCTTTCCCAAGG
TAAATAGGCCTCTTAAGTCCTTCTCGATTAGCTCTCTGTTAGGGGTCTATCA
GTTTAATAGACTGTGCATTTGATTAAAATGTTATTCCATCCTTCTTCCGGATT
GTAATGAAAAGAGCTGGCAGAACTACCCGCAGTTTAAGAGCCTAACTCTTT
CAAGGACCAGGCTCTGCTTTTAGTCACTTATGACTTTTGTCAAATTTTAACT
GATCTTACAGAGGTTTTCACAGTAGATGTCTGCCTCGGgctcattttcttcccaaatgtcat
aggaaaaaaaatatttcttagaatTTATTTGTATACAACTGTTTTGTTTATATTGAGGAGGA
ATGGAGAAAACAATAATTCAGTAAATACTTCTAATTAAAGTGAGACTTGACA
TTTCATGAGGTATTATTCTGAGTTTAGAGAAAAATTCTGCTGTCCTCACAAA
AATGAACATGGATTTGGTGGAGTTACAAACTTCTGACTGTCTGTCACTGGG
ACACGAGCAGCATACATGTGTTAGTATTTGGGTGCCAgattcttcatttattttgtctgtatg
AAAACTGGGAGCACTCTGGGCCTTTCTTTACCATCAGTCCTATTCAGGGCT
GAATGGAACAGATGAGAAGGGTGCTTCTACGAGCACAGCTGGTGAGACCA
GATGATCTGTTCCGTCAGTGATCAGGAGAAGCACATTGAAAGATCAGTATC
TAAACTACTCAACAAGACAGGGCCTGGGCATGGCCTGAGCCATGTCGATAT
GCCATAGCTCAAGCCAACTAACACTCACCCCAACATGGTCTAGGCATCATT
TTATTGGTTATCATAGTCCACGGCTACCAAAAAACAGACTATATAGAGCCAA
AATAATTCTTACATACAGGCACAGATGTACAAACATGAGAAACTGTACCTTT
GGCTCAGTAAAGATTCCCTAGTCCGATCTACTGCTTGTTTTGAAATAGCATG
GGAGCCTGATTTAGCAACTGATGAATAGAAATCTGGATCAGActatattgtttttaattt
catatctttcttcttattatatatgtattataatATTTAATTGCCATGATCCTCTCCTTGACTTCTTAG
AAATCTGCTGGCCCATTTGCATGTCTCTAATTCCTCAGTAATGTCACCATGAT
GACACCTGTCACTGTTAGTTGTAATTACTGCCAAACATAGTGAGATTAACTA
ATTCTTCTGTGCTGTTAACACTGTATGACTTTCTCCCACCAAAATGAAATCT
AGAACTTCtaggaaaaaggaagaactagaaaaaaccaaaataaatcaaTGGTGGAGAGTGAACA
GGcagccaaaaacaaaaacaataaaaagcttttaaaaaaactttccagtattttttcattcatttagtAAAGCTTCTG
AGTTTTCATCTTTGcactttttttcaaatataagaaaaccttttttttacTTACGTAtttgagattttatttcaaataa
gcTTAAGCCAAAGAGCTGACTCTTGCTCTCCATCTAAATTCTAGTCACTTTCA
TTTCTCCATGTACTGAAAGGAGTATCTAACATTTATTATCTCAGTATCTCAGA
CAACTTAGAGCAATATAAGTCCTACTGTCTATCCATGGGCCTTGTATTGCATC
AACACAAAAGCTATTTCAGGTCTAAGGCTCTGTAGAACTGACTTTGTTTTA
ATAACTGCTGCTGTACTCCTTTTAATAATATAGCTCAATTCTAAGGGCCACAT
TCACTGAGCTAATGCTaagtattttcttgcttttaaattcCAAAGCTAAGAAATACTAATGGT
AAAAGAGAAGATGGCTTCAGGCACACAAATCAAAAGCTCTCcaacaatttaaaaat
gaatttgcagCCACTAGCACATGTTTAGATTGTCTGGATTATAGACATACTTCCCC
TTAAACACTCTGCACACAAAAACAGCCCACAGATATACTCTTTGGTGAGAT
GTTATCCTTTGGACAAAGACTCAGGCTACAGCCCTGAGCACCAAGAGATTT
GACCATAGTGGTACTTGCAAGCCTACCCTCCAGGAGTGGAGGTTGATATTA
AAGGTTCTTTTAAACAACGTCATCTTCTAGCAAacatttaatgagaaaaaggaTCTGCA
CATGAAAATCTACAGCTCCATCCTTCTTTCCTGACGTTTGTCATTTCATTTGT
TATGGGTGCCTTGAGAGTTTGGGAGCTCAGAGGTTCTCTGAGGAAGCGTGT
GAGTGCAGACATCACTCTGCTTCTAGCAAGCAGCTACTGAAGATAactcctttctt
gagctttaTCCTCTTTTATCTTCTAACCTGCCTGTTCACAGGCTTAGCAATGCAG
CTGCGCAACTCAGAGCTGAGACCAACCTACAATTTTCAGGAAATAACCCA

GGTAGTGTTCCTTCACCTCAGGATGTATACCTGAGTATACTGAGTGCACTGC
TATTATCTTCCTACAGGCTTTCTGACAGCCTTAAAATTAATTATGTTCTTCTAT
GTATAATGCGGATAGTccaaaataatcctttttttctaaccaCTTCTAACTTCTACTTAAATTT
CTTCAGAAGATAAAAGGCACTAGGTGATGTCTATTACTTTCTTCCATCATAC
CTAAGCATTTTGCAAACCCAGTTTGCAAACCCAGGGACATGCTGTTGCTAC
TCAAGCATAAATCCTATTCTATAAACTTGCTTGCAGATGTTGGAATTCCATTG
TGTAATAactaaattgcattttcttatAA
>TCONS_02519218
CCTCAGTAATATGTGGCAAGGCTCTTCCTGCTGCATAACATTTTAAGCCAGA
ACTCCAAGGATTAATTTTATATTCTAGAGACTgcactttctgcatttttcccttGGTGTTTG
CTTGGCGTCTTGTGAGCTGTACCTCTCAGTTGAACCTCTCAGCTGAATACT
GGCATCCAGGAATGCAACTCACCTTCTCTTCTGCTATTAGCTCATTTTACAT
GCATAAGCAAGACTTTCTGCATCGTATTCAGAGATGTGAACCCGAAATCAT
CTCTTCTGGGACTACAAGGAGACTAAGGAGGCTAACAGTCACTCTTTGGG
GCACTTCAGACTGGCCTTTGATCACCAGAGATCAAGAGTATTTCCACCCAT
ACAAGGAGCTTCCAATGACTCAAAGAGCAATCATTGGATGATATATCAAAC
TCCAACTGCTAGATAAAGAAGAGAAATCGGAGTGCCTGCAAGGATACCAG
CTATTGTTGTATCATCATAGCTCTTctgtaacattttcttctcatgaGGTGATATGAAGTGCA
TGGAGAGGAGTAAGTTATAAAGTTGTACTAATGTGATTGTTTGGGATGTTAA
AACAAAGTCAACAATTCCTTTACAAACCATCCCAGCTTCTGGGCAGATATC
CCTGGAAGCTTCAGGAGTCCTCTTTGATCCTGGCTGCCCCATAAAGAAACA
TCGTTTATACTGAATTAATTAGCTGCCACTTAACCCTGGCGCCAAGAGTTGA
TGTCATGAGGCAAAACAAAATGGAGCTAATGAAATCAATAACATCATTAAA
ATAACTCACAGCAGAGTTACTGACTGCCGTGCCGACTGTGCTTATGGACCA
ATTGCTCAGCACGTAAAGGGACTTCTGCAAGAACATGTTCAAGCTATGaatgg
cagccagcagccaacTTGACATCACTCTGCTCAGGTGAGCTTTTCCAGGACTGCAC
AGGGAGGTTGCATGGGCACAGCCTTGGCCTGGAAGTGTCAGATTGGTTTG
GACAGATGGCAGTAAGGAGTAGAGGGCAAGGTGCTCCCAAAGCAAATTTC
TGAGTGGGAGTTTTAAGCAGGAAGGTACATTTAGGTAGAGCTTTTCTACTAT
TAATGAAGCTAGAGTAAGACATTACTAGGAACAATGCACACCATGCACAGG
AACAAGCTGACCTGGAAAGATTAATCTGTCCCTGAAGGCCTGAAGCATCTT
GGTTGGCTGCTGAGAAGCTGAAGCCCTGATCAACAGTGCTTAAAGAGCAT
GGACCACAGTGAGCTCTGGCTCAGTGAGAACTTCTTGATGCTGTGCTTGCA
GGCCTGAGCCCCCATCATTGGAAACACACTGTAATGTCAATGCTTAATTGAA
TGAAAGATCTAACTGCATGCTTCTTACACAGAATTTCCAAATGTTTGCAAA
GTCTAAGCCCCCTCACTGTGCAAAGATATAATAGATACCGTCAAAAAGTCTT
AAGACAGACAAACAGCAATGGTAAGTCTTTCACTGTTCAAGTTTTACACAA
TGTTTTCACCACTGTTATCCGGCTCTCCTGTATTTAATGACAAGTTCATAGGC
GGGGGCAGGTAATTGTGaacattaatattaatttttcctGACACAACTAAGTGCTAAATAT
GCAGAAGCATATCTATTTagctctgtgctggtgctcTTTCTGTGCTCTTCCCTGAACTG
ACTGCTGCTATCATCTCTTTAAGTTAGCTCCTTCCTGACTAGATGCTCTGCA
GTATTTGATTCTGACTTATACAGGAAACTTGGGTCTGCAGGCAAAGGTGAA
AGGCAGCTGGGGTGAGAGTGACCTTCAAGTGGCAAATCTGAGGCAGAAA
GGACATTTGAAGAGTTAACAGTGCTGTAAGGAAGTTGTGTGGAAGGCACA

ATCACAGAAGAAATAGTAAAGAACTGCTTGTGAACTGCTTGGTAGCAGGG
CAACTTAGCACAAGCCCTACAGGAGGCCCACAAGGTACAAAGTGATTAAA
TCATGAGCTCTTCATTAGTTTCAGATGTGCAGTGGAAGCAGACAGAAAGTC
AAACTAGGTCATGTTACTAAGAGgaggaatgaaatgaaaaagatataGGTGATGCAACG
AGCAGTGGGCGTGAAGTTTATGCACTTGAAGTAAAAGGAGGGCCAGAGAA
AGACCTGAGTTTCACAAGGAGGAGTTACTCAGTGACGATACCAAAGACCT
AAATGGTTGttactttcttcctttggtCTACTCAAAGGTGCCCAAGGAGTCAGAGAGCA
AGCAGCTTAATTTCAATTCCTGGAAAATGCTGGAAGTGGAAATTGGTTAAG
CTCTTTTCCAGCACCTAGATGACAGCAAGCAGATGAGGAACAGCAAGCAC
TGatttgacaaaaacaaatcacatcCAATCAATCTAATTTCCTTCTGTAGTAGGCCTTAGA
TCTTGATTTTAGTAAAGCTTTTGCCATTGTCTCACCTGACACTTTCATAAACT
AAGTAAAGAAACATGGTTTAGAAGAAACTGCTATTAGAGGAGTGTACAGCT
GGTTGGAAAACCAGACTAAAAGCAGTTATAAATGCTTCACTGTCAAACTGG
CGGTACGTATCAAATGAAGTCTGAAGCCATCTGTTTTGTGTCTGGCAGTGTA
CGgtattttcattaatatctTTGAAGAAATGAGAGTAGGGAACACTTATTAAATTAGCA
CAGGACACCATGGAGAACAAGCGCTTTGGAGAAACAGGATGAGAATGCA
AAACAATCTTGACAAATTGGAGGAATGCTGGGGAAAACAAACGCCATTGA
GCAGGgacaaagcagagctctgcagttgCGTAGGCGTCATCAAGTACTAAAAGACGGCT
GGGAAACAACCggcacagcagcagttctgcagaagaTCTAGGGATGACAGCTCAACATG
AATGAGCACGGTCATGCTGcagtaaaaaatgaaagtgcTGTAATGAGGTGTACAAACA
GAAGTTCAATCTGCaagaaacaggaaataattCTTTCCCGCTTGTCTATGCTGTGAAG
ACTTCATTTGAACTGTGCAGGTTCGGCCACTGCAGTTCAGGACAGTTTCAG
GACTGGATAAGCTTGAGGATTCAACTCAGCCTGCAGCCGAACACCTCAAA
CATGGGAGGCCATTCCACACTGAGGACAACTGAACCAAACAGAGTAATCG
TATCTTTAGTTAATGAAACAGCTTCGGTTAGAAAAAGAGGGTGATAGGAAG
GCATAAAACCAGCATGAATTGATTCAGCTTAATAAAATGAGCTGGTTTTATT
ATTTGCTTGCAGCCTACTTCGTATTATTCCCCTGGCAGAAGTGACATCTCAT
CACAAGCTTTTGGGAATCTGTTGTTAAGGGTTTCCTCTTGCAGCTTACAGA
GCAATACCATAGCTATGCAAAATTTCTACAAACTCACACTTTCTCCTCTCTT
ACCTTGATGGTAACCTCCTCTCTAATCTCCTGGTCTGTCAAATGCTTCCTGT
TGGCTGCTGTAGACATCCTTGCTTGTGCCTCGATGCATCTTTTAGCTGTGAC
AGCCACAACACAACTCCCTGCCTTCCCTCAGGTGCCTAGAAATGCAGCTCT
GTCATCTTCCTGTTCACGTTTAGCCTGCCCCCTGAAGTCCTCCAGGCTTTTA
TTATCATGCAGGTTTGTGTTCCTCTCTACTCATTAACCTCTTTTCCTCTAGCC
TTGTCTCTTCTCTTTGCCACATTAGACTTCTGCACTTCATCACATCAAGTCT
GCAGCAGATGTTTCCCTGGCTGCCCACAGACCTCGCACTCAGTGCAAGAA
AGGGAAACTCCATCTCAGTTGAAGTGAAACACAAAGAGGGAACTCTGGTA
GTCCTCAGCAATCCCATCTGAATCCTATCTCAAGTCCAGAggtatttcctttctcttttcat
gaGATTCATTCACTTCTCCTTTCTCATGACCCAGTCTCCTACTTGTACAGCAG
AACTGATGGCTCAGATGGTGCCTTCTGACCTTAAATGCCTGAAGGTTAACT
AAGAATGGGGGCTCGTGGCACAAGTAAGAGCACagcatgtttttaaagaaacGCCTTG
GATATGTTCATACACTGCCATAAAACGTAGCAGCTTGgctttgttcatttgttttaccACTT
GTTGTCTTCTTGCTAACTCTGTACTTCTCTGAAGTGAAGTTCATCAGTGCAC
TGACACAGCGACGTAGTTATGTATGCCAGGCACATAAAACACTGCCTAGCC

GTATTTTTGTTAGAAGAGATGTCACACGTGTACCACATGTTAGTGTGGCTGA
CTGTGTCTCCCTGGAGCTGGGTTTCCTCACATCAGCAGGGAACTGGGCTGG
TAGTGGGCCTGGACCAGTAGATATGCAGGGACATGGACGAGGAGCATTTTG
TAAGGTTGATCTCCCAGGGGGGATTATAATCCAATTCATTATACAGCAACTG
GGAGTCCCCTGTGCATCTCTATGTCTGTAGAGGTGGATGTAGATGCAAGTTG
CAGCTTGGAGCAGAAGCACGTGAAGGTGAGGGTAGATCTCATCAGTGTGT
GGGGATGATACTTGTCTCTAACTGCACACAAAGGACATTTCATCTTTCCTCC
CAGCTCATCCACTTCCCCTCCCATAAAGGAcataaaaagaaaccaaacaagaaGAGCAG
CCAGTAGAAACTATCACATGTTTGGGGAAAGCTCGCAGGACAAATATTTCA
AACCAGAACTGCCTGAGATGACCTTATAAGCAAAATAGACAACACCTACGT
ATTGCGATGGAAATGACAAAGCAGGGAAATTGTTGCAAACGCAGCACAAA
TGGACACTAGCTGCAGATTACACAGATTAGGAGAGCATTACAAATTGTACA
AAGGAAACgctcaatttttcttttcagtattaatAAAAGACACCTGAAATCAGCAGTACCAA
TGATTCCCTTTGTGAATCTCAGGCAATATGTTGGACAGCCACATGTTTCTCG
CTGGCATCAAGCAGCCTTGCTTTTACTGCAAGAGAAGTAAGAAATTCCACC
AACGTCAGGGGCACCACGCAGCCGTCTCTCTGTACTCATTCCTGAAATGAT
CTGAAATCTAACAGAAGGCATTTTATTGACAGCTATCGTGCCTATTAGCTTT
CATATGTTTTCAGGACTTCTCAGAAGGAGCTGGTTCAGGACCTCTTCCAGA
GGGACCTGTTCATCAGCCCACCCCAAGTGACCATTGGAGGCTGTGCAGTGA
TGCTCACTTCCAGGGCTGTAGTCCCCTGGGCATGAGGCTACTTATGGCCTA
GAGCTTGGGCAGGAAAGCCAGGACAGGCAACCAGGAGGCAGCTCATAGG
CTGGCAGCCTCCAcagctgcttgtgctgcttGGGGCCTCGTGACTGTCTGTGTGCCAT
CCTCAAGCGAGAGTCTGGAGAGTGGGGCGATGCACCCTGTGGTAATTTCTG
CAGTGTTGCAGGCCAGGAAGCACCAGCAGAAGAGATGTTATGACAGTGCA
AACAGTTACCTCCGCTGCAGGCATGATAGGAAGTGacaatgcaaacagaaatgtaaCC
TGTCAAGGCCACAGCTACATGCTTGGGTAGACGAGCTTGTCGTTTAACCAA
GAGCAGTGCCACAGCTCCCTGCTTCCCAGTGCTTGAAGGCATCTGCACAC
AAGAAAGCCACCCCAAAGCTCCGACCTTTCCATATGAGTAAGGCTAAAGG
ACAATGCTTCTGCTTCCTGGCATCagtctgtgtgtgtgcgtgtcAGCACTAATTACAGCC
AGCAGGATGGTGATTTTCAGATGTAAAAGCTTTCAATGCCAGCACGGgccttgc
agtgctgcagtggaaaaTATTAAGAGAGGATTACCAGAAGGCCCAGAAGAAATAATA
TCACAGCGAAAGGAAATTATACTGAGGAGCACAATGTTTGACTTACAACTG
TTGTGGTCTGAAACATAAATCCACACGGTACCGAGTGTCTGGAAAACTGAA
CACCAACTGTAATGAAAGTAATCATTTTGCTAAAGTATTCTTTACAAAGGGC
AACACTTAAAGACATGTGTTGATTAAGGTAAAAATCTATGGATGCTGGCCC
AGCTCTGGAAGGGCTTCTAAGATGAGTGCTAGCTCTGTGCACACGAATGTG
GTGTTTGAGGTGGCAAGCCCTGCCAGGGTGAAATCCCATGTACCTCTGCCTt
tggagagaagaggaaaatgtgcCTTGCTTCTGCTGTGAGACTGACGGATAAGGAAGTG
CTAAGCTTCATCCTCCcttctgcatgctgctggggcatgcagatgacagcagaacCAGCCAGAAG
GTGCAAATATCTGAAGCAGAAGGCGGTGGATTAGAGATGGTTTTGGAGGCC
ACTGGGGAGACaagaagtggaaaatgaagaaaacagaaaccaagtctgaagaaataacaaattccagaaaatg
agagaaaaaatcaGGGGATGAAAAGaagatggaggaaggaaggaaagagataTCTAGAAAAAG
AAGCATGGAAAGGGTCAAGTCggaagcagctgaaagcagaaacttcaaagaaaaaggcaatttaTA
TTCAGACTCTTTGGAAACCCTTGTTATCTATGACCAACAGGGTCCTGGTACT

TACTAGGTATAGACATAAAAGGGTTGTAGAAGAGTAAGACAATGGTTTAAA
CATGTACAAgccagaggaaaggaaaactcagctcctgaaaggaagggaaaagaaagcaagggaGGGTCA
TTTTAAAATTGGCTGAATTATCTTTTAATGTTTGGTAAAGCTACACTCTACAG
ATGCTAGAAATTTAAGAGGCACAAAAACTGGGATTAGTATAAAATCCTGTG
GACATGGAGAGCATTTTGAAGGCAGGAGATATAGCTTGTGAGACAGGCAC
CCCATGTCCCAGGTGGTGACTGCAACCACCTCTTCTGAAGCCAGGCCTGCT
CAAGTATGGCCAGCAGAGCACCCACGAGGAGCATGCATCTGGCAGGCGGC
CATAAAAAACAGAGGACATCCTACCAGGGCTGACCAGTACTCATACAGAAT
CAAAGCTGGGATCCAGCCCCTGCCAATGCCAAGTCTGAACAGGCCAGCTG
TGGCCTGGCAATAGCAGGGCTGCTCCAACCATGGCAGTCTGTGAACAGAA
GTCTGCCACAGACATGAGAACCAAGCACCTTGTCCCATCATTACAAGGGCA
GCATCCCCAGGTGGCCAGGGATGGTGGAGGAGGACCTTTGAGCTGGGGTG
ACACTGAGCTCTTTTGTACTGCATTCACCCCATCAGCACTGTGAGAGCAGC
TGGTGTGGTTGGGTCCACTTGAAGGCAACCTCCAAATCAAATGGAGGCATA
AAGACAACACTGATGACCATGAAGTCAAACACCAGACCAAAATACTCGAG
TGGTTCAAATGCACTGAAGCCATACAATGGCTGAGCGATTGAAATAGAAAA
GGAGTATGTATGAGTGTGACCAAAGAAggtaaaaaaattattttgagtgCTGTTTGTTGC
AGACAGAGGAAACTCTCTCTGTTAGGCAACTGAGCTAGTTAACAACACATA
CAACATTACATGTACCCTAGGAAGGAGTGTGGTCTGGGCATTGCCACTGTT
AAAGAAAGAGCCCTCTCATGTATTTCCCAGGAGCCTGAACGGTTACTTCAT
GTGTGAGAACGTCAGGCTGTGACCTACAGGAAAAGAGAGGGCATGAGGG
AGCCAGATGGCTGAGTGACCCCAGTGCTGTTCCCAGAAGAAGCAGGAGCA
GGCAAGGAAAAATCTGGTGGCTGGAAGGAAAAGTGAGCCCTAGGCAGGT
CCCTCCATGCTCTGGGTCTCTACTGTTCCCTCTCCCACCAGCCCCCTTTTCT
CCTCTGAGCCAGCTTCTCATCATACCAGACTGCCCAACTCCCACTCCCCCA
CCATCTCATCCACCACAGCTGCAATTTTCCAACTCCGGTAAGAAACAGAAG
TATCAGCATAGCATATGCATGTGCTTGCTGAGGGACGGGAGCAGAAACCTT
CCCACCTCTTAGTAGTgtctccagctgctgccttgctCTGCCTTCCCAATCTGCCACATT
CTTTATGTCCCACGTGGCTGCTTTGGCACAGCTCAAGTGagtgtggggagcagcagtg
gcCTGTGGTGGCCATGGTGACTCATACTGGCTCTCCAGCCAAGACAGACAG
GGGATACCCCATAAGCAGATGCTGGTGACCCCAGCATGTGAGTGCTACTCT
GGCCACCACCACTGCCATGAAGCCTCTATGTCTGTCTTACACACAGGTACT
GTACATGACTGATGTAATGCACAGGGTCTCATGCAGTGATGAGTTTTCCCAC
ACAGGCACTCCCCTCCATCTCTCTACAGAGCAGGAGGCCAGTTTCTAATGT
CTGCCCTTCACTTCAAATCCTTGGTTTATGAAACTGGAGGAAAACCCTGAG
ACAGCAGGTTGAGACTGTCACCAGGGAGAGAGTTAACTCATTTCCACGGC
TGGTAGTTCTCCCCACCAGCCCACGCAGCATCAGAGCAGCTCCGTAAGCCT
CAGCATAGACTCTTGGCCTGTTCTACAGGTGGTGGAAACTTGTAGTATATGG
TGTAAAATGCCTACAGGAAGGTGTTTGTGACAGGGCCAATGGCTAGTGCAT
ATACCTGCCCCAAGGCAGGTTCTGTTGTTGTTCCACCACTGCAGAAATGAT
GTAACACTGGTTTTATTACAGTGCTGGAATCCGGCAGTGTTTTGCACCTGG
AAGGCTGCATCTGACACCCACCTGTGAGGCATCAGTCCATGAAGACTGGG
GCTCTGCTTTGGTTTTGCGGTCACTGCTGCTAATGCGAGAAAACGGACAGG
GCAAAAAACAGGAGATAAAAGGCAGtcaaagcactgctgaaaaaataCCAGATAGTTG

TAACTCTTAGGACTAACCTTCGGTGTCAGAGGAGTCAATGCCTCTATTTTGA
AAGGATGAGCCATTTTATGGGTATAACAGCAATGCACTTCATAATCCCTTCC
ATATTTGGCTTTATTGGCTTATTGTGCAGAGCTATTAGAATCAGGATCATCTC
TTATGAAGCTGTTAATGGGATGCAAATTGCATGAAAGATTTCTCCGCATCAA
TAGAAAACAACCTAGTAATAAGTTATTGcataaaaatctgcataaaaATCTGCTACAAA
AGAAATACTTctaaagaagcaaaaaatcCTGCGTGACTGAGGAGGAAATATGCTTTAC
AAAGTGCAAATGAGTACTGGTTTTAGAGGTAAAGGTGACCTacctcagagctgctca
gatCCCAAAGAACAGCTGTGCCCTAGCTACCCAGGTATAGTGAAACAGTGCT
TCACACAACATCATGGCTACAGCCAGGTAAAAACAGTGTGATGAATGTTTT
AAGCACTGATAGATGCAAGTTCAGGCTGACCAAACCTGCATCTGTGCTGAA
TTCAGCAAGGAGCTGTGGCCAAAATATGTACatttgaataaaatgaaaagtctAAGCAG
CTCTCTGTATGTGATGCACTCCTACTTTTCATGCTGACACGATGTTTTGAGA
CTAGAATTGGACTTACAGTGAGCTAGAAACACCTAAAACCAGCATTAAGTG
CTGATCAGTAAGTCAAGAGAAATATTGTGCTGAGTCTGTCACATcgctttgctgtttg
tttttccttttttggtgtTGACATCAATGTGACTGTGATTTCAGCTGTGGTTTCCAGAGAG
CACTGCAGCGTGCCTGCGGCTGGAAGGAGCCTTTTGCTGCCATTCAGCCCA
TGCCACCTAGCACAACCACGGGGCACTACTGCTTCTCCCACGTGGCGTACA
CCCCGCTCCCTCGAGCATCAGAGCCTCCAGCACATCACACACCATCACTCT
CCCACATTATGCCACAGAAATGCTCTTTGGTCTCCT
>TCONS_02542185
GGAGCAGTGAGGTACTAGAAGTTGTCTAGGTCGTCTGGAGGGAGATGTGA
AGACACTGGGATGCCAGTGGCAGCTGCCTGGGGGCAACCATCTCAACAGG
CTCACCCATGTTCCCCAACTCAGCCCAGGACCTGGAGGACAAGGAAGAGA
CCATGCTTCTGCCTGGATAACATTGGACACCATTGGATCTCAGGACAGGTTT
GACAACAGTGGGATCTCCACCTTTATCCTAGGGTACCCATCACACACTCTAT
TGGTCAGACAGAGATATGGGGTTACATCCAGAAGATAGGATGTGGTGAGCA
GGGTGGCGTGTGGTTCCCAGCCTATGGTAGCACCCATCAGTGAGGACAGC
AGAGAATGGACCTGCTGAGGTCACACAGGCTTGTTGCCACAAAGAAGCTG
CTGTTGCTACTTTGCATCCCCATTAGGCACGGGGCCATTTCCATCATGAGCC
CGCTGGTCCCTCTTTATGTAGGTGCAATGGAGCAGGG
>TCONS_02543135
CCTCCCTTAGCGACGGCGGCGCGCGCTCAGAGTGGCGGGTGGCGGTTGGC
GGGGCGCGAGCTGTGAGGCGGCCAGggaggtggtggtaCACAGCTGGATACGCG
TCCAACAGCACACAAGGGCAGGCGATCCCCGGCACGAGATTGTGGTGAGT
CAGAAATAGATAGAGGCACTTCTTAACACACagtctttcattattttccttgcaAGGAGCA
CTACTTGCAAAGTAGCTATTTCATGCTAGTGCCCTATAATGAGTTGGCTGATT
TATTAGTCTTGTACTTTGCTGCCTTGGTTGATTGTTTTTGATGGAGAACATAT
TGCCTTTAGGCCCTATATGATGCAAGACCTAAATAAGTGCAGCAGTACTGTG
TGGGGACAGTCTCCGAGGTAACGTGGATGAATGCAGTTTGTATGGCACATA
GCAGAGAGGTTTTACCCACCATGTTAGCCATGAGATAATCTGTAGTAAATTA
TCTCCTAACCAGGACTATTCCAAAGAACATTTGTTACTGAAGAGCATGtatgttt
tcctcttgttttacTCTTTTTGTAAATGTTAACCTCAATCAGTCCTACCAGATACATAC
CTCATCGTGATGGGTAAAGCCTCCATTACTGACGTGATCACTTTACAGCTCC
AAAGCAGTCTAATGACACGTAAAGAGATTTCAGTGCTGAACAGCTGCTTCA

AAGCATTTGAAGTATAAAGACTGAGCAGTTTTGTTTGGTACTAGAAGGTGC
CAGACTGACAGAGCTACATGGCCCCTGTACAGAACAAGTGGAGTAATATAG
CTGTCATCCTCCATAGAACTTTCTGGCTTAGCGTTGTGCTGGCATTTAACTG
TCAGTGTGAAAGCACAAGGGCATATGGACATCCCGCAACTGGCCTCTGGA
GGGAGGCTGCTCTCTGCAAGGGCTCTCGTGCTTCTTGGCACTGGGATTATgtt
gacagcagcagagctgggcttgctGATAGCCTGACTTCGGCAGGAAGGCGTGCCTGGGA
GCCGCGTGGTTTTGGCTTAAGCCTTTTACTGCTCAAGTTATTATTACTGTCTC
TCATCTCTGGAGATTGTGACACTACTGCAAAAGATAATTTCAGCATAGTGCA
TTATGTCCAAAACTCATTCAGATTTGTCAGCATGACACAAACGTGCACATTT
GGTTAGTTCGGAAGGTTGTCTGGACTGATAGAGCCAGCTGAGAATTGCACT
GAAGGAGCATGCAGTCACACTAGGGCAGCAAACATGGATGTTAATATTGCT
AAGTTGCAGTAAAACTGCACTGCTCAAGCTAGGCAACAAGTCTAATTCTGG
AAAGATCAGTGATTTTATGGAGAGATGGgctcccttctctttcctttccttgatGACATTGTG
TGAAGCTATTACAACTACTCAGATAAATGCACAACTCACTAAACTTTTGATT
TAGcttatttttgtctcttctttaTGCACTTTCTATAATCACCTACTGTCAAAAATAATGCTT
GTTCATTCTGCTGATTAATATGTGCTCTTAGAAATAAGCCCTTGCATAGAAG
AGAAAGACCATGAAATAACTGATGGAAGTGCTTAGTAAGAAAGCTCTGCC
GTAACTGGGTAAGCAGGAGGCATTCCTCACCCAACAGTGGCAAAGTAACA
AAAGGGACAGTGGAGCAGCCTGGAAGTTGCCCATTTCCCAGTGTGTGTTC
CCCAGGTACTGCATGGACCAGTAGGAGGGCTGGGTTTCTACAGTCAGGTG
AGATTTCCAGTGACAGTTAGGTAGGTAGGAATTTGAGTGGACTACTCTGGA
CACCCTCAGAGTTTGTCACAAACTTAGTTGGCAGTATTTGTTCACTAAAAG
CCTCTGTTACTAATCCTGAAGGTTCTACACCCCATAAGTCCTGCAAAAAAA
GGAGTGCTGCACCCCTTTCATCACTGTTTCTTGTGAAGGTCTTTTTTCTATT
AAGCCTTGGGTCTTTAAAGTCATTCCCTATGATTTTTTTAGGTCTAATGCTAT
GCTGTGGAGCCCATGTAATGCCATGAGAGAATTCCAGATGACTTTCAGTGG
GAGTGTGTCAGTCCTGTTAATAAGTGAAGCTCTGATTCTCCCTTGATTATTT
CTTTATCTTCAGATCATAATATTAAAAAGCTGGGGGAGACGAAATATAATCT
TTTTGTAGCTCTTTTTCAGTTAGCTTTCACACTGACAGGTTAGGCAGCAGTG
AAAATATAAACTGCCTTCACTTGCCCCATCTGTTATCCAGCCTGTCACTGTA
CCCTGTGTGAGGTACGTGGTGCAAGTTCTTTGTTTgcaagacaaagaaataaatgagtgt
TGCTCATCTGATGTTACATGAAGCTAATGAAATATTGATCACATGCTTCACC
ACATTCACagcaaataaaaaggaatttgcATTTGGAAATACTTAAATGTTGTCTGTAACT
GGGAGACAACTCTTCTGAAGGTGCTCTCAGCAACACTGAGGTATTGCTGC
AACTTTTAGGAAGGCCAATAGAAGGTGAGCattaaatggaaagcaaaattaTCTCTGAG
GCAGAGACTGGAGAGTTGCGTGAGTTCTGAGCCTGATGGAGAAACATCTG
GATTTCAAAGCTGCTTGTTGAGTTCAGTGCTGCTTACTGTAAGGACGAGTA
ATCTGCACAAAGAATGTTTAAGAGCATGAAACCCCTGTTCCAAAGCAGTGT
GAGGCTGCTGAAATCCCTAGGGTCATGCAGGGGGAATTCCTATACAAGAAA
TCAATTGGTTCAGTTGGAATAAACCTCGTTTCTGtttcctggcctggccttgctcaGTTGT
GGTTAAATGAGGAGAGCAGATGATAGCCATCTGGTAATCTTGCTGTTGACA
GGCCCGGGTGGGAGTCCTGGCTTGTCCCCTTAACCAGTGCCACCTGCAGG
GATGAGTTGAGCATTTGGAAATAGAAATGGAATGTTCCACAGTTTAGAAAA
GGTACAGCAAGATGGCTATTCTACCACTGAGTGCTACTGGGAGAGGTCCTG

CAGCAGTCAGTCAGTTTGCTCATCTCTCCCATGCTCTTGACACAGACCAAC
AAATTACCCAccatgaaagagagagaatttttcagtaattaacTAAATCCATCAGGAAAAACTT
TCCAGGTACAGCTTGGAATTTCCGGTTCAGACCAAAATCTGACTATTACAC
CACAACCTTTGGTTTGATATGGAAGACCTGGCACAGGGGAGGCCAGTGCTT
CATGCTGCTGCCCTGTATCTACCAGTGTCACCATAGCTGGTCCCAGTAATTC
TGTGATCTTTGCTCCTGTAGATAGATAGAGGATTCTGAGCCACAGCACAAC
CAAGTCATGTTTAAAACTCTGAATGTCTAAATCAGAAATCTTTTCCACAGCA
GGTATTAGGCAACTACTAATGAAGCCAGTGTGCTCAATTACTGCCATAGATG
TAGGTTACTAAGGTTTTCCTGTTCATGCTAAtacatacttattttttcaatcaGACCTTAAT
TCCTATAATAGTAGCAAAAACAGAGCCCACACTTAATGTGTTCTTTTAAGCT
AAAGGTTGCTACTACATACTGATAGCTATCACCTTGGTCTCAGCTCTCTGAC
TACATTTTAGGTTAATGCACTGATTAATCCAGACATTAGATAAATAGAGTTTA
TTTAGAATTTAATATTCAGTTGTAAATGAAATGTGCGACTTATTTTTCAATGT
GCATACACATGTGCTTTGTTACACATGTATCCATAACAGCAGAAGAAGTACT
GAGCTGTAGTTACCCCATCTACTGTTTACATTGGTACGAACACAACTTTATT
ACTCATGTGTAGCTCTTCCTAACTTTCCGAAGCCACTTATCTCCATTCAAAG
TATTTCAAAGTCTGTTAATCATGCCTAACTTTTCCATTAACCTCTTCCCATCA
CCTCTAATAAATTACTATTCAGGTTCCTACAAATAAGGTATTTTCACCTCTGT
CATAATGATTTCAGATATGCAGTTGCCCTTTAGGATATGCTCCATTTTGTCTC
TAACACCGTAATTTGGTACTTCAttaagaaaattacattttctagGCAATACCTGGAGCAG
CATAAGTGGCCCTGTCTTTTTCCATGAGAGAAATACAGTTGCTGGACAAAT
GTGTGATGTATTTCTGGGTGAACAAGTGACAGTTCTGAGCAGGGAGACCCT
GCAAAGTTGCTGTCAGTTACCTGATGTCATCACACAGGTTCTGGGCGCGCT
GCATGGAGCCCTCGCTTTGACACTTTAACAAACTGCCTGAGTCGCTGAACA
GAATCAAAGTCATCTATGCAAGTTTCACTTTACTCAGTGCATAAGACAAAA
CAGAATCCATCACAACAGTTGGGAAtaaagcacacacaaacaccAGTGCTTTTTCCTT
AAGCTTTGCTACCATGTAACGTGCTTTGGGCGCAGGAGACATGAGAGCATC
TACCATAGCATCTACAACCTCACTGCCGTTCGTGCTTCCTTTGCTGAGAATG
TCGTTGAAAAAGTCTGCCCGCTCCTGAACGTACTCCTTATTGTAAACCGCC
TTCTCCTCTTCACTGAGTTCATTCCAGATCTCCTCGGCACTGACTGGTGGCT
GAATTTTTGTAGATCGGGCATAATTTCCTGGCTGAATAATACAGA
>TCONS_02553763
GAATACTTTCCCCACAGGGAGTTGTCACTATGATGGCATCATAGGAAACAG
CCACGACTTTCTgtagggcagagctgtgcagccttCAGAGATATTCTGAGATGCTCTTGG
CCTGAGACGGAACTTGTTGTGGGGAATCACAGAAGGAAGGagtttcagagaatcata
gaatcactgaatcatagaaccatagaattgttaaggttggaaaagacctttaagatcaacTTGTTCAATCTGTGA
ATGTCAGAAGGCAGATGGGGGTAGATGCCACAGTACATTTGCTGCAGTACC
TAACTCAGAAACCTCTTTGGTGCTTGTTTGCTGAAAGCTGGAAGAACATTT
GAGTGTAAGGATGACTCTGTTCTGGTCTATATCTGATATGCTCTCCGTAAGTA
TTTCCTACGTGGCAGAAAATAATTACCAGAGTAAGTGAGCTTCCAGCCAGA
TATGAGGAACCTAATAAATTGTGATGACTGTAGGAAGTAGCAGGGGATGGC
AAGAAGGTAACCAGTCCCCCTCTGACTCGCAAGGCAGATCAGCTTGTTTCT
GTACCACTTTCCCAGGGACCCTCGATACCTCTCTTTCATGAAAGAGGACAG
CTCCCCAAATAACCCCCTCAACATCTCACACCCATCCCTCGGCAGGAATTA

GCCTCCTTTCTTTACACAGTAAAAATTGATTCAGGTCTGTGTTATCTTGCATC
TTGAAAACAGCCTCCTTTCCTTTGCCTTTGGCCACATCTGCCTTAATGCAGC
TGGACCATCTAGGTCAATTATCCCTAGTGGGATCTCCATTGTGATTTCTTTTG
ACCATCATTTTACTGCAGTATTGAACACAACCTTGTCTTTGTCCTAAAGGCC
CATCGTGGCTTAGTCACCTACTCATTTTTCCTAGCAAACCTGTCTTCTATTAT
CACCTTCATGCACTTCCTTGATTGGATAGTCATTAACTTCCTCTACGGGTAG
AGAGGTTTTCTCAAAACTCATATCAACTGCAAGATGAAATGGGATATATTTG
AACAACAATTATCCCAGTCTTTCCTTAAAATGTCTTATATAATCCTGTAGAAC
TTGTCATTGCCACTGATGCTTCTGTGTCATGCTGTCTGCTGAGAGCCCtgttaaa
tatttactgttattCTTGCAGGTGAACATCTCTCCTGATACCTCCTGCATTTCACTCAC
CTATTAGTCTTGCCTCCTTACAGTAGAACATCTCCCCCTTCCAACAGGTCCA
CAAGCAACAGAAACGTCAGCGTCTCATGCTGTCAAAGGTTGCACAACGGT
AAAAGTCATGCCACTGTACACTGTTTTAAACCACATGCTTTTTACAGTGTAT
CTACACAGATCTTTGAACAAGAGTGGGTCATCTAGGTATCCCTGCTATCCAG
GCAACAGCATACATGCAGCACCAGAAGAAGCCAACTCCAAGGCTGTATGG
GAAAGGAATGGGTAGTATAAGGGCCTCAATCCAGCAggttctgcttttctctctctcagttc
TGCTAGTTGCCCTGTAAGTGATGCTAAGGGCTATCCTTTCTCATCCAAGAAC
AGCTTACAGCCATTTGGGAAAAACTTCTATCAGCAGCTCTATCCTCTGTCTG
GTTTTCCTAAAGAAGATACAGACGCCTTGGACTGGATTAtcacagcagtgcaggtggg
CATGTTTTTCTGCCTAAACTTTACTGGCCCATTGAAAGCTGCTCTGTGATGA
ACCTATACTGTTGGTGTGAAAAGCAAGGACTGCTCACACGGTGTGTCTCTG
GGAACGTAGAAACCAGCAAGAATTGTGGCTCCACTGCTCTGGTTccctgtggag
ctgctggagaggctCACTGCTCAGATCTGGTTGTTGTTGGCCATTGAGTGCCCCTAC
GTGGGAAAGCGGTGCTTCGATATTGTGGAAACTGCCTGTGGGAAGGTGTC
GATTCTCACAGCATTTCGGAGAAGGCACAAGGCTTTCCAGCTTATTGAAAC
GTTTGTTTTGACTTTTATAATATAGTACAAAAGCCTTCAAGTTTTGTCTATTA
AATTGCTGTGCCAAAATATTATGACGGGTCACGGAATAATAGTCTTACTGTT
TTGACTCTAAAGCGTGCTACGACATGGGTCAAGCTGatgaaatcaaaatgaaattactgttt
tgttttgacaagattgtgtttcttttccatttgtttgatAAAGTCAAAGCAAAGATCTCCGTTGGATTT
TTCCGCAGTATGGCCTCAAcgtttcattttctcattcaggTGTGGGTTTTGAATGGAACAT
AATGTTTTGTGCCCAGATCATAGAGGGAAAGATGCATGTGGGTAGCACCTC
AGAGTCACAGTTCAGCCCCGATTTCTGAGGACTGTACCCATTGCTTCGTC
AGCTTTAGCAAAGAGATCTTTGTGAAAGTTCAATCTTGGCCTGCGGTGCTA
GTGTCATACTTTGAGCTATGCCACCTTCTGCTGTTGTAGTTCCCAGTCACCC
TCCCCTTCTGTTTAGCCACCTGGAACCCACTCCAGAAGCTGGCTGAGAAGA
AGTAGCTCCTGAAAACTCATCCTTTGTGACCTGCTGAGTCAGGAAGCCTCT
AAAATATAGAATAAAACTGTCCTTAGACCATAGAGCCCAATGGTACAGATGT
GCGGCAGCCAGTCTTGTCCATCTCTCATGCATGTGGCATCCCTGAATCTATT
CCCAAACCTTGCCGCTCCCCTGTGATGTTAGGAGAGTGCCCGTTCCTGCTT
TCCCCTCCCAAGCTCTGTCTGGTTGGACTGGGGTGTTTCCCGGGCTGAAAC
TGTGGCTTGCCTATATTGTGTGCAAAGTACCTGGAGCATGATGGAGATCTCA
CACTATGCTTCTGGTGACAGGTAATGTATCCTTTCTATTGTGCGCTTTTCTAC
TCTGAACATTGCCTTTCATGGTAATATACTTATATCCTTTGTTtgttctgtctctttcttttttt
tttttccccacggATTTCCCTTTGTTTGCCTGGTTTTGCTCTGATGCGCAGGTATTTA

TCTAAGTGGGGTCTGCTGTGTTTGTACCAGCCCAGGTGGCATGTAAGATGC
AAACATCAGACACCTCTTTACACATCTCCAGGTTTCCTGGTTGATTGATTGA
TTAATAGCTTTCTCCCTTATCAGCTTGTCTTTCTCATAACTGGGGGTCAGCCT
GCATGGACTAACACCTTTGGTGAGGCCCTGTTTCCTATATACATCAGCTTCA
CTGACTTCTTATTATCTCAGCCCTATCTGAACCTTTGTTCAATTCAAGAGGTA
ATTAAACTTGAAGCCAAAGCAAATGTCAGGGTGATAATGAGACAGATAAgat
taaaaattctttcttttttttttttttttaaagtacctGACACATACCTGAAGTGCCCGTTGGCCCGCA
GCCAGTGGAAAGTTCCCTTTCTCACCTGGATGCAGTCAGAGCGGTGGCCAT
GTAAAAAATGGCACCACCTCCCGCCAGGTGTGAGCGGCCGAGAACAGCAC
CTACCTGGCAAAGCATTCGAGCGTGTGGAGGTGACGGGGCATGGAAGTGG
GAGTGGGGCTTCAAAATGTGACAGagctcttctctctgttctgaAGCAAAATCAGCCAC
ATCGTTCCTGACAGAAGTATCTATTAATTGGTCCTGATGACCTGCTGGGATG
GATATTCCATGTCTTATTCAAGCAACGTATTCATATACTTGCCTCTGGAagacatt
tttcaaatgcttaGATTGCTTTTCTTGTAGCGATGGAACCATCAGCTTTGCTCTGTAT
GCACAATTAAAAGAGCAGATCCCCTTTAATTTTGCAACCATCACATACACTT
TGAAAGTgttactgcttttccttcagtctATTTTCTGTAAGTAATCCTAAttctttagttttctttcatAG
ACTTCATTTTCTACATAGATGGCAAATCTTATTCTTCACCTTTTTGGAAGTGT
GTGCCTTAAGcttaagaaataatatttcattgAAGGCCCTCCCAGAACAGCGTAAATATTA
CATTTAATAGTGTGCAAGCTTGAATTCCTGTTTGCACACTTCCATGTAATATT
TGCCTTTTCCACACCAGCATGACTTTGCAGTTTCAGGTTCAGCTAACCCAC
CAATCGGTTTTACAGATAATCCTTTCTAACtaaaatttccttctctgtttttagCATCCTACAA
TTTACCTATACAATCTGCAGAGCAATTCATTTACTACCTGGATTTTATCTCTT
GATCTTGCTTTGTACACTTTTTTAAGGCTATTTCTTCAACTTTTTTACCATCA
TTCTGGAGTCAGATGTTTTACTAAACTGTATGGTAACACAACAGCTTTTCCT
CTACGTTGCAAAGTCTTTCTTTTCTACCTGCTCCTAAGGCTGTTCCTTCCCTA
TGCTAGCCTTAAATTTACTGAGATCTGCCTTTCTGAGGCCCAATGGGTTTggc
ctttctcctcttttgccTTGACTTCCCAAGAATCAATAGATCTACCTTTTCATTGTAACT
CCCACGTTGCCTTCCACCTTCTTATTCTCAGACTACTTCTCTCACAGTGCTC
CCTCCCTTGAGTGCTGATACCCAAATGGAAAGGAAGTTTTCCTTGACTTAC
TTTCCTTTACTATCCTTTCCTACTCCAGGATGCTGGAGTGATGGTAtgctgctgaaa
tgttttcagcaaaGTTTCTTAATTTAGCTAAAATCTTCTGTTCCCAACAAGACTTTTA
ATAGACTCCAACCAACTCTGGCAGTGGCATCCTCTTTGCAACTCTGTGAAC
TTTCAGCAAGGTCCACTCCATCTGTCTTCTGGATTTCAGAGGATGTTTATGC
AGGAAGTTACATCTCTTTTCtccagagatctcttccaaaccctgtgattctgtgattcttcagtGATGCT
GTGACAAATAGCCTCAGGTTTCATAGTTTCCAAGTCTCCCTCTAGGTTACTG
TGGTGTATGTGTGAGCACGTAAGGTTTTTACTCTCTGCACATCTTCCTCATC
CTGCTGTGTCATCTGATAGGTAAGAATGCTTGTTTGTGAGGTTCATACTTCT
GTGTgtgagagtgtgtgtgtgtgtgtgtgtgtaccaGTGCATGGGACACTATTCTGAAGTCTT
TCAACCCACCACTTGCTCAGAGAAATCAGTAGTGACTCTTTCTCCAAACTA
AAAGTAGCACAAATGAGGTTTTAATAATGGGGATCAGTTGAGGACGCAGA
GACACCATTAGAAGCAAATGCAGATGAAGGAGCAGACTGCCTGTCTGACT
AAGCAGAAGTTTATCTATTTCTTCTAGGTTAGGAAGGCATACAGCTATTTCA
GATCTTtaactgggaaaagaaatataGCATGCCATGCCAGTAAGAATGCTCACTTCATC
TACATGCAAGAGTTTTTCCTAACCTTGGTGACCACACAGCCCAGATCCAAA

GTTCTCAGCAGTATCACGAATGGAGGGAATTGTCAGTCAAGGAAATGTTTC
AACACATTCTGCTTTGCCCAGAAATGTAAGAGACCATCTGGAATTAGTGTT
GAATATTTTTGCTAATGACCTCATGATAGAATATTGAGAATGTTTATTAAATTT
ACAGATGACACAAGGCAGGAATGACTTGTGAGTTTGACAGAGGACAGGAT
TAGAATTCAAAATGATCCTGCCAAACTGGATGAatgctctggaaaacaaaacaacagaacg
CAGTTCAACAGGGACACACGCGAGGCATACACTTAGGCAGGAATAATCAG
CAGCAGAAGCCCATAATGAGGAAGAATTTGTAGAAAATTCGCAgaaaattaaagg
agaaaactCAGGTGGATCACCGAAGGAGTTGTCAAATGAGCCAACTGTGTCAT
ACTTCTTTGAAAATGTCAACTATTATCCTGGGGTATAGTTGGAAATAGACGT
TGGACAACACCTTCTACTCAGACCTAGGAAGGCCTCAGGGGGAACACAGT
TTTGGGCGCTGCACCTTCTGAAAAGATGTGGAGGTGTTCGGAAAAAGGAA
ATGACCTATTATCTTCATCTGCACATTTAATCACATGAATACAGCGGGATAAA
TGCACCTTAGGTATACTTAGGAAAACCTTCCCAATGGTTAAATAGAGAGGC
ACAGTGGAAAAGATTTTCTGGGAAAGAGGTCTCCATCCTGGAAAACCTTTA
AGGACAAATTAGACGTGTACCTTCATTTAGGGTAATTTTCAGACACAGACA
GATTTGGGCAGCCTCTCTGAAAGGTCTGGCTAGCTGAATGCACAACTCTGG
TGTACGAACCACAGAGATTAAgccaatatttaaaatataaaaacttaGAGGAGCTACCCAC
CTGCATATTGACTTGAATCTATACAACTTCTGAGCATGCCCCTTCCTTAAAAT
GCCCAGAAGCTGTTCCTACATGCTCAGAACGGGTATTCCCACGGCACCAGG
AGCATGCTGTTCTCTCTCATTTGGGGATGTTTGGTGACGTTTTGCTTGCCAA
TTCCTGAATACATCAGGTATGCCTGAATGAAAGACACCCACTGGCCTGCAG
GTGGCTGGGGCAGACCTCCACTTTCCCCTATGTCCCtgaaatcatttgagttggaagggat
ccttaaggGTCATCTAGTCTGACTCCCATGGTGATGCGAGCAGGGAACATGGCA
GGTGTGAGACACAAGGCAGTGGATCAGCACCTTTGTGTGCCCCTCGTTTCA
CATTCCTGCGAGTAATGCTGTCTCCAGGTGGActgagcaagcagcagcagccaagcgTG
TCCTCCCTTGCTGAGGGAGTATCTTTTTCTACATCTCAGCCAGCATGAAAGG
CAATGAGGGTGGGCAAAACGATCCTGCCAGACCGGCACGTAGCAGGAAAG
GTCATGATCTTTTGTGTTCTTGGAAAGCAATGTGCAAAGCAGTAGCCTGATT
ACTTTTAATTGGGAAGTCCCAGATTTTGGTGCCTTCCCAAAGGGAGTTTAG
ATTAGCATTTCTTCCACTTATTAGAAATTATCACCTCTATGTTGGTTCTGGTTT
GCTCTCAACAATGTTCCAAGCAAGTAATTTTAAACTCCTATGATTCACATGA
TCCCTGACTAATGGTGCCCACAATGGAAAATTCTAGGAGTTTCATGATGAA
AACCATATTTAGTGTTAATGGTATCCTTATATAGCAGGGACTGACGGGAATAT
AGCAACAAAAAGCTACCACTGGTAAAGATAGGAAATGAAGGCTCTGGAGT
GAGACactttttaaatgacatttaaaggacgtacttgaaatattttggtagGAGATGAAATATCACGGTT
CTCCACATTTAGGCGTGATGTTTGATCTTCTTGTTCAGgttctatggaaaaaaaaatgcac
aaaggcaaaaaaactTCTGTTCATCTCCTGTTTTTTTACTTGGTTGCCTCTTCCCCTT
TCAGCTTTGGCCCCTCCCATGTTCCACATCACTGCGGGGTTATTAATCAGTT
GTGATATCCACATGACCTCTAGTTTCAGGTCTCTTTGGTCCCTCTACTCCACT
AGCTGAACTATTTCTAATTGGCTAATCAGCTTATCCGGTTTGGTCCTTGATGC
AAACCAGGTCTAGACATGATCTATTCCTGGGAGGCCCCTGGGTTCAAGGGG
CCAATCTCACACCCTGTCCATCACTATTGTCAGCACAGTACCCTATCTCAAA
GACATGCCAATGAACATTAGTGAGAGATTAAAATGACAGCCTTGGTTTTCTT
ACATGCCTTTTAATCATCGTAACATGCAGTCTAATGTTTCACTCTGTCTTCTC

TCCTCTGCAAGAGCATGTTCTCAAGGAAGAAACCAGGAAGGACGAGAGC
AGGTCAAAGACCACAGCAGGGTGAGAGCAGtggaaaagaactgaaatggagagtgggggga
gaaggaggaggaggaggaaggaagagacaAAAAACAAGTCAGAATCTGGTGGCTAGACA
TGGAGCAACAAGGCTAGAATTTCAATTCCTCCACATGGCTCCAGCTGGCTG
ACATCTTATACTACTCACACTGGCATGCCATAGGCATTTGCCAATGGTGGTG
CCTGTGGAGGGCTGTCCAAGATCCATAGCCATACACCTGAAAGGCAATTTT
CTCAAATGCTGCTCATCTCTAATTGTCCTGGGAAGAGCTGGAGGGCTTGAA
AATCAAGCTGTCTTTGTTGAAGTGCCTGCATATGGACTTAGCAGCTGACTTT
TGCGCCTTTGGATTTCTGAGAAAGCCTTAGCCTGGTCACTCAGCTGTGCTG
TATGCAATTATTGGCCATTTCAACAAATGACAATCAGAGGGCTCCATGCAAT
AGGATCAAATATGAATTCCTTCTTGGTCTCTTCAGGCAGTGAATAGTTTTG
GCTCTTCTCAAACAAAGCCCCATTCTCTGCAAAGTTGTGTCAGTGTTAAAG
GTGGATGCTCTTGTATGAATATCTGCTGCACAGGCAGACATAAAGGTCTCCC
CATGCCTCAGATGGAGAGCTTGCAGCAGTGTTAACAGCATGCACAATCAAA
GTGAATGACCTCACTTGTCCGAGACTTCCTCGTGCTCGGCCAGCTCTGAAT
GAAGACAGAGCAAGTTCACACCTGCCTGCAAAATGCCATATGGACATAATC
TGTAACAAACTTGCTCAGATGCTTCCTTTCTAAAGGGCATTCAAATTTTGcta
gatgtatttttcctctcacagtcatgcttttactgaaaatttaGCTAAGGTAAAATCAGCTCCTTAGCT
TTTAAACTTCCTGGTCCAAAATTATTTGGCTGGTGGGATGTGTGAATTCCTT
TAGCCAACCAGTGGAGCATGTAATTAGCTCTATATGCACGAATGAATTACGC
ATATGTTTAGCTATGTGGCTTCAGCTGGTAGCTAATCATGAGAAGTTTCCAG
CTAGAAAAGGAAACTGGTGAGAAAGTCAGGAAAGGCTGTGAAGCAGGCA
GTGCACAAGAGGTctctggttttccttttcccctttctgcctGATACTGCTTGCATCCTCATTTT
TGTGCTGTGCTTTCCCTGGGATGTATATTCCCCAGCAGATGCTGGGAGCATC
CTGCAGGTCATGCTCTCTTTCAAGTGCCTCATGTTCTACTGTCACACAGCAC
CAAGCGTGGGGGATATTTCTCGATCTGTTAAGGATTAAATTTCCCCAGTTTC
AGAGACTAGTTCAAGCCCTGCTCACCCTTCTCAGCCTGCCTAAGCAGGAGA
TGGAAGAAGAGCCTTGCAATCTCACCTGGACAGTTTATTGAAGTTACTGTT
TGCGTGTGTGTTGCTAAGGCTGTTATAATATATGGTCAGATTGTCATGCTTAA
ACCACCTCATGGTTCAAGCACATCACACTTGCAGACAGAATGTGCTGCAAG
GGCAGCTGTCTCACTGAACAGTAGTGCCACTTTGCTCTTTTCTGTCATCTAT
TCAGCTTTTTCTTAAGGATGTGAGTCTAAAAGCAGGTCAGAGTTGAGGAGC
GTTGCATATTTGGCAATTTTTACTCAGCCTTTCTCAAGGTGCAGAAACGGGC
TCATGTTGAGTTGGCCTTCTCTGAACAAGGGGAATGTAACCCTGCTGGTAG
CACCAtcatatatattatttatatagcGAGTTTGAAGTTACAGagttcagactttttttttttcctttacatttttga
gtggaaaaaaatgatgagaatgTTTTGCATAAGCAAAATTTACCCTGTCCTTTTCAATAT
TCAATTTTTGTAAGACACCCTGGCATGTCAGAGctattatttcttaatataaaTAATACTC
TCATACACAGACTTTCTTCCTGTGAAAATCCATCCTTCTCTTAAGAACAATA
TCATGCCCAGATGGAGTAGGATGGATAGAAAGTAATAAAACAGTTGCTCTT
TTAACAGACTATCAGATCTCACCCAGTTCTGTGGTGAGAGTAGACCAGAAG
TGGATTCAGTGTAAATGCTGCAATCTTTCAACCGTGCTTCCAAATGAGAAG
CTTTGTACCAAACCGCAGCACTGAAGACCTGGATCAGTGCTGCCTAAAGTC
AGCAGAAGTCTCTGGGCCACCTCTACAGTCCCCAGGCATCCAAATGAACG
CTATCCAACTAGGCAGCGGTCGTCGTCTCTCCCCCGACCTCTGCGATTC

CAGACAAGGCCACACAATCTGCAGATGGGGAAATGCTGAATAGCAAGCAT
TTCGGCCTCTCACAGATGTGGTTAAATTATCTTAATGTATTTAAACAAAGTGT
AATGTCAAAGTAGCGTAAATACCATATTTCAGCAAAATGGCTGCTTCTCCAG
AGTTCTTCAAGTCAATGCCAAATGAAATACTTCTCACTGTTTATTCACAAAA
TTCTTTCTTACCCACTGAATTTTCATGTTGCTGGGCTGGGCCAAATAACcagttt
cctttaaaatagaagaacCATTAGTGCATTCCAATAGGCTTTTCACGCTAAGTTAGGA
ACCCTTGCGAGTGCAGTGTGATTAGCctattttggttttaattacTGCTGATTGGAGCCT
ACCTACGGGGAAACTCTGAAAAAGCAGACAGATTTTCAACCCTGCACACT
GATATATTTCCggaggagctgaaggaggaaTCTTGAAGTTTATGGTAGCCACTCGGTAT
ATCAGTGGCTTTTCTACTCTCTTTAAACAGGATGCCCTTTGTGTTAAGAAAC
ACACCTGCACCACTTCAGAGATTAATGGGCTATTCCTTGGCAGCACTTGTG
GACGTTTCAGTTGTCCATTAGGATGATATAGGACCAGAGGAGAATTTTTTTG
ACCAGCTTTAATTGAATGGTAATCTCTTCTCAAAGTAACCTCAGTTTGCTGG
GAAACTTGTAAGCCTCAGGGAGAGGAGTAAAGAAATGACAGAGGCCCCC
AAAAATATAAATCCTCCTCCATTAGTCAGGTCTCTTCTGTGAATGAGTTAAC
TTTCAAAGAAGCATATTACTAAGTATTTtatcaaagcaaacatttaatCAAAGTGAAAG
GAAGAGATACTAGTCCCCTTCTGGAATGGGATGGAGACTGCCAAGATGTGT
TTTAATtgcttaagaaaacaaactatgTCAAACACTGGGTTTTAAGCAAGCAGATGCTC
ATTTATCATTCGTTTTTGTCTGTGTACGCTTGGGATCCTGCTGAACATGagttttc
tgaaatggaaatgaccATATGGCCAATACCTTCCACATGAATTCAACATGCCCTGCCT
CATGTGAAATGTCTTCTTTCAatggtgcttttctttttcttcttctcttttcctcttttcctttttctttttggttgtttttt
tttttcttttctttctttttccccttttttcttttttctgtttttcctttttcctgttacATTATGGGACAATGAAAATAGAA
CATCTGCCTTTGTGGGAGTCCAGCTCATTGCAGAGGCAGTCTATGACCAAT
GCTGTGCACTACTGATGGAGTCTGGATACTTTGCAAGAGCTGTGCTTTCATA
GCACCATTTCTTTTAGAGGTTATCTCacacataccaaaaaaaaaaaaaaaaaaaatctttgctacTG
TTTTAACCCTAATCTTAATGATGCCAGAGGGCCCTCAGgaggcagcctgcagcttccctg
gggctgtgcagggctgttCAGGACTGACAGTGGGGTACTGGCATGTGGAAGATGTTC
TGGGATGGCAGTGGTTCACTGGACCCCAAATGTCTGGAGTTGGTGGCTGA
AGCAGTGCTGAGGAGCCATCAGGACCATACCACAATGCTGAACCGTGACA
GAAGAAGAGTTAATTTGCTGCCCAAAAGAGATTATATCTGATTTGCTGCGAT
AAATGGAAAGTATTGCTTCCACTGAGCATGGACAATTCCTCACCAAGTGTT
GCGCATAGCCTTCTCATACCACAAAACAGCTGAAGCTGGCCCAACTAATGA
CCATGCACGTATCTCGAGACTGTAGATCTCACCCTCCTCATAGAGACATCAG
TGCAGTCAAGGATCCTGCAAACATAGCCCAGGGACATCATTCAGAGATATC
CACATTGTCTTAGTCAGCTCTGTTCTTCTGCATAGGGCTGGAGTTGAATCTG
GAGGAACAGTGTGATCTCTTACGAAATCGGTTGTGCCTATCCCTTCAGTTTT
GTAGAAACTCAGAGCAATCTTGGCTCTGTTTGGGCTGTAAGCCCTCTGCCT
TTGGCAGTACATAGAGTGCCAGCTTTGAGCCTCAAACCTTGTGAGGGACA
AGATCAGAGAGTTTGCAGATGTATTGTTCTAAGTATGTGAAAGCAAACTGA
GGCAGATGCAGAAAAATCCTGGCCGTGAGAGCTGTTCAGCAGGGCAACAT
CTTCACAGCTCCAGTCCTCTTCAAGGTAAGGGAAGCCAAAAGCATCAAAG
TGTTCTCCTCCAGACTGAGGAACTAGCCATGCTCTTTCCAGAGAGGACGGG
GCGCAGGGCATTCCTCAAAGCCACGTTTCTGCAATGACTCCATCTCTGTACT
AATGTTCATCTCAGCTGTTTCCTCTCTCAGCAGAGCACCAGTGGTTTGTCTG

TCTGCCTGTTGGTATCATCCCTGTCCTGCCCTACTGCTGCATTGCTCCTCCA
CCTCCCC
>TCONS_02587419
GTTTACATTGTGGACAGATATACCTATCTGTACTCAGAGGATGCAAACTCAG
GATGTGTCCCATCTATTTCCCTACCACAGTCTCAGCATTCTCTGCTTCAGATT
CCTCCATTCCTCAGGCACCACCCTGATTCATAATCTCAGGAAAGGAGATAAT
AATGATCTTGCAGACCTGCATGATGACTGACTTTGCTGTGGATTTTCCAGCC
CAATTGATTGCCCACCTTCTGGCAGTTCAGTTCTCTGCTCACCCTTCCCAGA
CTGGCCACAGAGTTTCAGCACCTCCCTTATGCCCACTCACCCATGTTTTTAA
CTTTTGGTGAGCCCTTCACAAGAAGCTACCAAAGTCTCCAGCAGCTACTTA
TGGCATTACGCCTACtcactgaacagcagcaaacacCACTCTATTGAAAGACATTGGAA
TGAACTGTGAcactttttgctgttttggcAAACAGGATGGTTTCAAATCAAACTAGAAA
TACCTGCCCAAAGAACAAAGTGATtgtattctgaaagaaaggagagaaagtcTAAATGCTG
TACTGACGGACTCATAGAATAAGTCACCCCTTTGGTGTGCCAAGACAATTA
AATGAAACCTTGTCTAAAACAGATCAGGTGACTCATGCCCTAAACATACCT
GTTCTTCTGCACTGACCACCAGGTGAAGCCCTGGGTGACAGACTTAGTTAC
CAACATCTACATTGCATACGTAAAGCTAAACCGGGTTAATTTCTCTGGTTGC
AACCAT
>TCONS_02608761
GTggtgtttgtttcctttccataaAGAAGACACCAGCTCTGGTCGCTGATAAAACAAAGC
TTGGCAAAGTCCCAAGCACAATATCTTGTTCTGGGAATGCACTCAACTTGA
TCTTCTCATTTACCTCACAGGCATTCATTCTTCAAAAGCCAAGGGCGTACAT
CTTTGGGAGATGAAGCTGCAAAAGAATTTTGAATAGATTGAACCTGGGGAT
GCTTCTGGCTCAGTTAGTATTAATGGCAAGTTGGCTTTGGAAAATGACAAA
CTCCGCAGTCTGGTGCTGGAAGAATTAGAAGGGCTTGAAACGTGGGaaaataa
tggagaaacagCTTTGAAGTGGTTATTCATCTCCTTCAAATACACCCACCGGACA
ACTGGGACAGATACAAGCCGCAAAGGGAGGTACCTCCCATGGTCAGCCCT
GCAGAAGTTAGAAAGGAGTGGGGAGAAGAAATGGTGGAGCTGGCAACAT
CTTTGTGATCTCTCAAGTGGATCCACAGCCCAGGTGGAGCATCCTGTTCTA
CTAAAGGTTGTAGAAACATTTCTATCCCACCTCTTTGTCTTTGGGCTGAGTC
CAGAAAACTTCAGTGACATCTGGGAAAAAAGTGATGAAACT
>TCONS_02618330
CTTACCACTACAGAGGTGGATGGAGTCAGCATTAACCTAAGTTTTACAATA
AGCATTTGCTCAGATACAAGGTAAAATGGAAACAACGTCCTCTTCACTGAT
TCTTCTGAAGtgaCTGTCTTACTACACAGACTCCTATGTTTCAGAGGCTGCAT
GAGGGATATGCTAAGCCTGGGACTATATTCACTGAGATGCACTTCACTTTTG
AGGTAATGTAGTATATATACTATAAACCAGGGTTATGATTCTCCTCAGGCCTG
AATAAGTAAGAGTGGATGTAAGCTGCATTTTCCTGCAGCCACCAGGAACTA
GTGAAGGAGTGAAGTTGCTGCCACCAAAGGCAAGTATTCCTAGTAGTATAG
tgttacagaaaatgtatttcttactagttctgtttttcctgtctgatgggatacatttttattttaaaggtaaaTGCATAGTCT
TTCAATAGCGCCTCTCAAAAAACTGTAGGAGTTTTCCACGAACTCTCTACA
TTCTAAATTCCTCAGTCTCTAAAATGTAGAATAGGATTTAGAGGCCAATATG
ATAGTCTAATtgctttttactttctctgcCTGCACAATGTGTGCTACCTATGTGTAGGATA
CCATAAAATGGAATAACATCCATTCAGTGTTCTGAGGAGGTTTTATATCACAT

AAAAAACTACTTATGCACAATTCCTACCTTAGTTTACTCTGCACAGAACTCT
GCAGGAGCCCTGATGAAGCAGTATGTACAGCAAGATTTTGGTAAATGAGAG
TTTGAGATAATGatcagaaagtaaaataaatgctaaaagctgaagaaaaaagtaagataTATTGCTTAA
TTTCCCCTGGGTTTTGATGCCAGATAGATGACTTGCCTGGCTACATGTCCAT
AAAGCAAAAAAGCATATTAAGAGAGTTACTTTTTCTCTCATTGTAAGGGTA
AGATGCATTTCTCAGCAGTAATAATGGAAGTGCAAGCCTTCTTgctttatgattttattt
tcacccATTCACTTTGAGATAGAAGGGGAGGTACTCAATCTTATACCAtaataaaactt
tttaaattatgCAAGAATTTCACTGCTTGAATCATCCCAAATACTGGCACATCTGGT
ACATGACACAGCTAGAAGAACTGAGAATTCAGATCAAATGCTATATCACAG
TATTTGAAAGTAGGGAACAGATTGTTATGTGCTGAACTTTCAATGAAAATTG
TACTTATTAAACATATTACCTCACATTCAAGCTATTACGTTTACTGATTTTTAG
ATTTGAATCAGTGATATGGTCTGAGGTATGTCGTGTTGTGTGACTTTTTGTC
CTAAAATTCTGTCTTTGTAATGGGGGTAGGATTTAGCACATGGCTGGCAGTT
GACGATTGCTTCAAACTCTAATATAATTTTTCACTTCCAGTGCATGGAGGCC
CAATAGCATTATTATTAAAAGTAATTAGCTGTAACAGTCACCTtgtctgagaagaaatt
ttgtaATTCAAGAAAACATTCTGACTTCTTAACCTTGCAGTTTCTTCATGCTCCT
ATTCACAAAAGCTTTTATGCGCCATGACTAACTTATGAAGGAATAAAGATTT
CTAGCTTACCTGTCCCAGTTAGCGGGGACAAAAGGCACCATTCTCATTTCG
TCTTTCTAGATACTGCTTTGGATGGGGACGAGGGAAACATTATTCTTATATTT
CTTCCCTAAGCATAACCTTCGCAGGGCAATCATTTTTCTGCATGCTCTTTTG
CAGAAGTTAAATGGGATTGTGCTTGTGTCCTGACTGCCCCATTCCGCTTTtcc
cagaaacaaacaaacaaaaacctccaaAAAACACCCATGTCctttctttggcttttctatCATTAGCATT
TCCTAGATACTGCTGCTAAATTTTGTgacttcattactttttttttcctgacaggtagaaacaacaaatta
aaatcCACTTTCCTTCGTTGATTCCTACTAGAATCTGTGGAGACATGCTTGTAG
CAAAACACATTCATGAGGGAGGCTTTCCTTGCAGGGTGTCAATTTTGATCA
ACCTTGTTTCAATGTTAAGATATAATGATTTGGTAATTGGAAGAGAACAGAT
TCAGGAGCAATTAGTCTGGCTTGCAGCTGCAGTTAAAGAAACTTGTGGAGT
GCCAAAGATACAAATTCTGGTCATGTGTGACTTGTTAAATAATCAGTGCATA
GCCCCCTTCCCCCCGAGGACTGAAACCTGCATGGTAAATTCAGGGAATTCC
ATGCAGTCTGATAGCTGAAGTGGAAGATGATCTGCAGACTGGTGAGCTGGA
GCATATTCAAGATCTAAGCTACCGCTGTGACTGTTATACAGCCAAAATGAAC
ACAAATTGAAGTTTCCCTTTTAACACTTTAAGCTTTTTTGTAGGttcattctcctttctc
tttcattaaaaaaaaaaaa
>TCONS_02618541
CTTCTACATGAAGcggattttatttaaattacaatCTAGCATTATAAAAATTCCAGCGAGC
AGCAGGGTCAGGCGAGGACGCGGGGAGCTGGGCTGCCTCACGGCTGCCG
TGTCCTTCCCCAGCTTCTGACTGCGGCCACTTTTGCCACAAGGAGATGCAT
TGGCCTCAACGTTAACATAactattgcatttcttttccctttcagagaagaaaaaaaaaaaatcattcaacT
GTTTCACTTGCAaggaaacatctgaaaaatcCAGCACTGAGGTGCCCCAAGGGTGGC
CTTGTATGAAACACCAAGCAGGCTGTCCTTTACTGCTTTGAAAAGGGTCAT
GTCACCATGAAGTTTCAAGCCATTGCTGAGGGGCCCATTCCCTTCCTCCAC
CACTGGAGGTCAAGTTCAGCCTGCACTTTAGAGATTGTGACAGACAGAGT
TCTACCATGAAGTTCGGATGGATCTGCAGAAGCACAAGGCAAGAATACGCT
ACCAGCGCTGTGTATTTTGGAAGCAATAGCCGGTGTTACAGAAGGCTAACC

TTGAAAGTTTGTCTATGCAGGGATATAACGTGTGTAgtttctgcaggaaaaacatAACA
TTTACAGTTCACTTagaaaacattcacaaaaaGTGGTATTTGTGTAATAGCAGTCAGAT
TTACTTTGATTTAAATCAATACAATATCTCGCTGTTTAATAACTCTCATAATAT
ACAGTAGTGTTCACCATATgtccaccaaaaaaaaaaaagcatccaaccacaaacaaaaccaaaccagca
GTGCACAGCATGCCAGGGGTGTGTGCTGCCTGCCAGGGGTTACAccacacagcc
agcacagggcaCTGCACACCTGGGGCACGGCTGTCACCAGAGCACGGGGCTCA
GcgccagcagctcccaggcaccCACAGCGCTGCTGGATGCTTGGCAGATTAACAGTCC
CTGTTAGGAGTAAGCTGGCTG
>TCONS_02641075
gctgctcaccatTTTGCTCTCATGTTTTATGCAGCATCTTTCTGTTTGCTCATCTAC
AGGAATACTCTGCCTCTAGCCTGTCTGTTTTTGAAAAGGTGACTGCTGATG
ACAGCTAAGATAACAAGAgggtggtattttttttttcatgagcaCGCCTTCGGTTCTATTAAT
AAAATCCTGAATTTGACTAGTCCTTCTGAGAACTCCCGGCCTCTCTCACCCa
ttcaaaactgtatttttaagctGTTGTAGGTGGATTGTCGACAATGGTTGTTATGCAAACT
CTGAGCACTGGCTTCAGGAGCTTCTTGGAAACCTGCAATGAGTAATCTTCA
TGTTTCTGCCAGGTCTACAAAGAGGGAGGGTGCAGTTTTGCCACAGGgctgtt
cagatttttttcctcctctccttttccataTGGGTAAGAATAAAGTgaagaaacaacattttcagagaagaaaga
ctTGGAAagagtttctgttttctgggagTCTGCTACAGCTGTTAATTTGTGGAAAACCTCA
GTGGAAAGTAAGCAATATGGCAGCATCACTTTACACGAGAATTCAGTGATG
GCCTTGCTCCAAGCTGTGGCTGATTTAGGTTCTTGGCTTGCGCAGCCAAAG
CTTCATGTAGTGTCATGCTTTTTTATAGATAATGTTGATACAGCTGTGGAAGG
ctctgatgttcttttttttttttttaacatggaaTTGTCGTCATTCAATCAAGCTTAAGTATTGTGGA
GCTGTGATGTTGTTTGTCTTTGGTTGTTAGTTGTTGGCGGGTGAGTCCGAG
ACGGGCCGAGATGTtgtttgggaaaaagaaaagactccctcccttcctccctgccttCTCCACAGAAA
TGCACACAGACCATAACTTCCATCCAAAAATGCAGGAATGCTGAGAAAAAT
TGGAGCTGGACTCGAACATTGCGGGCTGGGCTCCCCCACTCCCCGGCCTCT
CCAAAGCACGCACGCTCTTCTCTTCCTGTAGACTTTGTTCCTCTCCAGCATT
TGTCATGCATAGTTGGTGGCACATCAAGATTTGAATACTGTACTGAATGCA
acaacctctttttttcttggatggACATTTTGTTTGCGGGGAATACAGCATGGTGCTCAGCT
GTCTGGACAGAAATGAACattgaatgaaatgaaattttcagcTATGTGACAGCTATTAGAT
CAGAGATTAGTTTTCCAGTAGAAATGTCCAATTATTTAAACCTCTCCGGTAG
ATGAAAGAGCTTTTGACAATCTGTTTTGCTGAAAAGCCCTGCAATTCGTTT
GGCATAGATTAGATGACCTATTAGAGatgagaaaaagggaaaaggatttATAATTAATGTC
AGCCCAGCTGTTAACTTGGGGAGTATTGCTGAGTTGTAGCACAAGACGTAT
CTTTCAGCTATTTCTGCAGTGACGATTCATGTGTATATTGCAACATGTGTCAG
CAGCCTTCTGTACAGCTGTGCTGGGTTGAAGGGACTAGAGTTCCCCATTCC
TAGGTAACGCATTAGCAtgcactgctgcttctccaaGGCCCTGCCTCCTTTTTGAAAGC
AGGCTATTATGAATGGGTAAGCCCCATGTTTTTCCAGTGCTACCTTGTCACA
CAATGACTGATCCGCTCACCTCCGTATCTCTGGGCTATATGACTTTGAGTGT
ATCTTCCTTGCCAACTTGGCAAGGTATCGTGTTTCTTTCCCTGAGGGTGACT
TAGGGCAGAATCCTTGAATCCTTCAGAGTTTCCTGGAGCAtctggaaagaagaggaa
aaccaGAGATGGGCCAGAAAGAGGCAggcagagaaagatgaaagaatttGGGGGTGGGA
AGTACTTGCTTTTGGAATAATCTTCCTCCTAGATGGCCTAAGGCAAAGTAAT
TTACACTTTTTGGTAACGTGCAGGATGGCCCAGCCCTTGCTCTTATGAGTAA

ACATTTCAA
>TCONS_02645442
CCGCCGTTGCCATGGCGGCGGCGCGGTCTGCTCGGAGCGGGCCGGGACGC
CGCGCCCTGTGACCCCTCCCGGAGGCAGAggatggctgaggttggaaagcGCCTCCGG
AGGTCATGGAATCCGCCCTCCTGCTCCCCTAGAGCGCGCTGTCCGGCACTG
TGTCCAGGGAAGGAGATTCCGTGGCCCTCTGCCGTCCGCCCCACACTGAG
GCCTGAAAGATTCCCCTTCCCAAGTGGCGCTGCGCGCACACTTCTGCTTTG
CGTCCTTCCAGTCAAAGCTGTGTGAccccctgggctgcctgtgACGGCCCGCTGTTC
GGCGTCCCCCAGCCTTCCACCGATGGCCAAAAGGCTCGAGAAAGCAGGAG
CGTGCCCTGCCTGGGAGGAAGCACCAGGTGTGCCACGTGTGGGACGGGGC
CTGAGGGGACGGcgtggagctgtgccaggggagggccgggggtcagggaaaggctgtgattttttgtgctt
tgaaaCGTGAGAAATAGCGGAATACGTGCCATAATATTTAACTCTTTAACAAAT
TTTAATCACATTCAGATAttgagaatgaaaagaaagtgaagcaTTCAGCTTTCCGAAGCAT
TCAGCTCTGTGAATATACATGAATTAACCAGCACAGTGCAGGTATAATGGAA
GGGCTTCCTGGTCATTCTGGAGCATCAGCAGCTGTACCTGGATGTGAATTG
CACTGTGATACAGATAAGTTTTGCATTAATACTATCCATTTTTCAATGTGTAA
TGTTTTCATATTCATACAAGTGAAGGGTTTATGTTTTTGCTTGGTATGTGTAA
CAGACACATATAACAGTTGCCTTAAAAACCTGTTGTTTGTCAAGAAAAATT
AGTTGATCCGTTGATCTGAAGTCACTCATCTTGTTGAACCCATTGCAAAGG
GGCTCAGACTGAGGAAGCAGCTTCTGCCTCTTGCTGTACTGTTAGACTTGC
TAGGTAATTCTGGACACATTGCTTAACCTTCCCAAATCTGAGTTTAATGAGC
TGGATATGTACCACGCCATATGATGCTTATGCTCACGAATAAAGCACTTGAG
CTCTGTGAATGAGAAGTGTTTCAGTTTGAGTTCTAATCCTCCGTTCATCAGT
TGACAGAATGCTTCCACTGTGTTTCCTTATGTAGCGAGGTGTTCTCTGCTCT
GAGATAAACTTGCCTTTATTTAGGAGACCTGAAGgaaaaacgaacaaacaaacaaatgatg
CATTTGCTAATTTTTCTGAGATTAAACATTCAGTAGAACTCAACAAATATGT
TTACTAGTTTAATTGGATTACATTAATATACATCCCCACAGCTTTTATTACATA
AGCCCTTTTCTACCAGGGATTTAGTCTATTAGTTAAGGAAACCTGAGGGATT
AATCTGATAGCTGCAGTCAGAAATTACAGTGTAATTAAAGTCACCCAGGAA
ACAGTGATACTGAAGTCTGCCAATCCCTGCAAAACAAACTTGTCAATTTTC
TGCATCTAGGATAGCATATAAGGTGACACATTAAAGGGGCACAAGTTCTGC
ACCAGAAATAGGCCTGTTAAGGAGGAACTTGTGTACTGCACAGTGTAGCCA
TCGCTGTTCTTGCTACCAACTACTGCTGTCTGTTTTTGAATAGACTTTAAAG
GTTTTGGTGTTTTATTAAAGGTCATGTACAGTGTGGGAGAGGAAGTATAAC
ACGGCTTGCACGGAAGTCTATTTTAAAGCTTACTTTACTAATTTAGTTGGAC
CGAGATGTTACTTCATGTCCAAAAGAAGATAGAAAATGCAATGGCTGTAAA
GAAGAAAGttgcagagcactggagcagcctTGTTAGTAGCAGGGAGAGACGTTTTGTC
ATGGTCTGTACTTGTCAGTGTCATTTGCTGCAAGCAGGGTAATGGAGAGCA
TGCATCGGTGCATGAGTGGAGGAGGAGATCTGTTGTCTTCATGAATGGCAT
CAAGGAACAGCACAGCATTCCAGAAATTCTGCAGAGTGTCAGTACTTGTGA
TGTGTTTGAGCCATTCTAATAGCATAATATACGCAGGAGAGATTTAATTTCAC
TATATCCAAAATACACAGCATTTGGTATTTCTTTTGTTCTATGAGACATAAGT
CTAATGATGCATATTCTAGTTAGTGCAACTTGCTATCAAGGTTGTAAAGAAA
TaagcaacaagaagaaaataacgtatgtatttttctgttgtcagaGTAGACTTGTATATATACTTCACA

GCTTTGTCTTCTTGCTCTAAGTTGTAAAGTAACCTTCACATAACATTGTGTA
AATGGAAAAGTTCTCTGCCAGAAAGCTAAAGCCCAATATCTGTTGCTTGCA
AAGCCACAGAGGGAACTACCCATTGTTTCCTAGGAGGAGATGGTGCAGCG
ACTTGCTGTGGCTTTAGCAAGAGGGCAAGGCTTTGTGATAGAAGATAAGCA
AAGTATGATGACTTAAGTGGAGTGCTGAAGCTTTTACTGCATCTGTCTTGTA
CAGTGTTGAATAGATTTTTAGGAACTTGCCAGTCAGGAGACCTGCCTTCGT
GCTAATGTGCAGACATAGAGAAACTGCATGAACAGAGGCAGGAAGCACTC
agaacagaaagggaaaatgagcTACTGATGCACACAGAGCTTTGCTTGGTATAGCAGG
CAAAGGAGGATCCAGCAGGAACAGAAGATATCTCTTCTGTGCCTGATCAGC
CTGAGAAAAGTCCTTAACAAAACAAAGTGGCAGCCTGGAGAGtataaaacacag
gaaatgaTGCATTTCCAGTCCcacttttgcttctttctcagcAGTCGCAGGGTGGGTGAGTAG
GAGTTGCCtgtgccagctctgtgcagcctggcaAAACTGGGGCCAGACCCGACGTTGTTG
AGGCTGCTTCCACTTTTGTTTGTGAGGTGGAGGGAAGGACACCCTCTGTAA
GTGTTGGTATTAATGCTTTTCTCTTAGttaatctgttttgtttgctgctgaggCATTTTACCTT
CACCtctaaattatttcattcttcatcaaaatccaaagaaaacagagattcAAAGAAAGCACCCCTTAGA
ATAGACTGAACGCTGAGTATTATTTGTAAGATGTATGAAACAGGAAACGGG
AAGTTAGAAACATTCAGGGAGTCGGTATGCATCCAAGTAGTCAATGGTTCA
GGGGGCAGATAAACCAACACACCCCCACCCAGCACTGCATGGCTACACCA
CCAGCATTCAGGAATTCTGACCTTTGCTAAATCTTTAATATCTTTGGTTATCA
AACCCAAACTCGCTCACATGCCAGAAGCAGTGTGGTACTGGGTAACCATGT
AGGTAATTTTCATGCTCAGAGAAGACAGCTCATTTGTCAAACTGCGTCAGG
TGAGAGTGCTCAGCAGGCCTgaatttctgcagagaaaaaggacCGGATTCTCCTCCCTG
TTGGCACCACCCAGTCTGAGTCATACAGGCTGGAAGCAGTAGTACAGCTGC
CAAACTTCCTCACCTAGATCACGCTATCTGCTATAGGCTGCACTGGGATTAT
TGAGTGAGGTGTTTGGGTGTAGAATTAAAATACGTTGAGCACAGGATTGTA
CTGTTGGGGATTTTTCCACAATGTTTCTTCTCcaggaaatgtttttcctctctaGCATTGCA
GGGTAACAGTGTAAacgtttgtttttgttggttcaTTGCCAGACAAACGTAAGAACATGT
TGTAAAAGTCACATTTTGCTGGATTTCGTTTTAGAATAGTAGAACTGTAGAA
TGGCTTAAGTCAgagggaaccttaaagatcatctagcttcaactcctctgccatgggctggctgcccccaccag
ctcagggtgcccagggcccatccaacccggccttgggcacctccaggaatggggcacccacagctctgggcagcagtg
ccagggcctcaccatcccctaagcaaagaatttctttgtaacatctaacctaaatctcccctcttttagtttaaattcCGTGT
AGAATTTCAAGTGTGTTTGGCACCTGCCATTCCAAATACAGACAAAGTTATA
TGATAGAACTCAGACTTTGTAAATCGTAATGGATTTTGATCACAGGATATGA
TGCTACTTGGTCACAACAGCCCAGATCCCTGTAGGACACATACTTCCATTGA
TTTCACTGGAAGTTAGGGGCCACAGAGAACTCTGTGGTTCTTTTCCTACCA
GCTGAAGTCAATGAAACCACCTTTTGAAAATGGGGTTCCAGATATTACAGT
CTAGCAGACAAAGCTGGCTTATCCCACATTAAATGTCACCTCTGAGAACGG
AATTTAAGTGATGATGGCGTAGTGGAAGTCTaagacagagccaaggccaGAGTGTAA
ATTATCTGATGCCCAGCCCAATGCATGAACCACAGTGCTGATGTTTGATTAC
TGTGAGGTTGGATACTGACTCTATAAACAACAACTGCAGTTTTCAAAACTT
ACCCGCAGCAAATGCAGAACCCTGCCTTGACATGGTTTTCTGATGGTAGAA
CTCTGCTTGCTGAAGTCTATTTACACCTCAAATTTTCCAAAAACAAGACTTC
AATTCCTCATTGCTGTGAAGCAAGCATTACTTTTAACAAATTACTTAATTGC
TTTTCTagccatttcttttcctgcatttcttAATTGAGTTTGTATAGAATATTTCTACATGAATG

AGTGCCTGAGGCAAGATGAGGTCTGAGCAGAAACCTGTATCTTGGTTTCCT
AGTCAGTGGGATGCAGGAGAGCCTACTGTGCTTCTGTTTAGGGATAAGAAC
TTGGTTAAAGGCATGCAACCCAACTCATGTTTCCTTGTGCTTGCTTACAAG
GAGAATATCAAGGTCTCAGACTGGATAATGATGCAGAAATGCACACTGACA
GCGGTCCCAATAGCCGGGCTCATGGAAAGAAAGTCTCAGATGTGAATGAA
GGATGTGAGGCTGCAGCCAAATTGATTAGAAGCAAAGAACTCTCAGACAT
CTGAAGTGTGCAAAGCATAAAGCTGTCCTCTAATTTCCCAGTTTTGGCAGT
TTTGTTGCCACTTGAAGGTAAAGCTGGTATTAATCACTGGGGGGAAAATGC
AACCCTGCTCTCTTTGATCTTTCCTCCTTTAATCAACAGTTTCTAAGTGCAAT
AAGACATTTCAGATGTTGCCAATGTATTTTTATCTGGCAATTGTTCAATGCGT
ATCCTAAAATGGAGAGGTGCatctgctgaaacacagcatggTGCTTCTGGAGGAGGAA
ACCATCTCCTTTCTGGCTTTGCACTGTTCATTGGTCTGACAGCCTTGGATAT
CCATGGAAATGTAACTGTTCCTAAGATGAATCCTACACGTTGCACAGTTGCT
CTGCTTCCTGTAACTCATCTAAATATTGGTGACTAATTATTCTTGAGGAAACA
TCCTTTCTCTGTGGAGTTAGCTTTGCAAGCAAAAGGACATTAGAAGCAGCA
CCCTGACCAGGTCACTAGCCAGCCAGGAGAGAGCAGGGATTGCAGGCAGG
AGGTGGCAGCCTCGAGTAGCAGCacactgctttctctgctcaGCTATGCTCAGACCTG
AATGCTAACAGGAGCAGGGTACAAACAGCTTGGGGTCTGGGTCCTTGCTTT
AAGCACTGCAGCTTGAAGATGGAAACCTAACTTTGTCCCTCGTGCCCACTA
ATTAAAAACGTAGCTAAGCTGCTCTATAGGTGCAGTGTTGGAGTTGTATGG
GTTTGCtcagaatttttaatattttgttcaaTATCTTCAGAGGGAAAGAATGTAGCACTCTTT
ATTTGATCTGTCCCGTTCTCATCTAggtttatttcttcttgcaaGGAAAATTATGTTGCAG
ACAAGAATAAGTCCCCTCGAAAtactgtattgttttgtttgtgtgtctAATTCTTGAGCCCCA
TCCTGCAAAGCTGATGGTCCTCCATGGCCTGGCTATAGTCCACAAGGGGAT
TTATAAGGGAATTTTGCCTCCCTCATGAGCTACAACGAAGTAACGGCAAAT
GAACCTACTTAGATTAAAGATGTCACTGACTGAAATGTTCTAATCAACAGA
ACCTTGCAATTATCCTCTCTCAGCAGAGAGGGGAACTTGATGACATCattgtttttt
ccctgtgaaaacGCTTAGttggaaaaagacaaaaaaagattcAATGAATCACATGCAACATCCC
ACTTGGAATCTTTGAATAATCCCAAAGAGTGCCATTCACTGCAACAATCAA
AATGTAAGCCAAGAGAGTTCTGCAGCATTGCTTTTTGCCCACTGGcCTTCAT
CTGCAGAAGATACATCGGTGAGGTAATAGTGGTCTTTCTAATGCAAAAGAG
TGAGGGACGATTTGTTGAGCCACATTCCCTGAAGAAAGAAGGCTTATGTGC
ACAGATTCTGTCTGGCCCAACAGCCAGCACTAGAACATGGAATCCCATTGG
GCAATTCACTTCAgtatgaaaaagagaagactccagTTTTCTGTCAGGACTGTCAGCTG
AAGGGCTGAGCCTAACAGCTGTTCTGTGTAAGAGTTTGAACtcaactgtttcattttct
ctccttcacagCCGTATTACTGGAAGAGGAGAATGTGCCAAAAGGTGTTTTGCTC
AATGAAAAAGTGCAGTAAATTTGAGCCGTATCAGGTCTGTGCCGCTGGAGA
aatatgctttcctttttaaagtgTATGGAATCCTACaacttcctttctgtttcttggtTTTCTTAACATAAC
AGTGGCAGCAGCATTGGCTTCTGTAGAATGTCAATAGAAGTTATGCTTCAG
ATCCAGATGTGCAGAGGAAGAAGACTTTCCTTCCACAGCTCTGTGGGTCCT
TCTAGCTGTTACATACCGACCTGTCACAGAGGGGGTTCTGTGAGTCCTCCC
TAATTCATATTAAGCACAATTGGGCAGATTTTATTAAGCAGTTTAAAATCCTG
TTCTGACATACGGCATCTGGTTTTGCATCACTTGAATTAGCAATGCTCCCAC
AAGCTCTTCTCTTAGCACATCTGAAGAGTGTTAACCCTATCCACTGGCACTG

CtagatggaaaagaaatcatcTGTATGTGACCTGAAGCCCTGTTGCCAATGCACAGGCA
TATAGCATTGTGGACGTGGCTACAGCCTGTACAGAAGAGAATATAATTCTAT
TTGGAGGGCTTGACTATTCAAGCTCTgtgaacaggaagaaaagggctGACTCATATAAC
AAGCCAACGTCAATGAGCAgaatcacattttaaatgagGTCACTTAAACAAGTGGTTT
CTTATACTGAGAAAAATTACAGGTAGATTGAAATGGTGTGTTTAAAGTACTG
CTTGGTGGTTGGGGGCACTAAGGAATCTATTCCAAGTGTGGAAAAAAGCCT
TCACCACATATGCACTGGAGGTCTTAAGAAGAAGGGATAGTTGGAGTGTAA
GGGATAATCTTGAGCTGTGAATGGCATGAGCAAACATTTAGGGAAGCCTAG
CTCTGTATGGTAGGAAAGTCTCAGGGAGATGAGCCAGTACATCTGTGAGCA
ATGGAATCAGCGAAGCCTGGTTTCCAATGGGTATGTGTCCCATTCTGGGCTC
TTTCACATCTAATCCGGTGCAAGATGTGATCCAAGCTTTTCCCGTGGCCTGG
ATGGTCTTTGTGTCTGTCGTAGGACATCGTCTAGTGAGTAGGGATTTGGTAC
ATCTTTTCCCATGGTTGGAAGTGCTGTTTGGGTCTCTTCTTCCCAGTTGGCT
GCCCATGTCTGCTAAACTTGCAACAAATTTCTATCTTGAATTCTTTACTTTGC
TTagaaatttatttgaaaagcacACATTGGATTCAAAAGTTATTTAAAGGGGAAGTGAA
AACAGGAGAATATGCACTCATACACCAAATGCAACGGCATAGGCCTTTTGC
CTT
>TCONS_02645912
AACAACTCCATTTTGGCCCACATCTACTTACAGCAGTGACAGTTTGCTCAG
GCACTGGAATTTCCTCATATAGTTAATGCAGGAGTACTTAATGCTATAATCAG
TGGTGTTGCCATGTCTCATAGCATGTTCCTCTTGGgtttgctgtttaaaaacacAGATGC
TTATTTCAATCATGGAATgctatttatttgaaaactcTATTTACTTGGAACATCCTTTCTG
AGGTGGCTTTTTACTTCATTAAATGTGTGGTATTTCCTTTTCAACTTGATTTC
TTGTGGGatctcattttctgatttttggtAAATCTCAGCCTTTTCTGTCAGGGCCTGAAAG
AATTTGACTGCTGTTTGTGTGAggtgctgcttctgcctctccTGTGCTCTGATATTGGGG
GTGACAGCAAAAGCAAGATTATCTTCTGAGCTACAGCTTGCACCCTTCCCA
ATAAAGGGGGAGCACAAGGTGCCTGGCTCTTTGAGGACTACTTTGAACAC
TTGAAGCTGATAGTATTATGTTTCTCCTTCTGGGATGGACCACTTGTTCTAG
GTGAGCGAGTTGAAATCTCAGTGATATGGTTATTCAAGCTCTCTTGCACAAT
CCGGAATATGTTTAACTAATTAGGGATAACTTTGGgactgaaataaaggaaaaagtttACT
TGCTTGAGTTGCTCAGGGGTGGATATATTATACTTCCATAATATATCTCATATC
TAGTCAAGGTTATTCATGTAAAATTTCTAAAAGCATACTCCTATAATGGGAG
GGAGGCTGATGTGCCAAAGGATTCGGGGAGCTTGACACAAGAAAATCATG
GTGTTTACTGTGATAATCAGATACAGGACTGACAGAAGAGAAAGTAATCAT
AGGCAGTTTTATTATGGTTGTTTTTTGAGTCTATGAATGTTGTCAATCCAACA
GTAcgtacattttgtttttaataaatatgtaCTACTGACTTCGAAATTATATTGAACAGTGCATA
GGAAGGAATAGAAAACTGGAAGGAGGAGAATGGCATTCGTGTATGTCATTT
TCCTAATTGTGTTGttcaaacagtttttttcttttggcttctgTGTTTAAGGTGATTTATTGGCT
CAGTTGCTATAATCCATGGGAACAGAGACGGGAATTCTGTAGATGGAAtatcaa
aggaagaaagaataggCATCTAAggcattttaatgcatttctaattttaataatgctttaaataagattattacatatttttac
aGGGTATGAAAATATAAGGAAATGCACTGTTCTTTCATCCCCTAGAAAAAGG
AATATACAGGCACTTAAGGTAGATATTAGCCATTACACTGAAATAAAggtgaaat
gaaatattataATGACGGAATCATGGGAATTTCTGCCGTAACCCTGGATCTTGACA
TATTTTGCAATTATTAAAGTTTAAGTTGTCTGGAAACTtaatttattatgtatttttattctatg

TGTTTTTATAGTGGAAGgattcatttattatttgtttttatgaagctGTGTTGCATGTGCTGACC
TGTTGCTTGGTTATATTCCATGACCTGTGTAACGAAACTGAGAGAAAACTG
TTACGTGGTTTTAAGGAAGAATCTGTCGATAAAGATTCAGTCccctttaagaaaacaa
acaaaacatcctCCCAGGATGAGAGATCTGGAAAAATATTCCAGTCTAAATCAGCA
GAGGATTAGAAGCTCTGCAGTGAAAGCAACGCCCATGGTACTTTTCGTTTC
AAAGCTTACctttcctgtgtgagcatttatGATATCACTGAAATTAAAGGGAGAGGAcgga
aaaaaacaaatacaacttGATTTCAGTTCTTGAAGA
>TCONS_02679154
CACCGCCGTTGCCATGGCGGCGGCGGTCTGCTCGGAGCGGGCCGGGAC
GCCGCGCCCTGTGACCCCTCCCGGAGGCAGAggatggctgaggttggaaagcGCCTCC
GGAGGTCATGGAATCCGCCCTCCTGCTCCCCTAGAGCGCGCTGTCCGGCAC
TGTGTCCAGGGAAGGAGATTCCGTGGCCCTCTGCCGTCCGCCCCACACTG
AGGCCTGAAAGATTCCCCTTCCCAAGTGGCGCTGCGCGCACACTTCTGCTT
TGCGTCCTTCCAGTCAAAGCTGTGTGAccccctgggctgcctgtgACGGCCCGCTGT
TCGGCGTCCCCCAGCCTTCCACCGATGGCCAAAAGGCTCGAGAAAGCAGG
AGCGTGCCCTGCCTGGGAGGAAGCACCAGATAttgagaatgaaaagaaagtgaagcaTT
CAGCTTTCCGAAGCATTCAGCTCTGTGAATATACATGAATTAACCAGCACAG
TGCAGGTATAATGGAAGGGCTTCCTGGTCATTCTGGAGCATCAGCAGCTGT
ACCTGGATGTGAATTGCACTGTGATACAGATAAGTTTTGCATTAATACTATCC
ATTTTTCAATGTGTAATGTTTTCATATTCATACAAGTGAAGGGTTTATGTTTT
TGCTTGGTATGTGTAACAGACACATATAACAGTTGCCTTAAAAACCTGTTGT
TTGTCAAGAAAAATTAGTTGATCCGTTGATCTGAAGTCACTCATCTTGTTGA
ACCCATTGCAAAGGGGCTCAGACTGAGGAAGCAGCTTCTGCCTCTTGCTGT
ACTGTTAGACTTGCTAGGTAATTCTGGACACATTGCTTAACCTTCCCAAATC
TGAGTTTAATGAGCTGGATATGTACCACGCCATATGATGCTTATGCTCACGA
ATAAAGCACTTGAGCTCTGTGAATGAGAAGTGTTTCAGTTTGAGTTCTAAT
CCTCCGTTCATCAGTTGACAGAATGCTTCCACTGTGTTTCCTTATGTAGCGA
GGTGTTCTCTGCTCTGAGATAAACTTGCCTTTATTTAGGAGACCTGAAGgaaa
aacgaacaaacaaacaaatgatgCATTTGCTAATTTTTCTGAGATTAAACATTCAGTAGA
ACTCAACAAATATGTTTACTAGTTTAATTGGATTACATTAATATACATCCCCA
CAGCTTTTATTACATAAGCCCTTTTCTACCAGGGATTTAGTCTATTAGTTAAG
GAAACCTGAGGGATTAATCTGATAGCTGCAGTCAGAAATTACAGTGTAATT
AAAGTCACCCAGGAAACAGTGATACTGAAGTCTGCCAATCCCTGCAAAAC
AAACTTGTCAATTTTCTGCATCTAGGATAGCATATAAGGTGACACATTAAAG
GGGCACAAGTTCTGCACCAGAAATAGGCCTGTTAAGGAGGAACTTGTGTA
CTGCACAGTGTAGCCATCGCTGTTCTTGCTACCAACTACTGCTGTCTGTTTT
TGAATAGACTTTAAAGGTTTTGGTGTTTTATTAAAGGTCATGTACAGTGTGG
GAGAGGAAGTATAACACGGCTTGCACGGAAGTCTATTTTAAAGCTTACTTT
ACTAATTTAGTTGGACCGAGATGTTACTTCATGTCCAAAAGAAGATAGAAA
ATGCAATGGCTGTAAAGAAGAAAGttgcagagcactggagcagcctTGTTAGTAGCAG
GGAGAGACGTTTTGTCATGGTCTGTACTTGTCAGTGTCATTTGCTGCAAGC
AGGGTAATGGAGAGCATGCATCGGTGCATGAGTGGAGGAGGAGATCTGTT
GTCTTCATGAATGGCATCAAGGAACAGCACAGCATTCCAGAAATTCTGCAG
AGTGTCAGTACTTGTGATGTGTTTGAGCCATTCTAATAGCATAATATACGCA

GGAGAGATTTAATTTCACTATATCCAAAATACACAGCATTTGGTATTTCTTTT
GTTCTATGAGACATAAGTCTAATGATGCATATTCTAGTTAGTGCAACTTGCTA
TCAAGGTTGTAAAGAAATaagcaacaagaagaaaataacgtatgtatttttctgttgtcagaGTAGAC
TTGTATATATACTTCACAGCTTTGTCTTCTTGCTCTAAGTTGTAAAGTAACCT
TCACATAACATTGTGTAAATGGAAAAGTTCTCTGCCAGAAAGCTAAAGCCC
AATATCTGTTGCTTGCAAAGCCACAGAGGGAACTACCCATTGTTTCCTAGG
AGGAGATGGTGCAGCGACTTGCTGTGGCTTTAGCAAGAGGGCAAGGCTTT
GTGATAGAAGATAAGCAAAGTATGATGACTTAAGTGGAGTGCTGAAGCTTT
TACTGCATCTGTCTTGTACAGTGTTGAATAGATTTTTAGGAACTTGCCAGTC
AGGAGACCTGCCTTCGTGCTAATGTGCAGACATAGAGAAACTGCATGAAC
AGAGGCAGGAAGCACTCagaacagaaagggaaaatgagcTACTGATGCACACAGAGC
TTTGCTTGGTATAGCAGGCAAAGGAGGATCCAGCAGGAACAGAAGATATCT
CTTCTGTGCCTGATCAGCCTGAGAAAAGTCCTTAACAAAACAAAGTGGCA
GCCTGGAGAGtataaaacacaggaaatgaTGCATTTCCAGTCCcacttttgcttctttctcagcAGT
CGCAGGGTGGGTGAGTAGGAGTTGCCtgtgccagctctgtgcagcctggcaAAACTGGG
GCCAGACCCGACGTTGTTGAGGCTGCTTCCACTTTTGTTTGTGAGGTGGAG
GGAAGGACACCCTCTGTAAGTGTTGGTATTAATGCTTTTCTCTTAGttaatctgtttt
gtttgctgctgaggCATTTTACCTTCACCtctaaattatttcattcttcatcaaaatccaaagaaaacagagattc
AAAGAAAGCACCCCTTAGAATAGACTGAACGCTGAGTATTATTTGTAAGAT
GTATGAAACAGGAAACGGGAAGTTAGAAACATTCAGGGAGTCGGTATGCA
TCCAAGTAGTCAATGGTTCAGGGGGCAGATAAACCAACACACCCCCACCC
AGCACTGCATGGCTACACCACCAGCATTCAGGAATTCTGACCTTTGCTAAA
TCTTTAATATCTTTGGTTATCAAACCCAAACTCGCTCACATGCCAGAAGCAG
TGTGGTACTGGGTAACCATGTAGGTAATTTTCATGCTCAGAGAAGACAGCT
CATTTGTCAAACTGCGTCAGGTGAGAGTGCTCAGCAGGCCTgaatttctgcagagaa
aaaggacCGGATTCTCCTCCCTGTTGGCACCACCCAGTCTGAGTCATACAGGCT
GGAAGCAGTAGTACAGCTGCCAAACTTCCTCACCTAGATCACGCTATCTGC
TATAGGCTGCACTGGGATTATTGAGTGAGGTGTTTGGGTGTAGAATTAAAAT
ACGTTGAGCACAGGATTGTACTGTTGGGGATTTTTCCACAATGTTTCTTCTC
caggaaatgtttttcctctctaGCATTGCAGGGTAACAGTGTAAacgtttgtttttgttggttcaTTGCC
AGACAAACGTAAGAACATGTTGTAAAAGTCACATTTTGCTGGATTTCGTTTT
AGAATAGTAGAACTGTAGAATGGCTTAAGTCAgagggaaccttaaagatcatctagcttcaac
tcctctgccatgggctggctgcccccaccagctcagggtgcccagggcccatccaacccggccttgggcacctccagga
atggggcacccacagctctgggcagcagtgccagggcctcaccatcccctaagcaaagaatttctttgtaacatctaaccta
aatctcccctcttttagtttaaattcCGTGTAGAATTTCAAGTGTGTTTGGCACCTGCCATTC
CAAATACAGACAAAGTTATATGATAGAACTCAGACTTTGTAAATCGTAATGG
ATTTTGATCACAGGATATGATGCTACTTGGTCACAACAGCCCAGATCCCTGT
AGGACACATACTTCCATTGATTTCACTGGAAGTTAGGGGCCACAGAGAACT
CTGTGGTTCTTTTCCTACCAGCTGAAGTCAATGAAACCACCTTTTGAAAAT
GGGGTTCCAGATATTACAGTCTAGCAGACAAAGCTGGCTTATCCCACATTAA
ATGTCACCTCTGAGAACGGAATTTAAGTGATGATGGCGTAGTGGAAGTCTaa
gacagagccaaggccaGAGTGTAAATTATCTGATGCCCAGCCCAATGCATGAACCA
CAGTGCTGATGTTTGATTACTGTGAGGTTGGATACTGACTCTATAAACAACA
ACTGCAGTTTTCAAAACTTACCCGCAGCAAATGCAGAACCCTGCCTTGACA

TGGTTTTCTGATGGTAGAACTCTGCTTGCTGAAGTCTATTTACACCTCAAAT
TTTCCAAAAACAAGACTTCAATTCCTCATTGCTGTGAAGCAAGCATTACTTT
TAACAAATTACTTAATTGCTTTTCTagccatttcttttcctgcatttcttAATTGAGTTTGTAT
AGAATATTTCTACATGAATGAGTGCCTGAGGCAAGATGAGGTCTGAGCAGA
AACCTGTATCTTGGTTTCCTAGTCAGTGGGATGCAGGAGAGCCTACTGTGC
TTCTGTTTAGGGATAAGAACTTGGTTAAAGGCATGCAACCCAACTCATGTTT
CCTTGTGCTTGCTTACAAGGAGAATATCAAGGTCTCAGACTGGATAATGATG
CAGAAATGCACACTGACAGCGGTCCCAATAGCCGGGCTCATGGAAAGAAA
GTCTCAGATGTGAATGAAGGATGTGAGGCTGCAGCCAAATTGATTAGAAGC
AAAGAACTCTCAGACATCTGAAGTGTGCAAAGCATAAAGCTGTCCTCTAAT
TTCCCAGTTTTGGCAGTTTTGTTGCCACTTGAAGGTAAAGCTGGTATTAATC
ACTGGGGGGAAAATGCAACCCTGCTCTCTTTGATCTTTCCTCCTTTAATCAA
CAGTTTCTAAGTGCAATAAGACATTTCAGATGTTGCCAATGTATTTTTATCTG
GCAATTGTTCAATGCGTATCCTAAAATGGAGAGGTGCatctgctgaaacacagcatggT
GCTTCTGGAGGAGGAAACCATCTCCTTTCTGGCTTTGCACTGTTCATTGGT
CTGACAGCCTTGGATATCCATGGAAATGTAACTGTTCCTAAGATGAATCCTA
CACGTTGCACAGTTGCTCTGCTTCCTGTAACTCATCTAAATATTGGTGACTA
ATTATTCTTGAGGAAACATCCTTTCTCTGTGGAGTTAGCTTTGCAAGCAAAA
GGACATTAGAAGCAGCACCCTGACCAGGTCACTAGCCAGCCAGGAGAGAG
CAGGGATTGCAGGCAGGAGGTGGCAGCCTCGAGTAGCAGCacactgctttctctgctc
aGCTATGCTCAGACCTGAATGCTAACAGGAGCAGGGTACAAACAGCTTGGG
GTCTGGGTCCTTGCTTTAAGCACTGCAGCTTGAAGATGGAAACCTAACTTT
GTCCCTCGTGCCCACTAATTAAAAACGTAGCTAAGCTGCTCTATAGGTGCA
GTGTTGGAGTTGTATGGGTTTGCtcagaatttttaatattttgttcaaTATCTTCAGAGGGAA
AGAATGTAGCACTCTTTATTTGATCTGTCCCGTTCTCATCTAggtttatttcttcttgcaa
GGAAAATTATGTTGCAGACAAGAATAAGTCCCCTCGAAAtactgtattgttttgtttgtgt
gtctAATTCTTGAGCCCCATCCTGCAAAGCTGATGGTCCTCCATGGCCTGGCT
ATAGTCCACAAGGGGATTTATAAGGGAATTTTGCCTCCCTCATGAGCTACAA
CGAAGTAACGGCAAATGAACCTACTTAGATTAAAGATGTCACTGACTGAAA
TGTTCTAATCAACAGAACCTTGCAATTATCCTCTCTCAGCAGAGAGGGGAA
CTTGATGACATCattgtttttccctgtgaaaacGCTTAGttggaaaaagacaaaaaaagattcAATGAA
TCACATGCAACATCCCACTTGGAATCTTTGAATAATCCCAAAGAGTGCCATT
CACTGCAACAATCAAAATGTAAGCCAAGAGAGTTCTGCAGCATTGCTTTTT
GCCCACTGGGTGAGTATTCGTGGTAGGAATTTTCCTCGTTCATTTTATTGTA
AAATCTGTGTATCCCTGACTGTTTTTTCCTGGAGTCCCACATTTTCATCTTCT
GGACCAAATCttagtttactttttttttcctttcttccttctgatgCACATGAAaactctttctttaaaagctgaCA
TGTAGCAAATGTTACATCTGACATCCTGCAAATGGAATCCTTCGGTTAAACT
GTGTGTGCACAAATCTGTTCCCCACAGAGCCAATGGTCTACCTGGATGGAT
ATGAACGCAAGACTGGGTTTTCTGTAACAGTCCAATCCTGTTAGACACAGA
AGACATTGTCCTGAATGGTTTTGTTTAATCTTTTCATTAAGCAAACATTTTCC
TAAGTTTGCATAAGCAGTCACCCAGCTGCGACTTCCACACAGCCTCAGTAA
CTCGGAGGGTGTGGTGATTAAATTTCCTCGGAGACTTTCCTTCCATCTGACT
ACCAAAGAGTAATTCATTCCTCAGCCAACAtcgtttgtttgctttgtttcttcagcCTTCATCT
GCAGAAGATACATCGGTGAGGTAATAGTGGTCTTTCTAATGCAAAAGAGTG

AGGGACGATTTGTTGAGCCACATTCCCTGAAGAAAGAAGGCTTATGTGCAC
AGATTCTGTCTGGCCCAACAGCCAGCACTAGAACATGGAATCCCATTGGGC
AATTCACTTCAgtatgaaaaagagaagactccagTTTTCTGTCAGGACTGTCAGCTGAA
GGGCTGAGCCTAACAGCTGTTCTGTGTAAGAGTTTGAACtcaactgtttcattttctctcc
ttcacagCCGTATTACTGGAAGAGGAGAATGTGCCAAAAGGTGTTTTGCTCAAT
GAAAAAGTGCAGTAAATTTGAGCCGTATCAGGTCTGTGCCGCTGGAGAaatat
gctttcctttttaaagtgTATGGAATCCTACaacttcctttctgtttcttggtTTTCTTAACATAACAGT
GGCAGCAGCATTGGCTTCTGTAGAATGTCAATAGAAGTTATGCTTCAGATCC
AGATGTGCAGAGGAAGAAGACTTTCCTTCCACAGCTCTGTGGGTCCTTCTA
GCTGTTACATACCGACCTGTCACAGAGGGGGTTCTGTGAGTCCTCCCTAAT
TCATATTAAGCACAATTGGGCAGATTTATTAAGCAGTTTAAAATCCTGTTCT
GACATACGGCATCTGGTTTTGCATCACTTGAATTAGCAATGCTCCCACAAGC
TCTTCTCTTAGCACATCTGAAGAGTGTTAACCCTATCCACTGGCACTGCtagat
ggaaaagaaatcatcTGTATGTGACCTGAAGCCCTGTTGCCAATGCACAGGCATATA
GCATTGTGGACGTGGCTACAGCCTGTACAGAAGAGAATATAATTCTATTTGG
AGGGCTTGACTATTCAAGCTCTgtgaacaggaagaaaagggctG
>TCONS_02679804
ggcagccTTTTCTCCGGATAAAATATGTGAGGGATGACAAGAATCTTCCCAGCA
TGGGAAGAggcaataacaaaaaaaaagtacaagggGCTGAAGCTGAGTGTAACATCCACT
GCAGAGTGTGCCTGCAGGCGACACTCAGATTTGAAAGaaatctCTGTCAAGG
GAAAGGGTAGAATTCACACACCTCTGCCTTCACACTACAACTACTGGAGGA
TATGATTAActctgagcagctgaagaaCTCAGATCTTTGGGGAAACCCTGCTGCCAGT
GACGTCAACAAGGGAGGTGTCAATGACTTGAGCAAATCAAGGAATTCCAG
TCCCACCAGGCTCATTATGTGCCATCCACACTTGCAAGAAGAAGACCCTTC
TACCAATCCCGACTTGCCCTGGTGCTGGGAAAGTTGTACAAACTGTAAAatg
ctggggaaaaggaaggt
>TCONS_02703088
cacttactcctggggtctgagtgagcatctggccccgacctggtaaagggtcggtttcgcccagcagtaagccctacatgtg
gacagaggacgaacaccggaCGAGCGAACGGAGACTACATGCAACAATAATACACCA
TCTACAGAATGAATCCATGTCCTCAGTACCTTAACTGACTGTCCTTTGAAGT
CTGAGACTTCTGTAAGTTACTGAAGTATTAGTATTGTTAGTTGCTGCATGTTA
GTTTGCAACTTCTGTTGTGAGTCATGCAGTAATGTTTTCCAGTGATTGAGTcc
actgttcttttcttcctctgacttTTACAGAAAACCGAGGACACTTAcagaaaagtttttattttctagatCA
TATCACATATAAATTCCTTAGAgttgtgaggtttttgttacattttgaaGAGAATTATCCCTGC
ATCAATTTCACCACATCCTATTCAGTTTATTTCCCCCAGTGTAACATAGAAGT
CctgtaaataatatttaaataatattttaattttttaacaggAACTCTGTTATAACCTCTTTTAGAAATG
TTAAAACAATGTTAATACTgtatattttctggaaaaatatgGAATGTTTGATATgtactaatttttatt
ttctatccaAGCCATCTTTAAATAGTCATTCATCTCTTCATCCCTAGAGTAAACAAT
TAAGCAAGGGTCTCAAACATACAAAGATGTATTTAGTATGATTTTTTGGATT
GGCTTTATATGAACAGCATTTTAATTCTGTGGCCCTTACGGTACTAATCCCTT
TCATAAGAATGATCTGCTTAGCTTTCTTTAATGATAACACTATCCttggtttgttgttgt
ggtggtagtgtttttttttatttgtggttttgtttgtttgtttgttcatttattttgtatttgtttttgttttgtaattattCAGCCAGGA
CTAGAAAAGTGGATTTCAGAATTCCAGTTCTGTGTGAAGCtcttctgtgactttttttttctt
ttggaaaatgatACAAATTTTTGTGAATAAGTAGGTCTTAGATTAGGTTCAGAAGT

ACAAGTATATATTCATATAcgtgtatatataaaaatcctATGGCAACATTGCTACTGGAAC
ATTCTAGACAATTGCTGCCCAAATAAGATCTCCAAGTTGGAAAGGGGACAG
TGAAGGTTTCAGTCTTGGTATTTAGTACTGGTGATTGCAGAGGTcgttttcatttcttc
tttaaatgagTTCCAGGTACACAGGCAAAATTCTGAACTGAATTTTTACCCCACA
AAGATTCATCATTGCActttaaaattctatttttcctAAACCACTGCCTGGAAAAGTGACA
TCATTCTGTGGCTGGCAATCAATGAAATAACCTGAGGCCATGCTAACAGAG
CTTTGAAGATTAAAAGTGTAACATTGAATTTGATGTAAATGTGAGAGGGGA
GACAGTTTAGCATACAGAATACTGTCACaccaaagctttaaaaattgtCATATGTATGGA
AGAGGTATCGCTAATGCCCAGAATAAAGGATTTGTATTGCTACTGCAGAAA
CCTAGCAAGAGTTGTCCAATACGAGACTCTAAAAATCTACATCTAAGTCAG
CATAGGAAAGTTCCTCTGTCTCCTTCATCTTGCATGAAGCACAGTGAATTAG
TAGGAGTGAAGTAAAGCATACTAGGTAGGTTTTCATATAATAACCATTGCCA
CGAAGAGGACATTCTATGTTATAATCCAAAAATGTGCCTGCAAAATTTAGTA
GAGGTAAttaatacagaacaaaaaatggacatacaagcaaaaacaaaaaaacaaaattctggCAGATGTGA
GTAGAAGTTCgtttgatattttcttttagtcTTGATCAAAATTTTAGCCTCCAAAAATCTTT
CTGACCATGCTAAACAGTGATTTCCTGTAGGAGATTACATGGTGCATGGTGC
ATATGGAACGTGAAACTAAACGGTAAGAATTCCCAGTGAAATTGAACACAG
TCCTCCTAAGAAATTCAAGTTTCTgtcaaaggaaagcaaatagcTATTTACAGTTCATA
ATTTTCCTACTGTTAATATCACGACCTTGTTATCaagccaaaataaaatcaacaaatGTGG
TGGTGAACagtatttaaatgcaaattattttcatggatTACATCCTTTATTCTTTTCAGCTATCTC
TGCATAATTAATTTACAATACCAGTTTTCAGTAAAAGATACTATGATGTATTG
CACTTTATCgacatttgttttccctttctattCAGCAATATTTGTAAAACTGCcttctttctattaaaaat
atttataaaattgccttaaaagacaattttatttattactatttagTTGCAAAAAGTAACTAAAAATAGTT
GGCTCTTCTGTGAGGTACATCAGCAACTATAGGCACTTACAGTTCTTCACTG
CAGACTATTTGAGTGCAATGGATTATAGCATTAAAATTAATACTATTATTTCA
AAAGTTAAATACACTTTTCTAAAGTTCTCTTTATGAACTAATATTGTAGCTCT
AgtaaaataagttgtttttctcagaaataaaattttaaggaCAAAGAAATGGATTCTTTTGAATGCTG
AATGTTGTTCAGCATGTGTTTTTTGTGGATGCTTAACACCTTTGCAAGCGAT
GCTAGTACCTCATCAGATCAGTCCATTGCTGCAGTAAGCTCTTGGTGTTTCT
GCCATACAGGCAATAGAAATATGTGATAAAGTGCTTAGATGAAATACAGCA
ACTTAGTCAGCTGCTAATACTGGCAGTGGTGAGTCAATTTTAAGTCATAGCT
GAAAGGATTCTAGTACTAAAATTGAAAAAGTTTTGGATCAATATAGGTAACT
AAGAACACAGTAGacttttcagagaaacagaCCTCCCAATTTTAAGTCTATGAAAAGT
AAAGGAATTGGATTGCTAAAATTATTCTGTTTAGTTGGCCATACAAACTCTT
CCAGTGTTCTAAAAACTGTTCACCCTCTTCAGAAAAGAtgtgcaaagcagcactggct
agcatttttattatcattatatttgttttctttatgcttGACTGCTCTATACAATGCCATTCTCTATAATA
CTCAGCAATCTAGCttgcaattttaaaaacatcattcgcaataaataaatgtatctgTCAGGTctcaaagaga
aatacaaatgtgctgtgttttctatGAGGGAAATATGCAATTGAAAATCTCCCCAAAGTAAT
TTTCTCCTAGGTCCATCATATACAGTTATTTAATAGGCTTTTTGGTTCAGACG
ACTATAGCTATGACTTGGAATTTTGTACTACAAGTTCTACATAGCATGTGtagg
aaataaatttaatagAATAATGATATGAACTTTTTATCAAAAACATAATATGTTTCACA
TATTTTCATAATACATTTTCAcatgttatttcatttctttccaatGTAACTTCCAAGTCTGCTT
GACCTAAATATGCAGAACCAGCATCAAGGTGCCTAGTGTATAATCATATACA
TGCCTAGATGTATATGATTATAACTGAGCCGTTCTTTCTGTTGCCAAACaattga

aataagaaaaacaagcagataaTACTAATAATAAACCTTAATTTTGGATCCACAGTTTATT
AAATCCATAGTATTTCTTTCCTCTAAATAGGAGCTCAAATCAAATAGTAAACT
AGAAGTTACTGGATCCACTtgaatggaaacaaacaaacagcaaaaaccaCAGATGGACACT
GTAGCGCCCCCTCATAATGCTGAATACAGACATCTTTCTTCACAGTTTTCAT
CAAGAAAGATGTGTTCTTTGTTTCAGAGCTGATGGAAATGACACAGTCTTC
TGTATGCATGTGTTCAGGATTTTCCACTgttctgctgcactgagcaaGGAGAATTTGGA
CCCATGAAACAGTAAAGGGATTTGACATACTAAACCTTTTAGTCACATTAGA
AACTGCAAAGCTCTTAAAAATCCATGACAGTGACCTTAATCCTTATCGAGA
ATCTGACTAGACTCAATGAATGGAGACACCACTGTGGCTCTTTGTGTCCATT
TTGTGCCGTCTCCCATTATCGGAGTGGTCTGTCACTGTAGTAACTGAACAA
GTACTTATGGACTTACTCTTGCAGGTATAAGTACTTTTGCATTGCAGAAGAG
GGTCTGAACCATAGATGGAGATAGATGTGAAGTAGTTAAACATTTAAGGAC
ATGCTTACAAAATGAAAGCCAAGCATGCATTTGAGAATTTTCCCCGACTTA
CATGATTTTCTAAGCTTCATACAGGTGGTGGTAGCAATGTTGAGTATTGAAC
CTCTAGGTCTCCTTTATGATAGTGGAATGCTTTAACCCTAGGTTCACCTTCTC
ATTTCGCAAGTATGTAGAAAAATCTTTATAGCACCAAATCTTTATAATAAAA
GCCATTGAATTGCTTGTCAGACAGTTCTTTTATAATCTTAGAGACAGAATTT
GATTGATAGTTGAAACCAAATCTAAAGTTATCTGGcaaagaaagatgaggaaaattCCA
TTATATGAAATAATGCCCTCTTCTtggccattttttctgctacaaGTATTATGCTCCCACTT
TGCAAATGGGAAAACCATATGCACCTTGCAGCATATGTCAAATGTACAGggttt
ctcattttcctctctcttttctaaaagaattgaactcaataaaataatacaaataaatacttatttttattattattaaacatcATAG
TAAAGATTAAATGCACTGTGATTCACATGGACTTGAAAAGCTACTTTTAACC
ATATTTGATCTAAGCAACCTTTTGAATAATGAATAGAAGACTTCAGTTTGGG
GAAGAGGGGTGTATTTTTCCTTGCTGACCACATTACATAGCAACAAAACGG
AAGTATTTTAAGGgtaatcattatttttcaaatcttttttgaAACTTCTTACCTGAAAATTAGTC
CGCTTTTAACAATAAACATCTcaaacctttaaaatacactgaaaaatattgaGGAGTTTTCACA
GATTACTGGAAAGTGACAAGTCACTTCACCACCAAAGATTAGATTAGTAGT
AACACAGTGTCAGAATATATTCAcaatacaatttattttatgtacatTCATCCATCAAATCTG
AACTGCAGCTGCTATAAGCAGAACAATTTTATTCAAACAGGAATCttattccaaag
aaaaatccatGAATTTAAGGAGCAGCTTTTCTTTATACCTGACTGTTTAAGAAAG
AGAACGTGTTCTCtagcttttcttaaaaaaaaaaaaaaaagaaagaaaaaaagaaaattagccGATTATTT
CCTTCCAAGATTAAAAATGTGTTAgcactaagaaaaataaatgaataaacagtATGTATCAAT
GTTATCTTGTTACTCCTATCATAACTCCAGtctcttctctgttcttccctgctgctgttttctttatcaT
GATTTTATACTGCagcacttttctgttttcagaaaaccaAATTCCCTTACATTCCCAGGTATG
CTATGAGACATTGCGGTTTGGTGGCCTGTTTTCTAGGaatatttttccacttctgatCTGT
TAAGGCAGGGTTAAAGACAATGGTCAGAATTTGTTGCaaacatattttgttctttgttgttaa
ACCAAACACACCAAATTAACATTAACTGGCTGTAAAGAATagtgaacaaaacaaaca
tataccctgataaataaatgaatattgaCAGATTAATCTTTTGAAGTTATGTGAATTTCAAGTT
GGTCTGCATTTAAATGATGACTCTTAGTTCACGAAATACTGGAGAAGTTCA
GATGCAAATAAGtgaaaaggatttaaaaattGTCTATTTCATGTGTCAGCCTTACACTTG
AGAAACTTAAGATAGCTTTAGTATCTGCCTGTATTTGTGTGTCAAAATTATGT
AGTGACTACAGATGTTCATCTTTGCAAAGTTTTCCTTTGCTAATGAACTTTC
TTATCATGCTTTTCCTGTAAGACCAGCTGTAAATTTGGCAGAGACATATATTT
ACAGCCCTGAAGAGTTTATTGGCAgctagcttttgtttttactacTTGATCTCTCTTAGA

CAGTAAAcattcttctgtcttctttccttctgtgatGATATATGTGATACAGACACCTGTTTTGT
AAATTCAGATAAGTATTGTATCTGGTTTGTATATATGTAGTTCTCTGCAATTG
CTCTTACTAGCTAAAAAATTATCATTAACAAAATTATCTCTTACTCTGACTGC
TTTTTTCTTGAACAAACTCACCATGCCTTGCCTTAAATGTTAACACTGGAAA
TGTTGAACTGATTCAGAGGAGTTTGGCAGAGATGCCTGGAAGCCAAATTTC
TTTCTgcatccttccttttctgttcccttGTAGTAGAAATAGCAGTGAAGAGGAGGGCCCA
AATGCATTTCATTGCATCTCAGTGGGACACTGGAGGTGGCCTAATGATGGA
GGAACCCACCAAGATTTACTATTACAAAGCCACAGAGAATTACAATCCCTC
ATCACTGAACTGTATTTTCTATGGCTGGCACAAAGCAATTCAACAACCCTG
ACATCCAGCCAAAATCTCTATAACAGCATCAGACAAAGCTAATTAAAAATTT
CAAATCTATGTTTTTGTATATAACAATCATATTACTTAATATTATAGGGGGTCA
TCATATTTCTAGTGCTTTAGCTGagatattttccatttctcccaGAGATTACCCAAGGAGA
TAATCTGaatgaaataatgtatttttggTTCAACAgttatgattaaaaaaagaaatcttagtTATTATGAAA
TGTCATTGTAAATATGACTTCTAAATCAACATATGCACTGGAAATTGTACAGT
AGAAAGCATATTCTATGCCATAAAAATTGGTTTTGAATAATTGGATACTCATT
ATCATAGTACATTATGGACAATgcattttctcaaaaaaatcCTTCTCAGTCTGCTCATCTT
ACAAGAAACTGATGCTATCCACATCTGGCATTATATAACTACTGTATATTGAA
GCATGTAGAGGCAGTAAGTATTCTGTATTCAGTTCAGAAAGGTATAGTTGTT
TTATCTTGAGGCAAAAAAGTATGTCTCTTGAATTGGTTTTGTAAAAATTTTC
ACTGAAACTAAAATGACATTGTAAGCATTATTGCtctgaaacaatttttcatgtcataattcatat
atatatattgtaaaatagagaaatatttaGATTCATGTAGTGAAACAGGTTTATACATTCATATC
CGAACTTGACAGttcaaataagaaaatgatttgGTCATGAAATACCCAGTCTTCGTGAGT
CTTTTGGACAGTTTCAGTGTCAGAACTTTATTTTCTACAGGTTTATATTAAAT
CAGCTTAAGAAGAGGCAAAACAACACAAACGAAGTTATAGAGCTTCAGGT
AGTAAAACACCTGCTCCCTAGGATGCTAGTCACTTATGATTATAGAAATATC
CTAAGGGTCAGCAAAATACTTGGCCTTTATGTAGCTGGAATCCACATtaatgtatt
tcagtttctgtctACATTTTGCATGATTTATATTCTAAATAAATCAGTTTCCACCTATTT
TTGAGCATTTTGAATTGTTGTATCTAATGCCAGCATAAATCCATTACATAATG
AGAATGTGAAATATTCTTTTATACACTAGTACAACAGTAGCATGTCCCTGAT
AGCATTTATCAACTGACACTGCTTTATATACAGCAATGATGAAATACTGTTGT
TAACTCAGGTAACTCCTGGTTTTCCAGTGCAGCTGATGTAGAATCTGTTGA
AACATAACCATACTAGTTTTGTGAATAtgagaaaacacatttataaTCCCACAGGCAAT
GCTACAAGAAAAGTTACTGTGAGTAAGGTAAGTTGTGAACCTGTATCAGTC
TGCAAAGTAATTACTTTGCTGGCTCATTTTCTCACAGCCAGTGTGAGGTACA
TCACTCCCTTGGCTCCCTTTGCCTGATGGGTATTCTACTGCACCTTTGCCTT
GGAGTAAAAGAATGCATACAAAAGAGAGTAGCTGGCATGCTCTCAATCTAT
GCTCATCACACTATGATCTGTGAGGACAGATAGACTTCGAAGTTAATAGAA
GTGTCCCTGCTTTGCCAGGTATCATTTCAGTACAAAGGTTAATACTACTTTTT
TAGCTGAATAGATCCTGGTAGAACCTTTGGTAAATTTCTGCCACAGTGTATA
ATTTATACAATATAGAATTTGCTCAAGATATTATGCTattcctgcctgccttcctaTCAAT
GAGGTTCTCAGGCGATGTGATTGACCCACATATGATGATGCAAAAGGGACA
TGATTGCTCCTGAAGCCAACCTGCTGGtggagagggctgcagggagccaaACTGGCTAT
GTTTTTGGTAACATATTCCTCAAATCTATGTCCCAGGTTTAACAACCATACggt
ataattttttcctatataagACTATATTAAGTTATTCAGAACTAAGTTGATTTCTGTCACA

AAttatttaactgaaaaagagggaaaatctGCCCAGACTTAGCTGACCTATGAAGACATAGT
TCAAATTGAACTCAAATTTCCCCTTAAACAAAGTCAGTGAAAAAATACCTC
TTGACACCTGCGAAATCTAGATTTCAGAGTGGTAGATTTCAATTTAGTATGC
AAACTAAAATTTAAAGCaactttccttttgtctttggtgtattttcccttttcctcctggttttgtatttttgtctgtC
ACTATGGTATGACTTTGGGAACTTCTCTCATGTGTTTAAGACAAGTCTTTCT
GGGTTTGCATACACGTCATAGTAATACACTTCATATATCATCCTTTCCTAGCC
ACAGGAAGAAGCAACTCTGCTCCGGAATTCAGTCAAATCTCGTCTAGATAA
CAAGTCTTCCAGTTCACCTCATCCCCTGTCTTCTGGCAAACATGACAAAAA
TGCTGAGATccaagagcacagaaaaatattagcAGAACTGGAGGGAtgtcaaagggaaaaagaattc
TCACATGTTTAGTCTAGGAATGATACTTTTTTATATTAGAAATGAACTCAGAa
aatataggaaagaaaatagaaaaacaaaggacTGTACACACATtgtaaaagaaaagttttacAAATAGAA
CTGAGTTAGAATATTACTTTTTTCACTTATATAATCTTCCATTAACACAAGTG
TAACAGAAATTTAACCCCAGTGTAGTGTTCAAATTCCTGACACAACTGGGA
TACCTTTATGagtgaaaaacaattttaagttttatttctcttagaGTATTTCTCACTCATACCTGTTA
TAAAGCCTCTACCTTTTCAGTAGATTTCTGTGGGCTTGACCATTGTCAgatttttt
gtttacttttgtttttacatttagTTTACTAGATTATCAGTGTAGATATTTTCTTGGGTAATTTT
AAGTTctattatttacttatttacacTTTTATACAGTAATTCAGTGCAcagttggaaaataaataaccaa
aATTAATAACCTCCCAGAAGGGCAATATACtttaaatatatctgaaaatCTACtattacacatttta
aaattattttttctgcaagtACTCAGAGAACTGGAATAAACAacaccagaagaaaataatgacagAGT
AAATCAACGAAGGTCCAAGTTTTATGATCCATGTGCTATCTAAAGTGCATCA
GTTTGAAACTTCTGTGCAAGATTCCTTCAGAAGAGCCTAGCGTGCTGTTTT
TAATCTTGAATTGAAAGAATGCATcagaacagaataaagaaaattgtTGGGTACTCCTCT
AGAAGTACAAGGTTTGAAGAGTTGTATGAATATCCACTTAAATTAGTCTCTG
AAATGTAATGTAAGGGACTTCTTACCAAGAAAACGTAAGTTATATCTCCATT
TATTCTGTAGAATATTTTATAGCTTTGTATCTTCTCTAAATAGAGTATAGAAAG
ATTGTTTACCTGAAAAATGTTATGCAATATTTCAAAGCACAAAGTCTAACAG
TATTAATTATAAATGTCATCTTAAATTAATAATAGGGCTAACAATAATGGTAA
GCAGACCAAGTATATAATACGCTTTCATATTTCAATAGAGTATAATGTGTCAG
CACACAGGTTATAACCTTTCTTAGTGAAATTGAAAAAGGTTATATGTTATGA
AACTAATGTAATTGTACCATAATTTTAGTATAACTACAGTACCTCTGTTTAGA
GAATAATTTAAGTGATGGTTTGTTTGCAAATTATGAAAAATCCAGCCCTCTT
CTTATTTCATTCTAATTTACTATAGAAAAGACTTCATGTGTAAGAACTATTCA
AATGCAGATGggatttttcttcatctcatactcatcttttttttccagtgactaATAGTAAACAGCGTAGC
ACAAAATATAACTCATTATGCTTGATTTCCTATACAagtttgtctttaatttttcaCTGCAT
GACTGGTTGGATAGTGCAAATTATAACTTGCAGCAGAGTATTTGGAGGGAA
TAATATTTGCCTTAGGTACAGTGTGGTCACATTGTATTTCCAAGGCAGAATG
CAAATACCTGCCCCAACAGAGTTCAGTCATCGCCAGGAGCTATTTATTGTAT
AGGTCAGAGCTTAGAAGTTctccatttttaaaagatactgATCCTTCAATATACTTAAAGT
ACTGAAATTCTATTCTGAGGGTTTGTGTTCTTTATCATAAGAGAAAATTAATA
TGTTTAACGTCTAAGTATAGGAATTCATATTTATCTAGTTACTTGAAAACATG
AAGCAAGGTCTGATTAATTAAGTTGAAAAGctccatacagaaaaaatgttacttAGCTGTC
TGCACCATTATTTTTTATGTCTTCCTGTCAACTTTCTCTTGTAAGCTCCTGTC
AGAGACTAGTTATTGTGTCAAGCAGATGGATCAGTGGCCTTCCACGTTGTA
ATGTCAGTAATAATTTAAGGGTAGTATTCCTTTCATATAACACTAATGAATAG

AGGTAGGTTTTAAACCTAAGCTAACAGTCTGTTACTTCTGTAGACCCTGAA
GCTAGGGCCTTCTAAAGGCAATTTACAAATTCTCAAATGGCAATTCATCACC
ACTGTATTTGATATTTAATTTGGGATAGACAGAAACACCATGTGGAAGAGCC
TGTTTCTTCCTGGTCTACATAGAAAGCTAAGACGAGACGTTTCATTTTTGAT
GGCCAAATTTACATAAAAACAATCTCAGATTTAAGCAATAACTAGGTATAAA
TCTATCACTTACAGAAGATAGTGCTTTATAGCAATGAATACTGTTTTCCTAAG
TTATGTTGGGTGCAAACAAGATCCAGAAGTTCTGAGACCCTTCTCACATAT
GTTCTCACATAACATCTAACATCTATGTTACATACAAGTCAGTAATGTCTCTT
GTTCATATTTTTGAATTACCCTAGTTATCCGCAACGCAGAAGGGAATCTAGT
GAgattaagaataataataataaaaaaagtaaatatagtAATTTGATAAAACCAGTGAGGACACT
ACTAGTGTCTTCAGAGTCTTAACTATCTTTATGCAGTGATTTTCAGATGAAca
aaaacatgaggaaaaaaatccacatctAAACTATTGGTGAAGTTGTTTGATTTTGAAAGGT
CTGAAAGAACACTAATCTGTCCTCCTATGTGTCCACAGATCTTTGTACTCCC
TAAAATAATGGGAACAGTGAACATCTGGAAGCTATTTACTAGGAGAATTTG
GAGAGTTTATTGTTATCAATTCATTCAGGATTCATTGCAGAGTCACTAATAAA
TCTCTTTAGTATTCTTATGGATCATCCTTAAGATTCAATTGGTCTTATGGATCC
ACCTTGCAGTTCAATTGCAGtacatttgtttaaataacaaaataacaccAGTATGTACTCAGAT
ATGTCTCTAGACAATAAatccatttgcttttttttttttttttttttttttttttttttttttttttgaatgttgGTTGGGATC
TTTGTTATATTTTAGGAGTGTTTATCCGCAGCTGTTTTTCAAACCTGAGCAAT
TTCCAccaaaaaagtaatttttacaCCAAATACATTGTCCATAAACCTTTAATAGATTCTT
TAAAAGATGCTTTATAGATTCCCGTCCTCttagccacttttttttttttttcataatcttggagaagaaa
atcacagaatcatggtATTGTTAGGATTGTAAGGGATCTTAAAAATTGTCTAGTTCCA
ATACCCATGACATGAGTAGGCAAGAGAGTACAGGTGAGGTTCTTCTGATTC
CTTCAGCCTTTGAAATGACTTTTTAGTTTGGTTTCACTACAAGAATATAAAG
TTATCAGACTAAgctaattttttcattattttgctaCATGAATGGAGGTACACCTCACACAC
TAACCACTAATCATCTAATCTTCTAAGCTATTTTTGTGAGTTGCTTACAACCA
TGTCCCAGTGATGTTCCCACACCTCATCCATTAGCATATTGTCACTCTTCTAC
CTGGTTCAGGGAATGTTCCAAGTGCCCATAGAGTTCCCTCAAAATCGTATTT
AATAGACAGTGAATTAAAAGAATAGTgtccaatttatttttaatgtcaagTTACTTAAAAAA
TAGCACTTTACTCACTCTGGTGAAGTAACCTGGGTGTTTTTTAATTGAGAAG
CTTGACATTTTGCATAATATTAAGTGCTCCGCTATTCACCACTGTATGTTCTA
CTTTTGGGAAACACATCCAGTATCATGTACGTTCCCATGGAGAATCTGCAGC
AAACTGTCAATGAAATGTTAAAACTGGTTAGCAGCAATAACAAAGCCTTTA
AAAGGCtctgtttctggaaaaaaaaaaaaaaaaaaaacatcaggaaacaggaaaggaattttCTACCCCATTT
TAGAATGAGTCACTGTGCTCATATATTTGTAATGTAACTATTTCTGTTATTTAC
ACTCTGTACACATACACTAAGAAGTACTGAGAGTCAGAATAGTGGTTCATT
TGTCTTTACAAATGTCAAGCCAAATAGTGGTCTAACCGATGTCTAAGACAG
CAACAACATCCacaacaaaacaattaaaataaaatgatgcaaAATTTTTAACCACATCAATGG
CTTACTGTCACAATAGGGACTGAGTCCCAAAGACTAGAATCCTCTTTTAATA
GTCCAACTTCCAGGAAACTAGCTGGACAGACAGATAACCAGAGTATTCAA
GACTGTTACAATAACATATATTTGATGAGATAAATGACCTTCAAGATAGTTGT
GCTTTTATATAGATATCAACACTTAcaattttattagaaaatagCCCATAGCCCCTTTTAC
TTTGTTACCTGCTTGCTCTGTTTAAAAGGCCCTGAGAAATTATTACAGCGTC
TGAAGATGATGCTCTATATTTTCAGCCTAGTGAGTTATTCAGCCACTAAATCC

AAAAAATAAGTGGACTGTCAGATTTGTGAAAGATCCTCTAACATGAGGAAT
AGTCTGTAGCTATCCAATTATCTGCTCCCTTGGTTGCCTAACTGATCTATGAA
ATCAAATTAAATACCAATCTGCAGATTTAATATTCATATTGCAGTGCTAAAAA
ATTTGAACAAAATCTACTGAAACAATGAGGAATAGCTGAGTCCTTTGCAAA
TGAAAGCCAGGAGACCATGGGTGGTTAAAATCTGGACTTAGTTATACCATT
TTCTACAGCTGGGCATTATTAACCTGTATTCTTATTTGCTCTACAAAAATCAC
AACCCTGTTCACTTTATTTATCTATTCCATCTGCaacaattaaaaagtaattgaGCATAT
GTCGTGGTTATGGCTGTcatcaaattaattttcttcatagaggCTTCTATGATGCCATGTTTTG
GATTTCTGATGAAAGTAGTGGTGATAACACAACACAGTTTAGCTGTTGCA
GAGCAGTACTTACAAAGAgccaaggacttttctgcttGTCCTGCAGTCCTATCAGCAAG
GAGGCTGGAGGTGCAGAATATGCTAGGAGAGGACATAGCTGAGACAGCTG
ACTCAAGCTGACTGAGCTTGAGGAGAATGCTGACAGCTTGGAGAGAATGA
GCAAGTGGCTTAGGAAAATCacaaggaaaagggaaataattaatggaatcatagaatggctagaagga
acctctggagcTTATCTGGTCCAACCCACCTATGGTCAAACCACAGGGATGCCTA
GAGCAGGTTACCCTAGCACCTGGAGcatgtttttcaggaaaattgttttaaaataattgatttaAGGG
AATTTAACTGATTTAAGGGAACATAATGTTAAGCATTTCTCTGTTTATAGTAA
TTCAGATGATTTATGTTTTTAGTCTCTATTTTTGTACTAGTGTGGgagacagaaaaa
caaccacTCCATCTGCCTCTTATTACCTCTTCTCAGTAGTTGCTATTCACAGATT
GGAATTATTAAGGGGAAAGGTCTCAGGGAGAAAAATCCCTGCCATTCTAAG
AGAGCAGCAAATTCAAGATCATCGAGATGCTTTTgatttatatgaaaatatagTTAAGT
TGATTTTCAGCTTTGAGTTTCTATTATATAAAGAAATCATACCTCCAAACAAA
CTAACTACACTgtatttggttttggtttgtatTTTGGAAATACCCATttagctgtgatttttatttagtAAT
TAATGTATTAAAGTACTGCATGAGTGAAATAGTTAATGGCACAAATATATCAT
TATTACTCAGTTGTTACTTTAGCTGTGCAAGTCCAGATAACATGAAGAGTAT
ACATATAAATGACAATTCTGTTCccctttccttatatttaAGACACCTGCACTGATCCC
TTGTTAATAATCAGGTTTTTGTGATAAAATGATGGATTGTATGTCTTTGTGAT
gggaatatatttttgtttctaacttCATGTTACATAGCAGAAATGTAATAATACTATTAAAAAC
CCCAGACAATAATCATATTGCAAAGAAATCTGTCTTGCCTTTCCTGCCCcaccg
aaaaaaaaaaaaaaagaaaaaaaaaaaaaaaacagggaagaaaaaggtaaacgcttttttttttttctgaagacaaatgttt
tccctataatcttaagaaaaataaaatctatcgGGTTTTTTGCTTCCATTACGCTTATGTTCTTCT
AATTTTTTATATAAGACTGAATCCCTAGAGTAACAAACTTCTCTTTCATCCCA
TTCTAATCTACTGAGCATAGCAGTAATTCGCAGTCACAAAATAAACTCATAT
ATTCAAGATTAGAGTTCTAACTCACTATTTTGGAAAGTTCTATCCTGATTATT
ATTACATGCCTTCTCACACCTGTTGCCTGATAAACTGTTAACAGAATCATATA
CCTTTGAATATATGTTGTTACCCTATTAGCTGTCCATGGAGAATTAGCTGTGG
TAAATCTCCACATGCAGCTCTTGCATACAGAGCTTCAGTCAAAGAAAAGTG
TAGAGCCTACATCACAAAAGTACTAATGCTCTGACAGCATTACAAAAATagctt
agaaataaatattttttcttaaatagctGTTCTGTAAACATATTTAGGCCATTCAGATATGGTT
CAGTTCTAACCAGTAAACTGAGCAGTGGTGGGACTGCACCAGCTGCTACA
CAATTAAATTAGCCCTAACTTTGATGTTGCTCAAGGAACTCCACTGACCAAT
TCCCAAGAACATTCAGAAGTCAGCATGGACCAAAAAGCACTCCCACTACA
CAGTTGATGCGCAGATActatttttaatctgagaGATATAGACTTTTAGAATtagttctttttaaa
ttattctatatatttttagaatattttaaaagttttataGAAGTCAGATTTATCAGACAGAAAATCGTT
TCACATTTACATTATTGTTTCAGACCTAGTCTATGTCTAGCAGCAAAGAAG

GAGCTAAATGCTTCATTATTTTCACCTTTAAAAATTTTAATGATTGTTATGTC
TTCATTTATTCAGATCTAAATTTATGGAGGTTTTGGGgggctttttatttcttttctaatgaaA
GTTTTCTGTATGGTCCATAATTATATTTAGTTGCAAGCCTCTGAAAAAAGCaa
gtacatatattttaaagttgtCTTCACAACATTTCAAAGctagcaataaaaacaaaattatgaCATGCAA
AAAATcaagttctttatttctttactaaCTATTAAGAGCTTATTCTGTAGAATTGCTTTAATC
ATACATAGATTTAGTAGCTCTCCTCAAAATTGCTCTGGGTGACGCCACAACA
AAAATggcttaaaaacaaattcataaTTTACAGCACATACAAATGTCTAAAATCTTAGAT
TgaattagaataaataaatatatatacagatgTACGTAAGATAGATTACAAAAGTTCCATGTGC
ACTAGAACATTCATTggaacattcatttttattattaaccacatatgacagaagaaaattttcacATAATATC
AGTTGACTTTAATAAATGTACACAAAACTTGAAGGTAAATTAAGGTTATATG
TAATATTCATTGAAGTTAGTGGAAAAGTTTCCTTTGATTCCAGAGGGGCTTT
TGATGACCATATGGACATACAACAGGTGTTAGTTGGATAATGACTCTGAGAT
TTTCAGAACCTAGCTTTGACATACTAGGAATGTATTATATTGTTACATTTGTG
TCAAGAGGTATGCTGTGTTTTGTCATATATTTATAAGTACACAAAGCAAAAT
CCTTACAGGTAAGGAGAATGtatagcaaaaatattttgttactgtattttttttgaaaaaaaaaaaaaaaattact
gatgaATCTGTACCACTCACATACTCATAAATAAGTATTTCCACAACAAGAGTT
CACATTCCCTTAGTACAAGGTATGACATTCCATTTGACTCAGAACATAGATT
TAATATGCATCAAACATGAGAAATATCTATTCTTAAGATAGAAATGTAGAATT
ATATATATTCTTTGATAAGGTCTTTGAATAATGATTTAATTacatgataaaaatatatactta
ttagacataaacaaaacaaaaaacaaaaacaaaacaaacaaaaaaacaataaaacgAAActaataaggaaagaaaaa
gaaagccaaagtCAAATGCTATCATCAGTTTTCCACGAGagaattatattttcttcacaatAAAA
CCAGTAATCATAAAGAATAAGAACAAAGAGGAATCTGCTCTCTGCATAGAC
ATATGTTGTCTGTTGAATATACTGAATTACCTTCATAGAATTACAAGTGACTA
GTAAAGTAGATAGcgtctcacttttttttttttttctccaaattttgAATTGTACTCAAATATGATTC
TGGTGAGCTCTTTCCAACTTGTCAGGAGCTTATTTAGAATACAACTTATCTC
AAGTACATATATGCAAAAAACCTTCCTGACATTTGCAGAAATTTTTTTTATC
GATGacattttttttataaaagcagCATATGGCAGTATAATGAATACCATTTTCCACCTatgaa
aggagagggaaaatttaaaatattattagaatatttcttccttttagtaAGAATGCtgaatgagaaaagcagaaatcctT
TAGAATATGCACATGGTAGGAAATACCTTACTCCACTGTAGAAGAAACTTTC
AAATTGTTATTAAATATCTTTTACTTTCAGCTAATAACAACATATTTAACCTTT
TACTTAAAATCCAGTATTGACACCCTAGATTTCAGACAGCTCCCTGTTTCTT
TGTTGAGACTTCTAACTTCCCACTCTACTGAGCAGCTGCAATCATCCATCTC
AAAAATAGTAAACAAAAGATTAGGGAACTAGTGCATATGAGGTGATCAAAG
GTAGATGATATAAGAAATTTACCACAAGAGACATAAGTGTGCACCATGCTTA
TCAACAACATACATATGTTCAGAATCATAAAAGTTGTATTTGGATGCATGCC
ATCTCTGAGATTCAGATTAATTCCAAAACAGTTCTATGTTTAACACCTAATT
GTCCTGTTCGAATGTAACTCAGAGTTTATGAAAAAagcttgtcattttttttccagatttaaaca
gatttgaaacaatttgaaaaaataataataataaaatgacaatagtaataataataaactgtCAGAGGAATAAGAT
TTTGTAACAGAGAGAAGCTGGTATGCTTGATATGAAGTATCTGCCATTCAGA
TTTTGCTGTTGAGGAAATTGAGTGGATCTCAATTTCCTTTTGAGTTGAtctgaag
aagtgaaaaattcagaatgatttttatttatttattattttcagattgaAACATTCCAGCACTGTGTTCTAGA
AAAACACACGGGTTTGTGCCACAAAACCATCTTTGAATAAGCTTTCCAGCT
CAGCACTAATGATCCCAGTGAATAGCATGTCTTAAGTAAATTGATGTCTACA
ATTCATACTCACTGGATTCTGATGTCCCATTATATATCattcctcaaaagaaaaagtgctAA

TGTGTAACTCAGAAGTGGGAATGCTGACTTCTTAATCagttccatttcttctttcctataga
ctctcaacattttttatttaaaataaattatcattCACAGACAGAGATGCATGACATTTTTCAGGaat
ctcatttcatttcatgtcTTCTAAATTCAGTGTAGAAGCCTATGGAAGAGAAGAGATAAT
ATGAAAACACTAAGGTTTAATTCAgacaaaacatggaaaaaattgtGTGCCAGACAGAT
GAAGTCCACATTGTTTATGAATGTCTGATTTCTCTGGATTTTTTACATggcattttg
attttaaaatgttaactatttagaaatataaattaTAGAGTATTGGTAGTCTATATGTTAACTAGCTG
ACTGGCATCCAAAGGGTACAAATAAGAGAGGAGATATCAACAGGAACCAC
CAAGAAACATTCTAGCAAAGCAATAGATCAATCATGACTTCAGGCCTTTAata
tctttctgtttaaaacaagCTCTGCTGTAATTCAGTTATCAATTTGGCCAACACAGTAGT
CCTCTAAGGCTTTGGAATATGTGTGTCATACATAAATACCCTATGGAACTCA
GTAGGTTCAGTTTATTGTATGTAAGAACCACTGTGGCTGCTGTCCAGGTAGG
TACAGAGTACTGTCAGTCCCAGTCGTGGtctcttcctcatcctcatTCACTAGAGAAGC
TTGAGAGCTGTGCAAAAAAAATTTCCTTGGGTAAATTAAAACtagcagcatttgttttt
ttacttttaaagaatGGATGgaacaaaaatgagaagaaattccTTTGTGTCTTTCCATGACTTAGGT
GTGATCAAAAGTAGTTTTTGACAGTAACACGGTCTTTGATTAGCAGTCTGG
GTGAGAGGGTAAGTATTTTGATGTTCAAAGAAATCTCAGAACTGAACACCA
TCCTTATCACTCTCTGATATCACCCTCCACATGATATACACTTAGGCCCACAG
TCTGTCTTTTATAAACTCTGTATTTTTCCTCCTATTGAGATCCCTGTGTCCTCT
GGATAATGTCTGCTTACTTTCACTAGGGACAAGCATCTGACAAGCCGCAAC
TTCTAGAAACACATGAAGAATGGAGATGGTAGCAACCCTCAGGCCAAATCT
TCAGCCAAGGCAGAGTTAAACTAATTAACACACTGCTTAAGAATCTGACTT
TCAGTGACTTCCTTATTTAAATCATTCAACAtaatacagaaagaaagtaGGACAAGT
AAATCTTTTCCGTGTATTAGCTCGGAGCACAAAACATGCTTTCTAATAATGC
TCAGGTAGATTTACTTTAAGTAGAAGCCATTCAGAGCTCCGGTTTTCCCATA
ACACAACACAATATATGGTGGTGCTTACTTCTCATTCCTGTACTTCTTTGTGC
TACAGTACTTCATTACAATCATGTATCCCTACAGTATCATCTTCATTCTAATTC
AGCTCATGATGTCAAAATAAAGCTGAGAGTAGAGAAAAAAAGCGTCTTCAT
AGTGTATCaaaatggataaaaaaaatgaaacaaaacaaataaacaaaaaccgACAAAGATTGGAAG
GATCAGTGAGAAAGTGcatgttattttccatttggaGCTCTTTCACTAGTAATATTAAACT
ATCAACTTTCTCCAACATTCCTAGAGTACTGCAACCCTCGACACAAGGTAC
ATGAAGTCCAAATAACTAACAATTAATCAGGTCTTTAGAAGGTCACTACCTT
GAACTCCACAGAGAAGTGCCAGAGACTAAGGCTTGAATAAAGCTGTTCAT
TCCAGTCTTTAAGCTGGAAAGGTAAATCTGGGTAACTAAGCATTAAATAGT
GCTTTGAGTGAATTGCACATGTTCTCTTTCAGCTTTACAGGCAACAGGATTT
ggtcaaattaaaaaatatgaaatgaggCACATAAGGAAAAGTATGAAGaataaaatgataattttaaagct
catttcagaaataaatgattttgaaatggGAGCACATTCTGAACTAACTGTAAATTCAAGGAC
GTTGCACTCCCCTTGGGCTGATCTACATTTGCAATGGAGAGTAAGATCAAA
ACCTGGAAACAGAGATCACTGCAGCAGTCCTTCCCAGACACCTTCACCAG
CTGATTCCTTTCATTACTATAGGTACAAACTTGTCACCATGTGAATTCAGTCA
TCTTTGTAAGAACCTCATCTgacttcttttctctccttacCTTCAGTGAGGAAtggtttctgtgga
agaaaatgaaggcagaCAGCAAAGTCACAAAAATGCACTACTAAAACACAtagtacattt
attttatacacCTTTAATTGCACAATAACTGCATTTACTGGTTTTGAATGTGTGTTTA
GCTAAAAATATACAGCAAAAACACCCTAGCTTTTGTTCCTGAGGAACTTCC
ATGTGACAATTTGTTATCCACTAGTTTTACTTACTTACAAAGAACCAAGTGA

AGGCTTCTGTTCTATTTATTCTAGCATGGGATTCTTTTGAAATCTTTGCTTTG
AAGATTGGTATGGAGTGGAGCCACATATTTCAGTGGCATTTTGTCTTAACGA
AGGTGTGCTTCAGTTCTCTTCTGATTGCCCCCTCCTTCCTGAACACCTACGC
ATATATTCACATACTTCTGAAAAAGGAAGTCCTGCCTCAATAATTCAGTTCTA
TTTCATCTCAACAAAAGAGTAGCTGTGACTGAGCATATTATTTTACATGATG
AAAAAATCTGATTAATGTTTTACAGAAATTATCCTTGCATGAGTTGCAAGTT
TAGGATAAAAACTTAGAGCATTAGTTGTAACTTTCAAGATATTGGAAACCTA
GAGGAGTCTACCATGGaaataacaaacacaaaagcagctAAGATTTAGTTGATCTGTCTA
CACATAGCTACTAATTGCAAATTGAGGTGTCTGAATGTGGATACCTATGTTT
AGACAGTTAAATACTGAGTAAAATTTTTCCTTGTAGATATCTAGAAATGTG
ACAGGTGTTTATTGTGGACACTTTGGCCAAGATTCAATTGTCTGTGATGACC
ACAGACAGACATCTAGTCTCATATGAGAATTTTCACTGTATCTGAGATATCT
GCACAGAAGAGACAGATCTGGAATAAAATCtgtgggagaaaagaaaccttCTGAAAG
CGTAGCTATGGGGATTCCAAATGGCACTTGATGCCCAAGTGTGGCAACAG
GATAATCTCCCTGAATCTGTCTGATGATAAAACTAATGATCAAGGATTAGTTA
TTACCTCAGGATGTAAAGTATTTAAAATCTATGTTAGTCACCTAATTTGGTTT
AGCCAATTCAGTACTCCCCACAAGCCTTTGAATTATATTAGGCCTGAACTAA
ATGAGCAATGTCACCAGTCATACCAGTTATTAAGATATACACTCAAATCCAA
AAGTTTATGGCATTTGTATGTGTTTTGTCCAACTTCAAGTGTAGGTGATTTTC
AGGTTTTGATTCAGCCTTTCTGTCAGTTTAATatcttttgttctgaaaataagtGGAGGTG
GGTGGAGGTATTTGCAGAAAAAGGCATGCAGCAAAGGAGACTAACACAGC
AAACACAGGACATGTCTGCACTGGATTCTGCGCTGATGACATCGCACCCGC
CAGCAAGCTCCAAGCTGCTCTCCGCTGGGTGAATGCACCAGCTTGCCCAG
AGACAGCACAGAGGTCCTTGCACAACACTCCACACTGTGAGCTAGATGTG
CCTCTTGGGAGCAAATTCTGAACTGGTAAAAATAGGTTCCGTGTATGGCTG
ACAAAATTTGCATCTAAGCATTTGGCTCTCCAATGTATAAAGTGctaaaacagagg
aaagctaGGACACTACCTCTTTgctttttggtctttttttttcttctggttgtGAGGTGAAGATGAAG
GGATATATATACTCAGAAAAACTCAAAAAGAGTTCTAAGcagaaatgcacattttaaaC
TAGAACTCTTTCTTTGCAAATGAACTTTAGATCTGGAAGTCTGGGGTCTTGA
TGGCTCATTTTTTGCCAAATACCTGGTAATTTCCCTATTATTTTGAATCTTATA
TGCcttctctaaagaaaaaatcttcTACTGGAATttattaaatgcattaaaaatatatatctgtgtAAATATATA
CACTCTCTAAATGAGAGACTCCAAATTTTCAGATTTGAAAACTGAATATTAG
GCAcctaaataaaaagaaatgtccTGTTTCAAAGACACAAACTACCATAGACATAGAGT
ACGtagaaattaaatttatatttgaTGTATTGTTCTTGGAATGTTATTTACTTCTTTACAAC
TTTATAGCTTTACAAGTTATTACACACACTTACATAGCAAATCTGTGGAAAtat
attcaattaaaaaaaaaaaatgtatttaacatTAGAATGCATCTTAGTGTGTTAAAAACAGAATA
GGCCATATCAATTCTTTACCAGGTGCGTTGGGCTACTGatgaaattttttttctaaaatacaa
atattgaTTGTGTAAATTCTTTATTAATTagataaaacattttatctgtCTACTGTAGATCATTA
GACTATTGGTTCTATCAGTAATGCTACATATACCTAACTTAAGACTGAGACA
CTGTTCACTGAAATATTAAGTGGATTTATTCCAATTGACTTTCTGCTACACTG
TTAATTAAGATTATATTattcagggaaaataaatagcCTAATGAAAGAAACCCAATTCCA
TTCTGCATTTAATAAGAATTTTATTACATATGTGATAATAATagatttaaataagaaaagt
cAGCAAGACTTTCTACAAAACAATTACTAGTTACTTTTTCTGAGCAAAGGG
GAGGAGAGGTGTTCTTTGGGAAATaagtcttcagaaaaaaataaatactcctCTCTATAAAT

GAAACATCTAAGAGAAAGAATTCCAAGTCCATAAAATGTATTACCTGATTAG
CCTGTATCAGTTCCCTAAATGTAGGATGACAATGTTTTAAGAATTCAAAGAG
AAATATGAAGTCTctgtttaataataatatttgcTATAATCAGCACAGGAGTAGCATGTAC
TACAAATGAATTAGAAGAGTGAGTGAAAAGCTCTGTTCTTGGTAGcctaaaaata
cagatgtttatCCAATATGCGCAAAGAGAACTGagtaaaataagaattattATAAGGCATTAG
TAAATAGGCACAATACAAATTTAGTCACTTGTGTTAATtacaaaaaggatttttttaaaaaa
agctttgtttttgcaCTCATCTTTTGAAACAGTTCCTAAAAGTAGTCTTCTGATCGTAA
TTCTTATG
>TCONS_02730384
CCCTAAGGAAACTAAAAATAGTGTGTTCGTTGTATCAAAATCTGTACACATA
TTCGGGCAGACATACAGAAACCGGGGGTATGTGAGCCTCCATcctctttttccctcctt
tctgacagggagctggaggaggacgcTGGACCAGAGCCCAGGATGGAGTGACGCTGCGA
GTGCCCATTGCTTGCtaccagctctgctgcagccacaagACCAGTATGACCACTGGGTTT
TACTGATTTTTACCTGAAGGCTTGGGTTCATGGGGTATGTGGGGTGCacctgcct
tcctgcagcaggggTGGGTGCAGATTGGGGGGTCCATATCTGCAGCAGGGTAGGA
AGGGTGCTGGCAGTGGGAACGCAGGGCACGCTGGGGGGTTGTGGAAGTT
TTCTCTTGCCTTTGAAGCAGCATTTAACCAGGTCAGGAGCTGGCTGCTCAT
CGAGGCAGGACAGAAGTCTTTCCTTGCTTCACCATGATGGGACCCAGTTCC
GTTTCTGGGTGGCTTTTGCTCTCCAGTGCTGTCATCAGCAGTGTCCCAGTG
CAACCCAAGGTTGTTACTTCGTTTAGAGCAGCAGCCATCAGCTTGCAGCAT
CCAAAAGATGCAATGAGAGTTACCCATCCACAGCCCAGCATCAGCAGTGCC
TCCCGCAGTCACAGCACTGAATCAGGGGATCTTGGCCAGCTCTCCTCATCC
TAAGGTTTCCTGCTGGGAATGTCTCATTTTCCGTGGCTGAGAGCCTTGCTCT
TGTGGGCGTTGATTTGGTCGTTGCACAGAGGAAGAGAGGTGGAAGCAGCA
GGATTTGATGCAACAGTTTGGGAACCAGATGTGGCTATGAACTGGActccaaaa
agcaaaagcaaaacaaaaaacattggTAGCATCCAGAAATAGACCTCAGGGAAGAGGTC
AACGCAAGTGGCTGGGAAGGTCCCAGGTGAAAATCCTCCTGCAGCAACCA
CTAAACAAACCTTATTTAGACTGAGACCATGGTGGCCTCCTTCCTTTTATCT
TCTTATTGTGGCTGTCAGCCTGAGACACTACCTTAATCATATTTTTCAAGTG
GGAGAGCCAAGGAAGGAGTGTTACCATGTTACCACACAGTTCATGTGGCTT
AAATGAGAGCTGCTATGGGTGTTTTGGCACAGAGCTTCCAGACAGAAAgcat
ggcattttttttctgtgacatacagggggaaaaaaaaaaaatcagggaaaaaaaagtgctttggCTGCTTAAATTG
AAtgtaaacaaaaacatacagacAGACATCTGTGGTTTTATGTGTTTTCATGGGATTACA
GGCCTCAAAAGctgctctgtttcttcagctAACCTGTGGTTGCACATGGCATGAACTGA
TGCGTCTGAGCTCCAGCACTGAGGAGGGGGGACCGgtctttgcagtgctgtggtcTGC
TGCTTGCAGATGGGGTTTATGGCACAGAGGCTTAGTTTTAGCCTGCCTTGGT
TTCCCTGTGCATCAAAGGGGCTGTGAAGATTCCGTCTGAAGTGATCCCATG
CCGCTCATGGCTGGGCACCTCATCTCCTACACACCTGACCCATCAGCCAGC
ACTGCGTATCGATTTGCTCAACCCTTCCAGCGAGGAGCTCAGCTTTGACTC
ATGGTTGAAAGCATTTCCACGTGCCTGTGGGCTGACTCACAGCTGGAGGA
CGGATCACGGCAGCACGGTGCTCACCGTGTGTTGTGCTCATCACCGTGCCC
AGTGACTGTCACTCGGGCCTGAGTCACCTCGCACCTCCGCTCAGGATCAGC
CAAGGAGCCCCCGGAGCTGTCAGCTGGCCTTCTCTGTGATGGGGCTCTCTT
TGTACCCTGTTCTCATCGCCGCCCCGCCAGTTTCTCTCACAGGAGGACAAG

GGGCTGCAAAGCACACAGAGGGCATCCAGGACCTCCTCCTTTCCTGGTGT
GGTGTGGATGAGCTGTCACTGCGGTGGGTGTCATGGCAGCCTagagctcctcacag
ggagtggaggggcagcacgAAGCTGTGCACTCTGAGGATGGTGACAGGGCTTGAAG
GGATGGTGTGGGGCTGTGTCAGGGGAAGGTTGGAGGGTTGGGGAAAGGCT
CTGCACcagagggaggtggtggagtcaccatcgttggagctgttcaagaaccatgaagatgtggcactgagggacg
tggtggagatgggttggtggttggacttgat
>TCONS_02812260
CCTGGAGGAGGACATGACTTGTTCTCGCTGTGTTGTGTCAGCTCCTCCTGT
TTCTCCCGCTCCCTTCCAGGCAGTAGAAACTGATGGGTAACTTTCAACCCCa
attattttcctgcagttcAGTGGCTTTGAATTGATTGTACGGTGGAAGGACCAGATGTG
AGACCAAGAAATGAGACACGCATGCAGAAATGTGACAGTTCTGGATTGAG
GTGTGTgtgtaaggaaggaaggagctcAGAGGTTTGAAGGACAGCAGTGGGAGAGGC
GGTGAGCAGAGCAAGTTGTTCCCCATGCTGGGACCCAATAGGGAAAAGAA
GTGGGACAAACGGACTGAGAAACGAACCTGGTAGCCGTGGTGGGCACAA
AACAATGGCAGACTGCCCCTAT
>TCONS_02845054
GCTTATAATCTGAAGCAGAACCCCTtcccagaaggaaaacaaagtgtaTCAGCAGGTCA
GTGCAGCAGTCAGACAGAGAACAGATCTTGATTCTCTATCTGCAGCCAAGA
TGCAAAAAAGACAGTGGTAATGAATgttccagaaagaaaatgaaactcaaagcataggaaagaaa
gggaacagCACACAGGAATAACGGCAGGCAGAAAAATTGATGCTTGCTCTGAA
TGACTTCTAATTTTAAATTCCAGAGTGCAAAAACATTTATGTAATTAAAATTC
TCCTGctattctaaaacaaaacaaaaacaaaaaaattctgtttacaCTTGAGAAACATAAAAGGATA
GTTGACAGAACACATTCGTCAATGAAGCTTTAAGTCATAGCTAcctcaagaaaaaa
taaacactttacAGACTCTTACCTAAAAATGAAACTTACTGTTTCTTAGACATGGAT
GAGGATGCTCACAGAGTGCTGAGACATGCAAaggaatacacacacacacacaaagggcAT
TTAAATACTTTTAGAGAAAACTGAGACAGAATGATTTTAAGGATCTGtgataaat
gaggaaaaacCATTAAAATCTAAGCAGATAAGAAACAACTGAATATTCAGagcatac
atacatacaagAACAAGTGGGAAAAGTGTGAaaatttgacaaaaaaaattgtgatgAAGAATTT
TTCCAATTAAACTTGCTCGCAGAGCGGACTGAAGCACTGTTAAGGATCTGG
TGGAAAACCTCCTCAGCAAGAGCTAGCTGGGTAAGGGTTGGGCTGCAAAC
TGGAGCAGCTGTGAGGACCTGATGCATAAGCAACATGAACAGTAGGATGC
GAGAACACCCACAGACACCAGAAGTTGTGCGAGTTCATCTCTGCCTCCTG
TCACTGTTATTTGTTCTTAGTTACTAGCTTGACATAAAGCAAGGGAGAGTGG
GAaggcatgttttattttatttcagaatataaaGACTACAAGCTGATCTGTTCCTTTTTGAGACT
AAAACACggtaaattggaaaaaaaaaaagatcaatgaTGTAGATGATCTGTGAGAGTAGGAA
ATAAAGATTTTTGTCAGTAATTTGAACTAAACAGCAGAAGTCCCAATTTTCA
CCATCCTTCAAAGACTTCCAGCGCCTCTAGCTCTGACACGTCAGCTAGACA
ACGCGCACAAGTTTTTAAACCAATGCTGTCTAAActatttatgttttatttaacaaaaaaaaa
agggagcacaaactaacagaaaaaaaaaaacaagaaaagaaaaacatacatacGCATACAAAACATACAt
ataaaaaccaaaccactgtCACAGTCCCTTACATTTTACAATAGCACTGAGTCCAGTGA
AAAATTCAGAATATAGAACTGGCAGTCTGGCATTCAGGCTGCTTGcacttgctttc
cttttacCTGCACGTACTATGGATGAATGAAGACGCTCATCCAAAGCCATATTTC
CAATAATACGAATtatgtttctctgtatttttgcaGAGTCTTTACGTAGCTGGTAtatcctctgcag
cagctgaaggccGCCCTGAGCTTCAATTTTATCACAGTGAGATGAAACCCTCTGT

GCAGCAAGAGTCTGACTACTTTCACGTAAAGCAAGAGATGTGAAGTACTG
GACGCATGGATCAAGGCCAGTCTGAGGTAAAGAAACTAAGAGCAGTCGAA
GCTCATCTTCTATGAGATAATCCTGTGAGAAGTAAAcacaaacagtttttaattcaatttttcta
aattctgaaacaaaacacaatatggaagaaaatgaagatcttAAATAGTTGAAGAAGCTATATTAGTA
AGACCTAGACTTCAGTGCTCTGCAATTTGGGTTTAATAGTTACTCTAATGCT
AAAACCAGCCACCTGAGTGATGGAAAGTAAGCTTTAAGCTTCATACTTTGG
TGGCCTTTTTCTAATATGCCTGGAAGGGCAAAAGAAAGAATAGGTTCTTCT
TGACACAAGATGCCTAAAAGTTCACCTCTGACTACACAGTAATACTGATGG
TGCTGCATTAAGATGAATGTATGAATTACTAGTTAGAACAACAAACAACACT
GCTCTCACCTTCAAAATACTCACACAGATGTCAATTTAGTAATCTGTGATAA
GTGACAGGAAAACAGATTCAACAACTTTATTTGCCCACAGTCACAGGATTT
ACAGTTATCATCTTATATCGTAAAAATACTCCTAAAACAGAGAATGTCACTAT
CAACCAAAGCTGGTTTTCAGAGTTGTGTTGGCTAAGATGCATTCTGTGACA
GGAATTTCTGCTGGTATTTATTCTAGTAACATTACAGAAGACCATCTTATGCT
CTGTGGGCCAATTCTGTGTGTTAAAGAAGCCGATCTAATGACGGCATTACTC
AAACCACcttatttatatataatcaACACAAAATCAAAGCAAGGTGAAATCTACCCAAT
TAGGATTTGTTGGTACCCAAAAGCTGAAAGTAAAGTGTCATGGATTTATGA
CAGACTGTTGACATAAAGAGATGGGCCTATAGAACaattagatattaaaaaaaaaaaaaac
taagtcAGGCAGAAATTACTGTACGTAAAAAGGATGGAGGAGAAGTCACATCT
CCAAATCAGAAACTCTGAGAATGCTGCTTGGGAATTTGAGATAGGTAACTT
AAGCTCTTCCAGATGTTAAGTATTTGTTTATCTGATTAATATGAGTCACTGTG
TCGAGGTacaaagcatttctgcagctaAGAATCACGCTCAGTGAATTTTCTGATGGAAT
ATTTTTCAACTAAATCTAACAAGTACTGTGATAAGCTAACTGAAGCTTAGTG
CATAGTAATTTTCTATGGTTCCTAAATTTAAAAAGTAGGCTAAGATGTCtaaaag
gagacagaagaatTTCTCGTGAAAGACAACAGATACATCTATATGAATTACTCAAC
ACATTAGAGGCATTTTCACAGGCAGAAAATTTGCCTTGTATTTGCACTTGGG
AGTCATATAGTATACTCAAAATGGTTTCCATACTTGACATAATGAAGGTTAA
AGCTGTGTGCCAGAGCATTACTCAGAGTATCACACTAGCACTGAAAATGCA
AGCTCCCCCAGGAGAGCTCTCTACATGTAGGATTCTGTCAATGTCATCAAG
GCAATCTATGATCACTTAAATATGAGCGCATTGCCTGTTAGGCACGGACAGC
AGTGTATGCACCGCACTTGTGACGTGCACAAATTACATTGCTGCTGGACTT
TGTAATTTGGACATACAGTCAAAGAACAATAATTAAACTCCTATCTTCAGCT
CTTTGCATTGCATAATTCACTAATATAGTCATGCTCCAGAGGTGCATTACAGT
tgatgttttggttttcatttttagtactcattttttacttttgaagAATTCTATTTTAGTGTGGTCATTAACAA
TCAAAGTACTAAAGACAAACAGGTAAGCAAAAATCAGCTGGTATCTTCTTT
TCATttacttacatttttcatttttggcaATGATGGTGGAGGCAGGAAAAAGCGAAGGTCGA
TATCTTTGGACCGAGCCAAACCTATAGCAGTCCTTTGATCACAGGTTTGGGC
AACTGTACCATACTGATAATCTGTAGTACAAGAACTCCAGCATTAAGGTTAA
AACTGattgtaaatatttcatttggctttaaatttaatttctgatttcttttctgagttgAAAAGAATAACCTCTC
TATGATCTCCCTAATAAGCTCTGACTTTGTAGTCACAAATCCCAAAAGGTAA
AAATAGCAGTTGTTAGACCAGCGCTatgctgaaacaaaacagaccTGTGCATTAATTGT
TGATTACAAATCAGTTAAAGATTCTCATATGAGTTGAGCACATGCAAAAGC
AAAAGTTCAGTATTGCTACTGTGAAAAGAATGCAAGAACTACAAATTCAAG
CCTAAACTTTGTCTTGAGTAAAAAtctgaatgcc

>TCONS_02891589
CACATGACTCCCTATTGCGCTGTTCCAGGGAGCTGATCCAGCCAGAAAGTA
ACCTGAAAAAgaagtgctgagctctgtgaaCCTTCTTCCTCGGAGATCAGGCTAAAgga
caacaaaagaaaaccagtgGATAAAGGTTCCGAATCAGAACTGATGTTGCCCAGACA
CGATGCTGGATAATTATGAGGCTGCGAGGAATCCCAATAGTCTCCAGTTGTT
ATGTGCTGTAAGTAAGGACGAAATGGCCTCCTGACATTCTTTGGTGAGGAC
ATTGTGTTTTATTACACGGATGAGTCTCTGTGCTTATTTACATGCAATTGTCA
TTATCCTTCAACCATGGAGTCTGAATTTTCTGTTCCGTGCTTCTCACAAAGA
TTGCTTAAAATAACAGATGCTAACTGTTGCAGACAGATTAGTTAATTTTATA
ACTAGTAAAACAGTAAAGTAATAAACATCAAAGTGCTTAGGTCCAAGGTCC
AAGAATATAAAGAAGGAACAAATACCTAGATGTTTGGTGATTAATTTCCAAC
TGCTGTGTATATGCATTTAATTGTATGCTATGACCATTCTCTTTGGTGTAGTAC
AGCCTGTGCGTTATCACCACCTAACTCTTCTATGTGTTGTGCAATTTATTTGG
GCTCACATCTCACTAGTCAGTATATTGGTTTTTGTTAGTGTTCTTGTTCCATA
AAGTGAATTCTTTGCATGAGAAATACAGCTAAGGAACAAATGTGTTGCTTT
AGAGGGGCAATGTTCTTGTTCATATGCATAAGGTATTATTTAACACTGGTTAT
ATACATTGGCTTGcatgccttttttgtttgctaacATACATTTTGATTGCATGTGTAAAACA
TGAGAACTATTTTAGATGCATATCTCTACATAAAGCAATGGAGTTAAACATA
CTTGCCTGTTCTTCTCATTTTGATATTTCCTCTCTGGCCAACTGTTATTACTTT
TATGCATTACAGATGAGCCCACTCCCCATATAAATCTAGCGATAAGCAAACT
GGCTGAAAATCTTGTTAATGCTCAGGAGTTCATATTCTCACTAGAGAAGAC
AATTAtgctaaaaagagaaaagggtaGATCATAGTATTTGTAGTCATGCAGGAAATAGGT
ACAAAATACAGAGACTAGCCCAGATTTCCCAAATTTCCATCAGTATTTTAAT
CAAAGaacatatttctctgttctttaccctctctttgttctttctcatcTCAGATACACGTTTTATGATGA
TAGCCCCAATAAAAATGCATGGCACAAAGACAATATATGGATTATctgctgggaaa
ataaaaactttcttgtttttctgtcagCAGTCTGTATGGAGACAGAGAGGCCTGCAAGTGCC
AAGACCAATTCAAGAAAAGCCTAGGgaatttcctttctgcaaaCCTTTCAGatctacttgtttttt
tttttttttttgccttaatttGTCTCTAGGAACAAATAGAGAGAAATCATTCCATGGTGATA
AATGTGGCACAAATGTTCTGTAATTTTACTCTTGTTCACTAGACAGCgatcattat
ttttctttatctcctAAAGTTGTCTTTATATAGAGATTGTCTGAAGACACAATCTTCAG
AGGACAGAAACAATATTCTTACGCATGTGGAAGACTTCCATTGTTTCCCCT
GCTGCTCCTTTGGCTTGATGCTGTATGCAAGGATTAATTGTAGGAAATCAGT
GCTCCTCTTCCTAAACATAAACTAGGTTGAACTGCATTCTTCCTGTCTGCTA
TTGTTAGCCAGAGCATTAGAAAACTGTGTAGCAAGTCAGTCATGGAGGAG
AAAAAGTAGGATGATTTGCTTTTTGCTCAGCTTTGTGCATTTTCATCTGCTG
AAAGTAGATTTATTTGCACTGCAAATTTTGTCTTTATGTGAAGGATGATTTCA
TTGTATTCAATAAAATTGTATggtgaaatgaaacaaaaaagtatagggtt
>TCONS_02992617
CTATTCTCTTGCTGGCCTACTTCCCTCTGCTTGCATTTTGCATTCCTAATGCC
ATGAATAAATCAGGACCCAAGGATTTAGAAGGTGGTACAGCTGTAGCCACT
CAGAAAATGGAACCTCTGAGGCTTGCACTGCAGCTCCAACAACTGCCATG
AGGCCCTCTGTGTTCCCTGCCCACACACGGTTACCTATCTGGTGTATGGGAA
TGGAGCGGCTCTGTCCCTTTGTGTGAGCTCCATCCCTTCTGCCTCTCTGAA
GGTGGGCACTGCACGACCGCTCCAATCAATGATCCCACATCAAAAACTCAT

CACTGTCAGTTTGATACGGACTGCCATGGTAGAATTCGGTCCCTTCTGTTGT
TTCAGATAGGTGGAGACCCAGAGGAGGGAGCTTCCATCTCAGCACAGCAC
GGGGTTtggtggcacttgggaaagagACAGCGTTTCTTTGGGCTGTAAGATGCACCCC
CTCTTTGTAGGCTTCCATAGAAGCACGGAGTGGTTGGAGTTGGTGCTCAGA
AGTGCACACAGTACTTCTCTATCATTCCTATAACCTATGCTCATCCCTttctctgca
ggcagagagatGGTGAGAGTAATTAATTGCCTTGGACTATGTGAGCAcctgggctgctct
gctgaagATGGGGCTGTCAGGTCTGCAGTTAATGCTTCGGTTGCTTGGTGCTAC
AAGGCACTTTGTGTTACGGAAGGTTCCAACGGGAGTGATTTGGCCAAATTA
ATATTGATTTTGAATAAAGAGCGAGCAGTACAGATTTTGGGGTCGTACCAA
ACGCTCCCATTTCCAGCTTTAAAATGATTCACAAATAGCATTCATAGACCCC
ATCCAGCCATTATCAGAGAACACGGTTGCACTCACAGCATCCCATACTCTG
CATTCAGTCAGTGTGAATGATGCCACCTT
>TCONS_02993340
CCCTAGCAGAGAATATCTACATCCTTTTCTGGTAATTCCTACAAAGAGCTGA
AACTCTCATTATCCTCTTCCTAAAGGTCATTCCTGAGATTCTACCTAAGTACA
AATACAGCCTTCTGTTGGCTTTCTCCACAGGTGATAGCCTTCCAATTTACAG
GTTACTTCTTCAAACTGGTGAGGTCAGGAGCATTAAGTACACTTaagaaaggtaa
aaagaaaaaaacaactggctGGACATTGTGGAGGGGTTACTAAGTACACAGTTGTGTT
TACTTAAGGATAAAGCACAAAAGATCAGGAGTCATTGGAATTAACAGCAGG
ATTCCTACaagcattgtttttgtttagtttgtcTTTCACATGCACTCTGCCCATCAGAGCTTT
TGCAAAatcaaacagacaaaaaaagccACCGAAGAGAGTGAGCTGTCTCTGTTACTGT
CTGAGGCCCTGCAGATGCCACTTTCCCTCTTGGGTGAAGTTTTGTTCCTCT
GCATCTGACAGCTCCTCAAACCTTCCTTTGCCACTTGCCTTTGTCTTGTTGC
TGTAGTTCTCCCTTCCTCTTCATTTATTCCACATTCCCAAGTAGGAAACACTT
CCTTCTTGCTCAAGCAAACATGCCATCCGAGGAAAatcccaaagcagcagcagcagctg
aatgtAATGACTCATGATGGATGTAATGGCTCATGATCATTTCTGGTCTGGCAT
GGATGGATCCCAGTCCTGTCAGTCTCTGATGGGTACACAAATGGAGGCATG
GAGGCATTTgataagaaaaaagttttcataGCGATTCATGTTCCCAGACTGGTTTCATGA
AGAGCAATAGAGCTGCTGGACCTTCCAACTGAGTCTGACTGAGACATGGT
CGCTAAAAGAACTGACCTGTCCTTCACCCAGAATCTGGCGGCAAATTTGTC
TCCCATCTGCACTGTGGTGTGTGTTGTAGTACGGTTGTGGAAGTTGCACTTA
AATGTTGAGAGATCTGTGAGTGTAGTTACGTGACTAAGCATAATCTGCTTTC
GGGGAGAAAAGCTATTAAGCTGCAGAAATCTTATTAGCAGTCCTGAATGGT
AGACCTTGGTATTAGAATTTAAATTGCAATCTTGGTGTGTTTCAGTTCATCA
GAGTTATTCAAGGCATGTTTGTGAGAATTATGCCTACACGGTAAGAATGCAT
AGTGATATTTAGTTTAATATACTAATTAATTATGTCTGGATGTGTTTACCACTC
TGACTGTACCATCACATTTCCACCACCGTTCTGAATTAACCTAGTCTTTCCC
CTCAGATCCTGctgcctttttcattttcttttccctgggaTGTTGTCTCTTTGTAATTACTTTATC
AGCATTTAAACAGAGTGTTTCCTCTCAGAATAACGAGCAGAGGAAGGCAC
CGGGCAGTTTGTCCCCATTTTGCTAACAGAGCATCTGAAGCTGCCTTGGGC
TGCTTCTTCCCACTGCCAGGTCAACTCTTTCTCCATCCCAGGGTACCGCTCC
CAAATGATAAGCTGCAGAgttacATGGCTTCTTTTTAGCAGGGTGTCCTTTGGT
CACTGCATTCCTCTTGAATAAGGTTTTTCTCCTCGAAATAAGAATaactataaaata
aataaaaaagcaaccaTTTGGCAGTTGAGCATTGTTCTCTCCATTGTGGTGCTGTATA

GTCCCAGAGTAATTTGTATTGCTGGACATGAGCTGGTATTCTGTTTAAGATAT
GAGGAGTGAAGTGatctttctctgttttagtAACCATAAACAACTTGTTTCTTCCACTTT
GTGAGGTTTTAATTTTGGATGAAGAGTTACTGCATAATTCTTCTAGGAATAC
TTGTACTCGCTACTtcaaaaaatatcagaaagcaCGCGGAGGTGATGGGTTTGTgtgctggt
ggttggactagatgatcgtagtggtggtcttttccatccttaatgattctgtgattcagtgaaaaCGTAGCATATTGT
GAAACTGTGATCCTGTTCACCTTGCTATCAGTGAATGTATTTGCAGGAACGC
ATTCACAGGGGATGCAGAGAACACAGTTCCAGGGACTTTGCTTAAAAAAT
CTCAACATTCAAAGTGACTTTGTTACTTATATATGTTTTCCTGATAGTTGGGA
AAGTGGCAGGGGAGGAAGACTCCTGCAAAGGAAACGCAGCCCAGGAGAT
TAGATCATGTTGCTTTTTCCGAAGCTGCGCAGCAGATTTTTCTGCGTAAGGC
tttgaataaattatttactttttagaTGCTTTTATTGTTGCATTCATGATTTAAAAGAGCACGG
CTCTGCTGGTGCGTTGTTTGTCACCTAAAACACCCTGCTCCTGATGGTGCCT
CTCTCTCTGCCTGGTGAGTGCCTTGGCACGCTACGGCGGGGCTCAGCAGTT
TGGTTTGGATAAGCATCAGAGAGCTTGTGTCCTCCTCAGGATTTCTAGAAAT
GATATGGGTAGCCTTGTGAGAGAAAAAAGCTCTCACGTGCTTTCTGGTATT
TGGCAGTTTGAGCTGTGTCACTGATTCTGGGAGAGTGTTCTTGAGATACCA
GAGACACCTTTCCAAAATATCCCCTGGAGTTTGGCAACATCTGTACACAGC
ACAGTGTGAGATCTTGCAATTTCCACTTTATTTGGAATGGGATGTTCTGGTG
ATAcatttaatcatagaatcaccaaggttggaaaatacctccaagatcatccagtccaactgtccacctatcatCTCAC
TGGCAGATAGTTTTCTCTTACTGACTTCCCCCTTACTGTATAACTCCTGTTTC
TCTTATTATATAGATTACTTACTTATAGAATACTTATAGAATACTCATTCTGTGT
GGTTTAAGACCAGATGCTTGCTGTGTCTGTCTACAGTGCACATcacaacaaaaataa
tccatACCCTCCATATGGAAATAGCGTGCAGTGTATACCCTACAGTGCATATGCT
ATACACCCGTAGTGTACTGTAACCTGACAGTGAATAGTAACCACTTGATTGA
ATTTCTAAACTTTGCCAGAGCACATGAGAGAATTTAATTTTCAtcaaaagaatatattt
taccATTGAATGATGTATACTTCATGAGAAGGACGGTGATTGCCTGAAGGATA
GATGCTGCCTCTCCAATGTTAGCCAGCATCCGCTGAGTGTGTGGCTGCTTTG
CTTAATTCTTTGCTGGTGCCGGATTGTTATGTACTGTAATGAATTAAttatcataga
ctcatagaactattaatgttggaaaagaccactattataaaagctttcttttctggtAAGAAAAACCAGTTGTTT
GAAATAGCTTTTTCACGATTAAGAATTATTTACTTTGTACGGTTAGTGATATT
TCTGAAGGAACGAATAGTTTGTAGATACTTCAGGGATATGTTTTAAATCATT
AGGCATTGAAATTGCTATTAATGACCTATGGATGTCTCAGTGTTTTTTATGCA
GCCTAGAACAGCCCAAGGAACATGGAGCCAGCATTTGTCTGTACAGGTGG
GCCCAAAAGCACGCATGCAGGCATGCTGCCATGTCAGTAACCTGGCACAA
AAAGCTGACACAAGTATGCCCATGTGGACAGCTCAGGCAAGAACTGGTAT
TTCCACAGTGATTCATCTAATCTCTGAAGCCTCTTCTAGGATAGCCTGGATG
AATTACCCCGTGGCAGTTCTTCCTCTTTGCCATAAAAAGAGCATGTGGTGA
CTAGTTCAAACCTAAAGGCCCAACTGCTTTTTTAAACTGTACCATAGAAGC
ACATTTGTGGGTTCTTGCAAATGGGATAAGGATCTGGCAGGCAAAACCAGT
GCTATGAGAAGCTGAAGCTCTGCTGGTCACCATAGCACTGAAATCACTTAC
GCCATCACCAGCCAGGCCCAAAATCTTTGCTAGGTGCTTTCTGACTATCCCA
TTGGTGTACTTCTGAGGGGAAGAGGCAGGAAACAGTAAACAGTCTTCTTT
AAGCAAAGAAGCCGGTGTTGGAGTTGTGTGAATTTATGAGATGTTGGGCAC
TGTTTGTGCCCTGCAGATAACTGATGCGTGGTGCCTCTTCTTTGAGGAAAG

CTGCACTTGATGTAGAAATCTCAGAAACGTTTAAAGGACTGAAGAGTTGA
GCTGATAATTCAtaaagtgctgctgcttccgTCTGAGGTTGTGCCGAGCTGATAGCCCA
GAATGGTGCTAAGAGGCACAGTGTTCTTGGAGAGGTTTGATTACAGAGAA
CTTACGAAACAAATGTCCTGAACTGATGACTAAAACCTTCTGTTGCTTTAGA
TAAGCTAGATGGTCATATGCAGAGGCATAATTCTCTATTAACCTTCTTCAGAA
TAACTtattcttgctttattttctgaacagcTAAAACATGACTTTACATTCTTAAACAAGTATA
ATGAAATGACGCGTATGCACAATATATATTTGCTCCGTTAGCATATTGGTATC
CTTTGGGTTGTGTATCCATGCCCCAAGGAGCTATTATTTTCATCTGTGATCTA
ATTAGAAGTCAAATATTGGCTGAATTAACAAGTTGCTCTGTGCAGAAATGA
AGTATGTTGAGCATAACTCAGCGCAAACTGGAAAGCATCATTCCCAGAATG
TGAGGGAGCTGGGAAGTGAAACTGCTCAGGGACTGATCTCTGCCCTCTGG
TGATAGCAATAGAACCCAAAGGAATGGCATGGAGCCACAACAAAGACCAG
CACATGcgtgtgttcttttttctcctctgtctttAGAAGCAGAAAGGGATATATCCTTCTAGagttt
ttctctgtatttgtttCATCTCGATATCTATTTTCAAGTTACATAATGGTCATTTTGGATG
TCCAACTTGAGAAACTTCAAAGAAGGTAATTATTGTCGAATGATAGGAGTG
CAGTTAGTTATCTTTAAGATGTGTCACATAAGTCAGAGAAAGTTGTGAGTTA
GCTATCATCTATATTCTCTCAGGATAAATCAGTCATagcagatagcagcagagggaccTA
TTAAATCTTGCCGGACCACTGATGCTTGATATTATGACCCCCCAAGACCTCT
TCCCAACTACTTGAATATATTGATGCCATCTCGGCAAGTCCTACTCAAAAGC
CCTCTTTCTCCACAAATTATAACAGCTATAGGAAGTAGCAAGTAAAACAGTA
ATTTCAGGGCAAATCACAAAAGTTAAATTTGGGAAATTGTCTGTAGACTCT
GTAGCAATATTGCAATAATCAGATGGCAGCCCATGTGcagacaaagcagcagaatgTG
GAACTGTTTCTGTAGTTTTGTCTGCTGGCTGAATGTGCCCAGCAGCTGAGA
CTGGGCTGCAGAAAGCGGAAAGCCCTCTTGGCTAttggaaatataaaaatgataTTGC
CCaacacagaggcagcagaatAAAGTAAAACCTTTCAGCTACACACATCATTGAAAA
ACCCACgttcctcttctgtttcttctgggAAGTCACAAATAAGTTCTGCTGTCTGTGAGCA
TCCAAGGAATGGTCAGAAGGGAAACATCTCCTGTTGTCTGCATCCCCAGGA
AACAACAGACCAAAGTCAGAGGAATGTGAAAACACTCATAAAGATGgacata
ctttctttttctttttttttctttttgtttaaaaataaagctttgaCAATCTGTTTTTATATCCTCTATGTTTCC
AAGTTTTCACAAAAAGAGTTCcatgtttttaattgtttatgGAATTTGGCAAGACTCTG
>TCONS_03013272
GCTACTGGTTGAAATATTCATGCGAGATTCTATTTAACGTGGCTCTTCAGCT
GTTAAGGGAGCATAATGAATATTTCAATTTGCTCTTGTCCAGTTGAGTGGTG
GATTTATTCAGCAAGTACTTCAGACTCTTTGCTTTGTTAGTATGGGGTTTATT
TATCACTGCTCCTTGCTGAAAAAGTTGGTCTTGTTTCTAAGACTTCTTGGttgc
aagcagcagcaacaaaagaatATGCAATTTATCATTAAATTTTTCCATTCAAGGGTTTTC
CTTTCATGTCTAAATTCCCTTACAATTTCCCACGTTTCATATTTTTCAAGGTAT
ATTGATTTTGttatctgtatcttttttttctcctttgtagTCTCTGCCTTTTTAACTTCTGCTtctatttc
tgtgttctgttgAATGGCTTTAGAGTCAGGGTTGGCTTCTCTGACCACAGAATCCTA
GCTGTGATTGCAGCTTCTTGATATTGAACAAACCCTTAACTCCCAGCTTTTG
TCATGATGTTTATCCCATTTCTTCTCAATTTCATTCACAGTCTTGACTTCAGG
AGAAGGACCTTTTTGAAGTAACAGTATATATTAAGTGTATTTGGAGAGAGG
AAAACTACAAATTTTAGATGAGAGGGGAGCTTGGTCATCTTATTGCTCTGA
AAGTGGAAAAGGCACTCATGGTGTGAAGAGGAGAG

>TCONS_03018542
CTTAAAGCTGAACATAGATTTCCTGCCTAAAATGAAGAAGgatgcagagctgggctg
gtgTTAACACTGAACTGCCTGCATTTTTCAAGAAGCAGTTGCTGTACAACCT
TCAGGCCAACAACAAATCCTTGAGTTGACATGTTGGGCTAGcaggttggttttgttttct
tttgtttttcctcccctctgcttttcttctagTTTCATTTGCATCTGTTAGCCAGACATCGAGATAA
TGTTGGTAGAATGCTCATTACTGAAGGACCAAAGTTGGGAACACCATGTGG
TCACAGATGGAGCTAGGCTAAAGTAATTCATGTGCTGCAATACCTGTAAAAT
CTAGACCTCCAGAAGTAACTAAGGCCAGTGTTGTTTATTGTGTAATGTTATT
GCATGTGACACTTCTGTTGCAGAGCTCTTTTACGCACTTTTAGCCTATGGCA
ATATGTTATGGATAGAAGAATTTACTGACATatccaggaaaacaaag
>TCONS_03021143
GTTTGCACAAAGCAGGATTGTCCCATCAGAATCCATCCATCTCGTGGCAGG
AAAATCCCCCCGCCGCTGGTGTGGGGCTGTTTACTATAACCCACGTGTGCA
ATGTGCTCATTTTGCTGTCTGGGAGGGGATGTCGAGCCAGATTTCTGCAGG
GGGCAGTGAGCAAGCGGTGTTCTTCCCCTTCCTATTTCTTCATGCGCATCCT
ATACACAGGACACTCCCAGGCGGGCTGCTCGGATGCAACTTCTTGAAACTC
TCTAGAACCAGGAACAGATGTGaggcttttcctttcatttccctgctccactttgcttttttttttacagctgg
TGGATACCTTTTCCTTACACGTCACCTGGCCCAGAGGAAAACCCGGAGCTG
CTGCGGGAGGTGGGTGCCAATTCACTGACATCTGCAGAGGGCAGATCCATC
CAGCAATTTTGGAGTGCCTTCTTCATTAACGTTAGTATTTaaagaactgtttttgtttgttca
tcaCCAGGCCAAACTTCTGGCTTTGATGAATCACCCAGCAAGTGCATTCCAA
CACCCGTGTGTGAGGTGCAGGGATTCAGTCTGCTGGTGGCAGTGGTCATTT
CTGGTCACCATTTGGGATATGCAGAGGCACAGAGAGTGGTTCGGCACTCTG
CACCTTCCAaaagccccagagctgcaggaaaatcACCTCTCCCAAGGGTAGATCTGTATC
CTCGGGGATACAGAGCTCGGGGATGAAGGGCTAGGCTGTGATAACGGTGT
GGACCTTGCAAGGTTGGGGCAGTAATAGCTGAGCCCTGTGCTACAGCTCCC
ACTGCACAGAGGTGGTGTTTGGAGGCTGAGAGGGCTCTGCCTTCGTAACA
ACTCCTTCTAAAGTTTATGTAATTCTCCAGTAACCCAAGAACAAGACTGGA
ACTAATTACTGCTGTCTCAGGAAGCCCGGTGCTTTGAGCTAGAAAGAATAA
AGCACGCAGACATAATGCAAATCTGCTTCTGAGAGTAAACACTGATGGGGA
AATAACAGTCAGGAGGGGAGTGGGGATGCAGTGTGACACGGGGCAGCTGT
TGTGCATACAATGGTCTGAGTGTGGGCTCAGCCATCCTTGGCCCAAATTGG
TCCTGAGTACAGGGCGGGGTGGGATGGTTCCCCCAGTAATTAGTAATAGAA
TGGCTGCTGAACATTgtgtgctgctcctgactgAGTGGAGAGAGGATTTTATGGAGAG
CACTGGTTTGCCCTGGGAACCAGGTTGGCTGATCCCACTGCATGGGCAGCA
CACTGTAGCCCCCTGTTGGAAAACCGCTGCTCCTTAATACAAGGCTGGGGG
GGCTTTTATTGTTCAGCATAAATGAACTGAGTAACTGGATCAAATCCAATTG
CTGGAGATAAACAAGAATTCAAGCAGGAAAAGCTCATAACCCCTCCTGGC
CTGCAAACGTctcccagggcagctgcagagagctggcCCAGGGGCTAGAGAAGCCTTGCT
GGGTGTCTGAGATTAATTGtgttttctgaacatttatCATTGGAGGGTCCTGGCACTGG
GCTAAGTCTTCTCCAAGGCAGTTATCATAGGGAACATGCTGTGCTGGGGTC
AGGAAGTGCAGCTCATACTGGGTTCAGCTCCATCAGCACTGTGTACAGATT
GCAGGCAGGCTCCCAGCACCTGGAGGGATCTCCAGGTGAGCTACTCTCAC
AGCTGTAAGTCTGGAAGGCTTTGGGCTTTCCTAAGGCACCTCTTGATGAAC

CTTGCTCAGTTCCCCCCAGCCACCCATTGCCCAACTGCAGCATTGGCTTTG
TCTCTTCAGATCCTGCTGAGACATATCACAGAGCCTTCCAGTTCCAAATtgaat
ttgttatttcttttgtatttcgtttctttttccctttctccttggCTATCTTTGCCATCAGCCCTTCCCACTTG
TTGCTCTGCCAGCTCTCTATCTCAGCACTTCTCATCGCTCCCCGGTGTGTCT
GCAGGCAGACAGAGCTCTGTCACACTGGGCTGCACTCTCAGTCCCCTTccatc
ctgcagctgcctgtgctcccctctctgctctgcaaaaaTCCCAGCCCCCAGCTATGCCAGACCTGC
AGCAGCCACCCGGCTGGGCTATATAACATAGACACACTCAGACAGCCACCA
CTCCTTCCTCGACATATCCAAGGCAGGGATAGTCCCCATCAGAAGCCATCA
GCAGACAGACAGGGAAAACAGGCCTGCACTCAGCACAAGGGTAAATATTG
ACAGTGGGCGCTGCTGGAGAGCTCCTTTTGTTACTTCCCGCTGTCCCATGG
AAGCCACAAGTGTTTAATTTATCAGTGCAAAGGAGAGATCTTCTCTGCAAC
ATCCAGCCTCGTGCTCCTCTATCCCAGCACGTCTCCCCTGTGCCCCGCTCTG
TGCAATTCCCATTGCCATCCTGGAGGCGACAGGTCTGCTTTGCATCACTGAT
CCTCTTATTCTGGCGCCCCGGAAAGAGCTCTACTAATGCAAGTTGTGCAAC
TCGCCTCCCTGAAGCACGAGGCAATTAAGAGGCTTTTATAAGTCAttgattttcctt
cctgctggcAATTGCAGAACCATCGCTTTCGATCAAATGAGACGCGCCGTTCGAT
ACCCAAACGTTCTGCTCATCGCTGGAgccctttattttttatttcatgccATAGGAAGCGAC
TCATTAACACGGATCTGTATTGAAAACTTGAAAGCTTTTATTCTGAGCAATC
AGATCCCAGTGCTTTGACACAGGAAGGCAGCGGATCATCTGACAGCCCCA
ATTTGGCACAACAGGGATGAGAGCGCATCATATGTTTCAACCAGAAAGCAC
TTGAGCACTTGGCCCATTTCTCTTCCTTG
>TCONS_03075416
TCAAACCTAAGAAGACTGCATCCTGAAGGGAACTCGGTCAGACTGTCCTT
GCAGAGGAGTTTGTTCTGGAGGAAAGCTGCCTGCCACCTGCCTTCCAAGG
AACTGCCAGCTTTACCTGGAGAAAATAGCCCATCTGCTAAGTTATACAGCTC
TTTCATTCTTCAGCCCAGGTTACTGGTGAAGATTTTATATGTACAGCCTTGTT
TTTAATACTTCCAGCTGttcatttttcctgtgaaagGAAATCCCTGGATAGAGACACAAC
AATCCTGAAGGTATGCCCAGATTTGCTTCTGGTGTTCAAACATAGCAATGG
GGCAGAAGTCTCCTGCCAGTCTCAGCTCTGCTGACCTTCTGTGATGTATCA
CctctccccctcaacacagatATGTTTAACATGGTGCTGCAAGTGAACTCTGAG
>TCONS_03119618
GGAGGTCAGATTTGCAGCTTGGTCTGATTCTTCAAAAAAGTGAGACTGATG
AATCCATCCTGGCCAATTTTCCCTCATGGTGAAGAGAGCTCTGTGCCATGG
GCCTGCCTGCCCTTTCATCAGCTCACCTCCTAGGTCCTATATGCCATGACAA
ACCAAAGAGGGCAGGGAAATCAGACAGAGAGAGGACTCTTGAAATTTGG
GTCCAAAGGCAACAAGATCTCTATTCCCACTGCATGAGGAGCTATATGGTTA
CCACTTTTCTTCTGTACAGAAGGATctacacatgaaaaaaaaaaaaaacaaccaatgCAGAAA
GCTGCATAAAGCTAGTACTGTTTGTATCGATGTGTCACAGTTCTATTAGTACT
TAATCACTGCAGTATCATTTGGTGTCAGGTACACCAACAATTTTGATTACAG
TATATTACCCACCACTGAGGAAATGTCATTACAGGACACTATCCTCCCCCTC
TCACAAGCTGCACTGCTGTCTCAGGAGTAATGAGTGTTGATAACTGTAACG
ATATGCCTATAAAGGCCTTGCAAACCCTCATTGATTGTTTCAATGTTTGAGA
AAAACATACATAAAGATTATtgattatttcaattttataaGCTCGTTATATCTGTGGATACA
GAAAATGCCTTTAAGCAGTGCAGATTATTCAGGGCAAAGCTCCTCCTGTAA

TGCATTGGGAGATTGCTGAAATGTTGTTATTTGCATACTCACACGTGCACCT
CTGGTGCTGTATACCACTGTGATGGCTTATAAAGCAAAGTGATATTTGCCTT
ATTTGATCGCTCAGAAAGTTGCTGATGTCCTAAACTGTGTTGATTTGCTAAA
GCTGTTGTGCTGATTCCCATGACGTAAGGGCTTTCCTGATGTGGGAGGTCT
GCTCTGGCTGATGATGGGGTATGCAAACTACTGCCATAAGAAGTTGCACTG
TAATTGTATCACATGTACGCATACCCCTGCGCTCTGCAGAAAGGAATGCCAA
ACTACTCCAGCGTTTATCAGTTCCCTATCTATGGGTAAGCAGAGAGAGCTGC
TCTTCTTATCTTACCAAGCCCATGTTTCTGGAGCTGAAGGTCGCAAATACCA
AAAAGCTAGATCACAACTTGGTGTGAGCCTGTGTTTGTGTCTGTGGCCTCT
TACCAATGGCATCTCGTGTTTAAACTGAATTCCTGTCAGCATATATGGGCTG
CCTTgaagtgaaaaacattttcagctcaCACAGAAAAGACACATACTGAGACTGAAGTGT
TGCTCAGTTCCTCTGCTTTCTACCTTCTacatgctggctgtgctgcagcaaatgagaaaggTCC
ACTTAACAACTGTCATATACTGTGTTTATTATGGTCTTCAGCTGTTGACAGTT
ATGCAACTGGGATGAAATGCATCCCCATAAAAAGCGCTCATACGAGGCCAG
TGCCACCACATCAGTGCTGCCTGAACCCTCAAAATAGACCTCAGGtaggcccag
cagcagcttgggATCTAAATGAATGGCAGCAATCTGTGACATTTTGACAGCACtctgc
aatatttttaatgaataatcCCTCTACTCCAAGTAATTTCACTTCCCCGTCTCTGGTGCTG
CCTAGGCACTTtgagaggaaaacagcaaacacagcaagTTGCTCACCACGCTCTTCCCTC
AGTGAA
>TCONS_03123639
agggtaagtttagcttggatatcaggaaaaacttctttacagaaagggttgttaagccCTGGAATaggctctgcaggga
ggtggttgagtcaccatccctggatgtgtttaaaaccatttggatgtgatgctcagggacatggtttagcagagggttgttaga
gtagTTTgattaggttgtggttggacttgatgatctttaaggtcttttccaacctgagaaattctatgattctatgaaaagttTT
TGGCTCAAAAACTACAACTATTAATTTAGAATCTTTGTTTGCATCTCTGGCT
TTCAGAGGAGAAAACcatcttcagtgttttcctcctatcagttttttgttttctgcttagAAAATTACATC
ATTAATGTGATTGTAGGCAGCAGACACTCCAGAGGTAATAACATATAACAAT
GCAGCTTAGCTTTTACTTCCTATGTGGGAATAGGAGTGATGAAAATAAGATA
AAGGACTTTCATTTAAGTATGTCATTACTAGCAAATCGTACCACTAGAGTCT
TACTGTGGGCTTCTTTTATTCAAAACCTGATAAATCCTTTCATGTTGTTTGCT
CTCCATTCCTCCACATCACTgttgggtgaaaaaaaaatgactccaAAATCTCATGAAGTTG
CGTGGTTTTTATCTTCAACTGTGTACACTACTGTTCACATATGTAACAGCAG
AATGACTCCTGTATAAGGAAGATGATCTGCctactctgtattttttttctgtccgGCTTCTAG
CTGATAAGCTCAGTGCCTGGATTGCTGTGTTGTTCTTAATTGATGGATATGG
TCACTATCAATATTGGtgaaggaaatatttatgtatgtacaAATGTACTGAAACCtggatggaaaa
caaagaatagTGTTTAATACAGTCTTCAGCATAGCTGTATCACGTCAGCTTCATCT
TGTTTAGCCCTGGGGAGATTTATTAGCTGTGAATTCCCAAAGGAAATTCTCT
GTGAAATCAGGAGGCAGAAGGACCATACACAGACACAGAAGAGATTGGA
CTGGTGTGATTTCTCCACCTCAATAACCAATGAATGTGCCTAGAGGTGAGCa
gagagaggcagcagaggaTGTGGGTATTGGCTTGCTTAATGGCTAAGCTAATTATGA
GGCATGAGTCACTGAGTCATTCAGGTTCTGCACGCTTCAGCCACCAAACTG
GAATCATCTCACCCTGTCTGACCCAGGCAAAAGGAAATAACATCTACCAGT
TCTTCTCTGTGGCAACAAGTGAAATCATGCTTATGTGtgcatgggaactgttgaattaTG
GCCTGAATCACTGAatgatcacctgaggcaagcaccgagtcagctgtgggagcacaggtgaaggaattcac
ctgtgtgactggagggggtggagcctggctgctcCTCTCCTAGACCCTATTTAAGGGCCGACTG

CCAttagggaaggatctctttccaGAGATCACTCTTTCTGGAGTTTTTATGCAAGCCTAA
GATATGGGCTCGTTAACCAGACTgcaaaggaaaggcagagacTGTGTGTCTCCACCG
GAATCCTCAATGCCAAAACAGTCCCTGGGTTTTACAGCCTCAGGAGTTCCT
GGCACAGCATCCATCTGCTTTCTCTCAACTAAAATCTACAGTAAGATCCAAC
AGGTAGAGATAATGGCAAAGAAACCAATAGCAAAActgtaatgctttcttttccagtgaatA
TTAAATTGTATGTGAGTTTATCTATAGGTTTCCTGGTGAAATCTCTACAGAGA
GATCAGGGTTTCATTTTACATGGTgatcttttctttatttgaattataactgtttggaaataaaattaaatgttt
tttttctatctataTCTTTCTCTACCTCTATCTCTGTCAGAGCTGAGGGGCAGCAACG
GCATTCAGCACTGTGATAGCAGCAGGCATCTGTGTCCGAGCATGGAGACAG
CTGGGCACTGTGTATCCACCAGAGCCTTTCTGTGCATCCAGCACACAACGT
TATTATTAACCATGTGGCAGTGTCACCTTTTAGTTACAAGATTATTCACATGG
ATCTGAGATTCTTAATCTATAAATTGTTTTCCTAATTAGAATATCTTTTGGTTT
GATTAGACTTTCTGTaactgttttctatttcttctttctttcctccatgAAGTTGAGAAGAATGCTG
AATTACAACAGTTCCCAAAGGACTAATCCTTCAGCTGAACGCTGATATAGA
AATgactttcctttcctttgtggTATATATTCATCAGAGGTAAATTCATATGCATGCACACT
TTACTACATTCTTACATTTGAAAGATGTATTCACAAATGCTAGATTTCATATC
AAGTCTTTGTTGAGGTTCAGTGACAGTCTAAATAAGCTCAGAGCATTTCAG
ACCAGTCTGACATCAAGCCCAAGATACTGACTGCCCTGCTGTATGGTTCTTT
CAGAAGATCACCTATAGTCTCCAGTTAATAACACACAGGtacactttttttaatgtagtgg
CCTTTCTGCCAGGCAGCTAAATGCCTAGTGACTAAATGTCACCTCTTTGAAT
GCAAAGATATTTCATTGTCCAGAACTGTTCCTGGAACACTGAGCAGCATAA
CGATCCCTGATAAAACAGGCACCTGGGACAGCTGATATCACAGGGAATTTT
TATAGTCGCACACAAATTCTAGAATCCCATTGTAGGGTAATCTCCAAAGACT
TTCATTTGCTGGGttcaaaatgtcttttctaCTATTCTAGCATTTCTTTCCCAGGACAGAT
AATTTGTGACATTTCCTCCACAATTTTTTCTGCATCCTATGTTTTTGGGggcaga
ttattttctttattttcagcaacAAAACCTGGATAAGAGCAATATCTGAGCAGGTGagctttccttt
taaatacaTAAGACATTTTAGTACTTTAACTATAGCATGGAtattagatttttctttaatacaacC
CAAAGAGTGTCACTTAGGTCTCAGTCACTAAACATTCTCCCTAAACATTCA
AAAATACCAGTTAGGTTTCCTAATATCTTCATAACAGGTAATTATTTTACTATT
ATACTAACATGtgtatatttcttttcctctctgatcTTGATCCTGCTATTGTGCCCCTTTGGTT
TCCAACCTTGCCATGTATTGACATGGagagaaatctcatttttttgtAGGTGAAATCTTCAT
ACAATCCTTCTCTATCAGTAGCTGTGCGCAGGATTGTGTCCAGTCCTATTTC
AAATATCTCCATactttttttactctttttgcTTAAGAACACCAATTATTGTAATACtcaaagaaa
atgcacaaatgTGTGAGGGTTGCTGAGTCTGTCTATTGGGGGAATTGCACCAAAG
GGTGTACAACACTTGACATGCTTCTTGGGAATATTCAGCGGCAATACGTCTG
GTTATGCAAGCTAGTGACTAAATCTTTTACCAGCTTATGTTACACATAAAAA
GTTATTCACTTTAGTAAGAAGAGATGCCAAAATCTGGTACCTGCTAATTCAG
CAGCCACTTGTTTACAAGGATTTCTACTGACAGTTTTCACTGTATTCCTATTA
CTGCAGTATGTGAGCATCTTGCTAATCTTTTGTAAAATAACTTGATACTTCTG
TGAGATGAGTGTTTTCTATCAGTCATCCCCTTTGTTTACCCCTTGTAGAGGTT
ATGGCAGATGAGATTTATGAAGGTGGTTATAAATATGATATGGATGTCCACaa
gcaagagcagaaaaacTCTGATCTTCAGCAGAGCAACCACATACGTTGTGCCATGC
CCTGGACTTAACATAATGTTGAGGATCTTGACTCCTACTGAGAACAGTAAG
TCTCGTTGATAGGAGTCTGAGTATTTCAAAGCCAGCGTGGTATTGCATACAG

CATTGACTAGAAGTGGTGCCTGCTGTTAACTTTCAAATCACCATCCCAATCT
TTCTTGATGAAAGAAATTGAACTCTAGACCTCCtaccaaattttatttttgctgtttcaatcctcttt
tcctttaaaaatatcagcAGCCTACCAGACTAAAGTACACAGAATAGAGGGTCAAGCA
GCAGAAGGAGGCCTTTTCTACTCCATCCTCAGGCAGCTTGACACTGAAGCA
CAACAGGGGTTGCATATAGGGTGCAGTACAGAAGTGAGTTACCATTTACAT
GGACAGATAGGATAAGGTTCATGTCAGGAGTTCTAAGAGTATTTTTTGAAC
TTAATATATTAACATTTGACAAATATCAATCTGTCAAATTTGTAgcaaaaaaacaatg
caaataaTCAAAGAATG
>TCONS_03163306
TGCCTCTGTTGTGAAGCACAAGCCATGATGATCGGTTTTTTTCAGCTCCTTC
AGTGTGATCCTTCAGTCAGAAAGTAGCTTCCTGGATCAGCTTCCATAGCCT
CTTCAAAGGAGATGCCCTGAAAGtttctttcctgaaagaaaatccaCTGAGCTACAATCC
AgtgatttttaacatttttcagTCAAAGtagcagcacacagcatttgGAGAGCAGTAGAGTCTTAAA
CTTCGCCCTCTGAAGAATCTGTAAGTTCAGCCACAGAAAGGTGACAAGTG
CGGCAGCAGGAAAGTCTCCAGCAAGATCAGAAGGGGTTCACAAAGAAGG
CAGAGGATTTGCACAAGCATCCTAAGGATCATACCACACACTCATAAATCCT
GATTTGAGTGTCCACATTTAAGCTCAAGCATACataaggtttcttttcttttcatggaaataCTA
CTGAGAATGGTAGAGAGGAAACAGTGCAGAGGGCTGCCAACACACTTTCT
GCAAAAACCCACAACTAAGAATTTAAGTAACGTGCAGTATATTTGTATAAAT
TTCTGATGCATATTCCCTACTATGCATTTGAGTTTCcattcactgatgtttttcCCCCtataac
agaggaaaaaatataccAGCAAATTAGGAAACCTAAGGAAAGTTGATTAGATAGGGA
TGCCAAGAAATGACACTTGCCATTAGCATTCAGAAACGATGGGATGTTGGC
TAGAAGAATATGATAGAACTTGCTTTGGCTATGGAAGTCTTGGAACTTAAG
GATAGTGCCATTCATAGAAATACAAGACTAATTACTTCACTAGGAAAGGTAT
ATCAATGAGATTTAATCCAGATGAGTATAAATCTGATTTACCACAGAAATTCT
ACCAGATTTGTGCATAGAAACaagatattttataaaaatattaaaatataaaatattagcAGACTCCT
AAATGACATTGTGATCACCTTAAGACAAATTTAGGAATCAACATGTTTGACT
ATTCAGACATATTAAGCCTTAAATATCTCCAATACATCTGATCAAAATATTCT
AGAAATAATGAAGGTTTGTCTGACATACATTCACTCCCTAAACTCATCAAAG
AAAtttcagaacagcagaaaaagacaCTTAAAAAGATAATACATCAAGTCTTGAAGACA
AATTACTCACTTAAGGTTAGGTAACTTACTTTAGAGAAGAAACAGCTGTATT
ATCATTAGGACAAAATTCTCATGATCACCTGTATAAAATATCCAGCTACCTCA
ACTTTAATGAACGTTTTATCTTCCTGACTAAATTCATTACCTACAGAGCTGA
CAAGTCCTCTTGGTATGACCTCTTGGTCTTACATGTTAAAGCTTTGCTCACC
AGATAAAGAACTGAAAGCCAAATGACATTTTTTGTCCAAGTGCTCTCTTTCa
agctcttctcttttcagtttaCTGACTACCTCCTTTAAAATCAGAAGTTACATGTCCTTCCC
AACTTCACAAGTGCCCAAGAACAACTAAGTCAGTTAGCCTAATtgaattattttggtt
tttttttctgaactatgTTAAGCTGTAAGCCTTTGTTTTGCTATTGTAACCTCAGGCTAC
AGTAAATGAATTCGCTGGTACCTTTGAAGGGAGAGATGAAGTAAAATTCCC
CAAGtgcaaaatcaaacagaaaagaaaatctcttctgGTTTGCTTGAAATATACATCTTGTCCT
GCTgttcttttaatgttttcttttatgataaGAATCAGTGAAGGGCAGGTAAAGggtttttctcccccccc
ccccccaaaaagtaccagtttattacaaaagCGTATTGATGATTGCAGACCACTAGAACTCAG
ATTAATCCATTTGTTTACATCAGATCAATGCTCTTATTTACATGTCAGATCAG
ACAAGAGGAGGTTCTATTTTTTGGCTTCCCACACTTTTCTAACATTTTAGCA

CATTTTTGTGCATCACCATGAAGCCAGAGCCTCCTGTGGTTAGTTTGTTGAC
ACAAGATACTTCTCTTAAGCTATGCAGTCTGGCTTGCATTCAGTTGAGACTA
ATgaagaatgatttttaaattgcCAATGCGTCATAAGATGAACACTCTAAAACAAACCAG
GTCTCTGCACTTAGTCACTCATTTCACAGCTCCAGACTGTTCCACCACTTAA
ACTGTCTTGATATGTGGAAACATTTGCAAATGCCCACAGGATTCCAAAGAG
CTCCTCCCCAGATTAAAGTGAACCTCAGgcaaagaaacagctttttctaATAGTAGCCAC
TTTTTAGTCAACACTGAATACCCTAAATTCTCACATCGCCCCACAACCCATG
TGGCAAAAACAAGAATGGGCATGCCATTAGGTTATGATGAGAGACAATACA
AGCTAACAGGAGTAGTGCACTAATTAAGAAATACATACCTGAAAACTCAGG
ACTAGAATTTAAAGTTATCTTCTAGCTAAGAGAACACCTTTTGTACTTCAGT
TAGGAAAACGTGTTAGATTTTACCCTATTCACTCTTCATAGAAACAGAGAGT
GTATCTCTGTAATGAGAGGACAAGTTCTCAGCTGAAAGCTTCGTGAAGAGA
GCACTGGGAACAAAAACAACTTCTCTCTAGAAGACAttataaaattttaaagtaTTGTA
TTGATGTCCTGTAACTCCCAGCAAACTTGTCAGAGATTGGCCtcaaaaaaaaatgag
ggagagaagagaggtCAAGGTACCCAACCCCAGAGATTTGCAGTGCAGATTTCAA
GAGGTTGATGGTTGCTTCTttaaatcaaaaaaaaaaaaaaaaaaaaccccaccatgATCGCTAC
TCATCTTTTCACATactaaaaatactaaaaaaaaaaattaaaatgcatctatcttaaagaaaaaaaaattgattttaca
GTCTTCTCCAATATTCAACTACTATACCtataaaattaatgattttttttttttgcatattttaaggCAA
AGTTCAGAAACTTCCCATACTATTTTGAGCTCAGTATCTGACAACACATTTC
AAAGACCTGCAGGAGACGATGAATTTTGAGTATACTTGATGAGCCATAAAA
TATAGCAGTATTCAGCATAATTAAAGtatgttttctaattttttaagcaatttgCATAGTAT
>TCONS_03192310
CATGAACTTGAAAATATGGAGCTTCAGATTGTGCCTTCCTTTTGCAGAGCTC
ACATTTTGAACCGGAACAAACTAGATGTTTTAAAGAcatcatttctcttcctttcagacAA
GAATCAAACAGTTGGGGATTACAGaagagTTACAGAACTATGGAGTTCAAAG
AATCTTCACGGTTACTTCGGTCAGGACACCTCTGTAACATGCTTCTAGAAGT
TCTGGATAACCACAGTACATATTTCCAATCATCTAAAAGCTACCTTTGTTAG
AACAGACTCCAAAGTGGGAAGAGTTAAGGAATTTGCCAGAAATTGACTGT
TCTCAAAGGACTTACCACGATTTTTCAGTTCCTGCTTCACACTGAGTTTTCC
TTAGCAACCcccagtgttttgttttattacataTGACTGTTGTTTTCAAAAGATTTCTGGAA
GTAAATTTGATCTTTGATAAAGGAAGCGGTTAACATTATTGTGGTTACAAGA
AGCAAAATATCAAGTCAGCTACTGTGCTATGTGGACAGGGAAGTCCAGAGC
CAACAAGGACAGCTGCTTCTTTAGAAGACAACTTCCTTAGCACCGTGGTAA
TTAATCAGGATGCATCTCAGCTGCAATGGGTTTCACTGTAAACATCTTATTT
AAAGATGTCTGAGGAGCCCCTGGAAAGCACCATAAAAATGCCTTGTGGAA
GTTCATACGAAACCATTTCAGCATGGTTCTGTTCCCTGCTACAGTAATCATT
AGAATCAGTTCCAACCTTTGTAATCAAACACAGTGCTTGGAATTCTCACGC
CTTTTCCTCAGGAGAGGCAAACTGGAGTGCACCTTCTCACTGCTGGGCAAT
ATTCACTTACTTTGCTTCACATAACATCGACAGAAGAGGATGGCTGAGTGG
TCCCAGAAAGGACTACTCAGGTTTTTTTAAGGATCTCTCctgttccttaaaaaaaaagct
caatAGAGCCAGAACTGTGTGGATCTACCTGGAATTTGTTCTCACGTGTAAG
>TCONS_03192600
GGTTTTTATGTCCGAAATGTATAACTGAGCATTTGGTCTCGTTAAGGCTCAT
ACAGTTGGCTTCAGCCCATCAATCTAtcctatccagatccctctgtagggccttcaTACCCTC

AGGTAGATCAACACCTCCTCCCAACTgggtgtcacctgcaaacttactgaggttATCCACCC
CTTATCCAAGTCATAAATAAAGATACTGAATAGGACAGACCCTAATACCAAC
CCTTGGGGAATATCACCCATGACTGGTCActagctggatttaactccattcaccaccactctctgg
gacCAGCCATCTATCCAGTTCTTTAGCAAGCAAAGagtacctgtccaagccatggcTGCT
AGCTTCTCCAGGAAAATGCTGTAGGAGACAGTGCcaagtgctttgctgaagtgtaggtaga
ctacatcaacaacctttccctcatctaTCAGTAAGGTCATTAGATCATACAAGGAGATCAGAT
TGgacaagcaggacctgcctttcatgaacccatgctggctgggcttGATGCCCTGGATGTCCTACGTA
AGCTATATGATCTCATTCAAAATGATCTGCTCCGtaacctttcctggcactgaggtTGGGC
TGACTGGCCTGTAAGTTCCCTAGATCCTCCTTACAACTGATCCTTTAGATGG
GAATCACTGAAgcatagattcatagaattactgaatggtttaggttggaaaggaccttaaagattatctagttccag
CTAGTTCAAGCTGTGACCCAGCTTGAAAGAGCAGGAGCTGATAGCAGTCC
TCCAGTTTTATCATACCTGGTAGCCACTTCCTGATGTCCTGAATGCAGGCCC
CAGCCAGGAAGTGGACCTTTCATGAGAGATTATCTGGGCAGCAAATGTGTG
CCTCAGTGCCCCTTTTGATATTTTCATGCTATTGTCTGCCAGCTCAGCGTCCT
TAAGATCATGACAAAGCTGGCACTGTAATAAGAATTACAAGATTAAGAGCT
TCAATATACATCCTATTGCCAAGGACAAGATATTACCTTTGATTTTTTGAGAT
GCATCTTTGTAGTGGCATATTATTTTTGCCTGTAgatttgaaaaatgtaattctCAGTAAC
TTCCTGAAGAATGTAAAGGTATAATCATTTAttagaaaatacagatttctccTTAGGTGAT
CTAAATTGCATAATCCTCATATCTCCAATGGAATATATTCTGCTCTTATCAGCT
GAATAAGTGATGCAGTCTAAACAAAGGGTAATAATGTTCAACTGGAATTCC
AGGAAAGGAAGCTTAATGAGAACTTCCTGATTTAATCTTACAAAAATAGAA
AGGTTGGTAGGATAAATGAATCACTCAGAAGAACAGATTTGCTTCAGttaatt
aaaacagaacataaaaatgTCAAAGTAGCCTTTTGTTACATTTAACGGCAAAACTCAC
AAAACTATTTAGAGCATCATTTTCAGGAATAAGATTGAGAAGAATCCAAGA
AACAACTCAATTTTCTGAAAACTTATATGaagtttcttctcattttctggcCTGGAAATCA
AGGTGACAGCTATATGGACATTTCTCATGCAATTTCCTGTATCTTCTGCTTTT
AGAGGACCAGGAAAAGagtattttcagaagtaaatttGGAACTTCTGCTCCTGGAAGAT
ATCAGTAAGTCAGAGATGCATggaaattaaggaaaaacaaaacaaatgaactaTTTTGAGCCC
TAAGAAAACATTTGGTTCAATTTGAGTTCACTTTGACTTCCTGACAGGGAT
AGTCACTtaagtgtgagtaaacccacAGAGAAAGTGGAACTTACATCTTGGATAAGAT
ACCTGGTAAGTTAGTAAGTAGTAAGTGAGCAAGAGAGATGGTCCTGTGCCA
CTGGgataatggaaaagaaaacatcagaagtCTTTTCTATATTGCAAGTTTGTGACCCATAA
ACACTTTTGAATATAGCCAATATGAAAAGTACCCTAAATGCAAGTCAGGCA
AGAGACACATTGTGATTATagcctccttctttcctcctaacaagacagcaggaaaatgagtgaaaaatatgt
atgtgcTACCATAGCCCTCGTAGCAGCAAGCAGAATATCCACAGGGCTGCCAG
AGGCCATCCATACCAGAGGAACAACTGCTATGCATTTCCCAAGGGCCTGGC
ACATGCTGCATCCATCAGCTACCCTTGCCTTGGGTACAATATGTTTATGTTAC
ATTTGAACATAGACGATTCATCTTAGAATCAACTTTTTTGCACCAGTCTGAT
AATTTGCATCTTCTAAGTATTTCCAGAATAGAGCTCTGTTCCATGGTAAACT
GGTGGATGGGAGCCTTTTTGCTCTCATGCTGAGGTTTGGTATAATTGTtggcatt
ttttctgtttgcttttttttttaatcccaagTTTTTGGTTGGATTCCTACTTTGTTTTTACcagtttttacttt
cttcttgcttgttaatttcttttattttgtttgtgcaGTTGTAGgtttttcagctcttctttaTAAGGATATAAAGTT
GTTAACAGTGGGAGAGCAGATAGTCACTTCAGGGAATAGGACTGCTGGGT
GCTTTTATGACTGAACTGAGTGAtgccagaagaaaataagaggcAGAGCTGGCGAAGT

ATCGCATCTGTACATGTAAAAGAGCTTATGAAACTGTCTAGGGGTGGTTACA
AATGATTAATAGCATCCGCAGTGGGAAGACGAGCAGCTCCAGCTAATATTT
CATTAGCTAACATTCACCTCTATTTTACTCATTGCTGATCCCACAGACAACTC
CAAGACAGAATTAAGCAGCTTACTGtaaacatttgatttttcacAATACCAAGAAGTAA
TGAATAGTGTGGTaaagatggaagaaatgaaaagtagCACAAAAGAATTCCAAACCATG
GATGATCTTCTTTTGTTCGGGTACTGATTCTGAGATAGTGAAAGTGAGAGAT
TGTCTCTTGCTTTTAAAAGGTGATAATCCTGAATAACACTAGAAATTGGGTC
AGTACAAGTGAAGATAGGAAACAAATATCAACAGAAGCAAATTTATAGTGG
TCAGCAAAGAATGTGATCTCACCACTCCCCACTGTGCATTAAATATTTGGGT
GTTTCAGACAGCTGTGAGAGATGAGGCATCCATCTGTGATGAGCTAAGGAT
GAGATGAGAGCCTTAGAGTAGAAATGGAATTGCCTACTTTGGACCAAAAAT
GTACTTTATGGGAGGGTGTGCTTTGATTGCCAActatctgttttgttttggtactTGAAATT
GGTCTTAGATACCTCCCTTTGGAGGTTTAGCTGCAACTACAGATATGTTTCA
GAAGCTTCTTCcatctcataaaaaaaaaaaaaaagatgaaaaataaaaggaacgTGTTCAGCATGTA
CAGCTTTGCAAGATATATTTTTCCTACATACTTAGTAGTTTGGGTAACTATTG
TCTTAATGAGGAGACTTGTAGAAACACATTATGGTGTTTTCACATCACCACT
GTTTTTCCCATCAGTTTTTGATTACTGATTATAAGATATAGGAAAGCATTCAC
TTTTAATTATTGCAAGCAATTTCTCAAAGGTCATTAACTGTCAAAGTACTATA
ATCTACCGAAGTATTTTACAGCTTtcctaggaaaaagaaaaattcaaactAAAAACCTATTT
TGGTTTATAGCTAGAATTTGAGATCAGAAAATGGAGTAGGTCTGTATTTGAG
TGTAAATTATGAATTCAAAGATAAACACAATGAAAAAGGCCACGTCTATCT
GTGCTTTTACTGGATTATAGAGCCTACAAATTTGTATgaagaaacacatttgtttGCATT
ACTGTGATACCTTTCAGTGTAGGTTTGCAGAGCATGCAACAAACAATGTATT
TGGTCTCCAAGTAATTTTAATGCATAGGCTTTCGCTTCACAGtaagagaaactgaagc
aaatgGAGAGGATAGATATTTCCACAGCTACATTCTGAACTGGCTGAGAAAAC
TATGAACAAAATCTGGGagccattttccatttcttggaATTTACCTTTAGGCACCACTCAC
AGTGCCCCACAACTCTCCAAGACACACCTGGTTCgcactttgttttcattcaattgtcacaag
aggaaaaagaacaaaaagtctCAGCACAGATTGCATTTGATGTATGAACTTGTATTTGG
TGTATGGTATGTACTCAGGTATGCATCCCATGTAGTTTACTTTTGTCTTATCA
CTTATCACCTTATTCATAGTCTTATTGTCTGGGAATAACTTTTGTGGTTATGTT
ATTTGAAGGTCTTGGTGCAGGATGTAATCTTGCCAATGTAACTATACCATTC
TGTACCACCTTACCAAAGTAATTTTAAGTATCCAaggatatttatttctttgacatTCTAAC
ATTATATTTATCTGCAGCTTGCATGCACTGCTAACAGATGTTTTGGTCCTCGT
GTCCAACATTATGCAATAAGACAGTGAGTCCCCAGAACTCCATGTGTCAGG
AACACAAAGTACTCTAAGATAGTTCACTTTCATATTTTCTGGCATTTAGTGAT
GAGTAAATCTCCAAATGGTTTATAGGTTCATTTAGCTTAGGCTAACTATCCA
AAAATCTGATTCCTAGAACTCAGTTTGCTCAAACTACAAAATCAGGTTAGTT
ACATAATTAGAGTAGTGGTACCATTGACcaagtgagagagaaaaaactttttcatattttccagaA
CCTTCCTGGTTCAGTttgaaatcaacagaaaatttccctaatttttccttttatttttccttttcctttctctaacTAGT
ATTTCGCCCCTTGTATTTGCTACTGCTTTCATATTTGGGATGAAGTTTTATCT
CAGCTAATTCTATGTAACAACTCTGACTACAGTTGAAAGATgcatcttttatttatatata
ccTGCATGAGTACCTTTATGTAAAAGCTtgtctggaaataaaataaggatTGTCTGAGACA
TACAGATTTCAAATTTACTCTGATCATTATATTTCTTGAGATGGCAATTGAAT
AGGAGGGACTTCATTTGGAGTCTGTTTAAAGAAGCATTTGAAGTCACAAA

AAAACTTCCTGTGATGTACTATTTACTGATATCAACTGGCAGAAACACAGG
CTgcaaaagcagatatttttgtCACAACTTCAGCAGTAtcaaatattaggaaaattcagttttgcaaGTGT
GGGACAGCAAAACAATTTAATCCGTTTTGAATGACTTTATTCCCTTTGCACA
TTATACTGCACCTTTTTGAAGGACTAAGCATAGGTAAGCTGGTACTTCAGCA
CAGATGTATGAATGTACAATTAACATTTTGATGCACAGAAAAAATTCCTTAT
TGAGGCAATTATATTTGAAATGCTGcatagcagattttttttctcctaatggGAAATAATTGAA
CACTATTAAATTACTATTTCTTCATAATTTTGCAATGGTAACATTTTGCAGCC
TGtgtcatttgctttctgcagctgttatTTTGTTCAAGGAGTCCATGTATGACTTGATTTTCTG
TGAGGTTTCCAGAGAGAACCACTTTTATTTCCTTCGGTGCTCCAAGATTTG
GCAGGGCCAGTCTCATGAGAATAAATGAGTGAAGAAATTGCATAAATTTCA
GTGAAGTcacaaataattaatttttcttgGACCTGAactcatattaaaaataataataaaaaaaaaaatcaagaag
catggtgaaacaaatgaaagtaaCAGCCTAACGTGGTGCTGAAACATAAATTCAGTAacca
aacactgcagaaactTTGCAGGCTATAGACACGATAGAAGCAACAGATGGCATTTTA
GATGAGGGTTCATAGGATGTTAGGAAGCTCACATAGCACTTCCTGTAGCAC
GAGCAGAGGCAGAGTCTTCCTATCTCAGCTGCGCTGCCTGTGGAGACTGCT
TATGAGGGGCATTCATGAGATCAGTCACACACATCGCCTGCGCAGTACTTT
CCTGGGGAAGATGCCTGCACCAAAAACATTCAGGTGGCATGTAGTAGAAA
AAGCCACTGGGGAGGTTACAAGCAGTGTCTGTTTTCTCCTGTTATTGTTAG
GTGTACTCCAGGCACACACAGTGTAAGCTCAGATAGAGAGGAGTTTCTTTG
CTAACCCTTTCACTACACCTTCAAATTCTGGAGGGAGTAAGCAGTTCTGCC
CCTATTACCTCCATCATCCACTGAGGACTTCAGATCTAGCTTTTAATTGCTCC
AAACTCAAACTCTCCTTCCTCTAGGCCATGCTTGCCACCTGCTTAGAAAgtcc
tctctcctttctttctggaCTTCTCCTTTGTCAGAGGTAGAGTGACTGACTCACACTTTC
CCAAGCAGGTTTACTGACAACCTACAGTTCCCACGGAGGGGCCTGCACCG
TTGCTGTGGGTCCTGCGAGCCATGCAGCTGGCTGAGTGCTCAGACAGatgctg
gaaaaaatgaaatgaaaatgattacTGAAGAGGTGTGCCAGCCTATTCCTTGCTGATATAT
TGGTAAGTGCTCCAGAGGAAGCAGAGcccagttttcctgtttttacaGCCAAATTATTGC
AGAAAAGCCAATGACTCTCTGCTTTTATATACTGGGAAAAATAAGTAGGCC
ATGAGTATTTTCCAGACAAGCTTTAGCCATGAACGCATGTAATTTAGAGAAG
CTGAAAAGAGTGCACTTGCACATGCATATGCAAGTTGTCAATATACCCTCTG
GAAAATCTGCTGGAGCTGGCTGATATTCTTACAAATAAGTGTGGGGTTATTA
TTTCACTGTGTTTAGGTCACTTTTGGACACAACATACTGAAAAGGCAAACA
TTAGCCATATTATATCAAGCAATGAACTTTCCATATGTTGGAAATGGTGTcatga
aaagaaagagggagtGCTTAGTTCATCACCAGAGATCCTGCCCAGAAAAGATTCTC
AGACTGAGCTTGTTACCATGCTGCCTGATCTCAAAAAAGGAGAGGGATGTT
ATTTctctcaaagaaataaaaaataaaaatagaccATCCATCAACTAAAACTAAGACAAgtatttct
ttctcactgttGAAATGGGATATTATTATTCCCATGGGGATTAATCAGCGTAGCTGG
AGGAGTCTGGCCCTTCGGGAGGTCAAAGGAGGAATGCTTATGATTAATATT
TTGGTCCGTGGTCTGTCTCTATGCTAAATTAATAAAGCTATGTCATCTTTTAC
TCTCCTTTATTAACATAACATTAAAATTCTACTAACATTGATGTTGCAGATTG
CCCTTGGCTCTGAGAAGTAAAATAATACTTTATGGAAAAGATTTTACTGTGC
AAATAAGCAGATTTAACATCCCGGTTCGTTCCCAGCTCTCAGATGTCAGCAT
TGCCTGTAATTAGCTGCGAGCTGGAATGTGAGTTGCCATGCAGTAACCACA
CTAAGCATCTTCGTTCACATCTTGATTTCCTATTTCAAATATCCCTGTCAAGT

TTAGAAAATGAGTTGTTGAAGCAAAAGAAAGTAAGGGAGAGGAGAGTCT
GGACCAGCACTTAAGGTTAGCCCAGCATAGAATCAGCCagttaaataaatacttaaaaaa
aaaaaaaaatgtagcttttaaaaaaaatagaaaccttTGTAGGGAAGCATTTCTTAAACAAATAGAT
AccttgaagaaaataatcactgGTATTCAGCAGTTAAAATTATGTTTCAAACGACAGTTT
CCATTACAAAGTTCAAAGCTCTCAGAATGAATGACTAGAGATTACAGTGGC
GCATTTACAGATGATGTGATTTTTGATAATCAAGATTAGAGGTCTGTGAAAA
GCAAATGCCTACAAAGTCCACACAAAGGCCTGCTTCCTCCCAGCAGGTATG
GGGAGGGTATGTTAACCTCCCCAGAAAACCCAGTCATAACCAGTCATTATC
ACGAGGCTGCCAGAAACAGATTCTGCTATTCAAAGCAAATGTAGGCTGCTA
TCACTCTACTGACTTCCACTGAGGTGCTCCATAttggcttttgagcagatcCAGGCTGG
CCTCCCTCTAGCATTTTGATGCCAGCAACAAAAATGTCAGCTACTGAGGTC
TGTAAACATGAGAGATCTGCTGTTTTAAGTCCCAGTTTTATTCTGCCACCGT
TCTGTTTGCTGCACacataaatgcatttgctgCCTATAGAGAACTTGAACAATACTGTG
TTGTGGCTTTCATTAGtatctgattttgtttttcaagtggCAGAAATGTATTCCACAAATTTC
AACTGgcctggagagcagctgagtGGTTTCCACATGATTTCCATGTGCAACTGGAAGT
GTACATTTAACCTCTGACATTTCAATTGCTGTGACACTGGATATCCCAAACA
CTAGCACTTCTGTTAGATGGAACAATTACTTTTTACAGAAATTAAGAGAGA
GATGCTAATAGCCCTTGCATTTAGTCATATGGGAGCTGTAGGGAAAGAGCTT
TCCATAAAGTTGTCTTCCTTTCCTGGCTGTCAAGAGCTTGGACACTTCTTGA
GATATTTCTGCAAAACCTGTTGGTTCTTCCAAACTTTGGTGGGAGATTTAAA
GAAAGGAtaaggggaggggagaaagaggTGGGCCTATATTTTTTCTGAACTCCCCATT
CGGTTGGACCAATACATCCTCAGATTGctaaaaagtatatatttacTTAAATAGAAATAC
ATCATAAAGTAAAATCATTGGTGTTGTATCCTCAATATACTCCTATGAATGTC
CACTAATACAAAtcatttgggaggaaaaaaaaaaaaaggtaaccAAGAAGCAAATAGTAAGAT
TACAAATGCCATGATGTTAAATGCTAAAGCAAGCAACTCTGGGATAAAAGA
TGCAAAAATGTTCAAACTGTACTGGGAAAGACGTGTCTTTTCCCTAACGAG
TTAGGGGTTGCAAGCCAAGCATCCAGTGACTTTCTCAGGAAAAATCACTGT
TCGAGGGGTTCCTGCTTTTGCAATCCCAAAAACCTTCCCCCCACAAAAGCT
GCATTAGACGGGGTTGACTAAATGTGGGATACAAATATATAGTTGTGAACTG
TCCACTTGCTTTGGGACAGCTCTCCCTTGCCTGTGATATGAAAGCAACACT
TTACATTCCCATGCAGATTGCTTTTCCTGACAGCAGTGTTCAGTCCCAGGGC
AGCCATGGAGCTGTTTCACCCTGCCTTACAGGGCTGGAGTGAGGCCAACAT
TTCTCCTCATGCTTCTCAGAGACTGAGTCAACACACCTGCTTCTGAGAATC
TGGAGCAGCGAGCAGCCAGCGATGTCATCTTTGGCTCGGGTGCTAGAACTT
GATCTGAATTATGTTCTTTAATTCAGGTTTTAACACTTCTAATGCAAGATACT
AGAGGAATTTTCATGACctaaagctttttcttttcccaaaagatgtgcagaaagaaagtaaaaccaCTTCT
TGATTTAACTAAACCTTTAATCTTTCTTgtgaaaaatataatgaatgttacttttaaaatacaaaatcttt
TACTTTGAGGGGCAAGAAACAGGAAGGAACTCACGTGAATAATGGTTTGA
AGGCCTAAGTTCATGGGATTTTTTTGATTGATTTCTTAATAGATCCAAAGCA
AAAAGAGAGTTTTAATTGAGGAACTTACTTTTACTCCACAGCAAAGAATCA
AAATGACCACAACATTGTGTAGGTTTTCTGACTTTAGAATTGCTGCCAACCT
CTAATAGAAGTAGAATGGTATAGACTATGGTGCAATAACACTAGCAAAGTTA
GCATACAGGTAATATGAACTCATCTGAAATCTTTGTTGCAATGCCTAGGAAT
GGGCAGCATCATACATGTGCAAATCATCACAGCCCACTGCAGAGCTAAGAT

CTAGTTACTACCAGCAGATATTTCACTTGCAGGCTGGTTTGGAATTGGTGAG
CTCCATGCACTTCCACAGCCAACCAAAATAAACTTTAGTTTATTGGCATACT
TCTTCCAAGATTTTCCAACTTTCTCCTCATTCAAAGAGAGAGGCTGCCAAAT
AAGCTTTGCTCAGTGCCTGAAGGAAACTGTTCAAGGAATCTCCATATCTTC
CTGCTGTGTGTCCTCCCACTTATTTGGTCACAATAGGAGCTAGACCAGTATG
CTGATGGAGTTCTCCATATTGCAAAGCCAATAGAGAATTTTGagcttttcacagaatc
atagaatggtttgggttggaagggacctttaagatcatctagttctaatgtccctgctataggcaaggaCACTTCCTT
CTAGAATAGGCTGCTgagagctccatccagcctggccttgaatgtaTCCAGAGAGGCAGCAT
TCACAGCctctttgagcaacctgttcccatgtctcaccatcctcacagtaaagaatttcttcctaaaaagaATTTCtat
ctaaatctaccctcatCCTGTCGCGACATGACCTCATGCTTCCTCATGACTACAGCTTc
cctgtaggccccccttcaggtactggaaggccactataaggtccccttggagccttctcttctctaggctgaagagccccaact
aTCTCAGTTTGTCCCCATAAAACAGGTGGTCTAGCCCTCTGATCCTCTTTGtg
gacctcctctggacccactccaacagctcaatgcctttcttgtgCCAGGGGCCCCAGAACTAtacatagtac
tccagatggtgTCTCATGACAGCAGAGGAGagaggcagaatcacctcccttgacctgctagCTACG
CTTCTCTTGATGCTATCCAGGGTATggctggccttcttggctgcaagtgcacattgctggctcatgctg
agATTTTCATCAACCAGCAATCCcccagtccttctcctcagggctgctttcaaaTCATTCTCCACC
CAGCCTGTATTTATATTGGGATTGCCCTtacccaggtgcaggaccttgcacttggtcttgttgaacttc
atgagattggGATGGGCacacctctcaagcctgtccaggtccctctggatagcatcccttctcTCTAGCatatcag
ctgcaccactcagcttggtgtcatctgcaaacttgctgaggatgcactcaatcccactgccCATGTAACctacaaaga
tattaaatagcATTGGTCCTAGCACCAATCCCTGAGGAGTGCCACTCATCAGTGGT
CTCTacttggacattgagctgttgaccacaactctccGAGTGCAACCCTCCTCATGAATGCCAT
CTATTATTTaatgctgcaaaacagaaataatttaaaaatagagcAAACTGATAtcacagatgcagaaaagga
TCACTTACTTATCCGAAATAATACAATACAGctcctttaaaaatagaagtctAACTTTGCT
TCAGATTTCTAGTCAAACTTCCCACAGCTT
>TCONS_03234147
GGTAGCACATGTATCTGTTACCTTACTGACTAAGCTCtacaatgtttttcttctaaatacaTA
TTGCCCTTCAGTCTACCTATATGGATGATACTCTacataataaaagaaatttcaCTCAGTG
TCAGAAGTAGTCAGTAgccttttgttttaaacatttaagtTAAAATACCTGACTTCCATTgcca
aatcatttttttcaccacACTGTACCTATTTCATATTTGTTGGCTTTGTAATATCTTCAGat
agcaaaaagaaatggatgtgCAAATTTAATTTGTCAGTAATGTACGCTGCATGCTTTT
GATGTCATATGTCATGGTTGTGTTGTTAATGTGTGTCATACCATTTCATCTCA
TTCAACCAGGGTCTACCAGGGCGAAATGGCTATCCGGGTCCGATTGGTATG
GATGGAAAGCTGGTAGGTGATTTGTTCAAAAACAGTGTAATTTAGGCCTTG
TGCATATTCAGTCTCTGCTTTTTGAATTTCTCatgctgaaatgtattttaatcacATATTGTC
TTGCTTCGGCACAATTTCAATCTCTGTACTATAGACTTCAAGGGTGCCAATA
AGCCACCTCAGTCTTCTGCCTCTACCCAAGCAGATACGTTACTCCTATTCTC
TGATCATTTTCTATCTGGGTTAGGCTGTAGGACAGGTGTTGTTAATTCAGGT
GCTTTGTCCAGAGGATGTCCCGGAAGGTTAGTGAGGTGGTGCCTCTGATAT
GGTATGGTGTTACTTCTGACAGCATATCCTTGGctcttgcattttctctttctgcaggcttttccttc
ttccatttttgtttctctacCTGGATTTTGTTGGCTAATTACATCTGAACAGCCGCATGTAT
CTGTATGCTATCCTAGATATTGTTTAAGAAGGCACTCTTTGAAGTTATTTTAT
ACAAGGCATTCTGGTCAGCTGCTCACAGTTTCACATGTATGAGTTTCTGctatt
ctctctttctctctttccagaaCCACCCTATTCTTTGATACCTTGAACGCATCTTTTGTCCT
TTCACTTACTAAAAACCTATTTCCTCTGCAAGTTCAACTTGTTAACCTGCAG

CATCAGCTTccattttttaagaatttttccTATGCTCCTGAAGTTCCTTTCTATTGCAAAGT
AACACTTTTGCAATGCTTGCTGCTTCTGGTCAATAGACATCCTTTAATCTTC
TCTTCTAGCAGATTAATAGCTTACCTGTTGACTTCATCTTGACCTGTCTAGAC
CGTGTatctttatcttcctttttagTGTCATTTACATATCCACAGTTGTTAGGTTAAATTGC
GTGTAAGTTGGTTAGCAACCAACTGTACTAGTCCTTCAGTCAAAGTAATTA
GAATTGGCTGCCAAACTTAACCCCTTTCTTTCCTGGCAAATAGCATATTAAG
TTGGTAAATGgaaatagagaaaggaaaactctTCAGAAGACTTTGCTGAAAACATGATA
GTTTCTGCAGCTACACATACTATAAGAATGCAGATTAACTGCATAAATACAC
CAGATAAGTTAATAGTGTTGGGACTGCTCTGTAAGTG
>TCONS_03237388
ACTGATTTTGCCAGAGGAACAGGTCCTGTGCTTCAAGGTGTTTAAAGAACA
CAATGAACTTGTAATTAGCCGTAGACTGCAGGGATCACGTTATCACACTCAT
CCCAGAGCACATGAGGGGTCGGAAAGCAATTTTGTTCTGTACCACATACAA
AAAGAAACGTGGGTttagcagctctcagcagcccaTCCCATCTTGTGGGGAATGACCT
CTCTTCACCAGGTTGTATGTACCAACTGAGAAAAGTCAGTACTTAGGCTCA
AGAATATCCCAGCTGGAAAGTAATTTGCTACAGCTGAATTTATTTAAGTGTA
AAGCTTTGAAACCTTTTAAAACGAAAGCCTAAGGTACAAGCTTTAAAAAA
GAGAGgatttaaagcattttctgtgATCTGATAAGatgcttttaATCAGTGATTCAGAAGAGTG
TGagaaaaagtgaggaaaatgaGGCACAGACAGAAGGAATGGCACAGTCATGAAGCC
AGCTCGCAGCAGAGCATAGACGAGGCCTAGGCTCCCATCTCTCCAACATCT
TGTGCTTATTCACAGCTGAGGACAATTGGCCATATGTGGTGAGTTCATGCCA
TTGCATTGCTCCACTTTCATCTTCCCTTAATGCCCAGAATGCTAAAATTCAC
CTTTGGTGAATCCATGAGCAGAGAGGTGCAAGAGGATGCTGGGGAGACAA
GCTGCAGGCTGACAGATCAGACTTGCCATTACTTATAGTCAACCACAGTCT
CGTCTCCCatggtgcagagcaggcagcGAAGGGGCCAGCAGTTCTGCCTGTGGCTGA
GTACTGTACACAGAACTACTTCAGAGTGTAGCCCAAAAGTCAGCCAAGGG
GCACAaaaattttgaaaggaaaattgcTTGACAGCCTACTGAGTTTGTTGAAAGACTTTT
TGCTGTAGCTTTGCACAAAGAAATGCACTTCTGACTCTGTAGGGTGATACA
CTAAACAGGTCCACTGCAGACACCTTTACCACGTTTGTTGTGTCTTATGCA
GAGATGTTTGTAATCGCAGCACTgtatttcatttaagaaaaagatattaATATTTTAGTGGTG
TTAGTGCCATTTCTGCAGTAAATGCTACTTTTTCAGTGTTCCACTTCCAAAA
GGATTTAAGAAAAAACTACCTGCAAGGTAATTATTTGTTGTTAATTATGTGG
AATATGACAGAAGACAGACCAGCTAGCATTGCGTGTGCTGAAATCTATTAC
ATATGGTGCACACACAGCGTACCAGCTTCAACATGTGCATCACAGCCCTCT
GTGTCCCCAGCAAAGAAGCTTTTAGCAGCCTCCTTCACACTTGTCCTCTaatc
tttctctgttttcacacAGGATGGGCAGCAGGGGTGACTGGCACAGCAGAGTCAGGG
TTTGCCAAGAACACTCACAGTGCATTAGCTCAGGCTCAAACATGCTGCAGT
GGCTTGAGAGAGTTTGCACTGTCTGCACTAGGAAG
>TCONS_03258277
CAAGAGCTTAAAGGAGCTAGAAGCTTCATCACAAACTTGAGTCAACACTG
ACCTATTTTAGCTCTCTCCATGCTGAACTTGATCATGATTGCAGAAGTGACA
GATATATCCCTAATTTTTAGTGAGGTATCCAGTCAAAACACTATAAAATCCAA
CCAGAACTAAAGGAAGTGCTTCTTATGCATATTCAGTGTTCAATTCCATTGG
GCGTAGTAAGTTACAGTGCAGGTTTGACATTTTACCACACTTATTTAAGGGT

AAGATTTataattcatctttctttcaccGATGAAAACATTTGAGGAATGTAAATACTGCA
GGGATATGAATTTTCCAGATCACTCATAGATCACCGGAATTATAACTGTAATC
ATATGCAATTTAGTGGACAATtctggtgataggtggacagttgtactaggtgatcttagaggtcttttccaac
cttaatgattctgtgactctatcTTGAATTTACCTATTTTCCATGTCTCATCTCAGAAAAGA
GTCTTGTTGCGACAGGTCAGCATTAATTTCTTTTAGGAAAGAAGTTTATAAA
AGGAGAGCTACTTTTAGCTATCCTATTTTCTAACTAAAATTTATTATATCTCTC
CTGCAGAATTGACTGTTATCCAATGTATTATTCACACATTCCAAGTGAGGGT
ATtagcttttcctctgcttaaAAGACAAGTACTCCACATAGACATGGCCAAAAATTTTAG
TAACAGAGCATAGGTCACAGGAATGAAGAGTTCATCCTTTTCAGGAGTAAC
CAAAGGAAGGTCTTACAGTATTCTTTCAAAAGGTATTTTCATATGCTTATTGT
TTTCTCCTCTTCGGAGTGAAAATACATTTCGGAGTGCAAAACTATTCAAAG
GGATGGAAATTCATGCTCCTTCATAGCAGTAATCTCTAATGCTGTTTGTTccca
ggcagcagggctggcaggatAGCTACTGTCATTATCTAGCAAACCATCAGGGCTGCGG
TGTCCCTGCAGCATACAAATAAGCAGTTgaagacatggaaaaaatggaaagttgTAAAGT
GATCTGGAAAGATATCAAGTGGTAGGTGTGCACACAGAGTCACTTTTATGC
TTatccctttatttttccctgtgttgaacagtgaaaaaaaccTCTTGTTTTCACTGCAAAGAAATATC
AAGGGCATTGAATAAATAATCACTTAGGGAAACTTTAATAAACACCTTGTTT
TGTGTGCTATAAACATGCGAAAATTTTCCTAGGCAGGACTTTATCATatcatagaa
aaataattcctgtcACCTGTCCTGAGCCACGCTGGTAAGTAAAAAGAAGGGTAAAG
ACAATTAGTGAGATTTACGagttattttcttaatgatAATCTATGTTCATTTGTATTTTGC
TATCCATTTCAGTAGTTGCAGCCTGTAATATTTATTTGCTAGAGTATGCACTT
GAAAGTGAATCAGCAGAATAATGTTATAACACTCTGCTTTTCATTCCTAGTC
TGGAACtgaagcataaataaataaataaataaataaatagcgTGCATTGGAAACAAGTGAATAG
AAAGACATTctagaaaatgaatatttataatttagtgtaaataaaatccaaattaaaataattttatatgtCTCATAC
TTGCACAGAAAATTTTAGctgtaataaaacagaaaataattctctgGTTAATGAGCAGGACA
AAACTTTCTTTAGGCTGTGGAAGTTGGACAGATGCATAAACCAAAAGGTTT
GGAGTGACATACATTGACTGTTTTCTCACACCTGTGAGATTCTCACTGAAA
CCTGAAGTCTCTGAAAATCCTCTGGTCAGAGGGAGCAACTTTTGTGTCAAA
TTCAGAAATTTCCAAGTACACAGGCCAACGAATGTGTTCCAAAGAAGGTAT
CAATGTAGAAATACTATCAAGGGATTGAAGGCTTcagagggaaacaaacaaaaacaaaca
aacaaacaaaaaaaagaaaaacacaaattgTCATAATGAGAAAGTAATAACCATACAAGTAA
ACgtataaataataatataaaaaatatgaCATAGAAGagacagtgaaaagaaatgtagTAAATAATAAAC
CTTTCTAATCCCCCCTCTGGACAGAAGGTCTGAATGCATGTTTTACTCTCAT
TGGTGTGAAAAGAATATCTATTTACATTCATGCtcataaataattaatttcaatgGTGGTGT
CACCTGAAAATTTGCACTTTTAATATACTTGTTTCcaataacaaatgaaaaactagcaaaca
aacaagcaaacaaaaataattctgtattttgcatTATAGGAGCAACTGTATTCTAGCAAAAACAG
ATCTTTTTCTGTTAGGATTAATGGACTACTATAATATTCTGGCAAAAGCCAAA
TTTCAATGCTAACCTTTGTCACCTATGAaatttttaaaggcttttacTGTATTCAAATATA
GCATTCAACTAACATTATTCATGCTGTAGTGAATTGTTTCTTTGACCTAAATC
AATGGCTGATATGCCCTTCTCTGTCTCAGAATATCATCTTTCAACATCTCAGA
GTGTTCTACTGAAATATGCACCTCTTGTCTTTGTAATTAGATACTATCAGACT
GGCAAAGTATCTCCTATCCACTAAGGTAGCTGAGGAAGAAACCTAGAGTGC
ATTCTAAACCGTGCTCCAGAGATGTTTCAaaagaattggTTCCCAGACCAGAAGA
CCTCTTGTAATGAACAAACCTCGTATTCCAAAATAACATATCTGTCCTTGAC

AGCTTTCCCAAATACCCTCCAGCACAGACTGCTCTTCAGGAGATGTCAAAg
agttttcatttgaaatcagaaaaaattGTAATACCACAGCAATGGAATAGAGGACACAACAC
TGCCAATCCCTGCTCTTTGGAATGCTTCAGATATTTATGAAGTTTATGAAGG
GTTCATGAAGTAcaaaggctgcagctgagctcacCTCCCAGTGTTCCTGTTATCGTTCAT
CCTGCACACACCCATGAAGCCATGCTCTCGGTGACCCCACTTCAGATAACA
GAACAGACCATGCCCTCAGGTTCATGGCTTCCACCAGATACTCTACCGGCA
TGGTGCTGACTTGGAACGGAGTGCAGTTCCTAGATAGCAAAGCATAGTAAT
GCTGGGGAAGTCAGCCAGGGCAAAGTATGTTACAGTGTTCAGAttagtaggaaaa
taaaaaaacttcAGTATCTGTCCATGCCTAATTGCATTTTGTTTATCAGGCTTTTGAA
AGCCTATCAATATTCTCTTATTAGTGTTATTATTCCTTTCTGCAGAAGAGAGG
ACTACATGAAACCCTtaaaatcagaagcaaaaaaattaatttccatgAGTAACTCTAATCTAGC
TTGCAATTTCTGCGGAGGGGATGTGTGGGGGAACAAACCAACACTGTGATa
cagtgctgttttctctctttctctctcttcctctctacttctctctttttatatCTACCAACTCAACATCTCTGAGA
AATCATTTCcagagaaaaagagcatttctgGGAAGATAGTcttggaaatcacagaatcacagaattgtagg
ggctggaagggacctccagagtccaaccccccctgcaaagcaggccgTTTACAGCcggctgcacaggtaggcatc
cagactGGTGTTGAATATctacagagaaggagaatccacaacctccctgggcatcctgttccatcACCCTtg
ctgtgaagaagttccttcacatattGGTGTGGAACTCCCTATGCTAAATagccgtttccccttgtcctggc
TCCACAGACCAttgaaaagaggttggccatgtctcTTTGAATCCCACACGTAACATattaataa
acattaataagaacacctctctgtcttcttttctcaaggctgaacagactcagttcactcagcctttcctcataggggagatgct
ccaggccctttatcatcttcgTGGCCTTCTATGGACTGTCTCCAGGAGATCCTTGTTTTTT
TGTACCAAgcagcccagaactggatacagtactccatgtgaggcctgaccagggc
>TCONS_03258300
CTGACTGCCCTGCTGTATGGTTCTTTCAGAAGATCACCTATAGTCTCCAGTT
AATAACACACAGGtacactttttttaatgtagtggCCTTTCTGCCAGGCAGCTAAATGCCT
AGTGACTAAATGTCACCTCTTTGAATGCAAAGATATTTCATTGTCCAGAACT
GTTCCTGGAACACTGAGCAGCATAACGATCCCTGATAAAACAGGCACCTGG
GACAGCTGATATCACAGGGAATTTTTATAGTCGCACACAAATTCTAGAATCC
CATTGTAGGGTAATCTCCAAAGACTTTCATTTGCTGGGttcaaaatgtcttttctaCTAT
TCTAGCATTTCTTTCCCAGGACAGATAATTTGTGACATTTCCTCCACAATTTT
TTCTGCATCCTATGTTTTTGGGggcagattattttctttattttcagcaacAAAACCTGGATAA
GAGCAATATCTGAGCAGAGGTTATGGCAGATGAGATTTATGAAGGTGGTTAT
AAATATGATATGGATGTCCACaagcaagagcagaaaaacTCTGATCTTCAGCAGAGC
AACCACATACGTTGTGCCATGCCCTGGACTTAACATAATGTTGAGGATCTTG
ACTCCTACTGAGAACAGTAAGTCTCGTTGATAGGAGTCTGAGTATTTCAAA
GCCAGCGTGGTATTGCATACAGCATTGACTAGAAGTGGTGCCTGCTGTTAA
CTTTCAAATCACCATCCCAATCTTTCTTGATGAAAGAAATTGAACTCTAGAC
CTCCtaccaaattttatttttgctgtttcaatcctcttttcctttaaaaatatcagcAGCCTACCAGACTAAAGT
ACACAGAATAGAGGGTCAAGCAGCAGAAGGAGGCCTTTTCTACTCCATCC
TCAGGCAGCTTGACACTGAAGCACAACAGGGGTTGCATATAGGGTGCAGT
ACAGAAGTGAGTTACCATTTACATGGACAGATAGGATAAGGTTCATGTCAG
GAGTTCTAAGAGTATTTTTTGAACTTAATATATTAACATTTGACAAATATCAA
TCTGTCAAATTTGTAgcaaaaaaacaatgcaaataaTCAAAGAATGCCTTTCCATATTTT
TACTAGATCTGTATTACCATAATTTGTCCATTCAATTcagattcattattttctgagaCGTA
ACCAGGATGTCAACTGTGATTATGgagaatattttaagaaaacactAAAGTTGAAAAG

ATTACAAATTAGTGTACCTCTAattacatttctatttcttcagtCATTACATAATATGGTGCA
CATGAAAATGAACCGCAATGAATTTCTCAGATTTACTATATTTGGAATATAGT
AAGTTAAGTTGATATAGTTTACAAAATCATGAtatattactgaaaatacataTGTACTGT
AAAATTCAACATGCCAGTGATCCCTTAGAGCATGTGGTATTAACATATTTCA
TGTGTCTATCACAACTTCTTTGGCATTTGTTCAAATATTTGACAAAATGTTTG
CATATATGTTAGTACTGCAGACTTAGTACCATATTTGCTGTTCTTGCACCTTT
CTCTGTCAAATGTCACCCAGCTTAAAAATTTTCCTCTAGATTTTTGGATAATA
CTGACTAGAGAATAAATGAGAAAGATTAACTATATatgaaattaaaggaaattatTCTGC
AAGTGATTCTATtaggagaaaacattttgtaataATCAGTTTTCTAGATACCTTTTAATTCA
ATTCAATGTTGACAGCAAATGGAGTAGTCAGGATGGAAACATTTGCAATCT
GAGCAAAGCTCCTTCAGCCCTGattctgatttctcttttgctctgAGGCACTGCTGGTTTT
GCTTCCCATCCTAGCCTTCCCACTTTgctcactttccttttctgctcgTTCCTTGGTGGAGT
TTACTAGTTGTTCTTttatagcaaaataaaattaattaaatcttCTAGCTTGTTATCTTTCAGTG
ATAGTTATTTGTGTTCAGTCAAGTTCTTGTTTATTAACtgcacataaaaaaaatactgagttt
cTCTGACTGGAACAAAGTAGATCACAGACTTATTTCCTAACATATTTTCAAA
CTATGCAATCTGCTGTTTCATGATCAATGGATTGTGTAACACAAATAAGTGA
TTTCATACTTCTCTGATTCCCGTAATGATATAGTTTTATTATACCAGAATTATA
TCCTCAAACTGACTGCACAGCTGAATGCGTTGTCTTGAGTAACACAGATAT
TCCTAAAATGAATTTGCTGatattttaagtgttttcttttgtagtttGTTAATTTAAGCAATCTGC
CTTCAGCGTTCCCAAGATGCCAGTACTTATGGGGAAGAAGCGTTGTATTTCA
TTTGATCTGggctttttttattgttatttcttttgctggaaTTAAGCAAGGATCAAAATTTATGCAG
GGATAAGAAATTACAGGatcttttcaaattgtttttgaGCTGAAGTAATAACTGCAGTTAat
actgtttaaatatttataagagAAGTTAGTTTAATCAGTGTGACAATGCAGACATGCATAT
TTGTACCTAGGGCCAACACTGAAAATAGCAGTTGGTATTTACGTCTtccttaatttg
ttttcatttggacATTGCTACTTCATAGGTCTAATTTACCCCTCAAGCCTGCAGTTCT
GAAACATAATTGTTGTAAAACTAAAGCGATTAAGTATTTACTGATTAAGtaatat
atttcaaagaaaatagttaCCTAATCATTAATTAAGCTAATAAGCATTTTACTACTTCTGA
TTTAGTTATGACTTTGCATAATTGTTGCTTACAATTATAAAGCTTTTAGTTATC
ACTCTACTGTTCTGTAGATTTCTATAAATTTCATGCAGTTACTATTGCTtttaccttt
tccttccctGGGCTGTAAGTTCAACAGCCTTTACCATTCCTTATGGACCCAAACTT
GAAAGTAAGTTCAGTGTTTTGCTGGAGAGCTCCATACAGCCACCCAGTGG
GTGGATCAGCATCCCAGTCCACCACTCATATTCATTACTGTCTGCAGATGCC
TACACTACCTGTGTCAGAAGATTTACACCCCCTTTTGTTTCTAAACTGATTTT
ATACTTTATTCTCAATTTACTCTATTTCCAGACCCTTCTTTTTGTCATTCCTGC
AccttattttctctgttcaaaCTTTTCCTGTGAGCCAGAGATGGACAAGGTCATTTGGTT
GAAGGCACATTTTGTTCCTCTTCAGCTTGGAGGGCAGTGGACTTGTTCTGT
ACCTGTAAGCCTTAGGCAGGGCCAgagccttcctcttgctgccAGAAGTAACCAGCTT
CCATCCTTTCCCTCTCCATAGGTTACACTTACCTGAATGCTGTGGGTGGGTA
TGGCTTGTTTCATTTAGAGTTCCTGATGATCTTGGACCTTGTCCCCAGAAGG
TCCACAAGCTCCTAAAGCAGAATGCACAGCCTGCTGAGCAAGATGTCCCCT
ctatttttgtctctgttgAACTTGTAGGATTACCCTAAAGAGTGAGTACTGAAACCCAA
TGGACTACCCAAAGGGTTTTTCCTAGTACCAAAAACGTCTGGCCTTGTaattttt
tgttggtttttccccAGTGGTAAAATTTATAGTTTATTTATATTCAGAATACAGAATATT
GAAACCTGGAGTTGATctacttatttttctcttgggCTGTATAACAGAAGATTTTGCAGA

AACCTGACTAACCTATTGATCTCTGAAAAAGAAGACAGCCACAGGAATACA
AGAAAACCTTGTCTGTAAGACAGAGTAGCCTCCTGAagctttgcagtgtttttttttttttcaa
cccaAATTATTTATGGAGCATCTTGTATGTCAAGCTTAAAGTCCTATGTTCATC
AATATTGCTGTTCTGTAAGCTGTCCCCATTAATCACAAAGTGGTGATTTGTC
TAGTGCAAAGTGCAACCTAGGGTGTTGACAATGCTTAAAATTTCTCTTACA
GCTTATTTGTAGAGCAGACAGGATCAGGCATCTTTCTATCCACCATATTTTAG
GGATAACCATGatacaaaatacagaaactgcTGATATAGGGCAGAAGTAGCATTTGCAC
TTAATGTCCAGCAGCAGAGTCTTTTCTACATGCTTCTATTTGCAGTTCATCCA
TTAGGATCTTTGCACAGATAGGAGAGAATTAAACAATAGTCTGAAGAATGT
AAATATGCTGAGGGCAAATAGAAACCTGTGGAGACCTATAAAAGGAAATCA
CTGCAttctcattcttcattttatcaATGTTTTGTTTACTATGCAAACCTAAGAGAGGCAG
AGGTGATAGGGTATTTGCTGAGGAATTATTCATCACCATCTGGTGTCTTGAG
GTAATTAGTCTGCACTGTACCTCCTTAGGAAACCATAGCAACTCTACAATAA
CTTAATTGCATTCTCTGATTATGGCATAAAAAATAAGCACCACTGCTGTAATA
TACTAATTAATGCATTCTTAACTGCCCGACTACTGTATACAAATGCACTGTCT
GTAATTGGCTTCTTGATAAGAAgtgtttgcaaaacagaaaaagattcaAAAAGAGCATAAT
TTTGAATGATATCAGCCCTGGATGAAATCAGAGAAAAGCTAGTGCAACATT
CAATTAAAGATTGATGTTAGTTAGATTCCTACAATCAACATGGTTTTATGGG
AAATAACTAAGTTTTGACAAGCAAGTCCAATTGCATCCTTTAAGACTGTAA
GTTTTTGCTGATTAAAGTAACTCAGCAGATATATTTCCCACAGTTCTGTAAG
GTATTTGACACACTGTAATGTGACAAAGGATGAACACAAATCAGAATTACA
GATACctattg
>TCONS_03293244
CCTCGACGGCCGCCGGGGCCGGCGGCTCCTCGGGCTCCCGCGGCTCCTGG
AGCTCCCGCGGCGGCGGCTCGGGCTCCTCGGGGAAGGCGCATTCTGGCCA
CTGCTGGAGCACACTGAGAGAAGTAGCAATGAGGTGGTTCAGCTGTATGA
ACCCGAGGTTAAAAGAGAGAGGAGCGTGAGGGAAACCACGTTTGATTGCT
GAACCAAAAggtgaagggaaggaggagcGGCTCACATCCGAGGGGCAGGAAGAGT
GAAGTCGCCTCCACTTCAGAAGAGATCCTTGTTTAGAGTACTGCAGTTTCC
TTACATCGGGATCTTGGAAGTTCATTGATACTTCACTACTGCACTGCATACA
GATACTGCATGCCGTATCTACAGCACGTACAACAGCTGTGCAATAAGTGAA
GCACTTCTAAGGCCAGCTGGGATTTGGACAACTACACTTTGCTTCTCTTAA
AAAGTTTCTATAAAAGAAAACCCTGCTGTTTATAACATCACATTTCATGCTG
CTTATGGAGTACTAAACAGTCAATACTGTGAGTCACCTCTTGTTCCCTGTCC
AACACTCACAGCAGAACAAAACTGCTCTTTACGACTGTTTCATCTTTCCTAT
GATACTGGAGATAACTGTTTATTCTTTCTGCTTAACGGGATCCTCAGAATTC
CTCATGGATGACTGCTTCCTTACGTCAGCTGTCTTTCACCTGACACCTTCAG
TGAAAACGCACGCTACCTTGGCATTAAAAGCACATATTCTAAGTTATGCACT
ATTTCCTTCTATTCTCCTGCAGACAAACAGTCCGTGTAACCTTCCAACTAAT
AATTAGCTTCTCTTGGGGTTGCTTTCCAAAACTGTGACGTTAAGCAACTTGT
ACAGGTGGTTGCTAACGCAGAGGTTAGACCTGAGACCAAACTAAAAATCG
CAGGAAGGAACAGCACAAATATACAACGTACAGCAGTCAGTGTGCAAGCT
GAGCATTTGGACTGCCTTTCCCACACTCACCTGCGGACAGGCTGTGCAAGT
TTGCTACGAGGCACTGGGATGCCCCCTGTGGAAGGGGCACTGGGTCTGGG

CAAACTACTCCTCATCCCCACTGTCCCTGCCGGCCTGGTGAGGGTGGTGCT
GTTAACACCGCTGGCAGGGCCGGGCAAGCCTGagctctgagctgcaagagGCCCCG
GTGAAGAACTGCTGTGTGTCAGCTGTGTGGCTTCCAGAGGAGCAACAGAA
CCAGGTCCGTGGTTACTAAATGCCTTCACCGGTTGTCGCAGAGCGAGGGG
AGATGGTGCTGCAGGTGAGCGGAATTTACTTGAAGAAGGTACTGGAAAGT
AAAACGCAGCAGTTACTTTCAAGCTTAACTGACAGTATAACTGAAATTAAA
CAGGCAAGGATTTTATGTGAAAATTCAAACAACACGGAATGTGTAATGGAG
TACAGCCAATCAAGCAGCAATGAGAAACAGGAATTCAGATGTCCTCTTTGT
CGTGTTTTTGATATAATAACTCCAATGCTCCCTTTACCTACTATGAATATTTAT
CTGGATTTGTAAGTAATGCCAGTAAGGCCCTAACCGTTCTATCCACCAACCC
AGAAGAAACAGAGGTGAATATATTTGTAAGAAGAAATTCAGCTGTTTCAGA
AACGTGTACCTAATTCCCATCCCTAAACCAACACATTCAAGTATATGGGAAT
CTAGTGTGACATTTCTGCATATTTGCACTTCCTCAGGTACAGGGGTGCAGCT
CAgacagagcagggcagcctAAAATTTCTTTTATGCTCTTCTTTTTAACCCAATCCAC
TGAGTCAGGATTCATCTCATGATAGGGTCCAGTTCAACATCTACAgtctgaaggca
aaaaaaacccaagtaCAGTCAGCCAACTCCCAACACCAAAGAGATGCAGCTCTGA
AACTTAAACCGGACAACTCAGACAACTTATAGCCTTCCTCTTCTTACAAAC
ATCTTCTAAATTACCAGTTTTACTTAGAGTTGAATTCACATACTTTGTTATCA
GTTAGTTAGGATGTAAAATGCAGGTCTTTGTCACTGGTTTATAAACAATTCC
CAGGGAGGATTTGATAATCTATTTATAGGCTTAGACTCAGAATTTAACACTC
TGTATTAGCCCTTAATTCAAGATGCACACACAAATGTATGCCATTAGTATTTT
TTTGGtcctatatatttattttactcaaTGCAGTAGCAACAGTTCTTACTTATTATATACACA
AGAATTAATTCTGAAGTTTCAAAGACAGCCAGCAATTAATGACGACCGAGC
CTTGCAGCAAAATACTGACAATATTTAAGTCCTTTGGTAATCCTAAATCTTA
AGCTCAATTCTTTATGagtatcagaaatattttaaatatatgcataaCGTATCACTAAACAGAAT
TCCTGAACTAATCGAT
>TCONS_03313496
TCTAAATGTAACATATCTATTTAAAACACAATATTCCACTCCTTCTAGGAATA
CATAAGAAATCAACTATTGTTTCAGATCATATAGTACCACTTCCATATGGAAC
TGATTTTCAAACGATACTTGAACACAAACATTTAGAAATCCAGTGACTAATA
ACATAAAGACAACGAGCAGATTTTAATCTTACACTGctactgctggctgcagagagaTT
TCACCCTTTTTCTAAACTACTGCATATGTAAGGAAAactataaaatgttttaattttttcttttc
taataacaaagaaaataacgACAATAACCTCCTTTTATTATCACTGGAATGCACAACAC
ATAGCAGAagggacagagaagaaaagtggGTACaactttaaaagcttttccatCTAATGATTTTCTC
TGTTATTCCCTCCTCCTTCAATGTATTCTCTAGTTCTTTACCTGGCCAACCCT
CTCCACATTGAAGTATTTTCCTTTGCGATTGTAAAGTTCTGGcgcCTTGTTCA
GCTCTGGAAGCACATGATCTTCTGACATCCTCAACATggctgaggagaaaaaaaaaaga
tattcttaATGCATGTGGGTTTCTTTCTTAATATTCTTAATTTTCCATTGCTATATAA
TATGACACTGCTGATAATATAGGAAGGATTAATTTGTCAGGAGTTAAAGAGT
CTGGAAAGAACAATTATGAATTGGGACTGAAATTAAGCTTTACTTGCCCTG
CTAAATTGCAGAAAATATCAATGATAGTAATAAACGGATGGATATAGCTAAG
ATTGGCAGAATTAATTGTTGGCATTTTGGCATTTACAAAACAGATACCAAAT
GTTTACTACATCATCAAGCTTACCAACATACAGCCATcgaaaaaatgctttgaagtttTTC
ATACTACTGTCAATAact

>TCONS_03323652
tgGAATTCATGTTGCAAGCAGGTTGCTCATAGAAGCCAGGTTTGAGGTGGAC
CTAAGAGCTGATGAAGCTCTGAAGTGTTGCTAAGCCCCTGCCTGTGCCACT
AATACACTGGGGTTAGTGGGGGCATCTCAGCCCTCCTGGAGTCTGAAAATA
AAGCTGGTAAAGAGGCCAGGCATTGTTTTTGAAGTTATCTGTAGTTGGATT
GACTATGATCAATCCTaacagcagaggcagagaaaatTTTTGCGTTGGCTATGGAGAAG
GTGTGAATGGGAACACGGGGTGTGGCAGATCTGCTGCAATGATGAATATTT
CAGGCACTGCATGATTCCacatcagcagctcagcacagtggTCCTTCATCTAAAggcttctgca
tttcttctaaCTGAAACATCTTGAGAGCCAGGTATATATGGAGCTGTTGTTTAcagaaa
aaattcttttcagaaagagtggtcaggcactggcacaggctgcccagggaggcagtgaagtcactgtccctggaggtgttc
aagaaacatggagatgtgtcACTGAGAGACACGGTCAGTGGTCATGCtggggatgggttggagtt
gaagtagatgatcttagaggtcttctccaaccttaatgattctgtgattcacaagTTAGAAGGACTAGAAAAT
CTCATAAATGTGTATCTTCTTCTTCAACATTTATTATCAAATCAAGTCGTCTT
GAGTTGTGCCTCAGGTTCCAATTTTGATTAAGATTTTGCACAGTTCTATTTC
CTGTTTCCTAAGACTTTTCCCCACACCACGTTGTGTATTTAAAGACCTATTT
CCAGAGAGTGTCTTGGTTAAAGTGCAGATGCTGCCAGACTACATGCTTTTC
AGGTACTGCCTGTGTTCTCAGCTGCTTATGGTCTCCTAGAATTCAAACAGAC
TGCAGTATTCCCTCTGTGTCATTTGCCCCTCATTCTTAGATAGCGGCAAATTA
AGAAATGGTGGACAAGAGTCAGGAGTGAAACAGATAACTGCTTCCACCAT
GATAATAAATGCACTATTTCCAGCAGCCTGGTCCATTAAACAACCAGAAGA
GACAATTGCATCTTTGTGGAAAAACTAGACAGTTTCCAAGTTCTTTGTTGG
CGGGCTTTGACAGATCAATTCATATtctcactggaaaaaataataaaagagaaagctggaaCCAA
CCAAGCTCTTTAGGCCAAAAGATCAATTTCAGGTCTAAAATTCTAAGTTGA
AGAAGAGTAATATACTTAGAGGGATGCTGCTTGTTTAAGGAGTTTGCTGTA
GAAGTAccactggaaataaaataaccaTTGGTAAAGATGTAATAGTAATGGCTATAATTA
GGAAATTCTTTGTAGAGCATTAACAGTGCTGAAAATGTTCAAACAGTTTTT
CAGTCAATGGGAAGCCCTGTACTGGGAAGCTCACATTAGCTTGGTAGGTGG
AACCGACACCAGGAGTGGAGGAACAAAGATGAGGCAAAGCAGGGATGGT
TTCTGTGCATAAAATCATTCTGAtaagtattttgaagtagatcCATGAGGAGCActctcagcag
caccagtgCTGGGACTGGTCAGAAAAGCAGCTTGAAAtctgtagttgtttttttcctttgtctgaa
AATCTTCAGAGAGCTCTGCTAACCACTACTCACCAGCTGGCTTCTCACAAA
ACTCATAAATGACAAAAGTTCAAGGGATTTGAGCGTGTTTTGCCTcacacacaag
cacacacactGGTACACCTGCAAGTGTTAGGCACGTGCATATGTCCAAGCTGACT
AAAGCCAGGCCAAATCCTGTCACTGgctttccccttcattccccaCTCTTTGTATGCGAT
TAAGAGGAtaccttccttctttttaagtTTTGACAAAAACTAAAGTTACTTAAAGTTACT
GAAGTCAAGAGAAGTATTGTTATCAGTACGCATAGGCTTAGGATGTCACTC
CTATTTTTCTAAGCCTCTTGCAGAAAGCTAGACAGCTTGAAGCAATGGAAA
TAATACTAAGAACAGTGAATAATAAAAGAGTGTCTCCTTTTATCTGGATCTG
ACAAATTTAACATGATAGAACTTTCCATGCCTTGCTATTAGGTCCCCTTTTGT
GAGGGCAGTATTGTGCACTAGGATTGTGCTCCGTTCCCATAACAAGGGTGA
AGGTGACTGGTAGGGCTGTTATTTTACAATGTGTTTATCCACTTCAAAAGAG
TCACCTGCAGTGCAGAAGTGCAAGAGTTTGCACACGAGCTCTCGGTTCAC
CCAGAGGAGTGATGTTTCACGTTCTTGCTCGTGCTGTCAAACACAAATCAT
CATGAGGGGGCACTAAAGAGCTGTCaataaagaagttattttgagGCAAGGTTTGTAG

GGAGAGgtaattatcttttttagATCACCAAATATTTGACTGTAATCTGAAATAGGCT
GATTTCGAAATGTCTTCAATTCG
>TCONS_03406660
TGACAACAGAGCCCAGCCATAACTGCAGTGCTGGCTTGCTGTGACTTCTTT
TGTACAGCTGCTTGGCACAGATGAAGGTAGGGTAAAATACAGGCTCAAGA
AGCAGCAAAGATCCTGTTTTTATGGTGTCTGTTCTATGTGCTTGgtttctcctcctgc
aggacAGGAGGAGAAACTCAACTGAAGGTGTTTGACAGCGGTGTTCTGACT
GATGACATTCCAGCTTCACCCAAGGACTGTGCCACATGCCATCAAAGAATG
GGTCACATACAACTGTGCCTGCTGTTAATGCTGCCAAGCGCTCTGGACGTC
TTCTGTGCTTCCAAATGCAACAATGCCCTGTAGGGACTGAAAGGCCTCACT
TAGCGTATCTTCCCGGATAGAGAAAAGGGAACCTTCAGAACGAAAATGAG
ATGTCTCTTAAGGAAAGCGTTCTTCCCTCTGCATTACTGAAAAGCAGGAAT
TGTTTtaagcagaacaaaagcaataccaaaaggaaatgtttgcaATATTCAGTATTCAACGTTGAC
GCTTTCAAAAGATGTGTTTCCTTGCGGAGCCAAGCAGTTGACTCCCTGCCA
CTAATACCATTATTTATAGTGTGGCACAGGCAAAGGGACTTAATCCAGCAGA
CCCCAAATAGggCTTCTTCATTCATAAGAAATATAAGGAAAGTGTGTCTGTA
CAGCGTCTGGCAAAGTAGTCCTCTGATCCCTTGCTGGACAGGGAGGAGCT
GCCAGCATTAAGATGTGAGGTGAGGGCACATGGATACCTGGATCTGCACAA
TGGAGAAGTGGAGGCATTGGGGAAACTTGTGTCTTTTAACCCAGGAAACT
GCAGGTGTAAGATGCCTCAAAGGACAGAAGAGCAAAGGATGCTGCAACA
GCAGACTAAAGTTTGGTATTTTACTATTTCAGATGCTCAGGTGGACTGGGTT
CTGTTTTTCGTTCAAGTCTCATCTTTGTCCCTACTTAGATGACTTGATTTCTG
CTGagtatttcccttttcttttacAGCTGTAATGGAGCAACTGTATGACAAAAAGtcctgtatttc
ttcttctcgTCAGTTCTGATAATGCAGGTGGGCATTTGAATTAAATGCCACAGCAG
CAGTTCATAGAAGAGCAAAGGTGATGCTGGGTTTCATTCCTCTTTGCCTGG
GAAGCAAGCTGGCATTGGGCCTGGGCCAGCTGGGGTTTACCAGGTAAGAA
CTGCCCATTTTCCAACTCCCCAGGCACTGTGCTCCGTTCGCACTTTCCTCCC
CACAGGGCACATCCCTGAAGGCTGATATTTCATATCACCACTTTGCCACAAC
CCTCCATGACAGGGAGAAAATTTCAGTCGTTCGCCTCATTTACTTGCAAAC
CTTTATAAATACCGCAACTAATAGCCTGGGTAAGTGGAAGATTAATTTCCAT
ACCACCTGGCGGCAAGCTGTGGATTAGGCTGTTGTCCCTCTTGTGGGTTTA
ATGTTTGAGGAGCAGAGCACTCAAGTGCCCAGACAATCAGCCTCTTGTTTA
AACAATGAGCTTATAGGTCTTGTTAGCCAGTGctcttaaattttcattttattccgGTATGCA
GGGTTGTGTGATGTGGAGAATTGCTTCAGTGCAGAAAGATTGTAAACCTGT
TCAATTAAAGGAAGTGCTCAGATCCAGCTGTTGGCATCCCATGAATGCCAG
TGTTCCTAGCAAAGTAAATAAGGCAGACACACTCCACCCTTGGAACAGATA
CGAAGATTTGCGCCTTAATTCTCCTGAAAGAAGAGTCTTTGTTTGGAAAAG
ACCGTTAGCAAAAGTAAGAGGAATGCAAAACTCGATGCATAAAATACCCA
GTTAGACAAAGTCTTCTGCAGATAACAGAGAG
>TCONS_03406688
TCATCCCATGAGCACGAACTGTGTAGAGACAAGATTTCAAGACTGGACCGT
ATTTTGGAAGACAGTGCCTGGAAGTGCcaatgtgaggaaaaaattaaataaagttAATTGTA
GTAGACCACTACAGGGTGCGAAGGGGAAATGCTTGCCAGGCTAGGTCTTT
CAGGGAGCTGGGACAGAGAACCTAGCAAGCATTGCTGGTTTGTGCTGAA

CAGATGGCCACAAGAATGTCATGCTCATAGAAGGGGATTTCCAAAAGGGC
AGAATTTGGCTCCATTTCTCCTAGCTTGTGGTATCTACGACAGTTGTGGGGC
TGTGGTTTTAACTTCAGAGTGAAAATGCTTTGTCTATAAAGAAAATAGTCCT
CTGCTAGGTTGTATTTGATAGTGAATGAATGTGTAAAGACTATGAGTggcatgta
atttttttttcctttctatggCAAACCATTTCATGTACTTCTGCCCTAATTTCCATCTACCTC
CAGAGTAGCATTTGACCAGTCTAGGGCTGTGATGATGTTGTATAAATACTTT
TAAGTGCTTCTAGAAAGCAAGTTCTCCGTACAAAACTATGGGAAATAAttcaa
gagaaataaagtaGCATAGGTGACTTTCTTTTACCAGACAACAAAGCCAGATATAA
AATCTGGGGTTTCTAATAATATATTTCTGATATATACAGAGAATAATAATTTCT
TAGTGCTTACAGAACACGTTTTCAAGCAAGGTTTAGTATCAAACAGAATGG
TTTTCTCATACTGAGCAGCCAACTTTTCCGGGTACAAACAGCTTGCAGTGA
TGCTATAGTCTACAGTAAGTTGTGGCTGCATACGGGAGGGAAATGTTGGTA
CCCCTTTGTGTAGTAACACTATCATGggctgctgcttcaaagaagtTGTCATCCTATCTC
AGGCAGTCagaggtggaaaaaatgacaggtCAGTCTTTAAAAGAACATCCCTAAACTC
CTCGCTGGTTGACGTGACGGGTGCATGTTTTAGGGGGTAGGTATGCTCCAA
AAAGTTATTGTTGACATATATGAAATATAGGGCTTCTGCCTGAAATCCGCTC
CTGCCCATTGCATCTTCCTCTCTAATCCACGTCCTCTCGCACCTCATGCTTTC
AGCAGTACGACTTGTAAagtttgttttgatattttagCATTCTCTGAAGGATGGAAGATC
AATAAGAGTACAAATATGCAAATTTAGTATGGTAAACTACTTGTGCCTGCTG
CTAGATAAGGGCACGGTTGCTTTCATAGCACCATCCTAATCTCTGCACATAA
GATGAAGTAGTGATGGGACTGGGCAGCACACAGGGCTCGTTTCTGCTAGTA
TTCTCCAAAAAATCCAGGATGAATAACAACTATGTTGTCAGTGTCTTCCAGT
TGCAGGAAGTATGCATGCTCAGGAAAGAAGTTTGGAATTGGTTCTTTCTCA
GTTGTAAGTTTGTGggattttgttcttctttttttttttttcaaaagtaattCCCTTCAtttaataattgtttttaaat
atcattcATTATTCTCACATGTATGTTTGTCTCTGGCCTCTTCCACCTTcctgttttatatat
ttaacCCCGTCATCGTGGTTCTCTATCATCTTTATCACTACAGCTTCtcagtaatggaaa
aaatatactGTATTGGGTAGTCCTTTCTGCCTGAGACAGTATTATTTTGGCAAAAT
GCGATCTTTGACTCTAAAACCTCTCCCTCTTGCCAAAATCTAATTCAAATAC
TGGTGGTCTGATCACAGTTCACAGTTAAAAAATTAAAGGTGAGCAGGACT
GCCCTCAGGCCTCATGCTTGTCttcgggggaaaaaaaaaaacagctcataAATAGTGCAGG
CTCTCCCACCCCGCTCTTTAGCAAACCTCGCACATAGCCCTAGACCTACTCG
CATTTGTCACAGCATGTCCCAAGAGCTCAGCCAAGGCAGGAATTCAGCTTT
ACTAATCCCAAGCAGCTTCCTGCAAGATCAAGTATCAGGGAAAGAAATATG
TTCTTTTATAGCAAAACTAAATTTCTAACCCCTCGCCTCGTGCCAGAACCAA
TGCTGCCACTCGCTCCAGTCTCAGTGGTGAGTCAAAGGTGGCAGATGTCCC
TGCTGCCTTCAACCTTACTCCCCCATTTGATCTGGAGCCTGCAAGTTGTTAG
TGGTAATTCATGCCTATTAGTTTCCCAGCCCTAACCTGCGCTGTATACACGA
GTCCCTCAGAAGTCATCTAGATGCAGAGATAGAAttgtttttatgctttctgGAAGCAC
TGACTTTACCAAATGCTGTTCCATCTCTGCTAGAAATGATCTGAGGCTGGAA
CTGTTTGAGTTTGCTTTAAATTAGAGTTTGAAGTAAGAGTTCACCTTTCACC
TTTGGCCAAGAAGTAGATGTATTCTGTATTTGGCAACACTAATTATTCTACAT
GCTGATACTGTTGTAAAGTAATACCTTCTTCACCTTATAAAGCTCTAACTGTT
GATATTTGGTATTGTTTTTCTAAGGGTAAGCCTATGCTTCAGGCTGTACTTAG
GTCTGATTTGAGAAATAGAGACAACTTGTTGATACCTGTTCATTTTAGTCTC

TGGGAGCtgggacagcagagctgagtgctATTTCCCACTTGTGTAGCCTTTGTTGGTCA
TTGTTTGGAGGCAAAGTTAGAATTGAACATCTTAATCTGACCTAAATAAAA
GGGACACACAATAAAGTCAATCCTTCCAaaatgagagctgctgctggaatcGCTAAGTG
CCTCCTTGCTGCTTCAGACCAGGCTGGGGACAACTGGCTGGCTGTCCTCTG
GCCTTGGTTCAGTAAGGGTGGTGGTCTGTCTGGCTCAGCCAGGTGTGGGTC
TGGTGCCTTGGAGCAGTACTGAGGCAGTTTAAGGGTAGATGTTAGTTGCTA
TGTGCATGGACTATACCTCTCATCTTATTTAAGATGAGTAGCAAAGATAGG
CAATAAAGTTGCTTCAGAAAATCGGTGGCAGTGAGCCTCTATAACTAGCTG
ATCTCCTGACATTTACAGCAACAAGCTAAAGGCCAGCTGCAGCAAAGGGA
TGTGTTCCTGGCCAGTGTTTGCTGGAAATACTTGTGATGATTGTGACTAGGT
CTAGCACTGAGACAAGGGGCTAGGACTGAGGGAAGGAGATCATTTTAAGC
TTGTTTATGGGGGGGCCTGTTGATGGTAGTTTTGTTTGCAAATGGCTGTTCG
TACAGGGGATTGCAGGAACCCACTCCTCTGTGGAGCACAGTTAAGTTTAAG
TCTTCTTAAACTGGTTAAACATTTTCATAGTGGTAAGGCAGTGGTTAGCACT
TGACGTGGAGGCTCCAGATCAGGAAATCCATGGCTATTGGTTCGGGTCTTG
GATCCTTCATCGAATTTggatgaagaaaaggagaatacTTCACTATTTTCATTGCTGGTC
AGAGGTACAGCTATGTTACGGAATGGAGTCAGGAAACAGGTTCAAATAATT
CATAATGGATGGTGCCTCAGTTGGAAATTTATAGCTGAGATGTCATGGCAGA
ACTAAAAGCTGCCATGGCTGTGACTTTGAGTCAGATCTGTGGGCTGGATTT
ATTGCCGGCCCtttgcagctcctcctgccaaGACTCCAACTTATCCTGATAACTGGTTTT
CCTGTCTCAATTCAAGATTGAATTAGTGAAGGACAAGTGAAATGATAGCTA
ATGACAGAGGTCGGTAGCAGGACACGGTCCTGCTCTTTGTGCTGCCTTAAG
GTTTGAGCTGAAGGCTGGGTCTGGGTGTATGGCTGTCCAGTGATTCACTCA
GAGGCAGTGGttacaggagggaaaatacaGAGCCAGAATGAATTAAACACCTTTTTT
GTTCAGTAGTGCCCATTATTTTCCGTTTTGGGAGATTCACTTTTCAATCTGC
GTCCAACTCATTCGGAAGCCTTTTGTGTATGTGATACATGGATGATACATGG
ATCTCTGTGAACAGCTCTGAccctttacatttcttttcaatggtaaaatgttatttatagtAGGGAGTT
TGAGGCTATGGAGTTATTGGCTGTATGTCTTATAAAGCAAGCCAAATGCAGA
ACCAAAATAATTCTGATTCAAGTTTCTCTAAGCCCAAGCTCTGCTGCTATTT
GTGggatccattaaaaaaaaaaaaaaaaaatcctccttaAAGCCTTTCAGATCTGTTCAGATCTT
TCTTTATCTTGCAGAATTTGGATCTTCCATCCaattaaagcactgaaaaacaagaattCGGT
GTAGCTGGAAATTTGGGGGGACAAGTTATAAAGTTATGCTCTCCTTCTGTG
AGGAAGGTTTATTGTAcctttttctggttttcttcctaGATGTGAGGTGAGGGCACATGGAT
ACCTGGATCTGCACAATGGAGAAGTGGAGGCATTGGGGAAACTTGTGTCT
TTTAACCCAGGAAACTGCAGGTGTAAGATGCCTCAAAGGACAGAAGAGCA
AAGGATGCTGCAACAGCAGACTAAAGTTTGGTATTTTACTATTTCAGATGCT
CAGGTGGACTGGGTTCTGTTTTTCGTTCAAGTCTCATCTTTGTCCCTACTTA
GATGACTTGATTTCTGCTGagtatttccctttcttttacAGCTGTAATGGAGCAACTGTAT
GACAAAAAGtcctgtatttcttcttctcgTCAGTTCTGATAATGCAGGTGGGCATTTGAA
TTAAATGCCACAGCAGCAGTTCATAGAAGAGCAAAGGTGATGCTGGGTTTC
ATTCCTCTTTGCCTGGGAAGCAAGCTGGCATTGGGCCTGGGCCAGCTGGGG
TTTACCAGGTGCAGCCTCTGAGCTAGGAGTTTTGCTGAAGAAATCTCTTCTg
tgagagcacagtagcactCATCATCTTCATCTGGCAGATGGTGAAGCACTAAAAGACC
TCTGAAAGGCTGTACTGGAGGCAGGGAGGTTCTTGCCACCTGGTTCACCTT

GCTGTATAAGTGAGGTATGAGTAACAGTAACAGCAGTGGCTTTGGCTTTGT
TGTGTATTTTAAGTATGCGTTATTATTGGTCACAAATTGAAAGAGGTGCTCT
GTAAATTAAACAGTAGGTACTTCTAGGCTCAGAATGATGTCATGcgtattgttttcttt
cacctaAGTTAAAAATGACCAAAATACACATATTTTGGTTTAGCTGATGACATG
GCATAAGGCCACATTGTGACAGTGAGGTGAGGGGACTGAAAGCACTGAGT
GTGGAACAAAGAAAGACATTATGAACAAAGGACCTTGCAAATGAGTAAGC
AAGCTTGGTAGCTACTTGCGCCAGATGTAAACACGCACCAGGCACCTGGTT
CATACATGTGACCAAGGGGATATTGCGTGGGTATAAAAGGTTCTGAGCACC
TGCAATAAAGGTCTTCATTCTGCACCAGCAACGGAGTCTGTGACTCTCCTG
ACAAAACTGAGTGCTTCCCAAAGatgttgctttgtgttttcatcAACTGTTGCTGTGTTGT
AGGAACCAGAGGGTCAAGAGCCAAATAGGCAGCAACTGAGGAGCCAAAG
CCTCACTGAGGTTCTTggctcttttgttttcagatgttttcagaCTTCTCCCCCTGCTAGCTAA
CAGATTAACATTGctattataaaaatattcacagaGCATGAGTAAATTCTGCTTGTTATTCtt
aaaaagtattatttctCTGACAGAAGGAAGTGGCCACCAAGCAGCAGCGGCCTCCGC
ACACTGGGAGGACACTAACTGCTGTGCAGCTGGCTTCTGGGTCGCCTGCT
GCGTCTAACACCGTAGTAGTTCTGGTGATGGAAGGCTCCAGCCACTACCCT
TCTGGCTTTGGCAGACATGCTGCCATCATGCTGCTCTCTGAACAAGGCAAC
TCCAACCCAGAATCCTCTACAGCTGAGCTCCTGCTCACCAAACTCTTGACA
GATGTACGTGTGCTCTTGCAGTTTGTGCCAGTTTCAACACAACCCTGGCAT
GTTGtacaaaagcagtttttctttccagcaccTCAGGTTTCAGGATTATGGCTGACAGTGTGG
GTCACACTGCAAAAACAAGCGCTCACATGCTTGGATGAGGCATTTGCAGG
GTAGGCCCTTACCCAATCCACTGAGGACTCTCAGCTCCATGTTGCTGTGTG
GTACAATGGTGGGGGGTGGGGACACACGACAGAAGTCCAGCCTTCCGAAA
AGCCACCAGGGATCCTGGCAGTCTCCTGTTGCAAAGCAGTCAGACTGGCT
GGCACCCCAACAGGGGCTTCTTCATCCCCAGACAGCTGCAGCGAGGATATG
ACGACGTACTGCACCAGTCAGGAGCGAGCTGGATGAAATCTGTCAGAGCA
CGCCTAGAGTGCGCGGATGCCAACACCAAAGAGCCAGCTGCTCGGGGACA
GTAAACACCATTTCGGCTGCTGGAGggcaagcagcacagccaggcagccacaggagccagcag
ctctgtgcctcagcCTCTTCCACACGTGGTTAGGGAAGAGGGCTGCCAAGGGGAC
AGTGGCAGTAGGACAAAGACCAGAGGAAGACAGAACAGGGAGGTATGAA
GGAAAGAAGTTAATGcatcacacagccctgcaccGGCTACACACAACAGGACATACA
CAGCATATCTGTACCacctcagggctgtgccacaTGTGAGATACACAACATCCATAGC
ACAAATAACACATGCTTTACAAACTCAGTACAAATACACTGCATAGCACATT
CACGCTCCCTGGCCTCTCTCCCCTTCATTAACAAGTACCAAACCCACTGCA
AACTGCTGCCTCTCACTCTCAACTCCCAGCAACCGTTGTGCCTCACTGCTAT
AGAAGGGTGCACtcaacaccaccaccaaaccCCAAACTAGTTACAAAATGAAATCAC
AGGGTGAGTGCTGCCATGCGTAAAGTACAACCAACCACTGCAGATAAGGA
TGCAATACGCTGATTCCATTAGGCTACATTTCTTCCAAGGAGATGCTGCCTA
CAGATTATGCCAGATACTGCAACCAAGAATTTTACTTCTACATACACTGCAC
ACCGCTTGAACAAGCGGCACAGCTTTTTGTATTCAACCATTTAGAACcataatat
atttttagagtgaccaaagcaaaaccaaatttCATCCACAGAAGCAGTCAACCGCCTTTTTAAC
AATAAAGAAATCACATAAAAACCTGCAATTTTGCTCTGACATTGTATCCAGC
ACTTATTTGACACCTTAATAATTATCACAGCGTCTTCCAGCTCATCACACCAT
GGTGCCATGAAATCCCAACACCTGCTAAGCTGCCACCACCATCACTTGAGG

TTGTAAGCCCTTTCCGCTAGCACTCCATGGTGCGTGCACCTCAGGAGCACG
TGCTTGAGCCCTGCTCTCCAGGTCCCCAGCTCATGCACAAAGCCCCTAGAG
AGGGCTGAAGGTAAAAccatgatcatagaatcatagaattgctcaggttggaaaagaccttaaagatcatatc
TCGGTTCACTGCTATTTGTTTTATTGTCTAAAGAGCTCCTCCTTTGCACGGCT
ACATTTCATCTGACAGGGTCTTTCTGAGAACAGTAAGTAGTCCCCTCACCC
CCTAGTATCACATTTTCAAGGCTCAGTTAGTGAAAATATAACTGCCAAAAGC
CAGAGAAAGGCCAAAGAAAGTAACAACTTCCTCCATCTTTGTTTAATGACA
GAAGTCAAGCAGCAAATCCACTTAAGTCAGTAGTTTTTACCCTGCAACATT
TTTGTGACGTTCCCATTACAACTTAGACAATATTTCTGGAGCAAGATGCAAA
ATACCATTTGGTGGAGGCCACACCCTTGGAAAGGTTTTCTGTTCATCACCA
CACCTACCAAGGGCAAAAACAAGTTGTACACGTGTCTTGGGCATGGCCAC
GTTGGGCTGGAACAGAAGTCAGCCTGCAAGCATCCTTCCAGTAGAAGAAC
AGACATCATTGAAGCCTAAGGCATACACAGCAGGTTTATTATTTAGAATAGA
GATTAGACTAGAATAGTTtcattggaagagaccttcagaAGGTCTTtcagtccaactgcctgaccact
tcaagGCTGACAAAAATTAAAGCACAGTGAGGGCATTTGACCACCCTCAtgataa
agaaatgtttctttacGTGTGGTCTGAGCCTCCTCagtgcagctttgtgctgctcctgtGCATTCAGC
CCTCAGTtcccaggagcacagcctggCACCTCCTCTGCTCCCGCTCATCACAAAGTTG
TAGGGAGCAGTAAGGTCGcctctcagtctcctccaaCACAGGCAGCCCACGTGTGCT
CAGCCTCTCCCCCCAGGACATGCACTGGGATGCTTTCAAGGACCTTCATAC
ACTTTCTATATTGTTGAgaccagaactgcacacagtagTCAAGGTAAGGCCACACAAAC
ACTAAATACAGCAGGAGAATCAATCACCTTTTGACCAAACAGCTACGCTGT
GTTCAATGCACtccaaaaagcagctttctctcttggctgccagggcacattTCCAGCTCATGCTGAG
ACTGCTGCCACCAGCAAACACTCCCAGGTcccttcctgctgagctgctcttcagCCACTTG
TCTCCCATCTGTCCTGCGTCTGGCATTATCCCACCCCAGGTACAGCACCCAG
CACTCACCTTTACTGAGCTTTATGCCACCGCTGATTGTCCACTGCTCTAatctag
atccctctgcaaggcctctcatGCCTCCAGAGAGGAAATAGCATGTTTATTATAGCATAGT
TGTATTCTCATTTCCATCTGGCAAAGTAACCGTTGGTCCTCAGCCTCCAACT
CAGAGGACGAAATGAAAGTGACCTGTTGGCCCCATTATCATGTCAGGCAGT
GACATGCCTCCCCATTacatcacacacacagagtCAGCTTTCAGAGCTTTACATGCAC
CTTTCTTTCCCAGCATCAACAGAAGCTCTGCTAGTCAATCTAACAAAGCAG
TGACCACTGAGCAAGCTAAGTGCAAGGCCACATTTAGTCCTGCACTTTGCA
CCGAAGCACTGCAGCTAGTAAAGGTTCTTGAACCTGGGAACAGCAAGGCA
TCTGCCTTCACTAAAAGAAAGGCCAGAAGGTTTGCTTTaccctcctccttcccagtgcA
TGGGTACATAATATGTCCTGGAGACTATGAGCATCTGAAACAAGAGATAGTC
ATTTGTCTTTATGGGCTGCATCAAATGGCAGATTTAAAACAGTTGGAAGTGT
GCGGAGCAAGGATAGGAATGAGGAAACTTCGCTTGCTCCCTTGGTAACAA
AAtctacagcacagcaggagaatGGAGCATGATAATTAGGGAATTACAGCTGTTAGTCA
CAGAAAATATGCTGCAGTTGCAGGTCAGGGCTCTGAGGTCGTGGCACAGG
AAGCAAAACTGTAATTTAGAGTTTGTGAGGAACTCCCTGCAAGCATTAGGC
TCCCTGCTACTTATAGCAGCTCTACACCTGTTTTCCAGGACGGCAGTCAAA
CTGACAAGAACGTTAATTAGACACAAGCATCAGGGAAATGGAACTTCTCTA
GAAGGAGAGCTCCAGACAAGAACCACTTCCCTGAGAGTCattaaaataactaaataaa
taaaaaattaaaagctcaTACCCAGAAGTTGAGGTTTCACCACTTCTGCATATCGCCCT
CATTTTCTGCTGGCCCTGTCTAAGCGGACAATTTTAAACGTGAACTTCTAA

AGAGGGAGATGACAAAACTCAATCCCCAGAGTCTCACCCTGGGAAACAGC
AGCTCCCATAAGTCGCACCCATCCAGGCTTGCTAGAGATGAGCCCAGGGGC
AGCAAGCCACCAAGTGTTCATGTCCACTGCCCAGCTTTTGCTCTTCCACTC
ACTGCACTTACACCTAAGGACGTGGACTTACTTGACATTGTTCCTTGGGAA
GCCCACATCACACCCAGCTGCAGTCTCCTGGTGCCTCAGCTTCTTGCACTT
GATTCAAAGACGTGATCATTCTGGAGACACCGCAGTCTTGAAGAGGGAAG
TAGTGCAAAGATTTCTTTTCGATGCTGCTTAGCAGGCAGACCTGTGCTTTGA
TCTTGATTGCCTTTATAAAACAATCAGGGAACAGACTGGCACTCTCCTGTTA
GGTCTCTTGCAAACTAGAAGGACCCCAGAGCAGAGGCACATCCCCTTACA
GGTTGACAGACACCCACCACACAGACCAGGCACTCCACATGCTACCAGGG
AGACAGCAACAGCTCTCACCAGAAGCTCTGCTGGGCCTTCTGGGCTTCAG
AGATACTCAGGGACTCTGACACATCATCACAGCCTCAcagaaggctggaaaagattaC
ATAATTGAGGCCTCTCAAAGCACAAGGTCAGTATTCAAGAATTCCTAATTTC
TCATCCCTAAGGATGACAGAACAGAACCCTATTGGCAGTGCCTTCCATGCT
GCAGGCATACCAAGGCAAGAGCTGCAACTGGAATAGCAAAGTACTGCTAC
TGCCCAGAAACACTGCAGCTCCGGACACGATGGGAAGAGCAGAAGTGTG
AGCTGACCACCATGGTCTCTGTACCAGAGGACTGCAGTCAAACCAGGCAG
GACCTCCGTTATCCTCCTGGCCTCCCCcaagagaggagcagagctctgaagcCCATATCA
TGGGTAACAGCCCCAGTGAGAGCTCTGAGCATGTCCTGAGCAGCATCCCG
GTGTTACATGTGAGGAATTATAAACCCCACAAACGTTACTTTAAGGGAGCT
TCAAAGAACAGCCACTGTCTTTAAAGCTTAAAAGGGGAAGGCCACTTGAA
GGAGAGGAGCTGTTACCCAACTGCTGTTAAGAGAGTTATCTAACAGAGGA
GATGGGTGACCTGAAGAGAACATTGCAGTTCATAGCAAAATTCCACTGCAA
TTTTTCGTAGGTGTGTCCAATTATTTCCAGTACGACTTAGccaagcagctgaaagcag
gtTTGTAGTAAGTGAATTTAACAGTATTAGGACAAgtaaagatttgttttgtttcccattaAAT
ATCTGTAGATGTCTGCAGCCACTTTGGAGTTCTGACACCTTGCACAGCCCT
TCAGATTTCTCACATGGTTCCCTGCTAAGATCCAGGACACCCGTGATGGCA
CTTGACGGTTCGGTTTGGGAATACAGAGCAAGTTGTGTTGCACAAAACCA
CTACAACTCATTTCAAAGCATGCCTCTGGTTGCTGAGTGACGAGATTCAAC
CCAGCTAACATGTTGCTGATAGAGTTACGGAGCTGATGTTACCACCCACCC
TCCTCCAAAGCCCTGCTGCAGGTTCAGTGGCAACTCATGCCACCAagacagccc
cagctcctttgCAAAGCCAGAAGGCAAATCCGCACTTGGCTGCTTGCACCCGGAT
GGAACCAGCAGGCACTGGGCGGCTGTGTGTGATGGAAATGGGAAGAGAC
GTGGCTCCAACCCCTCGGGCTGTCCCAGAGCATCCTGTCGCCCAGCATAAG
GATCACAGAACAAAGAGCTGTCTTACCTGAGCTGGGCAGGAAAATGGGTT
GTCAAGCACCAATGTTAATTTAACAAGCACTTGCTGGAGCAGAAGTCAGTA
GTACAGAGGTAGGCCAGGTAGTTCCTGTATTAGCACCCAGTTTCAACAAGA
AAGAGCGTGCAAGACCAGAGAAATGTGCCAGATGTGCTGCACCATTATCA
AGCCGGAGTTCTGCTAGGGAATGTTTGTACTGCTTTGTTCAAGGCAGAGTT
TCAACCACTGAAAGAGGCACTAAGACCACATTATTTCATGGCAGAAGGACT
ATTCTTCAAAGTAGCATCTCTTGCCAGTTACAAAACTATCAAAATACTGATC
CTGTGCTGCAGAATACTCCCAATAATGGAGTTAAAGGTTACAGTATTTGATA
TGGTAGTCTATGACACTTTATCAGAAAAAGGTGATAGCTAGAGCCAAAATT
GGTAATGCTATCTTGACTAGACAAAAACACGCTAAAGGTCTAAGAAATCCT

ACTATTGCCAGTGGAGCatggctccacctctcctagaccccacttaagggctgactgccactggggaagga
tctctttctggagatccctcctctGTGGAGTCTTCCATCGTGAGCCTGAATCTTCAAATACG
GGTAagcattccttctctgtttgtttctttccatttctccacGTTATTCTTTCCAACTGTAACATCTGG
CCAGAGAGGTCATGGAGTCTCCTACTCCAGAGATGCTCAAAACCTGTCTGG
TgtcctgtgcaacctacttaAGAAACCTGTAGGGAAACTACATCAGCAGCAGGTTGGA
CTAAAggatctcctgaggtcccttccaaccccctactGTTCTGTGACTCTGAAAACTGGGGGGT
GTTGGGCAGGAGGCAGCCGCTGCCTGGGGACAGGTTGGGCATCAGTCAGC
AAGCaaattgctttgtgtttcatttgttttgtgtatatataattattattatcattactgttacctctctcttccttttctgtcctagTA
GTGGTTTTATCTAAACCTATGATTTGTActaccttcccttccccttcccccccttcaattctctccctcat
ccgCTGAAAGACGAGAATGGGAGGGAGTGAGCAAAGGGCTATGTGgtgttcagct
gctgctgggttaaacTACAACACTACCGCTTACTTATTTCCACATATCTGCCAGTGAG
GGTGAAACAAGGGGACGGGAGGGCTGCTCACTTGGTGGCTTATTCACTGC
CTGCTTCCATGCTGCAGTGACATCCTAAACAGTACGAAAGCTTTAGCAGGC
TTTTTGGAAGTTGCAAGAGACCTCACAACACCCCCGTGCAACTAAGAAAA
GGATACTGGCTCATGCCACCCACCACTGAAACAGACACCTCTGCACTGGA
ACGAAGCAGCTGTATGACACCATGCAGCAATACTTCACAGCATTTTAGGGT
AGACAGCAATTAACCTTTGGCTGAATCTCTTTTGCCCCAAGCCACAAAAaag
tgtttgttctgttttcttctctgcccCACCTCCACAGTCTCACAGCTCTGGTGTTCTCATTAGC
TGGCTGCCCTGATTGTGTTCCGGATCCTGGTACTGGGAATAACAGTCCTCA
AATGAATGGGCTGTTTATTCAGATACTAGTTTTGGTTCTCTGTGACTGACCC
AGAGTAGAGGCAGCCGATTCCGATGAAGTTAAAGGgaagctgcacagcactgtgcatgt
TTTCTAAGAGTCGAAACACTGAAGCCAAGATCCAAGTCTTCAAGTTGAAG
GAGTTAGGGCAACACCTTCTCCTCACAAGCCTGAAATGAGCCACTACTTCT
GTTCAAAGAATGCATCTGTTCATGACAATGAAAACTGAGCAATTAAAACTC
CCACAAAAGCCTCATCCCCAGGCCCTCACCCAGAGCACAGTTTCCCAGGA
TTCAGGTCTGTCAGGTACAACTTGAAAGCCCAGAAATTCCAGAGCCAGAT
GTCCCTTAGGTTTTGAAGTTCGAATGAGCATCTTGTCCAGCAAAGGCAAAG
GTCGAGTGCCTTTGAGAAAGTGAGGAAGCAGGGAAGCAATAAGTCCAAAT
AGGTCTGACCCACTAGGTGGGCTTCCTTCCTGTTCAGCCTAGTATCTCCAGC
TTCACCTGGCCCACAGCCAGGGGTAATCATGAGCTCATCTCATGTTAAGAC
TGCCACCTAAGACAGGCCTGGCAAGTCCTGCCATGAAAGACCAGCACCT
>TCONS_03458618
cagaaagagaagctCTTACTACATACAGCTGAGGCTCAGCAACTTGGCTTAGGCAG
TAGCTTCTGACCTGAGCAGCACGAAGAGAATAAACAACACTCAAAAACTG
GAAGGATCAACCTGTCAGTGTGCTATGGCTGGGTGCACCAGCAAATCACG
ACGTGCTCTTCAGTTGCTGCGACAAGATCTCTTCTGCTGACTGACACCTAG
ATCcttggagaaagagaaatgatcCAGTGAGAGCAGTGCTTCGTGAAGTGCACGGTGA
CTTAAGACCCACTCCTGCCAGAGGAGCTTGAGgcagaaagacaagaaagaggagagatg
aAAGAGGCAGAGATGCTCTTTTTATTCTCCCCCCTCTCTGTCACTGCAAGAT
GGAAGCTGCTGCCCAACAGGAAGATACAGAAACATCAGAACAACGAGCCC
CTGCAGCCTGATAGCTTGGAGCTCCGGTGTAAAGGAGGTTTGTTGGGCTGG
GGGTGGCTGGCACATGAACTGACTGTCATCATGGATGACTTGAACCCATTT
CTGAGTGTGTCAGTTACGTTTAGGAATAAGCAAGTAGCTAAATTATAAGTAA
CAGGGAAAggctgctgggaaagaaagaaacccc

>TCONS_03490736
CTTCAGCCACTCTTTATACCAACTGGCAGATGTCATCTTAGAGGGTTTTGA
CTGACATGACAAACCTCATAGCTGGCATCCAGTTACAAATGAAGAGCTGGG
GAACGGGCAGAATTTTCATGTGcttgaaggagaaaaatagccgtgttccagtttctgcctcCCAAA
GAACTGACACCAACTACAGCAATGAACTTCATGACACTGatccctgggaatgaagca
GCCTCAGAAAAATCACTGgacAGGAGGAGAAACTCAACTGAAGGTGTTTGA
CAGCGGTGTTCTGACTGATGACATTCCAGCTTCACCCAAGGACTGTGCCAC
ATGCCATCAAAGAATGGGTCACATACAACTGTGCCTGCTGTTAATGCTGCC
AAGCGCTCTGGACGTCTTCTGTGCTTCCAAATGCAACAATGCCCTGTAGGG
ACTGAAAGGCCTCACTTAGCGTATCTTCCCGGATAGAGAAAAGGGAACCTT
CAGAACGAAAATGAGATGTCTCTTAAGGAAAGCGTTCTTCCCTCTGCATTA
CTGAAAAGCAGGAATTGTTTtaagcagaacaaaagcaataccaaaaggaaatgtttgcaATATTC
AGTATTCAACGTTGACGCTTTCAAAAGATGTGTTTCCTTGCGGAGCCAAGC
AGTTGACTCCCTGCCACTAATACCATTATTTATAGTGTGGCACAGGCAAAGG
GACTTAATCCAGCAGACCCCAAATAGggCTTCTTCATTCATAAGAAATATAA
GGAAAGTGTGTCTGTACAGCGTCTGGCAAAGTAGTCCTCTGATCCCTTGCT
GGACAGGGAGGAGCTGCCAGCATTAAGATGTGAGGTGAGGGCACATGGAT
ACCTGGATCTGCACAATGGAGAAGTGGAGGCATTGGGGAAACTTGTGTCT
TTTAACCCAGGAAACTGCAGGTGTAAGATGCCTCAAAGGACAGAAGAGCA
AAGGATGCTGCAACAGCAGACTAAAGTTTGGTATTTTACTATTTCAGATGCT
CAGGTGGACTGGGTTCTGTTTTTCGTTCAAGTCTCATCTTTGTCCCTACTTA
GATGACTTGATTTCTGCTGagtatttccctttcttttacAGCTGTAATGGAGCAACTGTAT
GACAAAAAGtcctgtatttcttcttctcgTCAGTTCTGATAATGCAGGTGGGCATTTGAA
TTAAATGCCACAGCAGCAGTTCATAGAAGAGCAAAGGTGATGCTGGGTTTC
ATTCCTCTTTGCCTGGGAAGCAAGCTGGCATTGGGCCTGGGCCAGCTGGGG
TTTACCAGGTGCAGCCTCTGAGCTAGGAGTTTTGCTGAAGAAATCTCTTCTg
tgagagcacagtagcactCATCATCTTCATCTGGCAGATGGTGAAGCACTAAAAGACC
TCTGAAAGGCTGTACTGGAGGCAGGGAGGTTCTTGCCACCTGGTTCACCTT
GCTGTATAAGTGAGGTATGAGTAACAGTAACAGCAGTGGCTTTGGCTTTGT
TGTGTATTTTAAGTATGCGTTATTATTGGTCACAAATTGAAAGAGGTGCTCT
GTAAATTAAACAGTAGGTACTTCTAGGCTCAGAATGATGTCATGcgtattgttttcttt
cacctaAGTTAAAAATGACCAAAATACACATATTTTGGTTTAGCTGATGACATG
GCATAAGGCCACATTGTGACAGTGAGGTGAGGGGACTGAAAGCACTGAGT
GTGGAACAAAGAAAGACATTATGAACAAAGGACCTTGCAAATGAGTAAGC
AAGCTTGGTAGCTACTTGCGCCAGATGTAAACACGCACCAGGCACCTGGTT
CATACATGTGACCAAGGGGATATTGCGTGGGTATAAAAGGTTCTGAGCACC
TGCAATAAAGGTCTTCATTCTGCACCAGCAACGGAGTCTGTGACTCTCCTG
ACAAAACTGAGTGCTTCCCAAAGatgttgctttgtgttttcatcAACTGTTGCTGTGTTGT
AGGAACCAGAGGGTCAAGAGCCAAATAGGCAGCAACTGAGGAGCCAAAG
CCTCACTGAGGTTCTTggctcttttgttttcagatgttttcagaCTTCTCCCCCTGCTAGCTAA
CAGATTAACATTGctattataaaaatattcacagaGCATGAGTAAATTCTGCTTGTTATTCtt
aaaaagtattatttctCTGACAGAAGGAAGTGGCCACCAAGCAGCAGCGGCCTCCGC
ACACTGGGAGGACACTAACTGCTGTGCAGCTGGCTTCTGGGTCGCCTGCT
GCGTCTAACACCGTAGTAGTTCTGGTGATGGAAGGCTCCAGCCACTACCCT

TCTGGCTTTGGCAGACATGCTGCCATCATGCTGCTCTCTGAACAAGGCAAC
TCCAACCCAGAATCCTCTACAGCTGAGCTCCTGCTCACCAAACTCTTGACA
GATGTACGTGTGCTCTTGCAGTTTGTGCCAGTTTCAACACAACCCTGGCAT
GTTGtacaaaagcagtttttctttccagcaccTCAGGTTTCAGGATTATGGCTGACAGTGTGG
GTCACACTGCAAAAACAAGCGCTCACATGCTTGGATGAGGCATTTGCAGG
GTAGGCCCTTACCCAATCCACTGAGGACTCTCAGCTCCATGTTGCTGTGTG
GTACAATGGTGGGGGGTGGGGACACACGACAGAAGTCCAGCCTTCCGAAA
AGCCACCAGGGATCCTGGCAGTCTCCTGTTGCAAAGCAGTCAGACTGGCT
GGCACCCCAACAGGGGCTTCTTCATCCCCAGACAGCTGCAGCGAGGATATG
ACGACGTACTGCACCAGTCAGGAGCGAGCTGGATGAAATCTGTCAGAGCA
CGCCTAGAGTGCGCGGATGCCAACACCAAAGAGCCAGCTGCTCGGGGACA
GTAAACACCATTTCGGCTGCTGGAGggcaagcagcacagccaggcagcccacaggagccagcag
ctctgtgcctcagcCTCTTCCACACGTGGTTAGGGAAGAGGGCTGCCAAGGGGAC
AGTGGCAGTAGGACAAAGACCAGAGGAAGACAGAACAGGGAGGTATGAA
GGAAAGAAGTTAATGcatcacacagccctgcaccGGCTACACACAACAGGACATACA
CAGCATATCTGTACCacctcagggctgtgccacaTGTGAGATACACAACATCCATAGC
ACAAATAACACATGCTTTACAAACTCAGTACAAATACACTGCATAGCACATT
CACGCTCCCTGGCCTCTCTCCCCTTCATTAACAAGTACCAAACCCACTGCA
AACTGCTGCCTCTCACTCTCAACTCCCAGCAACCGTTGTGCCTCACTGCTAT
AGAAGGGTGCACtcaacaccaccaccaaaccCCAAACTAGTTACAAAATGAAATCAC
AGGGTGAGTGCTGCCATGCGTAAAGTACAACCAACCACTGCAGATAAGGA
TGCAATACGCTGATTCCATTAGGCTACATTTCTTCCAAGGAGATGCTGCCTA
CAGATTATGCCAGATACTGCAACCAAGAATTTTACTTCTACATACACTGCAC
ACCGCTTGAACAAGCGGCACAGCTTTTTGTATTCAACCATTTAGAACcataatat
atttttagagtgaccaaagcaaaaccaaatttCATCCACAGAAGCAGTCAACCGCCTTTTTAAC
AATAAAGAAATCACATAAAAACCTGCAATTTTGCTCTGACATTGTATCCAGC
ACTTATTTGACACCTTAATAATTATCACAGCGTCTTCCAGCTCATCACACCAT
GGTGCCATGAAATCCCAACACCTGCTAAGCTGCCACCACCATCACTTGAGG
TTGTAAGCCCTTTCCGCTAGCACTCCATGGTGCGTGCACCTCAGGAGCACG
TGCTTGAGCCCTGCTCTCCAGGTCCCCAGCTCATGCACAAAGCCCCTAGAG
AGGGCTGAAGGTAAAAccatgatcatagaatcatagaattgctcaggttggaaaagaccttaaagatcatatc
TCGGTTCACTGCTATTTGTTTTATTGTCTAAAGAGCTCCTCCTTTGCACGGCT
ACATTTCATCTGACAGGGTCTTTCTGAGAACAGTAAGTAGTCCCCTCACCC
CCTAGTATCACATTTTCAAGGCTCAGTTAGTGAAAATATAACTGCCAAAAGC
CAGAGAAAGGCCAAAGAAAGTAACAACTTCCTCCATCTTTGTTTAATGACA
GAAGTCAAGCAGCAAATCCACTTAAGTCAGTAGTTTTTACCCTGCAACATT
TTTGTGACGTTCCCATTACAACTTAGACAATATTTCTGGAGCAAGATGCAAA
ATACCATTTGGTGGAGGCCACACCCTTGGAAAGGTTTTCTGTTCATCACCA
CACCTACCAAGGGCAAAAACAAGTTGTACACGTGTCTTGGGCATGGCCAC
GTTGGGCTGGAACAGAAGTCAGCCTGCAAGCATCCTTCCAGTAGAAGAAC
AGACATCATTGAAGCCTAAGGCATACACAGCAGGTTTATTATTTAGAATAGA
GATTAGACTAGAATAGTTtcattggaagagaccttcagaAGGTCTTtcagtccaactgcctgaccact
tcaagGCTGACAAAAATTAAAGCACAGTGAGGGCATTTGACCACCCTCAtgataa
agaaatgtttctttacGTGTGGTCTGAGCCTCCTCagtgcagctttgtgctgctcctgtGCATTCAGC

CCTCAGTtcccaggagcacagcctggCACCTCCTCTGCTCCCGCTCATCACAAAGTTG
TAGGGAGCAGTAAGGTCGcctctcagtctcctccaaCACAGGCAGCCCACGTGTGCT
CAGCCTCTCCCCCCAGGACATGCACTGGGATGCTTTCAAGGACCTTCATAC
ACTTTCTATATTGTTGAgaccagaactgcacacagtagTCAAGGTAAGGCCACACAAAC
ACTAAATACAGCAGGAGAATCAATCACCTTTTGACCAAACAGCTACGCTGT
GTTCAATGCACtccaaaaagcagctttctctcttggctgccagggcacattTCCAGCTCATGCTGAG
ACTGCTGCCACCAGCAAACACTCCCAGGTcccttcctgctgagctgctcttcagCCACTTG
TCTCCCATCTGTCCTGCGTCTGGCATTATCCCACCCCAGGTACAGCACCCAG
CACTCACCTTTACTGAGCTTTATGCCACCGCTGATTGTCCACTGCTCTAatctag
atccctctgcaaggcctctcatGCCTCCAGAGAGGAAATAGCATGTTTATTATAGCATAGT
TGTATTCTCATTTCCATCTGGCAAAGTAACCGTTGGTCCTCAGCCTCCAACT
CAGAGGACGAAATGAAAGTGACCTGTTGGCCCCATTATCATGTCAGGCAGT
GACATGCCTCCCCATTacatcacacacacagagtCAGCTTTCAGAGCTTTACATGCAC
CTTTCTTTCCCAGCATCAACAGAAGCTCTGCTAGTCAATCTAACAAAGCAG
TGACCACTGAGCAAGCTAAGTGCAAGGCCACATTTAGTCCTGCACTTTGCA
CCGAAGCACTGCAGCTAGTAAAGGTTCTTGAACCTGGGAACAGCAAGGCA
TCTGCCTTCACTAAAAGAAAGGCCAGAAGGTTTGCTTTaccctcctccttcccagtgcA
TGGGTACATAATATGTCCTGGAGACTATGAGCATCTGAAACAAGAGATAGTC
ATTTGTCTTTATGGGCTGCATCAAATGGCAGATTTAAAACAGTTGGAAGTGT
GCGGAGCAAGGATAGGAATGAGGAAACTTCGCTTGCTCCCTTGGTAACAA
AAtctacagcacagcaggagaatGGAGCATGATAATTAGGGAATTACAGCTGTTAGTCA
CAGAAAATATGCTGCAGTTGCAGGTCAGGGCTCTGAGGTCGTGGCACAGG
AAGCAAAACTGTAATTTAGAGTTTGTGAGGAACTCCCTGCAAGCATTAGGC
TCCCTGCTACTTATAGCAGCTCTACACCTGTTTTCCAGGACGGCAGTCAAA
CTGACAAGAACGTTAATTAGACACAAGCATCAGGGAAATGGAACTTCTCTA
GAAGGAGAGCTCCAGACAAGAACCACTTCCCTGAGAGTCattaaaataactaaataaa
taaaaaattaaaagctcaTACCCAGAAGTTGAGGTTTCACCACTTCTGCATATCGCCCT
CATTTTCTGCTGGCCCTGTCTAAGCGGGACAATTTTAAACGTGAACTTCTAA
AGAGGGAGATGACAAAACTCAATCCCCAGAGTCTCACCCTGGGAAACAGC
AGCTCCCATAAGTCGCACCCATCCAGGCTTGCTAGAGATGAGCCCAGGGGC
AGCAAGCCACCAAGTGTTCATGTCCACTGCCCAGCTTTTGCTCTTCCACTC
ACTGCACTTACACCTAAGGACGTGGACTTACTTGACATTGTTCCTTGGGAA
GCCCACATCACACCCAGCTGCAGTCTCCTGGTGCCTCAGCTTCTTGCACTT
GATTCAAAGACGTGATCATTCTGGAGACACCGCAGTCTTGAAGAGGGAAG
TAGTGCAAAGATTTCTTTTCGATGCTGCTTAGCAGGCAGACCTGTGCTTTGA
TCTTGATTGCCTTTATAAAACAATCAGGGAACAGACTGGCACTCTCCTGTTA
GGTCTCTTGCAAACTAGAAGGACCCCAGAGCAGAGGCACATCCCCTTACA
GGTTGACAGACACCCACCACACAGACCAGGCACTCCACATGCTACCAGGG
AGACAGCAACAGCTCTCACCAGAAGCTCTGCTGGGCCTTCTGGGCTTCAG
AGATACTCAGGGACTCTGACACATCATCACAGCCTCAcagaaggctggaaaagattaC
ATAATTGAGGCCTCTCAAAGCACAAGGTCAGTATTCAAGAATTCCTAATTTC
TCATCCCTAAGGATGACAGAACAGAACCCTATTGGCAGTGCCTTCCATGCT
GCAGGCATACCAAGGCAAGAGCTGCAACTGGAATAGCAAAGTACTGCTAC
TGCCCAGAAACACTGCAGCTCCGGACACGATGGGAAGAGCAGAAGTGTG

AGCTGACCACCATGGTCTCTGTACCAGAGGACTGCAGTCAAACCAGGCAG
GACCTCCGTTATCCTCCTGGCCTCCCCcaagagaggagcagagctctgaagcCCATATCA
TGGGTAACAGCCCCAGTGAGAGCTCTGAGCATGTCCTGAGCAGCATCCCG
GTGTTACATGTGAGGAATTATAAACCCCACAAACGTTACTTTAAGGGAGCT
TCAAAGAACAGCCACTGTCTTTAAAGCTTAAAAGGGGAAGGCCACTTGAA
GGAGAGGAGCTGTTACCCAACTGCTGTTAAGAGAGTTATCTAACAGAGGA
GATGGGTGACCTGAAGAGAACATTGCAGTTCATAGCAAAATTCCACTGCAA
TTTTTCGTAGGTGTGTCCAATTATTTCCAGTACGACTTAGccaagcagctgaaagcag
gtTTGTAGTAAGTGAATTTAACAGTATTAGGACAAgtaaagatttgttttgtttcccattaAAT
ATCTGTAGATGTCTGCAGCCACTTTGGAGTTCTGACACCTTGCACAGCCCT
TCAGATTTCTCACATGGTTCCCTGCTAAGATCCAGGACACCCGTGATGGCA
CTTGACGGTTCGGTTTGGGAATACAGAGCAAGTTGTGTTGCACAAAACCA
CTACAACTCATTTCAAAGCATGCCTCTGGTTGCTGAGTGACGAGATTCAAC
>TCONS_03496367
CTCTCAGAATTATGCTTGCATTGCTGAGAAGATGGGTCACATTTCTGGGAAT
CCACCCTGACCTCCTCTGGTGATGCTTTGGCTCTCTGAGGATAGAGACAGG
GAACAAATAGATGCTTCTTACCCAGCTGCTGGTTCCACTTGAGGAACTTCA
CAGCTCTGGGTGATTAACAAGCTCCTGGATGCTTGCATGTGGGGCCTTCCC
TTCTGGACAGACCTCCAGGAGATGTGGTCAGGACAAGGCGATGGTGTGGG
CAGCACCTGCTGGGGGCActggagcaggaaggagaagcagagaaagaactCCGGATCTTCC
CTCATTCCTAACAGCAGGACACATCTCCAGCACAAGTCCTCGTTGTGCTTC
TTGACTTGGGGGCAAATGGCTTACAGgaagaaaaaaatacagacagacATCTTTAGCCC
TTCACAAGAAGATAACTGCTGGCTGGCCTGGTGGGAAGCGACGTGCCAAG
GAGAAGTAAAGCAAGATGAAAGCTCTGCCAGCATGTCACCGCGAGTGCTT
TTGTAGAAACAGCACGAGTACGATGAGCAAACCTTTGACTGCCTAttggaaaca
gatttttctcGACACAGAAGCAATCCCTTTGGAACGAGAGCCCTGACCAAAGCTG
GGCGTCAGAGAAGGGCTTACAGCGAGTTGTGGGGAGGAAGGAAACCCGC
CAGTGGGACAGACAGCATTGccttttgttttagtttgtaGCACTGATTGTTGAATGCTTT
TTGATTTCACAAGAATCTCTGGATTTTGTCAACTTCCTTCAGAGattttgctgggaa
aaaatGGTAACTGTGCTTAATGCTCTCCCTTCTCAACAAAACCACTCAACATTT
TCATTCAAATCAAAATTTTCCATGGACAGCACTGCGTGTTCCTCAACCAGC
AGCATAAACACGTTGGGTACTGCATTACTCTCTAGATATTTGTATTCCAGAG
GCAAGAACTCAGGTCAGAGTCATTTTCTGCTGGACCTCACGTAGCAAGAG
ACAACCTTACTCTGAGGACTTTGTAATCAGAGTGGAAAAGACAGaggacaaga
gaaaggaagaagaaaaaaaatactataaataTGCTTTAACAGATGGGGCACTGAGGTGGCAG
AAGATGAAGTGATTCGTTTGACGTCATAATAATAGGAAAGCTAAGCCAAAA
TGAAGAACTAAATCCATCCCTCTTGGAAGGCTCAGCACATATCTTTGACCA
CAGCCCATCCTTTCCCTCTAGTGCTGTTGAGTTCTTTGCAGTTCGCGAGGG
GATGGCACTTGGCACAGTGCTACTTGCTGAGGATGTGTCACCTAACAGGGA
CATTTCTTGGGTGATTCATCAAATATGCCTGATTGACCACTTATTCACATCAG
CTCCTCCTGGGGAAGCCCAGCACCTGGGTGAAGCTCTGCTTTATAATGGGT
GTTGGCACTGTGGATATTGGGGATTCTAGGCTTTGTCACCATCTTGGTGATA
ATGACAATCATGATAACAATTGTAGAAACAATAAAAACTGAGGTAGATCCA
GGTAGCTTCATCCAGCTGTCTGGCTTGCTACAGCCACAATGGTTGGGGTGA

GCAAGGGTGAGCAGAGCACCCATGGCAGTGTTCCCTCTGTGGGGATGGCA
GGAGAATGAGGGGTTTATCCATCAtctgggaaacagaaaacatgcttGGGCTTGGTTTCA
TCCCACCTGGCACGCTGGTGGATCCCTGGCAGGGATCCTCTGACCATAATG
GAAAGCATGCATAACACATGACCGAAATCTGGAGATTTACACCGATTTTGT
TTCCTGTGAGAGCACAAACCAGA
>TCONS_03596853
GCAGGTGTAATCAATCCAACCTTACTCTGTCAGCTAGACCACAGTAAATGA
CATATCTCAAGGCCCTTCTTATGCTACTTTTAATGAGTATTAATGGATGCAAC
CTCCCTAGTATTCAAGAGAGTATCATTTCACATCCACTAACAGCAGTGTAAtt
aacaacagaaagcaaagcaatggTGAGTGAGGCTCCGTATGATATTAGAAATAGCTTGA
AGTAAGCAGCATGTTATTTCAGGCCTTGTATCCACTGCCCCACATTTAGGTG
CAAGTACTCATCTAAAAATCCCCATAGCTCATATTTGGAAAGATGTGAGTTC
TTCATTGGTTCTACCTGTGATAAACAACTTCCTGAGGATTAAGCTCTTGCAC
TGCAGCTTTGCTTATTACTGTTTGCTCCAATGGTAATTTCTGAGGTCTCTTCA
TGGTTGGGTTAGCTTTTCAGAGGAATCTATTTGCTCTCTCTATATGAGTTTTG
TGCATGCATGTAAAGAATTCTCACTCTAATTGCAATATGTAATTTGCATGCTA
CTTTATCAGAGAAGCCTCCCATCCTTCTTTTAGAACTACAGCTTTGTGACTGt
atgtttttattcttcctggATTTAACAGACGTGAAGATAAACTTCAGATTTCAAAAGGga
GCCTTTGTGCATGAAGATTTTGCCCTTCACTGGCAATGCAACAAGAGGGTA
AACTTCTGCTGGAATGTATCTGCCAGAACTGAGCTTCTAAGAAAGAATGCA
TATGCCTTACTGTACTCACCTCTACTGTCTGGTCTCCATGAGTATTTATCAAG
CATCAATGAACGTCAATGGGTGCCTCTTTTTCCTAATGGACAAATTCAATGA
CACGCCgttgcttcatctgcacttccatgtagaacatcattttgtcaggctgTCCCACTGCTACCATCTgtt
acacagcaacaacatgtaatggaatataggcaggaaggttcaacctctactgccataccaccaacatctgcctctgatgttgt
gggccaaaataataaaataggaggcctcacttttggagcagtccttgtataaatcaagaaaatgtatgtataaatgTGTA
AACTTGATTGAGCTTCCTTACTTAGAACTTAACAATTTGAACAAGTTTCTAA
ATCTAAGAATACATAGAACTACTAGAGTAGCAACCATTCAAAATTTCCCTCT
CCTTGTTCAATGCACTGCTTCTGACTTTATTTATAGAGTACTGTCCAAGTCTa
gctttaaaaactgaattattaTTCCTCTATAGGTTTTTGGTGACAAGTATCATTAAAGTATT
CTAGAGTTTCATATTACTACATATCCATGAGATGAGAGAACATTAACAGCTC
TTACAGATGGGAATAGCAAGAAACTAAAGACATCTGCCTTCTTCCAGCGCT
AAATCTCAGGTTCAAAGTACCTGTTTTAGACTATTTAGTTATACCTATCACTC
AAACAGATGCAATTACACATACTTCAGCAAATGAAAGCACTATATCTCAAG
CTAGAACTCCAAAAAGAAGACAATGAAATAACTCCTTAAAAGCATAGCTTG
ATGGATAACCAATAAAAATGCTATGGTGAAGGACAACAATCCTGTGCCATA
ACcaagaaaacatgcttttatCTCTCTTGCGTTCCTTTAGAGAATGTACTCAGTACCTTA
AAGTGTCACATCTGGAATACAGGGTAAGAAGtctaaatattaaaatgcataaaTTTTGTT
GGATTGAAAGAATTTACACCACTAACACTGTGTCCAAGAATGTACCTTTAG
ATACCAAACATTTAAGACTGGTTGTTTTGTGAAAAATATTGAAGTGGTCGTT
TGCTATGAAACAGAATCAAAAATGGGCACTTCTATGAGAGCATAGAAGCAG
CACCTTGAAAAGAGGCAACTGATTTTAATAGGaaagttcaaaaagaaaaaaaaaaaaaacc
accaaactAGGTACTTAAAAAAAGGCCTCAGCAACTGAAGATCAATCCCTCAG
CCAAATGCCTTATCAATAGTTATCCAATCCTGGAAGGCTTCATGGAGCTTCT
CATTGCTTTACATGAGATTCCTCAAAATATGGTTTATGACTACATTTCCAATA

GCTTGCAGAAGGATTTGTAAAGCAGCAATAACACAGGGGCAACAGACTCA
TTTCTACTActattcttttcctgaaaatacaTGTGTTATGGGAATAGACTTGTAGTAGTAGA
ACTTTAAGGCCCAAATGCTGGTTGGGATCCAACTACAATATATAATGTCTCA
AGACACTGTGGAAAGAAAGTCCACTATAAATAAATTGAACTGgcaaggcaaagaa
aacaaaagatagaAAGGAAGCCAAAAGCACAGTGAACAAAGGAACCTGACTAAG
CTGTAAATCAGATGACTGTCAGTTCTGGGAACAAGCTCTAGGTTTCCTGAA
TCCTTATCTGCTACTGCTGATGACATCTTCCAAATACTGGATTTTTGGGCAA
AGTGAAGTGAGAGGTCCTTAAAAAAAGGCTCATGGGACAAAGGAGGGATA
ATGTTCAGTCTGAATTCAGTCAGCTTGCACATTATTCCTCAGgatgaaatgctattttca
aGTAAGAAAAGTTAATGAGAGATGCTCGCCTTGTTCATATACAGAAACAATC
TTTTCACAAGAATCACAACTGTACCTATTTTTTCAattgaaaacaagcaaatttaGTGTC
TGTTTGTAAAGAGCTTCAATAGTAATTAGTCATAATCTCAGCCCTGATcatataat
aattatttttatcattgcTAATATTAATCAGAatttacacacatatatatacacacacacagtgactTATTAAAT
TTCAGCTTTTAGTAAAGTTATTAGAATATTGCAAGATCTTGCAGCTATCACA
AATTAAGATTTTAATTGCAACCATTAAAACCATTACTACTGTATAGGCTTGTT
CTGGTAGTCTGTGTGCCATTGAtgatttaggaagaaaaatcaaagcttaCCTACAGTGCAAG
GAGTATGAAAATTAAACAGTTACATGCATACAGAAATTCAAGTGAACAAAC
AAATACTTAAGTGGAAGCTGAACACATGTGCTTCTTGTTTATATGTGCTTTT
CAAAGACTGTTTTTCCTaacagaaatgcagcactgtcccagtgctcagtcactgtGTTTCTGGGGA
TACCTCTTGGCTGCAACATGACCCAGAATTTAGGCTGCTACTGAAAGAGGA
AACAACTCTCTCCTGAGGCAGTTATGATTTAAATCTCCACACCCAACATTttg
cctcctttttttcttcttaccaGGTACAGCTGGTATCCTTCAACTTCTTGCTACATATTCTGA
GCATAAATACCAATACATAGAAACATAGTGTTGAAACTTCAAGACAAGTTC
ATGAAAACCAACTTTCAAAGGCAAAataacacagggaaaaaaaaaaaaactattagaAAGAA
ACTCAAACAAATCATGAGTGCAAACATCCAAAAAAATCCACAGTCATC
>TCONS_03602403
aTCGGGTCAGCTTAGCTGTGGATACACTCTTGATTATGTTATATGTTTATTGA
GAGACTCGTTATCCTTTACTGCTCTTCTGCAAACACTACAAAACCTCATTTC
AGTGAGAGCTGAGCAAGCGAGCAACACTTCTTAAAGTTCTTGTTCAATAG
GAGAGTGTGGGCTCCTTGTTGGTTTCAGAGCAGCTCACTGAGTGCACAAA
CCCAAAGCCCAAAGCATTGTGTTTCCTGCTGGTGTCAATGAGCCCGGTGTC
ACGGAGcaaacagagctgtgctgctgaggatcTGGTCCTGCATGGAAGTTGTTACGCGTT
GGTGTTGTCAGTGTTTAATGATAACCATTGCTTTGGCTCCGTGTTTCACATA
AAGCCATTTACAGAAGAATTTATGGAGTCCATATGTTTGTATTTCACTTAGCT
GCCTAAATAAGGTGCGTGTGGTGGGACGATATATACAATATTttgtcttgatttttattttg
aagctgCGTTTGCAGACTGGCTGGAGCCAAGCAGGGTAACTGAAGGATCGGA
CCGTATCACCCAGGAATAGGGTGCTGaatcttcccttccctctgcaggcAATTTCCACAG
AACTGCCTGGCAGGACTTCCCGAAGTTCCTCTTAGAAACTGAGATCAGTGG
GTACCCAGGGGATAATGAGGGAGGGGTGCCAAAGACAGCCTCAGTCATGC
AGCAGAAGTAGGATGGGAAAGGACATGATTAAGTCTTGTGCTCCGGGAGT
TTAcgtttttatatatttatttactgagTCTAGCTGCAGGCCTTTCACAGTGCTGGAAGAGC
AGATTAAAGCAGGAtgtgcaggctgcagcctgACACGGTGAACTTGAGCGCGCTGTA
GAAGGTTGGTGTTCGTTTCCCACAATGACAGGGGTTCTTAGGAGTTTatgaaat
ttttaaagaattaaagaGAATGAATTTCAGCCAACTTCTCATCAGGGATTTAACTGCA

GGTAGGATAGGTGAAACTCTGTAGTTTGTACCACAGATAACTCAGCTAAAG
GATGCAGAAGtgttctttttgctttccccGGATGTTTACATTTAACATCACTCAACTTA
GGGAACTCTTACTCTGGAATAGAATCAGATGCCAAGCAGTGAGGAATGACC
AGATTTCCTATAAAGCAACAAACACTTCTAAGTCtgttaaagaaaaggaatgaatgACA
AAAGGTGCGGTTACCTCCTCTTCTTGCCCTAGTGATTTCTAGTTGTGCTTAT
GCAGCAGAATTGATATTGAGAATATGAAAAGGAATAGCAAATGACTTGAAA
ACTCAAGGGGAGATGTAATCTTTGCTTTGGTTGTGCCAGCAGGGCATGCCA
AAATGCATCATAGACAGCATTTTACAGTAAACTTCTATCTTCAAAGTTTCTA
GAGTGATCCTATGGGTTTGGGCAGGTGACAGAATTCAGCTCATTCCGTTTC
CACATTAAGAAACTGCAGTATCCAAATCAGATTCAGTCCATGTCTGAAACG
AAATACAGTATTGTCTTGAATATTGTGCATCTTGTAGCAAAGTTAAGTCACC
ATACCCAGACaagcagcagagacagcagcCTACAGTCAGAGCCTTCTTCCATTCCATG
CGCTGTGCCAGTGTTAGTAAAAGAAGGAATATCTGACAGGCTTTACCAGAA
CTCCTTTCACTCATTAAATCCACACTTGTCTGAGATGAAGGCACTAGATCTA
GCAGCTTCTTTATACCATTTACAACTGAGCTTAGCTGTATACAGGGTTCCTG
AAGTCTCTGCAGTGACCTGAAGGTCAGACCAGTGCCcaagaaaaattacttaaatTGA
CATAGTATTTCTTACTTTACACACCTTCTGTAAGGGACAGCGGGAGGTCTGA
AACAGGCTAAGAAGGCATAAGAAAGACTGAGTGAAGACggggaaaaagaggagga
agaaaaccatTGCTTCTTGTATGCTGCAAAGagatgggaaggaggaagggattGTAAGGAAA
ACATCAAAAGACAAAATGGAGAGTACTGGTACTCAGATAAACCTAACTTAA
AAGTAGGCAAAGGGGGAGAGGGGACAGGATAGAGAAAACTGGTCTCAGT
GTGGTGGGGAAGTGAGAGCCCACCTCCGTGTGCCAGCTTTTTCTGTTCCC
AAGAGCGATGTGCTCTGCAGAAGACAGACAGATGGCATTTCCATTGCCTGT
GCTCCTGTGGGGGATTTTTCCAGATGGGGAGTTGGAAGCTTCTTTGTGCTG
TTACAGCCTTCCTCTCTAGCAGCTCTCGACCGCTCAGGGCCTCCACCAGTC
TGCGTGCTCAGATCCTGTGCTCCTTATACACGCGCACACACACGTCATGCT
GCTGCATGAATTTCAGCTGAACCTGTTTGAAAAATGGGTCCGTTTGTATCTA
GGGTGTTCTTCACACTTGAAAAGTATCCttgttgcctttttcttcttATGACATGCACCTG
TGTTTTGTCACAACGAAGAGATTGCTAACAAAAACTGAATTACTACCACTG
ATCTTAAGGAAAAGCTGACAGGACTGTTTTTCACAGAGGAACCACAGACA
CCATCGCCTCCATCACCCATTAACTTCAGCAGTGTCAGTGCTTTCCTACCTC
TGACAAACAGGGAGAACAGAAATGACAGGTTTCTCAAAattacctgaaaaaaataaca
accgTGGGGTGACCGAGGAAGCAGGAGAAATCCAAGGAAGCCATTCTAAAT
AAGAAGCTGTCACCCTTGATCTGCTCTCCCTTAACAGCTGAGCAAATGAAC
ACAGAAAGTAGAACTTTGGAAAGGTAATGTGATTTCATGTTTTACAATCAA
CTACAGGGAGAGACTAAGTGTGATCCCTTTATCTTAGCTATACATTACATTTC
TGAACATCTCACCTGCGTACATAAGTacattttttggtttgtttttatactTCCCTTCCTCTGC
AACTTTCCATTTGCAGCAACTGACCTTGCTCTTTCcactccattttctttcagtttcctttttgtaC
TCCTTACTGGATTGTCACCAGATATTACGGTCTTCCTGGTTAGTTTCCTTTCC
CCAGGAAcagcagaaggaggaaaaattgTAGCAGATTAAAATATATGGAAAGTCAAAT
ACGTGCAGCAAACATCTTCCTTAAAAATAGCATCACCctgatttcattatttaaattcatGA
CGTGGTTTCTGTTCTGATCTTTCTGCACATCCAGACATTCTTTAAAATCAGC
TTAGCAAATACATTAGAAAagtttgctgttgtttaaagaaaaaaaaagccattcaaACACATTCAG
GCCTGGAGATAGGAAAATGTGCAGGGCAAATGACGAAACACACAAGGTGT

GGGATTTGTGCTACTGGGAGCATCTCAAAGCAAGGAAGGTGGTAGGTCAC
ACAGCTATTAAGAAAGCAAGTTTACAGGTCTTGTAAGTCTTGAAATTCATacc
agagagagaaaactgcaTGATCCTCTCTCTTTGAGCACCTAAGCACAGCATTAAACA
GAACTGGCTGAGCCCATCCAGCACAGCGCTCGTGCTCCAAGCACAGTCAG
CAagagcagggtgctcagagcGCCGCTGTCCACTTGGTCACACCTCAAAGGATGGTG
ActctacagcctctctgagcaacccatcaccaccttcacagtgaCAAAGATTTTTATTACATCCAAGC
AGATTTTCTCCTATTTCAAAGACACAATAATGCAAAGAGATGGCGATGGTG
GAGAGTCCATAAAAAAGATCACAACATCGGTGGTAGGACTGCATCTCATCG
GGCTCTGTGATTAATTTTCACTCTTGTACTCAAAAtgatttttgtctcttctggGGCGTGa
atatttaacttaaaaaaCGTGTAGAATCGTAACGACCAACAGAATCCAGGAAACACA
ACAGGAGCAGTCTGGGCAGTCATCTTCCTGAAACAAACTTAGAGACCCAA
GGAAGATGCAAAATACAACAGGTGTGGCAATAGGATTGGGGACATGAATG
AAAAACTGCGAAAAAATCATACCAATGGAATGACTGGGCTTACATTTAATA
CAGCAAGGTGCTGATAAAGTCCATTTCTAGGGAAGCTGATGCTGAGCTGTA
GCACAGACCCTTCTGTGAGGGAAAAAGCAAGAACTGAGCTGAGAGCgcact
gaaaagctgcagaggTAAGCGGTGCCATCACATACAAAGCCTTTCATCTGGTCCCC
AAGGGAGGGCCCACCGTGTGCACAACACCGCGATGGGGACCAGCAGTAAA
TAACTacccaaataaagaaaaaggagagggaaaaggcaTGCAGGGACTACTGTAATGAAGTG
GCTTTgtttaaaatgatttcaaacacAGAGCAACCAGTCATCACCTGATTAGAATGCGAC
ATATGTTCCATTAGACAGAACTACGAAACGTCTGTACACTCAGCTGCATTTG
GGATAATAAGCTCTCATTTGCTATAGGCACTGTATGTTCTTTTCGTTTTACTT
CTATTGGCAATTAACCTCCCTGTTATTAAGAAAATGGAGCTCAAAGTGGAA
GACAAACACACTCATTTGCAACGCTGAAGAAACAAGATCCTTTCACATATG
CTCATTTcttcaaatgtgtttttacCTCCAAAGCTCTTTAAGATACAGCGAAGCAAATCA
AACCTTCCTCCAATCTTACTGATTCCCAAACTTCTCAGGCAGAAACCCCCA
CAGCTCTCACAGGCTCTTCGTGTTAGCAAGCTCTGCACCTCCAGCTGCTTG
GGGCTTACATTCCCACTGCAGATGGTGACCTCCATCCTAAGTAagtcctgctgctgg
aggtaAAATCTGGAAACTTCAAACAATCAAGGAAAGTTTTCACCTcccatctaaaaa
aaaaatagttggaaAACTGGGAGCAAATCCAAACAGTACTACTTTGGGCCTGcatgag
agcagcacagcagctggagTGCACATGGAGCTCCTGGAAAGATGATGATgtagaaagaaaac
tgaagaagcagcactttgctttgttgtgaGAGGCGAAAGGTCAGTTTAGCATTCAAAGCTctgc
acagaaagcacagcGGTCTTCTCCCAGTTTAGAGAAAGAACAGTCTGCCAAGCCT
GATGGGACTGTAACGAATGGTTACTTCTCAGAACATACAGTGGGATTTGCA
GGATCACCTGGCTTTGGCCAAAGTCTCTTAGCCCCAACtagcacaagcagcagcacat
gtTCTGAGCAGCATTCAGCTGACCATATCCCTGGACTGCAGttagcagcacagagaaat
gctgcttACTTACACGCTGTTGCAAATGTAAGTTTTACTCCTGATTCCTCCCAAC
CTGAGTTTACAGCCAGGTTCTTGGGCTGCAGCACAAACCAGTGAGCAATG
TTCTGTATTCAGCATGCACCATCTTCACCTTGAAATGGGACCCCATCATACT
GAGCTATGCCAGCACTTCGGGGATAAGGACCTGGAACCAGACAAAAATaagc
agcagggagaaagaagatggcacagctcagccaggaTACCTGACAGGACATCACAGCAAGCA
CAGCTTTAAGCATGAGATCAGGGAAGCAGTAATTCAGAAGATGTCTGGAA
GCATTCCTCCCATGTacaaaaatgaaggcaaaaaaagctgcacagaaaaaaGAGCTAGAAGAC
ACTCGATCAGTATTTCAAGCAGTAGTAGAGAAAAGGCATCAGGGAACACA
GGAACAACCTGCAACATGGACCAGTGGCCTGGCCAACAGCATACTATACAC

AGTATAAGAACACACACcgaaaaagaaattaagtgtCAGATCAATGGGATTGTAAGAT
CTTTGCTGCAATGCGTAACAGAAGAACATGATGGCATTTGTCAGTGCCAGA
GGGAAGAACGAGGAGGTCCTGACAGTTTTACAAGTTctgtttgtatgttttcaaCACA
AGCTAAGTGAATGCACGGGATTTAAGCAGTTTCCGCATATAATGTATGAAAG
CAGTGAGATAAACAGGGCAGCTCACAGACTTGTGCCACGGAAAGACAACA
GAATGGCATCAgcttcttcttgttttctctgctaAATCAAAATTTAAAGAACATACTCTCTAT
CTGCAGGTAGGCTGCAGGTGAGTATTCAGTCAAGACAACAGGTTTTGAAA
AAACCACAGGTGAAATGAGAACTCACATCCAGAAGACCCCGTGCTGAGGT
TGTGGCCCTCAGTTCACTGTTGCCAGCAGTGCTATGATTACCAGACTGACA
GACATTTTGCCCTCTTAATTCCATCTTAGATATggaagttaaaacaaaaatccaataGGAA
CAACTGGCAGAAAAATACTGTCCTCAGGCAGTGCCTCAACAGACCAACCT
GATCCTCATCTGTTCCTGCATTTGGAAAAACGGATTTTCCTTGGGGCAGATC
GTGGTAACCTTTGCTCACTTGCGTTGTCTATATTTGGCTGCACGAAGTATGT
TAGTTCTGATCTTGTCAGGGCTTGTAGACTTGCAGAAGAGTTCTCTGGACT
TCATCCCTGAGCTTGCTGCAAATTCAGATGAAGGTttagaagcaaaggaaaaagttaAG
AGATGGGCAATCCAGCATCGATTTAAGGTATACTCTACAGACAAATCCTCCT
TTCACTTTCCATCTTTGCATAAAACATGCAGTAAATTCTACTGAGAATTACA
GCCTATTCCTCGAACCAAAAGAACGCTTTGGACATTTGAAGGCTTACTACG
GTTGCAACTTCTGCACTTAGTTTGAAGCAAATGACAGATATTCTCGGATGTT
CTTGCCCTTTTCTCTGCATTCTCGTTTGAAAAAGGACATTCCAGCCTTGTGT
AACTGACTCCCAGCAGTGATTAATACCAGTCcctagaaaagaaacagtgttGTATGTCG
TGTTGAGCACTTCCACAGCAGGGCGTATTTCACTTCACAGAACTTTACCAG
AACTGCCCTTACATTACAATCAGCACAGAGACGTAACAACTAAGGAGGGA
CAGAAAAGCTAAATTCCCCTCACCGACTCTCGAAACAGGAGCAACTGTTA
CATCTGTACAGATTCAGAAGGCTCCTACTGCAATAAATAAGAACCTAACGT
GTTAAAGCAAGTTTTATTTAAGTAATTGGAGGGAAACTTCCATACGGTAAGT
AAAAATACTAGAGTTGAAagttgttaaaataaaaaaaaagatgatggtTCTCTGTGCAAACG
CAGCAGGCTCTGATGGAAAGGGGAAGCAAAGGGGCTCATAATATACTCCTT
TTCAAACCAAAATCAAATGCGAGCTAATGGTGGTACATGGCGTCGTTTTTTA
TACAgtcaagagaacaaaaaaacccaaaaaaagcaatttaagaGAACGCAACCTACCATGGAATA
AATTCATTCGCAGGAGAGACAGAATAAGACTGCTTTTATGAAAATTACAAA
GAAACTGATACAACAAAAATGGGGTGCGGGGAATTTTTCCACTAAggttaaaaa
atgaagaaacaaaaaaaaaaattcactttttgAACACCATTATTATTCAAACACATATGAAGTTC
CAACCTAGTTTTTTTCTTATACATGGGAAACACTGTTAAAGTGAATCTTCAA
GAGTGAGAAGGCACCGTCAACTGTCAGCTGATACCTTTAATGCACACtcaaga
aagaggaaatgatCTGGCATGATGCAAAAAATCATTAAGTGTTTATATATAGTCATAT
AtctttccataaaaaaaaaaaaaaagcggcAACAAGTCATTACTTCAGTGCCAAAATGGACG
TCTGTCTGGATAACTCGCTCGTTCAGATGGTTTCTCATGTAGACCTGTACGG
TATTTTTATACAGTGGTGAAAGTGGGTGATTCTGTGTTGTTGCAGTTCGTATT
AGTTGGTAAATTAAGCATTTTCTATGTTCTGAGAAGCCAATCCTATGGAGTA
TTCCTAAACTACACAAATGGAATAATTAAATGCATCCTGAACGGAATACTCA
TCTGTACTATGTAGCAATGGACCAGATTCCACTGGATTCGGTTTGATATGTAA
TGTCTTGTAGCAGATGttataacaaaaataacttttgctttttgattTACCTCACTGAGGCCATTA
ATGCTGTTCCTATAGTTGGTTTTCTGGAGCTGGATGACACTTCAACTGCTGT

CCAAAATGAGTTTGAACCTTAATCCTCCAGAGTTTGTTCACTTTGTTGCTTC
TATTATTGCACTAACTACTAAAGTCTTATATCTTCCTATATAAATCTAATTGTT
CAGAGTGCAGATTCTATATTTAGACAATGAActgtatgaaataaataaaacgaGGGGATT
TTCTGGTCAGAGTTGCTGATATGTTGAATACCTTAATTAATTCTAAAGTAAA
CAGTTTTGGTGCATATTCTTAAAAACCTATGGTCGTTCAGCATAAGAGCCTT
GACGTAATACTTTAAGaatggatttgttttccttctcaaaaaTGCATCAaagaattgttttaaatacagaatc
TGTGAGGCTTCCTACAAATTCATCTAAATCGTAATACAAACACAGTTTGTAA
TTCAGATAATATAGAAAGTTCTCTAGTGTCCATTGAAGTAAGGTCCCCAAAA
TGcagtctgttttgtttctgaatgggaaaacactgcaaaacattAAGCGGTTCCACTGGCTGAATAGG
AGAACTGAGGAAAATGAGGTCTTCTCTCATTGTCTACACAATCCATGCTGTc
atCTTTTTCTCGTATACATCTTGCCAAACAATGCCAGCAAAGAATTTGTGCTG
CATAATCTCCTTGGCATCATCAGGACCGCCTCCTAACCTGTTTGggataaaaataaag
aagtgttTGAATACAACCTCATTTTGAATACaaccttatttttcctcatacaATTTTTATTAAAT
GGCACTGAAGAAATTCGGAGTTTACATAACACATTCCAGAGCAGTTCTGGC
ATTTGcttcacatttgtttttcttcaaggTCTGCAGGACAGTCTATTTTAGATGTCAACGCA
GTTCCTCTAAAAGGTTTGAAAACACATTAAGAAATCTTAGTATGTAATTAAG
GCTtctgttaaacaaacaaacaaacaaccctcAGAAAGGATGCTGTCCTACGGCCCAGGGC
AGATACTGCAAGTAAGGGTCTGGGCAACCTGGCCTTCCCTGCTCCTAAATT
AACAAGTGACTGCCCCTCAGAGATTTCGAGAGCAGAGCAATGGCAGCAAT
AAACCTGAATTTGATTTTGATGCTTAAGAGAGGCAAGGATTTTTAAGACAA
ACTGATCTATGCAATCTGATTTAAAtttccattaaagaaaacagttgaCAACTGCAGTGAT
AATATCATCTTGTATGCaaatccttcctcctcttcagtTTCAAGAAAGCACAAAGGGTGT
TTTATTGACTTAAAGGTATATCAAGATAAAGCATTTTTATTCCAGAATAAAAC
TGTCCACACAGCTTATAGACGTAATCAGTTTAGTTGCAGTCTGGAGCTAAC
AGATGCTTAAATTTTGCTGTGGAGGTTAAAGTGTTCTCTGAAATACTGAACC
ACATCTGCACACGAAGACGACGCACTGTGAAAACTAAACACATACTGAGC
AATCAGCAGGAGACTTGTGCCAAGTAACAAGGTGAAGAGACTCTCTGGCT
TTTAAAGTGCAAGAAGTGCTAGCCCTGTTAACCTAGTCTGACCTCTAGTATC
TGCGAGccctctgttcctgctgccacCCCACTGCCACTAGCAAACTGAGAAGTTAACTT
GATTTTATGGGCTTGGAACATTGGCTCCAGGTGATCAAGGCTTACTAGTACA
AGACCCATGCTTGTTCCCTGGCCCTGTTTTATTTAGTGACACAGTATTCTAA
GGGGTTTGCTCGTCTCTGTTTCTTCAGGAAATCTGCAGTGCAACATACAAC
AGACTGCCAGGCTTCAAGTCACAAAGCGCTGAGGGACACTGTCACCCCTG
GTAAACTCTCTAAGTGCAAGCCTAAGTCCACCCCAGAGCCCCGCAGGCCA
CAGCAGGCTGGACGATGGCTCTCCCCTCCGTGTGCCAGCATGGCATACAGC
TGCAAAGCCACCTGGAACAGAGACAAACGCCAGGGTAGCCTGGGAACAG
ATAGAAAATCTGTGAGTAATCATCTCTCGGCTGGTTCCCTGCAGGTCTAGAG
AGTTTGCCAAGTCTATATCAGGCTCCTCCACTGCTTCCCTGGTGCAGATGAG
GATGGCCAGTGAAGAAGAGGGTGGAATCGATCAGTTCTTTCCTCTTTACTC
CCAGATGGCACCAGGCTGCACTCCAGCATCCAAGTTTGGGAGTTATGAAGT
GACTGTAAGCTACAAAGAACCCAAGTACTGTTTTGCACAAGTATGCAGAA
AGGCTGGCTGATTGCAGCCATACACAAGTGCTTTGGAGAATACATTATCTAT
TATGAGCCTTTGTTCCATGAAGGGCAAATACACACTCAGGAAAATGCCAAG
AGAAACCCCAGATGTTTACTTACCTTTTAAAAACTAATACTATTAAACAAA

TCCATTCAAATCTAATGGAAAAAACGATCAAAAACGCATGTTTTGGATATG
AACTTATTCCAAAATATGGTTGTGTTCTTCAGAATTATTATGAAAAGACCCT
CTCAAGGTGAACAAAATTGCGTCAGCACGCAATGCAAAAAGCATGGGGAG
AACTGAAAGCCCAACAAGTTCGTGTCTCGTAAGGATATGcaacaactgaagaaaaac
acagtatgAACAGCAGACTTTTTCAAGACTCCTGTACAGTGAAGGTCAATTCTA
TACATGGAAGTCTTCAAACAGAACCAAAATCAGTATAAAgagaacttaaaaataaaag
gcctCTGAGGTTTACTGTATACAAGGAAATACCTCAGAACGTGCAGAGActgaa
aaacattctttctttctttaaaccAAACTGGTCTCCTTAGAATGGATCTGCTGAACGTTTCT
GGTTATTAATTTAACTTCCTGCAAAATCTGAGACCCTCTGGTTTGGTGCTTC
AGTTAAATtccaagaaaattatttataaatgcaACATTAATGTTAAATTCAAATCGTTCCCA
CACTTTCAATTTTGATGATATTGTGTGTTACTTGCAAGTTACTTTAATCACTT
AATCACAAGTACTTGTGTTCCACAAGCCAAGGGCAAAGAGCTATCATACCG
TAGAGAAACTATTTACAAAACTTTTTAATATGTATATGGTCATTAGAGATACG
TATATTATAAATATAGTAATGCACATTATAAATATAATACATGCACAGACACTC
AGCAACGAAGGCACTGTTATCACAGATGAGATGATTAGAATTATTAATTaacag
gactttttttttctactcgTCCGTGTAAAGGTAAGCAGCACTCAGCTCTATTCTCAACTC
TTTAAAACATCCCA
>TCONS_03616015
CCCAAACTCACTGTCCAAAACAGAGAGGCAGACTCTTCATTTTGCAGAGT
GTTTCTTGACTATGATGGGAATTTCTCTTGACACAAAGGAATCAAGGTGGT
TTCAGGGATTCTGTGGCTCTTGCCACAGCTGATGGGCTCAAAGTCTCCCTTT
AAGCCCAGAGTACTGGTACTCACCCAAAGCTAGCAAAAAGGATGCTGGGC
AAGCTCATTTGATAATGCCTGAAAGTTCTTTGAAGATATGTGACACCTGTTT
GGAGTTATTAGCACAATCCAAGCACAGTTTATCATTCTTGAAAATATTACAC
AGATGTTGAAGACTACACCTACTACATGTATACATAAATGTTACATTTAGATA
TTATACATTTTGTGGCATTACGTTGTTCTGCAGAGTGCCATGCACATCTGAA
ATACGGACTGAATAAACCCAGATGTCATATACTGTACTTCTATTATAGTGACC
AGATTATGACTTCCTAAGAAATGCAAGTTTTCCTTGACAGCAGAAGTTGAC
CCAGAGTTTCagtttcagtgaagaaaaccaTAAATTATGTACCAACATGCATAATGGGC
AGAGGCAAGTGGGATCAATGCCTGAATAAACAACTAAGTAGAAcaagctttctgttt
taaacagatACCTTAGACTGAGAAAACTCGAACTTTTACAACAGACACTCAAAA
ATTCGTTTTCATTTCACCTTCGTAACACCTCTGATAATTAAATAGCAGACTAA
TTTCATCCTATTCAGAACTGGCATGCAGCATGcaaaagaacagcagaactAACAGTTC
ACAATTTAGATTTCTCGGTTCATGCACTGAATGAAGGATGAGACTAACgtaaaa
catatatatgtgttAAATGATTATCTAATAGGAAGAACTGCATTCTGAAGTACACAAA
ACAGGAGAGATTAAATTTGCATTgaaagactttattttcttttttttagacCTGTCATCTACTTA
TAGTAGACAAGTCACTACCAATAGCTGATTAACCTAACTGACAGGCAGCTT
TGTAAGGAGAATATATTATGGCACAACAGCAGTCTGACATGGTTCAGCGAG
TGCAGAAGAAATACAACTTGTGAAACCACCACCACACAGCAGCGCAGTGA
AAGCTGGGACAAATCTTCGTGTTGGTCTAACCAGAAGGGCATGCTGCAATC
TTCCCCCACTTCACAATTTGTCCTGAATTCATTAGTATCAGCTAAATCATGAG
TCATCTTGTAAACTTAGTGCTCTGCATAGAACTGTTCTTTTCCAGCTCACAG
AATCCACACTTATTTATAACATTGGGCTGATAGTTCACTACCATCATCTTAAG
CCATTACAACCATCTGTGGATTGCTGAAGAGCCATTCAGCAGACTTGTAAA

CTTGCACATTTCATTTGGGccttttacatttcattttcctccacttgaaaaataaatatttaagttctGTTCTT
GCAGTTAACCACAGAGAAGTGAAGTATCAGACAAAAAACTCAGCAGTGTT
TGCTCTGGAAGTTTTGCTCACTGTCCTCTCAAGAGCAGTAACTGCTGATCT
TGGCGACACAATTTCAGAAGGATTCATTTCAGAGATCTGCCAGTAACTTTA
GGTCTTGTTCTTTAGAAGTCATTGGAGCTTTGCTAATCCAGGGTGACTGAA
CAGAAAAGCCACAGAGCCATGGCCTTGCAGTACTGCCTAGTCACTGGATTT
TGCTCCCATCAAGTCCTGCCCCTTGCCATTTCTGCCTCC
>TCONS_03648098
GTGCTGTATATTTCTCAGTACTTGTTTGAGAACGATTCTGGAGCTCAGCAGA
TTTCGGGATAAGCCCTGGGAACGGGACAACCTCCGTTTGTCGGCAGCCATG
GTCGTGCTTTGCACGCACGAAGCTCTCTGGAGgtgTGCAAAAGTGGCTTCA
CTGAGCCTGATGCAGAACAGTTTTTAAGTTTTGTGAATTTTGGCGGGAGTT
CAGCAGacttcagctgttctgcagtgtaCGTTGAGCTGGAAAGAGGACAGATGAGAGTA
GCTCACACAGTCTTTTGATCAGCTGATGTCTTCCTTGTTGCCAGATGAGTTC
TGTACAGTTGGATAATTTGAATTTCTGGCCAGGCTGCAAAATGAACACAAA
TTGCTGTCACAGTAATTTGGAAACAGTTGTTACAGCCAACTCTGATTGATTC
TCCTGGACGGTAGGAATCTCCATTGTGAACACACGGGCACTCTTCAGCAGC
TATGCAGCCACCTTTGCCATCTGATACATAATTAGGAGGACATATGCAGCCA
GAGACACAGTGGGTTTTGTACTGCAAAGACCATGTAGAAGATGTGATTTAA
AACACTGCCCTGGTGGAAGAGTCCACCTACAACTGGTGCAACCGTCAACT
GGGACAAACTTGATGTTGCATGTGACATCAGGCTCACTCAAAGAACGACA
AGTAGGTTGACAGGAATCAACATTGTAAGTGTACTCCAAGGATTTTGGACA
TGTGTTGGTGTATTTCGCTTGAGAGAGTAAAACAAGGTGGTAGCAGGTTTC
CCAGCTGCTTCCAATTTGTAGCCATGGTACCATAGATAGTGGAACAACTAGT
AGGTATTTAATGCCACCTGACTTTTGCATCTTTGCTAGGCTCTCTGATTCAG
AGGAAtaaagggggagaaaaaagtcatttaatTTGTTACTAAGCTAGTAGGAGCCTAGGTT
Gaatttttggaa
>TCONS_03750071
GTTCtcgtttttttttcccttgtataTCCTTACCtgactacagaaaagaaaagaatattctgctttctgcagctccacaA
GCAAATCCCCAGTAGCGATACCACCACTGCAGCCTTAGGCTttttgtctgctgctgacc
tgtCTCTCTGTTATGTTGGTAGAAAGGTTTTGAGGGGCAACTTTATCAGATTT
AGGCAGCATTGCCCTCATACAAAAGATCAGCTAGGGCTGGACTGTTAAAGG
GATATTGATAGGTCCCTGAGTGCAGTGTCTCTTACAGAGCAGGGTTAGTTCT
ACCCAGACCTTCCCAACAGATGGCTGTAGGGGCATGcatgagcagctgctggaggcca
TCTCCTCTTAAGTAAGATGGAGTGTAATTGGAGCTacatcccagcagctcaggcatGAC
TGCTTGGAGAGGCTAAGAGACTGGAGATAACACCTCAAAGGCcagagagaaaag
ctgctgttcaCCTCTCTGACAGACTGTGTTGCATATTGAGGTATGACAGTAAACTT
TCAAAACAAGCGTTTGAAAGTACCTTTCTGCTGCTCATAATCTGTCCTTTAC
CCCTTTATGACCCATAGAGGCTGTATGATGAAGGACTTTGCCACTCTAACCA
ACAAATACGGAGtagaaaactgtaaaattaaGGGGAAAGATCAAAAAGTTGAAGattcttt
tttattgctgttttctggCTAGTAAGGTTTTTTGGTGTATTATTCCCCAGACAACTAGAAT
GTACAAACAATGAAAAAGCCACGGACagttttttccagaaatgttttctgtcttctatcTCCCATC
CCATGCTCAGCTTCCCCAGTGGCATACAACCATTGCCCCTGCTGAAAGACT
GTGGAGTTAACAGCTGAGCTGTACATCCCTCTGTCTGGGTGGGTGGACAGT

TGGATCTCAGATGGATGAAAACTCATTCCTATGGTGAAACAGGAAAGGGTC
AAGGTTGCTTCAGTCCCTTCCTGTTGGTTTTCCTCGTTCAAAACAGGGAAA
CTCAGATCCGTCTGGGGAATGACTTTTAGCAAATAGTAGTATTTGTAAGGTA
ACGTCTGATACGTATTGTAACAGCTGATGGTTCTGTATTTCCTCAAGGCGTT
TGACTGTTTTCAAGTCATTGTTATGAAGAATGTTTGCAGAAAAAAACCTTGG
CACTTGATCTGATTTCTCACATTTCCCCCTCTGAACTCCACGCCCCTGATGA
AGAACCAGACAGGAACGGGACCGGCTGCTCTGTATGGAGGAGGGCTACAG
AGCAGTCTGTGAGATGGTTCGCATCACCACTTGCTGTACTGAAGGCAGCAG
ACATAGATGTTAAGCTCAGTGAAAAGAAACTGCAATGCAGTGCATCAGCAT
CGGGAACAATAAACATAAATCAGGTAAGTTCAAATTAATATTAGGGCATCTT
CTTGGTTAGACTAGAAAATGAGTAGTGAAGAACTATGCGTTATATCTTCTAT
ACAGTGGAAATTCTGTACATCTACGCTACTGGTAATTTCACTCTGTCCTTAA
CTCACTTGATAACTCAGTCCTATAGCGTATTGCTATGGGTTGTTCCCTACAG
ACtaagaacaaaagaacaaattCTGGCTGTATGGATTGTACTTTGTACTTCTGCTGAGT
TGAACATGGATTAGAAAGCTATGATAGTAGGACAGAAgtctacttttcttttttttagtccgT
TTCAGAAGCTCATAGGATTTGCATGATATTATTCAACTAACCGTCTATCCTTC
CATTCGCtttctaaataaaagaaattctgGCTACAGTCATCCTCGAGTCAGAGCTTTAAGT
ATTTCTCATCTGCACATCATTCTGTAGTTTGTATAAACTTAATTGGGTCTAGT
TCGGTCCCAGTAAATAAGGCCTAAAATCCAGAACAGTcctttatgtttttcctttgattttgc
AAG
>TCONS_03752196
CAAAAGTTCAAACACCTTTTAAGAAATTTAGGCCAAAATGTAAGCAGGCCA
GAGCCATGCAGCAGTTCCAATGGCAACGGGGACAGCGCTTTGTTGAACCA
TTTTCAGGATCATGACTACTGGGTAAGATGCATGCTGAGTGGTGCTGCTTTT
ATAAACCACAGCAGGCGCAAaggtactgtgtgcagtttccTATAGTTGACATAAagatatata
cattttttttttcaacagtctCACTTCTACACAGTTTCAATTAGAAGTGCATATAACAACAA
TCCCCAAGAAGATTCAACATTGGGTCTTTCAACTGACTGTCAACACATCCT
GTTCTTCCTCATTTGCAATACTTGTCCCTAAAAACAAATTGGACACAAAAG
CTTAAGTCTATACTTATGAAATGCTTTTGGATTATGTAAAAACATTGCAGAG
GATTTTATTGCCTCTTGTTGTACTGGCTAAGAGTTACCCAAACCATCCACAG
ATGGAAGAAGAAGGGGTTATCTGCAACTTCTGGAGTAGCTAGAAGGAAGG
TAAGCATTCTTCTTAGTTCTCTAAGAAGTAGAAATTAAGAGCAAAAAGAGT
ATTACTCTATGGaaaccaacacacacacacgaggCCCAGTGTTCTCTCctgcatttaaatacaaaag
cacTTGCAAGTATCATAGCTATGCACTATTTTGAACTGAACTACAGAGTATTTT
ACACCAACTGCACGTTTCTGGTATCCCTTTAGTGCGACAAGCTCCTTAGCA
CAACGCTGAGCAATacctcagtgctgtttttttctgccacaaACCCATTAAGTACTGAGAAG
TTAATCATTAGCCGGCCAGTTGTGCATTGAGACAGACTCCCTTCTTTGTCTT
ACAATGTTACTAAATGCCTTCTTTGAAATGCCGCATAAAAGTCATAGATGCC
CTTTCTTTACAAGTCAGCCATCCAGGCAAGCGATGTTTTATATAAGTTCAGC
CAACAAAGAGCCCTCCTTGTTTAAATATAGAAGCATAGGGCAGATAAGGCT
GAGCAAACCGCTGCTGCCATGAGCCCGGGCTGCAGATAGGGGCTGGGGGC
TGGAGGACATTGCTGCACCAGATTTCACCCAAGAATTATGGGGTGGGGACA
GGATatgacattaaaaacaaattaggATGAGGTCCAATGGTCGTATATATTTGCAGTAGTG
CAATGGAAAGCTGTCAGCATTTTCTAAGGATGTTTCATTGTTTTGAAGGGG

AGATTGGCGTGGTTACTGCCTTGAAAACACACAGTCCTACACAGCAGGTG
AATGAACAGGGAGATGAAAAAGCAGGACTGAGTAAGACAGCAGAAAGTT
CCCCCTCCCAAATCACTGACCATcattcctccagcagcagtgcttccctACATTTTGCTAA
CTGGCTTTTACAAGGTAAAACTGGTGCTCAGGAGAATAATGCTCACTTTGT
TTGCCCCTGGTGGAAACACAAGGCCGGCAAGGCAGGGCTCAGCTAATGTA
TAATCTATATTTacatgtcaaaaaaaaaaaaaaacacccttatTTAGGCTCTTGATTTATAATCC
CTAGTTTTCATAAAAGTCACTCATTCCCAGTGAAAGAATGCAGAGTAGATTT
ATAGGATCTGCATTATGCCTTAAATCTTaatagcatttttaaagcacatttaTGAGTCATCCT
AAGAAGCGTTTTCCCTCTATTTTCAAGACATTATTGTGTAAAAACAGATCCA
AATCTTTACCTTAAAAACAAGATTATTTTAGCCAACCATGCTGGCATCGTCT
CTGCTGCAATGCTAAACACTATGTCTGCAATGTCCTTATAAGCCCCTATTTCC
AAGGGTTCACAGCTTGAATGTAAAATGCGCTCTGTACTAAAGTTTGTCTGAT
AAGATCTTTAACCTACATGAGTGATGAATTTGTGTTCACCATTATAGCCAAA
ATAAAG
>TCONS_03762785
TCCTATTGAGAAACCCAGAGCACTTCTGTACAGCTACATTGTTATCTGCAGG
ACCTGCACATCTCTGCACCTCCACTCTTGACTGGAAGAACTGAGAGTCTCC
TTGGAGTCTCCAAACTTTATTAGTGAGTCCATGTTGTTGTACTGGAGAGAG
GAGGGAGTCCCAAATCTGAATGCAGTCTTCTAGATGGGGTCCGGTAAGTAC
TAAGTAGAGGGTAATGATCACTTCTTTGATCTACTTGCTAAGATCCTGTTAAT
GCAGGCTGGGTTGTTGTTAGCCTCTCTGGCTGGTTTAGCACTTTCTTCCAG
GATGTTTGCCAGGGTAAATCCTGCCAAATCCAGTAgggtttgtgtgtgtttcaAGTACT
GTGCTGCTTGTTACTTAGCTTAGAAGTGCTGAACCTTGTTCTGATGTCTTGG
TAGTGATGGGGCTATAGTTTGGTGTTGTAGTATGTAGAGACGGTGTACCCTA
ATCTGTTCATAAATCACAATGATATCTGGGACCTTGTTCTGTAACAGTGATG
ACTATGGTAAATTCTGCTTCTGATATGTCAGAGACTCTTTAGGTTATCTTTGT
GTATTACTATGATAAAATAATTCAAGACATATTTTATGCCAGTCACGTGGTCA
TGTATTGAGGGAGCTGATTATCTGTATATCTGGTTGATGTGTGGATGTGCCCT
TCTCAAACTTTATGGACAAATAGACACTTTACCATTGCTTCTTAATGGCTTAT
GCTATTCAATGCATAATTACGTAGTCTCAACACATCTAGTTGAGGCATGTTTT
CAAGAGACGTGAGTCAGTACTCTGTATTTTCACTCATTGCGTGGTGAGTCA
TTACACCCACTGtcattaaaaatgcagagacagacCTCCATCTGGATAATGTTAAAAGCA
CTGTGATGACTTGCTTAAAAAGTGTATTAACTGAATGATTCGGTTGAAAGAA
TATCTGAATAGAATGGCAAAATGAATCCTAACTGTTTGGTTTCACAGCACAT
TGTCACTGGTACTCCTGTCTGAGACTGTGACCCTGAATTTTGTTAGGGTATC
TATTTGGTACTGTTTGATGAACACACTTGCAGACTTATATAAAAACTGTGGC
CATAACAGGTGTTTTTTTGGTTGAGACTAATCGGCACTGACTGTTTCTCTAA
CACAGTAAGGAAGAATCAAGATAGGTTATGCTGTAGGAAGTAATGGGCAAT
CTTCATCACTCTGTATAAGAGATGAGGGCAATTGATGAGACATCGCCTATTC
AGCACCATAAAAAACCTCAGACTTGGTGGGACAGAGCTGTCTGTGAGTTC
TGTTTGGTGGGTTTGCACTTTGAACTGGGTTGGATGCACAGTTCCTTATAAA
CTCATGTGTTTGCTTTCCTGCCCCATTCATAATTACTTTAAGAACAATGGATA
AGAACCACCTCCTCATACTGTTGGCAAAATGGAG
>TCONS_03763379

ATCTGCTTGGAGCAGGTGGTGGAGACAGAACTGggctttctgctgtttgctgctgctctacT
GTCCAGACTGCCTCAGAGCATCAGAATGTGATGCTTCCCCATGGTAATATTA
TCCAGTATTAAGAACCTAGTAATATTATCCAATATTAAGAACCTGGGAGACT
TCATTTAATGATGTCATCTCACATATTCTGGCAGAATATTATATGAGTAAAATA
ATTAAAGCGTATTTTGTGGCACACCGATATAAAATGCAATGACAGATCTATA
GCAATATGATGGCTAATTAAGGGTGAAACTTTCAAGAAACCAGCAATGGCT
AGAACCATCTTCCAGTGGTAACATGACTGTTTCCTTAGGGCCTTCCGTTGTC
CAGTGAACTTGATCTCTTTTAGGGTTGTTTATGTATTTAGGTCTTTGAAAAA
CTTCTGgcaactttcttttctgttgtggtTAAGCAGATGCATTAAGTGCCAGAGGgcaataaaatt
aaagaagtcAATAGCAAAGAAAGGCAACCATTAATCTTATTTGGCATCTACAAA
GCATCTAAAACTGAAATGGACTTTACACAGACATAAAATGCAGTTTGTGTG
ACAAAGCTCTTTGTAGATTGCACGTGGACCTAGTGTCTTTTCCTGCTTGGAA
TACCAAGAATCTGGATTTCACTTGATAACCTGGTGCAATGTACTAGATTGCA
ACTGTGTAACTCTGGAAAGCACTTACAAAATAGCCTATTCGGAGGATTTCA
GAACTCTGTTTAAAATTAAGCACCTTGTAAAACTGTTAGAAATTggaagttctgtttt
tctggctgctgggtgctgaaAACTTCTGAATATCTGGCAGTGGCAAAAAGTGAATAAG
GCAATTTTCTTGAATTGGTGCCTGAATCTACTAAGTTTGTGAGCACTCCCAG
GAAACACGTCCAAGGATTTATCTGAGAATCAGCACATCACAAGATCCTCTG
GGAGCCTTCCTTCAGTCCTTCATAGATTGTACGGCTCCAGCCAGCTGGATGT
AAGTCATTAAAGCTCCTTATTCTACCCATTAGTAGATTCATTAATATTTCTCTG
CAACTTGGGACACAACATTTCTCATGCCAAGCTTAGGAAAGTTTTCTCTTC
ATCTAATTTTGACTTAGAAACAGTTATAAGGACCAAAAGCTCACCACtgagagg
aggaaaaacttTTTGTAAAATTTAAGTCTGCAGAGTCTGTCATTCGTGGTAGAAG
AATTTCACTTCAGAGATTAAAGTCTTCCACAGAAATGTTGACTGTAGGGGT
ATTTAACTCTTGTCATTGGGCCCAGTATTCCTCTCTGAAGCTTATTAACCTAT
TAGAAGCAAGGCAACGAGCTTTTCACGGCAGGcctcctgaagaaaaaaatacaaagcacat
CTCCTAGGAGCTGAGAAACTGGATAAACATGGTGCTAAATTGTCCCTATGC
TCCAGAGATGGGTACAACTAGAAGGCTGCCAACTTAAGCACAAATAATCAT
TTTTGGATAGAAAAACACAATCAGTGTATTCTTTCATATACACTTAGATAtacttt
tattctttatatgtatatatgtatgcatatattatatatatgcatgtatatataaaaaatgctAAATGTCATATAAGTAT
AcataaacatgtattttaacAGCAACATCTGGGTTCTCAGGAGAGTTTGTGGACTTAAA
TTTGCAGAATTTTTCTGTAAacctgaggggaaaaataaaacaacttcaACTATTTGATTCCTc
caagaaaaaatacttcagtgctGAAACCAGCTGGAGTGCTAATTGTTCTCTGAAACGTT
GACTTGgttacttttctgaaagcaaacagtaaGCAGTGGCTCCTCATTACTTACCATGTGGT
AATTCAGCCTTGATGTCAGAGGTtaagcatttgctttttgctCTTCAACATATGTGAGAA
AACTCATTAagtagatcttttttttttaataaaggtcCGTCATTGCTAAGAGGAATTTATGGAA
GAAGCAAACTAGGGGCAGCAGGAACAagctcagagccctgcaggGTGTACACTGTA
TTGCCAAAGTCAATATTGCCTTTCTGTACACAAAACAAGGAGCCCTCAGGC
TCCAAAAACAAGTACAAAAACTCCCtttctgcaggtttttgttttagcTTTCCATCTGGGAT
ACAATGTTCTGATTCTTGCATGAACAGCAGCACTACTGGAATTTGACTTTCT
CTTCAACATTTTCTACCTACTTAGATATTTGTAGCATTTCTCCATTGATCAGA
GGTTAACTTCTTGTCCTCTGGTTCTCTTGTGTTTATACTCACTGAAAGATCT
GTACAATTATCAGTGATGTCTGTCTCGACAGTGCAGGACTGGAGCTGTGGT
GTAGTATAGCTGATAAGCCAAACCTGCTAATTGAGAAAGATAGGGCTTTATG

GCCAGAGGGGCTACCATAAGGATAGGATGGGCAATGGCCATGGGGACAGT
TGAGGAATTGACAGAGTCCATCGGATCCCATGTATTTTGAGCTTTGTTGTTT
GGAGGTCTTTTGGGGGCACCACGGGGTCTAGCTGCTGAAGGTGTGGGTTTA
TGGGACGATTGCTAGGCCTCTGGCACCTTGCCCAGGAAGAGCTTCTGTGGA
CAGTGTCTGGAGCCTTCAGCTGAGCAAGTGCTGCCACTGGCTCTTAAATTG
CAGCCACATTTCCTCCTGTGAGTGGCAGCATTAGTAATGATGAGCAGAGGC
TCTTCTTGTCATTCCGCTACTTGCCACATCTACCCTCAGGCTTTCATCGCTG
GTCTTCAACAAGTGGGCTTGATCTGAGCATGAGGTAAACAGTGGCTGTCAG
AGAGACTTGTGACGCACTGCCAAGTCAGGACAGGGACTTTTCTGGCCTTC
CATGCCAAATTCCTCAATATATTAGATGTGGCTCctcaaagaatgaaaattcaGAGGAC
CAAAATACACATCGTTCCGTGTCTCCTTGCTTCTCGTAATGCTTCTCCAACA
TCGTATacacctttcttcctctgtctcagCTCAGAATTCTTTGTTCTGTTGAGGCTTCTGCA
TAGCTGAGGACTTCAAAGCCTCTGCTTAATTTGTGTTTGTACTTCTAAGTAC
TGAATGAAGGTGTTTGGTGGAGGCAGGTTCTTTCGAAGTATTTGTGTATAA
ACATGAAAGTTACAAccttctgctccttgctgctttcatcttttcaggCTCAGAGATAAAAATGT
AGTTGAGTTTTTGGACTTTACAGaaagttttattaaaagaatGACTCAGGCTCAGAAGT
TCTGCTgagctcctttctttttctccgtctcttgcttttttttttttttttttttttttaatttcccttgtTACTTTCTCCTG
CAATCGAGCACTGGTATCAGGCTGTTTTGGTAGATGCCTTGTTGCACTGTTG
CTCTGTGGGGCACCGGGGCTGTGTACTTGGCAGCTTCTTTTGTTATAGACCC
CGAATCTGAccactgcacagctcctgtcACCACTGATGAGGGTGAAGGTGTGAAAAA
CTGAGTGTTTCAGCATCATGTTGCCAGTTCTTCTCCAGATTGCCATGGAGAG
ACACGCTAAGGATGCCTTTCCGTTGACAACAGAGGGAACACAGATGACTA
ACTTCAATATTGgcttttcagatctttttttcatatttgagaGGTAGTTGAGCTCAAGAAATCTT
TCAACACAAAGGTGAGAATTGGAGCAGGAATGGCAATGATCTTTTTGTACA
CAGAAACATGACTTTGAACACCCCACGTCATCCAGCAACGCAGAAATAAC
TTCCCTCTCTGGTTTTCAGAAGGTTCccagcttcctttctttctctcttttcgGGTGGTTTGGT
CTACTTATATGAGGTTGCATTCTTATATTGATGCCAACTGTAAAGTAGCACTG
GCATGTAAAACAGCACAGTATTTCAACTGTTGACACTTATATGCCTAGGTTA
TAGTGCCTGGTAACTGTAAAGATGTTAATGATAAAACCAGAGTAGGATCAG
GGACTCACGAGTCTATGCATTTTATTCACTGCATCACAGAAATAGAGCAGTG
TTATATATGGAGGTCAGTGACATACAATACTTATTTTGCTGAACCTTCTATTA
GTcccaattttctttttgtcttccaaCCAAAATCTCAGCAATGAGTGTCCTAGTTGCAAATAT
CATCTctttggagaaagaggaaattaTCAGACATCAGGATATTTGTGAGTGAATCACCCT
AGTTACTTGTAGGATGAGCCTCATACGCATATGGCTCCTCTTGGAAGCTTAC
TTGCTTGACTTGACTTTTGTGTCAGCTGGAATCTGATAAGTTAACTGTTAAT
GACTTGTACCAGTGCAATTGTTGGTCTTCCACCACTCATTCCTTATGAAATA
TTGTCAAAAGTTTACTGCTCCAAAAGCTATACAGAGCtatacttttatctttttctaaTGC
TGAGCACAGTAGCTATAATATAACCTTTCTGTGCTTGCAATAAACCTCACTG
AACTCCATTACAACAAATCTCAGTGCTTCACTTCAAGCACTTAATTTGTATGt
agagaaacaaataaacaaacaaaaaatcccaactAATATCCTGCAAAGTGATTAACTCTTTTGT
GTTATTGCCAATGTGACGTTCATCCACAGACATCCCATATTTCCTGGGATTA
AAATACTGGCCATGCTAATTAGAATGGGCCAAGGATGTAGGATATCAGGCA
GAATTTGCAGCACTTTccttttaatgaatattttaatgagGATTTTCCATGTTCAAGTTCTAT
CACCTGcactgacatgaaagtgcagTGCAAGCTTTCTTTTGAAGTCCACTAGGATTTC

CTTAAAGTTATGGTCAGATAATTTCATTGCTTGCAACTATCATCTCCCAGGTG
AGAGGAGTCAATTTCCTGCATACATTTGTGCAGACCATTTCCAGCAAATGTT
GTTTAAGATAtcccttttattctgcattacAAGATGCTGGAATCACGGCAGCTTTCAATT
TCTGCAGTTCAGCTAAGAGTAGaaataaatccGCCACTCGTCTGGCATTTGTTGC
TTCATGTGATACTACAAACTACTgagaaaaatcagcatttcatcTCCAAGTATTTCCTTA
ACCAATATACATGTCTGTTAAAAATATCTCCTAAGAGAAAAATACACCAGTG
TATCTACACTTTGCTTCTCTGAAGAAAGCTCTGCATATTTAGGGAGCATAGC
TTTGCTATCAGAGACACTGTCAAGTTAACTACAGATACATAATTCTGAAATG
TTCTGCTCGTTTAAAAATGTCTGAATGTTCATATATCATTAGTAAGAATACTG
CAGTTAATTAGTTACATAGTTAGTTCAAAAATGTTGTTGAAGCTTCAGTGCC
AGCTAAAAGAATGTTTTCAGGctcttttctcagctttgtGAGTTTCTGTGCAACTCTAA
GCCTCAGTAAGTTTTTGGGTATGCAGTGTTAATCTGAAGTAATGTGAGTTAT
GCCGACTCCCTCTTGAGCTACTGACCCAAGGCTGGAAGGCACCTCCGGAG
TTATTCATTCCAACCTCCTGCCCGAGGTGGGTTCAATGCCAAGTGcaggtcaggtt
gctcagggctttgggcTGAGTATGCAGAGTCCACAGACTTTCTGGTTCCTTGCTTCA
GTGCTTAATCCTAatgaagagctttttttttcctcatgtcctTGTGATTATTGTCCCTCACCCTTT
AATTGTGCTTCTACAGGAGAAGCATGGCTGCCTTTTCTgtaatctcctttcaggtagcgA
AAGAGAACAACCCAATTCCTCACCAGCTTTCTCAACTCCAGTCTGAGCATC
CCTAGTGCTTTCAGCCTCTCTGCGTTCAGGATGTGCTCCAATCCATGCTGAT
GGACTTTGGCTGGGCTCTCCCCCAGGATGTCAGCAAACCAAGTTATATCTT
GTTGGTCCCATCAGGTATGAGTGTGCACACTTGCTGTAACTCAGGTGGCGC
ACACACCGGTCTGGTAATCTGTGATAACCAAGCAATGCTAACCAGTTCCAG
AAACATGCATAGTTTTCTTCCAGATCTCCAAATCAGACTGTATCAATATACTC
TGCTACTTCGtatctctgcagagaaatggCTTTGAAGGGCTAAGTGTGGCAGACAGAG
TCTTCCTCTTTAGCatgaatgtaaaatatttaagagtTTAGACTAAGGAAGTAATTTCAGG
GCCTAGTTCACCATTACAGCTCTCCTCATGAAGAATCCATCTTCAAAGAGAT
ACTCCAAGGCCAGGGATGCCAAAAAGTATATTTCCATTGACTTCTGcacactttg
ctttctgttctaaAGACAATCCAATTTTCAACTAAGCTATCAATTGCATTTGGTGGAA
AACATGGGCCAATTTCCCATTTCATCTACCTCAAGGAGAGAGGGCTGCACA
GCTGGACTTCTGTTTTCATGAAGCAGAGCATAAAAACCACGCTTCTTTTTA
ACAACACCAAGAGCTCTGCATGGGGAAATGAGCTGCCTTCGGAAAGGCAC
AACTCATGTTTTAGCCTCAGAAtccttttctcttctaaagGTCGCAGTGCAATTTTAAAC
TCAGAATATTGCAATGCATTTAAATGTCTGAAATCTAGGGTAAAGAAGATTT
ACTaatatgaaatagaaatgaaggaaTCACATCTACCTTCCTTCCGCCAGAACTGACTCA
TCCCAGGAATAACAATTTCaagcactgggaaaataaaagtgGCCTTTTGGTGCTGTAAC
CTTAAACTTGAGACCTTGCTGCTCCTCACTGTACAGAGGGGTGATAGCCTC
TGTAATAAGAGGGCTGTGTTGGTGCATGAGGAGAGAGCTGTGGGTTTGTTT
ATTGTGACTTTAACAGGTGACTTTCAACACTGTCCACTGTCATTTCCTCATA
GGCAAACTGACAAAGCATGAGGCAGGTAAGTGGAAAGTGATATGGGAAAA
AACTGGCTGGACAGAAGGCCTGGAAGTACATTGTATGATATTGGGTATTCTA
TAGAGGTCATTAGGAATTGATAAATCCTTAATACTGCTTTGAGAGAAATTCA
GAGCAAGACTTTATGGTAACTGAGAACATTTTTTATAGCACAAGATTATGAT
AGGCatatattaatttttctctATGAAAATTCAAGCTTTTATACACACAATATTTTCCAAG
GAAATACTTTGTTGAAGTATTTGGATCTTACATATAAACATACCAGTTGTTCT

ATCTATAGgacattaaaaaacagcaaaataccCTCATGACTATATTTccttaataaaaaaattgatGAA
ATTGTAACTACGTAAGCATGGGTGTATTGACTTTTATTCCTAACACAGATTTA
ACTGCAAAGGCCTGGTAAAATAGAGATCAAAGCATCTACCAAGCTGCTCAC
TTAGCTCTGTAAAAGCACATACTTAGAAGACATAAGAAAACGATAGTAAAT
AGAGATTTACAATGTAACTTGTAATGGAAGTGTAATTGGGGGTTCACTGTAC
TAAATCCTGTTGAGGAGAAAAAGCCTTGGACTCTGCCTGGAGAGAGAACA
GGATGAAAGACAGAGCTTTATTGCACTTTTGCCTAAAGAGAAGATGAATAA
CCGAG
>TCONS_03763394
TCTCCAGGGACCCATTCCGGAGCTCAAATTAGCGATGAACCATAAACTGAC
CAAACATAGGATTTTGGACTGAATTTATGATGGATTGTCCCAAGTTTAATTTc
tagttctgttttgttgctaCAAACTTTCAACCCTAGATTTTCCCCCAGGTAATTAACATAA
TGTTATTGCTTTAAAGCGTGTCATTTGTTCAGTAACAGTCCCAATTACTCAG
GGGTTACTGACTTTGGGCTTTCAAAATGTGCTACAGGATGTACCGTGACCC
TAGTTACTACAGAAATGTTCATGGATCAGAGACCCTGCAGTCAACCTTCTT
GTTCTGACCAATCATTAACCAGGAGGAACATTGTGGAGCTGCCGCTGCTTT
TTCCTGAGTTACTTTAACAGACTGACCACAGGCATGTTCCTTCGTCCTTAGA
AGAGTTCCAGAAGATTAGTCAGCTAAACCTGACAAGGCTTCTGGGAAGCT
GGAAAGGACTTGGCTCAGCATATTAGGAACTAAGTGATGATGACAACCCCG
TGGATTTGTTCACTCCATCCTAAAGAGGACTTCCCGTCATAGTACTTTGTGC
ATGGATTTAGGAAAATTTGAATGGTaagtgctaaaaataaaataacaataaagcTGTCAAAAT
AAGAAGGATTTGGGGTTAATCGAGG
>TCONS_03764853
CCAGTGTGTAGTACAACCGGGCTTGATAGCACTGTTGTGGCCCAGAGCTGT
AGTTTTGTTTGGCTATTCTTTTTGCATGCTAGGGCACACACTCAGCTATTAA
GCACGGAATTAAAGGATCGCATTGCATATGTTTGACctggctttctttctttgtgaatTAGT
ATAAACTCTGGGTTGTTGGAGTAGCTGCAGGGATACCCAGCTTCCTGGCAT
TGGTCCTTGCAGTCATCTTGGCAGTGGTCTGTGTTCTTCAGTGGTCAAACT
TCAGATGGAAACAGACGAGTGAATGTGGCGAAGTGCAGGAAGAACATGAA
TATGAAAAATCTCCTCCCAGACCTCCAAATGATATATACCTGAATGTGATGG
AGGAGACAACAAATATCTATGTAAATGAAGATCAAGGAAAACACTTTGGAA
CAGGCATCCAAACACAAGCAAAGGCCCCACGGATGAAGAAGGCCAGAAG
AGAGCCCACTGTGATGGAATCTGTTTATGAAAATCACCCTCCCtcttactgaaagaa
aagtacCAAGGGATGGCTGGGACCAGAATTAACAGACACGGTCGACTTCCCA
GACCCCAGGGGTCCCATGGCTGCATTTCAAGAACATAAGAGTTTTATACTT
GTGTGTTTTTGAAGTTCCAGAGGACAGGACAGAGAGCCATTGTGAGGGGA
AAGAACCTTTTGAGGTTATGCTTCTGAGAGGCTCGGAGATGCTGTCCTCAT
TAGGAGAGTACGAAAACTCTCTGGGAGAGGTGGCTTTGCTGTCCTCTTATG
TTTAACTTACTCCATTTTAATAAAGAACATGTATGTCCTGGTAGTGATacagctg
gaagagaagaacaaccAGAGTGTTTGCAGGAGTCCCCATCACTCAGATGCTTTTGC
TCCTCACAGATGCCAGGGAGGACAATGAGGCACCCTTGTTCTCATTTGACA
GGAAAAGACTAGACTGTAAGTGAGGAAGAAAcctgaaggcagaaagaaacTCCGGC
TGCTGTCCTTACCCCTGGAGATGGCAACTTCCCTCTGTTCTCCCCTAGGGA
AAGGCTGTAACATAGCATGTCCAATGAATGAGAGGGGACCCCAACAGCAG

GCAGAGGTCTCACGAGCACTGGAGCTCTGGCTTCCTTCAGCCGCTGAACT
GGGGGCAGAGCTGTCCTCCAGGAGGgatgctgcagctttctgtcaGCACCCTGTGTGC
AGCATACTGGCAGGGATTgttgcaggcagcagcagtgagtcCAGAGGTGAGGCGGGCA
AGGCCTCACCCTGGGGAGGAGAGCCCTGCATGCAGGGTGCTGCCCTCATG
CATTCTGACCTACTCTGTTGTGTCGAACAGTGGCTGCGGGCACGGTCCTAA
CccaaagaaaggaacagctCAAGTCCTCCTAAATAGATTGCTCTTTATTGGGTCATTA
GCATACACATACGCAACACAGTCACTGTGCAAAGTGTGCTGCTCTGGGTGG
GACACATACAGCCCTAGAAGCAGGGGATGGACAGCCATGGAGTCACAGCT
GTGAGCAAATCAGCTGTTCAGTGGgactgctgtgctcagggagcACATGCACAGAATG
TCACTGCAGTGAGGTGCATCATTACCTTCTGTGCTGTGGTGAGGAGGAGCA
GTTCAGGGAGGTGTTCAAAGTATCCGGGTTTGATCTGCCTGGGACTTTGTC
TGCGGGCTGTCATGTGGAGGCTTTGGGTTGGTGGGGCAACGCTCCTGCCA
CTGGTTTGAGTTGGGGAGGTGTAAAGGAGCAGTCTGGctgaggagcacagcagtgag
gtTTTGTGCCCCTCAGCGGGGGATAGCACAGAGCTGGTAGTAGGGAGGAAC
ATTTCCTGTCCCACTCAGTGTAGGGGTGATGCTGAGTTCCTCAGGGCTGAT
GACAGGCTTCAAGGTGAAGTTCTGCAGGATGGCGGTCAGGAGTAAGAATA
TCTCCATGCGTGCCAGGCCCTCTGCAAGGCAAATGCGTTTCCC
>TCONS_03775965
cttgcatttgtttttaagagaTACCTAGTGATACCAATTCAAACATAAAGTCAGCATGCA
GATCCTGGTGTGATCAAGTAACTTCATCTTACCTTTGAGAAAATCCCTCCAA
GGATGTTGCCTTCCTTTCAGTTCTCTGAACCCTTGagaaaatttatttaattaaaaacttttCC
AATGGATGACAACAGATTCAAGTAGAGATCCATGCGTCTCTCTGCCATGTA
CTCCCAGAGGGAGCTGCAATCAAAACTCCAGGGATGGAAAGCCAGAGAC
AGAACCACATGGATGAAGCTCTGCATTGCTCGAGAATACCTCCTTCAGACA
CATCATCTTCTTCCCCTGCACTTGACTTTCACGTACTTGATTCTGGCAGTGG
CATTATGAAGAACTACAGGACTGAAGCAGTAATGACACTGCATTCCCTGCA
GAACAAAGAGCTGCTTCTGCCACGCTGTGCGTTGTGTTCCATGACAGGCTT
ATCCAGCAGAACCAGGGAATAACCTAACCGGACCGAGACTGCAAAGGTAT
CCGTGTATATATTTAAGATATATAGCAAAGTACTCAAGATGCGGTTGATGCAA
GCAAATACACCTGATAAGGATTTTGAATGCCtaactttttcatttctgtagatAAGGATTT
TCACAGGTCTGAAAAGTGTGTTGCACTTAAAACTGACAGTAAGTATCAGGA
CAATCAAGTTACCATAGTACCTGGAGGCATTGCTGGAAGGTAGGACAAGGA
CAACATCAGCCTTATTTTGAGAGGAGTTACTCCAAGTTCCCTAAACATAGCT
AAATCAGTAGCTGTTGGTCCAGTCTAACAAGGACAATGCAGATAGATAGAA
GGGACCATGTGAAAGTCTGTCTGTTGATTCTCTCTGTTTGAAATGGTGCAG
CAATCTGCAACTACTGATCTACTATCCCTGAACTATGGTAACTGCTACTTCA
ACCTACAGCAAAGAACATTTGCATGagtgttgcagaaaaaaaagcccttgtCACAGAAAC
AAGTGTATGACAGAGTGAATCCAGAATGTCTTAGTCTGTAAAATATGTAATG
TTTGAACAATGAAATGTTGCAAATACAAACATCATGAATAAACTACTCTTGC
TTACAAAACATACCTATTTCATATTGTAAGCAGGACTTTCTGCATCAGGCTCT
AGTATCTTTCTCTGTGCAAGTGTTTTGAGCAACAGCGATATAAAGAGCTGCT
GGCAGACCTCCAGGATGAGTTCAGAATGGAAGTTAAACAGCCTGAGCTGT
TATGGTCCAGCAAACAATCTCTCTTTTGTCTGGTAGCAGTTTCTTAATAATTT
tggaacacagaaa

>TCONS_03968192
TACTTGAGTAGTTAAAACCAGATGATATTCTGTTGTGAACAGAAGAAGGGA
CAGGTGGGattcaaggaaaggaaagggaaaagtgtGTGTAAAATTACCATAtcctctttcttaaaaata
catgcaATATCCACTGTATTAATGGAAGAAACTTCAACCTCCCTTTTAACATTTC
CTCTCAATAGCATGTGTGGGCCTGGAAAGGGAGGGGATTATGCCTGTagttttctt
aatttaaaatacttttctttgaGCTGCTATTGTAGCTTTTAAAGTACTTACGTGCCCCATGT
GTGTCtggtttttaatttgtcttttacATGCTGTGCAGGAACAGAATGTAACACTGCTCCA
AAGTCTTGGCATGCCAGTGTTGCCTTTGTGTATTTGAAGTATTTCCACTGGT
CTTCGGAGTCCTGTCATCCACCAATGTGGAATCCACAACTCAGAGCTGTGA
CTATCCTTTGGAAGGAACAGACATGACAACCATCGTCTTAACTGCACTGTG
TCAGAGGAACCATGAGTTTGGCAGCAATCCATACCCAGTGCATGGAGGACC
TTCAGATGAATTTTTCTGCTACAGTTTAACTTGCCACGTTTAATTTGCAAGT
ATCAAGGTAAGGAGCCAAGCAAGTTTGCATTTATATTCAAACTCATTTTCTA
CCTTAATAATTTGTGCAGTTGATATGTTTATATTTGCTGTAGAACATCGGGTT
TCATTTCTAAACCTTGCCTTGAGTGCTGGCCATAAGCCTACCACgaacttcaaacca
tttctcTACATAAAACAGCATGCTCTTTTAGGACATAGGGAAGGAAGAATATAA
CCTTACACCAGTCAGCAGGTGGCTTGCATTATTAGTCAACTCtagctccagcagagc
tggaaaacacaGCTGTCCTGCATATGCCATGAAAGCTTGCTTTACTCCGGGACCA
CAGTACTTGTGTTCAGCTCTCTGTTCTCTCTGtggcatttttctgtttgtcactGATTCTGA
AAACTGAACTTACAGAAAAGCTTTGTGGTCTCAAAAACCAATACTTAACTG
TGttcaaatactttttcataGAACATGCATTAGAGAACTGGGCTTTTCTGTACTCTGTTA
AATTATGTGTTTGTTTATGTGACATAGCACTAATCAGAAACTGTTTTTCCCCT
TTAGGAAGTTGTGACTTTCTaaagtgaggggaaaaaatggaaatagttATTAGAAATTGTAC
TTACAAATATCTTCACAGTTCAATTTGCTAATTTTTAGTTTGGGtattgagttaaaaaa
aaaaaaacaaaagtttaatttgaaaagcaatCATACATGTCATTTGTCTCAAATGCAGCACAA
ACTTAATGCGGATGTATCTGAATTGTTCATTTAGTCAGttttaaatcttatttaaaaaaaaaat
acactgctgtGAAATGATGGAAATCATTCTGATTATAATCAGGAGGCTGTCAGCCC
TGTATTTCCAGACAGGCTcatcacagcacacagcctgtCTTGTGGAAGGAGAACTGTA
GCAGCATGaagtggcagaagaaaaaaatattttctttgtcagtTGAGTGTTATGTATAGGAGCGG
AATATGTATAGATAGAACTTGCTCCAGCAATTTTGTGAGAtttgaaaaagaacaatttcC
TAAGGTCTTTCATGTCTAgacttgtttgcttgtttgttgaatgaattttttgttttattgatatTATGAAGGC
TGCTACAAATGTAATGGTACTGGCTTTAAAGTAAGTTGCATGATTGGAGGTT
CtggttggatattgggaaaaatatttcccagaggtgtgaggcattggaacaggctgctcatgGAGGTGGAAGAG
TTACCGACCCTGGAGCTGTCCAAGAAAAAGGTAGGTGTCCCACTGAGTGA
CGTGGTGCAGTGGTGATGGATTGACAGTTGGACTatatgatcttagtggtctttctaaccttga
tgattctgtaatgcttcctatttaatGATGTTGACCTATGATATCAGAGGCaaatgttggtagtatggcagt
agaaagGCATCTAGCATGGAAGTGCTGATGAAGCaaaaggtgtgtcattgaattcctccagtCG
GAGAAAATGGCACccttgacattcactgacacttggtgagtgtttatggagatcaaagagtgcatgtgagcaca
gggaggtggtggctggcatgtttctgcagtggcaacagtgggtcacttctgctAGTGCAAATTTgtatgagtgtgg
catgcaggttcttgttcagtagctatgcattttcactggcGGAGGTGCTatgctgaaaaactgttttttaactGAGA
CTTTGCCCCATCAAAttgtgttactgtgctctttgtagctggtgtattttctgtggaaataaataggaagcattactt
ccTATCTCTAGGAAGAGAGATAGATCTCTCTAGGGATCTAAGTAGATCTCTAG
TAGAGATCTAAGGTGCAATACATATTAAATTATCTTCCTGTTTTAGAAGAGCA
TATCTCTCTCTATAGTTATTGCTTATCTCATATTCTATCATATCATACGCTTCTA

TCTATTACTTCAATCAGGTAGTGTTGTTGTGCTGTGTAGTCTTTACAGTGGC
CTTTAGCAGACAAATACATGCAGACTAGAATGTGTGTCAGCACAGGCACTT
GGTTTCCCCTCTTCCTCATTCTTGGTTCAGAATATAATGGATTGGAAAATGA
AGCATTGCTCTAAACAAACCATGCTGTGATGCAGAGCTTTGCATTTAGTTTC
AGCTTGTCCTATCTCAAAAGCTTACTAGTAACTCTTGATGAATAGAGTACAG
TGTACATTTACCTACAAAAAGATAACTAACGTTCACAAgtattttaattgttatttcaattttg
cttctttatctTCAGTGTGTGTAGGTTAAGGGAAttctaaattctttttttttttttttgcatatttttttgaAGC
AGATAACTACATTGGCTTATAACTTTTACTTTGATAATGACTGTTGTGACCAG
AACAGTTCAACGTACATTAGCAGAgcatttagaatatttttaaatgaagtagCCTTCATATCTT
GCTATCTGGTGGAACTGCTATCACATTGCAATGGTATTTTAGAAGTGATAAG
AACCTTGTTCTTGCCCCACCCAGTACAGTTGCTGGCACTTCTAAGTATTATA
CAGATACCTGTCCTGTAGgggcattttctcttttaaaataaatgtaatagaAGTAGTTTTGGAATC
TCTGTGTACTCATCTTCAAGACTTTTCTTTCAGGCACAAAGTAGCATGTTTT
TATGCACCTGTTGTTGTGGATATCTTGTcacttttcctgctgctggacAGAAGGGTGGGT
GGTAAGGTCTGGATTTGGAGGACTagctttttcatctttttctgtatatttatatCAAGGTGTATG
GAATTGTTAAATGTAAGAGTTTCAACTCTGTAGTTACAGTATCATAGGAAAG
CAGCCAGTGGTAGTAGACACTGGTAGAGATCAAGTAAGCCACACTCTTAGC
TGCAATAAATAGATACAAAAGAATATTAGTAGCtttagttaaaaacaaaaataacctacaacaa
acaaaacaacacaaaaacatc
>TCONS_03977992
CAGGTGCTGAGGCTGTTCCGTGTGTCGCTTCACTCATACACAAGTTTCTTC
AGAAGTTGCTGAGTTTGCTGAAAAATATGACTTCAGCCACTCGTGCCCTAC
GGACGAACTGTGCTGCAATGAGAAACTTACAGCCACCTCAGCAGGTAAGG
TTCACTACTTTTCTCTACAATAGTTTGAGTACGTTGGTCTCCCTCTTCTTCAT
CAACACCAAAAAATACATGCATTGAAAACTATTTCTTCTGAAGTGACCTTTA
TTTGAACTGAGCAAAAGGGCACTGAACGAGACCAGCATTTGCTGGTTTGG
AGCTCTCCTCTAGAAGCTCAGGAAGTGGAGAGATTCTCACTTGTTCAGTGC
CCATCGGCCCAGATAACATTCTCTTAACTAGCTTCCTATTCAGCCCTTTATCT
GGGCAAAGTAAAAGGCCTGCTTCTGCACAGCCTCTGCTACAGTTAAATTCA
CTTTTGAATCCCTGTGCCACTGTATGttcagagagaggaaggagatcTACAGAAAGCTA
ACTAAATGCTTTAAGCGATTTGTTCCCTTTGATAATTAATGTCTGATGAAGG
GTGTGTGGTATTGGGTGAGCATGGATCAAGTAGATGTTTATCTGTAAATGTT
TGGgctcttaggaaaaaaaaagatgtagttGGAGATGGATGGTTTAACCCATTTGCAGCtgca
acattttaaattaatgttgATGGTGTGAGTATGCATAATTAATCTTTATGGAGTTCTGAAA
TAAATCCTTTAAGCACATGTTAAGAGATATTATTGAAAACAGTTCACAAAAG
GAAGTATCGTGGAAATTTGGTGTTGGTACTGCACTTAGATAAACTGCTGAA
AAGGCACTTGCACACCCCAGATAGTACAAACAACACGAagttcagaaaataagaaaa
atcacaatCAGCTTCACAGTTTCTTAgccaaattttctttaaaaaaaaaaaggttgaaatCTGATAATA
AGTAATTTATTTCACCTTCCCGTGCACTTATTTGAAGTAATATGTCCTATAGA
AACTTCTGCAATCTCCAAAGGTTGGTCTCATAGTTTCCAAAATTTGAATAGA
TTACAAGATAGCTGCACATTGTTAACCACCGAAGGGATGTAACGTGGCATC
TGGTGATATTTTGTCAGAGACTGAAAAGCCTGTACAAAGGAACACAAAAT
GTCAGTGTAGATATGTGATAAAAGTAACAAGCAGACATGGTCTGCCTGTGA
AGCTGTAAAGACTAGGTATGTCTCTTCTTGGCTTCTTACGTTTGAAGGCCTG

TGTTACACATTTGGGCCCTACAAGATTGGTCGTGGCAAGTCCTACACACCT
GTGAAGTGAGGTAATGTTTCAGCACCCAACATACAGGTTTATCTGAAAGAT
GCAATAAAACAGATTGTTTCTGAAATACTCTGTGTGACCTATGGCCTTCTGA
CAGCACTAATCCTAACTCTTTGTAAGAGGTTTATACaaataatcaaaagaaaaagtctCA
GTTTTCACCACATTATTGAATTACTTTAACAggtaatttaaaaatgcatttatatatgCTCATA
TTTATTAATATACCAATAACTTAGAAACTCAGAGCATGTGAAGCAGTATAAC
TGAACTGGAGATAGAGTTCTGTTGATGTAAATAACCTCAAGACATGGACCA
TCATTTTCTTTGAGGTGGTTAAGGTAAGGGCAATGTTAAGATggaaaaacattgaaa
aaaacACTCACTGTTCCCATGTTGCTGTACTGATACAGCTCTTGTCATTAATTT
CTTAATCATGCGTTGTTTTGGAACTGGTATACAGATATAGCAGTCACCGTGC
TGATAACTAACTGAAGCTATCCAAAACTAGGCCTGCATTGGCTGCACCTTGT
ATTGAAAGAACACTCCGAAGCATATTTTGATTATTGCCATAATGTTTTGTACA
TGCTTCAACTGCAGCTATTGTGAATGCGCCGAATGCTGTGAATTAAAA
>TCONS_03995295
gacagagagagatggTGAAAAGAACAGGTTGGGTCTTAAAAAGAGCCAAGATTGA
CTCAAATGAGGCAAGGAAGCTGAACAGAAGGGATCAGAGTATGTTTGGTC
TCTTTCAAATTCACAGGACAGACAGAAATCTCAAAGAGATCTTTCCTTGCA
AATGTCTCTGCATGGCTTACAGGACAGAATATGAGAATAATCCAGCACAAG
ACAGTGGGGACTGAAGAGGAAACCCGAATCTATTTACCATTAAGAAGCCCT
GTACTGAACTGACAGGTAAAAATGGATCTTGACTGAAGTACCTCAATTGTT
CAGGTGTTGCTGTGGCTTTCTGGCACTCTGAAAGAACAGGTGCAGCTCTGT
TGTGGCCTTCAGTAGGTTTAAGGCTCACATATTCTTTGGGCTGCACTTCAGA
GACAGTTAGAACACTCTGCAGATGAACTGACTTTGAATCAAAAAGCAAAG
TTGAGTAATTTTAATGACACATAGTTGGAGGGAGCATAATATTATAATTCCCA
GTCACATGTCCATGCTAAGTAGTTACAGATGATGAAGGAAgcttgcaaagagaaaatg
acaaGTTCAGAGCAAATGGAGAGAAGTAAGTTCTTTTTTTGGTGAACTATGC
TTTCTGTCACCTCTGATATGACAGCATATTGCACACAACATCCATTTGTTACT
GGAGGTCTCACGTTCTCAATTCTGAAGCTGTTGTTTATACTGAAAGGCGATA
GTGCACATCAGAAAAAGTCTGGTCTTCTTCAGCATTCAAAATAAGGAACAG
AATGTAACACTGCTCCAAAGTCTTGGCATGCCAGTGTTGCCTTTGTGTATTT
GAAGTATTTCCACTGGTCTTCGGAGTCCTGTCATCCACCAATGTGGAATCCA
CAACTCAGAGCTGTGACTATCCTTTGGAAGGAACAGACATGACAACCATCG
TCTTAACTGCACTGTGTCAGAGGAACCATGAGTTTGGCAGCAATCCATACC
CAGTGCATGGAGGACCTTCAGATGAATTTTTCTGCTACAGTTTAACTTGCC
ACGTTTAATTTGCAAGTATCAAGGTAAGGAGCCAAGCAAGTTTGCATTTATA
TTCAAACTCATTTTCTACCTTAATAATTTGTGCAGTTGATATGTTTATATTTGC
TGTAGAACATCGGGTTTCATTTCTAAACCTTGCCTTGAGTGCTGGCCATAAG
CCTACCACgaacttcaaaccatttctcTACATAAAACAGCATGCTCTTTTAGGACATAG
GGAAGGAAGAATATAACCTTACACCAGTCAGCAGGTGGCTTGCATTATTAG
TCAACTCtagctccagcagagctggaaaacacaGCTGTCCTGCATATGCCATGAAAGCTTG
CTTTACTCCGGGACCACAGTACTTGTGTTCAGCTCTCTGTTCTCTCTGtggcatt
tttctgtttgtcactGATTCTGAAAACTGAACTTACAGAAAAGCTTTGTGGTCTCAAA
AACCAATACTTAACTGTGttcaaatactttttcataGAACATGCATTAGAGAACTGGGC
TTTTCTGTACTCTGTTAAATTATGTGTTTGTTTATGTGACATAGCACTAATCA

GAAACTGTTTTTCCCCTTTAGGAAGTTGTGACTTTCTaaagtgaggggaaaaaatggaa
atagttATTAGAAATTGTACTTACAAATATCTTCACAGTTCAATTTGCTAATTTTT
AGTTTGGGtattgagttaaaaaaaaaaaaacaaaagtttaatttgaaaagcaatCATACATGTCATTTGT
CTCAAATGCAGCACAAACTTAATGCGGATGTATCTGAATTGTTCATTTAGTC
AGttttaaatcttatttaaaaaaaaaatacactgctgtGAAATGATGGAAATCATTCTGATTATAAT
CAGGAGGCTGTCAGCCCTGTATTTCCAGACAGGCTcatcacagcacacagcctgtCTT
GTGGAAGGAGAACTGTAGCAGCATGaagtggcagaagaaaaaaatattttctttgtcagtTGAG
TGTTATGTATAGGAGCGGAATATGTATAGATAGAACTTGCTCCAGCAATTTG
TGAGAtttgaaaaagaacaatttcCTAAGGTCTTTCATGTCTAgacttgtttgcttgtttgttgaatgaattt
tttgttttattgatatTATGAAGGCTGCTACAAATGTAATGGTACTGGCTTTAAAGTAA
GTTGCATGATTGGAGGTTCtggttggatattgggaaaaatatttcccagaggtgtgaggcattggaacagg
ctgctcatgGAGGTGGAAGAGTTACCGACCCTGGAGCTGTCCAAGAAAAAGGT
AGGTGTCCCACTGAGTGACGTGGTGCAGTGGTGATGGATTGACAGTTGGA
CTatatgatcttagtggtctttctaaccttgatgattctgtaatgcttcctatttaatGATGTTGACCTATGATATC
AGAGGCaaatgttggtagtatggcagtagaaagGCATCTAGCATGGAAGTGCTGATGAAGC
aaaaggtgtgtcattgaattcctccagtCGGAGAAAATGGCACccttgacattcactgacacttggtgagtgttt
atggagatcaaagagtgcatgtgagcacagggaggtggtggctggcatgtttctgcagtggcaacagtgggtcacttctgc
tAGTGCAAATTTgtatgagtgtggcatgcaggttcttgttcagtagctatgcattttcactggcGGAGGTGCT
atgctgaaaaactgtttttaactGAGACTTTGCCCCATCAAAttgtgttactgtgctctttgtagctggtgtattt
tctgtggaaataaataggaagcattacttccTATCTCTAGGAAG
>TCONS_04006350
GtatacaaatgttttattttattattgttacaACAGGTGCAGTAGACAAACATCAGCATTCTTCAT
TTGCATGATTTACACACAACGGGAATAACTCCTACGTAGTGGCCTCAGAAC
ACACGTCAGTCACATATTTCACTCTCCGTGGCCTTCACGATTCATAAAACCA
TCACCATCTACAGTACGGgtaacagaagaacaaagagcTGACATTGCAGCGAGGATCC
TTCAGGAAGCTTGTGGGAAAGCACCTTTCGCCTCTGACGTAGCATGTGAAA
ATACAGCTGGggaaagactgaagaaaaaacagacaaagGAAGAAGTCAGGCTTCTGTGTT
GGTGTAAAACACACTCACATACTCAAGGTCATTGattctgttaaaagaaaatctgcttctcC
ACTCTGCTCAAATCATCTACTGGCTAGAAACTAGCAATATTGCCCTTAGACT
TCAGgtattatttaatttttgttagaACGATGCCGTTTTTAATTAAAGgcaaaatgcattatttcataATT
AACTTGGCACATACAAGAAGAATGACAAAGTTTCTTGAACTGCATGCATAC
AATAAACAAACTgctttgtggggaaaaaaagcaccagGAGAAATGGAAGTCATCCAAAA
GAATTAAATTCACCGATTTGTTTCCGATCTGATGATTCTAGTGTCTCTTTTTC
AGGATCTAAAAAAAACTTTATACAACCTGGAGCCTCCTGCATCTCTACCCTC
CCTGcaaagctttttcctcctatttcgAAGGTTACATTACACAGTATGCTTGCAGAAGAAT
GAACTAGCGGACGTAAAACAGTGAAAGCTTCTATCTTTGTGAAGAATGTGA
AAGTAAAACAAGGTCAATATCAACATAGAGACATCCGTTACGCTTGCCTAG
TGTTTGTTGTGAGTGTAAGACAACCAAAGACAGCTTAATTTGtacttctttttcccta
aagttagcattttcttctttggcttTCTTTGCCAGTATTAAACATCTTGACTACCTCCTCCAA
CCCtgcctttcatttcatttaactCCCCCAGTGTTAAGTTGTGAATACATCTGTCCAGAAA
CATGGAAGTATACAGAGCCTCGTGGTTTCTGTTTGAAGTTTAAATAATTGGT
ACCTATTACATACAAAGAATTGAATGATAACTTCTCTGGCAAGATGTCTATTT
GACAAGCAAGAACTAATAAGCCAGATAAATGGTAACGTCCAAAATAGTTCA
CAGAAAGAATAACTGCTTATAGACGGCCACACTACTGAAACTTAAATCTTA

GAATACGATTTGTCAGTCAGCATAAGCAACAAGGTTCACATAAACACAGCT
ACAGAGATAGCtagcagcagtgccactgcatTATTTACACATTAAACTTCTATTTGCAC
ATCTAAATTGGCTGAAGACCTGCATGCTCTTAATCACTGTTTCTAAGCAGCA
AAGCCAAACCTTCTCCTGTGAAAACATTCACCAAAGACAGTATCAACTCAA
TATCAGTATCAACGCCTCCCCAGCCACTACCACAAAAGCAACATTCTTATTC
CTGAACCACATTTTAAGAAACTGCTTTAAACCTTGTTCTCCTAGAAGTCTGT
AAGTGGGATCAAAATGAAGATCTCAGTGTTTGCTAAGTACTACGGTTATTTT
ATGTATcaaatgtaagagaaaaaacacataTTACTAAGCTACAAGCATTGCTAGAAAGCA
ACCCATGCAGTTTTaactctgatttctttctttaaagaatgtCCAAAACGAATTTTGCAAAATT
AGTTGTGCACACGCTAAACCCGCAGTTGTTAGCTTTTCATATTAAAGAACTA
ACAAACTTGTACTTACGTGGAATGTAAGAGATCATAACCAGGATCAGGAAT
GTATAATAGTCAAAAGAAAAGTTGTACTTATTAGGTAAACTAATGGAATACA
AGCCAGACTGCCTGACGAAGGGTAATGCAGCATATATTGTGAGTAATTCCC
CAGAGACTCCCATTGGATACAATATTATAAACAAAGTGTAcctacaagaaaacatttaa
gaaaaggaTTAAAAGTTGTGACAAAGTACTTAAGTACCTCTATGGATATTCAAC
TTCTAAATCCTtaacttttatttcatttgagcATGGCAGTTTGTGAACATCAATGCAAGAC
AAAATATTGACATCAACAACCAGTTTTGAATTATTTGAGGACAATCTTTGAG
AACCTGAATGGTATCTTTACAATAATTCCCCCACCCCCATACTTCATTGCTTT
GACTGCCAAACTCTTTGATTGAGGACATCAAACTCCTTAGTTGAGAACATA
TTATTAAAACAGGGCCAGTCAGTATTAATTAATGAGCTCACTTGTTTCAGGT
ACAACCCTCAGATTCAGTGGTTAATATTAGTTTACTTAATATAATCATTTCAT
AAATGGTACTGAGTTCTGATAAAGTACTTTCACAGCTaacatttaatacattttaaatccA
GAAATCTGTTTGAAGGAAGCTAGGAAATCACATTTACAAACAGAACAGCTA
CCCTGAGACAGAATGTaccctgctttgctgcagctaCGTTACCGGAGAGATACCTCCAT
CAAAACAGTGCTTCAaggctatttcttttttaattgccacCTACCTGGCCCATTTAATGAGA
TAAGGGAGATGGTTTAACAAGCTGAAGGTGTAAAAAGAGTAACGGATTATC
TCTGTGATTGTCCAGGCCACCACAAACAACAGGACGCTGTCTTCAGTTTGT
ACCTGCAAGGAAGGAGCAAAAGGTTAAATATGAACACAGTGTTACTGATTA
TTGACCAGCTTTAAACACCCCAAAGAAAGTAATTCCCATGGGCTGCTTTAC
TGCATCAGTTTAGAAAACAGACTCTAGGAAAAAGCTTAAAAGCCACATTAG
TAACGGCAGCAAAAAAATCTCTTGCCATGCTCCATATTTTTCTAGTTGGCTT
AAGAATAGCTCTGCTGATCAGATACTGAGAACAGCTTGtaacttttcctttccactccTA
TACAGTACCAAGTTCTGCTTCAATCCTGTGATTGCTTTCTAGCACAGGAAGA
TGCACTCAATGCTAGCACTCAAGTTCTGGTCTAGATTTTACACTCCAATCCT
ACCCAGTGCTATTTCTTGAGTAAAAGGGAAACTATTTTCCTCACTCTGTTCA
AGTGAGAAACTGGCTCTAaattctgagcagcagcaaggcagcgATCAGTATAGGTCACA
GGTTCAGAGCAGGGAAGAACCAGAAGCTGAGTTTGCCTTGCTTCCGATAA
GCTTAACAACTACCTGCTAcatccttccctgctccttctcccCACTTACTGCACGCAACAT
TGTTCCTCTAAATGTTACTGTTACGCTCAAGGCACCACTTTGTTTCACTGCT
TTACCCCCCTTAGAGCTTGAACTGTAAATACATGTTACAAAAACAGTTTCTA
CTCCCCTCGTGTTTTGAAGATTAACCTTCAaaagcatgtattttaaaatgaaccaTTAGATT
TGGGAGCATAATTATGCGTTTCAGATGGATAGCAGATATGAGGAATTATATAG
GGTCACATACCTGCACAATAAACTCCAGAGAGTGAACACAATTTTTTATGC
AGCACCCTCCCCACCCAACAGAAGCATGATGTGAGTTCTCAGCTGTCTGAG

TACCTTCAGCCCAAATTCTGTAGACCGTgggaacaaaaaagagctcAGCTACAAGG
AGCTGCATTTACTTTCCTCCTCCAACTcttgaaaaagggaaaaggaagtcAGTAGGTGT
GGGTGGTACACCCTATTCATCTTGGGCAGAGTACATgtacaaacaaaggaaaaagaaaat
atatgggCAAGCAGAAACCTTTTAATAGGAAGGGACTGATCATATACTTGTGTA
TAAAAAAGTTCACAAGGattactcattaaaaaaaatccctttgttACAGAGACCACAGAATC
CAAGAACTGTATAAAAATGTTGGGAATCCTGTGAAGTACAATTACTGTTATT
CTATATTCTGCTGTGTATAACAATTAAAGACACCCTTATGGCATTTTGCACCA
AACACACCTCATCCTTTTTCACCTGAAACCGAAAATGTCCTTATATATTAGC
CTACCTTCCAAGACCATAAAAGAACAATATCCAAAACCTATTCTAagactttcaga
gaagaaaccaaatttatttattcacaCTGCTTAAGTAACTAGAAAAGGATCCTAATTCTGCT
CGTGGTCTGCTTTATAACGAAGACTTAGTTCTCAACCTGGAGGTCAGCATC
ACTTCACAGATGACGCTGTTCCCAGGGAGCATCACGCTTCACAAGCACTCC
AGAGCTGCATCTTCCAGTCAGATCAGGACTAGCAGCTTAGTGAGTAAGCTG
AACCTATGCATCACAGACTTCAACCTGTGCTTTGAAACTCCACTTCTTTTCC
TAACTGTGGAAGTCAGTAGAACCAACAAATGAAAATTTGTAGCTTGAAGA
AGCACCCAAAGAACCAACAAGAAACTGTTAGGCCAACAGTGACTGATGCA
CGTAGAAGGAGAGAGAACTGAAATTGTAACTGCCAGATGGTTGCAGGACT
TATAACACTGCTTTCTCCAGTCTAGTCTTTTCCATCGACCTTACTATTGGAAC
TGTGCTTGGAAATTACTGAAACAGCTCACACCATCAAGAACAAAAGGGTC
ACTGAAAAGGGAATTACAGACTGTGTCTTACAGTTTGTGTCCCTACTTAAA
GAAGGAGGAACGCTGGAAACTGGCAGTTGTTTTGTGCAAGAGTGATCTAT
GAACTTTTCAAACACTCTTTCATAACAAGGAGCAGAAGACTACTGTGCATT
TTATCATCACATTTTAACCACATTCCACAGCTCataattaaaaagtatatttcttACACACT
AACAGATCTGCCGAAAAACTGTCACTATTAGAGCTTCTCTTTGAGGACTGA
CAGTTTCTCAATAGTTTAGATAAAGATGTTTGAGATGTACATTACCAAACTC
CTGAGAACTGATTTACAAAACAGTCCCTCAAGGGTCTTATTCATTTAGGCA
CTACTAATGCTGCATTTGTATCATTCCTCTTCCAACCTCTGTGTAAGGAGGAT
AATGCAGCCAATAAACAGCAGACCCTTTTTAAACCACAGATCTCAGTGCTG
CCCTCAGAACccaatttttcttcctctaccTTTTCATTCCAGTCTTTGAAGGCGGTGTTA
GAAAAAGTCAAAGtatgttttacattttatatacaACTTATTTGTCCAACTGCCTTAATTAG
GGTGAATTATACCATGGGTATAATTTTTGACATGTTTCCATTTATCATCCAGG
GGGTCTTGAACATATTTTAGCTTGCCTTCATGACTGCAAGTAAAAAACAGTc
ttgaaatacaagaaaatttATAGCCTTTTAAGATGCAAAATTGAGATTCAGAATTGGGaa
tcaataaaataaagccTAAGATGCTTTCACTGTTCTTCTTATGCACAAAAGTTACAAG
ATTTGAAAGGTATAAAATATCATAAATCTAGAAATATGCAGAAACCTATAGA
ATTAAGAGGAACAGCCTGTCAAATAGGTTAGTGATAATTTCAGATAAATACA
TCATAACAAAGAAAGGTAAAAACAGCAGCACTATGTGACAGAGATGCTGA
AACACATCTCTCTCCAGAGTCCACAGCTTGGGTTTTGTGGTATACCAGTTAA
AAAACACCGCAGCCTCACAGGAGGTACAAAGGGCCTGACAGACATTTGTG
TTGTACAGAATTACTCAAACTTTACACAGATGTAAAGCAGTGATAAATCAG
GCCTGTCAGCTACACTCACCAATATGGTATTTGGAACTTCCCAGTGCATGCT
TcttatttaaattgaaatgcTCTTCAATCTTAAGTGTAAGTATCCTTTACTATTGATGAAA
GATGGCAGGTTTGCAGATGCTGGAGTCTTAATTATCTGCTCAATAAGCTGTA
CATGTCTTTCTCAAACTGTGCTTTTTGATTGTGTTCTAGAAAGGTTCCTCTA

AATATTTGCAAAGCTACCTTTGTAATACTATCTTTAAATCACAGCTAGCACCA
TTTCTAATTAGGTGTCAGAACCAGGTGCAACACTGAACTCCACAAAGGCCT
TCTGTGATGGCACCAGATGATCTTACTGTTCACATCAATTCAAAAAGACAA
CTTTGAACTGAAAGGcaccttctgttttcctctccctATCTTCTTTCAGCCCttcattctcttctctcct
ttgcagaagaaacagcaaaattcaATAAACCACTTCGtttccaaaatgcttttctagAGTACAAGTGCT
ATATTACACACCAGGCCCTTCTCCACTACATTAACAGCTAATTACACTGGGG
AGATGAAGGGAAATCTAATCATTGCTCCAAGAAGTGTGCAGTGCTTTAACA
TTTGCCATGCtttaacatttataataaatactgaatttaaCAACACATCAGTACTAAATTcaacaaaa
agcacaaacatgTTAGCAGCAGGAAGCATGCTTCAAATCACATCAAGTATCTCAA
AAGTATATTTGggcagaaagaagggatgcaatacaggtttttcttcttttaacctCATTAGATATGAAAC
AGTCTGAAAAAGCAAGATATTGAACAGCTCTCAGTCAAAAAGTTAAAGAG
GAAAAttgagttttattttacaaGAACCTCATCATGCAACTGCATAGCTTACAAGTTCCA
TAAGCTGTGTGAGAATTTAATCCTGGACTTCATCATGGAGCTCACTTGTTCA
GACAGCCCTTCAGAAAGAGTAGAACACTTGTGACACTTGAAGTACATCTC
CCCAGGCAAGACTGCACGCTCTCTGCTTGGGGTGAAGATAGACTGATGGG
GGAAAGAACATCACCTCTTCATTTGACTTAATACTTCTAAGTCAGAGGTGA
AAAGCTCGTCACAGGACTCCTTATCATCCTTATGCCTGCTTTCtgagacagtaaaaa
aaaaggaacacataAGACAGTCTCTCTCAAGACTTTAACCAAACAATCACACAAgtg
tgagggaaaaaaaacccacgtgCAATATTCCTGAAATGATCCTTGTTAAAAATTCAATG
GTGGGCTAAACGTTCTTGTATATCCTCTCAtttcactgttgcttttttttttcctggtgaaaATGT
GGGCATAAGTTTAAAAAAGCCCAACCCACAATCACTTTGCAGACTTATGTA
GCATTTATCTCCACCGAGCTGCAGAGAATAGAATACTTCTATGTgcaccaaaaataa
aatctgtcagaacataaatatttacatgaTTTTGAGTCTCTTGAGAAAAATAACACTCATTCA
CTTCAAGTCGCAGAAAACTAAGGATCCACAGACACAAACAATATAACATCA
GAAAGGGAAGTTAATTATTAGGATCTCCAGAGACTTACATTCCTCTGCATAC
GAATGGACATTCTGATAACAGGTGTTGgagcaaggaagagaaagcagcttcccTAAAGAC
TTGGCAAGGAGACAAACTCCTTAAGACATCCTCCACGTCAAGCCACAAAA
CATCTTTCATAGGCACACATAACACCACTAGATGTCTGTCTTTCAGTCAGTC
AGGCTGACTGTGAATCAAGACtaatttctttccttaatgAGACAAAAAGATCAGCTGG
CTACACTAAAAGCATAGATGCAGCATATGTGAACTACTGCAAAGTATTCATA
CGATATGAAGTCAGTCAGCATTATCTATATCAATTAAAGCACATTCACATATT
AAAATAGCCATCCACTGGGAATCATTTTCAAATGATTGCTTCCAGAGGATTC
TGCAGACCTCGGTGAAAAGTACAGTGATGTTTGGTAATGGTAAGCAATGCG
AAACAACCAGAAATCCAAACCCGCCTACACTTGAATGCAAGAAACACTGA
CGACAGACAGAGGGAAGATTATCCTGGAAAAGAGATCACGGAAAACATAC
AGGAATTGCCACGAACAAACAGTTGAGTAGGAGCTTCCATATGCTGTGGAA
TAGCAAGTAAATCATTTATAGATAAGACAGTGAGGACATTTTGCTATTGCTC
CAGCAAGATCAATACCAGGATGCTGCCCCAAATCCTGGGGTCCAGGTGGTG
CATTGGAACCTCTTCAACTTGTAGTAAAGACTACAAAAGCTGTGAAAGACT
CAAACTTCCATCTATCtgatttttgagaaaaaaaaaaaaacgtcaTAATCTTATTCCTTTGATCC
ACGTCAACTCAATTCTACACGGTGTGAAACAACAGAAGACGTTTGCAGGA
AAGTCAGCAATCAACAAACGCTTTTCAGGATGGGTCATTAATTTCTGTAATC
TGTTTTGTAGCATAACTTACCTCTTTTACACTATGTGTTACTGCCCAGGTCAG
GAAAACCCTTGACATCACTTGGAAAGCAGTCAGAACAACAGAAGAGGGA

ACAATGCCTGGAAGATATTTTGCAATTAGTAAATATCCCTGACCATTGGAAT
GAGGGCGGGGAAATAAAGCACAGCTAAGTCAAAGTCCACTTACTTGCAGT
TTTTGGaaggaaatcattttttcttttgaaatttgAGACTATGAAGCCTGTCCTGTGGTGTattca
gagctcagcacagcgcAGTCCCCTGACCAAAACTTTGTAtatcaaaatgcaaataaaagttTGCA
CATTGCTCAATACACAACACAGCTGAATGAGATCGTTTTTGGGATACAATCT
CTCAAATAAATCTGATTCACTAAAATAAACATACAAGATAATCTTTGTTACTA
TGATTCAGAAGAGATGTTTTGCAGAGTGTACCCCAAagagcagcaaataaaaaacCC
CCCATCTATAAACAAGCCAAAAGAGGCTGACTAGGAACCAACCAAGATTAT
GTCACAGTAAGacaaacaagtttttttgttatggCCAAGACAATGTCAATCTAAGCCAAA
AACATGAGATCAAATTCAATGACTCACTAGCTATAATTTATGTAAACTCTACT
CACCtctaattatttattaattgcTCTAAAAAGGTACCCAACAGACAATTTTAAACttgacatt
ttcaaaacatctgTCACCTCACACCGTGCATGCCAGAGCAGCTCTTGGCAGACAGA
GGGCTCCCCAGCCCAGGACCAAGCAGTAAGCCTTGCCTTGCTCAGACCTC
TCCCATGTACTGCACTAAGCCTCCCACCTCCTACAGAAAATCCTCCAAGCT
CTGACAAgttctccctctctcccaggAAGTTCCCAATTGGGAACAGGTTGTATGCATCA
TAGCTTAAAGGCGGAGCTCTTACACCTCTCCTCACCAAGCAGGCTAAAGCA
AAGTCAACCAGTGCTGACACCGGCTTATCAGAGGGTTACTGGGAAGCAGT
TCTGCAGCCATCTGGAACACAGGACCACGTCTCCTGCCACCCACTTGTGTA
CAG
>TCONS_04020237
gagatttttcccagtgcttcAGAAACCTTCTTCGTATCACCTCATGTCAGAAGCAATTGT
ATGGAGTGGATGTAAAAAGGCCTGGGACAATTGCACAGTTCATACTGTAAA
GATGAAAGCAGCCTGCTCTTTCTGGTACATCTCCGCTTTGATCTACATAGTG
TGGAAAACTGAGACCTTGCCCAGCATAATGCTGCAGGCTTCTGCCGTGTTG
CAAGTCCTtggaacagagagaggaaaagGTGCTGTACTCATACCATGGCAGTTACAGA
AGAACAAAGACTGGTATATGCTGAAACTTGTCAGTTGATGAAGTTAGCTCT
GCAAATGGATTTAAAAACTTCTTTGTCAAAAAAGTGGATGCTTATCactcaattat
ttttatgtcCTCAAGCTGAATCTATTACTTATTCATCACAATCGTAATACTAAATTG
AGTTACCAGAAGAGGTTGAGATTGCTTTTACTTTCCATCCTTCAAGGGCTG
TGAGGCTAGATACGGCAGGTACTCTGAACCTGTTGCTTCAGGACCCCAGGA
AGACTCAAGCCTTTCCAGTATCAAGTTGTTGACAAAGATGAGTCCCTGCCA
GTAGGATCCAAGGACTACTTGTGTCACTGCAGTGAGGAGGACATCACGCA
AGTCACATACAACCAAATTGCAAGTTAACTGCCGAGCTCATGAGGGATATG
CAATTCTACAAACCTCCCAAAGTAAACTCACAATTTGCGCTCCCTGAAGGC
ACTAAAAATCAACGAGAGTCATGGAATACTGGAACATATGGAAGAGTTTGC
CACCCTTTGCACAAACACACAAGACACCATTACCCTACAGGGCTACATCAT
ATTTATGTTTGCTATTAGAATGCACCTCAGTAATACGCACGGCCCCATTCAA
AGTGAAGCATGGCAACACCTGGTGTTTGTGTGAAACCATCCCAGAAGGTTT
ACAaattcaattaagaaaaaacGCAATGAGCTGTGGCAGGGAAGATAAGTGCTATACA
GGCAGGAAGGCATTACTTATAGTGACAAATCTGCTGGCACCACTCAAGTGT
CTGTGCCTAAAGAAGGAGACTGAATGTGACAGGAAAACAACCCTTAACAG
AAATGTTATTCTTCTACTTTATCGAGATTTCTTAGTGACCTTTGGGATGCAGT
TGCAATCACACATCCTTAtctgttacattttaaattGCTACTGCTTGCTTTCACACACGC
ACCAATCCAGTTGAAATGCAAAACTATTAAGATGTACATAAAGTCGTGAGG

TATCTTAGAGGCTcagataaaacacagaaaatttcAAAACTTCAGATTCCactaagaatgaaaaat
aaaaataataaaaaaacataccTGATGAATGCTTACATATGTCCTCTCAGAGTGCTCAGC
CTCTGTCTGGTTCCCAATGCAAGGCaaagaatcgtagaatcattgcagttggaaagaccactaagat
cacccagtcaACTCATCAACCCATTACCACCATAGCACTAACCCAGGCCAACCAC
ATGTAGCTTCCTTCCCACCACTGTGAGTGGAAGACTCGCACATTCAATCATC
ACAAAGCCAACCAAATGCATCCCTGGACTCACTTCAGCCTTTTCCAGCACC
CACACTAACAACAGCCCCAAGGAGCTGTGAGAGAACACCTGGGAGCCAG
CTTCAGCCTCAGGTACCATCCAAAACCCTTCCCTTTAGAGTTCCCTGTTCCC
TACAGAGGGGAAGCAGAGACAGGCTCTCCTTCAAACATGTagctcctttcctctcctc
acTTTCCTAAAGAGGTCCTTGGGGTCCTGCTCCCCAGGCTGGgtgttctttccctttttcct
gctctATGCCATGTGAAAGGAAACTGACCACTCACAGAGGACTGATGCTGTCT
TCTTGTCAGATGCTGTGTTGTGCAGTTGCACCGCATTGGACGCATTTTGCAC
AGCCCCACAATTTTAGTTTGTGTTATAATTAACAAATACTCATTGACATagcaaa
gaaattaaacattCTCATGCTATTCACATTATACATTATTAAGACAGGGAAGAGTCTC
CTCCTAATGCTGCCTTTGAGCCACAGTTTACTTATTCCACTCAAGATAAACA
GCACCCTGCTCTGTTTCCCTACCGTGACAATGTCTCTGTGAAGTAATGTGCA
GCTCATTCTAATTAGCCGAATTTCATGCTCTCCTACTTTGTTATTGTTGAGCT
CTTAGTTTGCGCTCTGGGCTTCAGTGCTTTTTTGTGGCcaagaaaacacagtgattGC
CACACAGGATTTCTGAACAGCGATCCTTgctacaataaataaataaataaaaaacacccAACC
TCTACCTTGCTTCCAGCTCAGAAATAAGTTACTGAACCCAAAACCGAAGGA
TA
>TCONS_04126449
AATTTCAGTGAGAATATCATGAGCAAGCAGCCTAGCACTACAGGAAAACAA
GTTATTATTTGGGTCGAACGGCTGTATGAAAAAATGAATCAGCAACTGTTCC
ACAGAACAACAAGGCTCTCTGCTGAGATTACCAGCACAGCCTCTCATTAGG
GCTGAAGAGGACAGTGCTTTGTGCCATGGAAGCACATTGTTGTGCTACTGT
TAACCTGCTTCACAGACTGTTCTACAGTGTCTGCCTCCCAACTGGCACAGG
TGTTATGGTAAAAACACATTGCTACCAGAATGCCTTCTGAGACTGTTAGATT
TTAGCTGAAATAATGGATCAGACGCCACCTCTGTATCTGCAACACACATGCA
TAATGGAGTCAGCCATTATGGTAATGAAATACATCAGAGACATTAATTCTGT
ACTTAAAATATGCATCATACCTTCTTCATCTTCCAAGGTAAGCAGTGCGTGG
TTTTGGTTCAGTTTGAATGGGATTCTTGTTGTTACAGAAAAAACGCAGCGG
GTATTCATATCTGGAAAGTCACCCTCTGAGTCTTCCATGTTAGTGAACACCA
CTTTCATTTCTGCcaccttttttttaat
>TCONS_04164379
ACCTTGACCATCTGGGTGCAACCCAAAACACCCTCCTATGGATGCCCACCT
GAAGAGGAGGGGCCTTTGAAGGCCTTATAAAGAAACCAGTTTGCAAATCA
GTTTAAATACAACTTGCTAAAAGGATATATACTTCACGCTGACAccatcctttctttct
gtttatagCCCTTTACCACTGAACTGCTGTGGCTTGATTGTGTGAAACTGAGTTT
CAGGATGCAAGATACTGTTCAGATAGTTCTGCAATACATTCTTCAGACCCAG
CAACTTTGGCAGGCCCGTGGATGATGGAATAACCAGATCTCTGCGGCCACC
CCTGAAAAACAGATTCTTTCTTCTGGATTCCCTGGGATTCTCTCAGCAGCC
AGCCTTGTTTACAGAAGTCAGCGCGGACAGTAACAGCCCTTCACCAATGA
GCCACAGTGGAACCTTTGGGGAAGGCTCCTCACGGTGCTCTGTCATCTGCT

GACAAAGTGGTGCCAGGACCAGAACTTCACAATgaactctgcagagaaaaatccaCAG
CAGATGAAGGAATCTCGAAGGTATGGACCTGCAGTAAAACTGCTGTAAGA
AATGATTTAGTTTATCTTTATTCACCTCTGTAAAGGCTGAAGGCTGAGCCTT
GTGGCAGGCTGTGATATCACCTTCTTAGTCGAGtt
>TCONS_04175191
ATGACATGGAATTAATACAATGTGCATCAGATGTGTATTCTCTTTGTTCTGAG
TCTTTCTGtatagtttgtttttctgcatgctGGTCAAGTGAACCTATGTAGctgaGGCACAGT
GGACGTCAAGAATGTCAATTCTTAGAGCGCCTGGCCAGTTATGGATCCATC
AGAACACCGTGGAAAACAGTGTTTGCTGCCTCTGGGGGAGGAAAGCCAA
CCGAACATCAATGAATGACTGTGGGAATTCATAGCAGGGCTGGAGAAAAA
CTGGGATAGACTCAGATAGTCTGTCTTGTCTCCATCTCATTTTCTAGATATAA
ATGTTACTATTAAGGAC
>TCONS_04195253
TAAGCAAGGTGGATGGATGCATATAAAGCAAGAACCATCAGACACTGGTTG
ACATGCTTTCCCTCAGCCCTTGAAGAACACTCAAATAATAGTAAATTTCAAA
TcaCAATGCCAGTCTGCACTGTTGTGAAAGACCTGTGCATAAACATTCATAG
GGATGAATTCAGAGGTGATGGGCAACACAGACTGAGGAAAAGGAGCCCA
GAGAGTAggtaataataaagaaaatcatgaaaagaggaaacagaggaggaaaagcagctaAGTGAAAGG
TATGTAACACCTAATCTAAAGcacatttctctctccttcagcGCACACAAAACTTTTACC
GGTTCCTTTTTATGGTTATTGAATGCCCACATTACCTGAAAGTGGAATTTATT
GTGCTGAGTGTTAAACTCTAAGTTTTACAAATAAGCTTCcgtacaaaaatatttattatct
gTAACGTACATCATCAGCTCTTACACTCCCACTGCGTTCATCCATTGTCTCCC
AATACAGTTACCGTGAACGTATGTCAGAATATACGTCAGCCTCAGAAATACA
CACTTGTACTGCAGTGACTATTTTCTATTCCTTATTTCTTTGCCTCTATTTTAA
ACAACGGATGCGTACGTTTGCTTAGAACCTAGCTTCTGAAACAGAAGGAC
AGAATTACTGGTTCTGACAGctgtgaaaacatttccatAATACAGAAAATCTAAATGCC
TCTTCATTCAATCACTTTCTCAATGAAAACATCCCAGGATTAAAGAAAtccagg
gaaagagaaaagattaaCCTAGAAAAGGCAAATAAGCAAGTGAATAAAGCTGGGTT
TATTGCTTCCTGCTTCAACAGTGTCCAACAAGATCTGGGCCATGGTGCCAC
AGCTTTCACACGCGTGTGCAGTGGACACAGCTCCCCTGCATGCAAACAAT
GCACCAGCTTGCATGAAGTGAATTGGGGCCAGCACAATTGTTTGAAGGAG
GAGGGATGCACAGTccctggaagcacagaagtgcctTTTCTCTCTGGTTTCATGCAGCT
GACATACCGAGCAGAGGAGCCAGAGAGAACatgtaatggaaataaatgactTTGCAGA
CCCAACGGAAGGAGGTGGGAGCGAAAAAGCATCCTCACCCCCTGCCCGGC
GGCTGCGTAAATCTGTCCCTGCCAGGAGAGCTCCGGGAGGTGCTCAGCGG
CCGCCTCCCCAGGAGTCAGTGAGAGTTCAGGTGCTTACTGTGTCATCAGGC
TTGACCTTCACATGCGACACTGCCAGGACGTTTGTTTTCCCACAGCAGTGA
TTGCTTCAGTCAATTATTATTGCCCATCGTAGAGTGCAAACACATAAGGGCT
TGATTTACAGTGCAAAAATATACATCCTGAATTTActtcctcttttgtttttggACTGTCAT
AGCATCTCCACCTTAAAGAGATGACAGCAAATTACTTCATAggtaaatatatgtatgtat
aggGAAGGATAAATAGCAGTCATTCGGGTCATTGTGAGATGAACGTTCAGTA
TAGCTTCAGATCGGCTGTGTCAGACGTGACAATATATTttaagctttttgttttaaaattttcat
tttttctgcttttctttgaagaaatcaTCATTCGCAAATTTTGCAAGTTGGCAATCTAACCTCT
AAATCCATTCTAACGCAGATTTAAGATTGTGCATTCAGTTCCTTAAGCAATG

TTTAGGTATTACCAATATACTGAAAGATAGATGAGACAGCCTCATGGTAATA
CAGCTTTTAACCTTTCCTCTCCTCTACCATGACGGGGGAGAATTACAGTAAA
TGATGGTTACCTTGTCTGCAGTATGGACAATTGATTTGACCTCACTGCATCT
GATGCTCTAATGGACTTTGTGGCTGAATCCCTGGAGATATAAAATATGTCCA
AGTGAATTGGGGCAGGTTAAAGAACGGACCTGCTTTTCATGGCTGGCTCAA
GCTATAATAGATCTCCCGGTTGTTCTGAgaagaagattaaaaatgatCAATATccaaatcatta
aaaaaaaaagcggtGGCTTCTGTAAATCTACCataggtgggtttttttcctgctaaacGCCGGAGGA
GGGTGTCTGGGTTGATAACGTGAGTTTCCATAACAAATGTTTACTTCTGTTT
ACTCTCTAGTAACATGTTCCTTTTTATATTAAGACTCAGACACACAGATGGC
GTGattcagcactgctttcctgaAGTCATGCTTGTTTACACCATGAAGAGTCAGGCCTT
AGGTTTATTTAATATAGATAAAATAACAATACTATAATATTtcctgaagcagaaaataaaa
tcaaggaCAGATTTCAGAGAATGATGGTAACTGTTATAACTGTGAATGTCCTCA
CAGTTCTGCTAGCCACAGTGTCtaagacaaaaataaaggtTTAATTTAGtgaggaaaatagaa
aaaaaagattttccttgGCTGCTCTGAGTCCTTCATTTGGCAAAGTGCAATTGAACAT
CTTGTTTATCCTTTCCCACCCTCTCACGAACATCTCTGCTCTTTTCTCACCAG
CACACAACTGAGCTCAAGGTGAGTGTAAGTCTGTCCAGCCCCTGGAGGTG
CCAGGAGAAAAGCTTTCACAGCCCACAGCTGGGAGCTCACCCACAGACCT
TCCTCTCTACATACAGCAGCAATGGGGCAGGCAACTTCAGTGGGCTTATAC
GTATGCACATGCTTAGCTTCCTGTAAAACCCATGCATTAAGGAGACAGATGC
ACAGCTGTTCCATTTGAGGCATGCATGTATTGGTTTGTGCACTTATTCAGTA
AACTTGAAATGCAGAGGAGCCCAAATCTCTGTTCTGAACAGTTTGAGGTTG
TTTGGATCTGACATCTCTGTGCGTTCACTGACAGAAATAAGCTAATTATGAA
CCTGTGTTGGAAGAGGGATGCCCTTTTTCTGAGTTTTGAGAAGTGTCTGGG
CAGAGTAATTGGACTGCCCTATCTCAACTGCGTTACAGCAATCGAGCAAAT
GCGAAAAATCTTCcttagaaaggaagaaatatgtgTTTTTAAGTGCTAGGACAATTCCAC
GTAACATCACGGTTTGCATCTATACCCACAGTACGCAGAGATTCATGCAGAT
GTCCAACAAATTGAAAAGgcttcaaatatattttatctaGTTGTATGTCTAACAGTATTTA
CCTTTTGCCTTTCACAGGTTGCTTTAAAATCTGACTATGTAATAACATCCTCT
GAATGATTTAATTTAAGCTAACGTGCTATCCCTTTATGTAAAAAGTAAAGGT
ATTTTGAAAGCCCTTGCTGAATACTCTGAGGTTGCCTCTACTGTGTGGCTAG
CACTGGGCTGAATGCCTAGCATCCGTGCGTAGAAGAGGGAACAACTGCTG
CCCCTCCGATCTGGCTATTCTTGTATCTGGAAACATTGTCAGGTGCTACAAG
CATCACGCCATCTGAAAGGCCAAAGTTGAGTACTACGTTATTGGAGTAGAT
GACACCTTATTCTTATTCTTCAACAATTCTAACAACTTAGAGTCCCTCACTG
TCCCTTTATGAACACAGATGTCATTCTGAGGGGGGGGACAGCCAAAATGAC
AAGAAGGTGACCTGTCGGTGAACTCAGGAGCAGGTGAGGCCTGTCAGCTT
CTAATTTGAAACTGGTGTCAGTCCTGAGGAATGGCTGGTATAATCACATTTG
TAACTGATATCCTATAAACGCTGCAAACATTCACAGTGCGCCTGTTATGAGT
AATATGAAATTAACAATTCGTGTTTATGACACAAGACACCGACCGAATCAA
GCAGGTCTCCGTAACTGGATCAGCTCCGTCTGTCTGCCAGAGCAGCGCATC
GAACTGCTTCAACCAAAGCCAAATTACTGTGTGCTTATGTTACAATAGgcattg
tatttattttgaagacaaaCTTTTGTAACTAGGCGTAAAGTGAGGACCTCAGAACCAGC
TTCTCGCTCAAGTTATTGCATATAAAACTTCTCTTGCTCGAGCCCAGCCATT
AAGATGTTCGTGACTTTTAAGAGTTTGGGACCTAACAAATCACGTGTTGTAT

CTCACATTTTCTGAATAACTGAAGGGAAGGCTCTGTGTAAATCTTCTCTCTC
AGACACATAAATTACCAGCTGCGTGAATACTGCCATTAAGACAAGCAGACA
AGTGCGAGGGGTGGGGACAGGGCAGGATGCCCCAAAGAGGTGGAGTTAC
AGAGAAAACAAGGCATTTGCTTACTGAGTAATTTATGCTTCATCTGCCTTCA
CTGTAGCTCAGTAAGAATGAACACCATGAGGCAGTCTGTCAGGCGCTCTCA
CTTCACTACCGCCAAGGCATTTTTCTGCAAATTAGGTATTTGCTTCCTATTTC
TAGTCACTTCCACCTGGATTTTTTATTAGGAAAAGTCAAAATCATAGTATCT
GAAGCTAAGATTTGAATTGAAGTTACACTAAAAGATAAACTGTTGCTCAAG
TATATTTCACATTTGAAGACAGCAAAATCTCACAATCCTTTGCACAATTATTT
CCAGTCCAAACTGACAGAAGCTCTAAAGTTCGGGTCCTttcctgcctgcactgctgctg
agcctgGTGCATAGATTAGAAGTTCCTTTCCAataacaagcaaaataaaagaagaacCATACG
AAGAATCTATTGCAATTGTTACACTGGAATTTAGTTAGCAGAGCTACAAAA
ACGCAAGTCCAAATCCCAAGTAGCTAGTGGAGATTTCTGAACAGTTCTGTA
CACCTTCACGTAGACAATCCAGTGGAGCTAACAACCTACTGTAGCACTCTT
TTGTGATAGGCAAATaccattcatttccattttccacagAAGAACTGCACTCCACAGAGCT
TCAGCAATGTAAACAAGGCCAGGTACAGCATTACAGCTGTGGTTCAGGCA
ACAACATTCCTTGTCCTCTGACACAACACTTACACATCATACTTGTAACACT
ACCTAGTTCAAGTTGGTGGTCAATACCACCTAATTAGCAGAGTAAAAGTGC
AATTTTGAAACCAACTTACAAAACTGCTACAACCAACCTACAAAACTCCAT
CATACCAAAGAAAAATTATGGCTCTAAATTTCACAATCACTCTCTGCCAACC
AAAAACTCACCTTTAAGAAGAGTCAGGGCTGAAAGCTGAAAGTTGAGGCA
GTCATCTCTCAACGTGTAGAACTCCTCACGTTGCTACGAAGGTACAGCACG
GTTGTTCAGTGTTTGGATAAAGTACtgcatgggaactgttgaatcacagcctgaaccaccaattgatc
acctgaggcaaggactgaatcagctgtgggagcacaggtgaaagcaattcagcTATGTGACCAGAAGCT
GTGTGACCACATTCCACCCCTcccagaccccatttaagggctgactaaCACTGGGGAAGAA
TCTCCTTGTGGTGATCCCTCCTTTGTGGAGCTTTTCAGTGTGAGCCTAAATC
TTTGCATACAGGTGAGCAGTTCTtctctgtttgttcctttgctaTCTCACTGCAGTACTCT
TTCCAACTGTGCTTTGTAGACTATACAACTACTTTGGTGGCTAC
>TCONS_04213841
AACAGACGGAAACTGCCTATGGGCTCTTAGACCGATCCTTGCAGTGCTGGC
ACTCTGCTAGGTCAATGCCGTGGAAACAGCAGAGCCCCATGGGGTTATGGG
GTACCAGCTCCCAGCCCCTTGGGAAGGCATCTCAGTGCCCAAAGGTGCCA
GGGGAGCATGGAGTGCCCTCACTGTGATGATGATTAACGTGGCCCCATGGA
TGGAGGGAAGTGTTCTCAAGGCAAGGATGGGATGATGGAAGGATGTCCTG
ATCCTCCCAGGTGAAGACTTCTCCATGCTATAGGGGTGCCAATGCCTGGGA
CACGCTTGCCCTGTCCCCATTCTCACATCCAAATGAAGTTCATCCCAACTCG
GAGAGCAGATTGTCCATTTTTCCACCCTGTGCTTTACAGAGAGCCCTGGCA
CTGAGGGGTGCCAAGGTGCCCAAACTGCCCCCAAGAAAGGACTTAGTCCC
CTGCCACTGGGACCAAGGAAAAGTCCCCAAACACCCAGCATGGTCGTGCC
CCTCCCATGGGCCACCTCCTATCCATATACAACAAAGCCTGGCAGCGAATTA
ATCTTCAACTGGGCACCGAGGATTGTTGCTGGAACTTTATTACATCGTGTCC
TGCCCAGCGCAGCGTTAACCATTAACCCGGTGGCTCTGATATCAGCTCCAG
CACCCTGCACTTCAGCTATCCCTGACAGCATCTTATCTGATCGCACGGCCAT
AAATCTGGGCATGGAGCTGCCTGAAAGCACCCACGCCTGCAGCAACCCCA

GGGAGTTATCGTGGggtgagaaggaggaagaaggagcagGTGAAGGTGGCAACGCCTC
GCTTCTCCCACGTCCTTCGAGGGtgtcctctgctctgcagtgggaACGTTAGGAGGGGA
TGTCTTCACGGCTGGCACTTCTTGGCCAGGTGCTGTGGATTGAGTTcgctttgtttt
ccattcacaCCCTTTATGCGCGCAATCTGTAACAGCTGcaattcttcctcctcctgacaACTGT
CACTGACCCCCCACCAAAGGTAAttaaaaggagaggaaagctCATTTCCTCCAAAG
>TCONS_04220554
caattttattttatttttttaacatggtGAAAGGACAGCAGATGTTGCTCTATTTCACATCTTGT
GACTACTttatcattttccatttctcattttatctaAAATTCTTGGATTTAATTAATAAAAGataatga
gaagaaatacatCAAATCTGTCAGAAAGATGGGGAGAATTGGGGAATACATATACT
CGCCTGATCAAGGAGTCATAACAGTCTTATCAGAAACAACACCAGATTGCT
TGTTAAAATGGCCGGAAACAAATGCAGATTTAGTCATCTAAGAATTACTGTT
GAGATGAAATCAAGAAAGATTGGAAGGTTTTTGGAAGTAGTTAATTAACCT
TTCAGTTGTATGATTtggatggttttgtttttttacttgtCCAAAAGAGAAGGATTCtccaggggaa
gaaaaaagttccccatattttttattgttttgcttaTGGAGGAGCACCACTGTCTCAATATGACTCT
TTCCATATTTTAGGATAATCTCAGGAAAGCAATAAGCTACATCATAGCTTTAT
TTGTTCAAAAGGGACACTACATGTCCCTATCATTCATATTTCACTAACAAAG
TAGTAAGCTGTGGGGCCgttatttctattttcctgGAGATAAATGAACACTCATAAACAA
TCTGTCTTCTTCCCTTATGgctacttatttatttaagtcTGGGTTTTGATGTAGCCACTATC
AATAAAAACTGGTTAAGCATAATGACCCCTTGGATACAGAAGACGCGGTGG
TTATCAAGAGGCCCTGTGTACAGTCGGTGGGTGAGTTTTGGTTCCCCAAAT
ACCTTGAGGTCCCTGCAGATTGTAACTTTCATGGTTAGCCTGCAGGTTCCCC
ACCGTGCACCACTTCCTTGCCTGCATTTGACATCTGCTGTAATACAATGCTG
TTGTACAATAAGCATTGGAGGTGACTCCTATAAAAAGTGCGAAGTTGTGAG
CAATGTCTGTTGGAAGCTTCACTGTGATGTCCTCCAAATGGAAACACCTGT
AATCTTAAATTACACTACCATAAATAGCAAAGAGATTAAAATGGGATGTGCA
TGTAACTATCAAAGGGAAAGGAAGCTCTTAAGTAATCGATAGATAAGGagata
tttctgtattatttaatTGCGACTTTCAGCTATGTTAGGGTAACCATAAGTAAGGCTAAA
GCCAAGTTCTTGTGatgcatttgcatttctcttgcatgtatttttcaaaaactgaaagttTGCATAAGGTG
CTAGGATAATACTCTACTatggaagaacaaaacacaaatgtgCAACATAAGTGCCCTAGG
ATCAGTCAGCAGGTGAACTTTGAGAGTTTGTGAAGATTAGCTACAATGTAA
AGGTAATACATTTATATAGCTCCTGCAAAGTATCTGAAATCTGAAGGATAAA
ATTTTTTTGTTAAAGCAAAAATGCTGGGAATTTACATTGACATATTGTGTTG
AAAGTGGATGAAAGTGTTTGGAGTGCTTTTGTTGAGCTAACTCTGGAGTGG
GGATAAAAACATTGCCCCTGTAGGTCTCACAAAACTCTTCAGAGTGAGTAA
GAGTTATTGCTGCTAGTGAACAATAGAGAGGTTGATGCCAAGGGAAGTCAA
ATGGTTTGCCTGCCCTAACAAAAGAAGGGTTCAAACCCAGGTCCTGTGCAT
TCCTGTTCTGTTTACTACATCGTACTGTTTTTTCAGATGTATGCAACAGTAGA
GTTGATCACTGTTTCCAAGGGAATTTGGAGAGTCTCCGATTGAAAAACATT
TACTAACTAGAAGTTAAAAAAGGAATATGCAATCTTATCTGCTTATAAGTGG
CACATGCAATTgcataaaatgtttaaatattctGACAATTAGGAATAATGATGTTGTGTATG
CCTTCTTCAAATCTGACCAGTTAAATCTTAGGCTACTTCATGTTCATATATCA
GGAAGATAAGAAATACTTGCAAAACTTTACTGTCTCCTGTGCTTTATCCTGG
CTTTGattttacttacatttttctctcattaaaaTGAACTCAAGGTATGATCTCATGATCTTATGtc
ataatttctttctgaaacataCTGGGTGATAACATGCTGTCTATTAAGTGGAATTTTGACT

GGTGTAGTGCTTTATTTTCCTACTCTTTTCACTGGAATACCAGAGCAATAAG
GTGGTGCGATCAGTTGGCTTTTACCACTCAGCAAAAATGAGAAGTAATAAA
TTAATGCGTCAAATAATTAAAGCTATTAAGTACTTCATGATAGTTTTCACCAA
CAATATTTATTGCTACACACAGGCTAGCTCTAGGTATCAGTGTGCATTTATCT
TCACAACAGGGCAGGCAATTGGGTAATAGATGGACTGCAGATGGATAATAG
ATGCTGGTCTTTCCTAATGATCCTTTTCAGGTGCACTAGCTTAGCTGCAATA
CACTGCACAGGTATAACCAACAGGATCAACTTAGCCAGTGCTTTAGttaaaatta
cctttttttaagcttataTTATCACGTCATTCAGCATAAATGTCTAAAAGACACTTGTAC
CAAAACGCATTAGAAAATTGCTATCTTGGTTTAACTATACTGCCTCTTAACA
AAATTTTGAAGGCTCTGATTATTGATTTGTCTTTCCAAAAGTGATGTTTTGG
TACGTACACAATTAAAATTCATAATATGTTTTGGGATTGACTGATTTGAagatgg
acagacagactgaTCTACATTTTTTACACAAAAATATTATGCAGCTGTTACAATTGA
GTTAATTTGAAGGTAACAGGATTGTAATAAAATTTTATGAGTTACTGTCAGA
AAAGTTCTTCATACTGTCACATGGACTAAAGATAGCATCACTGAATATGGGT
ACTGATCACACTTTGACAAAATATTGAGAGAAATGAGATATCAAAGAATCC
AGTTACAGGGCTCTTAAAAGAGTAAAAactatttccttttgtacatGACAGAGGGAAAG
TTCTTGCATAACTTCTGATGTGCAAAGGTTAGGATTTAAATTAGAATTTAATT
ACTAGTGAAGACTGAAGTAACGTGAGATCATCAGGGAATAGGTAACTGCA
AAATATGATTTCACTTAGAAttacttgctttattttatttacatgtattttCTCCAATTTTCAGAAT
GCCAAGTTGTATCAGTGTTGAAAAATAAGACATCTACATCGATCTGGACAC
AGTTTGAGTATGTTCCTCTATAATCAGTGTGTTATGTTTCTATAGAGATGTCG
TATCAATACCTTCAGCGTTCATGATAAAAAGAGATGATAGCACTACTCAAAA
AGTCTTCCACTCACATGTGAGTCTGAACAGCCAAAACTGACTTTTTTCTCC
CACTTATTCAGAGCCCTGAAGATAGCAATGTTACTAACTCATACACTCACTT
TTATTTTAGATATGGAAGCGTCACACACAAAATGTTcaatttttatgaaaatgtatttctaaaat
gcttattatttttattttttttaagtgacagTATATTTCTTTGAAGGATTcttagagaaaatgaaaatagagtTCT
GCTAAATAACATTCAGATAAAAAGCTGAATGTCCCAATAAGGTTATCTTTTC
CAGTCCACTCCTTCCTGTGTCCCCAGACATCTCCCCTATGCAGGACCCAAC
CTTACAGACGCTGTACATCCGTAACTCCTATTGTGTTTGTCCTTCTCTTTCGG
CAGCTTTGGCAGAGTCTGCAATGGAAGTCTGCCTTCCTCTGATCACTGATC
TCCCCCAGGACTGCACATATTCCTTTGCACCTCCTCCTATGTGAAGAACCAG
TGTTTGCAGCATGCCTGTGTAACGTGTTGGGTGGTGGATACATATGTACACA
TGACATTGGAAGATGCGAAAAGGCTGTAATTCCAGgttGCTCCATTTTCACAA
TGTTTGACAGATTTTAATGAGGCAGTGTTAAGAGCAGTCTGAGACTTTATTC
CAACTGACCATGTTATGCTCTAAAAGACCCAGGATTTCAGCATGTGTAGTA
AGCCACCATGCTTCCACTGAGGTTAATGGAATCAGCGTTTTGAATCACCTG
AGAATCAGGAAGAGGCATTTTATGATATGAAGAGAAAAGGTAAACACAAA
GATGTGAAGGCAGAAGATACCTGTTCTTTGAGGGTAAGTGATTGTAACAGA
TTCTGTTCTGGTAGGAATGTTATTGACAATTTAACAAACATTAGGTAAGCTT
TACATATCAATGTGCCATTAGGGCACAATGTTTCACATCATTAAGATAGGTTT
ATCTGTATATTACTCAATGGTTTTAGAAATGCTGAATGGCTTCAGAAGGCTAT
ATAAATGCACTTTAGTTATTTCCAAGATAGTCTTCCTTTATTATTTGGAGTAG
TATAGTAATCTTTGTGGATATCTGTCATTAAATTGATGAGACTGTATTTATCAG
TGACATTAATAAACCTTTTACATTTCCCACAGTGATAAGCAGAGGTGAGAA

GGAATGTAATGCAAAACTTATCAGAACTTGCTTTATGTTGGTGTTTGCATTT
CTCTCATCTCCCCGTGGGCAGATGGAAGATGCATTGAGGCTCTTTCTCTGCT
GTATATTTTCCACATGTGTATTGACCTAATTAAAGTAAGACTAGGGCAACCTA
TTTCCTCCCACAACAGTTACTGTAGGGGAATATATCTTCTTCAACCACAGCC
CTTTTCTTTATTGTGATTAGAATATTAGCATTCAGCTTTCAGCTAGCTAAGCG
AAGAAGACATTAGCTAGCAGAGCTCATTTTTTTGGCATCCTCATTCTTcaaacttt
tttcttcaagtgGTCTTATCTTAATTTTTGTAGACCCATGTAGACCCAGAGTAACTCC
TCTATCACCCAAAACTGCAGACACACTTGGATTTTGGCAGCGCCTGATCTC
AGCTGCTGTTGAAGGCAAAAGACATACAAATTGATTCTTGAATCTGAGCAA
CCTGCTGGGAATCTGGCTTTGTGTGTCTGAGAATCACTTCTGCTTTGACCA
CCTTTCGCAATTTTTCCCCAGTATTAGGTTAAAATTCAGCGAAAttgaaaatgacaa
aatgtaGTTGAACtgggaaggaaaatattaaaaaatacaatggaaaatctgtgtcaggaaaaaaaaacattgcaaga
aagacatttgtCATCTGTCGTATCGGTGTCTAATAGCCATTAATTACAGATGACACT
TTGAACATTTGGCATGCTGAGGAAGAGAAGTTACCTTTGGCAGCTGGATatcc
agaaaaataatatagTCCATTAGCTAGCAACATTCATTAATGAAGCTGAATGCTGTAA
TACCAAAAATATTGTGTAAGATAGCACAATTATACTGTGATGGGTGCCACTT
GAAAAGCTGGCAATTCTGATGTTAATCAGAtgtacaaaatatttatgtaaatagaCATAGAA
TGGAGTTACATATTGAATGTAATAAGTCACATATGTTTAAGGTGTTTATAGAA
AATCTTGAAGGGCAAAAGGCTTGGAATTTCATTAGAAACAGATGGCCCTGT
TCTTAAACATTAGAAGTAACTAAGTTGGACGAAACAGACCtgcagcaattaaaaaga
agtatttttgaaaaaaatgtgtggCCAGTGCTATTGAGTGCTATAGATACATAGTGTTAAAC
TGGCCACGGAACAAGAACCTGACACTGAGTCAGCAAAAGCTTTCTACTG
AATTATCATATTAATATGTTATAAGTACTGTGCCCATATTGTGTtctaccaaaaaataaa
atttaaatcaaGCCTTTTTTGTAAAAGTGGTAGGTCAAATCAGAGCACAGACCTG
GGAACATATAGTCACTCAGTCTAGCCTGTGTCTTTCCCAATACGCAGTTGAC
TCAAGTCTGTAACCTAGAAAGGCCAACAGAACATCCAAGCTCATTGTCCAA
TGCTGTATGACAGGCTGTACAACACTTCCCATCACCTGCCAAATATCTGAGA
CGTTAAAAAGATCAAAAGCCCCAATTCCAGTGTGCCAAAGGAAGATGGTG
GGTATTATTATGACCTTGCAGCAATAGGTGATTAAGTAACATTTCCAGACTAT
TTTCACAAGAACACACCAGGCTCCagggtaggaaaaaaaaactagGTTCTGCTGATCCA
GCTGGGTAGGAATCTTCCTCCTAACCTCGATTTGGTGACTATAACGGATGGA
AAGATAACAGCAGTAGTCATCTTTTCACTGAGATGCATTATATTAATGAAATT
CTGtgaataagttaaaaaaaaaaaaaaaaaggggaaaatgagcTGGAGCTTACTGCTATCATTCTGG
GTTGACAGTGGCTACAGAACAACACTTAATGCATTTATCATTGGTATTTATTA
AGTTTTCACAGATATTAACTGGGGCTTAAGACCAGGTGACCACAAGGTTGA
ATCTTACATGTGAGACAATAATCactatttcagtatttgttataaaagcaaatgaaatgatgTATTAC
TCTGGGTCTACTTGGAGGTTTTTGATGCTATTCATGCTTACcagtgtatgcattcactgA
AACAGAGTTGTTAGGACTTGATCAAGAGCTTGTAAAAATAAAGTTCTTCCA
ATTCTGCTAAATTCCACCACAAGCGTGCCCAGTGAATTATTTTTGATATTAA
AATAGCACGACTTATTTAGAAATTGGGTTGTCCACTGAACGTAATTTGGAAT
TCAACTGACAAATCCACAAACatttgatatttcattttaggaaataaaatttatttctaaaaaattaAGAG
CATAATGGAATAAAATATAAGCCCACTGTACAGTATGTTGCAT
>TCONS_04332034
CTGATTGCTGCTGGTTCACCATCAATACAAAGGCCAGCgactttcttttcccccacagC

TCAAAGGTGGGCAGGAGCCATTCCATCCACTCCAGTCTTGGcaggGAAATGT
ATACGTGCAAAGTATGAAAACCTCAGAAGAACTTAAGTGCTCTTCCATCAG
ACAGTCTGACTCAAGCCCACTGTACCATAGAACCAGCAAGCAGCCCTAGC
CATCGTGTCCATGCCCTACTCCTAAGACTGTCTGTGAAGATGATTAAAATCT
GCAGGTTCTGATGATGGTAGACATCAACATTAACTAAAGCTGACAGATGTA
AATCAGCCCATAAAGTGTTTATTCAAGAATAACAGCGATAATTTTCTTATCT
ATAAACATTAAAGGAATATATTCACCTTTATActaaaaaatgcaaacaaatagAGGACAT
TTTCTGCTGGTTTGCACATGTGCACATTACATCTCCTCCTCAGTACTCATTAC
CTCATGAAAGAACAGACAATTACAACTGAAAGCCACCAGTACCCTTTCTGG
TATGGATCACATCCTTCTGTTCTGAAGATAACCACACACTGCAAAACCCAA
GAACACTGAGTGACCTCACCTTCCTGCATGCAACTCTCAAGCTGtatgaaagctg
aaaaacatgCCACGTTATGGGAGAAATGGTTTTAGATGTAGAACCACGGGACTG
AAAATACAGGTCACAAATACACCAAACTCTTGAGAACCTGCAGGGATACC
AAAATCCAACTTGCTGTCAAGAGCTGCCATCCTAAAGCTTGCCCAAACGAT
CTCACCACAAGCAGACTTCTGCTCACCTTACCAACAAGGCTGCAGCAAGT
TCCTGctcttaaaaatgcattatctGTCTGCCCACCCTTTTGATAGTTTCATTCAATAATC
AGAAGATAAGAACAGGACTTGATTGTAAGACAGTGGGTGGATgagagtaaaaaaa
tatatatattaaaatggaTGATGTGAATAATTAAAACCACTTTTCTATgtactgaaagaacagaaa
ccaCAAAGATGAAAGTGAAGTGGCTTGTTTTATTAGTTGCCTAACCAATCAAT
ACCATCCGTCAGCAGCAACTGTATAAACACATCCTCTCCGCAAAGCCTTTG
TAGAGCCACCTGCACTCTGATCCACACTGAACCTCAGTGACATTTCAAACA
TTCTCATGAGAAGTTTCCTTCGTTCAGTGAATACAGCATAATGCATGGCTGA
ACAAATTACTGGAACATATGCAGTGGTAGTTTAGGATTTTCAAGCAGTAGTT
TAAGGGTGCTTTGTTTTAACATTTGAAACCCCCATATTCATTTCCATCCATGT
TCCAACCGAATTTCCCAAAACCTTTGCATACTTTGCAGTCTGACTCACAAC
CAGCTAATTCCCCAGCCACTGTAAATCAGTTTATCTGCCCTGAACTCAGTGA
GAGAGGAGGATTCAGCCAGGATGAGGATTTTAGGCCACtgcattttaaagttttctttagA
GTGCCTTCAGCAGTTATTTATTATTGAGTTATGAggtctgtttatttttgtaacaTTTGACA
GAGTCAGAGGGAGTGAATTGTCATAGCAGTATGCAGGTGGTTATCTGCACA
ATTCTCATTAACAGTAGTATTTTTCTTGGGATTTTGAAGTGCCAGTCGAGAT
CATCAGTCCTAACTTTCACGCTTCTGCATGCACAAACCAGAAGCAATAGGA
CACAAGGAAGGTAAAGGGAAATTACCGTGCcaaaaaaatactcattttatCACTGAACT
TCATAAACTCCAAGCAATGTGAACCATGATATTTAATGATGTATACAAAACT
CAGACAGTCTGCCCTCAGTCTCGCAGTTTGCACTGTAGAGCTTACCCCTTA
CTATAGACTTTGACTTTAAATATCAGAATGTATTTAAAAGTCTATATAAAGAT
AACATTCCACCAAAACAAAAGGTTTACTTTCCTGTTTGAGACTACTGAGCA
CTTTTCTGTTCCTAAATAACAGAAGCGCAACAACCCAGAGGTGCTCAGTGC
ATGCACTTAACaggcaaaaacaaaagacagaacaCACTGCATCACAGTTTCCTCCACCT
ATTTTTTCCAGAGCACTAATTAATTTTGCTGCTTCGTTCTCAGAATTTAATCC
AAAAAACAGGATCCAGAGAAGTTACACAAGTAATataagaagtatttttctatttttcttttcctt
gcctgCCCAACCACACACATAAGAACACCACCCTATGGATTCAATAACATCTA
ACTTTTTCTCTCTACACAATATTGGGAGATTCCTCACCAACACTTTCAGTGG
CACATGTGCTTAATTCTCAGGTTCTAACATTCATCATCATTCATCAGTACTTT
GTCATACTGCATTTTAACACAGGTAACACTGGAACACTAACAGGAACATAG

AAGGAGCAGAAATCCAAGAGAACATGGACAGATCTGCAAAGCATTCGAGT
GAAACtttcaatatttctttcttttatcgCCATCAAAAGAGAACGTTCATTACTGAAACTTCT
GTCATCAAGGCCAGGAGAAGAAAACCCCAAGCAGAAGCCAACTCTGAAA
CATTCCACCAAATGCTTGCCTTAAGTGAGTTACAAGAAGTACTAAATGAGG
AAAATGGGTTGAAACATTAAAAGTGTGtgagaaaacttgtttttctagCAAATTTAAAGG
AATTCCTTGCCAATACTATGAGATTACCAGGCTCCACACTGCCTGGTTAAGC
AATCCTATGCCTTTTGCTTGCATAAAACTTTCAGTGCAGTAACCACCTAATA
ATTCATTGCTGGATGTgtcaaaaaacagcaaaacagaaaacgCTATAGGCAGAACATGCA
AAATCTCCTCATAAATCAGTAATTATCTTCTTTCAGTGCACTTCCcagaacagaag
cagcattACTGGAAGCACAGGCCAAGCAAATACCTCAAATTCTAGCCTCTTTCT
GACCTTATCAAGAgtataaaacacttttttttttttttttttttggttactGAAAATGTTGTGGAAGAA
GCTTATTCATATTTAGAACAGcttcttttggttttgaaatatCAGTTCTATTTGGCAGCCTT
GGAATCGGACTTTGCGTTCCCTCTGCATACACAAACACATGTATAGAGTCTG
GATTATTTTGGGAAGTAagttaaatacagaaacattgCTGCCTGCATGAGGTATTGTTAA
CTTTCCACTGTACAGCTGAGAACAGTGTTCAAAACTGTGTGGTCTTCTAAT
GAAATAAACACTGGGAAATAGAACTAAAGGATATTTTTTGAATAGAACAAA
TTCAAATATTCCACTGCCAAGATCGTACACTGTACAATATAAAAATGCCTTA
AACGATCACATCCCACTGTAGTATAAATCATTTTGAGAAAGCACTTTTTTCT
GTCACCTTGCCTGACAAgcattatgtattttatttgtatgcAAGTGAATGGCAGTTTATTGC
GTCCCAGAGGATGAAGTATTAATGATCATTTGCTCCCTGCTGTGTGGCCTGT
CATAATTTTGCAGCAAGACTGACAGCAGTAACAACCAATCAGTATGTTAATA
CTCACCTGGCACTGGCTACTCCGTCCATTTTAACCAAGGGTCTTTGCCAAG
CCCTTAggtaattttcattaaaaaatgcaatttctgaTAGCATCCTTAGATTGCTAGATAGAAGC
AGACTAAACAAACcatatatttcttttgtgtAGCAGTCAGACTTCTATCTCAGCAGGTG
TTTCCTAGAAAAAAAGACTTCCAATCTGAAAGCCCATTTTGGTCAGCAAGA
ATCAATCTCTAAAACATTCAGCAATTTGTTATATCAATATCTTAACATGGTGA
ATTCCTTACAGCATATAAGAATTAAATCCATCTCTGACAAGTAAATAGAAggc
agaatttttttaaatgatagtTGAAAGTCGAATGTCAAAAAATCCCACAGATTTATACACT
GTGATGtcattcatttctcttctctgcctgCTTCATTTTACCTCGaggcttgcaggaaaaaaaagccacaac
agaTAAAGACAGTTGTTACCAGCCCTGACACACAAATAACCTCACATTTTAG
TAGTCATTTCGTATCATACAGCCCTGCAGTAGACGCACTCTGCGCtattaaaaaaa
gtttcaagAAAATGATTTGAGTGTTTGTGAGCACACGGCACGCATGATTACAGG
GGAGATTAGCGGATCCTGGTATTGACAGTCAGTTGCCATAAGCTTCCCCCAT
CCCCAAATCAAGAGCTGTGTCTTTCCAAGCAACTACATTTTATTCTTATTCA
CATGGAACAACGCAGAGTCCCAACAGCCTTGGAAttagcctgaaaacaaaatcctgaCA
GGAATGATCCTTGGCAACACCGTGGGAGGGAGAgagcaaaaatgagaaaaatcccTGA
CAGGTGCACATCAGCTTGAGACAAAACCCTGCCTGCTCGGTGATAAACAG
ACTGTGACAAGGTGCAATGTGCCTGATGGACCCTACCTGCAGAGAGCAGC
GTTACCAGCCACCTCCGTATTGTGCGCTTCCTAGACCTAATGGCAGTTAATA
TCACTCAGGTGTTGCAGCTATAATGTTTTGATGTGAATTAAATAGATGCATC
GCATCAAAGGCACTCATATAAATggaataagaaaggaaaagatacttgtttgctgcctgctgttctTTA
TATGCAAATCTTAGTATATCACATGGCAACATACTTGCCTTCCATAGCCACAta
aatgttttactgttttatgaGATGTTTGCAATAAATGTGattgatgatgattttttttttcataattgcCTATTA
GAAATACACTATTTTGGTGACAAAACCTTTAGGGATATCTCAGTTACAAAAC

CGAAGCGAATGAGAGGTACCTGCACCAAAAACTGGGTGGTGCTGCTAACA
AACGCCAAAACGCACCGTCAATCTGCCGGTTCATTTTACTCATGCATTTCTT
TAAGCTTGTTAGCATCCTTTCCAGGTTTACTGAATTTTGCATTTCAATCAGAT
ACGAAGATGTATAAAAATACTGCTACTGTACCTTAGGGCAGGCAAATAGCTT
TAAGCTACACACACAAATAGTCACAAGGGTGAATAGCTATAATTATATTGGA
GTGCATCCAAGAAATCAGCTATTCTGTTTATACTTATTGCCTCTTTGAATTTA
TGTGCCCTTCAAGCAAACATACTGTAAACTGATTTTAATACTAAAACTGTTG
ACCTTACTATTGCAGCTTGTTGACTATTTTTCATGCAATTGCAGATGTGCTGA
AAAAGATATAAGCATCAGGGTAAACATTTATAGCAGAAAAACTCCTGGACC
ATTTATAAATATTA
>TCONS_04333493
CTAGAAAAACCTGTGAAAGGGAAGACTACCTGTTTGTTTCTGTCTACTGCT
TTGCCCTCGTTCCCAAGtcctgggaaaaaaatctcGGAAAACAATTTAACAATGTCTT
AGGAATAAAATATACATCAGGACTTCATCTGGTGAAAACTGTGGTATGTACA
TTAAAATATGCAGAACTAAGCCAAATGAGAGTGAATCTACTGCAGGCATGT
GTGTACATACTCAGTTTTTTGATTAGTGATCAGGTaattgaactagatgatcttcgaTGGT
CCCATCCAACTATGCTATGCTATACTATACTATTCTATACTATGCTATTCATGA
AAGACACAGTAGTTTAGACTACAGAATTATCTTTAAGAAATATACAGATCTG
ATAATAGTATAATAAGTGAAAGTACCAGGGGCAATGTAAGACTGATTTTAGG
TTACCAGCAATACAACTTCACTTATTTGTGATGCTCCATGACAGCTAACTGC
ACTTTAAGTGGCTCCTAAACAGTATAATGATCTTCTCTAGTTGGTCTGCCTT
GAATAAGTTATTTGGTTAGTTGTTTTAGgttgaaagatgaaaatgacTCTTCATTTGAAT
TCTGAGAACCCTAATTTATTTGAACTTTGATGCCTTCCGTTTTTATATGTGTT
TATTTCCAACGCAATATGACTGTGCTCATTAACCTAAAATATCTGCCTCTCTG
CTAGCTGTTCAGCTGGCCTTTTCAAACATATGGCTGTTGTTTTAGCTTGTTAT
GATGTGGCCTGTCTTGTCTTTGGCTGGTGTCATTACAGGTGATTTTCATTGT
GGAGTGTCGTTGGGATGGTAACATGAATGGGTAGGGTTTTAACTTGCAGTG
TCAGTTAGAAGGGACTCTTTCATTAAGTCATATTTTAGTGGCGTTGATAATTA
TGCTTTTCTGGATATCCCTAAACAAGGTGCAAGTAAAGTGAACTCATCAGA
ACAACTAAGACTTGTTTACCTCTGGAAGAGGTGAAGAGAAACTGAACCAA
TACGCAGCATCGTTCGCTGTGAAGAATTCTCACAAAAGTCAGTAGGAGTTT
TGAATAAACATTAACAGCAGGGTAGTGTCCTTAgaggttatttctgtttcagatgaaTCTTT
CTGTGCAGCATTACCACCTGCGCTGTCCCTTGtttgataaattaaaaagaagcatcgcaaatatt
ttaataaaaatcatagaatgctGCCAATGTTTTAAAGAACGAGGCAGCgctacagatattttaaattca
gaatacTGGATGACTTCAAACTATTTCTGACTCTGGGCTTGTTGAGCAGCATGA
TCCATCCACCTAAACAAAatcaataaatgaaaaatagagtGAAATAACCTTTCTATCAAC
AACACAAACACCAAAGTCTGTACTGTGTTTTCTGGAGAAAGGTTCAGAGG
GCAAGCCATATGTTTGAATGTTCATTCTTCCCTTGCTGATCTGCAGAAAAGT
GCTCAAGTTGAGCAAGAATGGAAAGGAAGTATAATGAGCGTAATGTTTTTC
AGTGCTACCTGTCTTAACTTGCTGCATTTCCTTGTAATAATATTACAATCTGC
TTAATAGGTACAACTCAGTCTTCTATTCAGCGATAAAGAGGCTTGGTTCTCA
GTCATTCATACTTTCTAAACATGTTATTCACGAAACAATTTTGTGTAATTTAC
TATTATAGCAGAAAAATAgttctgcaatttttttttctagtaagGATATACcaattatatttttgtctttaaga
TGTGTATCCCATAGTGGATAACACATTCTCTGCTGCTGAATTATAATTGAAAG

CTCAGGTCAAATCATAGGAAATACGTCAGAAACTCTTTTCTAACTCTGCCTT
GTGTGTTGTCTTGTTTTTGGAATGTCATTCCTCATATGTTCTCgttcatttttcttgtctttt
cttgtTACAGGTATGTGTTTCGGGGCATGTGTGTTTGTCCTTCCTGTAGAATCAT
ACCAAATGCTTCCCTAAACTgttgatatttgttttcttggaacAGTTTATCTTTGTTTAGTG
GACTATTCCCTCTGTTACCTAAACAATGTCCAAGAACTGTTGAATTTACTGT
ATTATGATGGCTTATCTTCGTGTTGCAATCCCCTCCTTCAGAATAAGACAGC
AAGATGTCTCAGTTTCACGTGTTGTTGTGCCCCCAGCTGGACACCATTTAG
AGACTGctcatttattatttctgttgttctaATATGGAAGAAGCTTAAATAAGaaagtgtttatttattttat
ttttttttaaagcaacactGTTTACTTTCTGTCTATCCTTAGTCATGTTTATCCTTagttgtgtttt
cctttggaaaaagagaaatcaagtcagtgctgcagcagaatcAACAGACTATCCACGGTGAGCATC
ACTGTGTGCACCACAGGATATTAGAGTAGTTAATAAATATGTGCATTATTAA
AATAGCTATAACTATTGTAGCTGCTGCAAATTGCATCTGATAGTTTGCTCTATt
gattggggaaaaaaatgaatttccaaTTTGACAGAACTTCATTTTCCCAtctctttcatttcactgaatgG
TAGATCCTTCATTTAAGGAAGCTTAAAAACAATATCTGTGATTCTTACTACCA
TCATATGGATAGTTATCTAAGGACAGTGAATTCCAACAACAAACTAATGTTT
CATGGATTACCAAACAGCAAACAAGTTGACGATGATTCTCTGCTAACGTATT
TGAATTTTCACTGCAGCCAATGAATACTGATGCACAATGTAGAGTGctcagaaac
ttttttttttacagcttgaTATTAAGTTTTCTGTGCCCTTAGGAATGTTTGTGTTTGATGT
AAGTGCTAGAAGtctttgattattttcaaaataggaactggaaatattttccaattaAGAAATGTGTCAC
AGTTTGGAATTCTTTGATTAGAATACTTCACTTGACAATGAACAAGGTGGT
GGCTTCTGGCTTGCAGAGGAAGATGATGTTTTGCTGTAAAAAGGTAACCGT
GGAGATGTCAGTAGATGTTACTTGTTGAACATGAATATGCAAATGCAgagttcaa
aaacaaaagagataTTGTGCCTTGGGAatatttcctttgttgtttcctttcctcttttcttctgggGAGAGAGA
GTTGTTAGGAGACTAAGAATGTTAATTACTATGATAAGCTTATAAAAACATT
CTGAGTAACGCCTATTGTATGAGGGGTACTTTCTTGAGTTTATTAGCTGGTG
AACATTCTGCTGTTTAACAAAAATTCTATTCAAATGTTGAGGAGATGAgaatta
gggttttttttgctgctgttttaggactttcttttccacttaTTGTTGTCAAGGAGAGaatgaagcaaaagcaaaa
tagtCTACTTAATGAAGTGAcgaaaaagaaaatgtaatatatAACTTGGTAAATTTTCCTTG
ATAATTCTATAAGAGGAGAAGAATTAGGCGATAAATTAAGAAAGAGTGGTA
TCTTGCTTCATGGTGAGTCACAAGGAAATCACAAAGGTATTCagtg
>TCONS_04343421
CAGCTACATTTGATCTGGCATTGGACCACCGCATGTACCAGTCTTCTACAGT
TCTGAGGGGGAAGAGTTTGGCATCATTTGCCATAAATGCCTCAGGCATCCT
TGAGgattCTGGGAGTCCAAAAGCCCAGACAGCAAGAAAGTCTGAATTTAAC
AGAAGGAGAATTAAAGGAGATCCATAACAGTCAGTAACTGCTGTAAAGCT
GCTGTTTTATGGTCAAGTCGTGCTACTACCATTATCAACCAGCTATGGGCAT
CAGTAGGAGACTGCAGTATTGCCATCAGGTGGGTTAGTACTAATTACAAGG
TGCTTATTCAGTCTAAGGAAAATACACTGCAGATTAGTCACTGGCACCCAA
AATGGCATAATTAATTGGAGCAATTTATTAAGACAAATAGAGGGTCACAGCT
AAGCATGGTGCTTAGAGTTAATTTACTGTTAACAAGGGTGCTCATTTAGCCT
GCTAAGAGAATGCATCTACGTTGTGAGAGGATTTTATCATGCTTTCAAGAAG
GCAGATGCTGGGTCTTCTCTAGTGCTCTCGTGCTACGTGACCTCACTGGTGT
AAATATGTTTGCAGTATGGAAGGACTCATTCCTTTGTCCAGGTAAGATTAAA
ATGGTCTTTCTCAACtaaatttttcttcatctgactGTCTAATAATCAAAGTGCAGTAAAAa

cattctctatttttttttgttttgtatttgctgAATCTTCTATATGCCTATAATC
>TCONS_04343422
TCTAGGAAAAattatgggaaagaaaaattaatattaaatcaTGAAATAATGCAAAAGGGCTTC
ATTAAGATCCAAATGGGCTGTAATCaattttgttcctttcagaTTACTGGTTTCAGAAAT
CGAGGTGACCATTTCTGGTCCTATTTAAGATTGAGGAAGAAATGTGGAATT
CTGAAATCTTTTCAtgactgaaaaacagctttccaCCAAGATCCCCTCAAATGAGATTGC
AgaaattcttctttctccttcctccctggcTCTCTTATCATGCAATTCTCTTCATGCTGTTCACC
AAGACCAAATCTGAGCCAGCCTGAGATTTGTGCTAACGGCATCAGAATGGG
CTCCACAGAACATCCTTTGCATGGAAGGcagccagtgatgacagcaAGCTTTATCCAT
CACAAAGCTGAAGAAATAGGTCAAGGCGATCCTTCTCCCTGCACTGAA
>TCONS_04351227
ATTCTCTGGCGGGTGGTTCAGATACAAGGCCCCGAAACCTCAAGAAGGCA
AGAGGTCAAAGGATGCAACAGAGGTGAAGgaaaagagtCGTGGCAAATAAACC
TTACTATGACCTCCAGAAGTCAACAAGCTCTGTTTTGATGTTAGAAGGGAG
ATgaaaatagaatatatatataaaagaagaaacaaatccaAAGATTACTGAAATTCAACAGTGCTT
CAGATGATCactcttcagtgaagaaaaggaCTTTTCACTCTCGACATAGGGCAATGCCT
TTGAAATCGATGAGCTTAAATGAAGCATAGACTGGGGACATCTACCGTTCtca
tcacagctgctgccacaTCATGGAGAGTATTGCTAACACAATGGAAGAGGAATTAAG
CTGCCCATCTGTGCAAAGTAGGCTGTTTTGTCTGCTCACGGTTGATTAGTGG
GAGCAAATGGACTTTTGCTAATGTTGGGGGCACAGTTCTGGAAGATGGAA
GACCCACATTCGAGTCCCTCCTCTTGCTGATTAAGGTAAGAGATTTTATTGT
TTCAGTATATTGCATTGTTTCAGCCGTATGTCAGTGCTACTGCACAACCGTA
GTGATGTTACCCAAGTTTGAGTTTGCCCTTGTCAAGCCAAGTAGGGATGAC
TGAACTTGGTAGATTTGTCTCTACAATGCTTACTAGTTACATCCACACCTTAT
TAGAAATGTGCCAGAGCTTCCTATAGGAGTCTGATGTACTTCAGCATTCCAC
TACTGCTGAACATTATATCATCCTTCaccaaattcattttcatgacTTCCAGTGTGTTCTA
CTCCAAAAAAGGGAGGGGCTCAGGCCTGTTGGTATTTGCACAACACCCAA
TAAATTACCTATAGTTTTGATAGTGGAAGACAGCAGTCCATATTTCATCAGC
TCAGGAATCTACAGAACCTTCACTGatgttttttctgcttctttccaaaAATTGAAAGGCAA
CATTTGGGGGGGAGAGAGTAATCATATTTGTTTCCATACTTAAAGAGCACAT
GAAATTATAGACTATTTTACTCACCTGAAGGTACCCGTTTCAGCatacatttctgttct
gttcgttttgtttttccagtcaAGTATTTTgataaatttatttaatttctggTGGTGTATGGAAGAAAAGC
CTGAAGCCTGGTTACTCTGAGATGGAAATGCTGTTTGGTTTTCAGGAGTTT
CAGCGACTCGTAAAAAGAAACATGTCCCTCATATTGCAGAAGTAGAAAGTA
CCAAAAAGGTCATGTTCCATAAAGGTTTGAGTTAGAGAAACCTTGCTAAAT
CTTGAAGGAACGTGAATCACTTTACAGATCTTAGCAGTGCAAAATGTGACT
GAGTTGAGCCCCAGACGTGAGAAAGACTTGTACCTGCTACACTGCAGTCT
CAAGGGATTTCTCCCTGCTCTCACTTTTCATCAGCCGCTGCCATTGCTGCTT
TGGACAGGGTTTGCATTTATTACATTTGCTTCTGTTAGGAACAAGAAGTGG
GACTTTTCTTCCTCAAGTCCAGCTTTTATCTAGTTACTACTGCTGGTTTGTTG
CTTTATACTTCTAAAACCTCAATTGTACTTGCTTTGGGACATTAAACGAAATA
TACGTTtataaaattagaaaagcatAACTTTCTTAAAGTCCACTTGGCAGAGTTGATCC
TAGTTGTTTAAATTTCTAGTTGAATCTCTACTGAGGTCACTTTTTCTATTAtaaa
aaaatggagcagctggttagtgaggaaaaaaaagcgtCTGAGACTGGTAAGGAATGTGTTgtctgga

agcagcagcaggcaatTAGCAACAGTTAGTGGTCAGTTTTACATTTGAAGGCATTCC
TTTAGacagagccagcagctgtttgcttttgaatgGGAAGGTGCAGGAATATTTTCTGGCTGA
AGACTTGGCCTGGGAGCAAACTTTTTCAGCTTGATGCAGGAGGTTAGGGA
GTGGTTCAGTTGCATCCTGCAAAGGTAAGATACGAGCTGGAgtctccctgcttcctctt
ctctctgctgatTTAAAATTGCAAGGCCCGGAAGACAGGAACAAAAGAGTTTCTG
CTGGGCTCCCCGAAGCCGGGAGCTGTAACTCCAACGGCAGACGTTGTTGT
ACTGAGGGAGGTGGTTTAGCGGGGCAGCATTGGTGGAGTG
>TCONS_04358188
AGCACCAGGGCTCCTGCCAGATCCGTTTCACAAATCCACTCAGCCTCATCT
CTCTGCTCAAAGTCATGGTGCCGGATAGCTTTTCAGGAGGGTTTTTCACTC
CAGCACTAGCACTGCTTTCAAAACCCACTGAAGATGGCCAAATAACAGATG
TTTGATTCAAAACTGTACCCAGGATGACAGTTGCAGACTTCACTGGTACCC
CCACAGCATCTTCAATGCAGGTTGATGCCAGTGGTGACAGCCCAGAGCTCG
GCGCAAGCTTTTTCAGAC
>TCONS_04367020
CCCACCGGCTGCTCAAACCCACTGCTTTCCACCAGCAGCTTTCCCTCTGCA
CTGACCACAGTGCTGGGAAGTAGCAGGAGGGACCTCGCTGGGACACCCCA
TGCTTCTGCCCTCCCTGAACTCCTACACAGACTTTCCCTGACACATTGCCCA
CAGCACACCAAGGGCTTATCGTGCAGCTAAGACATTTATGGCACACCTTGT
CCTTCATCTCAAAACAGCTTCCCAGATCTGGAAGTTCCATCctgatgctttcattttcta
ccCCTTCTTGGGGAACTGAGAGCACTGACAGCCTCCACTTGCTTTACTGCCT
TGAagcagggtttgttttttaaggcatgcatatttttaaaacaaatcttgCAGTGCAGGAAAAGCGAGC
AGCTCAGGAAAGGCATCACCTGTCACTTTACACACATTTGATCTTAAAAGC
AGGAGCCTTTCCCCAGTGGATGGCTCCATCCACACAGTGCACATCACCCCA
CCGATGCGCAGTGACAAACCCCTGCATGCTAATGGTATGGCAATGCTTTGC
AGCTTTACCCACTGGAGCAGCCTGAAAGACATTGGGACACCTTCAGTCTCC
CGTCCCATCACACATCAGCAGTTCCACATAACGAGCTCTGTTACATGGCTCT
TCCTGCCCAGGAAGGGCAATCATGTCACctgctcagacagcagcagagactTAGGGACA
CAGgactggaaggagcagctcCCTCCGTGCTGCTGGCACGCTTGCCTGTGACCCCAA
AACATCAGCACTTGATGGCAAACCAAACTCCTCCCCACCCCAGACCTCATG
CCCTAGCTTACTGAAGCCACTCTGAGCCGGGAAGCGCTGCAGACGTGTGC
TCAGtgatgctgctggggaagcacaCCCAGGGATGGGTCCCCTGCCAGGACCCCAACA
TTGCACCCCGCTGCCTGAAGCGCTGCGAAGCAAAACGAGAGAACCCCACA
GCCATCTCCACCACTCCCAAAGCTGCCCCTCTCCCCGCTGGGAATGGACAC
AGTCCCGGGAGGGACAGCGTTTTGCTTTCACACCAGGATGTGGTTAATCAT
TCCCCGGCAGCAGCACACCGTGTACCGGACGCAGCACATCGCGCCGCTCG
CACCGAGTCCTGGTGCTCCGCAACCTCATCTGGGGCCATGCTGTGGGATTT
GCACTCTGCCCCTTTTTTATCAGCCTCGGTTTCAGCACCGGGACTGCATCGG
TGCCAACCTCCTCCTCCCGCATCGCCGCTCCTTACCTTGATGTTGCAGAAGA
TACCACGGGAACAGGTGCTGGAGCTTCTGGGGATATCCACTGGGGACTTG
GCGGCTTCGGGATGGACCCCCATGGTGCACCCCAGCAGATGGGCTGTGTG
AGGGCTCCGGCCCCGTCCTGCTCCGAAAACGGTTTCACGGGCTTTGGAAA
GACGTGTGAGGACACTGGCAAACAAGGAGTGCAGCCTCCATCCATCCCCC
AGGGCTTCGGTGGTGCTGTGTTTCCCGACAGTTGAGCACCAGGGGACGGG

CAGCCAAGTGGAAATCCAGAGAGGTTATCAGATCCTGCCGAATGCTGAGC
AGCAACAATTTCCTGATTTCCTGTGCTTGCGCCGTCCAAACGacctgggcactgctg
gaggaTTTACGTCTTACCGGTATTTGCTCCTTGcacaactgaaaaacagctttctccTCCAAA
AGAAGAGGAATGATCCTTCttgttgttgcctttttccccccctatatggagaaaaatgaagtggATGTA
GAAGTGAGATGTCTCCAGCGCCGAGCATGGCCGTGCGCTGCCCCAAAGGA
TGCTCACAGCCGGGCTCCTGCCCACCCACTTGCTCCCAGCTCACTCCCCAC
TGAGGTGAACTGGTCCAGGGCCAGAACCTTGGAGGGTTCTTGGctcATAGAT
TTCAAGATTAAAGAAACACACCCAGAATGGCTTTAACTATCCAAAGGAACA
AAGGAGATTTGGAAGTGATGTATTTTTTACGAAGACTCAGACAATATCCAC
AAGTCTCTTCTTGTAATAAAACTTGGAGCTCTGCGTGCACAACTATGGTcatta
agttttctttttattattaaagtGTTTTTAACTTGTTTGGGAGTCATGCTTAAGCTCCCAGTG
TGGATAATTGTTGATGATGGGATGTACTGCTCATTAGATGAGTGTTGCACAA
GGAGAAGGGCAGTCCTAACACAGCCACGCGGACAGGCAGACGCAGCATTT
TCATTTGCCACAGATGTGGAACAGATTCGGTGCACTTCCAAGGCACAGAA
AAACCAGATCAGAACAACAGGAAACCCAGCATTAGGTTATGAACCAGAGA
AAATAcgaagaaaaaatgaaatgtcattaTATCACTGGGTAAATCAACAGTCTATCCACAG
TGGGGtccctatccctggaggtgttcagagccatggagatgtggcactgaggagtgtggtcagtgggcacagtgggg
tgggctgggatTGGACCTGGGGATCTGAGGGGTCTTTTCCTACCTCTACGAGTCTT
ATGACTCCTCATGTATCTTGCTAGTGCTGTACGATTCGCTGCCTAAAGTAATA
AAAGTAAGTAATAAGAGGAAGATACTTTGGAGTCAAAGCACCTAAGGAGG
TGAGCTGGGAGCCACTGGAAGGGTAGAGGATGTGCAGTCTCAACAGCACT
GTTGCTGGACTGAAACCCATCCTCGGGCAGCAGGTGCATCAAACAGCTCC
AGAGATGGTCCTCACCTAAACAGGCAAAACTTTGAAGATCCATGACGTTAA
CGCAAGTGCAGTTTGTTCTGACCCAGCAAAGTCCTCTTACTTCTAGGAAAG
AAGCTGCTCCTAAGTATTCCAGTTGTTCTCCTTTCTGGCTAAGAACTTTTAG
AAATTGCAATGAAGAGCCAAAGCTCAAGCTATGAGCATGGTCAGACATTGT
GCAAAAGCTGGCACCAAGAAAGGAGACTGGGTAAGGGCCAACTCCAACC
TGAGCTACACCAGTACACAAGGAGCAGGACATATGCACATATGCAATACTT
GCACATATCATGCAACATTCCCTGAGGCACAAGAACTCCCTACAGAAACAC
AACGTGATTGTATGTCAGATCCAGCCTTGTTTTCTCCAAATATTGAGTGGAA
CTGGAACTCACAGCTTCTTCCCAGAGCTCACATTCTCTGCATTATCCCAGCG
TGGATGCTGCCAAAACAAAGCCTTCACAGATAGGGCTGAGATATCCCAACT
TAAAATAACATGGGAAGAGAAGGTGATTGACCTGAAGTGGCCTTAGGTTTA
AATTAACCACACAATGAGGGCTGCCTGACATGGCTAACAGTGACCATGTTT
GGACCCTTAAACATCCCGATCCTGCCAGCAATCCCTCTTCGCACGCTTATTT
CCCCTATGCTATTACCAACTCACTGGCAATACTGCAACACAGGCAGCAGCT
TGTCCATCAGCTGTACTCTGTTCTTACTCAAATTCCTCCTCctcaaagcaaaacagtttt
aaacacaAGCAGGAGCAGAACTGACACTACTGAACCTGCCCAAGACCCTTCCT
CCAGCCCTACAAAGCAGATGGCACCAGTCCGAGCATGATGAAGAGCACAA
AGCACGTTTTGCAAGAGCTGGGCAGACAAACGGGCTGCTGTACAGTTGCA
GCACAGTTTGGTCACTGGTACAAAGCAGATATTCAGCTTACAAATTGCTCTT
CAGCAATTTTATGGCTGGCCCTACGCACTGCTCAGGATGAGCAGAACTGTT
TTTCTGACCTTGTGCAGGGTCCTGAAtcaaaaaaaagcaactctgGGCAGTGTCGTGC
CATGACATTTGCCCAATATAGATTGAAGTCTCCTGTCAAGTCAATGTTTCCA

TCATCACCTGCTTGTAGTCTATCTCACTGCTTCTGCTGCGTTTATCATCCTTC
TCATACACCCATCAAATCAATATTCACATTTACAGTCGGGTTTCTGCCTCCCC
TCCTCCGAGACTTCTAACATTTGTGCAAGGACAAGTTTAGTCAGTCCACTT
CCCTGTTCTTTCAAGCCCTGTCTCAGTCAAACACTATTATTCAGTCTGCTTT
ACAGCATTTCCTAGAAAGGTGTTGCCAGCTTCTGTGCCATTTGCATCACAG
AGGACAGAAAATTTGTTTGCTCTCCTTCTAGAGCATGAATCATCCCCAACTT
TGCTTTCCCGTATCTATGTTTCTCTCAAAACCAAGACTTGGAGGACAGCTTT
ACTTCAAATCTCACCAGCTTAGATCCTTTTTGGTTTAGGGGAAGGGCACCT
CTGGAACCAATCTGCTTCCTGGAAGGCAAAGGTATGGGTAAGGGGCTCAG
ACAATCACAAAGTGCTCACAGTGGTGATAGCAGCACAGTATGGACAGGGG
TGAGAGGTGGAGATGGacagtgcagcaggcagggcagcccCAAGGGATCAAGTAAACT
CAAAGACAGCTATTAGATGATACATCTGCACAGGGGATTGCGTGTCAAGCA
TACACAGAAGAGATTCCTACTTGCTTTCACCCAAGCCTCACTGCAGAAAAG
TAGCCAGCACCCAGACCTTCAGCtttcagccttctctgagcttattccagaggaagaagcaggaCA
AACATACTGCAGTCACTGCCTCATCTTCAATCCCTACCCATCATCCTTAGTG
TTCAGCTTCCGTTGAGCAGGCAAGCCCAGGATCTGCACAAAActtggttggaaaaa
aaaagcccccccACCACTgccaacacaaaacaaaaacacactcTCAAAATCCACTCCAGAATAA
AAACgttgaaaatgcctttttttttggcagttgTTGGACAGCTCCCCAAGGGCAGCCCCACGC
AGATGGCCTGGAGCAGATGACCCAGGTATGCGGTGTTCCTCCTATTCTTCTG
GATCACAGCACACCCCTGTCCCAAGGGGCTGCCAAAATCTAATGGGGCTTT
CACTGTACCCTAAAGAAAGGATCTGTGGTGCAAGGGGAGTTGAGATAAAC
AGCACTGATGGCCTCCCCCACTTGCTAACCAGACAGGATGGAGTCCACGA
CAGCTCAGCTCAAATGCAGCCAGTATAAGAAGCCTCACCAAGCACGTAGG
CCACGAGGCGGGCTTGCTCCTTTTGGTCCAAGTCATTTTAATTGCCACCTGC
AAGTCAGTGTACCCTGTTTGAGCATGAGGAAAATGTGGAGGCTGAGATTAC
AGACGAGCCTTCTGAGCAGAAAAGGAGGTTGggggcaggagaggagaggacagggTT
ATTTGATGCACTCAGCCTCTCCTACCCCAGGAATTCATGGAAGCAAGATAG
CTGAATCAGAAATACACTTAGGGTAGTTATGAGGTTTAACTTCGCATTTTTC
TTGGGTTTCTCAGTGGAAAATGTTTTGGAGCTTTTATAAGCCTCACATGCCC
AATTGCTTCAGCAGCCATGCAGCCCTAAAAAGGGTACTTGCTATCTGGAGC
GGACTTGGAAGGAGACCTCGCTTAGAGTTAATGAGTCCAAGCTCATTTAGT
TATGCAACTGGGGACAGTTTCTTTGCCatgaaaggaagaagacagaACTAACGTTACT
GCCATTTCCAGCTCAGATGGCTGAAACAAGAGCAAGCAAAAGAGCACCCA
ACTGGTGCAGCCCTGGTGGGACTGTATGCCACCAGCATAAGCCAGCATGTG
AGAACACATATTGAGCTCTGCCAGGTCCTAAGCCGAGTACACTCAACATCA
ATCGGGCAAAAACTGACCAAAAAACCCACTGAGACTTACAATTAAACCTT
GCCTTCTACTTTAGAGTTACTGTATGGTAATACAAGCCCCTTTATTCCAACA
GGCAATATTTAGTAAATGCTTAGCAGTGTTGGACACAAGATATTGCTTAATG
GCAGGATACAGTTACAGGTCATGATAAGCAAGGTCCACTCCCTACAGGAAC
AAAGCTACAATATAGAAGACAGATGAGCtatcagaaatgaagaacattCCCTCCACCCC
CAGAACATACTCTCCCTGCGAGgccattttgttttgctatttgcatggaggcactgcagagagaagagT
TGTTCTCCACATCTTTAccagggaggaggcagaaaTTACCAAGAGCATCACTACATG
AAATACACAAATGGCTGGAGGAATGTCCTTATCTGGTTCTAACTACCTGGA
CACGCAAGTGTATAGAAGTCTCACTCCCTTAACAGCCACACTGCAGATTTT

TGTTGCCAGATCACTCCTTATCAGATGCTCTGTGGTTCAGGTGAACTATTTG
TACTAAACCTCAATCACATCAGTTCTCACGTCAAGTGAGAGTTTTAATGTCA
GCACTGGTATTTCTATGCGGCATGACTTGCCTGCTTTAGCATTCCAGCACGC
AGATCTACCTGCAAGGAGCACAAGCAGTATGAGATTGCTGTACTCACACTA
CTCTTCCTGTGGAGTTTTTCCAACTCCCCCTTTAtaatcctgcttttttttttttttttccccagaa
tgaTTCATATCTGTCAGCTGTGGCAAAAAAACTACAAAACTCAGTACCCTTA
CACAGATTCCACACTCCCAGCagtttttctactttcttctgGAAAGCACAAGCTGACTCA
AGCCCTTTCCCATACATGCAAGTTTAGAGTACACCATGTACgtcttttccagaaaaaga
gTTGAAAACCTATTCACAAATCCTAGGACAAAGGCTGACAGACAAACACTT
TGGGGTGGAGTACTGATGCCCAGTGCcaaattcaagaagaaaattaaatttcaataAAACAA
GGTCATATCCAGTAGAAACAAGTGGCAACACCTTCACATGCTCTGTTTGTT
TGCCATGTCAGCCTCACCCTTCCATGAAAACCCAGAAACAGCTGTGGTGG
GACCAGAGGACAACGTGAACATGAAGGCTGGCCAAGACAGGTAAGAGCA
GCACTTCAATCCTCTGAGGTGTCTCTTGGTTGAGTTGCATGGAAAAACTGC
TCAAGGAGCAGCCATTTCCTCAGCATTGCTTTCCAGGTCTCTTTCATATCCC
TTAGGTATCATCTCCATAGCTCTCAACCCGTCACTGCTGACGAACTGAATTC
TCCTAAGCCTCTCCTTTGGCTTTTTTCAAGATCGGAACACTACAGCTTCCAA
AACCAATTCATCCCACTTAGTTTACTCATTAGTCCCTAAGGAGCCTTCCCCA
GCCCTCTGTGTAACACCTCCCATACAGTTTCAACACATACTTCATTTGAAAT
GGCCTTTGCAGAGGTCTTCAGGGacattattttacagaaaatccACATCAAGCAGCTGC
CATGCTCCACAGAGGTGAGCAGAGAGAAATTCTCACAGCCCATCCTGCCTT
TCTACAGAAATCTGATTCATGTTTTCTCAGTTGTTTGCCTCAGCTCTAGCCC
GCCCACAAAACATCATCTTTACACTTCACGTCTAAAGAAGTAGCAGGTACT
AACATTATTCTCCATTCAAAACGCCTGTGTTTTCAGTCTTCCACCTAAATGA
CCTCCTCCTCAAAGGTTCCCTAAAGCATCACAGTGCTAAGATTTAACACAtg
cagcagtgaagaaaaatgtcaccATGAAAGCTATCTGGAATCTTAGCTACAATAAATGCC
TGCACAGCATCTACTCAAGGTACTTCTGCTGTCAGTAAAATGCATACCTTAG
CTTCAACTCCTGTAGGTGAAGCACGTTTGGATGAAAGTTGAGGTTGTTTTG
GTAACTCTACATAATACTAAACACTTCTTTGTCTCTACTGATGTTCAAACTC
ACTTGAGAGTAGATAAGAAATTGCCTGCTGACGTAGTTTACCATACCCCCA
GCTAGAGCTAAGCAAGTGGAGCAGCCTGAAGTGCAGCTCATTTATAACCCA
TCTTAAGACCACAGCAGTTAACTGTCACTTCAGGTACCTATTAGAGGTGAA
TTCATCTTGCAAGATATACACCTTGCATGTGACCACTACAGTGCACTCGGTG
AAGCACATGCTGTCCTGCACAACGCATACCATCTTCATTTTGAGTTGTTTTC
TAATCTCAGGTAAACTTTAGATCCCATCATACCAAAGATCTGGATTCAAATA
ACTGACAGCTTTAACAACAAAGTCATCATAAGCAAGTACTGACATTGTGAC
ACTGGGTAGTTCTCCTGgatcagaaaggagaaataccacagaagtccacaaaaaaaccaaacccttaCT
GCTACCCTCCAAGGCAAAGATTCCAAAGCCAACCAAATAAATGTAGTTTCA
GTTCACTCAAGTAAACACAGCCACTCACAACACGCCCAAACTCGAGCTATA
CTAGAGCCAGCAGGAAAATTTCCTTCTAGGATAAAGCAAGACTTGGATCAG
TAGGCACAGACAAGTAGCACCAGATAGGAAAGCTCCTGCATTTAGTTCAGC
AAGGCAAAGTCTGTGAGAATGAGAACAGTCTACAATACTAATTAGAAAGG
ACTAATTGCCTTCAACTAGGTTCAGCTTTCAGAACCCtggaaagaacaaaagctgCA
CAACTCAGACAAATCCAGCTGCTCATACTTGCAGATCAGAGTCTCCTAAAA

TGCCACAATCACTATGGAGTCTGTCAGCACAATGCTCCCAAACACACAGTT
GCACAAAATACCCCAGGTTACGGGTGCTTTACAATGGCACAAAAGGTAACT
TTCCATGTTTCACACTGCACTAAGGTGCAACCTGTCTCCTGGTACAGCCTAC
AATAGATGTGGAGGTTGCATTCTACAGTTCACATGGAGAACTGAAGGTCAT
TCCATATGTAATTTTGTGATATAGAATATGAAAGTCATTGACACTCAGTGGAT
CACCACACATTTTCCTGGAACATCTTCTTCAGTCCTCTTTATATTCAATCAAA
ATAACCTGGGATGGAATGGATATTTGAGTTAATGAAGGCAGCAGGATGATAT
ATAAAGTCTCAGGAAGTGGTTCTGCAGGGAGATAGGCAACTCACCTTGTCT
TCATACCAACTTTACCCCAGCGCCAACCACCAACCTGCATGCTCAACAGAC
AGGGCAATGCGAGGGACCAAGCTTTCACATTAGATGTAATAGAAAAACATT
CTTTACTCTGCAAGTGtctttacaaatatatatttatatatttaagcCCGTTTCAGGCCATAAAA
GCCCATAAAAAACAACCAGTACCTTTTCAACAAGAGAAACTGTGATCCAG
AAGTCTGAGGTGAAAGACTCCCAACACAAGGGCTGCTTTGGC
>TCONS_04375039
GGTAGATCTGCAGGGCACAAAAAGCCCCGGACTTCAGGGCTGTCAGTGTG
ATATTCTTCAACAGATCTTTAAAATGAGAAGGGGACAGCACTGAGGCCCTG
AAGCTGAGGAAAGGGCCACAAGGAACAGGTGCACGCCTGCAGCAAGCGC
AAGAGAGGGACACCGATGCCTGTCCTTATCTGACAAAAGCGCAGTTCTTCA
CCCAAATCAACCCACTCCTACCGACTGTCTGGCCTTGGGCTCTAGAGATGT
GGGACATCATCGGCACACTGAAGGAGGGTGTGTGAGGACCAGACGCGCTA
CTTCACAGCTCCCGCTCCATCACAACCAGGAATTTAAGAAGCcacaagaaggaaa
actgaagaagcTCCTTCCTGTATGACTGCTACAGGTGCTTACTTCACTCTGGATAA
ATACAGCCCTCTTTTCCccccaacattttttttttttttttaattcctctcaATGTGTCGTAGCTTTAT
ATGAGGCTAATGGAGCAAGGCAATGCTCAAACGTCAAATTAAACTGCACA
GCTCTGTAATCACTCAGACTGACATGGCATAAAAGGATAATTAGTAGTTCAT
TACATGGAACATTGTTTGTTCAGCTAAATTGATTTCTCTCAGCTGCCc
>TCONS_04376150
TGAAATACAGGTTTCCAGTGCTCAGCTAAGTGggattgcttttctttccctgtggcCTTCTG
GCATTGCTAATACACAGGAGAACGGGATTTGTGTGCATCCTCACTGCAATC
TGCATGAGGCTGTGTACGTGTGTGCGTATATATGAATGCATGAAGACAGCCT
GTAAATCAATTTCCTTTCACCATGATTCAAGGATAAATTTCTTCCTGACTGA
AGAAGCAGAAGCCAAAGATAAACAACTAACATATATCCTTATGGAGCCATT
CCTTTGCTCTAACATCCTGTACAGCAGATACTAAACTGCACAGTGCTATTAG
TGGCTGCATTACCACAGCGCACAGAATGCAAGATGGTCTGaccttgctttctgctttgtg
tggTGAAGTTTTCACACATGTGCAAGCCTGAGAACATAAGATGATGAAAGAG
GATGAAATGGGtgaaggaggagagcagcatcTGTAAAATGATATGAAAATGTTCTTCT
TGGTAATTTCAGGCACATGAGAAAGCATAACAAGGAAAcaacttctcatttctgtttcaaa
tttgCAGGGAAAATCCCTAAATCCAAAcagttgttttctgtcttcttgaAAAGCCCAATtaagaa
tacaaatgaaaacTGACATGAAATCTTCAAGTTCTACCAACAGTTTGCTCCCAGCT
CACAtgagaaaaacactgcatggAGAAGGGGGATGTCTGTGTGACGGCTGTACCGTC
AGCGTGTTCCAAAAGGGTGTATTTTCCATATGCTTTCTCTGAGACTTCTTAG
TGACCTCTGATCGCCTCTAGGCTGAGCAGGTTACATGATGGCTGCTTGTGT
GTGCTATTACAGTTTGTCATTGCTAGCATTCTCCATCTCTTCCAGAATCAAG
AAGCATTGCCCTGCGTTTTCTCCGGCCCCTAATTCTCTTTTGCTCCGAGTAG

ATTTCCTTTCTCGGCGTTGAACCTTTCATCATGAACCTCTGTTGGACCCCTC
ATTGCCACTGAGAGCGGTGTTGCCTCCAGCCCTGTTTCTAAAATACCTCCC
CTCACATTCCCACACTTACATCTGTCAAGGACACCAACAGGTGacaccttcctcctc
cccatcGCGTCAAGGCTAGAAAAGGCTGTGGCAAGCAGAACGCTTACAAACA
AGGCCAGATTTGGTGACCAAGGTCcatgatgcattttttatttataccCATGCACGCACAC
TTGTGTGTATGTTACAATGACTTCAGGGGACTGGAGGGGACTTTCCGCAAG
GATTAGCTGAGTGTGCCTTCAAACAAGGTAATACAACGATCTTGCCCCGGC
GGCTGTGTGCTTAATTGGGGCCTTTTGTCACATACgagctgtttttaaaagagctTAGG
GAGGTTAGTGAAAAATACTCTCTGTGTGCCATGGCTCTTGCAATCCTAATGC
AGTTAAAACCCTCACAGCAAAACGGAGCAGGTTTGTAATGGGTCTTTGTTT
GAAACTGGTGCCGTGTTTCTGCTGATAGTGCGAGGTTACGGGATGATGTTT
GCTAGTTGCCATATAGACGTGAAAGATGAGCCCTTGCTCCAAAGAGTGTAC
AGATGCAGATGTTGGGTGGAATCTGAAGGAGGGCGGAGGAGGTCCGGGGA
TGGAAAAGTATGCAGTTTGTTCCAAGAATACTTCTCTCCCTAAGGGCAACG
TAGCAGGCATTATGGCTGAACTGGGtcctgaagagaaatgaagagggAACGCTCTGGA
CTTCCTTCGTGCACTGTCCTTCCCTTTACAGACAGCTTCTTCTCCAGCTGGA
CCAGctgcTACCTCTGACCAGTGAACTATGGCATCCAGAGCTCATACAAACGT
TTAGGTGTTCGTATGGAGGTGAAACAGTTCCAACAGAACTCCAAGAGAAA
CCCACCCAGTCAAGTACATGACGGTAAGAAATGTTCATACTGACTTCAGCT
GAGGATGTGTGTATCCCAGGTCCTGTCTCTGGCTGTTCCCCAG
>TCONS_04376152
GCACCATCCATCCCAAGCACTGCAAGGTAACTTGCAGGAACCTCAACAGG
ACCTGTGTTGGAGCCTCTTTCTTGGGCTAGCAGGACATGCCCATGGCAACC
AGACTTTTGGTAGAGAGGTTGGAGATGAGCAGGCAGGCTGTGGTGGATGG
CTCCAGggTGACCGGTGTCCCGTCTTGCCACGGAGGTCAATAGAAGTTTAG
CCGCTGACATCATGAACAGGATTGCAACCCGTATCTGTGATTAGTGATGAAA
TGAAACCCACAGCTTGAAATCATTTATCCATCATATCCGATCAAACACAAAC
ATCCTCTGCACTTGCAAATAAATGAGGGTTGGAGCCAGCTCGCCTCTGCAG
AGGTTTGTGACATGTGTGATAGATTGATGAGCTGAGAACAAGCTGCCACCC
TCCACCCCCATCATCGATGATTTTccagcctggggaaggggacacTGAGGCCAGAGGT
GAAGGATATACTTTCCAATAAGTGTTTCAGTGGGAGGTGAGAAATATGAGT
CCCCGGAGgggcagaagggaaaaaaaggaaggcacTATTAAGCAGGGCAGTTTAGAGAG
AGCAGCTGCTTGACAGACTTCTGGCACAAGGGTaatggcagtggcagcagccc
>TCONS_04382499
GAAGGAGCCACTCTCAGCAGACCCCACTCTCCCCTTCTCATACTTGGATAA
AGACAGGCAAGCCTGAATGTGGTGTCTCACTTGTTTTATTTCCCCTGGATTA
ACACACAGCTCCAGTTATCaccacagcagcaaagcagcccctgggagcagcacagacCAGGGC
CAAGGGTGCAGTGCAGGAGCCAAGCCCTGGCTGAGTTTTGGTGGGGCACA
GAGGGGATAACAGAGCCCCTGGTTCTGCTCTCCAGCTGCCACCCCCTTCTC
CCCAGCTTCTCTTGCAGCTGTGTTAAACAGAGGGTGGGTAAGCAATTACCA
GGAGTGTTTTCTTGGGAAGCCAACCTGCTGAAGCACTTGAATGGCCTGTAG
GCAAGGGAGGTGACGTCCAGGAACCAAGGCATGCCAGTGGCTCAGAGGC
ACTCACAGTGTTCTCCCCTGCAGGTGCAGCAGTTGAAGAGATGGAGCAAA
TCAACCTTCCCTCACTCTTCCTGGGGGCAACACCACTAGGTCTGCAGCCAC

AGCTtggcaggagggagagggagagagatcCATCAGCACACCTGAGACATTTCTCTAGC
AGAGGCTAAAGAGAAGCAGCTACTTCTGGCTGAGGAAGATAAAAAGGGTC
CTTGGAGGGGGTACAGCAGTGCTCGGGAAGGACAGGGAACCCCAGCAGC
AAAGACGGGCCCAGCaggagatgctggcacagggaGGAGGGGTCCCACCGAGAGCT
GTGCTAAACACAGAAGAGGCACTCCTGAAAACTCCACAATCATATTAACCT
GTTAATTTATACAGCATCTCTGTACAAAAATAGAAAGGTTCTAATAAGATTaa
caatattaaataaatatttaatagaaaaaaaaaaaaaaagtctttggcCTAGTTCATAGCTCGTCC
>TCONS_04423005
CTAAAAATGACCTAAAAGATGGCACAGAAAGACTGTTTCAACTATGATCCC
AGCTTTGTAATGACTAGAGGTGATGTATTCAGATTATGGAAATGCATTTAAG
CTGTCTGAACATACATAGTTGTATCTCAGCAGCCAGGCACTGAATGAGCAG
GTACCTGCTCCTACTGCTCCAGGAAACAGCACACTACTAAGGCAAGGTCA
GCTGAAATGTGAATGCATTGGGGGTCCTTGCAATGAAGGGAGTTCTTCAGT
CAGCTGTAAGAAGTGGTGTACTCAGGTGTCGGTAAGAGTTACTCACATAGG
ACCAAGAGGATTTTCAAAGGTATGAGAGTGCCTGTCCCACGTTTAGAGGA
AAAAGCTGCGTCAGTAAAGCAATATATGTGCAGTTTCTCAGCTTGATCCGA
GCCAGCAGGAGATGCTAAATGAGAACCCCAATTGTCATGTTACAGAGGAA
GAAGCACAACTGTTGTAGTTTCCTAACCTCATTAGTTGTTACACAGACTCCA
TCCTCTCTTCTCGTAGTATAAGAATGTGGCTCTTACATGCTGTTCATTTCCAA
GCACTTCATTGCTCTACCATCACAGTGCTTTGGAAACATACTGGAAAGCTG
TTCTGGTCGCACTTCTCCAGCTTCTCATGCATTCTAGTCCCCTGTAGAAACA
ATAGGCACAAAATAAGAATCCTTTCAACAAACAGAAGTCCTTGAACTTAGA
AGCATGCCAAGCATGATGTGTCAGGCATTAAGGGTGACTTACAGGCAGCTG
GAGGGAGGCTCAGGTTGTGTCTGAGGAGTATAGACCTAGAGTGCAGTTCA
GACCTTGAATCCTATCCTGTTCCTCACCTGTTTGATCCTGTTACCCTTCCTCT
TGGATTTTTCTTTGGGAGGCATGCCAGATGGCAAGTCAGTGTCCTTTAGCTC
TGCAGAGATGAAATCATTAAGAGGTTGGAGAAAAATAGCACCTCCTTCTCT
GAGCCCTGGGCATCACAAAAGgcatttcattttgtgaatCTGTGAAACTTCACTGCAC
TTCCAGATGtttattgctgctttcttcccatGTGGTCCAGATCATTTGAGGTGTAAATGACA
GGAGAATGAAATTTTCCTCCAAATTTGTGGCAGTTTTTAGTGTGGCAAAGA
CTGGAACTTCTGAGCAGTGAGACTTCCCAAGTTGTGCGATGAGTTACCAAA
TCTACTGCAATCTCACAATAAAACCCCCACCATCAACCACAACCACACCAC
AGGGgagagaaagggggaaaagggggggaggggaaagagaGACCAgacaatattttcctttcctctaaG
GCTGAATGGTTAAGATATGTGTAATAAACTTGTTTTTGAAGCCTGGACTACA
TGAGAACTACCATTCAGTACTTCGTTTGGCTACTAATTAATGGGATGACAAG
ATGTGATGGCCCAAACCcgtatttaaaacaaagttatAGTGAAGAGGGTGCTCATCGTA
CTCATGATTTTGAATAGTTTATTAAATGAAAGGTGAAGCATCAGTTTTAAAttg
tacaaaaggaaaaaaaaaaaacctcatatTGTAAATGTACAATTTACAGAATTACTAGCAAAA
CCAATCAAGGAGACACTCATAATACAAAAACCTATTGACTGCAAACTGAAC
TGGAAACCCATTGCAGGTACCGTGATAATAAATGTTTGTTATACAGTATTCTA
CAATGTTAAATTAGGAATcagttttccacacagaggacTGTGAAGCACAAGGTTTGTCT
GAGGCTCTGACTCAAACCAGGCAGCAATTGTTGTGCCTCTATGCTCACAGA
ATTACTGTGATGACAATTTAATGCAGCCATCCCAGCCCACCCAGCACCATGA
GGATggacagaagaggaagggagtTAATGTTATTTTATGGAGCTCCAGCCACTTCTCCT

GTTTGGTGTGTGACCCAACTGACATCAGAGGAATGGCTGAATCTACCAGTC
CAACATTTTTGGCAACCATTTCTGTTTAAGAACTGCACatgtaaaaacaaacctcagct
gtgtgaaatgctggtttgttctttttaggtgtttttcccctcccagcaAATAAGCACTGGGTATTTacgttgttttg
tttttaaataagctcattaaaaaaaaaaaagttccatgATTACTTGCTgttgcttaaaaagaaagaaaagcacctCTA
AAAAactgctgtgctccaggtgGGTCAGCCTGCTTTGTATCTCGAATGCAGAAAGGA
AGGTTAATGCTTGGGGCTGATGCCCAGCTCAGTGCAGGCTGTGGATggccagc
agctctggcagcaggcCTGTTCAAACTAAGAAGCTACCAGGTGGAGGCATCAAACC
CAAGCACAATGATATTCCAAATTAttgaagacactgaaaaatcCCTTACTACAATGACT
TTGCtgaggctgtttttttttttaatgtttttgctttttgaaaaatcatatttttctttttccaaataattacTCTTAATATGC
ACAGTTAACACTGAAAATGTAGCTGTATTCCATTGTAAGCTTAAGAACTTCA
CCATCTAAGCACAAGTTTCTATGCAGCACATACAGTACTTCTAACTGGCaaaat
taatttacaaaaaTAGCGCAGAAGGGAATTTACGGCACGGAGAGCAGAATTCTGTA
TGGTACAGATCAAATTGCTTCTAGGATGCTAAAGCGATTCCTTTCCCCCACC
CTTGAgttattgtttctttaaataGGAAAGTTGCTGTTTATGTACCTTGGGTTGGCAGGG
CTTGAAGTAGGTAGTAAAAACAGCAAAGTGATTACTTGAGAACCATTTGTG
TGTTTTACTGAGAATACTTTATTTGCTGGTATAAGTTGCTAAAAATGCACAG
AGCAAGTACCAATAGAAAACTTACTGTTTTTGTGTCCCCATGTGCTATATAA
AATGAATGTTACACAGCGTCTGTTGGAAAAGTGGCTTCATGGACAACTCTC
ACTTTATGTCCcgttataaataaaaataaatcttctcaAGAGTATAAAATCTGGGTTTATCATA
TCAGAATCTGAATTTTTTTGGCAGAGATGCACGTCGTTAGAATTCTAACCTC
TTCTATGAGAGTGTTCTTCTCAATGCCTATGAGAGGATATATGCGTACCACA
ATGTTAATCAAATCCAGTGTGTtcaaaaacacttctgtagCCAGAACGACCTGCTTATA
GCATCATTGCTAGTCAATGGCTGATAGGCATAGGCTTAATAAAAATGGCtagctt
ttaaaacataaaaaggcATATGTTAAAACCGTTTTAAATtgtacagcagaaaaataaagttaaaaagtT
CTGTTATCACtcaatgttttcagaaaacatacGTAGAACAGTGTTTATTTTGACAGCTGTC
TCGACAATTTAAAACTTCCCCctcatccaggaaaaaaaaaaatctgtattagGACAGTCAAGA
ACAAGGGatacaaatgatttttcagGTCAGCCTTTCTGAGGTGACGGGATTGGCCAAC
ATTCCTTTGTGTCATTAAGAAGTTCCTCTTCTTTCTGTCACTACAAAAAGGG
ATTGACCTTAATCATTTGGTTAAAAATCAAACCGTATCACATCAAAAATGTG
GTTTTCATACAAGCTATCACAGTATATAGGCCTCTAGCTCTAAACAATAGAG
TTCCAAATTTGTAAAATGTCTTCAGCAGACTTCAGAACATAGGCATTCCAAA
TCCACTGCATGCCTTACACCAGTTCTCCCTTTCATGTAGTTCATTCCTGTCCT
GTCCTTTCGATTTAAATCTTCTAGCTTCTGTTGGCCCAACCAAGATATCTGCA
TCTGGGGACTAACAAACAGAGCAGCGTTATGACTGGGAACACTTAACGATT
CTGAGTTGGTTGTTTTTAGACAGCATTTCCGCAAGTTTTGTGTCTGGGTATG
CTTCAGATTTAGTTATTTAAATTACATCCATCGTTGTACAAGTCTTCTGGATA
CCCTTCTGCAATTTGTCTCCTCCCACTGTGCGTGcacaaaaaaatccattaattGTTACA
ATAAAAACAGCTACATAAAATGCTAGCATCACTCATTCATTAATGCAGCATG
CACCTGAAACGAGAACGCTCCAAAAATTTAATTCCCTGCCTCAGATCAggca
gaaataagaaagaatagATCCAAGTTaataacagaaacatttcaagacaaaaaaaaaaaaaaaaaaaaaaaaaa
aaaaccacacacacacaaagtgaaaaacaaattgctttcaaaTGCATACTCCCAATCAACTTTAGCA
TCCAGTAACTTATCCATATGCAAACTCCGGAAAGGCTGTACTTAGCACCAA
CTAAATCCAGCATTAGAAAGCAATGCATTCAATGTTTTTTGTATTCCAGTTA
GAAATAGACAGTAGTAGAATAAGTGCAGATCTACAAGAGGAACATCACTTT

TTAATTATGTAATCTCATACCCTAGTGAGATTGACACAATAAAAGTGAGGAA
CCATGTGAAAACAACTCCGGAGGAAATACTGCTCTGGGCATGACTGACCA
CTTCTGTTCAGCAACTGACTGCTCCATTTTTTCTTGACCCTCATCTTCCATAG
AATTACttaggctggagaagaccttcaAGATTGTCAAGTCCAACCAGCGTTTTAAGTGT
CTAGTGTTAGATACCTGTATACAGCTATTATGCAAATAGTATACATTAGATTG
TCTACAACTGtatacattcatatttttaGGGATTTTCTATGGAATGCATAGGATGTTTGG
GATCAATTCACAAGAGGATTAGGGATCAGCAGGATCCAACTATAAGGTATG
TTGTGGCACACAAGAGACAGCATTTTAAGTGGGAAGATGAGGTGACCCTC
AGGATGTGTGTGGAGGAGCAGCTAACTCCAGGCTGAAAGGAAACTATTCT
CTACTGTTAGTTCACACTGTGAACTTCTGTGTTGGATCCAAGGAGAGCAAT
GCTGTCTGTCTTTGCTCAACAgttctctgcctgcagcattCAGCTGAGATAGGAGAAAC
AGAGGGTGAAGCACAGAGTGAAAAACCTGGTGCAGCTTCTCCCTGAAAA
GAGTAAGGTTCTCCCACACCTTAAGGCAGCTCCTGTTCATGAGGCTCAGGA
AGGTGATGCACAGCAAGTGGAAGCAGCCACACTTTCCTTAACCGATTAAGT
ATTTATTCACAGAGAGTTCAGCACAATGAGATGGGATCAGAGAGGTGCAGG
GAAAAGGCTCATGCTTCACcaagttctttgttttttggtttttgtttgtttttaaattgaggactagaaaaaa
ggagaaattgtTGAGCTACAAAGTCTTCTTTATAATCTAGAAATTACAGCAACACC
TGGTGAGGCAAACTCCTGAGCTCAGGAAGCAAGGAACATGAATGTAGATT
CTCAGTACCATAGGAGTAAGGTGATAAGATACTTTGGTTTGGATGTTAACAG
TTCATCCTCAGTTGCGCTTTCATGGCCAGCTGGGGCTTCCTGCCCATCTCAT
CTGTATAGGTGAGAAAACCCATGCCCCCACTTGCCTTGTATTCACATCTTTA
TGTGCCAACCCTCATTCCTTCCAGATAGCACCAGCTGGGAAACCTTTTGCC
CATGCCAGGCCTTTGTGCAGGAGAAcaagcagccctgcctgcagcgtGAACCTGCCCG
GTGAGCCTTTCACCACATCTCACACTGCCTTGTGGTGATACCACGTTGTTAG
TGTGGGGAAATCAGGGAGCTGAATTCATTTATGGAAACATCAAACATGCTG
CAAAAACAAAGTTATCTGTAGCCAGCTGAGATGCAAGTATCTCTTGTGGCA
AACACAGATTCATGGATCTTATCAAGGTTTCATCTGTTGTACTGCTGATGTC
TGTGCACACTGCGTACACAACTGAGGGACAAGAATAGGctataaaaattagaaaaaca
gCCTCCAACCCAGTCACTGTTATTCTTTGTCTTCAGACTGATAGAACTAGCA
CATAACAGCATCCTCTGAAGACTTTGTTAAATAAACTACTGACAGATAATAA
CAACCATTTT
>TCONS_04485995
aggggaggagggggtaACCAGTGTGTGCTGCTAGGGAGCACCAGTCTAGCTTTAGA
GTTTAAACAGGCCTGATTTATCATTCTTATTAGTGTTGTCACCACACGATTTC
TAATTCTCTCATATATTTCTGCGTACGTACTCTTAATGGGAACTGTTCTCTTC
TGTCATACCCACAAAGGCTGGCTGACTCTGGGCGGTACCAGCTGGCTGTGT
TCACAGGTAGCTGCCTTGGCAGCTCAAGTTAACATGCCTCTGATGCTGACC
ACAGGGGATCCTTCTCTTAGGGCCTGCTAGCAGCCCCTGAGGCGCCAAGG
CATGGTGCTGAGCATGCTTTCCCAGTGGACACCCAAGGGACCCAGAGCTTT
CGCTTTCCATTTCTGTAAGCCTTTAGGTAGGGACTTTCCTAATACTTGCACT
GTATTTTCAACATTACTGGCACGTAATTAAGGCCTGCATTTTGAGCCTGGTA
ACATTAACGCAGACATGTATGTGTTGGCTGGAAAAGTGACTCTTGAAGGGC
AAAATGATCCAGTTCTCTCATCCCCTAGGCTTTGGGccagtaatgaagaaaaaatgaaca
accTGCAAGAAGGAAGGTTTTCTTTCCtagctgtgtgcaggcagaccctaaaaacagaagcagaac

tgtGGACCTGATACGTGCAGCAGGTGAGGTCGTCAGGAGACAGAATGACTCT
TACTGAGCCCGTCTTGAAGACAGTCACTGCTGTAGAAGAGCACTGAAGCA
CAGTAGTTTGCGTAGCGCTGGATTTCCAACTTTGCCTTCGTATGGCATAGAA
AAAGTAAGGTCACTTGAttgcctttgtttcttctgtttcttctccttgaAGAAACAGCCCTCCTCA
GAGCCTGTAGGTCACACTGTCCAAACTGCCTCCATCCTGGGAGGGGAAGG
GCCGTAGGTCCATGGTACAGATGTGTACAGGCTTatgttgctgctgcagagcagtgtgggtT
GCCTGCGCTAAATGCAATGGGATTTACATGTGATGCTCAGGCAGTGGCAGT
TTCACACGTGCTCCCCCTCGCACCTAAGCAAAGTGAGTCCTGCCTCGTGAA
CGATGAACTGCCTTGCATCTGGACTCAGTCCAAGCCTTTCACATCCTCTGA
GAACCTCCCATGTGGTATATGCAACAGGTGGAAGGCCCTCAGCACGGAGAT
TCAGGCTCCAGCACTCAACGGCCAGCTGGAAGAAGAGGGCTGGCAGCCC
GTTTCCATCTGGTTGCTGATGCTGTGAGCCCTCTGTGTTCCTGCGGGGAGG
ACAtccccttttcctcctctcccagaGCCCACAGCTCGCAGCATTCCAAGATTCTAAAGG
CAGGTGGAACCATTCAGAGGTGCTTACAGAAGAGAGTCTGCAACATCTCC
AACTGCTTGGAGGCACTTCAGGTCCCCGAGTCTTCCAGACTGCAGAGGTC
CAGTTGTTATTACACCACAAGCTTTAAGCATTTTGAAcgtgaaagagaaaaaagaaggg
gagattTCCCACGTGTGTAAACTGGCATTATACAGTCCAAATGCCAGTAGGTGG
CACCCAAGAATGTGCAGCTCTGTTGGTTGGCTTTGTGCAAGCCTCGCTGCT
TGTTGGCAGCTGCACCTCGGTGCTGTCGGCCTCTGCTCTCCCCGTGGCCAC
GAGGAAACCTGCAG
>TCONS_04486729
GCTCAGCAACTACTTGCGCCATACATAACACACACGAGGCGCCTGGCTCCT
ACATATGTCCAAGGGGTTGTTGCGAGGGTATAAAAGACATCTTCGTTCTGC
ACCTGCAATTGCGTCCGTGCCTCTCCTGCCACAATTGGCGCACGAATAGGG
ACACGAAAAGCAGAGCGGGCTGAAATAAGCACTATCGAGAGAGCACTGA
GAAGCGCGGGGCTGCGAGAAGCCCTGTCAAGAGAGAGCTGAGAGGAGCG
TGGGGCTGAAAGAAGCCCTATCGAGAGAGCgctgagagaagagcagagatgaGAGAC
GCCTTATCGAGAGAGCGTTGAGAGAAACGCGAGGATGAGAGAAGCCATGG
AGAAGGCGTTGAGAGAAACGCGAGGCTCAACATAGAAGAAGCCTGTACA
GGGGAGCGTTGAGAGAAACGCGGCTGCCCGAGGTTTCATCGCACAGCCGA
ATCACTTCTGCAGGACGGAGGTTCTCGATACATGATGTGGATGGGCTTTTG
GGGAGAATCTGCACAACAAAAGGCGCTGGAATGGCAGCTATCGGAGCTCA
GCGACTCGCAGTAGAGCATGCAGACCCTGAGCCTATGGCTCATCCACCACC
ACAGGCACTCGGCATTTACCGTCGGCACGtgggagcaggagctgtggaAAGTTGCTG
CTGAGACATAGGCTTGTGTTTGGACCCTCACTCGTTCCAGCTGTTGGCACTg
atgcagtgtatttttttctgaacctTTTAGCTTTTTTAGCAGTTGCTCGATGCTCCAGAAAAT
TCTAAGGTAAAAGATCGTGGTACGCAGATTGAGAAGGCCTAACAACATTGG
AAATATGCTTGAAAGTTGTGCACGTCTGAGTTCCTCCAGTACTCAGTCAAA
TGCTTTAgagccaggagcacagctggaagtGGTGAAAATACATTGCCTCATTGTCTTGC
AGATGTGAGGTAAACCTCAGTAAACAGGCTTCTGATGttaaagctgttattttctatAGT
AGGTAGTGTAGTCTTTCCTACAAGAGAAGGAATCTTTCTCTtataaacagatttctttttt
ctttctgttaaaagaaaacattatggGATACTTGTGGGAATCCAAACAAAGATTCTCTTattcac
aaaaatgatcagagatTGACTAGAAGCGGATATTCAAGATGAATGCAAGTAGCTAAA
TTAGAAGACAGTATTTGCTCTAAAATGATGCATGTTTGTGTGTAGTAATTGG

CAGTGTAGGAGGCTTTCTCATATTTGATTGAATAATCCAACTTGATTGGCTG
AATATCTTACGGCGTTTAagaatgtaatatatatatttacaactGTATATCTTACCTGCAGCTG
GCTGGCT
>TCONS_04500316
TGCAGATGAAAAACACTTGATTAAGTAACACTTGAAAAACCGTGATTACGT
TTGCTGAAAAAACAGGGCTGGAAACAATGattagagaaaaagaacaagattaGCAGCA
CAGGGTGACCAGATTAGCACTTACTGAGCTGCTGTAGCTCAAAGCCTGTAA
ATCCTCCCTCTGCCCACGGTACTAACTTGGATGTTGGAGAGACTGTACTTAA
CGCTATCTACTGCTGTCACCTCAGAGCATTGCCTCATTGTCTCTGAGCCTGG
GCAGGCTGACTGCAGAGTCAACTTCACACTCCcaccctgcaggagctgctaaAAGGT
ACGACTGCATGGATGACCATGGGCTAATCCAAGTTATGACCCAAGTTAAAA
TCAATACATAGTCACAAGTTTTCCTCAGCCTGTTAGATGCtatatcaaaattaaaaaaat
gaaactggtTTTTATATAAAACCAACAGAGGAATTAGATCTAGAATTGAGGGAAA
CAACTCCTTCCCACACATCTTTAAAGTGAATGTAAGGTGTGGCTcaagaaactgaa
atgagaCCAGACATTATTGGACATACCAAAGTCAATGAGAAGGCTGCTCTTTAT
TTTGGATATGCTGTTACACAAGTAGATCActgctttattcttttcagGAATCATAAGATAT
AACATAGAAAAATTGGCCTGAAAAGGGAACAATCCCGACCTTGAAAAAAG
CTACATCAAAAACAAGCAATGCAAAAGCAGCCTTTCATCACTTTACCAGTC
TTTTGAGTAACCGAAGATGGAATTTGGATCTTTGTAGTATGTTCAATACCCA
AAACATATTGAGGTAGATGGTTTGATTTGAGCCTTGCTGCCCCTCAAGCATG
GggtggtgtttgttttgcagagctttatcagatttgcttttcaaataaacTAATAACAGTGGTAATAAAACA
CTTTTCATTTCTAACTGCTCTTTCATCTAGCATGTCAAAGCTCTTTGTGCAA
GCTAAACATTTCTCACAATGCTGAAACGCAGTTATGGATTATTATCTCCATG
GTAGTAGTAATTAAAACATATCAAGAGAGACTGAAAGTGTTTTGCCAAAGC
TGGCAAAAAAGTGGAAACAGAATCTAGGGCAGAAGCTGCTGTATAGACAG
TGCAGAGAAAGCCAAATGTGTTTAATCTGCAAGTCCACTTTCCTCTGTTTT
GGTGTTGAGGTGATGTTTAGTTTTTTTCTACCTTGATCATTATACTGGAGGA
GATTTTTGAGTTATTTTTGTGATGTTGAGTTTTCAagggcaaaaaaataaagtaaaaaaatc
gAAGATTCAGAAGAAAGAGGC
>TCONS_04504459
CTTTCACAACAACTTGATGCATTTCGACTCCAATCTTAATCACAAATTCTTT
ACAAATAAGATTTTTCCTTATTTGATCTTCAGAATTTTTTGACAATGCTTAAT
GTGAAGAAACTTCGTTCTCTGAATCTCATGCATATTTTGACTGGGAATAACG
TGTATGCTGTAAAAGCAGGCTACTTCTTACCTACATTGCCTGCAATGCCTAC
CAGCCTTACAGCATTTTTTAGGTGTCCTAATGTGGTCTTCTTGTCTGCACAT
GCAGCCAGAAAGCCTGGAACCTCTGGGAGAGCACAGTCTAAATCAAGAAT
CAAAACACCTCCCCAGTGCAACTGCAAATTGAAAAGAAGCCCTTGTACTG
TGCTGGCAGTGATATTGATGGAAGATGactttgaaatgctgaaaaggTTCTCAGATCAA
CCTGGTGACTTTTTTCAGgtgagaaaaatgttaaaaccagtatttgtatttgttgtgGTTTGTGGAG
GTCTGAGATGTGAGAAAGGGAGtgcttaagaaaagaaattatttgttttctgtataaaAACAGTAT
GATAGAGTCTGGTAATAATCAATCATggtgaataaataaaatatca
>TCONS_04538352
ATTACGAACAAAAGACCTTGCAAATGAGCAAACAAGCTCAGCAACTACTT
GCGCCATACATAACACACACGAGGCGCCTGGCTCCTACATATGTCCAAGGG

GTTGTTGCGAGGGTATAAAAGACATCTTCGTTCTGCACCTGCAATTGCGTC
CGTGCCTCTCCTGCCACAATTGGCGCACGAATAGGGACACGAAAAGCAGA
GCGGGCTGAAATAAGCACTATCGAGAGAGCACTGAGAAGCGCGGGGCTGC
GAGAAGCCCTGTCAAGAGAGAGCTGAGAGGAGCGTGGGGCTGAAAGAAG
CCCTATCGAGAGAGCgctgagagaagagcagagatgaGAGACGCCTTATCGAGAGAGC
GTTGAGAGAAACGCGAGGATGAGAGAAGCCATGGAGAAGGCGTTGAGAG
AAACGCGAGGCTCAACATAGAAGAAGCCTGTACAGGGGAGCGTTGAGAG
AAACGCGGCTGCCCGAGGTTTCATCGCACAGCCGAATCACTTCTGCAGGA
CGGAGGTGAGCAACGGGGAACAATGGGAACAAGTATAACGAAGGTGCAG
GAGGCTGTTTGCAAAataatggaaacattttgcAGAAGCACCCACGCTAACGTGGAA
AGCTCCAATTTATTCTCCCGTAATGTAGAAGTAGGTGGGACTACACAGAAAT
GTGGGACAAAGCGGGAGTGCGACTGTGGGACGCAGCTACGCGCTCTGATC
CTGTGGCTAAAAACCTCTTGGGTTCCTGGAGATCAGTTTTTGAATCACTAA
AAAAACATATGGAGCCCACCGCTCTTCCTGTGGTGGGGGCCGCTGTAAAA
AACCGCGATGGTAAGGACAATGGCCCTTTAGACCCCAGACAAGTGAGCCC
GAGGAAGGAACCAGACTTACAATCTCCCTGATCCGTGTGAATTTTGGCGAG
AGATCCGCGAAGAACcggagcaggagggagacttTGGCATCGTTCAGAGATTGCATT
CGGCTCTCGCCTGCCCTGCGATTTCGGTTTTTGGATCAGGGCTCGGCTCCC
CCTCTACCAGTAATCTGATGAGCTCTATTATGAAAAGTTACACTTTTTGTCTT
CGTGATTGCGAGACTTTGACACAACTTGACAGGTTCTCGATACATGATGTG
GATGGGCTTTTGGGGAGAATCTGCACAACAAAAGGCGCTGGAATGGCAGC
TATCGGAGCTCAGCGACTCGCAGTAGAGCATGCAGACCCTGAGCCTATGGC
TCATCCACCACCACAGGCACTCGGCATTTACCGTCGGCACGtgggagcaggagctg
tggaAAGTTGCTGCTGAGACATAGGCTTGTGTTTGGACCCTCACTCGTTCCAG
CTGTTGGCACTgatgcagtgtatttttttctgaacctTTTAGCTTTTTTAGCAGTTGCTCGAT
GCTCCAGAAAATTCTAAGGAATGGGTCTGAGATGTGTACCAGCATGATGGG
TCCAGTCATTCCCCTCATCATCTGCTGTAACATGATCACCATGCATTCATCAG
CGGTTTGGATTCCAGCTCAACCAAAACAGAATATATGGGTCACCCTCGCCA
ACAAGATGGGACAAGACACACTTTGCTTGTCAATTGCGTCACCAGGGAAC
CATATGAATTGGAATTGTTGGGATTGGTTAGAATGGACTATTGATCGTACTTC
AACTATACTGGAAAGAACCAATCATTCAGTGTTAGTTTAGTTGTTTATAGGA
ATGTTTCTGAACTGcgcctttctttttattaatagAGAAGGGGGAGATGTGGGACAAAGA
AAGACATCATGAACAAAAGACTTTGCAGATGAGTAAACAAGCTGAGCAAC
TACTTGCACCGGACATAAACACACACGAGGCCTCTGGCTCCTATATGTGATC
AAGGGGTTGTTGCATGAGTCTAAACGGTTGCCTCTCCTGCCACAAAGGCCC
ACCAATTTCCTGTTACTGAGCTGAGAAATGGTGACACTAAGTGGCCATGAG
TAAACCTACAGTGCCCAAAATAGACTCAAGAGCAGTGGTCTTGTGTACACC
AGCATGGATACCAGTCCTGCTCAGAACACATGCTATCCACACCATGCTGCA
GGACATCTAGGCCTTAGCTTGTTGGAGCATGACTGAGCCCCTGATACCACA
AAGGAGAGCATATTGCTCAGTTGGTACTGGTGTTCAGACCACTGAGCTTTA
GTGGCCTGTAGCTGTCCTGAGATCACACTGAGAGGCTGAGAGCATCTCCAT
GAAATTTTTAGCTTAGATGCAGGATGGGCACTTCCTCTGTCAGCTGTGGGC
ACCTGGGGGAAACTCAAGAGCTTGTCTGATCCAGGCCCCATCCAGCTTctttat
atgaaatatattattaaaaacaaaacaaaacaaaacaaaaaaaaacatggttCCAAGTCTTCTCTAGATAC

TGTTAAGATTGCATAAGCTTAGGGAAATAATATAACTGAAGTTATGTCAGGA
GCTGGAAATAGTATGAGGTTCACAAGGTGATGCTGAGGGAACATAAAATCT
GATGTAGTTCCTTCTCATTTCTATTGATCTGAAAGAGGAACTACATCTCAAA
TGCAGTGTACCTGATGGTTTAACTGTCCTTTTTAAGATTTATGTCTAAAACT
GGAAATAACCTGTCTGTTCAATAATTTCTTCTATTAATAATCAATAATTCTCTT
CTATTAATTCTAAAAGTGGtggaattagaaaaaaaaaattactgaattcaTTCACTTGAATGTT
TGTTTCCATGCACCAAGTAATAAATATTCATCATGGAGATTTACATAAAGTAC
TTTTTTTGCTGcatgttttatatttattactGAAGAAGATATTTTATGTAATTACAAACTTC
CACATAGTCTACCAAATTGCTTTTCAAACTCTTAAAGGGTTCACTGGAGATG
CTGCGTGCTATTGTCATTTTGATTCATAACAACCAGTTTTAACAACCTTACA
AAGAAACACAGTTCTCATTTATAGTCTGCATTAAGGGTAGATATTTGATATAT
GTCATCATTAATAAATGAAAGATATTCCTTTCAGAACCACAAAAGACACTGT
AATTAACAACATGATGGTAGGTTGGAAAATAGATGGCAAACTGTAGAAGTA
ATTGGCCACATAGGGACTGTCTTCcagtcatttaaaaacagaaatatttgaggTGTATAACAT
CAATTTAATACATGATTTCAGCTGAGGCTGATTGTTCCAAGAATAAGAGAAT
GTGTGGTTTTACAGTTACAGTAAAAATCTTCATCTAGCACAGCTATTAATCCT
AGCCTGATGTTACcatttaattaatttcattatcagatttaaattaaatatatatttgacaTTAAAGGTGAAA
TGTTAGGAGAAATAACTTGTAAAAAAGCTAATAGCCAAACCAAGGAGGGA
ATGttatattttagtttttctaaTTGCTTGATTTAACACCAAACATGTGTCTTCTTTGTGCA
TTTTCCTGTTCCCAGTTCGGTTAGGTAATCATTTCTGTGTGAATCAccaaagaagt
attttaaactGTATCCCATTAAcactgaaagagctggg
